# Supplementary material for: Electrochemical desulfurative borylation of thiols, disulfides, thioethers and thioacetals
Source: Nat Commun. 2026 Jan 15;17:632. doi: 10.1038/s41467-025-67363-7 (PMC12816636; doi:10.1038/s41467-025-67363-7)
Supplement: Supplementary file 1 — Supplementary Information [file 41467_2025_67363_MOESM1_ESM.pdf]

Supplementary Information

Electrochemical Desulfurative Borylation of Thiols, Disulfides, Thioethers and Thioacetals

Julius Kuzmin,[a] Cristiana Margarita,[a,b‡] Johannes Winter,[a‡] Helena Lundberg[a]*

* Corresponding author

‡ Indicates equal contribution

[a] Department of Chemistry, KTH Royal Institute of Technology, SE-100 44, Stockholm, Sweden

[b] Current affiliation: Department of Basic and Applied Sciences for Engineering (SBAI), Sapienza University of Rome, 00161 Rome, Italy

E-mail: hellundb@kth.se

Table of contents

General Information 5

S1 Optimization of electrochemical borylation 6

S1.1 ... Optimization of boron-based coupling partners 6

S1.2 ... Optimization of solvent 6

S1.3 ... Optimization of HBpin equivalents in MeCN 7

S1.4 ... Control experiments 7

S1.5 ... Control experiments with disulfide 8

S1.6 ... Comparison of conditions for thiols 8

S2 Reproducibility 9

S2.1 Setup for electrochemical borylation 9

S3 Cyclic voltammetry measurements 10

S4 General procedures 11

S5 Synthesis and characterization of starting materials 12

S5.1 ... Synthesis and characterization of thioethers 12

S5.2 ... Synthesis and characterization of thioacetals 23

S5.3 ... Synthesis and characterization of products 24

S6 Scaled-up procedure for desulfurative borylation of thioethers 38

S7 Unsuccessful and suboptimal substrates 39

S8 Computational studies 40

S9 Quantitative NMR (qNMR) 42

S10 NMR data 46

S10.1 . 1a 46

S10.2 . 1b 47

S10.3 . 1c 49

S10.4 . 1d 51

S10.5 . 1e 52

S10.6 . 1f 53

S10.7 . 1g 54

S10.9 . 1h 55

S10.10 1i 56

S10.11 1j 58

S10.12 1k 60

S10.13 1l 61

S10.14 1m 63

S10.15 1n 64

S10.16 1o 65

S10.17 1p 66

S10.18 1q 67
S10.19 1r 68
S10.20 1s 69
S10.21 1t 70
S10.22 1u 71
S10.23 1v 72
S10.24 1w 73
S10.25 1x 74
S10.26 1y 75
S10.27 1z 76
S10.28 1aa 77
S10.29 1ab 78
S10.30 1ac 80
S10.31 1ad 81
S10.32 1ae 82
S10.34 1af 84
S10.35 1ag 86
S10.36 1ah 87
S10.37 1ai 88
S10.38 1aj 89
S10.39 1ak 90
S10.40 1al 91
S10.41 2a 92
S10.42 2b 94
S10.43 2c 96
S10.44 2d 98
S10.45 2e 100
S10.46 2f 102
S10.47 2g 104
S10.48 2h 106
S10.49 2i 108
S10.50 2j 109
S10.51 2k 111
S10.52 2l 113
S10.53 2m 115
S10.54 2n 117
S10.55 2o 119
S10.56 2p 121

S10.57 2q 123
S10.59 2r 125
S10.60 2s 127
S10.62 2t 129
S10.63 2u 131
S10.64 2v 133
S10.65 2w 135
S10.66 2x 137
S10.67 2y 139
S10.68 2z 141
S10.69 2aa 143
S10.70 2ab 145
S10.71 2ac 147
S10.72 2ad 149
S10.73 2ae 151
S10.74 2af 153
S10.75 2ag 155
S10.76 2ah 157
S10.77 2am 159
S10.78 3b 161
S10.79 3c 164
S10.80 3d 165
S11 References 166

General Information

All reagents were purchased from commercial sources and used without further purification, unless otherwise noted. All reactions were carried out in glassware that was not pre-dried unless otherwise stated. Commercial IKA® ElectraSyn vials and caps (Ident. No.: 0040003170) were used for the electrochemical experiments with Aim TTi MX100QP as the power supply. Magnesium electrodes were cut in dimension 52.6mm x 8mm from a 2 mm thick magnesium sheet (99.95% purity) all other electrodes were commercially bought from IKA®, unless otherwise stated. THF was dried using a solvent dispensing system, where the solvent is passed through activated alumina columns, stored under N_2, and over-activated 4 Å molecular sieves when needed. Water used for reactions and HPLC analysis was obtained from a Milli-Q® system. All other solvents and reagents were purchased from commercial suppliers and used without further purification unless otherwise noted. TLC analyses were performed on pre-coated silica gel 60 F254 plates, and visualized using UV light, $KMnO_4$ (solution in a mixture of $KMnO_4/K_2CO_3/NaOH$ in H_2O), phosphomolybdic acid stain (solution in EtOH), vanillin (solution in 1% H_2SO_4 in EtOH) or hydrazine (solution in EtOH). Flash column chromatography was conducted using 40-60 µm, 230-400 mesh, 60 Å silica gel as stationary phase. NMR spectra were recorded using either a Bruker Avance II 400 MHz or a Bruker Avance 500 MHz spectrometer at 298 K (unless otherwise stated) using $CDCl_3$, $(CD_3)_2CO$, CD_3OD, D_2O, or toluene-d_8 as solvents. Chemical shifts are given in ppm relative to the residual solvent peak (^{1}H NMR: $CDCl_3$ δ = 7.26; $(CD_3)_2CO$ δ = 2.05; toluene-d_8 δ = 2.09; ^{13}C NMR: $CDCl_3$ δ = 77.16; $(CD_3)_2CO$ δ = 29.84; toluene-d_8 δ = 20.40) with multiplicity (br = broad, s = singlet, d = doublet, t = triplet, q = quartet, quin = quintuplet, sext = sextet, m = multiplet), coupling constants (in Hz) and integration. HPLC yield was analyzed by Agilent 1260 Infinity Quaternary LC (Eclipse Plus ^{18}C column, UV detector, 265 nm) with a gradient of acetonitrile and 0.1% formic acid in Milli-Q water at a flow rate of 1.0 mL/min. High-resolution mass spectrometry analyses were performed using an Agilent 6530 quadrupole time of flight LC/MS with electrospray ionization (ESI) or a Thermo Scientific Q Exactive HF Hybrid Quadrupole-Orbitrap with atmospheric pressure chemical ionization (APCI). Full analytical data is provided if the compound was previously unreported.

S1 Optimization of electrochemical borylation

All optimization experiments were carried out using 0.5 mmol of the corresponding starting material at a concentration of 0.1 M using an oven-dried 10 mL IKA®-ElectraSyn vial (**see General Procedure 1 and 2** in section S4), unless otherwise stated. Yields were determined by HPLC analysis, unless otherwise stated.

S1.1 Optimization of boron-based coupling partners

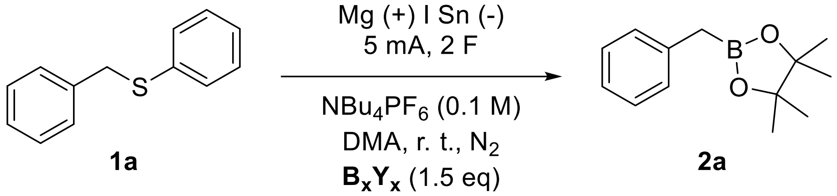

Table S1. Optimization of boron-based coupling partners

Entry	Boron source	Yield (%)
1	Pinacolborane (HBpin)	28
2	B_2Pin_2	0
3	B_2Cat_2[a]	7
4	Catecholborane	0
5	$B_2(OH)_4$	0

[a]After electrolysis transesterification with 1 mL of 2 M pinacol in Et_3N, added dropwise and stirred for 1 h.

S1.2 Optimization of solvent

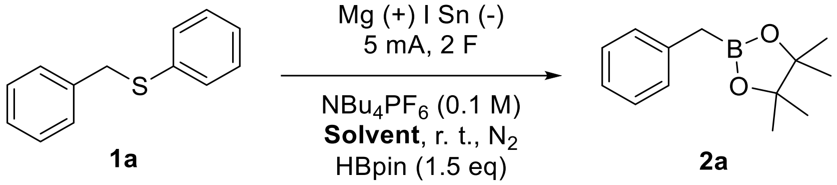

Table S2. Optimization of solvent

Entry	Solvent	Yield (%)
1[a]	NMP	0
2	GVL	0
3	DMA	28
4	Propylene Carbonate	13
5[a]	1,4 dioxane	0
6[a]	2-MeTHF	0
7	MeCN	19
8[a]	THF	47
9	DMF	14
13[a]	DMI	0

[a]Resistance too high for all/any current to pass through the system.

S1.3 Optimization of HBpin equivalents in MeCN

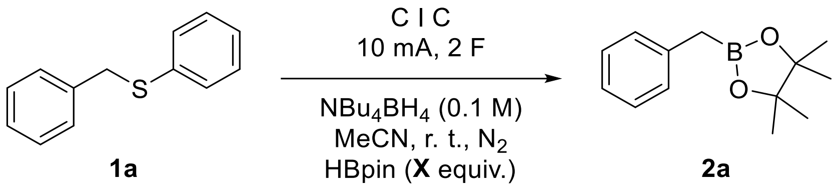

Table S3. Optimization of HBpin equivalents in MeCN

Entry	Equiv. HBpin	Yield (%)
1	2	31
2	3	50
3	5	70
4	6	65
5	7	64
6	8	71
7	10	75

S1.4 Control experiments

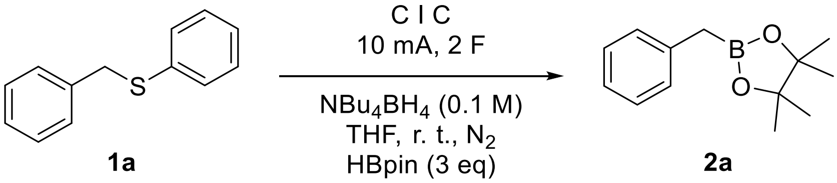

Table S4. Control experiments

Entry	Deviation	Yield (%)
1	-	99
2	No electricity 24 h	0
3	Under air	60
4	Non dried glass and THF	0
5	NBu_4PBr	4[a]
6	BDD (+)	96
7	GC (+)	98
8	20 mA	92
10	B_2Cat_2	8
13	1 eq HBPin	57
14	1.5 eq HBPin	99
15	MeCN	33
16	MeCN with 10 eq HBPin	78

S1.5 Control experiments with disulfide

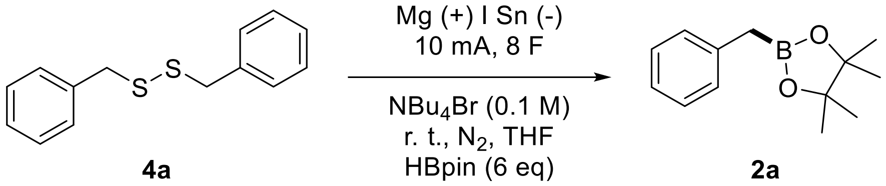

Table S5. Control experiments with disulfide

Entry	Deviation	Yield (%)
1	-	77
2	C I C with NBu_4BH_4	30
2	4 F	39
2	NBu_4PF_6, MeCN	0
3	NBu_4PF_6	0
4	3 eq HBpin	46
5	4.5 eq HBpin	59
6	C_{gr} cathode	48
7	5 mA	69
8	15 mA	38
9	Under air	68
10	No electricity	0

S1.6 Comparison of conditions for thiols

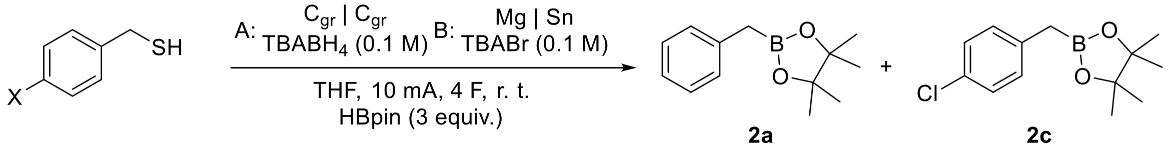

Table S6. Comparison of conditions

Entry	X	Conditions	2a (%)	2c (%)	conversion (%)
1	H	A	88	0	>99
2	H	B	80	0	>99
3	Cl	A	21	0	>99
4	Cl	B	26	0	>99

S2 Reproducibility

Four independent experiments were conducted and evaluated using HPLC to confirm the reproducibility of the reaction, see general information for more details.

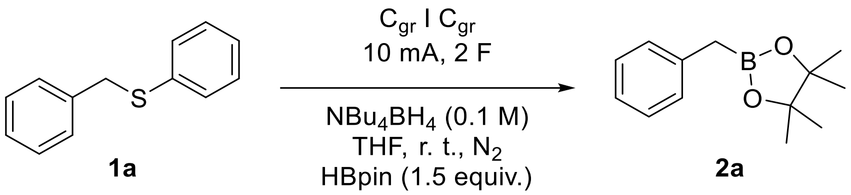

Table S7. Reproducibility

Entry	Yield (%)
1	98
2	99
3	98
4	99

S2.1 Setup for electrochemical borylation

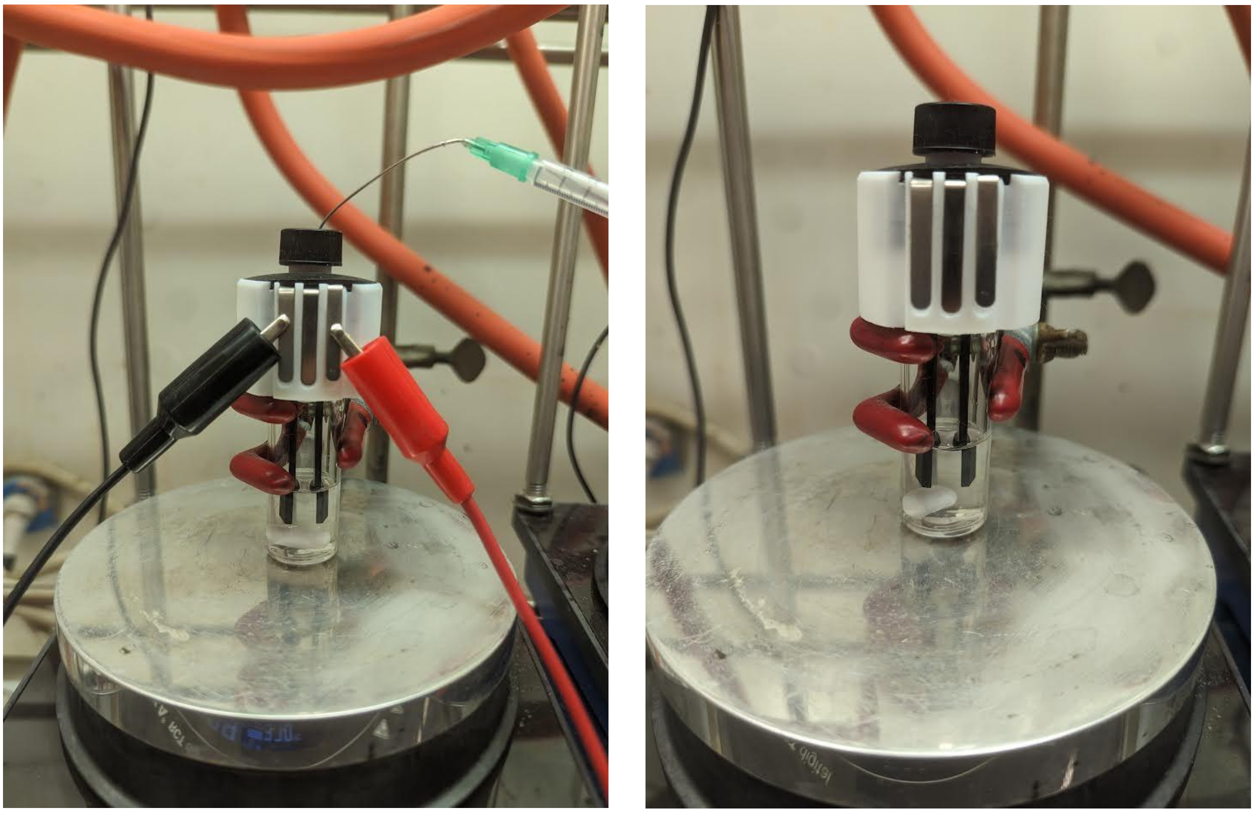

Figure S1: Left: Electrolysis setup before electrolysis, nitrogen through septum on the top if the IKA cap, crocodile clips connecting the power supply to the electrodes. Right: Reaction mixture after electrolysis.

S3 Cyclic voltammetry measurements

A potentiostat (CH instruments CHI750E) was used for all cyclic voltammetry measurements, which were carried out in a single-compartment cell with a three-electrode configuration that consisted of a 1.0 mm glassy carbon working electrode (ALS catalogue No. 002411), Pt-wire counter electrode, and an Ag/Ag$^+$ (3 M KCl in DMF) reference electrode. Prior to each measurement, the 0.5 mM substrate solution in 0.1 M NBu_4PF_6-THF (10 mL) was purged with argon for a duration of 15 minutes. The measurements were then done using a sweep scan rate of 100 mV s^{-1} and were referenced to ferrocene (Fc$^{+/0}$) by shifting the V values to the Fc$^{+/0}$ half wave potential ($E_{1/2}$ = 0.548 V, see figure S2). After each experiment, the disk electrode was polished using 0.3 µm alumina polishing powder on a microcloth polishing flannel, the platinum wire was flame dried and the reference electrode was washed with THF.

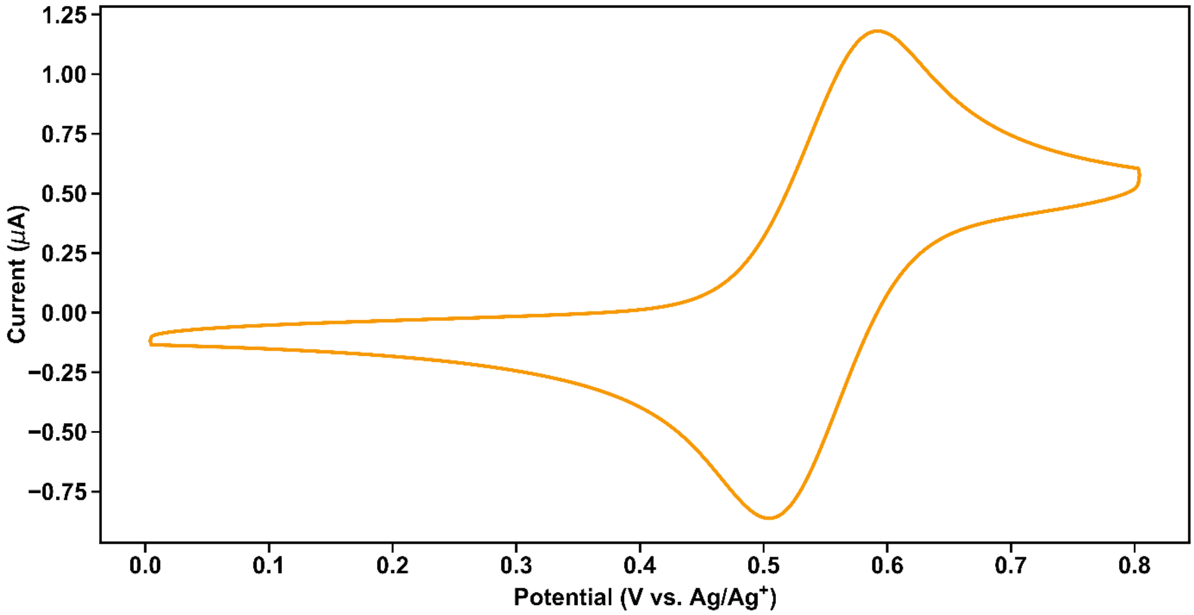

Figure S2: Cyclic voltammetry measurements of ferrocene

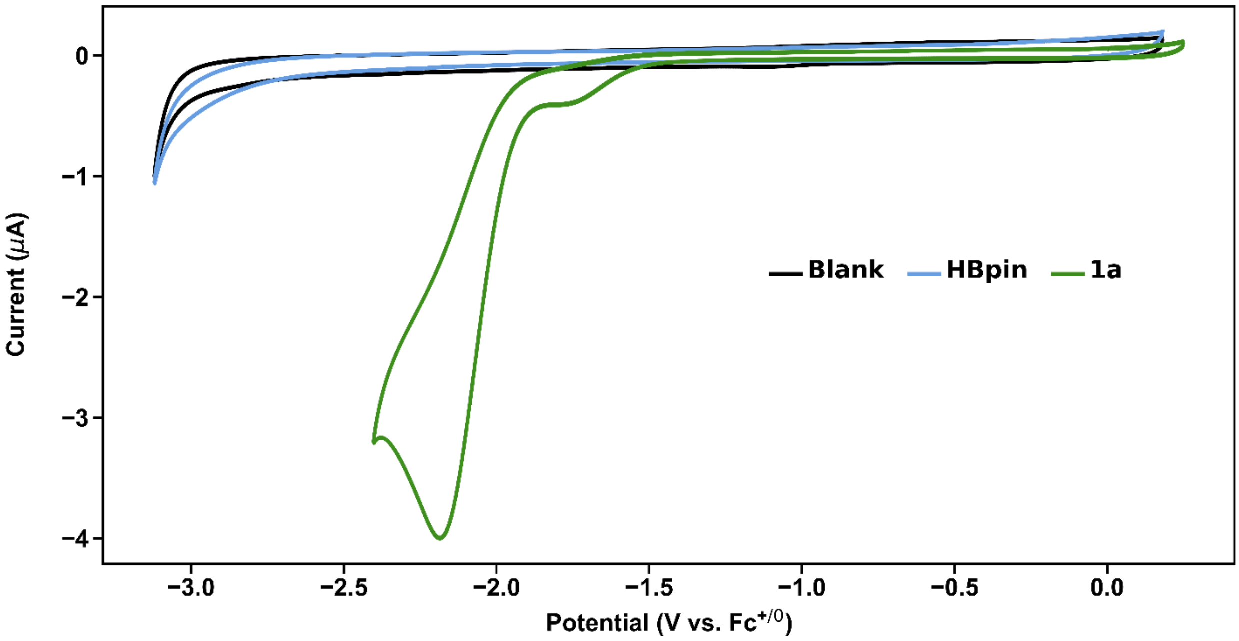

Figure S3: Cyclic voltammetry measurements of blank system, HBpin, and 1a. No clear peak was for HBpin which indicates that the reduction of it is challenging.

S4 General procedures

General Procedure 1: To an oven-dried 10 mL-ElectraSyn vial equipped with a magnetic stir bar, graphite electrodes, the corresponding starting material (1.0 equiv., 0.50 mmol) and NBu_4BH_4 (1.0 equiv., 0.50 mmol, 130 mg) were added. The mixture was evacuated and back flushed with nitrogen three times before adding anhydrous stabilizer-free THF (5 mL) followed by HBpin (1.5 equiv., 0.75 mmol, 110 µL). The reaction was carried out by applying 10 mA (≈10 mA/cm^2) at room temperature for 2 F. After electrolysis, the solvent was evaporated under vacuum, and the crude reaction mixture was dissolved in EtOAc and washed with an aqueous solution of NH4Cl (30 mL) and extracted with EtOAc (15 mL x 3). The combined organic phases were dried over sodium sulfate, filtered, concentrated in vacuo and purified by column chromatography.

General Procedure 2: To an oven-dried 10 mL-ElectraSyn vial equipped with a magnetic stir bar, magnesium anode, tin cathode, the corresponding starting material (1.0 equiv., 0.50 mmol) and NBu_4Br (1.0 equiv., 0.50 mmol, 161 mg) were added. The mixture was evacuated and back flushed with nitrogen three times before adding anhydrous stabilizer-free THF (5 mL) followed by HBpin (6 equiv., 3.0 mmol, 435 µL). The reaction was carried out by applying 10 mA (≈10 mA/cm^2) at room temperature for 8 F with a stir rate of 750 rpm. After electrolysis, the solvent was evaporated under vacuum and the crude reaction mixture was dissolved in EtOAc washed with an aqueous solution of NH4Cl (30 mL) and extracted with EtOAc (15 mL x 3). The combined organic phases were dried over sodium sulfate, filtered, concentrated in vacuo and purified by column chromatography.

General Procedure 3: To a solution of K_2CO_3 (5 equiv., 25 mmol, 3.5 g) in DMF (50 mL), the corresponding thiol (1 equiv., 5 mmol) was added and stirred for 5 minutes. Subsequently, the corresponding alkyl halide (1 equiv.) was added, and the reaction mixture was stirred overnight at 90 °C. The resulting mixture was extracted with ethyl acetate and washed with water (x5) and brine (x2). The organic layer was dried with sodium sulfate, filtered, concentrated in vacuo and purified by column chromatography.

General Procedure 4: The corresponding alcohol (1.0 equiv., 5.0 mmol), Ph_3P (1.2 equiv., 6.0 mmol)., and 1,2-diiodoethane (1.2 equiv., 6.0 mmol) were dissolved in DMF (50 mL) and stirred for 5 minutes. Next, the corresponding thiol (3 equiv., 15 mmol) was added, and the resulting mixture was stirred overnight at room temperature. The mixture was then added to EtOAc and washed with water (x5) and brine (x2), and a 2.5 M aqueous NaOH solution (x2). The organic phase was dried over sodium sulfate, filtered, concentrated in vacuo and purified by column chromatography.[1]

General Procedure 5: The corresponding alcohol (1.0 equiv., 5.0 mmol), Ph_3P (1.4 equiv., 7.0 mmol, 1.8 g)., 1,2-diiodoethane (1.4 equiv., 7.0 mmol, 2.0 g), and tetrabutylammonium iodide (1.5 equiv., 7.5 mmol, 2.8 g) were dissolved in DMF (50 mL) and stirred for 5 minutes. Next, the corresponding thiol (3 equiv., 15 mmol) was added, and the resulting mixture was stirred for 3 hours at 90 °C. The mixture was then added to EtOAc and washed with water (x5) and brine (x2) and a 2.5 M aqueous NaOH solution (x2). The organic phase was dried over sodium sulfate, filtered, concentrated in vacuo and purified by column chromatography.

General Procedure 6: Potassium *t*-butoxide (1.5 equiv.) was dissolved in DMF. Thiol (1 equiv.), copper iodide (0.05 equiv.) and 1H-benzotriazole (0.05 equiv.) followed by bromobenzene (1.1 equiv.) the reaction was stirred at 40 °C for 25 min then quenched with sat. aq. $NaHCO_3$. The layers were separated and the aqueous portion extracted with dichloromethane. The organic extracts were combined, washed with aq. NaOH (1 M) then dried over sodium sulphate, filtered and concentrated in vacuo and purified by column chromatography.

General Procedure 7: Corresponding aldehyde or ketone (1 equiv.), thiophenol (4.8 equiv.) was dissolved in DCM (10 mL) and held at 0 °C while boron trifluoride diethyl etherate (5.6 equiv., 28 mmol, 3.5 mL) was added dropwise. The mixture was stirred at r. t. overnight followed by quenching with water and extracted with DCM, the organic phase was washed with 2.5 M NaOH (3x 25 mL) dried over sodium sulphate, filtered, concentrated in vacuo and purified by column chromatography.

S5 Synthesis and characterization of starting materials

S5.1 Synthesis and characterization of thioethers

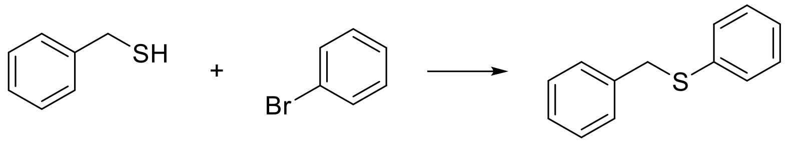

1a

Synthesis according to **General Procedure 6** with 2 mmol of the corresponding thiol and purified by column chromatography (cyclohexane/EtOAc 9:1). **1a** was obtained as a clear solid (246 mg, 61% yield). **^{1}H NMR** ^{1}H NMR (400 MHz, $CDCl_3$) δ = 7.35 – 7.23 (m, 9H), 7.22 – 7.16 (m, 1H), 4.13 (s, 2H). **^{13}C NMR** (101 MHz, $CDCl_3$) δ = 137.6, 136.5, 129.9, 129.0, 129.0, 128.6, 127.3, 126.5, 39.2.
The spectroscopic data matched those reported in the literature.[2]

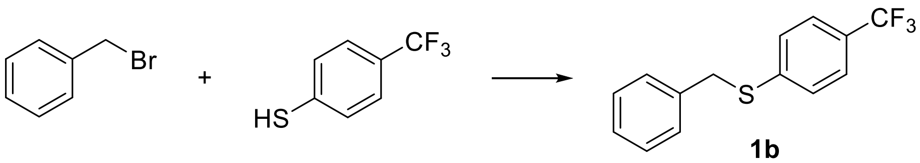

1b

Synthesis according to **General Procedure 3** with 3 mmol of the corresponding alkyl halide and purified by column chromatography (P.E). **1b** was obtained as a white solid (800 mg, 99% yield). **^{1}H NMR** (500 MHz, $CDCl_3$) δ = 7.50 (d, *J* = 8.2 Hz, 2H), 7.40 – 7.32 (m, 6H), 7.32 – 7.28 (m, 1H), 4.21 (s, 2H). **^{13}C NMR** (126 MHz, $CDCl_3$) δ = 142.2, 136.5, 128.9 (d, *J* = 5.5 Hz), 128.1, 127.7, 125.8 (q, *J* = 3.8 Hz), 124.3 (q, *J* = 271.8 Hz), 37.8. **^{19}F NMR** (377 MHz, $CDCl_3$) δ = -62.4.
The spectroscopic data matched those reported in the literature.[3]

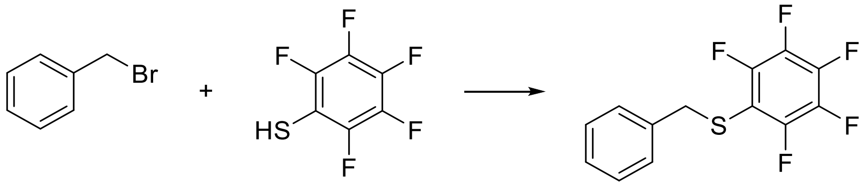

1c

Synthesis according to **General Procedure 3** with 7 mmol of the corresponding alkyl halide and purified by column chromatography (P.E/EtOAc, 19:1 v/v). **1c** was obtained as a white solid (500 mg, 25% yield).**^{1}H NMR** (400 MHz, $CDCl_3$) δ = 7.20 – 7.05 (m, 5H), 3.95 (s, 2H). **^{13}C NMR** (101 MHz, $CDCl_3$) δ = 148.9 (dq, *J* = 10.6, 4.2 Hz), 146.4 (dq, *J* = 10.8, 4.2 Hz), 142.6 (tt, *J* = 13.6, 4.9 Hz), 140.1 (tt, *J* = 13.6, 5.1 Hz), 139.1 – 138.6 (m), 136.5 – 136.1 (m), 128.8, 128.6, 127.8, 108.5 (td, *J* = 21.1, 4.4 Hz), 39.1 (d, *J* = 2.4 Hz). **^{19}F NMR** (377 MHz, $CDCl_3$) δ = -132.2 – -132.4 (m), -152.5 (tt, *J* = 21.3, 2.6 Hz), 161.3 – 161.5 (m).
The spectroscopic data matched those reported in the literature.[3]

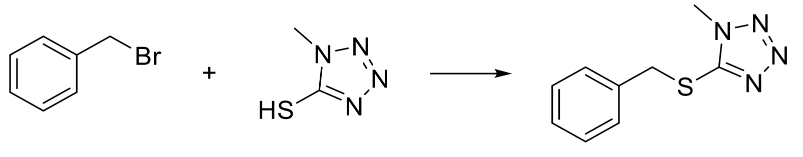

1d

Synthesis according to **General Procedure 3** with 8 mmol of the corresponding alkyl halide and purified by column chromatography (P.E/EtOAc, 19:1 v/v). **1d** was obtained as a white solid (1.530 mg, 93% yield).**^{1}H NMR** (400 MHz, $CDCl_3$) δ = 7.39 – 7.27 (m, 5H), 4.52 (s, 2H), 3.79 (s, 3H). **^{13}C NMR** ^{13}C NMR (101 MHz, $CDCl_3$) δ = 135.7, 129.2, 129.0, 128.3, 38.0, 33.5.
The spectroscopic data matched those reported in the literature.[4]

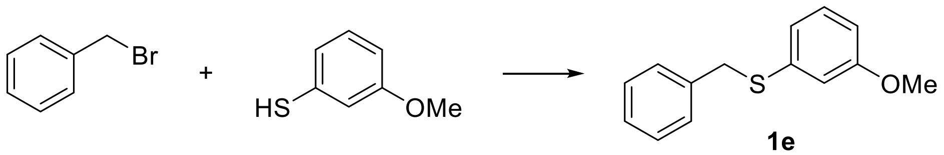

1e

Synthesis according to **General Procedure 3** with 3 mmol of the corresponding alkyl halide and purified by column chromatography (P.E/EtOAc, 95:5 v/v). **1e** was obtained as a clear liquid (540 mg, 78% yield). **^{1}H NMR** (400 MHz, $CDCl_3$) δ = 7.38 – 7.21 (m, 5H), 7.18 (t, *J* = 7.9 Hz, 1H), 6.92 (ddd, *J* = 7.7, 1.7, 0.9 Hz, 1H), 6.84 (t, *J* = 2.1 Hz, 1H), 6.73 (ddd, *J* = 8.3, 2.6, 1.0 Hz, 1H), 4.13 (s, 2H), 3.74 (s, 3H). **^{13}C NMR** (101 MHz, $CDCl_3$) δ = 159.8, 137.8, 137.5, 129.8, 128.9, 128.6, 127.3, 121.8, 114.8, 112.3, 55.3, 38.9.
The spectroscopic data matched those reported in the literature.[3]

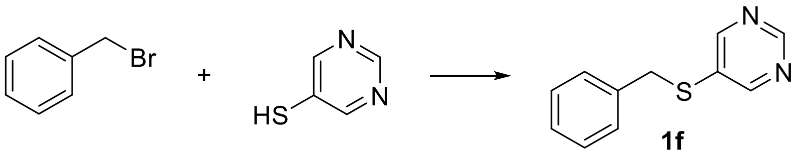

1f

Synthesis according to **General Procedure 3** with 8 mmol of the corresponding alkyl halide and purified by column chromatography (P.E/EtOAc, 1:4 v/v). **1f** was obtained as a white solid (1.45 g, 87% yield). **^{1}H NMR** (400 MHz, $CDCl_3$) δ = 8.53 (d, *J*=4.8, 2H), 7.49 – 7.41 (m, 2H), 7.34 – 7.28 (m, 2H), 7.28 – 7.22 (m, 1H), 6.97 (t, *J*=4.8, 1H), 4.43 (s, 2H). **^{13}C NMR** (101 MHz, $CDCl_3$) δ = 172.3, 157.3, 137.5, 129.2, 128.6, 127.3, 116.7, 35.4.

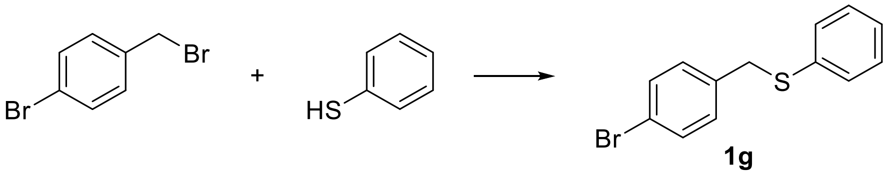

1g

Synthesis according to **General Procedure 3** with 3 mmol of the corresponding alkyl halide and purified by column chromatography (P.E). **1g** was obtained as an off-white solid (1300 mg, 89% yield). **^{1}H NMR** (400 MHz, $CDCl_3$) δ = 7.43 – 7.34 (m, 2H), 7.32 – 7.24 (m, 4H), 7.21 – 7.10 (m, 3H), 4.04 (s, 2H). **^{13}C NMR** (101 MHz, $CDCl_3$) δ = 136.8, 135.8, 131.7, 130.6, 130.4, 129.1, 126.9, 121.2, 38.8.
The spectroscopic data matched those reported in the literature.[3]

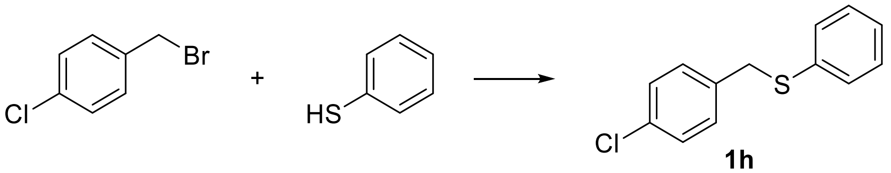

1h

Synthesis according to **General Procedure 3** with 3 mmol of the corresponding alkyl halide and purified by column chromatography (P.E) **1h** was obtained was obtained as a white solid (1 g, 90% yield). **^{1}H NMR** (400 MHz, $CDCl_3$) δ = 7.25 – 7.08 (m, 9H), 3.99 (s, 2H). **^{13}C NMR** (101 MHz, $CDCl_3$) δ = 136.3, 135.8, 133.1, 130.4, 130.3, 129.1, 128.8, 126.8, 38.7.
The spectroscopic data matched those reported in the literature.[3]

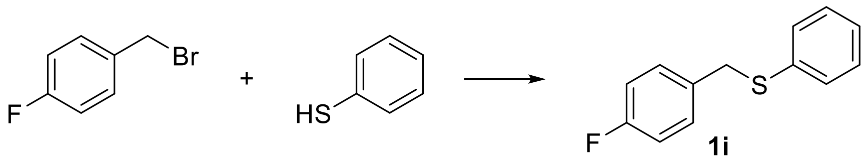

1i

Synthesis according to **General Procedure 3** and purified by column chromatography (P.E) **1i** was obtained was obtained as a white solid (1 g, 92% yield). **^{1}H NMR** (500 MHz, $CDCl_3$) δ = 7.31 – 7.18 (m, 7H), 7.00 – 6.92 (m, 2H), 4.07 (s, 2H). **^{13}C NMR** (126 MHz, $CDCl_3$) δ = 162.1 (d, J = 245.6 Hz), 136.0, 133.4, 130.5, 130.5, 130.4, 129.0, 126.7115.5 (d, J = 21.5 Hz), 38.6. **^{19}F NMR** (377 MHz, $CDCl_3$) δ = -115.4.
The spectroscopic data matched those reported in the literature.[3]

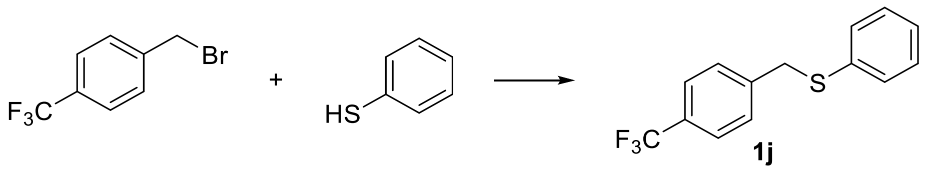

1j

Synthesis according to **General Procedure 3** and purified by column chromatography (P.E) **1j** was obtained was obtained as a clear liquid (1 g, 81% yield). **^{1}H NMR** (500 MHz, $CDCl_3$) δ = 7.53 (d, J = 8.0 Hz, 2H), 7.37 (d, J = 7.9 Hz, 2H), 7.31 – 7.24 (m, 4H), 7.23 – 7.18 (m, 1H), 4.13 (s, 2H). **^{13}C NMR** (126 MHz, $CDCl_3$) δ = 142.0, 135.5, 130.6, 129.2, 129.1, 127.0, 125.5 (q, J = 3.8 Hz), 124.3 (q, J = 272.0 Hz), 39.0.**^{19}F NMR** (377 MHz, $CDCl_3$) δ = -62.5.
The spectroscopic data matched those reported in the literature.[3]

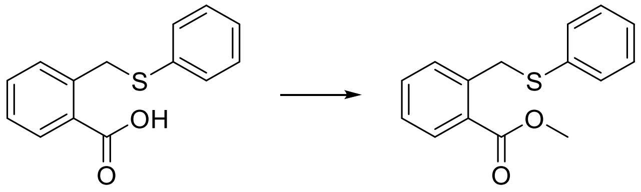

1k

2-[(phenylthio)methyl]benzoic acid (1 equiv., 3 mmol, 730 mg) was dissolved in MeOH (5 mL) and cooled 0 °C and thionyl chloride(2.5 equiv., 7.4 mmol, 0.55 mL) was added dropwise. The mixture was stirred at rt for 16 hours, quenched with aqueous solution of $NaHCO_3$, extracted with DCM, dried over sodium sulfate and purified by column chromatography (19:1 P.E/EtOAc). **1k** was obtained as a clear liquid (700 mg, 90% yield). **^{1}H NMR** (400 MHz, $CDCl_3$) δ = 7.96 – 7.89 (m, 1H), 7.36 (td, J=7.5, 1.6, 1H), 7.33 – 7.27 (m, 3H), 7.26 – 7.18 (m, 4H), 4.52 (s, 2H), 3.89 (s, 3H). **^{13}C NMR** (101 MHz, $CDCl_3$) δ = 167.8, 139.8, 136.1, 132.0, 131.2, 131.2, 131.1, 129.4, 128.9, 127.3, 126.9, 52.3, 38.1.

The spectroscopic data matched those reported in the literature.[5]

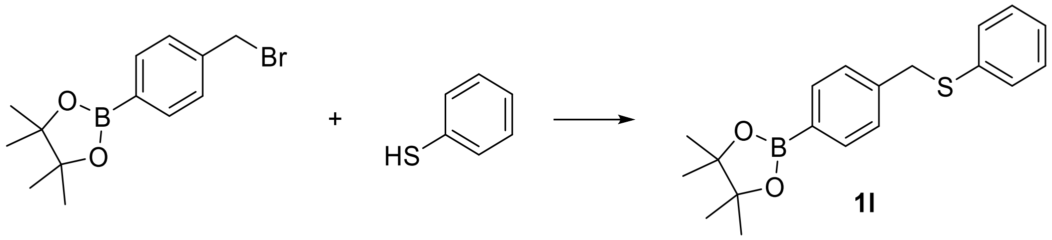

1l

Synthesis according to **General Procedure 3** and purified by column chromatography (P.E). **1m** was obtained as a clear liquid (590 mg, 36% yield). **^{1}H NMR** (400 MHz, $CDCl_3$) δ = 7.73 (d, J = 7.6 Hz, 2H), 7.36 – 7.27 (m, 4H), 7.27 – 7.21 (m, 2H), 7.20 – 7.15 (m, 1H), 4.11 (s, 2H), 1.34 (s, 12H). **^{13}C NMR** δ = 140.75, 136.1, 135.0, 130.0, 128.9, 128.2, 126.4, 83.8, 39.2, 24.9. **^{11}B NMR** (128 MHz, $CDCl_3$) δ = 31.2.
The spectroscopic data matched those reported in the literature.[3]

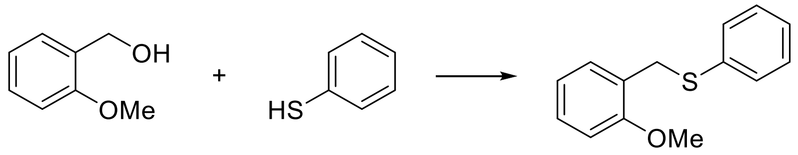

1m

(2-methoxyphenyl)methanol (1 equiv., 3.5 mmol, 484 mg) was dissolved in DCM (30 mL) then HBr (aq., 48%, 10 mL) was added. Reaction was stirred at RT for 24 h. The mixture was extracted with DCM (3x 15 mL), the organic phase was dried over sodium sulfate, concentrated under reduced pressure and used in **General Procedure 4,** on a 3.5 mmol scale, then purified by column chromatography (P.E/EtOAc 19:1) **1l** was obtained as a colourless oil (650 mg g, 81% yield). **^{1}H NMR** (400 MHz, $CDCl_3$) δ = 7.41 – 7.37 (m, 1H), 7.32 – 7.19 (m, 2H), 6.91 (ddd, *J*=7.6, 6.2, 1.2, 1H), 4.21 (s, 1H), 3.86 (s, 1H). **^{13}C NMR** (101 MHz, $CDCl_3$) δ = 157.3, 137.1, 130.3, 129.9, 128.8, 128.6, 126.1, 125.8, 120.5, 110.6, 55.5, 33.4.
HRMS (APCI) m/z:$[M\text{-}H]^+$ calculated for $C_{14}H_{13}OS$ 229.0687; found 229.0685.

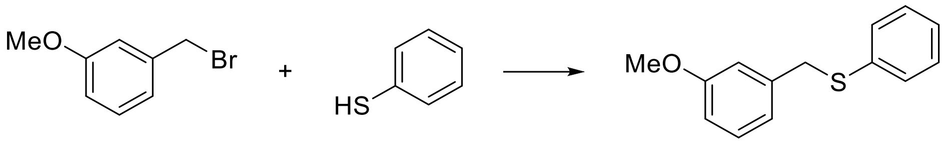

1n

Synthesis according to **General Procedure 3** and purified by column chromatography (P.E) **1n** was obtained as a clear liquid (930 mg, 81% yield). **^{1}H NMR** (500 MHz, $CDCl_3$) δ = 7.33 – 7.30 (m, 1H), 7.28 – 7.24 (m, 1H), 7.22 – 7.17 (m, 1H), 6.91 – 6.86 (m, 0H), 6.83 (t, J = 2.1 Hz, 0H), 6.78 (ddd, J = 8.3, 2.6, 0.9 Hz, 0H), 4.09 (s, 1H), 3.76 (s, 1H). **^{13}C NMR** (126 MHz, $CDCl_3$) δ = 159.8 139.1, 136.5, 129.9, 129.6, 128.9, 126.4, 121.2, 114.3, 113.0, 55.3, 39.1.
The spectroscopic data matched those reported in the literature.[3]

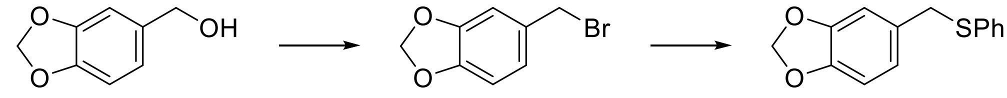

1o

Piperonyl alcohol (1 equiv., 8 mmol, 1.2 g) was dissolved in DCM (30 mL) then HBr (aq., 48%, 28 mL) was added. Reaction was stirred at RT for 24 h. Mixture was extracted with DCM (3x 15 mL), organic phase was dried over sodium sulfate, concentrated under reduced pressure and used in **General Procedure 4,** on an 8 mmol scale. And purified by column chromatography P.E/EtOAc 19:1) **1o** was obtained as a red solid (1.4 g, 72% yield). ^{1}**H NMR** (400 MHz, $CDCl_3$) δ = 7.36 – 7.26 (m, 4H), 7.24 – 7.19 (m, 1H), 6.85 (d, J=1.5, 1H), 6.78 – 6.69 (m, 2H), 5.96 (s, 2H), 4.07 (d, J=1.0, 2H). ^{13}**C NMR** (101 MHz, $CDCl_3$) δ = 147.8, 146.9, 136.4, 131.3, 130.0, 129.0, 126.5, 122.2, 109.3, 108.2, 101.2, 39.1.
The spectroscopic data matched those reported in the literature.[6]

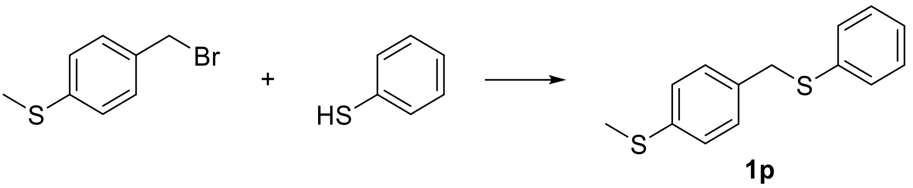

1p

Synthesis according to **General Procedure 3** and purified by column chromatography (P.E). **1p** was obtained as a white solid (1.1 g, 89% yield). ^{1}**H NMR** (400 MHz, $CDCl_3$) δ = 7.31 – 7.22 (m, 4H), 7.22 – 7.14 (m, 5H), 4.07 (s, 2H), 2.46 (s, 3H). ^{13}**C NMR** (101 MHz, $CDCl_3$) δ = 137.4, 136.3, 134.4, 130.0, 129.4, 129.0, 126.8, 126.5, 38.8, 16.0.
The spectroscopic data matched those reported in the literature.[7]

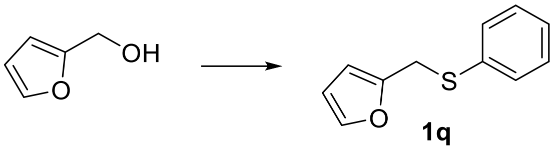

1q

Synthesis according to **General Procedure 4** and purified by column chromatography (9:1 P.E/EtOAc). **1q** was obtained as a clear liquid (320 mg, 34% yield). ^{1}**H NMR** (500 MHz, $CDCl_3$) δ = 7.36 – 7.33 (m, 3H), 7.28 (dd, J=8.5, 6.8, 2H), 7.24 – 7.19 (m, 1H), 6.27 (dd, J=3.3, 1.9, 1H), 6.10 (d, J=3.2, 1H), 4.10 (s, 2H). ^{13}**C NMR** (126 MHz, $CDCl_3$) δ = 151.2, 142.3, 135.7, 130.8, 129.0, 126.9, 110.6, 108.0, 31.8.
The spectroscopic data matched those reported in the literature.[3]

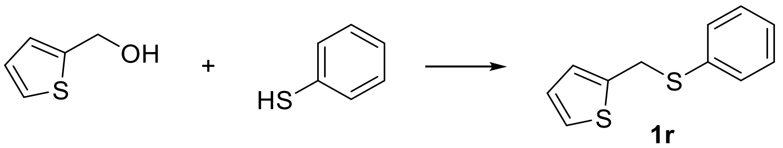

1r

Synthesis according to **General Procedure 4** and purified by column chromatography (P.E). **1r** was obtained as a clear liquid (190 mg, 18% yield). ^{1}**H NMR** (400 MHz, $CDCl_3$) δ = 7.36 – 7.32 (m, 2H), 7.30 – 7.25 (m, 2H), 7.23 – 7.20 (m, 2H), 7.17 (dd, *J*=4.6, 1.8, 1H), 6.88 (d, *J*=4.7, 2H), 4.31 (s, 2H). ^{13}**C NMR** (101 MHz, $CDCl_3$) δ = 141.0, 135.8, 130.5, 129.0, 126.9, 126.9, 126.4, 125.1, 33.9.
The spectroscopic data matched those reported in the literature.[3]

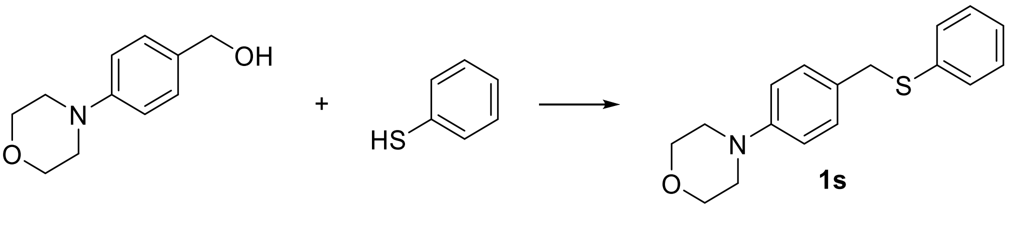

1s

Synthesis according to **General Procedure 4** and purified by column chromatography (P.E/EtOAc 3:2). **1s** was obtained as a white solid (730 mg, 51%). **^{1}H NMR** (400 MHz, $CDCl_3$) δ = 7.35 – 7.13 (m, 7H), 6.84 (d, J=8.2, 2H), 4.08 (s, 2H), 3.87 – 3.84 (m, 4H), 3.16 – 3.12 (m, 4H). **^{13}C NMR** (101 MHz, $CDCl_3$) δ = 136.8, 129.8, 129.7, 128.9, 126.3, 115.8, 67.0, 49.4, 38.5. **HRMS (ESI) m/z:** $[M+H]^+$ calculated for $C_{17}H_{20}NOS$ 286.1266; found 286-1262.

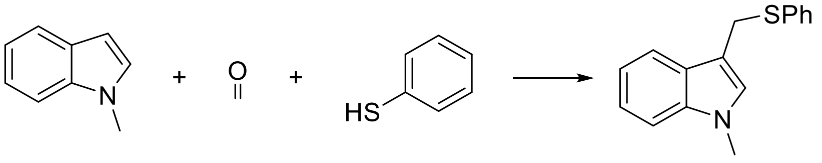

1t

25 mL oven-dried flask was charged with 1-methyl-1H-indole (2.0 equiv., 10 mmol, 1.25 mL), paraformaldehyde (4.0 equiv., 20 mmol, 600 mg), thiophenol (1.0 equiv., 5.0 mmol, 0.51 mL), ethane-1,2-diamine (0.1 equiv., 0.5 mmol, 170 µL), and water (12.5 mL) was added to the sealed reaction vessel. The mixture was stirred at 140 °C for 4 h. Volatiles was removed under vacuum and the residue was purified by column chromatography (P.E/EtOAc, 19:1 v/v).[8] **1t** was obtained as a white solid (550 mg, 43.4% yield).o **^{1}H NMR** (500 MHz, $CDCl_3$) δ = 7.68 (dt, *J* = 7.9, 1.0 Hz, 1H), 7.39 – 7.33 (m, 2H), 7.31 – 7.26 (m, 2H), 7.26 – 7.21 (m, 2H), 7.20 – 7.11 (m, 2H), 6.96 (s, 1H), 4.36 (d, *J* = 0.8 Hz, 2H), 3.73 (s, 3H). **^{13}C NMR** (126 MHz, $CDCl_3$) δ = 137.6, 137.3, 129.4, 128.9, 128.0, 127.4, 126.0, 122.0, 119.3, 119.3, 110.2, 109.5, 32.9, 29.9.
The spectroscopic data matched those reported in the literature.[3]

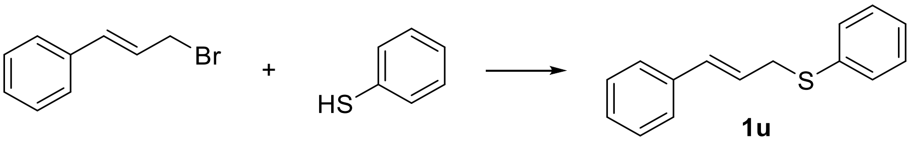

1u

NaH (1.4 equiv., 7.1 mmol, 290 mg) was added to THF (10 mL) and held at 0 °C . Thiophenol (1.1 equiv., 5.5 mmol, 560 µL) was added dropwise and stirred for 1 h. Cinnamyl bromide (1 equiv., 5.0 mmol, 740 µL) was added and the mixture was stirred at room temperature for 16 h. The mixture was quenched with sat. NH_4Cl solution (20 mL) and extracted with ethyl acetate, the organic layer was extracted with 2.5 M NaOH (25 mL x 2) and dried over sodium sulfate and purified by column chromatography (P.E) **1u** was obtained as a yellow solid (1.1 g, 97% yield). **^{1}H NMR** (400 MHz, $CDCl_3$) δ = 7.40 (dt, J = 8.1, 1.2 Hz, 2H), 7.35 – 7.26 (m, 6H), 7.26 – 7.18 (m, 2H), 6.44 (dd, J = 15.7, 1.3 Hz, 1H), 6.27 (dtd, J = 15.5, 7.1, 1.0 Hz, 1H), 3.73 (dd, J = 7.1, 1.1 Hz, 2H). **^{13}C NMR** (101 MHz, $CDCl_3$) δ = 136.8, 135.8, 132.8, 130.3, 128.9, 128.6, 127.6, 126.4, 126.4, 125.1, 37.2.
The spectroscopic data matched those reported in the literature.[3]

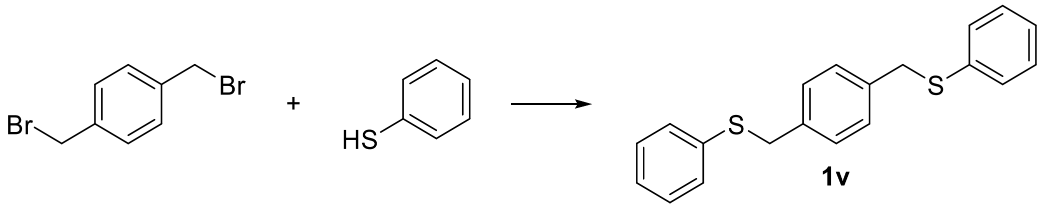

1v

Synthesis according to **General Procedure 3** with 2 equiv. of thiophenol. The crude product was purified by recrystallization from hexane/DCM. **1v** was obtained as a white solid (1.1 g, 68% yield). **^{1}H NMR** (400 MHz, $CDCl_3$) δ = 7.32 (dddd, J = 13.4, 8.0, 6.8, 1.3 Hz, 8H), 7.27 – 7.22 (m, 6H), 4.14 (s, 4H). **^{13}C NMR** (126 MHz, $CDCl_3$) δ = 136.6, 136.4, 130.1, 129.1, 129.0, 126.5, 38.9.
The spectroscopic data matched those reported in the literature.[9]

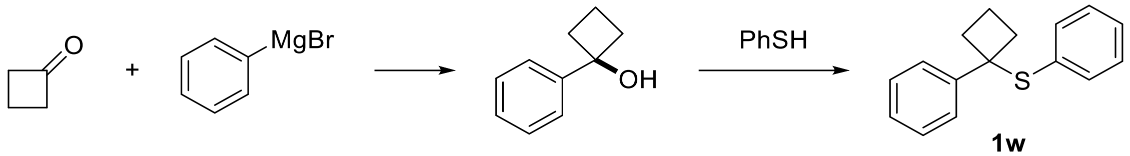

1w

Cyclobutanone (1 equiv., 5 mmol, 0.37 mL) was dissolved in DCM (10 mL) and held at 0 °C while 2.5 mL phenylmagnesium bromide (3.0 M in Et_2O) was added dropwise. The mixture was stirred at r. t. overnight before quenching with water and extracting with DCM and concentrated in *vacuo*. The crude was dissolved in DCM (10 mL) and held at 0 °C, thiophenol (2.5 equiv., 12.5 mmol, 1.3 mL) was added followed by dropwise addition of Boron trifluoride diethyl etherate (3.3 equiv., 16.5 mmol,1.3 mL). The reaction was stirred at 0 °C for 1 h, followed by overnight at r. t.. The reaction mixture was quenched with water and extracted with DCM, the organic phase was washed with 2.5 M NaOH (3x 25 mL) and dried over sodium sulfate, concentrated under reduced pressure, and purified by column chromatography (P.E). **1w** was obtained as a white solid (660 mg, 41% yield). **^{1}H NMR** (400 MHz, $CDCl_3$) δ = 7.29 – 7.13 (m, 6H), 7.10 (dt, J=7.0, 1.4, 2H), 7.02 – 6.95 (m, 2H), 2.67 – 2.57 (m, 2H), 2.54 – 2.44 (m, 2H), 2.44 – 2.32 (m, 1H), 1.94 – 1.84 (m, 1H). **^{13}C NMR** (101 MHz, $CDCl_3$) δ = 147.4, 136.1, 133.3, 128.5, 128.4, 127.8, 126.3, 126.2, 56.6, 34.6, 16.6.
The spectroscopic data matched those reported in the literature.[10]

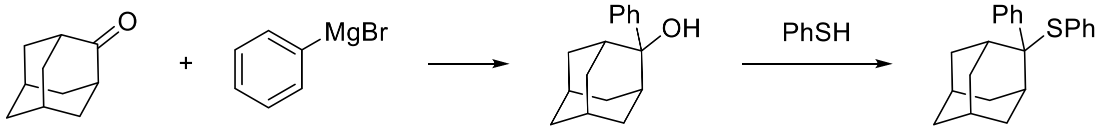

1x

2-Adamantanone (1 equiv., 5 mmol, 750 mg) was dissolved in DCM (10 mL) and held at 0 °C while 2.5 mL phenylmagnesium bromide (3.0 M in Et_2O) was added dropwise. The mixture was stirred at r. t. overnight before quenching with water and extracting with DCM and concentrated in *vacuo*. The crude was dissolved in DCM (10 mL) and held at 0 °C, thiophenol (2.5 equiv., 12.5 mmol, 1.3 mL) was added followed by dropwise addition of boron trifluoride diethyl etherate (3.3 equiv., 16.5 mmol,1.3 mL). The reaction was stirred at 0 °C for 1 h, followed by overnight at r. t.. The reaction mixture was quenched with water and extracted with DCM, the organic phase was washed with 2.5 M NaOH (3x 25 mL) dried over sodium sulfate, concentrated under reduced pressure, and purified by column chromatography (P.E). **1x** was obtained as a white solid (660 mg, 41% yield). **^{1}H NMR** (400 MHz, $CDCl_3$) δ = 7.24 – 7.14 (m, 1H), 7.14 – 7.00 (m, 5H), 6.99 – 6.93 (m, 2H), 6.82 – 6.74 (m, 2H), 2.93 (dd, J=13.0, 3.2, 2H), 2.63 (t, J=3.1, 2H), 2.07 (p, J=3.2, 1H), 1.90 – 1.79 (m, 4H), 1.78 – 1.71 (m, 3H), 1.67 (dddd, J=11.4, 3.8, 2.6, 1.4, 2H). **^{13}C NMR** (101 MHz, $CDCl_3$) δ = 143.7, 137.5, 132.3, 128.4, 128.0, 127.6, 126.6, 125.6, 63.3, 38.7, 34.4, 33.9, 33.6, 27.7, 27.6. **HRMS (APCI) m/z:**$[M-H]^+$ calculated for $C_{22}H_{23}S$ 319.1520; found 319.1526.

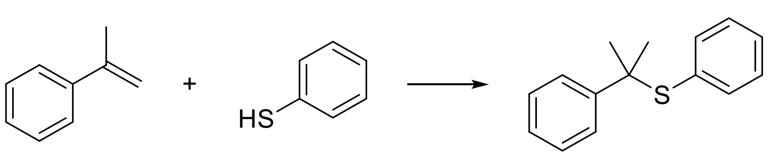

1y

Isopropenylbenzene (1.0 equiv., 5.0 mmol, 590 mg) and thiophenol (2.0 equiv., 10 mmol, 1.0 mL) was dissolved in DCM (30 mL) and cooled down to 0 °C. Trifluoroacetic acid (3 mL) was added dropwise, and the solution was stirred at room temperature for 4 hours. 15 mL saturated aqueous solution of NH_4Cl was added slowly to the reaction mixture. The organic layer was separated and washed twice with 5 M aqueous NaOH solution and three times with brine. The organic phase was concentrated *in vacuo* and purified by column chromatography (P.E) **1y** was obtained as a clear liquid (1.0 g, 88% yield). **^{1}H NMR** (400 MHz, $CDCl_3$) δ = 7.41 (dd, J = 7.7, 1.8 Hz, 2H), 7.34 – 7.24 (m, 3H), 7.24 – 7.11 (m, 5H), 1.69 (s, 6H). **^{13}C**

NMR (101 MHz, $CDCl_3$) δ = 146.5, 136.7, 132.9, 129.2, 128.7, 128.4, 128.0, 127.6, 127.3, 126.7, 126.7, 51.1, 29.8.
The spectroscopic data matched those reported in the literature.[3]

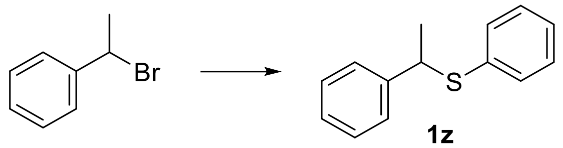

1z

Synthesis according to **General Procedure 3** and purified by column chromatography (P.E). **1z** was obtained as a clear liquid (740 mg, 69% yield). **^{1}H NMR** (500 MHz, $CDCl_3$) δ = 7.22 – 7.14 (m, 6H), 7.14 – 7.08 (m, 4H), 4.27 – 4.22 (m, 1H), 1.53 (dd, J=7.1, 1.3, 3H). **^{13}C NMR** (126 MHz, $CDCl_3$) δ = 143.3, 135.2, 132.6, 128.8, 128.5, 127.4, 127.2, 127.2, 48.1, 22.4.
The spectroscopic data matched those reported in the literature.[3]

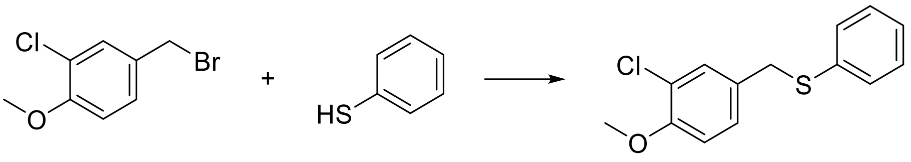

1aa

Synthesis according to **General Procedure 3** and purified by column chromatography (P.E:EtOAc 9:1). **1aa** was obtained as a clear liquid (1.03 g, 78% yield). **^{1}H NMR** (400 MHz, $CDCl_3$) δ = 7.33 – 7.23 (m, 5H), 7.23 – 7.17 (m, 1H), 7.12 (dd, *J*=8.4, 2.2, 1H), 6.82 (d, *J*=8.4, 1H), 4.03 (s, 2H), 3.88 (s, 3H). **^{13}C NMR** (101 MHz, $CDCl_3$) δ = 154.2, 136.0, 130.7, 130.7, 130.2, 129.0, 128.2, 126.7, 122.4, 112.0, 56.3, 38.3.
HRMS (ESI) m/z: $[M+Na+K]^{2+}$ calculated for $C_{14}H_{13}SOClNaK$ 162.9950; found 162.9931.

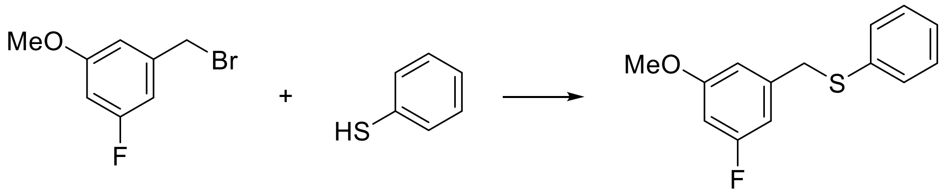

1ab

Synthesis according to **General Procedure 3** and purified by column chromatography (P.E:EtOAc 9:1). **1ab** was obtained as a clear liquid (630 mg, 85% yield). **^{1}H NMR** δ = 7.31 (dd, *J*=14.4, 7.3, 4H), 7.24 – 7.19 (m, 1H), 6.64 (dd, *J*=8.4, 2.1, 2H), 6.51 (dt, *J*=10.6, 2.3, 1H), 4.05 (s, 2H), 3.75 (s, 3H). **^{13}C NMR** (101 MHz, $CDCl_3$) δ = 163.6 (d, *J*=245.0), 160.9 (d, *J*=11.5), 140.7 (d, *J*=9.5), 135.8, 130.2, 129.0, 126.7, 110.2 (d, *J*=2.7), 108.0 (d, *J*=22.3), 100.6 (d, *J*=25.1), 55.6, 39.0 (d, *J*=2.3). **^{19}F NMR** δ = -111.7 (t, *J*=9.7). **HRMS (APCI) m/z:**$[M-H]^+$ calculated for $C_{14}H_{12}FOS$ 247.0593; found 247.0583.

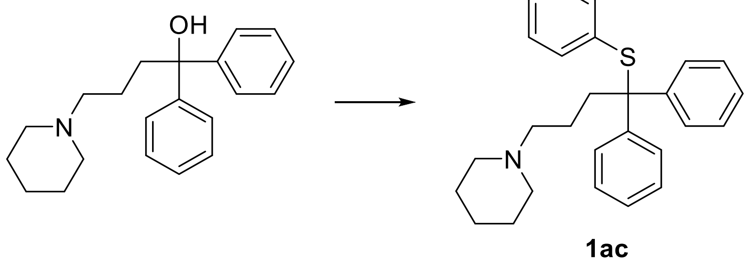

1ac

Difenidol (1 equiv., 5 mmol, 1.5 g), thiophenol (4.8 equiv., 24 mmol, 2.4 mL) was dissolved in DCM (10 mL) and held at 0 °C while boron trifluoride diethyl etherate (5.6 equiv., 28 mmol, 3.5 mL) was added dropwise. The mixture was stirred at r. t. overnight followed by quenching with water and extracted with DCM, the organic phase was washed with 2.5 M NaOH (3x 25 mL) dried over sodium sulfate, concentrated under reduced pressure. **1ac** was obtained as clear liquid (1.8 g, 90% yield). **^{1}H NMR** 1H NMR (400 MHz, $CDCl_3$) δ = 7.30 – 7.25 (m, 4H), 7.25 – 7.15 (m, 7H), 7.08 (t, J=7.7, 2H), 6.93 – 6.89 (m, 2H), 2.28 (s, 4H), 2.22 – 2.18 (m, 2H), 2.16 – 2.11 (m, 2H), 1.52 (ddt, J=11.4, 8.1, 4.9, 6H), 1.40 (q, J=5.9, 2H). **^{13}C NMR** (101 MHz, $CDCl_3$) δ = 145.5, 137.3, 131.6, 128.8, 128.8, 128.1, 127.6, 126.4, 63.9, 59.6, 54.7, 37.2, 26.1, 24.6, 22.5. **HRMS (ESI) m/z:** $[M+H]^+$ calculated for C27H$_{31}$NS 402.2255; found 402.2263.

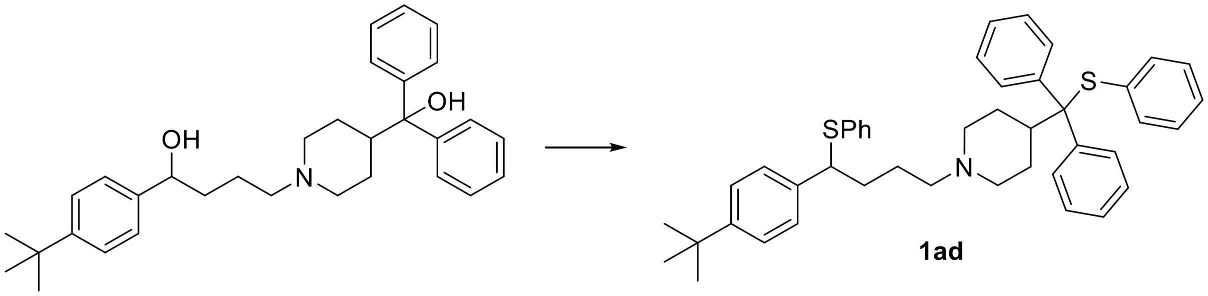

1ad

Terfenadine (1 equiv., 5 mmol, 2,4 g), thiophenol (4.8 equiv., 24 mmol, 2.4 mL) was dissolved in DCM (10 mL) and held at 0 °C while boron trifluoride diethyl etherate (5.6 equiv., 28 mmol, 3.5 mL) was added dropwise. The mixture was stirred at r. t. overnight followed by quenching with water and extracted with DCM, the organic phase was washed with 2.5 M NaOH (3x 25 mL) dried over sodium sulfate, concentrated under reduced pressure. **1ad** was obtained as a white solid (2.4 g, 73% yield). **^{1}H NMR** (400 MHz, $CDCl_3$) δ = 7.31 – 7.13 (m, 20H), 7.06 (t, J=7.7, 2H), 6.82 – 6.77 (m, 2H), 4.12 (dd, J=8.8, 6.0, 1H), 2.95 – 2.87 (m, 2H), 2.31 – 2.17 (m, 5H), 1.91 (ttd, J=13.7, 6.8, 3.3, 4H), 1.45 (tdd, J=13.1, 7.8, 5.4, 2H), 1.31 (s, 9H), 1.09 (qd, J=15.3, 9.1, 2H). **^{13}C NMR** (101 MHz, $CDCl_3$) δ = 150.1, 141.9, 138.6, 137.5, 135.7, 132.0, 131.8, 130.4, 128.9, 128.7, 128.1, 127.4, 127.1, 126.8, 126.6, 125.4, 69.1, 58.4, 54.7, 54.3, 52.8, 42.4, 34.6, 34.2, 31.5, 28.7 (d, J=3.2), 25.1. **HRMS (ESI) m/z:** $[M+H]^+$ calculated for $C_{44}H_{50}NS_2$ 656.3385; found 656.3378.

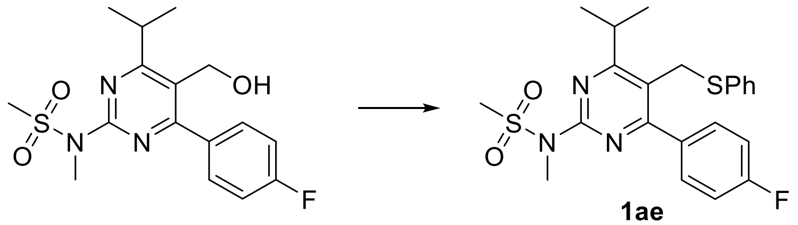

1ae

The corresponding alcohol (1.0 equiv., 5.0 mmol, 1.8 g), Ph_3P (1.4 equiv., 7.0 mmol, 1.8 g)., and 1,2-diiodoethane (1.4 equiv., 7.0 mmol, 2.0 g) were dissolved in DMF (30 mL). Next, tetrabutylammonium iodine (1.5 equiv., 7.5 mmol, 2.8 g) was added followed by thiophenol (3 equiv., 15 mmol, 1.5 mL), the reaction mixture was stirred at 80 °C for 1 hour. The resulting mixture was extracted with ethyl acetate and washed with water (x5) and brine (x2). The organic layer was dried with sodium sulfate. Purified by column chromatography (P.E/EtOAc 9:1). **1ae** was obtained as a white solid (1.1 g, 58%). **^{1}H NMR** (400 MHz, $CDCl_3$) δ = 7.85 – 7.75 (m, 2H), 7.30 (d, J=3.9, 4H), 7.27 – 7.22 (m, 1H), 7.18 – 7.08 (m, 2H), 4.10 (s, 2H), 3.54 (m, 7H), 1.35 (d, J=6.6, 6H). **^{13}C NMR** (126 MHz, $CDCl_3$) δ = 177.3, 166.0, 164.6, 162.6, 157.8, 135.8, 134.2, 134.2, 131.1, 131.1, 129.8, 129.3, 127.0, 118.0, 115.6, 115.4, 42.6, 33.2, 32.9, 31.8, 22.4. **^{19}F NMR** (377 MHz, $CDCl_3$) δ = -111.2. **HRMS (ESI) m/z:** $[M+Na]^+$ calculated for $C_{22}H_{24}FN_3O_2S_2Na$ 486.1192; found 486.1224.

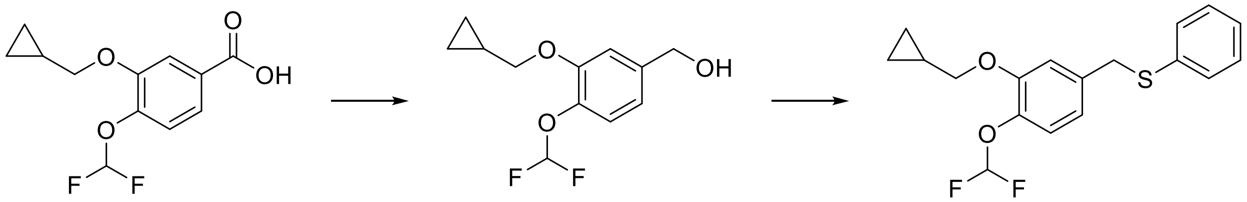

1af

To a stirred solution of the carboxylic acid (1 equiv., 6 mmol, 1.55 g) in anhydrous THF (30 mL) BH_3*THF complex (1 M solution in THF, 2 equiv., 12 mL) was added drop wise at RT under nitrogen atmosphere. After 3 hours the reaction was quenched with saturated aqueous NH_4Cl solution. The mixture was extracted with EtOAc and dried over sodium sulfate, concentrated under reduced pressure, and used directly in **General Procedure 5** and purified by column chromatography (P.E/EtOAc 19:1) **1af** was obtained as a white solid (800 mg, 40% yield). **^{1}H NMR** (400 MHz, $CDCl_3$) δ = 7.33 – 7.22 (m, 4H), 7.21 (d, J=7.0, 1H), 7.05 (d, J=8.0, 1H), 6.86 – 6.79 (m, 2H), 6.59 (t, J=75.7, 1H), 4.05 (s, 2H), 3.78 (d, J=7.0, 2H), 1.30 – 1.16 (m, 1H), 0.63 (p, J=6.4, 2H), 0.33 (t, J=5.1, 2H). **^{13}C NMR** (101 MHz, $CDCl_3$) δ = 150.5, 139.5, 136.4, 135.9, 130.4, 129.0, 126.8, 122.7, 121.4, 116.4 (t, J=259.3), 114.8, 73.9, 39.0, 10.2, 3.3. **^{19}F NMR** (377 MHz, $CDCl_3$) δ = -81.5. **HRMS (ESI) m/z:** $[M+H]^+$ calculated for $C_{18}H_{19}F_2O_2S$ 337.1074; found 337.1059.

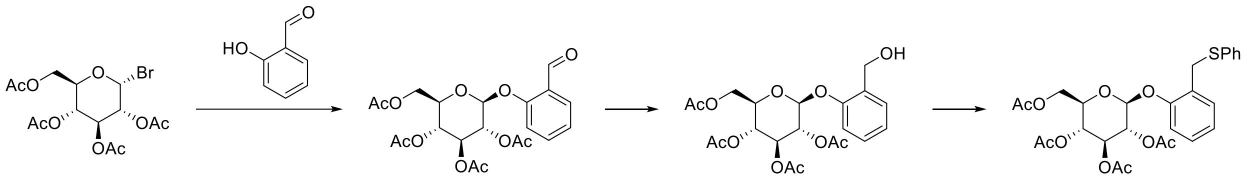

1ag

Salicylaldehyde (1 equiv., 6 mmol, 730 mg), peracetylglucosyl bromide (1.25 equiv., 7.5 mmol, 3 g), and tetrabutylammonium bromide (1.3 equiv., 8 mmol, 2.6 g) were dissolved in DCM (30 mL) and warmed to 35 °C, and a soliution of sodium hydroxide (5%, 10 mL) was added. The reaction mixture was refluxed overnight. The mixture was washed with 1 M aq. HCl and brine. Dried over sodium sulfate and concentrated. Purified by column chromatography (P.E/EtOAc 2:1) to obtain the first intermediate as a white solid (730 mg 1.7 mmol) which was dissolved in chloroform (12 mL) $NaBH_4$ (1 equiv., 1.7 mmol, 64 mg) was dissolved in 5 mL of water and added to the mixture. Stirred at r. t. over night. The organic phase was washed with 1 M aq. solution of HCl, dried over sodium sulfate, concentrated and used directly in **General Procedure 4**. Purified by column chromatography (P.E/EtOAc 7:3). **1ag** was obtained as a white solid (600 mg, 17% yield). **^{1}H NMR** (400 MHz, $CDCl_3$) δ = 7.30 (t, J=7.3, 3H), 7.20 (p, J=7.9, 4zH), 6.99 (dt, J=15.0, 7ZZ.8, 2H), 5.40 – 5.27 (m,Z 2H), 5.18 (dd, J=11.2, 7.6, 1H), 5.11 (d, J=7.3, 1H), 4.30 (dd, J=12.3, 5.4, 1H), 4.23 – 4.16 (m, 2H), 3.95 (d, J=12.8, 1H), 3.88 (ddd, J=10.6, 5.7, 2.4, 1H), 2.07 (s, 3H), 2.05 (s, 3H), 2.03 (s, 3H), 2.00 (s, 3H). **^{13}C NMR** (101 MHz, $CDCl_3$) δ = 170.7, 170.4, 169.6, 169.5, 154.5, 136.6, 130.8, 130.1, 129.0, 128.6, 127.2, 126.5, 123.3, 115.3, 99.1, 72.8, 72.1, 71.0, 68.5, 62.1, 33.0, 20.9, 20.8, 20.8. **HRMS (ESI) m/z:** $[M+Na]^+$ calculated for $C_{27}H_{30}O_{10}SNa$ 569.1457; found 569.1461.

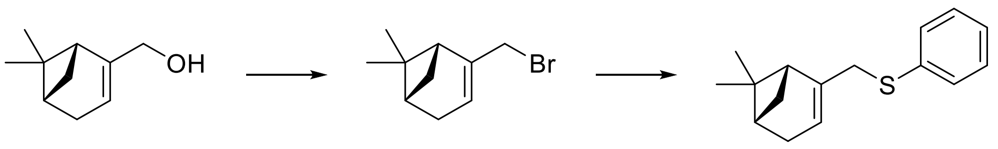

1ah

To a 0 °C solution of (1R)-Myrtenol (1 equiv., 10 mmol, 1.5 g) in benzene (30 mL) CBr_4 (2 equiv., 6.6 g, 20 mmol) and Ph_3P (2 equiv., 5.2 g, 20 mmol) was added under N_2.The solution was stirred at the same temperature for 3 h, and petroleum ether (80 mL)was then added. The reaction mixture was filtered. The filtrate was concentrated under reduced pressure. The reaction crude was used in **General Procedure 3** and purified by column chromatography (P.E). **1ah** was obtained as a clear liquid (860 mg, 35% yield). **^{1}H NMR** (400 MHz, $CDCl_3$) δ = 7.35 – 7.28 (m, 2H), 7.25 (dd, J=8.6, 6.7, 2H), 7.19 – 7.11 (m, 1H), 5.43 (d, J=2.0,

1H), 3.58 – 3.48 (m, 2H), 2.38 (dt, J=8.6, 5.6, 1H), 2.25 (td, J=5.7, 1.5, 1H), 2.23 – 2.18 (m, 2H), 2.06 (dp, J=5.9, 2.7, 1H), 1.28 (s, 3H), 1.08 (d, J=8.6, 1H), 0.77 (s, 3 H). **^{13}C NMR** (101 MHz, $CDCl_3$) δ = 143.0, 137.1, 129.3, 128.8, 125.8, 120.7, 45.3, 40.6, 40.5, 38.3, 31.8, 31.4, 26.3, 21.3.
The spectroscopic data matched those reported in the literature.[11]

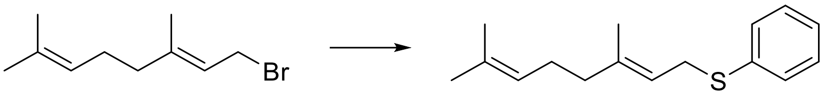

1ai

Synthesis according to **General Procedure 3** and purified by column chromatography (P.E). **1ai** was obtained as a clear liquid (1.1 g, 90% yield). **^{1}H NMR** (400 MHz, $CDCl_3$) δ = 7.37 – 7.32 (m, 2H), 7.30 – 7.24 (m, 3H), 7.21 – 7.15 (m, 1H), 5.31 (tq, J=7.6, 1.3, 1H), 5.06 (tdd, J=6.7, 3.0, 1.4, 1H), 3.55 (d, J=7.7, 2H), 2.03 (qq, J=9.3, 4.5, 4H), 1.67 (d, J=1.4, 3H), 1.59 (dd, J=5.9, 1.4, 6H). **^{13}C NMR** (101 MHz, $CDCl_3$) δ = 140.1, 136.9, 131.8, 130.0, 128.8, 126.1, 124.0, 119.3, 39.7, 32.3, 26.6, 25.8, 17.8, 16.2.
The spectroscopic data matched those reported in the literature.[12]

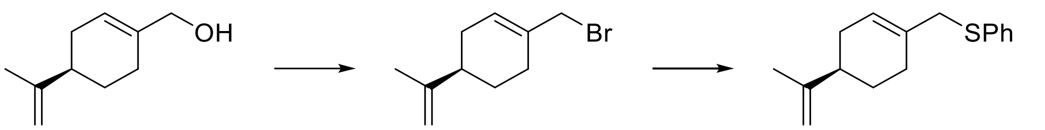

1aj

To a 0 °C solution of (S)-perillyl alcohol (1 equiv., 10 mmol, 1.5 g) in benzene (30 mL) CBr_4 (2 equiv., 6.6 g, 20 mmol) and Ph_3P (2 equiv., 5.2 g, 20 mmol) was added under N_2.The solution was stirred at the same temperature for 3 h, and petroleum ether (80 mL)was then added. The reaction mixture was filtered. The filtrate was concentrated under reduced pressure. The reaction crude was used in **General Procedure 3** and purified by column chromatography (P.E). **1aj** was obtained as a clear liquid (370 mg, 30% yield). **^{1}H NMR** (400 MHz, $CDCl_3$) δ = 7.35 – 7.31 (m, 2H), 7.27 (td, J=7.5, 1.2, 2H), 7.20 – 7.15 (m, 1H), 5.55 (ddq, J=4.9, 2.6, 1.3, 1H), 4.74 – 4.66 (m, 2H), 3.53 – 3.44 (m, 2H), 2.26 – 2.14 (m, 2H), 2.13 – 2.03 (m, 2H), 1.96 – 1.78 (m, 2H), 1.72 (t, J=1.1, 3H), 1.46 (dddd, J=12.8, 11.4, 9.9, 6.7, 1H). **^{13}C NMR** (101 MHz, $CDCl_3$) δ = 149.8, 136.9, 133.0, 130.2, 128.8, 126.2, 125.2, 108.8, 42.0, 40.9, 30.8, 27.8, 27.7, 21.0. **HRMS (APCI) m/z:** $[M+H]^+$ calculated for $C_{16}H_{21}S$ 245.1364; found 245.1359.

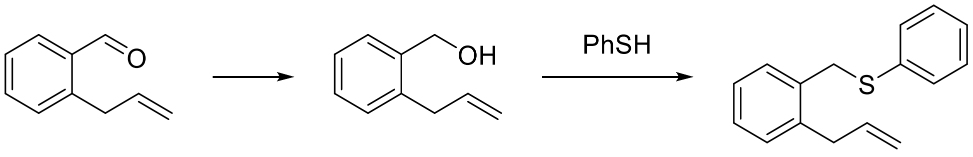

1ak

2-allylbenzaldehyde (1 equiv., 5 mmol, 730 mg) was dissolved in abs. ethanol (10 mL) and $NaBH_4$ (2.5 equiv., 12.5 mmol, 470 mg) at 0 °C. The reaction was stirred at room temperature for 2 h, then cooled to 0 °C and quenched with water (20 mL) and extracted with DCM (3x 40 mL). The combined organic layer was dried over sodium sulfate, concentrated under reduced pressure. The crude was used in **General Procedure 4** and purified by column chromatography (P.E). **1ak** was obtained as a clear liquid (550 mg, 62% yield). **^{1}H NMR** (400 MHz, $CDCl_3$) δ = 7.37 – 7.10 (m, 10H), 6.00 (ddt, J = 16.5, 10.1, 6.3 Hz, 1H), 5.13 – 4.96 (m, 2H), 4.14 (s, 2H), 3.52 (dt, J = 6.4, 1.7 Hz, 2H). **^{13}C NMR** (101 MHz, $CDCl_3$) δ = 138.5, 137.0, 136.7, 135.1, 130.4, 130.1, 130.1, 129.0, 127.9, 126.7, 126.5, 116.1, 37.1, 36.8. **HRMS (ESI) m/z:** $[M+H]^+$ calculated for $C_{16}H_{17}S$ 241.1051; found 241.1040.

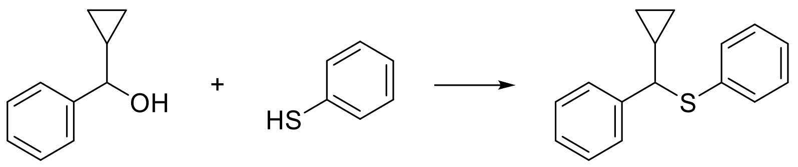

1al

In a round-bottom flask equipped with a stirring bar, α-Cyclopropylbenzyl alcohol (1.0 equiv, 3.5 mmol, 0.50 mL) and and *p*-toluenesulfonic acid monohydrate (0.05 equiv., 0.18 mmol, 30 mg) were dissolved in acetonitrile (20 mL) and degassed with nitrogen for 15 minutes. Thiophenol (1.05 equiv., 0.38 mL, 3.7 mmol) was added and the mixture was heated to 50 °C and reacted until completion, tracked with TLC. Water (10 mL) was added and organic was removed under reduced pressure, followed by extraction with ethyl acetate (3 x 10 mL). The combined organic layers were washed with brine (15 mL) and dried over sodium sulphate and purified by silica gel flash chromatography (DCM) **1al** was obtained as a colorless oil. **^{1}H NMR** (400 MHz, $CDCl_3$) δ = 7.35 (ddt, *J*=12.6, 9.9, 4.8, 6H), 7.29 – 7.20 (m, 4H), 3.60 (d, *J*=9.5, 1H), 1.46 – 1.35 (m, 1H), 0.80 – 0.71 (m, 1H), 0.64 – 0.55 (m, 1H), 0.42 (dq, *J*=10.2, 5.1, 1H), 0.33 (dq, *J*=9.8, 5.0, 1H). **^{13}C NMR** (101 MHz, $CDCl_3$) δ = 142.2, 134.8, 133.0, 128.5, 128.2, 127.8, 127.1, 127.0, 58.9, 17.1, 6.5, 5.1. **HRMS (APCI) m/z:**[M-H]$^+$ calculated for $C_{16}H_{15}S$ 239.0894; found 239.0875.

S5.2 Synthesis and characterization of thioacetals

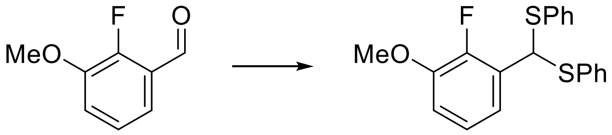

3b

Synthesis according to **General Procedure 6** with 5 mmol of the corresponding aldehyde and purified by column chromatography (19:1 P.E/EtOAc). **3b** was obtained as a yellow oil (1.1 g, 60% yield). **^{1}H NMR** (400 MHz, $CDCl_3$) δ = 7.42 – 7.38 (m, 4H), 7.30 – 7.22 (m, 6H), 7.19 (ddd, J=7.8, 6.1, 1.5, 1H), 7.02 (td, J=8.1, 1.5, 1H), 6.84 (td, J=8.2, 1.5, 1H), 5.88 (s, 1H), 3.84 (s, 3H). **^{13}C NMR**(101 MHz, $CDCl_3$) δ = 149.1 (d, J=246.9), 147.4 (d, J=11.0), 134.3, 132.6, 129.0 (d, J=1.5), 128.1 – 128.0 (m), 124.1 (dd, J=4.8, 1.6), 120.6 (d, J=1.8), 112.8 (d, J=2.1). **^{19}F NMR** (377 MHz, $CDCl_3$) δ = -140.4 (d, J=5.5). **HRMS (ESI) m/z:** [M+Na]$^+$ calculated for $C_{20}H_{17}FOS_2Na$ 379.0603; found 379.0596.

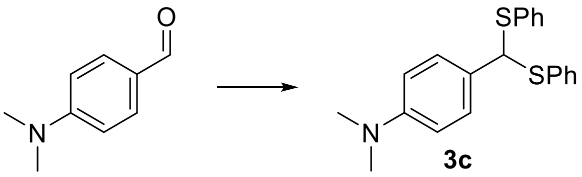

3c

Synthesis according to **General Procedure 6** with 5 mmol of the corresponding aldehyde. The crude was dissolved in minimal amount of hot DCM and put in freezer for 72 hours. The white precipitate was filtered off and washed with pentane. **3c** was obtained as a white solid (1.1 g, 63% yield). **^{1}H NMR** (400 MHz, $CDCl_3$) δ = 7.36 – 7.27 (m, 4H), 7.25 – 7.15 (m, 8H), 6.63 – 6.54 (m, 2H), 5.38 (s, 1H), 2.89 (s, 6H). **^{13}C NMR** (101 MHz, $CDCl_3$) δ = 150.3, 135.4, 132.1, 128.9, 128.8, 127.5, 127.0, 112.3, 59.9, 40.6. **HRMS (ESI) m/z:** [M+H]$^+$ calculated for $C_{21}H_{22}NS_2$ 352.1194; found 352.1189.

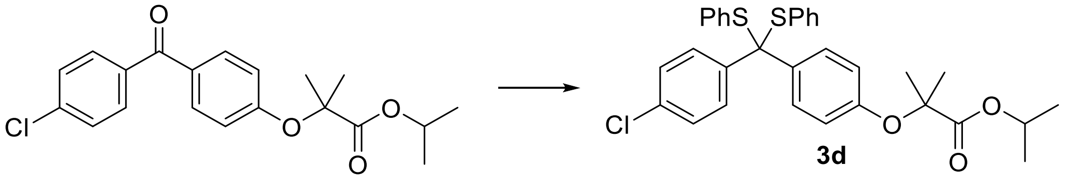

3d

Synthesis according to **General Procedure 6** with 5 mmol of the corresponding ketone and purified by column chromatography (19:1 P.E/EtOAc). **3d** was obtained as an off-white solid (2.3 g, 82% yield). **^{1}H NMR** (400 MHz, $CDCl_3$) δ = 7.30 – 7.20 (m, 4H), 7.19 – 7.14 (m, 2H), 7.13 – 7.06 (m, 10H), 6.64 – 6.56 (m, 2H), 5.08 (hept, J=6.3, 1H), 1.56 (s, 6H), 1.23 (d, J=6.3, 6H). **^{13}C NMR** (101 MHz, $CDCl_3$) δ = 173.7, 154.6, 141.4, 137.0, 135.6, 132.9, 131.7, 130.9, 130.3, 129.2, 128.3, 127.4, 117.9, 79.2, 74.4, 69.1, 25.4, 21.8. **HRMS (ESI) m/z:** $[M+Na]^+$ calculated for $C_{32}H_{31}ClO_3S_2Na$ 585.1295; found 585.1295.

S5.3 Synthesis and characterization of products

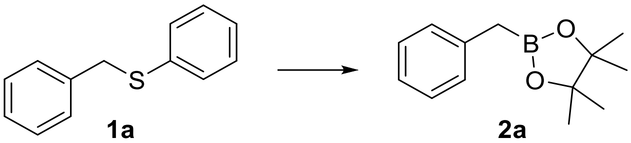

1a **2a**

Synthesis according to **General Procedure 1** and purified by column chromatography (P.E/EtOAc 49:1) obtained **2a** as a clear liquid (104 mg, 95% yield). **^{1}H NMR** (400 MHz, $CDCl_3$) δ = 7.26 – 7.16 (m, 4H), 7.13 – 7.09 (m, 1H), 2.29 (s, 2H), 1.22 (s, 13H). **^{13}C NMR** (101 MHz, $CDCl_3$) δ = 138.7, 129.1, 128.4, 124.9, 83.5, 24.8. **^{11}B NMR** (128 MHz, $CDCl_3$) δ = 33.2.
The spectroscopic data matched those reported in the literature.[13]

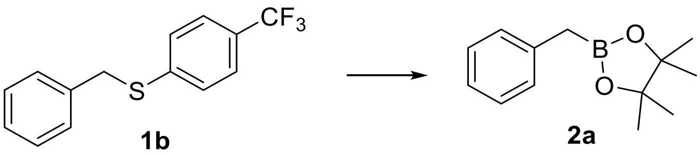

1b **2a**

Synthesis according to **General Procedure 1** and analyzed by HPLC. HPLC yield 78%.

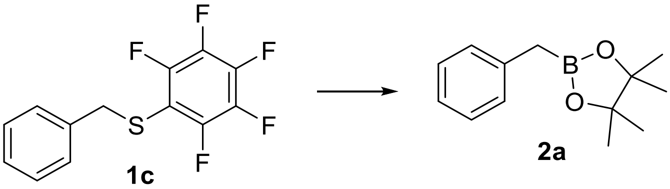

1c **2a**

Synthesis according to **General Procedure 1** and analyzed by HPLC. HPLC yield 57%.

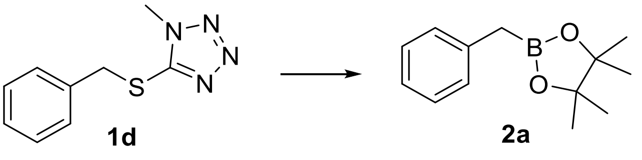

1d **2a**

Synthesis according to **General Procedure 1** and analyzed by HPLC. HPLC yield 21%.

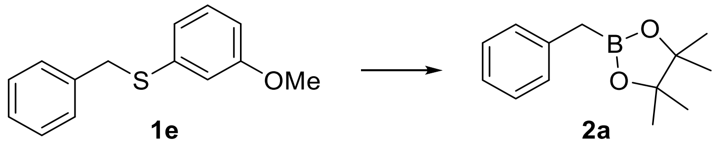

Synthesis according to **General Procedure 1** and analyzed by HPLC. HPLC yield 36%.

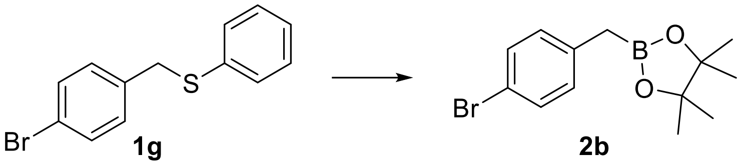

Synthesis according to **General Procedure 1** and purified by column chromatography (P.E/EtOAc 49:1) obtained **2b** as a clear liquid (78 mg, 53% yield). **^{1}H NMR** (400 MHz, $CDCl_3$) δ = 7.30 (d, J = 8.3 Hz, 1H), 7.01 (d, J = 8.4 Hz, 1H), 2.19 (s, 1H), 1.18 (s, 7H). **^{13}C NMR** (101 MHz, $CDCl_3$) δ = 137.8, 131.3, 130.8, 118.6, 83.6. **^{11}B NMR** (128 MHz, $CDCl_3$) δ = 32.9.
The spectroscopic data matched those reported in the literature.[14]

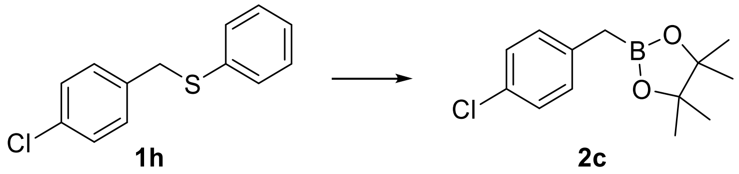

Synthesis according to **General Procedure 1** and purified by column chromatography (P.E/EtOAc 49:1) obtained **2c** as a clear liquid (92 mg, 73% yield). **^{1}H NMR** (400 MHz, $CDCl_3$) δ = 7.18 (d, J = 8.4 Hz, 2H), 7.10 (d, J = 8.5 Hz, 2H), 2.25 (s, 2H), 1.22 (s, 12H). **^{13}C NMR** (101 MHz, $CDCl_3$) δ = 137.3, 130.7, 130.4, 128.4, 83.7, 24.8. **^{11}B NMR** (128 MHz, $CDCl_3$) δ = 33.0.
The spectroscopic data matched those reported in the literature.[15]

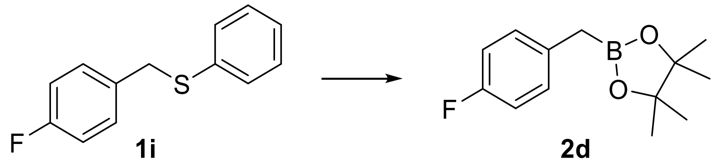

Synthesis according to **General Procedure 1** and purified by column chromatography (P.E/EtOAc 49:1) obtained **2d** as a clear liquid (78 mg, 66% yield). **^{1}H NMR** (400 MHz, $CDCl_3$) δ = 7.12 (dd, J = 8.5, 5.5 Hz, 2H), 6.92 (t, J = 8.8 Hz, 2H), 2.25 (s, 2H), 1.23 (s, 12H). **^{13}C NMR** (101 MHz, $CDCl_3$) δ = 160.9 (d, J = 241.9 Hz), 134.2 (d, J = 3.2 Hz), 130.3 (d, J = 7.7 Hz), 115.1 (d, J = 21.0 Hz), 83.6, 24.8. **^{11}B NMR** (128 MHz, $CDCl_3$) δ = 33.2. **^{19}F NMR** (377 MHz, $CDCl_3$) δ = -119.3.
The spectroscopic data matched those reported in the literature.[16]

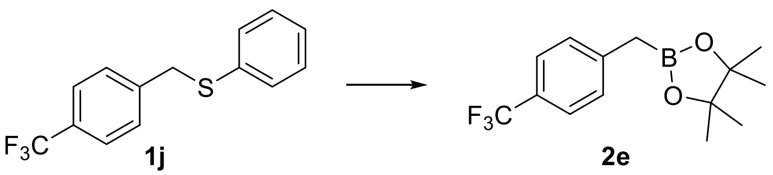

1j **2e**

Synthesis according to **General Procedure 1** and purified by column chromatography (P.E/EtOAc 49:1) obtained **2e** as a clear liquid (112.3 mg, 79% yield). **^{1}H NMR** (400 MHz, $CDCl_3$) δ = 7.47 (d, J = 8.0 Hz, 2H), 7.27 (d, J = 8.0 Hz, 2H), 2.34 (s, 2H), 1.22 (s, 12H). **^{13}C NMR** (101 MHz, $CDCl_3$) δ = 143.3 (d, J = 1.6 Hz), 129.3, 125.3 (q, J = 3.8 Hz), 124.7 (q, J = 271.5 Hz), 83.8, 24.8, 20.4. **^{11}B NMR** (128 MHz, $CDCl_3$) δ = 32.9. **^{19}F NMR** (377 MHz, $CDCl_3$) δ = -62.2.
The spectroscopic data matched those reported in the literature.[14]

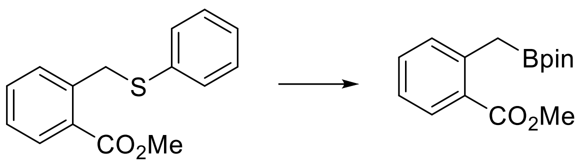

1k **2f**

Synthesis according to **General Procedure 1** and purified by column chromatography (P.E/EtOAc 19:1) obtained **2f** as a clear liquid (19 mg, 14 % yield). **^{1}H NMR** (400 MHz, $CDCl_3$) δ = 7.97 – 7.93 (m, 1H), 7.39 (tt, J=7.5, 1.4, 1H), 7.20 (t, J=7.2, 2H), 3.86 (s, 3H), 2.56 (s, 2H), 1.22 (s, 12H). **^{13}C NMR** (101 MHz, $CDCl_3$) δ = 168.2, 142.8, 132.5, 132.0, 130.8, 128.3, 125.3 (d, J=2.3), 83.3 (d, J=2.1), 51.9, 25.0 (d, J=2.5). **^{11}B NMR** (128 MHz, $CDCl_3$) δ = -32.8. **HRMS (ESI) m/z:** $[M+H]^+$ calculated for $C_{15}H_{21}BO_4$ 277.1611; found 277.1600.

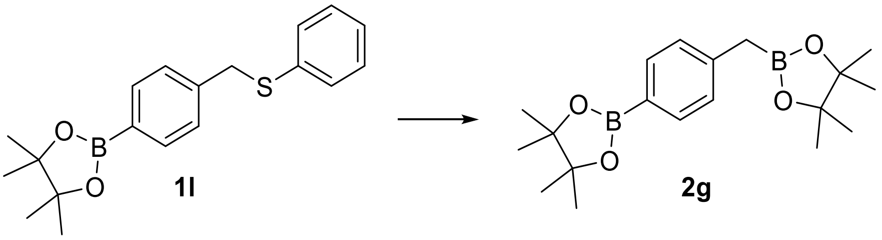

1l **2g**

Synthesis according to **General Procedure 1** and purified by column chromatography (P.E/EtOAc 19:1) obtained **2g** as a white solid (141 mg, 82 % yield). **^{1}H NMR** (400 MHz, $CDCl_3$) δ = 7.65 – 7.58 (m, 2H), 7.15 – 7.09 (m, 2H), 2.23 (s, 2H), 1.26 (s, 12H), 1.14 (s, 12H). **^{13}C NMR** (101 MHz, $CDCl_3$) δ = 142.5, 135.0, 128.6, 83.7, 25.0, 24.8. **^{11}B NMR** (128 MHz, $CDCl_3$) δ = 33.2.
The spectroscopic data matched those reported in the literature.[17]

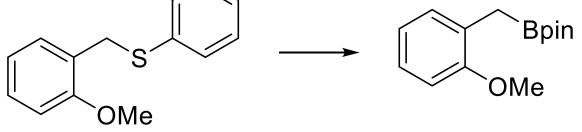

1m **2h**

Synthesis according to **General Procedure 1** and purified by column chromatography (P.E/EtOAc 19:1) obtained **2h** as a clear liquid (98 mg, 79 % yield). **^{1}H NMR** (500 MHz, $CDCl_3$) δ = 7.15 – 7.10 (m, 1H), 6.85 (td, *J*=7.4, 1.2, 0H), 6.82 – 6.79 (m, 1H), 3.80 (s, 1H), 2.18 (s, 1H), 1.24 (s, 6H). **^{13}C NMR** (126 MHz, $CDCl_3$) δ = 157.3, 130.6, 128.1, 126.4, 120.6, 109.9, 83.3, 55.2, 24.8. **^{11}B NMR** (160 MHz, $CDCl_3$) δ = 33.5. **HRMS (ESI) m/z:** $[M+H]^+$ calculated for $C_{14}H_{22}BO_3$ 249.1662; found 249.1655.

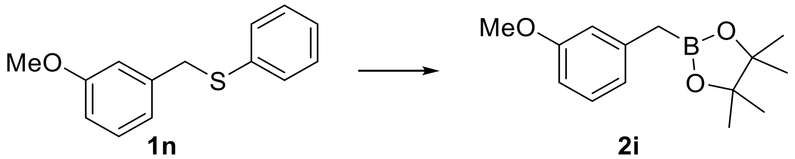

1n **2i**

Synthesis according to **General Procedure 1** and purified by column chromatography (P.E/EtOAc 19:1) obtained **2i** as a clear liquid (110 mg, 89% yield). **^{1}H NMR** (400 MHz, $CDCl_3$) δ = 7.15 (t, J = 7.9 Hz, 1H), 6.81 – 6.72 (m, 2H), 6.68 (dd, J = 8.3, 2.6 Hz, 1H), 3.78 (s, 3H), 2.27 (s, 2H), 1.24 (s, 12H). **^{13}C NMR** (101 MHz, $CDCl_3$) δ = 159.6, 140.3, 129.3, 121.6, 114.7, 110.5, 83.6, 55.2, 24.9. **^{11}B NMR** (128 MHz, $CDCl_3$) δ = 33.2. The spectroscopic data matched those reported in the literature.[18]

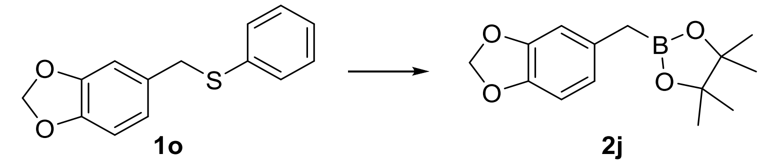

1o **2j**

Synthesis according to **General Procedure 1** but with 3 equivalent pinacolborane. Purified by column chromatography (P.E/EtOAc 49:1) obtained **2j** as a clear liquid (119 mg, 91% yield). **^{1}H NMR** (400 MHz, $CDCl_3$) 1H NMR (400 MHz, CDCl3) δ = 6.72 – 6.66 (m, 2H), 6.65 – 6.58 (m, 1H), 5.89 (s, 2H), 2.21 (s, 2H), 1.24 (s, 12H). **^{13}C NMR** (101 MHz, $CDCl_3$) δ = 147.5, 145.1, 132.3, 121.6, 109.7, 108.2, 100.7, 83.6, 24.9. **^{11}B NMR** (128 MHz, $CDCl_3$) δ = 33.2. **HRMS (ESI) m/z:** $[M+H]^+$ calculated for $C_{16}H_{26}BF_2O_4$ 263.1455; found 263.1452.

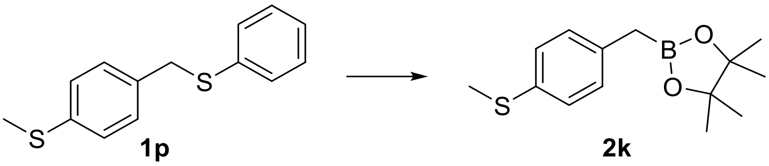

1p **2k**

Synthesis according to **General Procedure 1** and purified by column chromatography (P.E/EtOAc 49:1) obtained **2k** as a clear liquid (97.0 mg, 74% yield). **^{1}H NMR** (400 MHz, $CDCl_3$) δ = 7.16 (d, J=8.4, 1H), 7.11 (d, J=8.4, 2H), 2.45 (s, 3H), 2.25 (s, 2H), 1.23 (s, 11H). **^{13}C NMR** (101 MHz, $CDCl_3$) δ = 135.9, 134.0, 129.5, 127.4, 83.5, 24.8, 16.5. **^{11}B NMR** (128 MHz, $CDCl_3$) δ = 33.2.
The spectroscopic data matched those reported in the literature.[19]

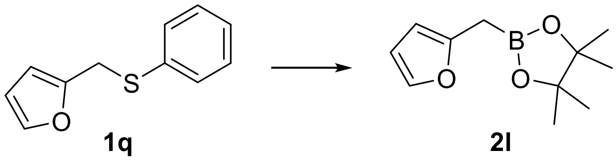

1q **2l**

Synthesis according to **General Procedure 1** and purified by column chromatography (P.E/EtOAc 49:1) obtained **2l** as a clear liquid (56 mg, 54% yield). **^{1}H NMR** (400 MHz, $CDCl_3$) δ = 7.21 – 7.18 (m, 1H), 6.18 (dt, J=3.3, 1.5, 1H), 5.96 (dt, J=3.1, 1.1, 1H), 2.23 (s, 2H), 1.20 (s, 12H). **^{13}C NMR** (101 MHz, $CDCl_3$) δ = 152.5, 140.7, 110.4, 105.4, 83.8, 24.9. **^{11}B NMR** (128 MHz, $CDCl_3$) δ = 32.7. **HRMS (ESI) m/z:** $[M+H]^+$ calculated for $C_{11}H_{18}BO_2$ 209.1349; found 209.1340.

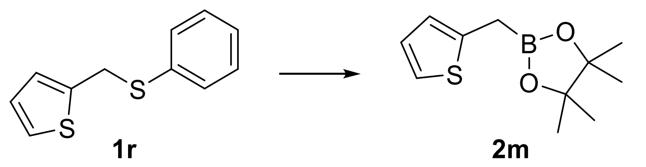

Synthesis according to **General Procedure 1** and purified by column chromatography (P.E/EtOAc 99:1) obtained **2m** as a clear liquid (76 mg, 68 % yield). **^{1}H NMR** (400 MHz, $CDCl_3$) δ = 7.05 (dd, J=5.2, 1.2, 1H), 6.89 (dd, J=5.2, 3.4, 1H), 6.82 – 6.80 (m, 1H), 2.49 (s, 2H), 1.27 (s, 12H). **^{13}C NMR** (101 MHz, $CDCl_3$) δ = 140.4, 127.0, 125.0, 122.8, 83.9, 24.9. **^{11}B NMR** (128 MHz, $CDCl_3$) δ = 32.8. **HRMS (ESI) m/z:** $[M+H]^+$ calculated for $C_{11}H_{18}BO_2S$ 225.1120; found 225.1117.

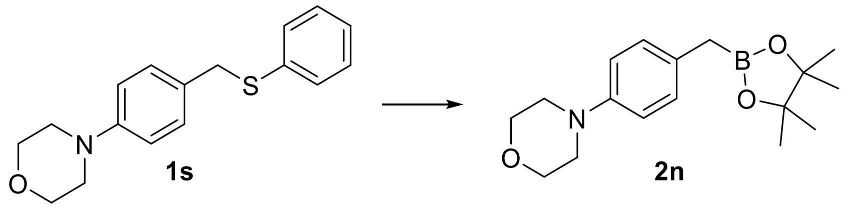

Synthesis according to **General Procedure 1** and purified by column chromatography (P.E/EtOAc 3:2) obtained **2n** as a clear solid (135.0 mg, 89% yield). **^{1}H NMR** (400 MHz, $CDCl_3$) δ = 7.14 – 7.06 (m, 2H), 6.84 (d, J = 8.1 Hz, 2H), 3.86 (t, J = 4.7 Hz, 4H), 3.14 – 3.07 (m, 4H), 2.21 (s, 2H), 1.23 (s, 12H). **^{13}C NMR** (101 MHz, $CDCl_3$) δ = 129.8, 116.4, 83.5, 67.1, 50.1, 24.9. **^{11}B NMR** (128 MHz, $CDCl_3$) δ = 33.4. **HRMS (ESI) m/z:** $[M+H]^+$ calculated for $C_{17}H_{27}BNO_3$ 304.2084; found 304.2086.

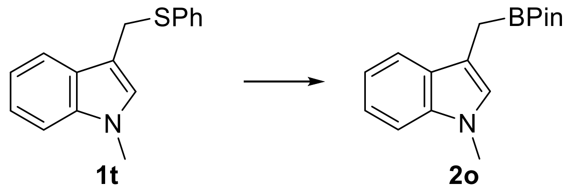

Synthesis according to **General Procedure 1** with 3 equiv. HBPin and for 4 F. Purified by column chromatography (19:1 P.E/EtOAc) obtained **2o,** inseparable from pinacolboronic acid (second peak in ^{11}B-NMR),[20] as a clear liquid (74 mg, 51% yield). **^{1}H NMR** (400 MHz, $CDCl_3$) δ = 7.58 (dt, J = 7.8, 1.0 Hz, 1H), 7.24 (dd, J = 2.8, 1.8 Hz, 1H), 7.19 (ddd, J = 8.2, 6.9, 1.2 Hz, 1H), 7.07 (ddd, J = 7.9, 6.8, 1.1 Hz, 1H), 6.92 (d, J = 1.1 Hz, 1H), 3.72 (s, 3H), 2.31 (s, 2H), 1.27 (s, 12H). **^{13}C NMR** (101 MHz, $CDCl_3$) δ = 136.9, 128.8, 126.6, 121.2, 119.2, 118.2, 110.1, 108.9, 83.4, 28.6 (d, J = 803.0 Hz), 24.9. **^{11}B NMR** (128 MHz, $CDCl_3$) δ = 33.8, 22.6.
The spectroscopic data matched those reported in the literature.[21]

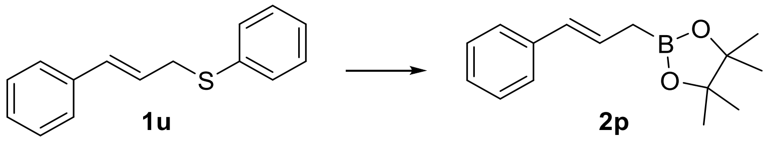

Synthesis according to **General Procedure 1** and purified by column chromatography (P.E/EtOAc 49:1) obtained **2p** as a clear liquid (48 mg, 39 % yield). **^{1}H NMR** (400 MHz, $CDCl_3$) δ = 7.35 – 7.31 (m, 2H), 7.30 – 7.23 (m, 2H), 7.21 – 7.11 (m, 1H), 6.37 (d, J = 16.0 Hz, 1H), 6.28 (dt, J = 15.7, 7.1 Hz, 1H), 1.87 (d, J = 7.1 Hz, 2H), 1.25 (s, 12H). **^{13}C NMR** (101 MHz, $CDCl_3$) δ = 138.2, 130.3, 128.4, 126.5, 126.3, 125.8, 83.4, 24.8. **^{11}B NMR** (128 MHz, $CDCl_3$) δ = 33.0.
The spectroscopic data matched those reported in the literature.[22]

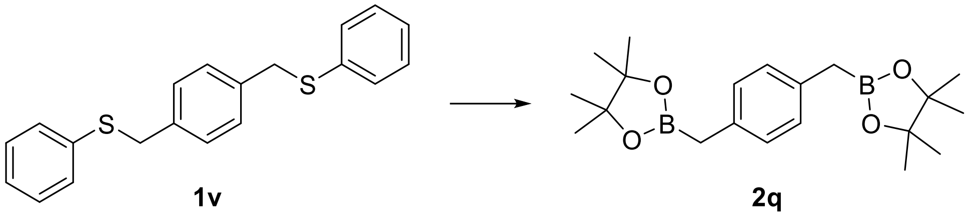

1v **2q**

Synthesis according to **General Procedure 1** with 3 equiv. HBPin and for 5 F. Purified by column chromatography (P.E/EtOAc 49:1) obtained **2q** as a white solid (143 mg, 80 % yield). **^{1}H NMR** (400 MHz, $CDCl_3$) δ = 7.05 (s, 4H), 2.23 (s, 4H), 1.22 (s, 24H). **^{13}C NMR** (101 MHz, $CDCl_3$) δ = 134.9, 129.1, 83.5, 24.9. **^{11}B NMR** (128 MHz, $CDCl_3$) δ = 33.1.
The spectroscopic data matched those reported in the literature.[17]

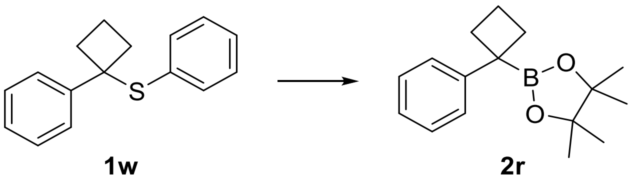

1w **2r**

Synthesis according to **General Procedure 1** and purified by column chromatography (P.E/EtOAc 49:1) obtained **2r**, as an amine-borane complex,[23] as a clear liquid (79 mg, 61% yield). **^{1}H NMR** (400 MHz, $CDCl_3$) δ = 7.33 – 7.27 (m, 2H), 7.14 (ddt, J=14.67, 6.97, 1.36, 3H), 2.59 (tt, J=8.40, 2.49, 2H), 2.40 – 2.27 (m, 2H), 2.16 – 2.01 (m, 1H), 1.94 – 1.82 (m, 1H), 1.23 (s, 12H). **^{13}C NMR** (101 MHz, CDCl3) δ = 149.0, 128.1, 125.6, 124.5, 83.5, 32.1, 24.6, 18.9. **^{11}B NMR** (128 MHz, $CDCl_3$) δ = 34.4.
The spectroscopic data matched those reported in the literature.[24]

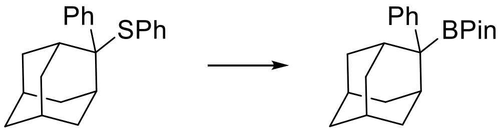

1x **2s**

Synthesis according to **General Procedure 1** and purified by column chromatography (P.E/EtOAc 49:1) obtained **2s** as a white solid (132 mg, 78% yield). **^{1}H NMR** (400 MHz, $CDCl_3$) δ = 7.42 – 7.38 (m, 2H), 7.31 (ddt, *J*=8.3, 3.6, 2.1, 2H), 7.13 (td, *J*=7.2, 1.7, 1H), 2.74 (s, 2H), 2.04 – 1.95 (m, 5H), 1.89 (d, *J*=12.5, 2H), 1.75 (s, 3H), 1.55 – 1.46 (m, 2H), 1.07 – 1.03 (m, 12H). **^{13}C NMR** (101 MHz, $CDCl_3$) δ = 144.2, 128.1, 127.3, 124.6, 83.0, 38.0, 37.5, 32.1, 31.1, 28.1, 27.5, 24.2. **^{11}B NMR** (128 MHz, $CDCl_3$) δ = 32.5. **HRMS (ESI) m/z:** $[M+H]^+$ calculated for $C_{22}H_{32}BO_2$ 339.2495; found 339.2496.

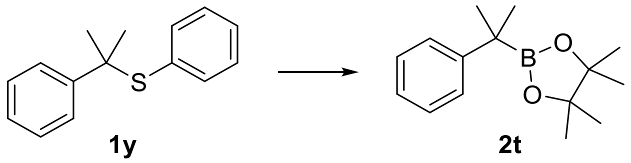

1y **2t**

Synthesis according to **General Procedure 1** and purified by column chromatography (P.E/EtOAc 49:1) obtained **2t** as a clear liquid (87 mg, 71% yield). **^{1}H NMR** (400 MHz, $CDCl_3$) δ = 7.35 – 7.26 (m, 4H), 7.16 – 7.12 (m, 1H), 1.36 (s, 6H), 1.21 (s, 12H). **^{13}C NMR** (101 MHz, $CDCl_3$) δ = 148.7, 128.2, 126.4, 125.1, 83.4, 25.7, 24.6. **^{11}B NMR** (128 MHz, $CDCl_3$) δ = 34.2.
The spectroscopic data matched those reported in the literature.[25]

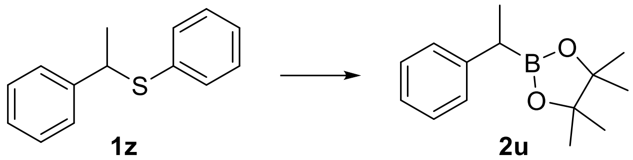

1z 2u

Synthesis according to **General Procedure 1** and purified by column chromatography (P.E/EtOAc 49:1) obtained **2u** as a clear liquid (80 mg, 80% yield). **^{1}H NMR** (500 MHz, $CDCl_3$) δ = 7.27 – 7.22 (m, 2H), 7.22 – 7.19 (m, 2H), 7.12 (ddt, J=7.7, 6.6, 1.5, 1H), 2.42 (q, J=7.5, 1H), 1.32 (d, J=7.5, 3H), 1.19 (d, J=6.8, 12H). **^{13}C NMR** (101 MHz, $CDCl_3$) δ = 145.1, 128.4, 127.9, 125.2, 83.4, 24.7 (d, J=4.1), 17.2. **^{11}B NMR** (128 MHz, $CDCl_3$) δ = 33.7. **HRMS (ESI) m/z:** $[M+H]^+$ calculated for $C_{14}H_{22}BO_2$ 233.1713; found 233.1719.

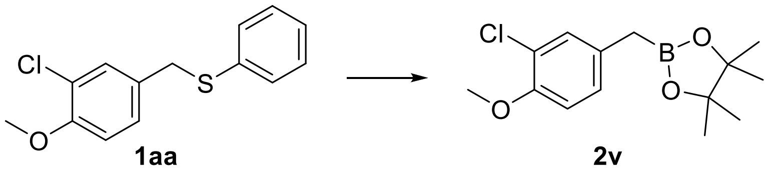

1aa 2v

Synthesis according to **General Procedure 1** and purified by column chromatography (P.E:EtOAc 9:1). **2v** was obtained as a clear liquid (18.5 mg, 29% yield). **^{1}H NMR** (400 MHz, $CDCl_3$) δ = 7.19 (s, 1H), 7.06 – 7.00 (m, 2H), 6.81 (d, *J*=8.3, 2H), 3.85 (s, 4H), 2.20 (s, 3H), 1.23 (s, 14H). **^{13}C NMR** (126 MHz, $CDCl_3$) δ = 152.7, 131.9, 130.8, 128.2, 122.1, 112.3, 83.7, 56.3, 24.9. **^{11}B NMR** (160 MHz, $CDCl_3$) δ = 32.9. **HRMS (ESI) m/z:** $[M+H]^+$ calculated for $C_{14}H_{22}BClO_3$ 284.1351; found 284.1347.

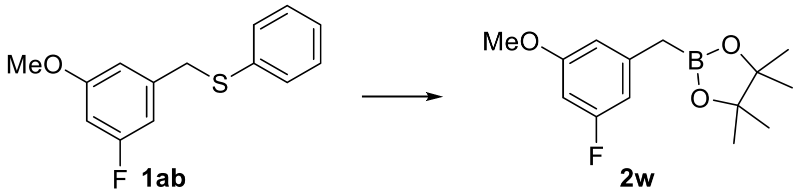

1ab 2w

Synthesis according to **General Procedure 1** and purified by column chromatography (P.E:EtOAc 9:1). **2w** was obtained as a clear liquid (76 mg, 57% yield). **^{1}H NMR** (500 MHz, $CDCl_3$) δ = 6.53 – 6.47 (m, 2H), 6.39 (dt, *J*=10.8, 2.4, 1H), 3.76 (s, 3H), 2.25 (s, 2H), 1.24 (s, 12H). **^{13}C NMR** (126 MHz, $CDCl_3$) δ = 163.7 (d, *J*=243.4), 160.7 (d, *J*=11.8), 141.9 (d, *J*=10.0), 110.6 (d, *J*=2.6), 108.4 (d, *J*=21.5), 98.5 (d, *J*=25.1), 83.7, 55.5, 29.9, 24.9. **^{11}B NMR** (160 MHz, $CDCl_3$) δ = 33.0. **^{19}F NMR** (377 MHz, $CDCl_3$) δ = -113.1. **HRMS (ESI) m/z:** $[M+H]^+$ calculated for $C_{14}H_{21}BFO_3$ 268.1568; found 267.1567.

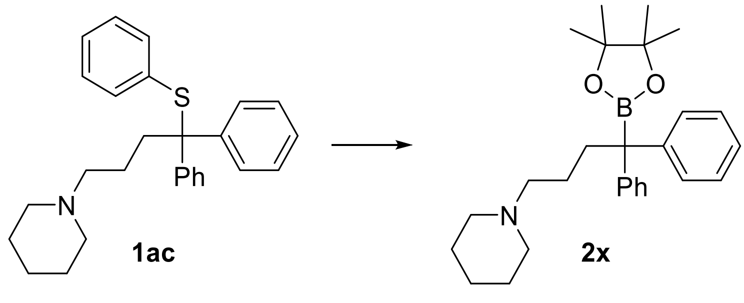

1ac 2x

Synthesis according to **General Procedure 1** and purified by column chromatography (P.E/EtOAc 9:1) obtained **2x** as a clear liquid (75 mg, 36 % yield). **^{1}H NMR** (400 MHz, $CDCl_3$) δ = 7.28 (d, J=3.9, 4H), 7.25 – 7.13 (m, 6H), 2.88 – 2.80 (m, 2H), 2.74 – 2.67 (m, 2H), 2.66 – 2.59 (m, 2H), 2.15 – 2.02 (m, 2H), 1.87 – 1.73 (m, 2H), 1.60 (ddt, J=11.6, 7.6, 4.2, 2H), 1.53 – 1.46 (m, 4H), 1.18 (s, 12H). **^{13}C NMR** (101 MHz, $CDCl_3$) δ = 146.0, 129.4, 128.1, 125.7, 83.9, 58.1, 35.4, 24.5, 20.7, 20.5. **^{11}B NMR** (128 MHz, $CDCl_3$) δ = -12.3. **HRMS (ESI) m/z:** $[M+H]^+$ calculated for $C_{27}H_{39}BnO_2$ 420.3074; found 420.3083.

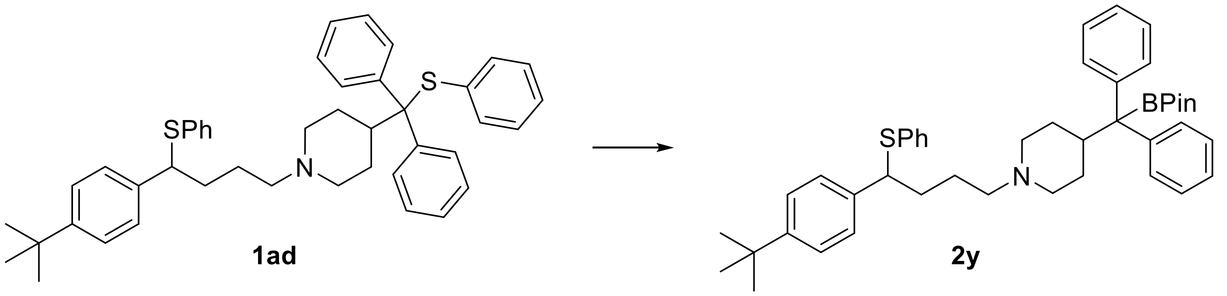

Synthesis according to **General Procedure 1** with 3 equiv. HBPin. Purified by column chromatography (P.E/EtOAc 19:1) obtained **2y** as a clear liquid (100 mg, 30 % yield). **^{1}H NMR** (400 MHz, $CDCl_3$) δ = 7.39 – 7.18 (m, 19H), 4.16 (t, *J*=6.9, 1H), 2.96 (t, *J*=10.2, 2H), 2.76 – 2.47 (m, 5H), 1.99 – 1.78 (m, 6H), 1.61 (d, *J*=13.7, 2H), 1.35 (s, 9H), 1.10 (s, 12H). **^{13}C NMR** (101 MHz, $CDCl_3$) δ = 150.2, 142.0, 138.3, 135.0, 132.5, 130.7, 128.8, 127.6, 127.3, 127.2, 125.9, 125.5, 83.8, 67.8, 59.6, 59.3, 53.0, 38.6, 34.5, 34.2, 31.4, 24.2, 21.2. **^{11}B NMR** (128 MHz, $CDCl_3$) δ = -15.7. **HRMS (ESI) m/z:** $[M+H]^+$ calculated for $C_{44}H_{56}BNO_2S$ 673.4125; found 674.4199.

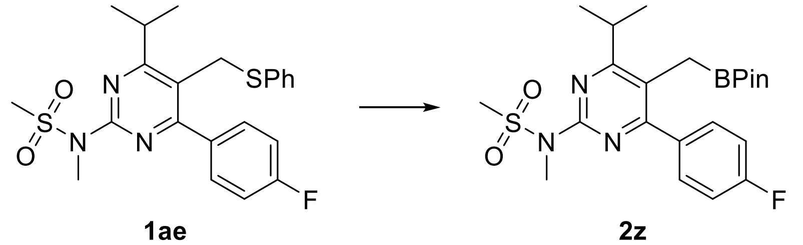

Synthesis according to **General Procedure 1** and purified by column chromatography (P.E/EtOAc 9:1) obtained **2z** as a clear liquid (174 mg, 75% yield). **^{1}H NMR** (400 MHz, $CDCl_3$) δ = 7.60 – 7.53 (m, 2H), 7.16 – 7.06 (m, 2H), 3.52 (d, *J*=11.2, 6H), 3.13 (p, *J*=6.6, 1H), 2.19 (s, 2H), 1.27 (d, *J*=6.6, 6H), 1.23 (s, 12H). **^{13}C NMR** (101 MHz, $CDCl_3$) δ = 174.8, 164.8, 164.4, 161.9, 156.4, 135.4 (d, J=3.3), 131.1 (d, J=8.4), 128.4 (d, J=162.5), 120.9, 115.3 (d, J=21.5), 84.1, 42.5, 33.3, 32.1, 24.8, 21.7. **^{11}B NMR** NMR (128 MHz, $CDCl_3$) δ = 33.5. **^{19}F NMR** (377 MHz, $CDCl_3$) δ = -112.5. **HRMS (ESI) m/z:** $[M+Na]^+$ calculated for $C_{22}H_{31}BFN_3O_4SNa$ 486.2010; found 486.2027.

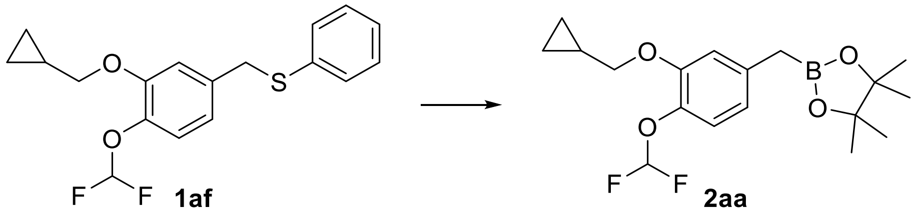

Synthesis according to **General Procedure 1** and purified by column chromatography (P.E/EtOAc 19:1) obtained **2aa** as a clear liquid (122 mg, 69% yield). **^{1}H NMR** (400 MHz, $CDCl_3$) δ = 7.01 (d, *J*=8.1, 1H), 6.78 (d, *J*=2.1, 1H), 6.72 (dd, *J*=8.1, 2.0, 1H), 6.57 (t, *J*=76.0, 1H), 3.84 (d, *J*=6.9, 2H), 2.24 (s, 2H), 1.23 (s, 13H), 0.65 – 0.59 (m, 2H), 0.36 – 0.31 (m, 2H). **^{13}C NMR** (101 MHz, $CDCl_3$) δ = 150.2, 138.1 (t, J=3.2), 137.6, 122.6, 121.6, 119.2, 116.7, 115.4, 114.1, 83.7, 73.8, 24.9, 10.3, 3.3. **^{11}B NMR** (128 MHz, $CDCl_3$) δ = 33.1. **^{19}F NMR** (377 MHz, $CDCl_3$) δ = -81.3. **HRMS (ESI) m/z:** $[M+H]^+$ calculated for $C_{16}H_{26}BF_2O_4$ 355.1892; found 355.1894.

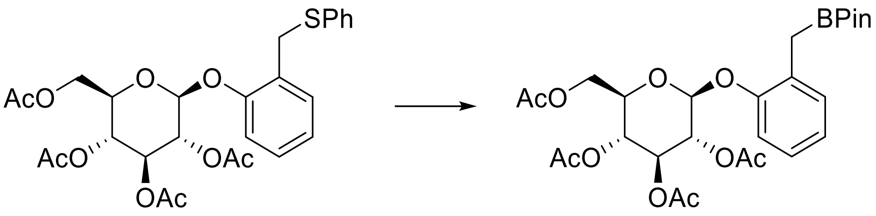

1ag **2ab**

Synthesis according to **General Procedure 1** and purified by column chromatography (P.E/EtOAc 7:3) obtained **2ab** as a clear liquid (135 mg, 48 % yield). **^{1}H NMR** (500 MHz, $CDCl_3$) δ = 7.21 – 6.95 (m, 4H), 5.38 – 5.22 (m, 2H), 5.21 – 5.13 (m, 1H), 5.03 (d, J=7.5, 0.3H), 4.99 (d, J=7.5, 0.6H), 4.28 (ddd, J=12.2, 10.0, 5.4, 1H), 4.18 (dt, J=12.2, 2.2, 1H), 3.86 (ddd, J=10.1, 5.5, 2.5, 0.3H), 3.80 (ddd, J=10.0, 5.4, 2.5, 0.6H), 2.24 – 2.14 (m, 2H), 2.10 – 2.02 (m, 12H), 1.26 (s, 2H), 1.2Z3 (s, 2H), 1.23 (s, 4H), 1.22 (s, 4H). **^{13}C NMR** (126 MHz, $CDCl_3$) δ = 170.7, 170.7, 170.4, 170.4, 169.6, 169.4, 169.3, 155.3, 154.9, 130.9, 130.1, 129.9, 129.0, 128.2, 127.0, 126.3, 123.8, 123.4, 123.3, 117.0, 115.4, 115.2, 100.1, 99.5, 83.5, 83.2, 73.2, 72.9, 72.1, 72.0, 71.3, 71.2, 68.6, 68.6, 62.2, 62.1, 25.0, 25.0, 24.7, 24.7, 20.9, 20.8, 20.8, 20.7. **^{11}B NMR** (128 MHz, $CDCl_3$) δ = 22.7. **HRMS (ESI) m/z:** $[M+Na]^+$ calculated for $C_{27}H_{37}BO_{12}Na$ 587.2276; found 587.2284.

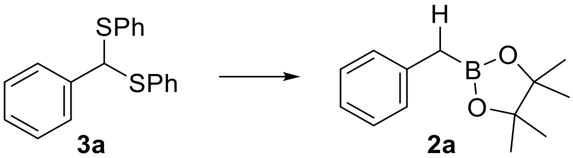

3a **2a**

Synthesis according to **General Procedure 1** but for 4 F and with 3 equivalent pinacolborane. Purified by column chromatography (P.E/EtOAc 49:1) obtained **2a** as a clear liquid (66 mg, 60% yield). **^{1}H NMR** (400 MHz, $CDCl_3$) δ = 7.26 – 7.16 (m, 4H), 7.13 – 7.09 (m, 1H), 2.29 (s, 2H), 1.22 (s, 13H). **^{13}C NMR** (101 MHz, $CDCl_3$) δ = 138.7, 129.1, 128.4, 124.9, 83.5, 24.8. **^{11}B NMR** (128 MHz, $CDCl_3$) δ = 33.2.
The spectroscopic data matched those reported in the literature.[13]

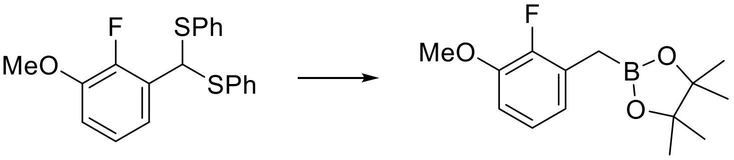

3b **2ac**

Synthesis according to **General Procedure 1** but for 4 F and 3 equivalent pinacolborane. Purified by column chromatography (P.E/EtOAc 9:1) obtained **2ac** as a clear liquid (36 mg, 27% yield). **^{1}H NMR** 1H NMR (400 MHz, $CDCl_3$) δ = 6.93 (td, J=7.9, 1.5, 1H), 6.80 – 6.73 (m, 2H), 3.86 (s, 3H), 2.26 (d, J=2.4, 2H), 1.24 (s, 12H). **^{13}C NMR** (101 MHz, $CDCl_3$) δ = 150.8 (d, J=242.5), 147.7, 147.6, 127.1 (d, J=14.4), 123.5 (d, J=4.8), 122.9 (d, J=3.8), 110.4 (d, J=1.6), 83.7, 56.3, 24.8. **^{11}B NMR** (128 MHz, $CDCl_3$) δ = 33.4. **^{19}F NMR** (377 MHz, $CDCl_3$) δ = -139.7. **HRMS (ESI) m/z:** $[M+H]^+$ calculated for $C_{14}H_{21}BFO_3$ 267.1568; found 267.1564.

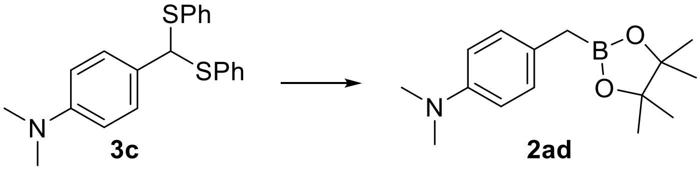

3c **2ad**

Synthesis according to **General Procedure 1** but for 4 F and with 3 equivalent pinacolborane. Purified by column chromatography (P.E/EtOAc 4:1) obtained **2ad** as a clear liquid (50 mg, 39% yield). **^{1}H NMR** (400 MHz, $CDCl_3$) δ = 7.07 (d, J=8.6, 2H), 6.68 (d, J=8.6, 2H), 2.89 (s, 6H), 2.19 (s, 2H), 1.23 (s, 12H). **^{13}C NMR** (101 MHz, $CDCl_3$) δ = 148.6, 129.7, 126.8, 113.5, 83.4, 41.2, 24.9. **^{11}B NMR** (128 MHz, $CDCl_3$) δ = 33.5. **HRMS (ESI) m/z:** $[M+H]^+$ calculated for $C_{15}H_{25}BNO_2$ 262.1978; found 262.1975.

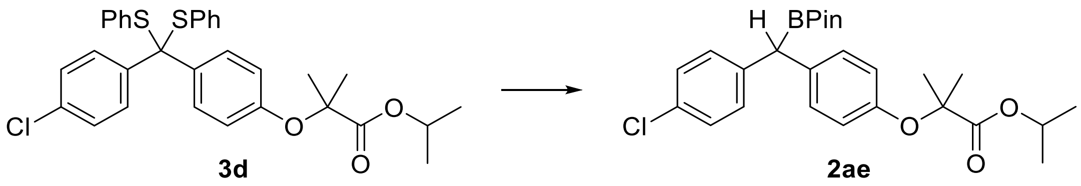

Synthesis according to **General Procedure 1** but for 4 F and with 3 equivalent pinacolborane. Purified by column chromatography (P.E/EtOAc 49:1) obtained **2ae** as a clear liquid (115 mg, 49% yield). **^{1}H NMR** (400 MHz, $CDCl_3$) δ = 7.2 (d, J=8.5, 2H), 7.1 (d, J=8.5, 2H), 7.1 (d, J=8.6, 2H), 6.8 – 6.7 (m, 2H), 5.1 (hept, J=6.2, 1H), 3.7 (s, 1H), 1.6 (s, 6H), 1.2 (dd, J=6.8, 2.1, 18H). **^{13}C NMR** (101 MHz, $CDCl_3$) δ = 174.0, 153.8, 141.1, 134.9, 131.4, 130.4, 129.8, 128.5, 119.2, 83.9, 79.1, 69.0, 25.5, 24.7, 21.7. **^{11}B NMR** (128 MHz, $CDCl_3$) δ = 32.6. **HRMS (ESI) m/z:** $[M+H]^+$ calculated for $C_{26}H_{35}BClO_5$ 473.2266; found 473.2207.

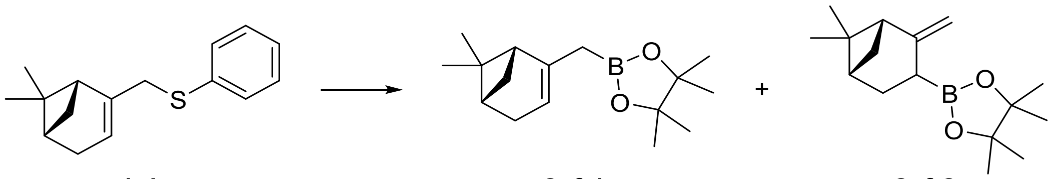

Synthesis according to **General Procedure 1** and purified by column chromatography (P.E/EtOAc 49:1) obtained as a clear liquid (98 mg, 75% yield). NMR contains a mixture of regioisomers. The regioselectivity was determined by allylic protons in ^{1}H NMR spectrum after purification (**2af-1**/**2af-2** 13:1). Only the allylic peak of the minor is reported since the aliphatic peaks overlap and are therefore reported together. **^{1}H NMR** (400 MHz, $CDCl_3$) **Major:** δ = 5.18 (tt, J=3.0, 1.5, 1H) **Minor:** δ = 4.61 (t, J=1.8, 0.1H), 4.59 (t, J=1.9, 0.1H) **All the other peaks:** δ = 2.45 – 2.40 (m, 0.1H), 2.31 (dt, J=8.5, 5.6, 1H), 2.26 – 2.11 (m, 2H), 2.04 (ttd, J=5.9, 2.8, 1.3, 1H), 1.98 (td, J=5.6, 1.5, 1H), 1.71 – 1.58 (m, 2H), 1.24 (s, 3H), 1.22 (s, 12H), 1.17 (d, J=8.5, 1H), 0.85 (s, 3H). **^{13}C NMR** (101 MHz, $CDCl_3$) δ = 144.7, 115.8, 83.2, 47.4, 40.7, 38.1, 31.8, 31.4, 26.5, 25.0, 24.9, 21.2. **^{11}B NMR** (128 MHz, $CDCl_3$) δ = 33.0. **HRMS (ESI) m/z:** $[M+H]^+$ calculated for $C_{16}H_{28}BO_2$ 263.2182; found 263.2185.

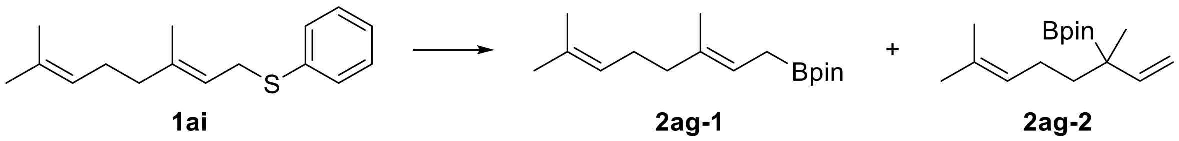

Synthesis according to **General Procedure 1** and purified by column chromatography (P.E/EtOAc 49:1) obtained **2ag-1** as a clear liquid (61 mg, 46% yield). Traces of **2ag-2** were observed after purification. **^{1}H NMR** (400 MHz, $CDCl_3$) δ = 5.26 (tdd, J=6.3, 2.6, 1.3, 1H), 5.12 (tdd, J=5.5, 3.0, 1.5, 1H), 2.12 – 1.97 (m, 4H), 1.69 (d, J=1.4, 3H), 1.65 – 1.58 (m, 8H), 1.26 (s, 12H). **^{13}C NMR** (101 MHz, $CDCl_3$) δ = 135.1, 131.1, 124.5, 118.5, 83.0, 39.8, 26.8, 25.7, 24.8, 17.7, 15.9. **^{11}B NMR** (128 MHz, $CDCl_3$) δ = 33.4. **HRMS (ESI) m/z:** $[M+H]^+$ calculated for $C_{16}H_{30}BO_2$ 265.2339; found 265.2343.

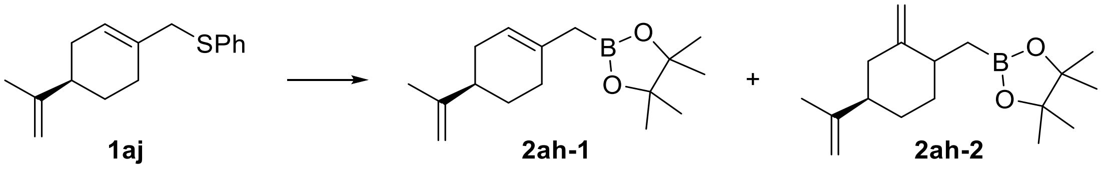

Synthesis according to **General Procedure 1** and purified by column chromatography (P.E/EtOAc 49:1) obtained **2z-1** and **2z-2** , as an amine-borane complex,[23] as a clear liquid (59 mg, 41% yield). NMR contains a mixture of regioisomers. The regioselectivity was determined by allylic protons in ^{1}H NMR spectrum after purification. Due to allylic protons overlapping calculations were done by subtracting the area of the major product to get the area of the minor product (**2ah-1/2ah-2** 4:1). **^{1}H NMR** (400 MHz, $CDCl_3$) δ = 5.42 – 5.34 (m, 1H), 4.67 (d, J=1.48, 2H), 2.37 – 1.83 (m, 5H), 1.84 – 1.66 (m, 4H), 1.60 (s, 2H), 1.44 (dtd, J=17.87, 11.89, 6.02, 1H), 1.23 (s, 12H). **^{13}C NMR** (101 MHz, $CDCl_3$) δ = 150.4 – 150.2 (m), 134.2, 120.4, 108.4, 83.2, 41.1, 31.1, 30.9, 28.1, 24.8, 24.8, 20.9. **^{11}B NMR** (128 MHz, $CDCl_3$) δ = 33.3. **HRMS (ESI) m/z:** $[M+H]^+$ calculated for $C_{16}H_{28}BO_2$ 263.2182; found 263.2184.

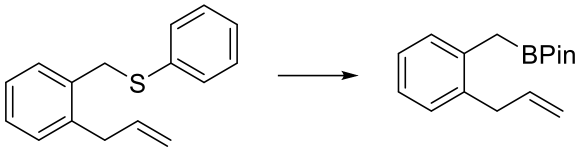

1ak **2ac**

Synthesis according to **General Procedure 1** and purified by column chromatography (P.E/EtOAc 49:1) obtained **2ac** as a clear liquid (99 mg, 77 % yield). **^{1}H NMR** (400 MHz, $CDCl_3$) δ = 7.20 – 7.05 (m, 4H), 5.97 (ddtd, J = 16.6, 10.1, 6.4, 1.2 Hz, 1H), 5.11 – 4.97 (m, 2H), 3.39 (dt, J = 6.4, 1.6 Hz, 2H), 2.29 (s, 2H), 1.23 (s, 12H). **^{13}C NMR** (101 MHz, $CDCl_3$) δ = 137.6, 137.5, 137.1, 130.1, 129.3, 126.4, 125.4, 115.8, 83.5, 37.8, 24.9. **^{11}B NMR** (128 MHz, $CDCl_3$) δ = 33.2. **HRMS (ESI) m/z:** $[M+H]^+$ calculated for $C_{16}H_{24}BO_2$ 259.1869; found 259.1863.

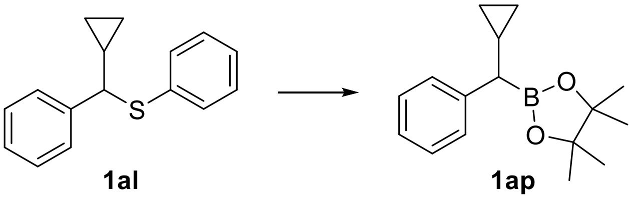

1al **1ap**

Synthesis according to **General Procedure 1**. Analyzed by qNMR (see Section S9),**1ap** observed to a 79% yield. **^{1}H NMR** (400 MHz, $CDCl_3$) δ = 7.31 – 7.22 (m, 4H), 7.14 (ddt, *J*=6.5, 4.9, 1.7, 1H), 1.71 (d, *J*=9.7, 1H), 1.21 (d, *J*=1.7, 12H), 0.56 (dddd, *J*=9.3, 8.0, 5.5, 4.2, 1H), 0.46 (dddd, *J*=9.1, 7.4, 4.8, 3.8, 1H), 0.24 (dq, *J*=9.5, 4.9, 2H), 0.08 (dq, *J*=9.5, 4.9, 2H). **HRMS (ESI) m/z:** $[M+H]^+$ calculated for $C_{16}H_{24}BO_2$ 259.1869; found 259.1869.

The spectroscopic data matched those reported in the literature.[26]

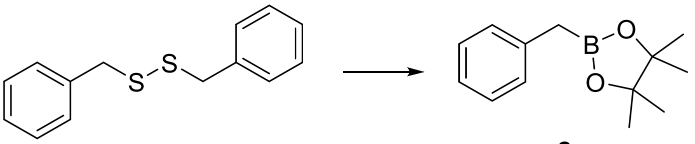

2a

Synthesis according to **General Procedure 2** and purified by column chromatography (P.E/EtOAc 49:1) obtained **2a** as a clear liquid (168 mg, 77% yield). **^{1}H NMR** (400 MHz, $CDCl_3$) δ = 7.26 – 7.16 (m, 4H), 7.13 – 7.09 (m, 1H), 2.29 (s, 2H), 1.22 (s, 13H). **^{13}C NMR** (101 MHz, $CDCl_3$) δ = 138.7, 129.1, 128.4, 124.9, 83.5, 24.8. **^{11}B NMR** (128 MHz, $CDCl_3$) δ = 33.2.
The spectroscopic data matched those reported in the literature.[13]

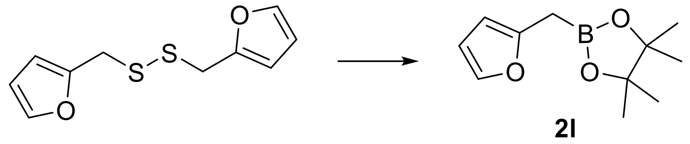

2l

Synthesis according to **General Procedure 2** and purified by column chromatography (P.E/EtOAc 49:1) obtained **2l** as a clear liquid (72 mg, 35% yield). **^{1}H NMR** (400 MHz, $CDCl_3$) δ = 7.21 – 7.18 (m, 1H), 6.18 (dt, J=3.3, 1.5, 1H), 5.96 (dt, J=3.1, 1.1, 1H), 2.23 (s, 2H), 1.20 (s, 12H). **^{13}C NMR** (101 MHz, $CDCl_3$) δ = 152.5, 140.7, 110.4, 105.4, 83.8, 24.9. **^{11}B NMR** (128 MHz, $CDCl_3$) δ = 32.7. **HRMS (ESI) m/z:** $[M+H]^+$ calculated for $C_{11}H_{18}BO_2$ 209.1349; found 209.1340.

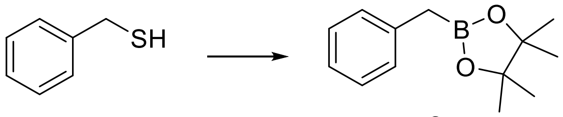

2a

Synthesis according to **General Procedure 2** but for 3 F and with 3 equivalents of HBpin. Purified by column chromatography (P.E/EtOAc 49:1) obtained **2a** as a clear liquid (87 mg, 80% yield). **^{1}H NMR** (400 MHz, $CDCl_3$) δ = 7.26 – 7.16 (m, 4H), 7.13 – 7.09 (m, 1H), 2.29 (s, 2H), 1.22 (s, 13H). **^{13}C NMR** (101 MHz, $CDCl_3$) δ = 138.7, 129.1, 128.4, 124.9, 83.5, 24.8. **^{11}B NMR** (128 MHz, $CDCl_3$) δ = 33.2.
The spectroscopic data matched those reported in the literature.[13]

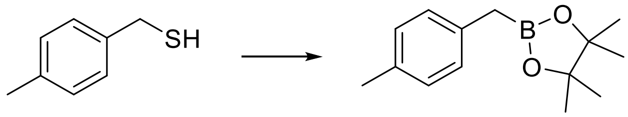

2ai

Synthesis according to **General Procedure 2** but for 4 F and with 3 equivalents of HBpin. Analyzed by qNMR (see Section S9), **2ai** was observed in a 75% yield.
The spectroscopic data matched those reported in the literature.[27]

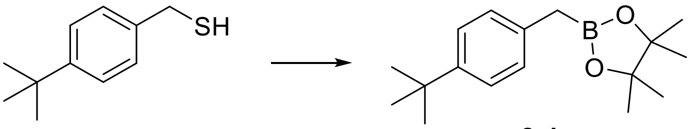

2aj

Synthesis according to **General Procedure 2** but for 4 F and with 3 equivalents of HBpin. Analyzed by qNMR (see Section S9), **2aj** was observed in a 71% yield.
The spectroscopic data matched those reported in the literature.[28]

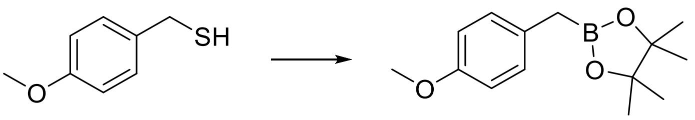

2ak

Synthesis according to **General Procedure 2** but for 4 F and with 3 equivalents of HBpin. Analyzed by qNMR (see Section S9), **2ak** observed to a 45% yield.
The spectroscopic data matched those reported in the literature.[27]

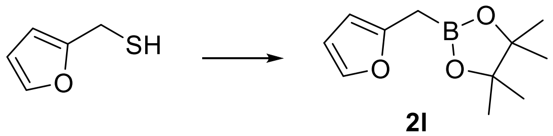

2l

Synthesis according to **General Procedure 2** but for 3 F and with 3 equivalents of HBpin. Purified by column chromatography (P.E/EtOAc 49:1) obtained **2l** as a clear liquid (56 mg, 54% yield). **^{1}H NMR** (400 MHz, $CDCl_3$) δ = 7.21 – 7.18 (m, 1H), 6.18 (dt, J=3.3, 1.5, 1H), 5.96 (dt, J=3.1, 1.1, 1H), 2.23 (s, 2H), 1.20 (s, 12H). **^{13}C NMR** (101 MHz, $CDCl_3$) δ = 152.5, 140.7, 110.4, 105.4, 83.8, 24.9. **^{11}B NMR** (128 MHz, $CDCl_3$) δ = 32.7. **HRMS (ESI) m/z:** $[M+H]^+$ calculated for $C_{11}H_{18}BO_2$ 209.1349; found 209.1340.

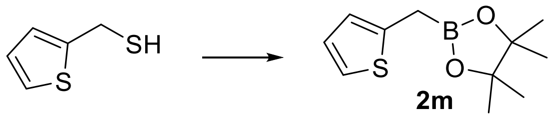

2m

Synthesis according to **General Procedure 2** but for 4 F and with 3 equivalents of HBpin. Analyzed by qNMR (see Section S9), **2m** was observed in a 74% yield.
The spectroscopic data matched those reported in the literature.[27]

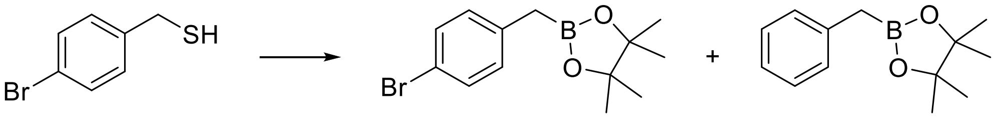

2b **2a**

Synthesis according to **General Procedure 2** but for 3 F and with 3 equivalents of HBpin. Purified by column chromatography (P.E/EtOAc 49:1), Product **2b** was not obtained rather the dehalogenated product **2a** was obtained as a clear liquid (33 mg, 30% yield). **^{1}H NMR** (400 MHz, $CDCl_3$) δ = 7.26 – 7.16 (m, 4H), 7.13 – 7.09 (m, 1H), 2.29 (s, 2H), 1.22 (s, 13H). **^{13}C NMR** (101 MHz, $CDCl_3$) δ = 138.7, 129.1, 128.4, 124.9, 83.5, 24.8. **^{11}B NMR** (128 MHz, $CDCl_3$) δ = 33.2.
The spectroscopic data matched those reported in the literature.[13]

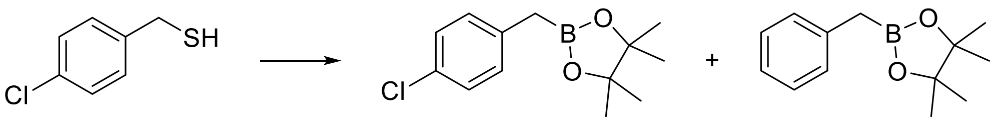

2c **2a**

Synthesis according to **General Procedure 2** but for 3 F and with 3 equivalents of HBpin. Purified by column chromatography (P.E/EtOAc 49:1), Product **2c** was not obtained rather the dehalogenated product **2a** was obtained as a clear liquid (28 mg, 26% yield). **^{1}H NMR** (400 MHz, $CDCl_3$) δ = 7.26 – 7.16 (m, 4H), 7.13 – 7.09 (m, 1H), 2.29 (s, 2H), 1.22 (s, 13H). **^{13}C NMR** (101 MHz, $CDCl_3$) δ = 138.7, 129.1, 128.4, 124.9, 83.5, 24.8. **^{11}B NMR** (128 MHz, $CDCl_3$) δ = 33.2.
The spectroscopic data matched those reported in the literature.[13]

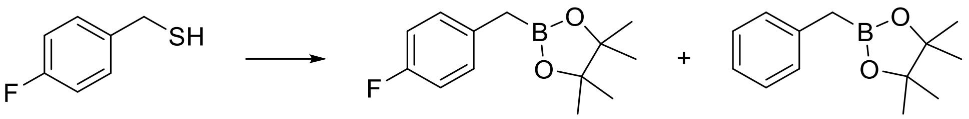

2d **2a**

Synthesis according to **General Procedure 2** but for 3 F and with 3 equivalents of HBpin. Purified by column chromatography (P.E/EtOAc 49:1), Product **2d** was obtained as a clear liquid (8 mg, 6% yield), dehalogenated product **2a** was also obtained as a clear liquid (21 mg, 19% yield). ^{1}**H NMR** (400 MHz, $CDCl_3$) δ = 7.26 – 7.16 (m, 4H), 7.13 – 7.09 (m, 1H), 2.29 (s, 2H), 1.22 (s, 13H). ^{13}**C NMR** (101 MHz, $CDCl_3$) δ = 138.7, 129.1, 128.4, 124.9, 83.5, 24.8. ^{11}**B NMR** (128 MHz, $CDCl_3$) δ = 33.2.
The spectroscopic data matched those reported in the literature.[13]

S6 Scaled-up procedure for desulfurative borylation of thioethers

Borylation of **1a** (1.2 g, 6.0 mmol) was carried out using an IKA® ElectraSyn 20 mL vial, leading to the formation of **2a** in 77% yield. The electrode area immersed in the solvent was increased 3.5 times in comparison to **General procedure 1**, prompting an adjustment of the current to 35 mA to maintain a consistent current density of ~10 mA/cm^2. Furthermore, the concentration of **1a** was increased to 0.39 M, while the supporting electrolyte, NBu_4BH_4, remained at 0.1 M.

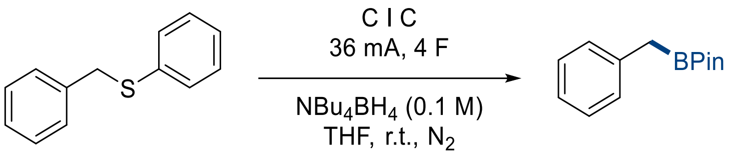

1a, 6 mmol **2a**, 1 g (77%)

Scale up procedure: To an oven-dried 20 mL-ElectraSyn vial equipped with a magnetic stir bar, graphite electrodes, thioether **1a** (1.0 equiv., 6 mmol 1.2 g) and Bu_4NBH_4 (0.3 equiv., 1.8 mmol, 700 mg) were added. The mixture was evacuated and refilled with nitrogen three times before addition of anhydrous and stabilizer-free THF (18 mL) followed by HBPin (3 equiv., 18 mmol, 2.6 mL). The reaction was carried out by applying 35 mA at room temperature for 4 F, stirring at 750 rpm. After electrolysis, the crude reaction mixture was added to an aqueous solution of NH_4Cl and extracted with EtOAc (x3). The combined organic phases were dried over sodium sulfate and purified by column chromatography (P.E./EtOAc 49:1) **2a** was obtained as a clear liquid (1 g, 77% yield).

S7 Unsuccessful and suboptimal substrates

Table S8. Unsuccessful and suboptimal substrates

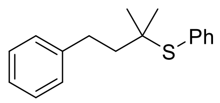

No conversion

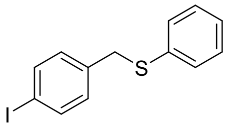

C-I bond cleavage

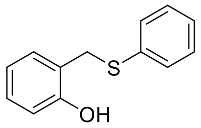

No conversion

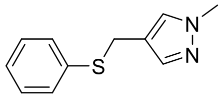

No borylation product obtained

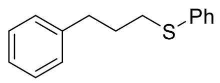

No conversion

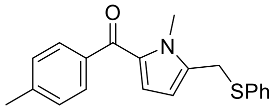

No borylation product obtained

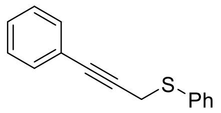

Alkyne reduction

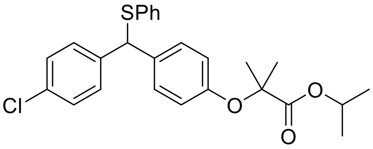

No borylation product obtained

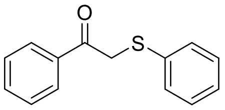

Reduction of ketone

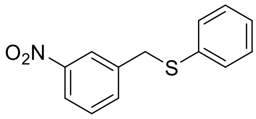

messy NMR,
product not
detected in MS

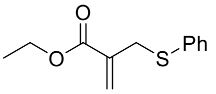

No borylation product obtained

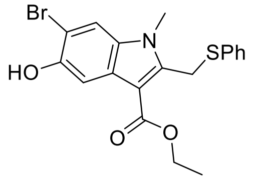

No borylation product obtained

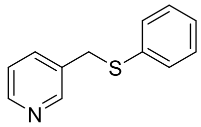

No borylation product obtained

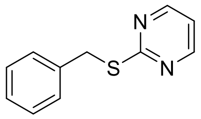

No borylation product obtained

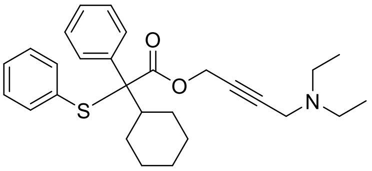

Hydrodesulfurization

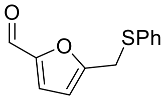

No borylation product obtained

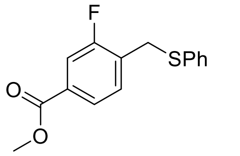

Hydrodesulfurization

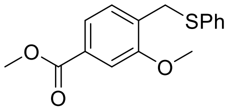

Hydrodesulfurization

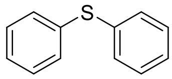

Obtained 4% of product,
according to general procedure 2
8 F 6 eq HBpin.

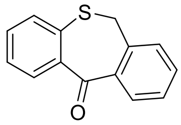

Product not observed on MS

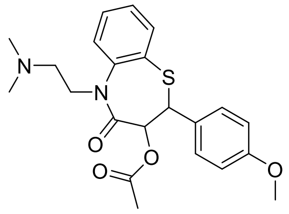

Product not observed on MS

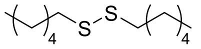

No borylation product obtained

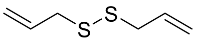

No borylation product obtained

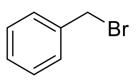

Product not obtained
(88% conversion)

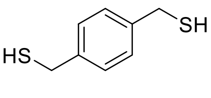

Obtained 5% product

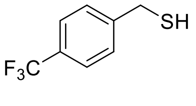

Product not obtained

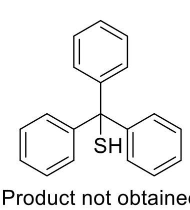

Product not obtained

S8 Computational studies

All computations were performed using the Gaussian16.C.01. All geometries were optimized using the B3LYP functional[29] with the 6-311++G** basis set, the iefpcm solvation model with standard settings for tetrahydrofuran, and zero-point vibrational energies, thermal corrections to the enthalpy, and entropic corrections using the harmonic oscillator approximation to the vibrations. Gibbs free energies were then calculated by the summing the terms as G = E(6-311++G**) + G_{solv} + ZPE + ⍰H – TS where T was set to 298K. All geometries were characterized as minima or saddle points on the potential-energy surface (PES) by using the sign of the eigenvalues of the force constant matrix obtained from a frequency calculation. Transition states with one imaginary frequency were confirmed to describe the correct movement on the PES by mode analysis and by intrinsic reaction coordinate (IRC) calculations connecting the correct reactants and products.

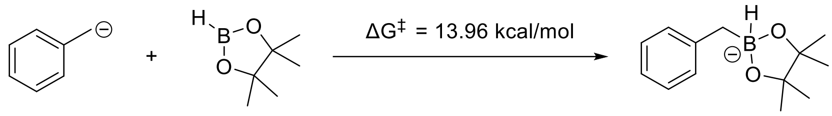

IM

Coordinates IM

C	2.20578300	-0.59561200	-0.34407300
C	3.09276100	-1.09901300	0.62485300
C	4.30488800	-0.46905000	0.90492200
C	4.67241000	0.69211400	0.22166300
C	3.80653000	1.20905700	-0.74447300
C	2.59639400	0.57365900	-1.02016400
H	2.82158400	-2.00118300	1.16450700
H	4.96642900	-0.88665900	1.65773100
H	5.61560800	1.18298300	0.43594400
H	4.07593100	2.11003500	-1.28706300
H	1.93319000	0.98926700	-1.77208500
C	0.88149500	-1.23999200	-0.59181300
H	0.53594700	-1.02170800	-1.60938900
H	0.97097300	-2.32987600	-0.49981900
H	0.07581600	-1.04053800	1.62799100
C	-2.02618200	0.84106300	0.39057300
C	-2.54933400	-0.49006000	-0.26286900
B	-0.29731400	-0.76521300	0.47030800
C	-3.95401100	-0.90714500	0.18006900
H	-4.68879200	-0.12905900	-0.05176500
H	-4.25135200	-1.82054600	-0.34388100
H	-3.98377100	-1.10887300	1.25156600
C	-2.50510400	-0.42499700	-1.80223000
H	-2.64618200	-1.43481200	-2.19731700
H	-3.28897400	0.21953300	-2.21247300
H	-1.53527600	-0.05760600	-2.14041500
C	-2.44058800	0.95556100	1.87047400
H	-1.89007700	1.78460600	2.32355400
H	-3.51112900	1.15015600	1.98532300
H	-2.18821800	0.04187700	2.41133200
C	-2.43946100	2.11654600	-0.34593900
H	-3.52928400	2.20053700	-0.40823100
H	-2.06128300	2.99186600	0.19050500

H	-2.02769800	2.13734900	-1.35554500
O	-1.60656400	-1.45195400	0.19334200
O	-0.61598200	0.68644500	0.30566200

TS cooridnates

C	-2.49502500	-0.50126600	0.59193700
C	-3.25289500	-1.19879200	-0.41748400
C	-4.41413000	-0.67836600	-0.96838100
C	-4.92569200	0.57081200	-0.57691100
C	-4.20634700	1.28329500	0.40031300
C	-3.04469400	0.78031400	0.96179100
H	-2.89810500	-2.17208900	-0.74843300
H	-4.94106000	-1.25819500	-1.72357200
H	-5.83485300	0.97052000	-1.01169500
H	-4.56826200	2.25581200	0.72806300
H	-2.52194400	1.36566900	1.71475300
C	-1.30734400	-1.00168600	1.13602700
H	-0.81433400	-0.47935400	1.94905100
H	-0.97173800	-2.00854500	0.91449600
H	-0.38224800	-0.55544100	-1.54628300
C	2.38838100	0.89218200	-0.17374200
C	2.63970800	-0.64825200	0.08398100
B	0.57337700	-0.33854500	-0.88202800
C	3.98035900	-1.17580900	-0.42255300
H	4.81071600	-0.68634400	0.09418100
H	4.04639600	-2.24843100	-0.22432200
H	4.09789500	-1.02142600	-1.49503500
C	2.44031600	-1.06717800	1.54523000
H	2.47043600	-2.15769300	1.60608200
H	3.23146400	-0.66804200	2.18474400
H	1.47433000	-0.73481600	1.92534200
C	3.15360900	1.43894000	-1.38558600
H	2.79671200	2.44852200	-1.60201800
H	4.22845800	1.48939900	-1.19458000
H	2.98541400	0.82363600	-2.27187800
C	2.62020800	1.79388500	1.03649700
H	3.66213900	1.74389900	1.36531800
H	2.40197700	2.82977000	0.76573900
H	1.97662100	1.51923000	1.87208600
O	1.57891200	-1.27059400	-0.69866100
O	0.97413500	0.93411500	-0.52116600

S9 Quantitative NMR (qNMR)

Unless otherwise noted, qNMRs were carried by adding 100uL of a 0.0333M 1,3,5-trimethoxybenzene (TMB) solution in $CDCl_3$ as internal standard to the reaction mixture after electrolysis and concentration in *vacuo*, followed by NMR analysis.

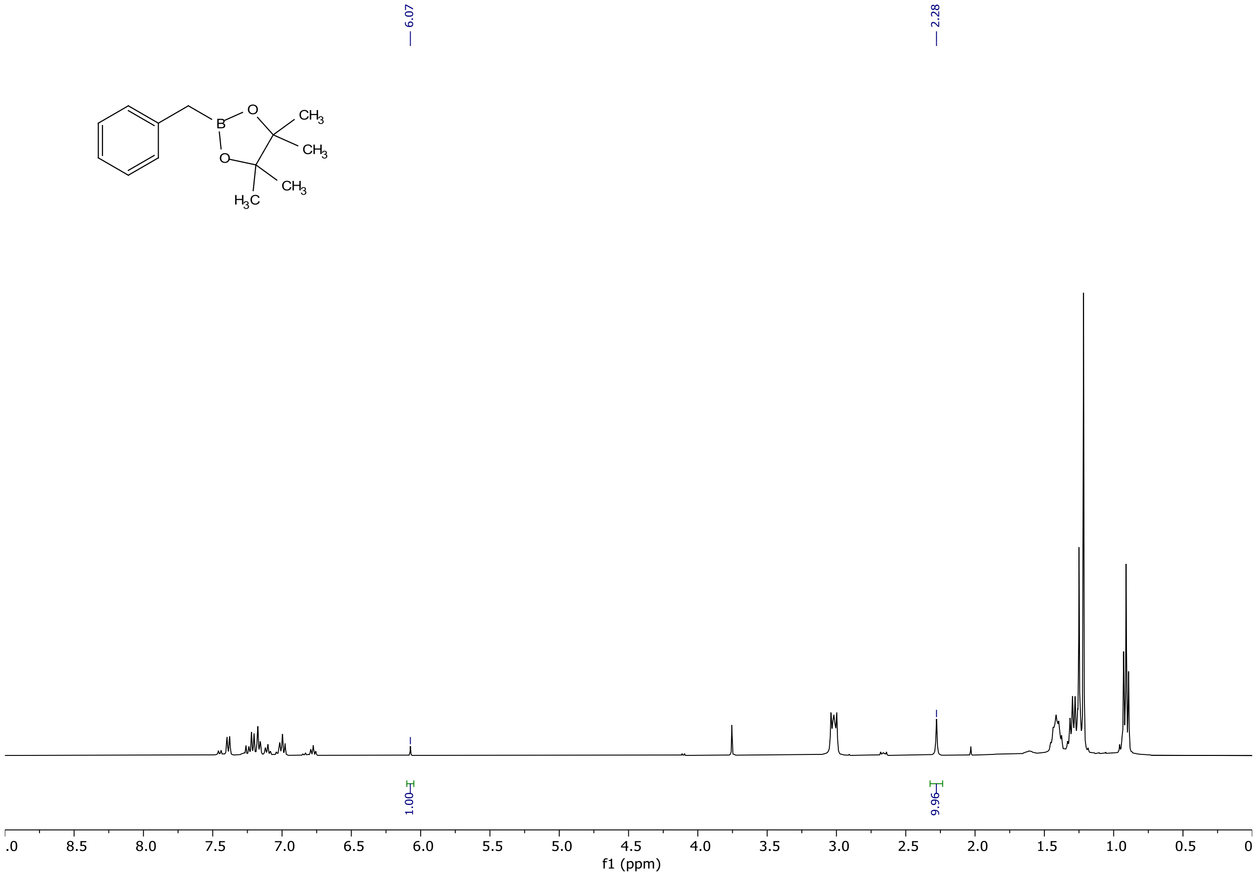

Figure S4: qNMR of compound **2a** against TMB seen at 6.04 ppm, gives a yield of 99% which is comparable to the isolated yield of 95%. Spectra recorded in $CDCl_3$ at 500 MHz.

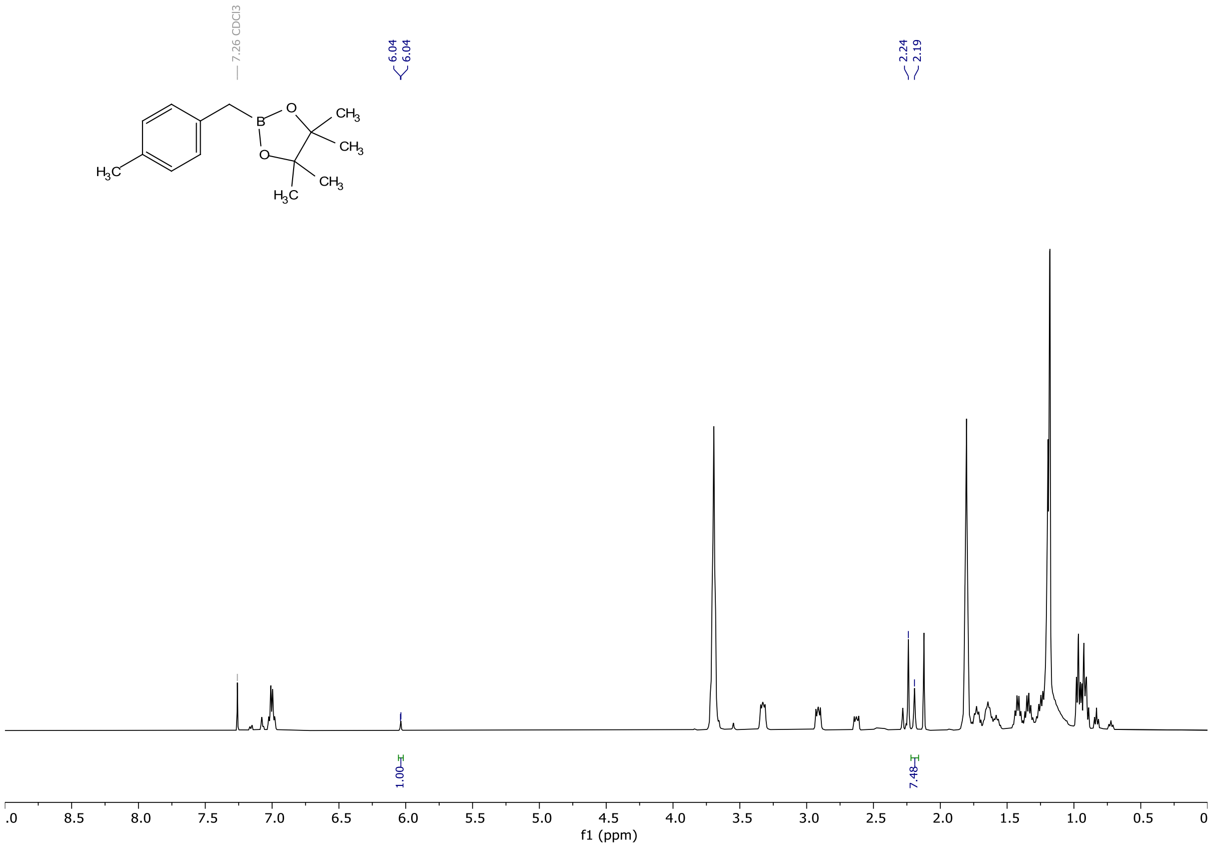

Figure S5: qNMR of compound **2ai** against TMB seen at 6.04 ppm. Spectra recorded in $CDCl_3$ at 500 MHz.

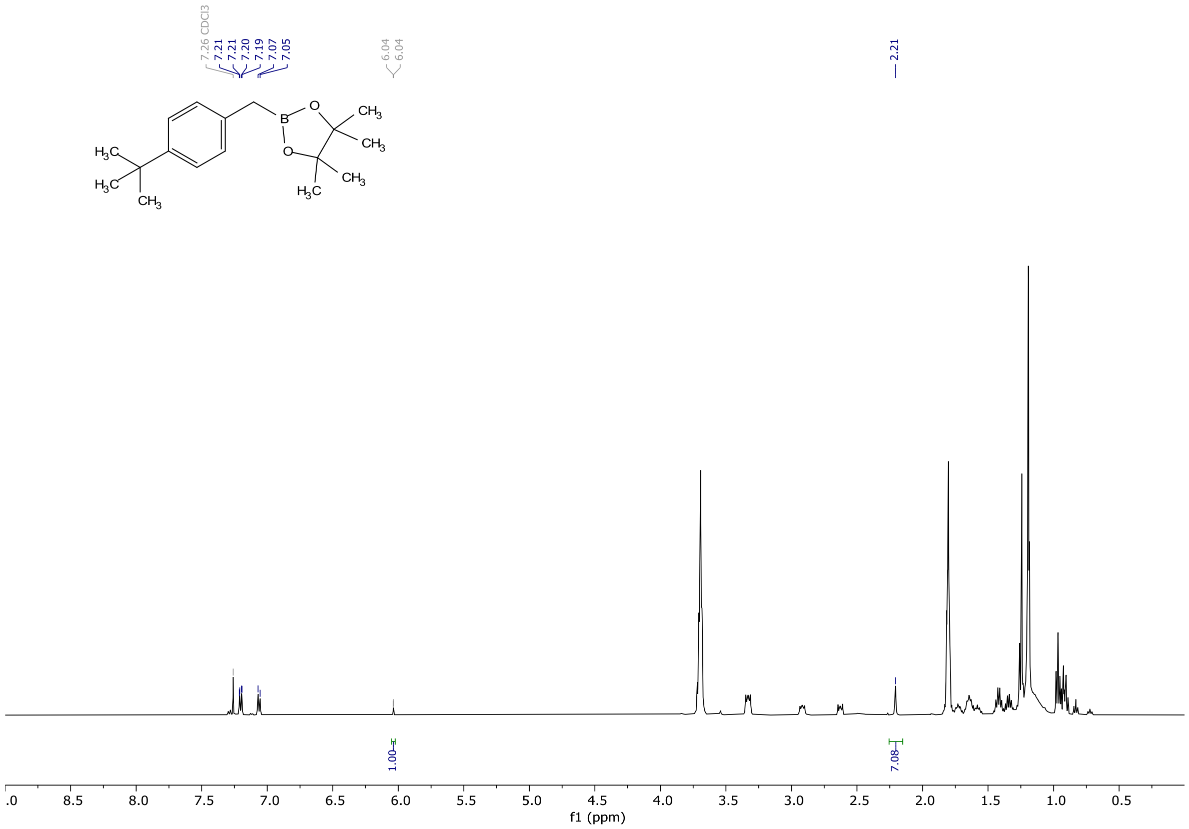

Figure S6: qNMR of compound **2aj** against TMB seen at 6.04 ppm. Spectra recorded in $CDCl_3$ at 500 MHz.

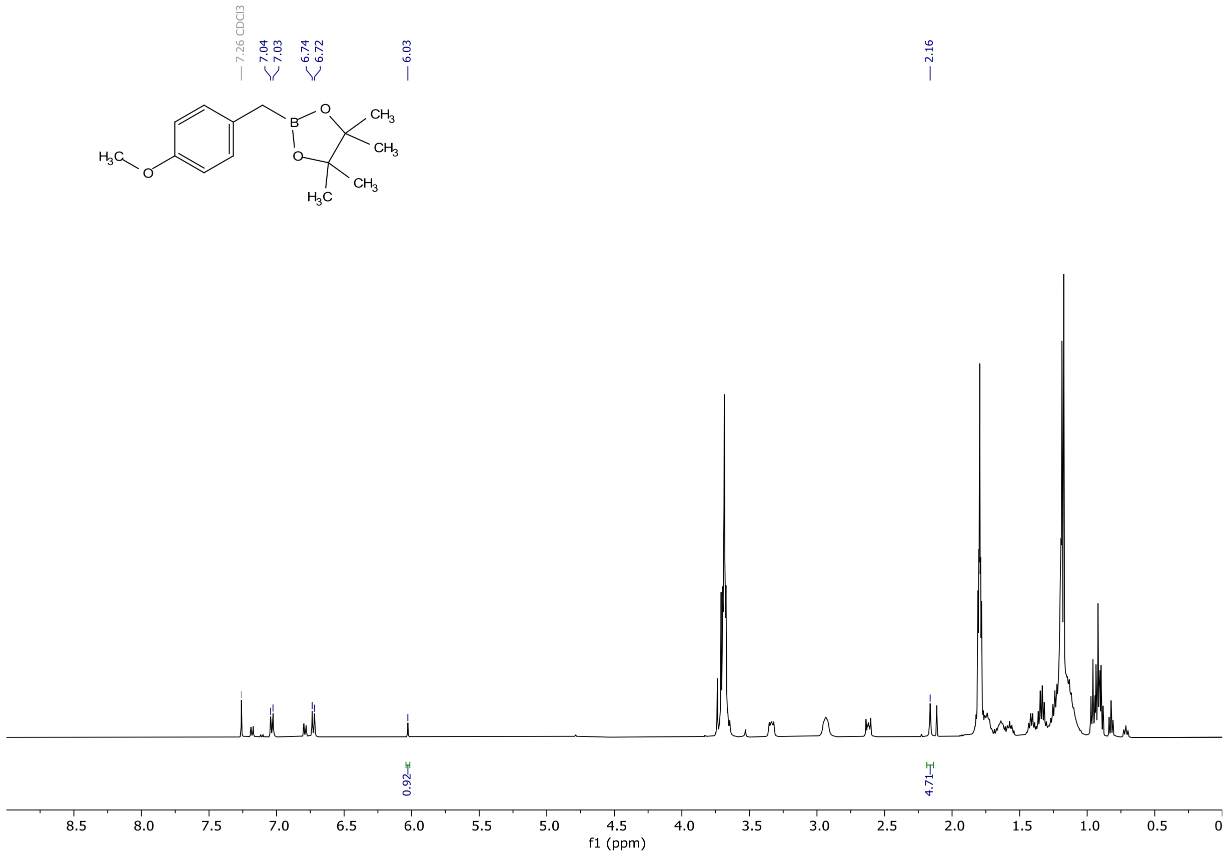

Figure S7: qNMR of compound **2ak** against TMB seen at 6.04 ppm. Spectra recorded in $CDCl_3$ at 500 MHz.

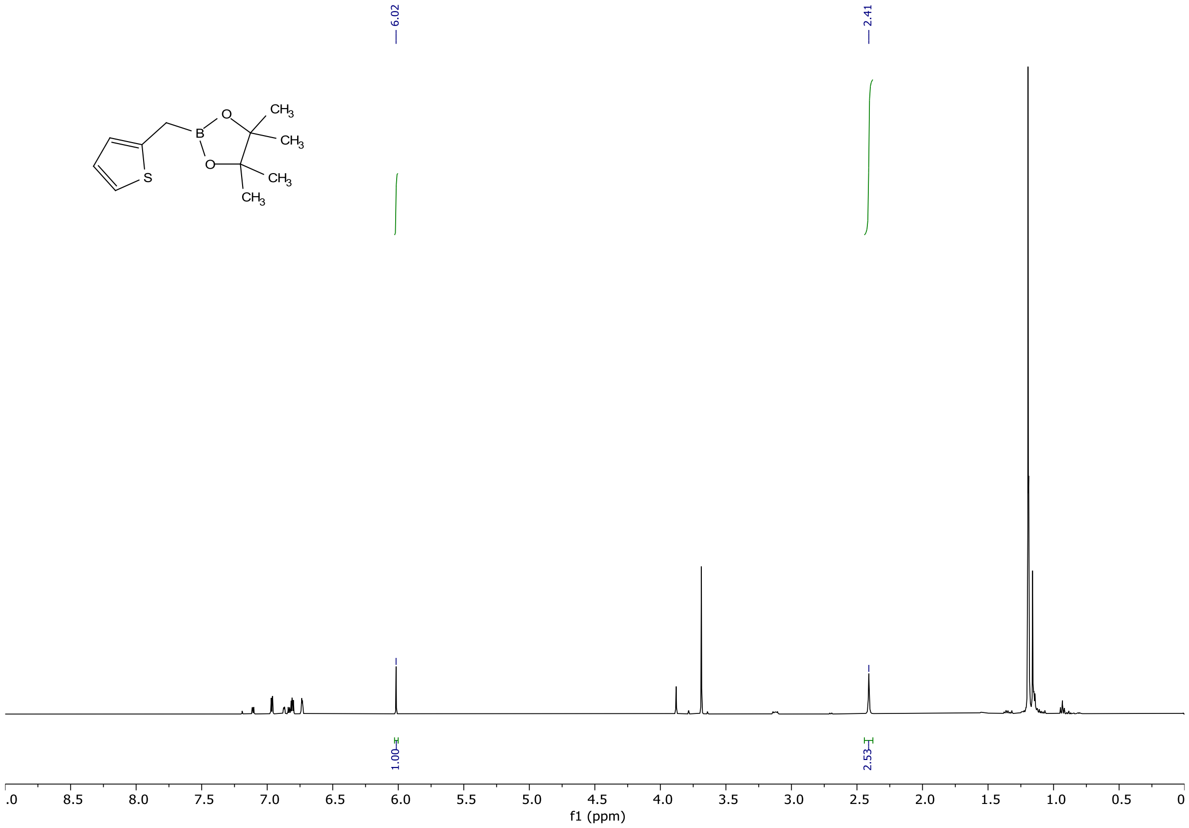

Figure S8: qNMR of compound **2m** against TMB seen at 6.04 ppm. 16.5 mg TMB used as internal standard. Spectra recorded in $CDCl_3$ at 500 MHz.

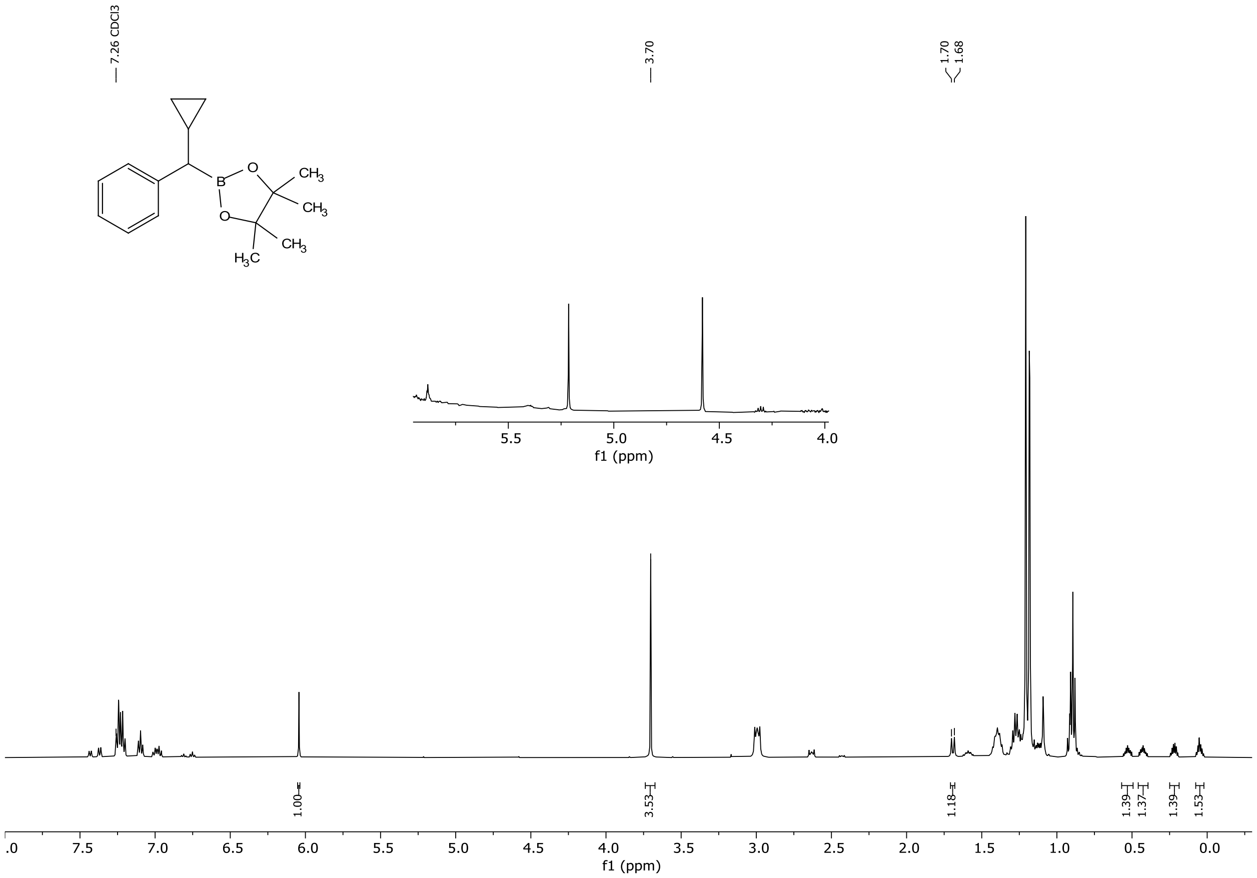

Figure S9: qNMR of compound **2ao** against TMB seen at 6.04 ppm. 22.4 mg TMB used as internal standard. Spectra recorded in $CDCl_3$ at 500 MHz.

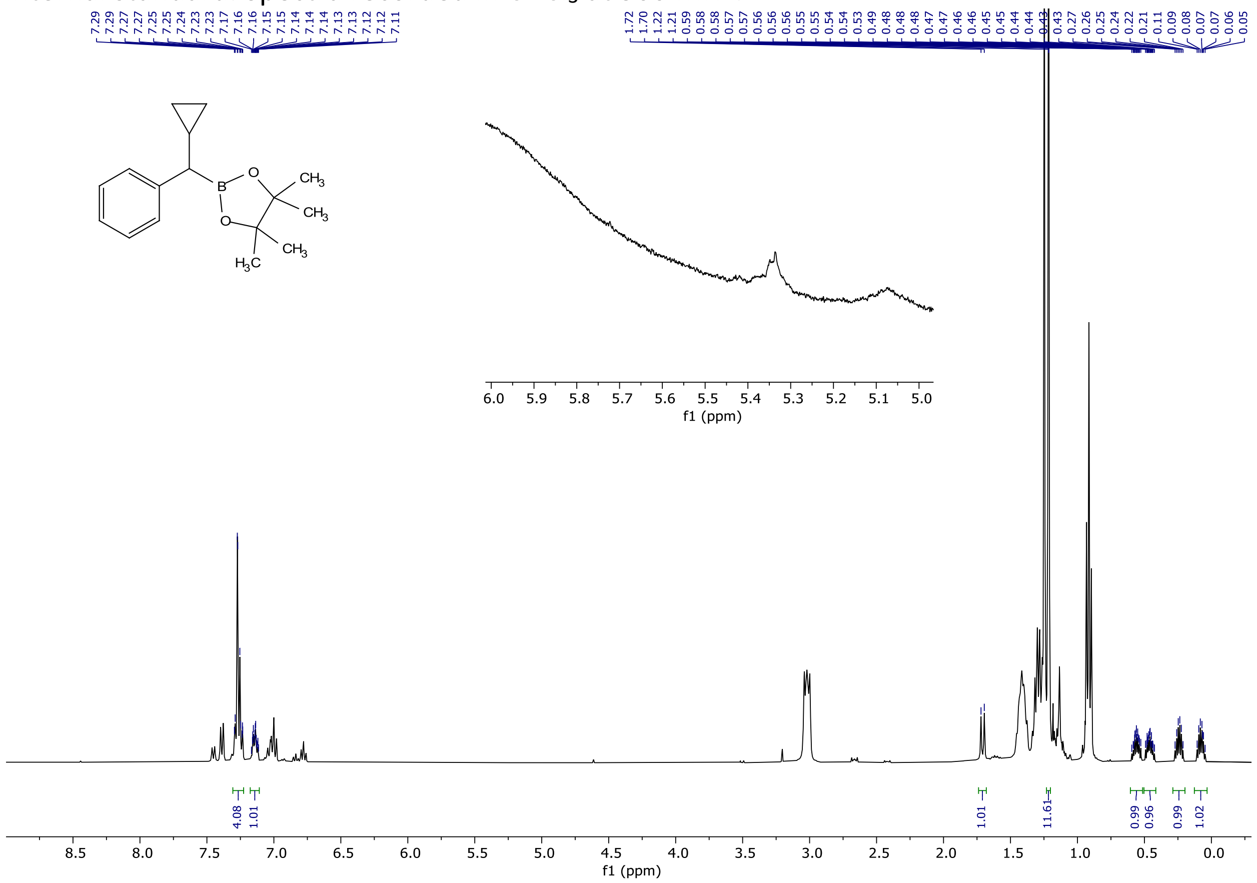

Figure S10: NMR of compound **2ao** showcasing the lack of terminal alkene after electrolysis.

S10 NMR data

S10.1 1a

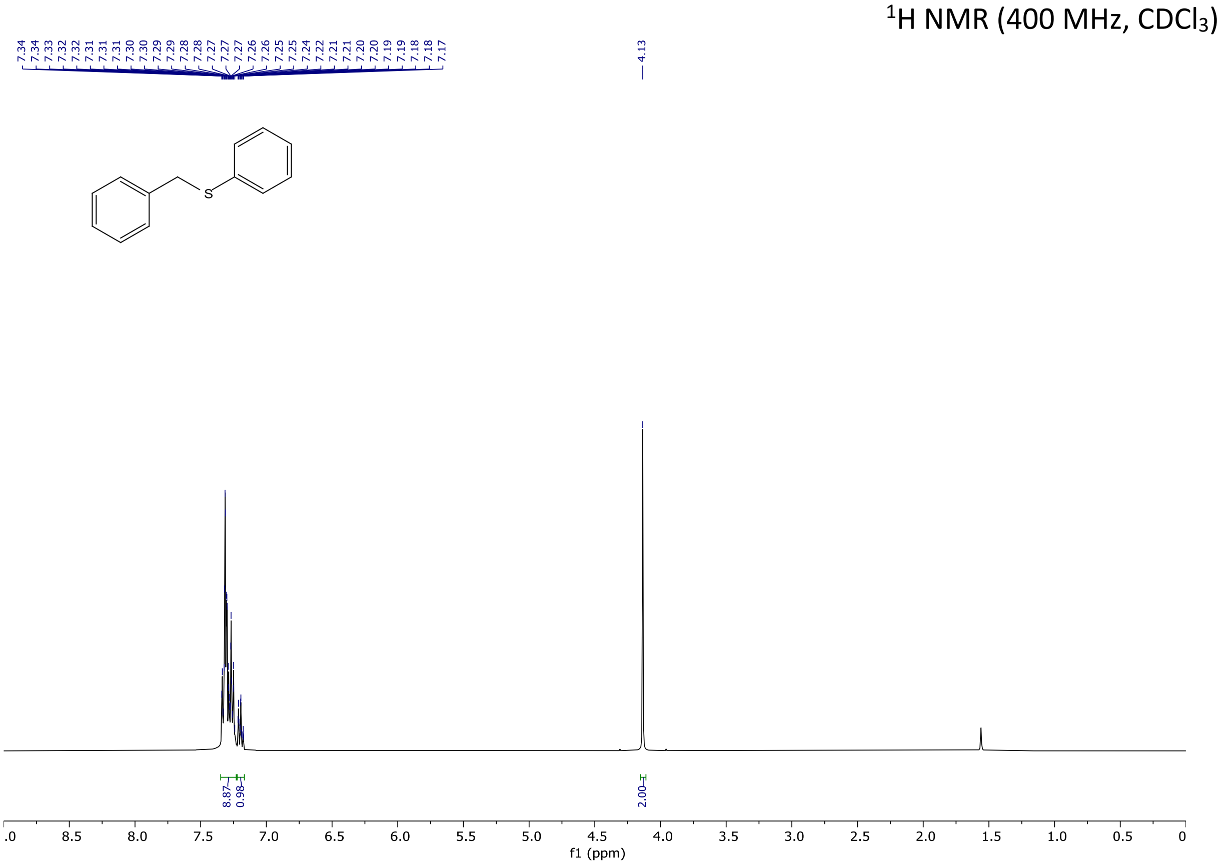

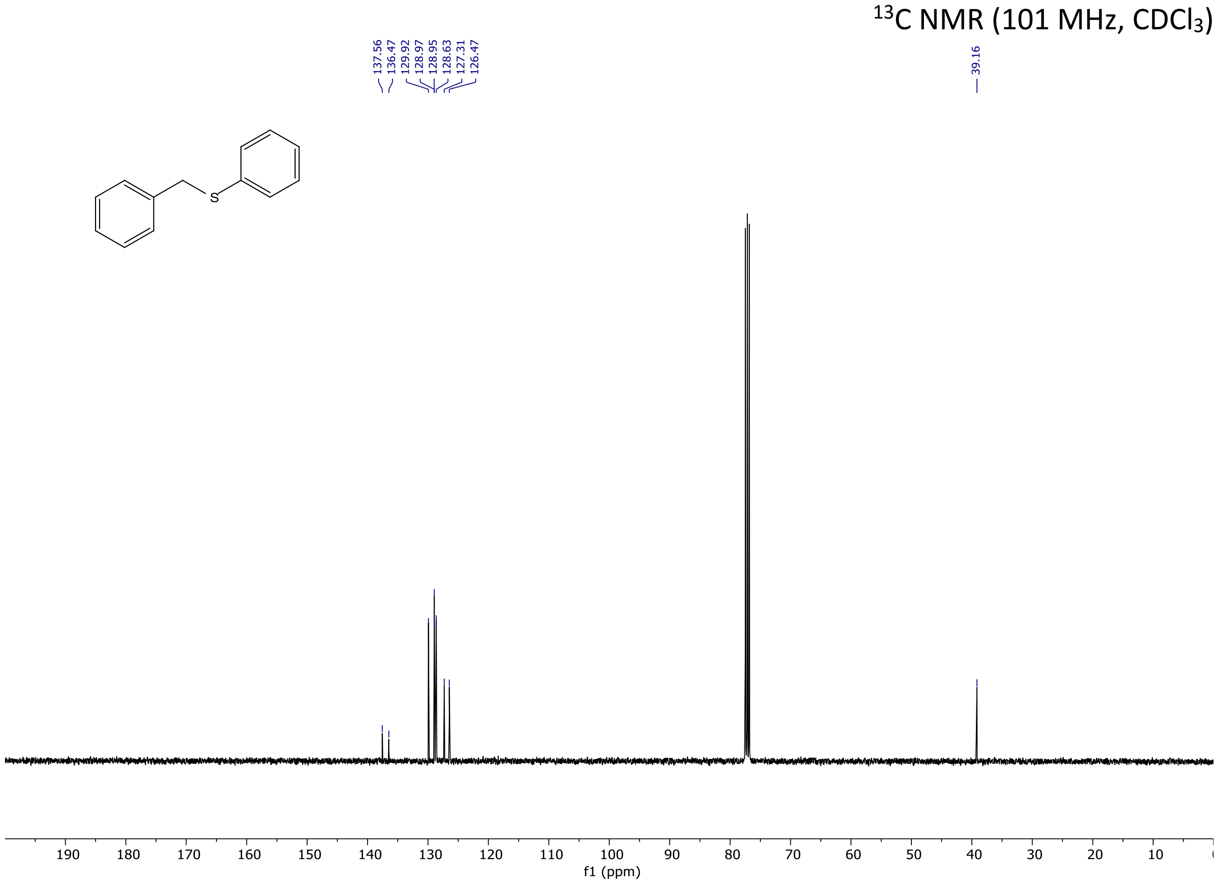

S10.2 1b

^{1}H NMR (400 MHz, $CDCl_3$)

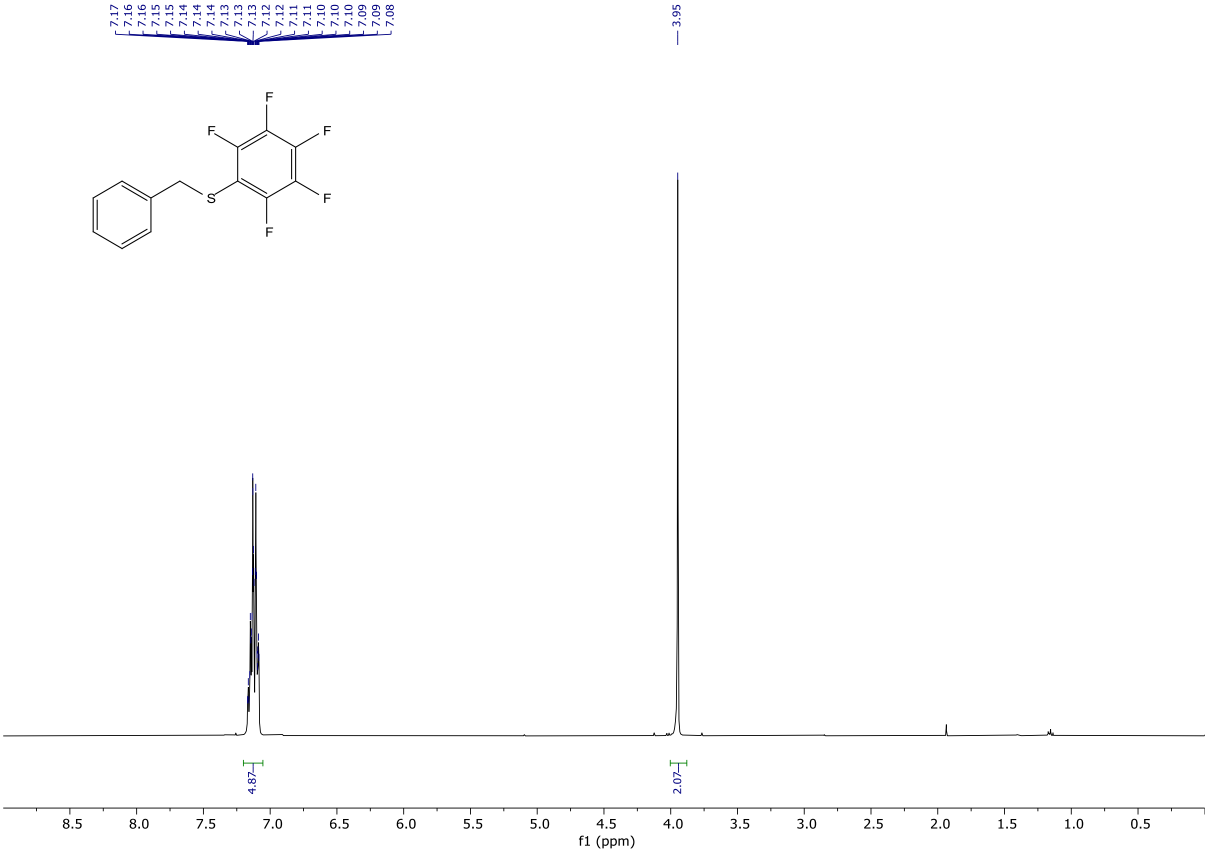

^{13}C NMR (101 MHz, $CDCl_3$)

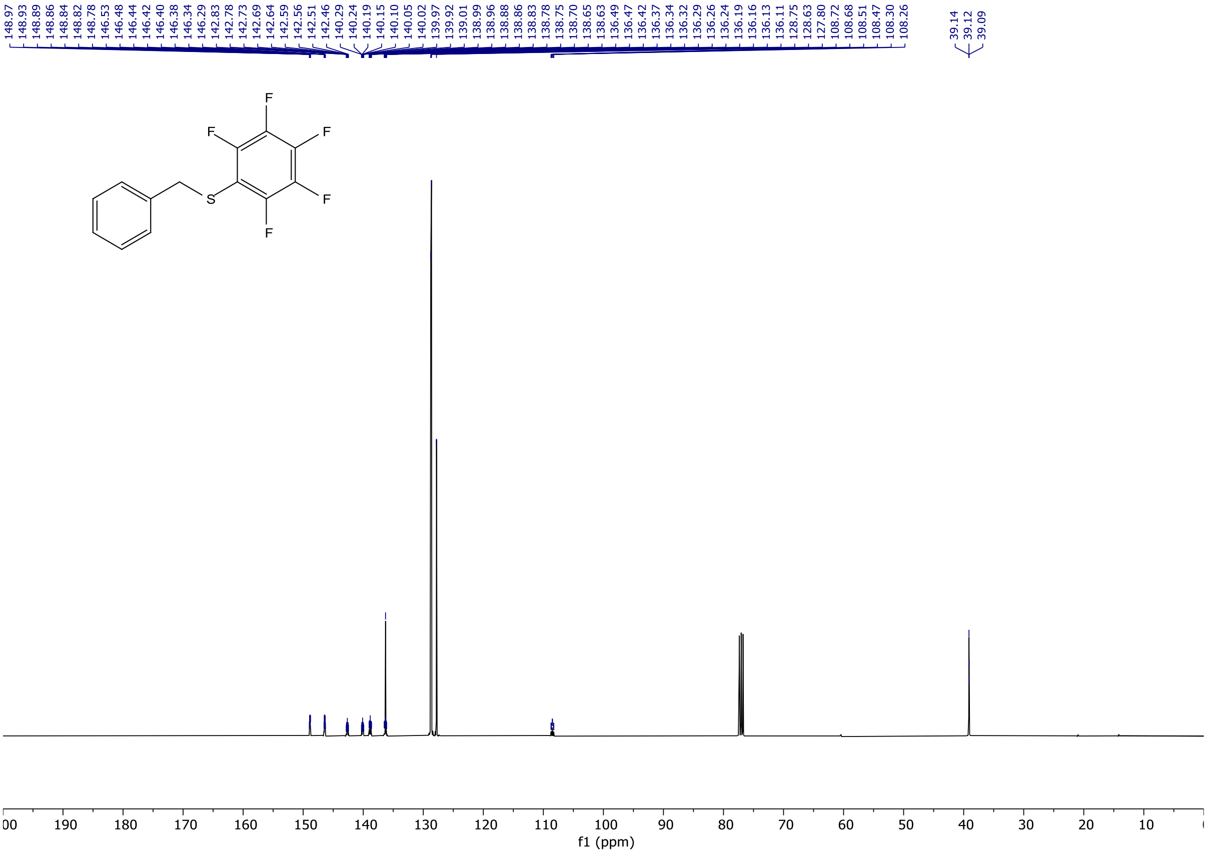

^{19}F NMR (377 MHz, $CDCl_3$)

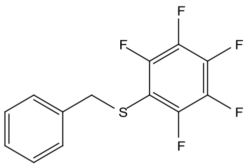

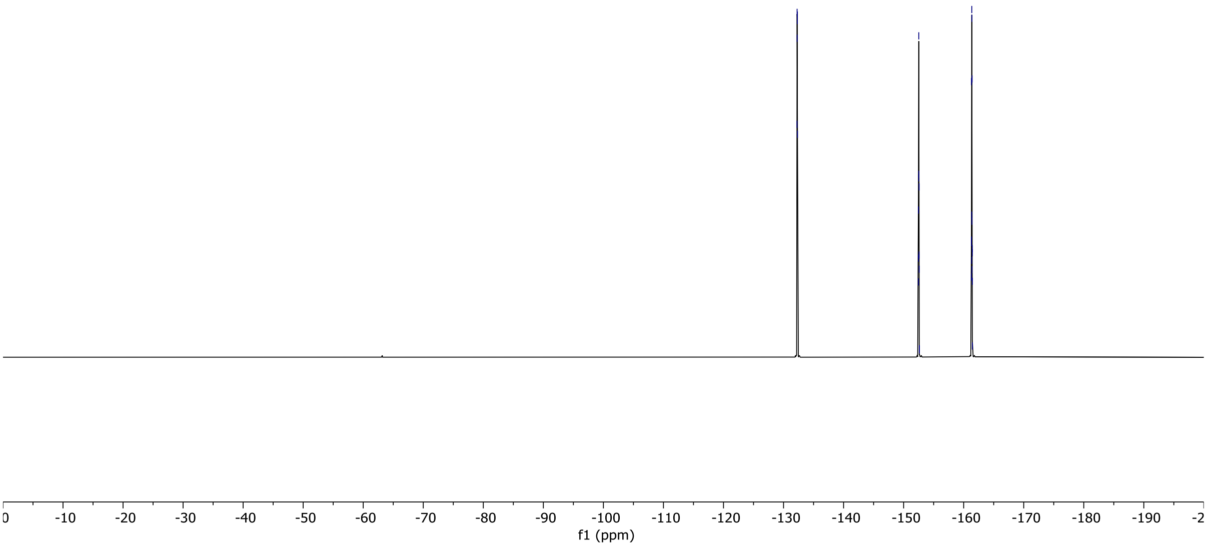

S10.3 1c

^{1}H NMR (500 MHz, $CDCl_3$)

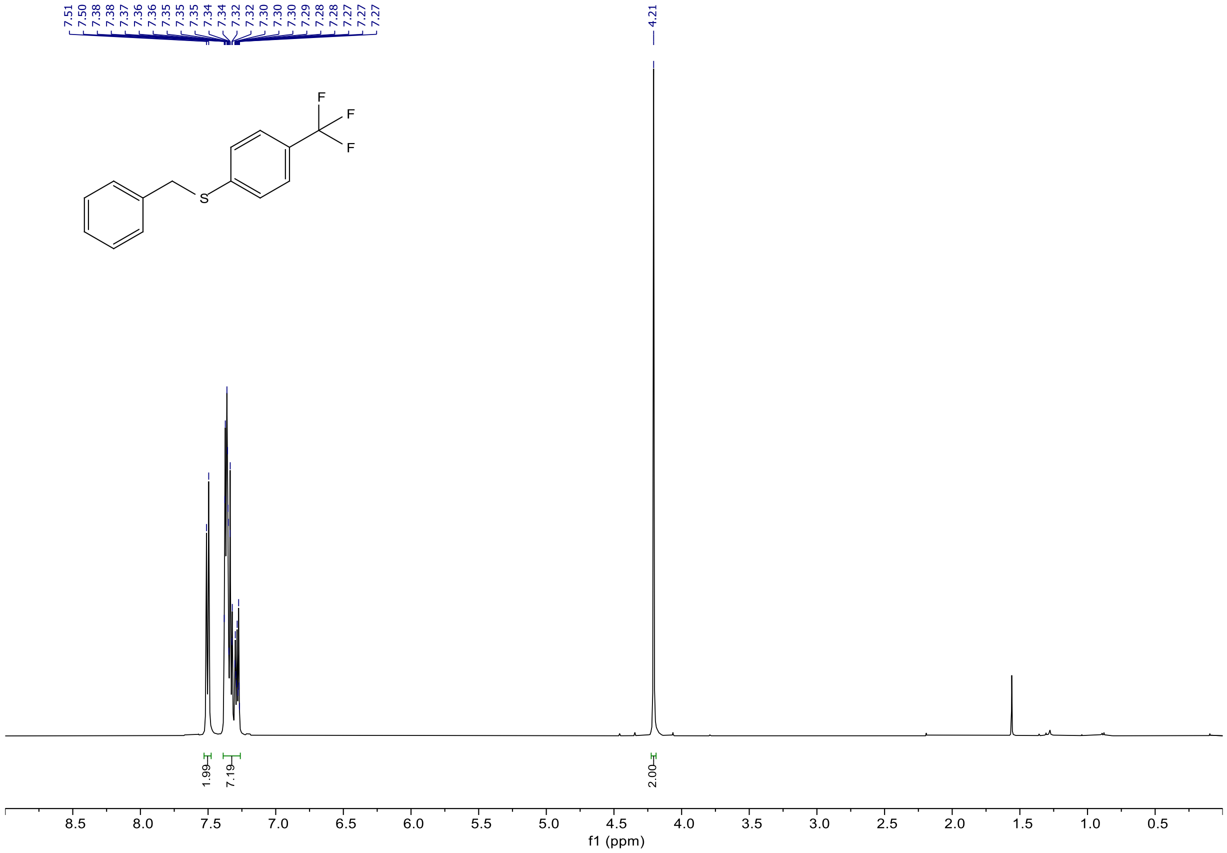

^{19}F NMR (377 MHz, $CDCl_3$)

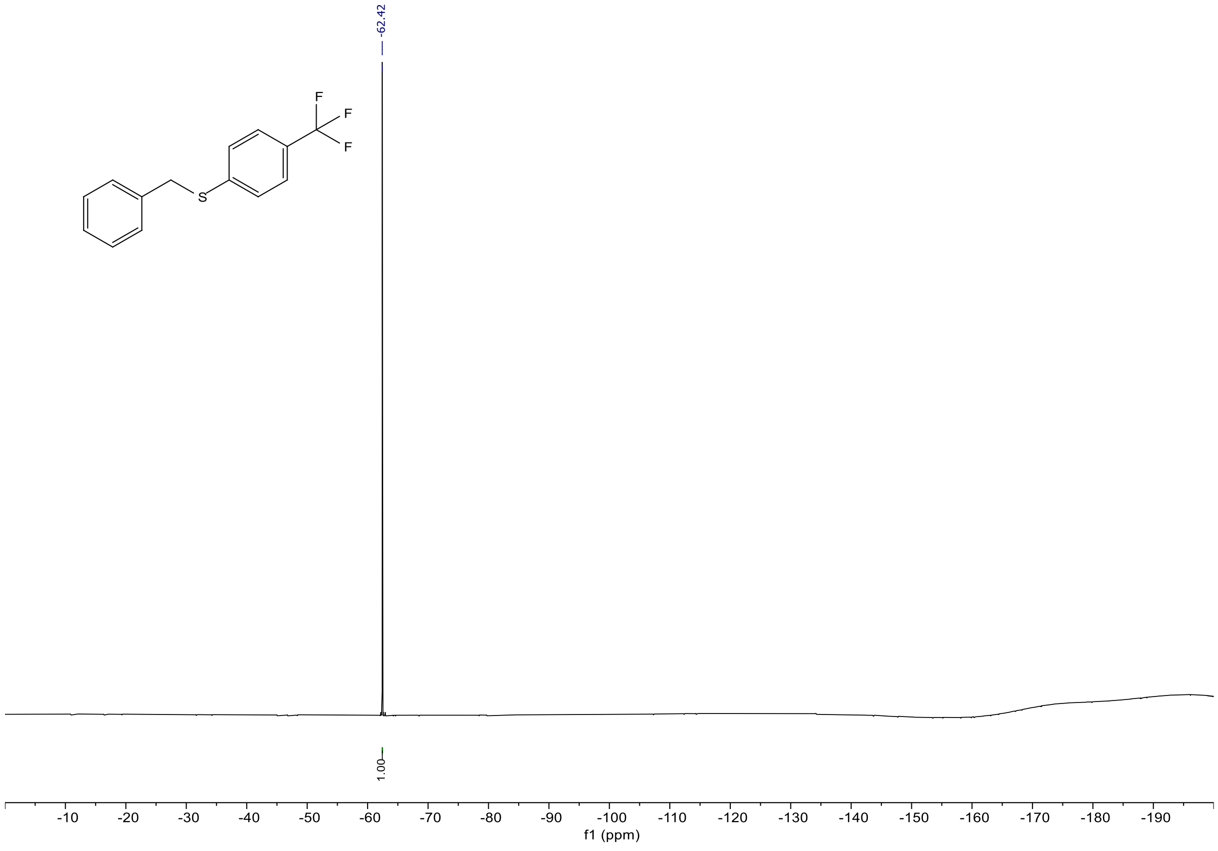

^{13}C NMR (126 MHz, $CDCl_3$)

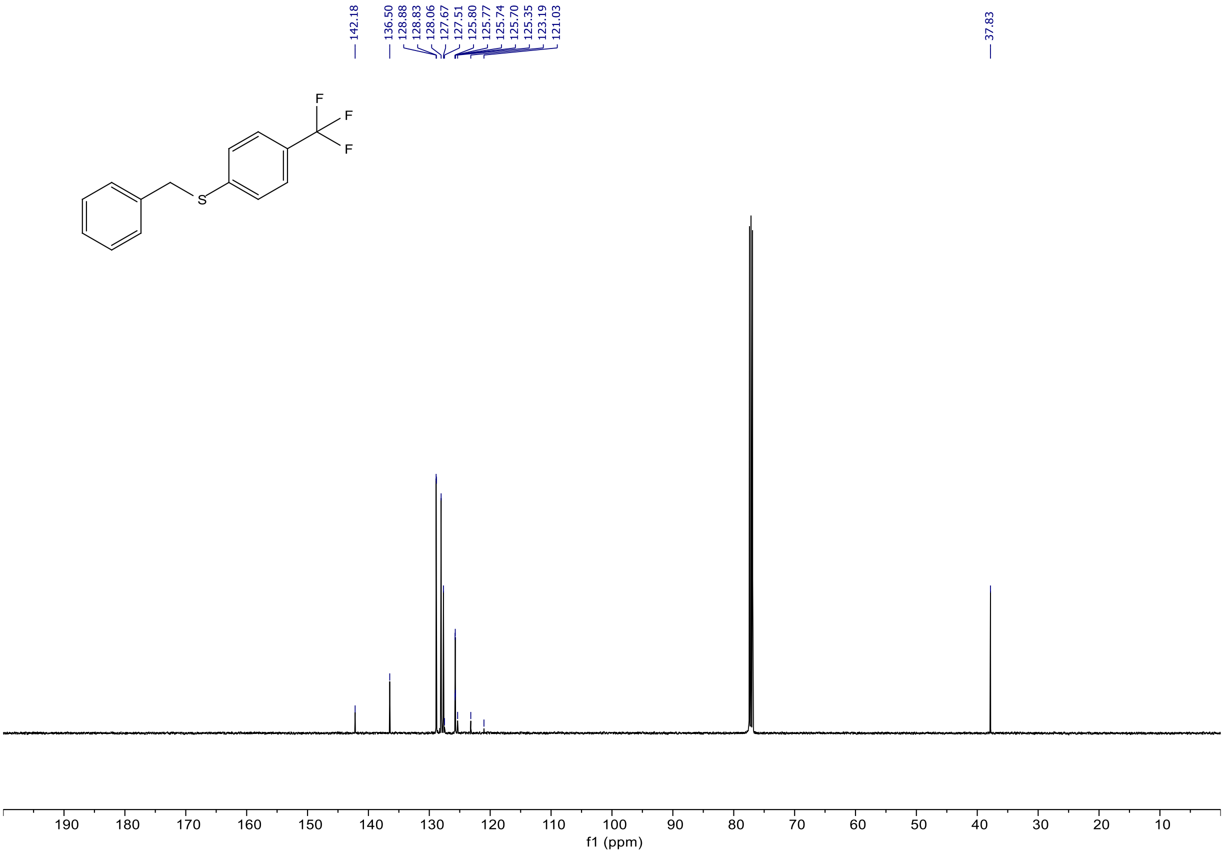

S10.4 1d

^{1}H NMR (400 MHz, $CDCl_3$)

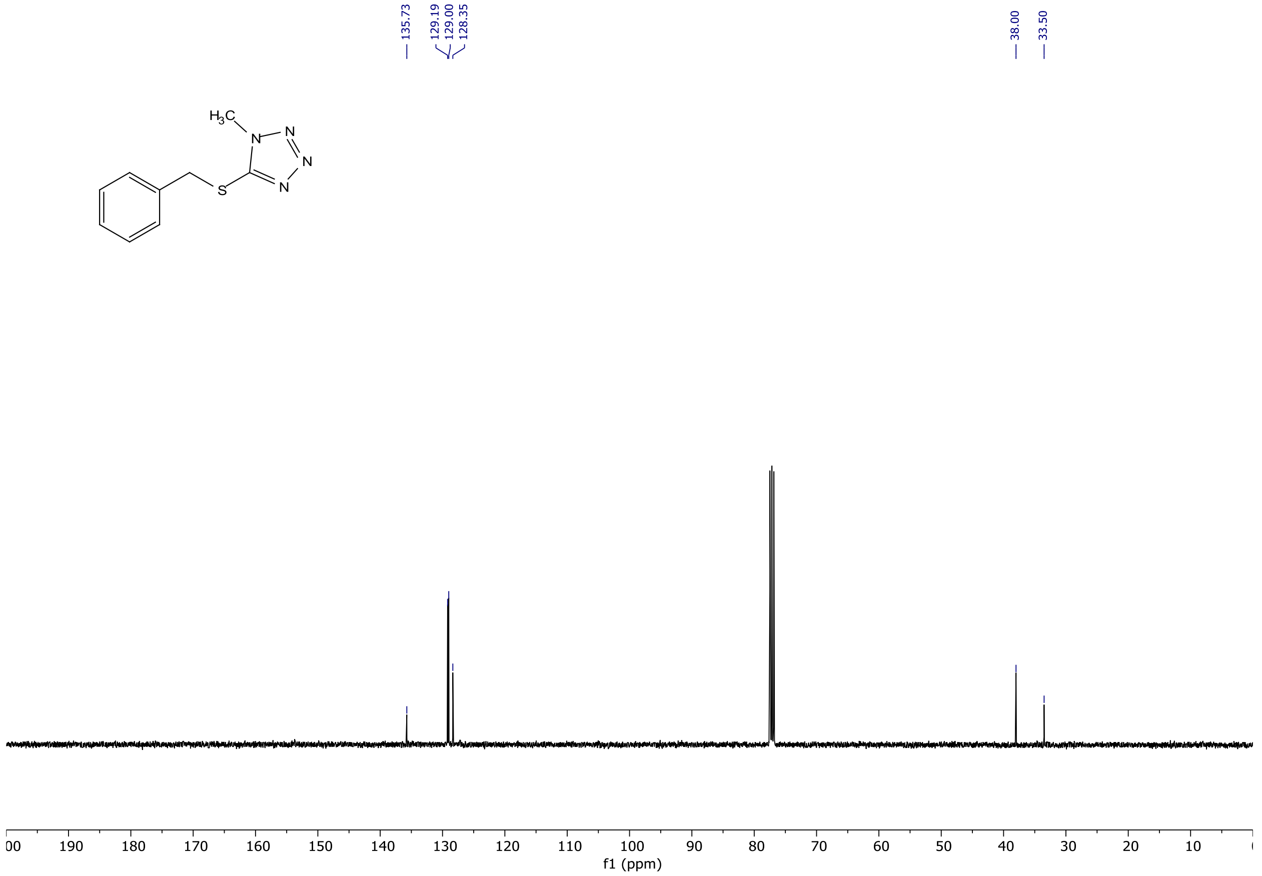

^{13}C NMR (101 MHz, $CDCl_3$)

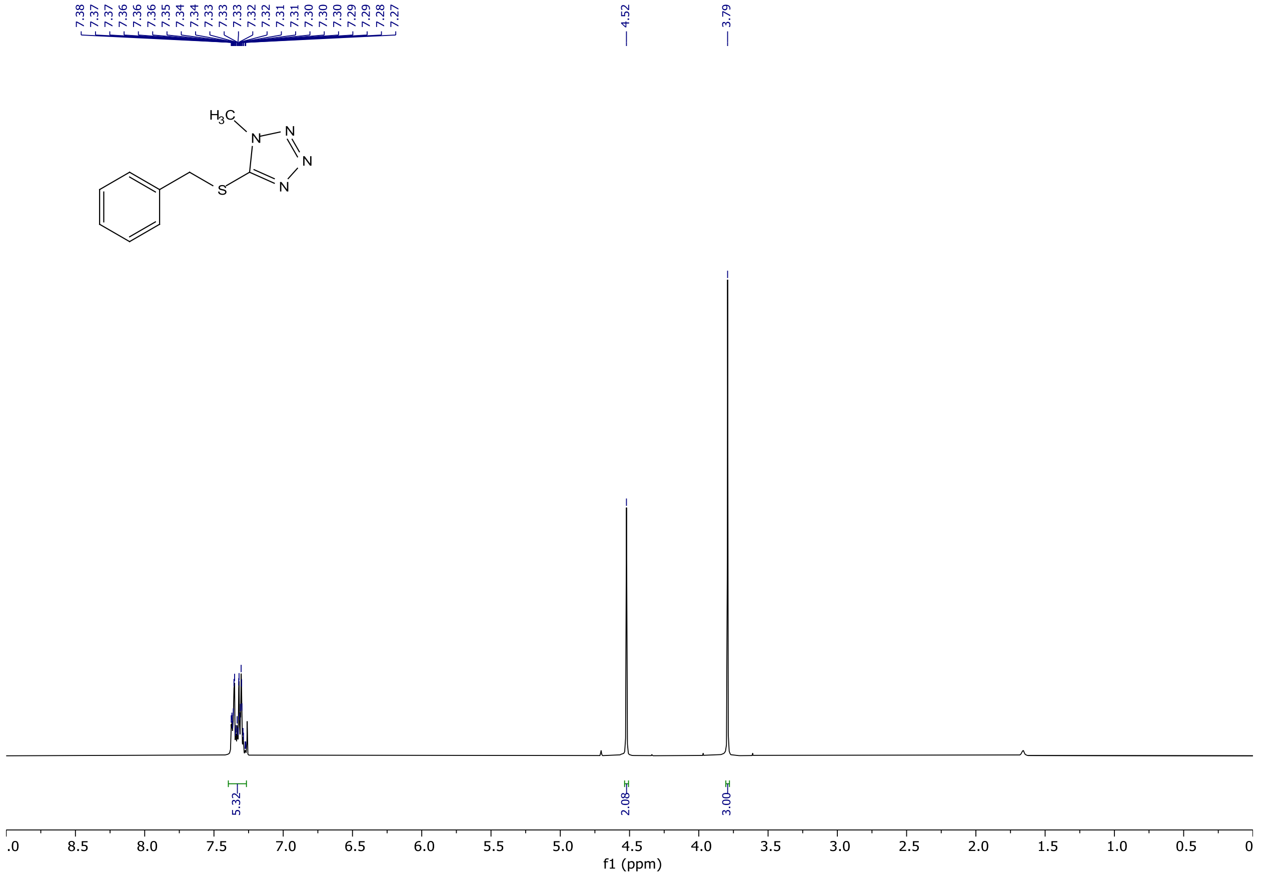

S10.5 1e

^{1}H NMR (400 MHz, $CDCl_3$)

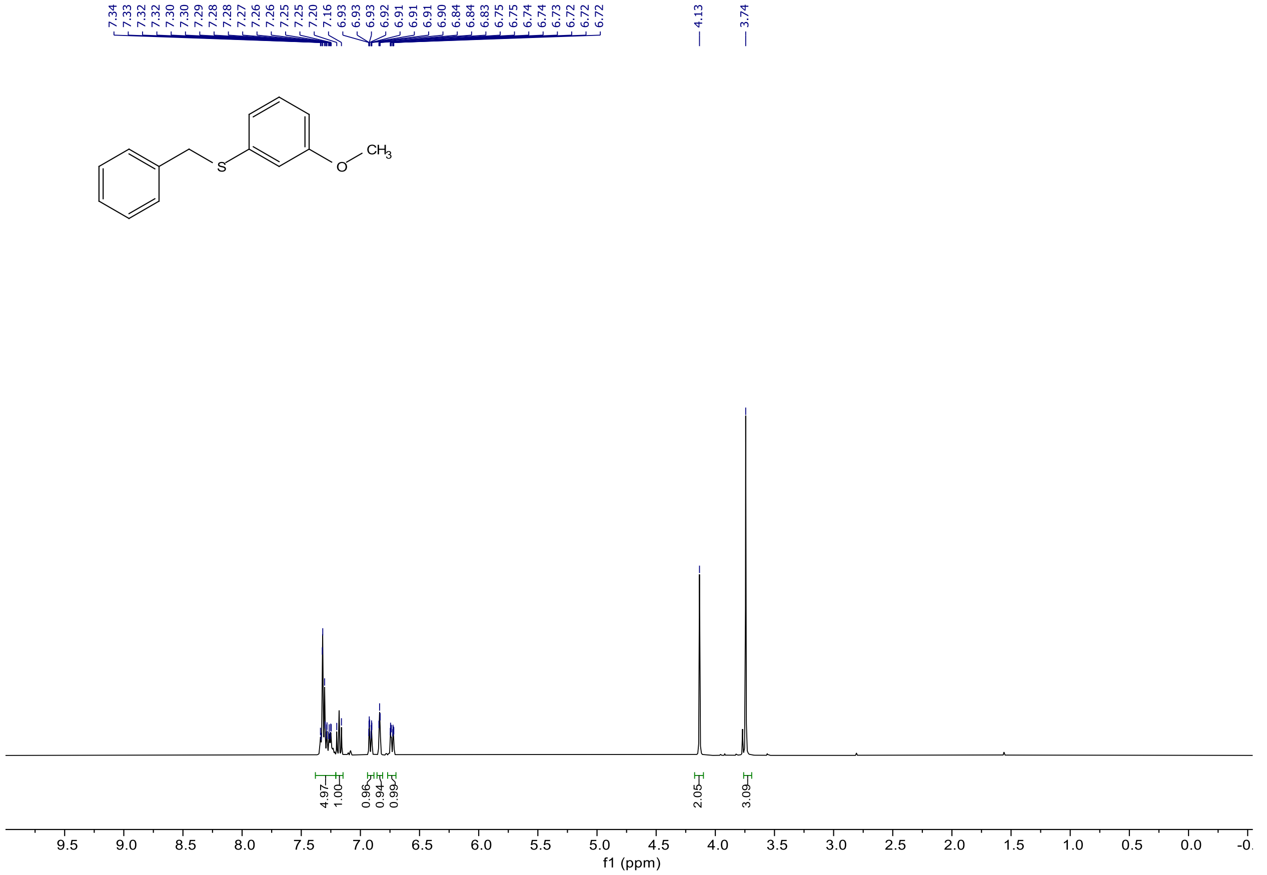

^{13}C NMR (101 MHz, $CDCl_3$)

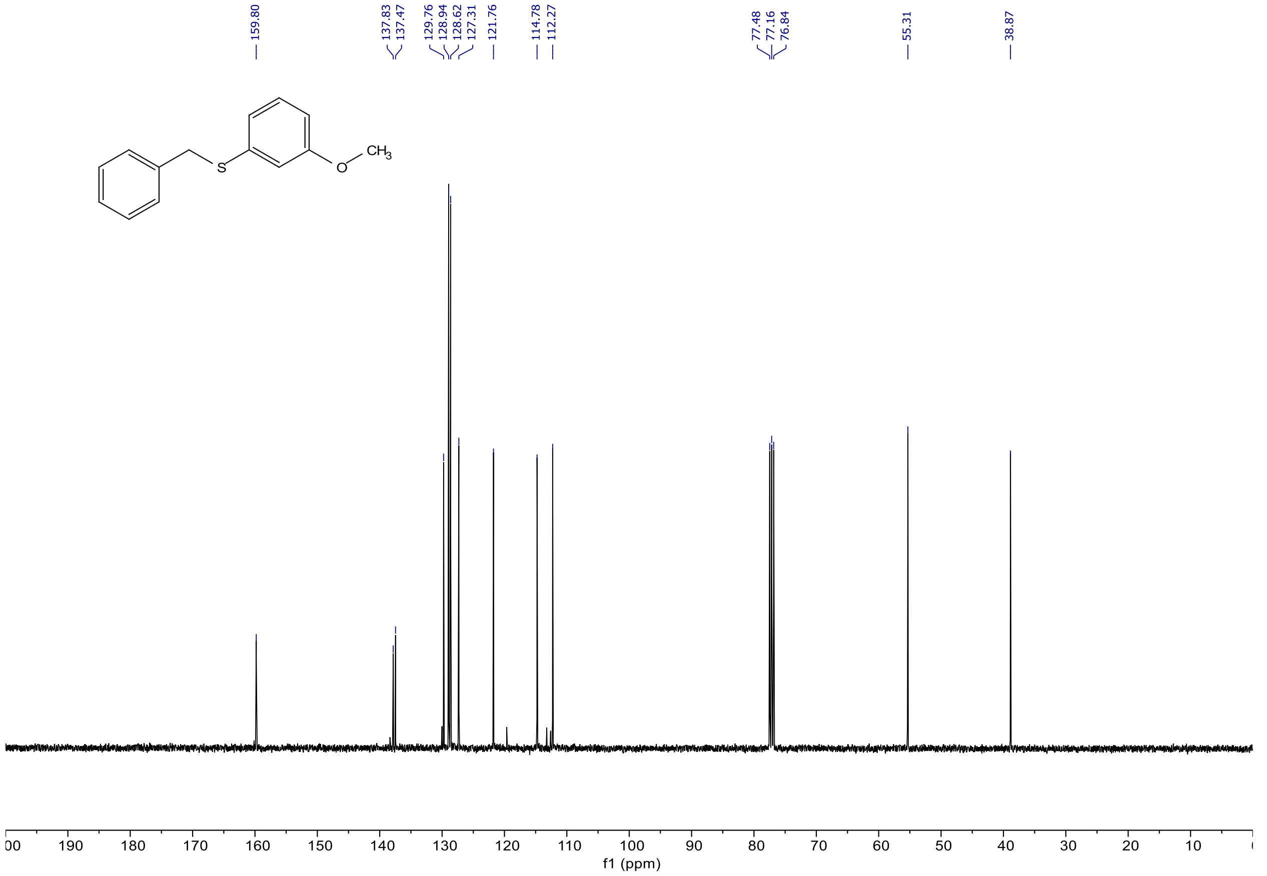

S10.6 1f

^{1}H NMR (400 MHz, $CDCl_3$)

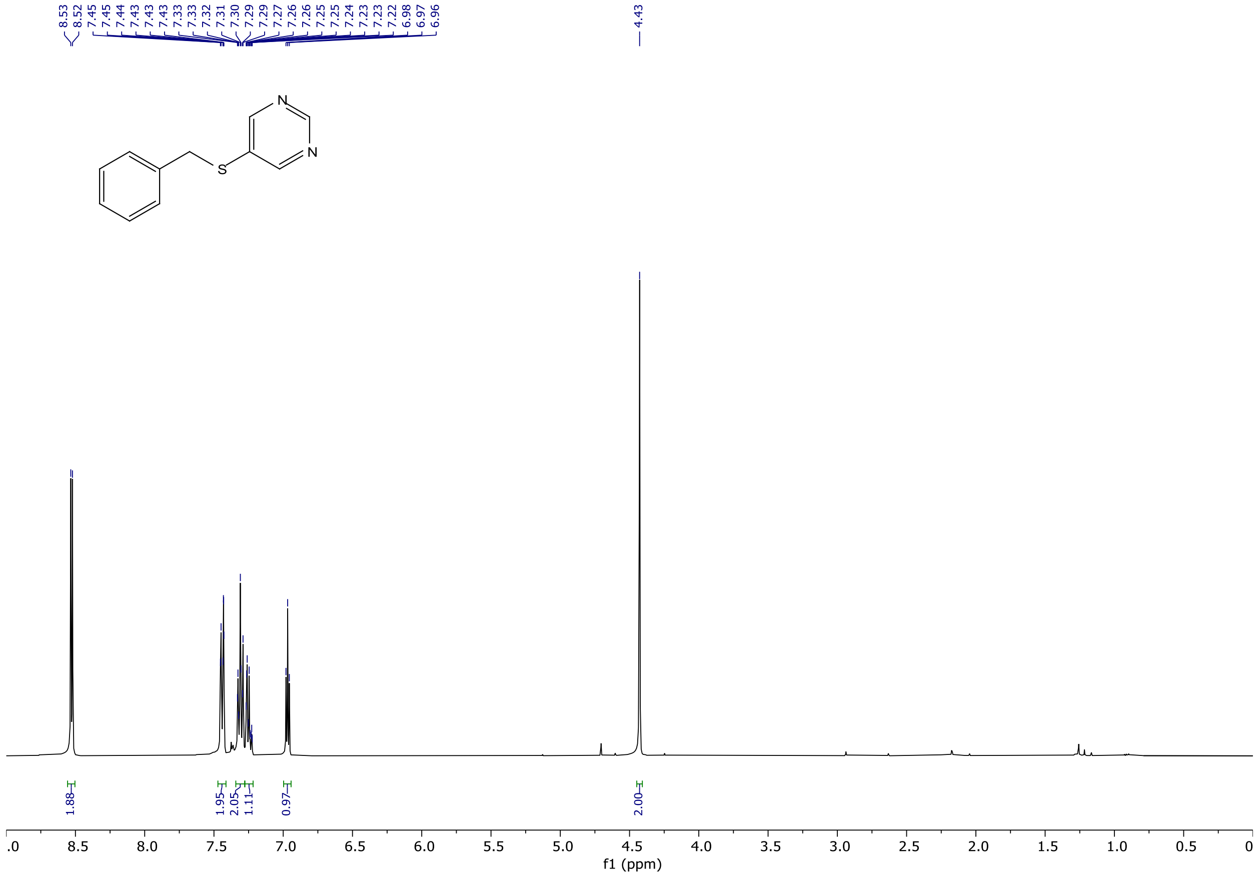

^{13}C NMR (101 MHz, $CDCl_3$)

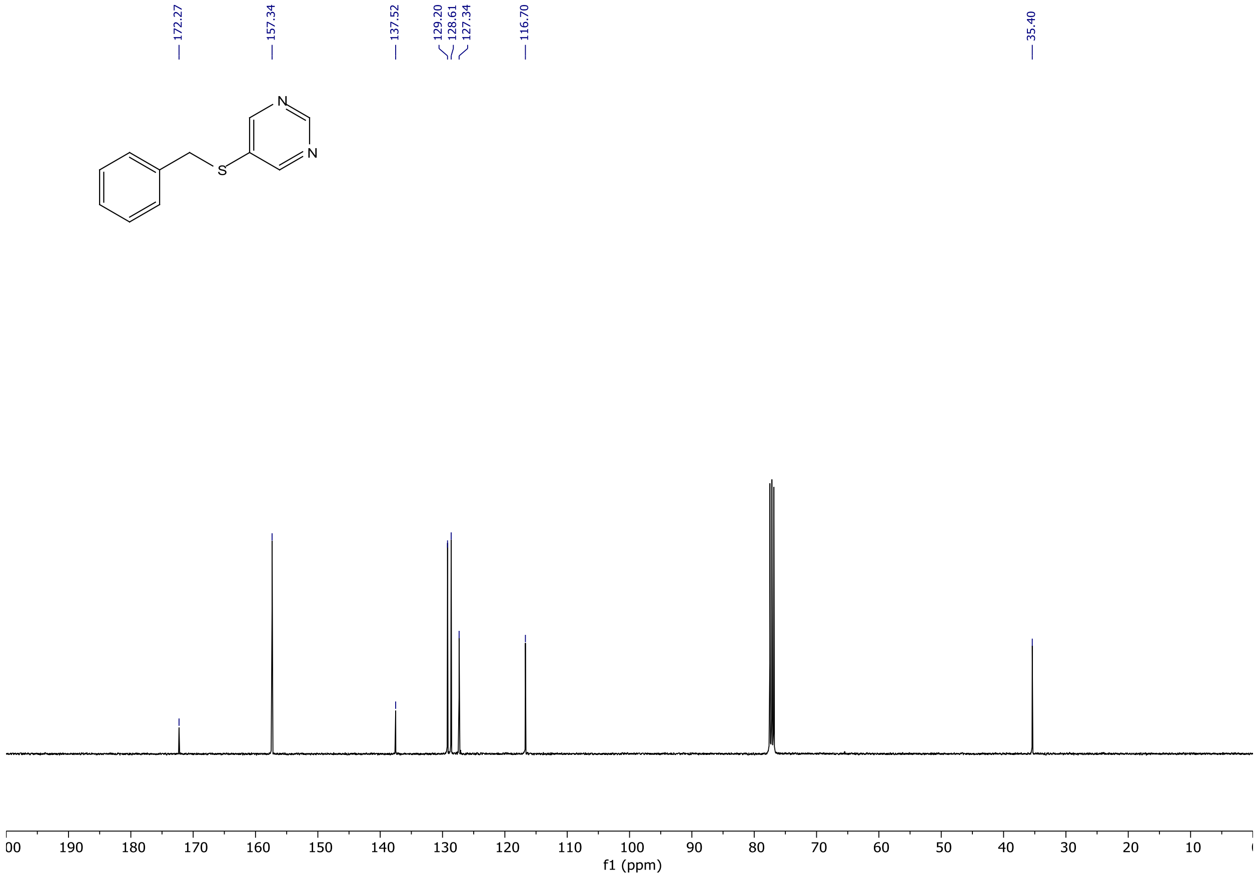

S10.7 1g

^1H NMR (400 MHz, $CDCl_3$)

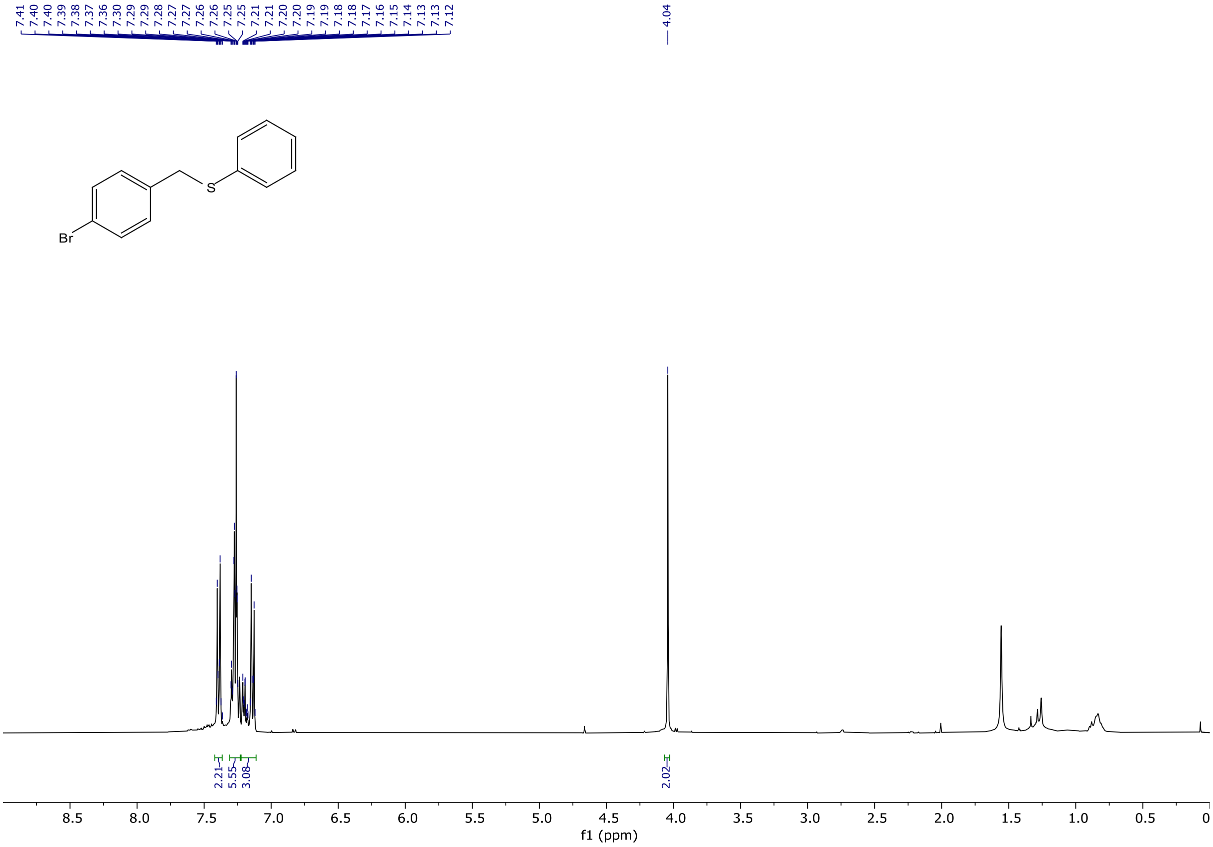

^{13}C NMR (101 MHz, $CDCl_3$)

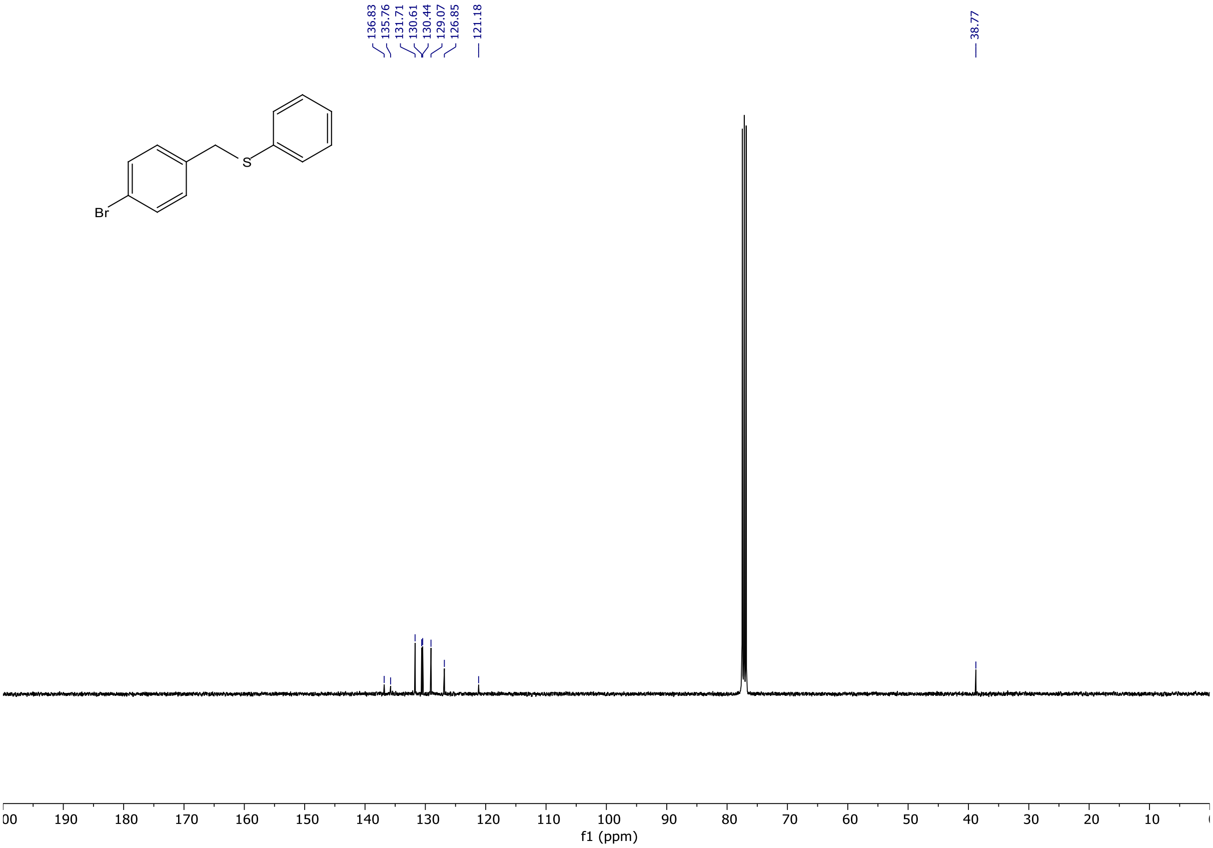

S10.9 1h

^{1}H NMR (400 MHz, $CDCl_3$)

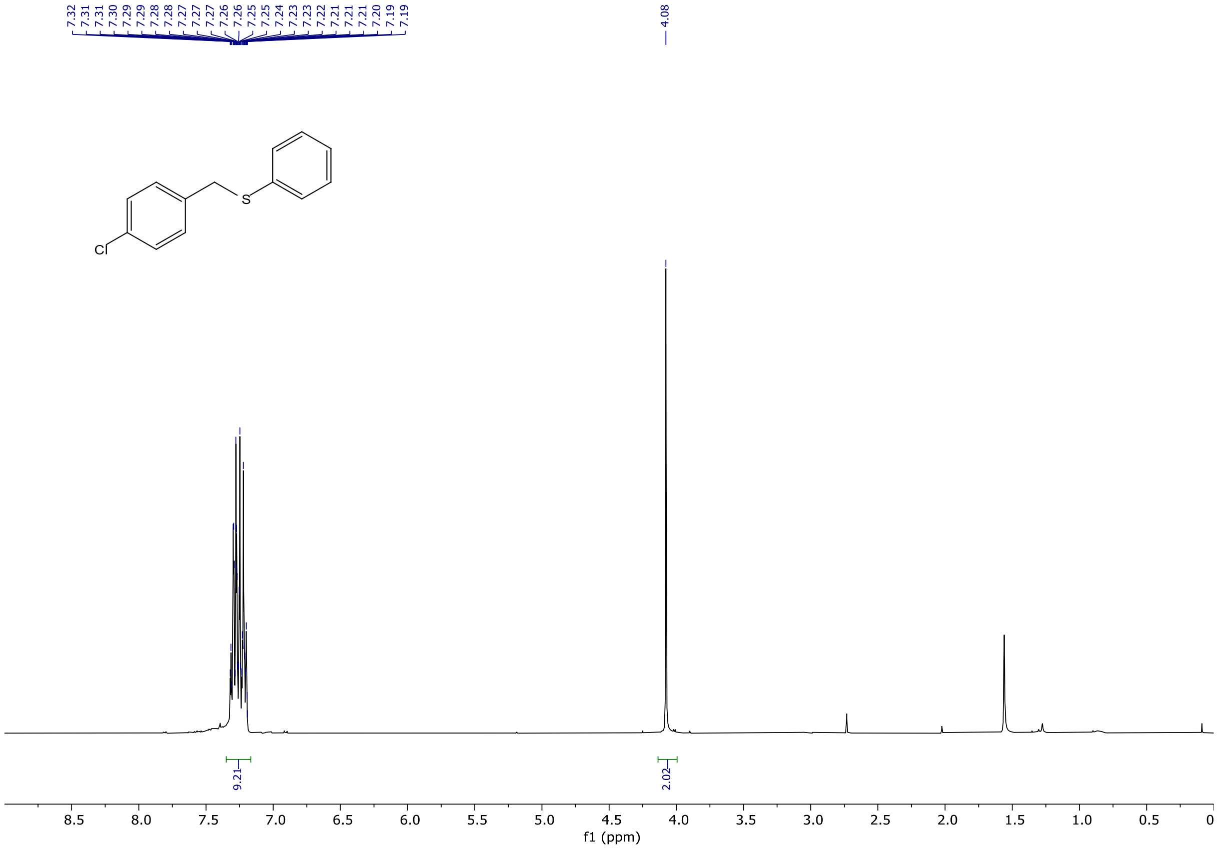

^{13}C NMR (101 MHz, $CDCl_3$)

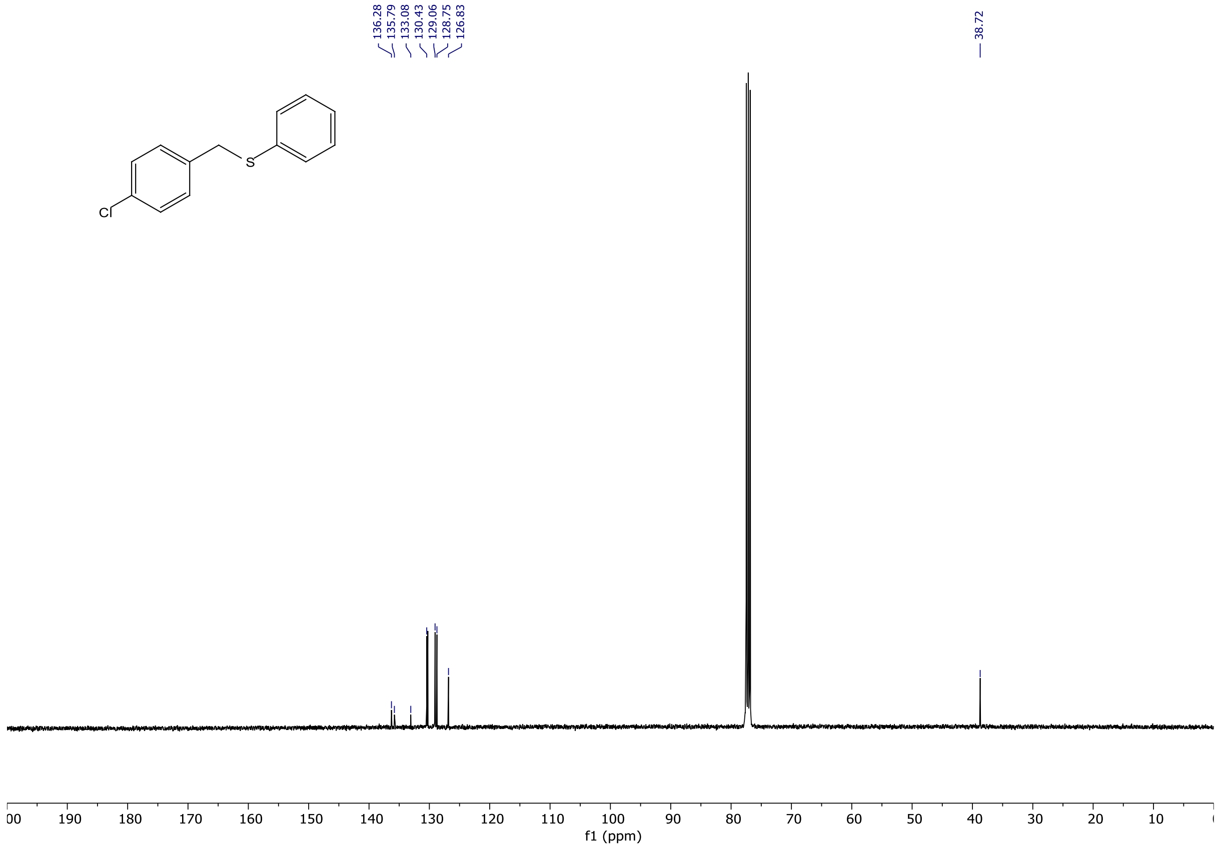

S10.10 1i

^{1}H NMR (500 MHz, $CDCl_3$)

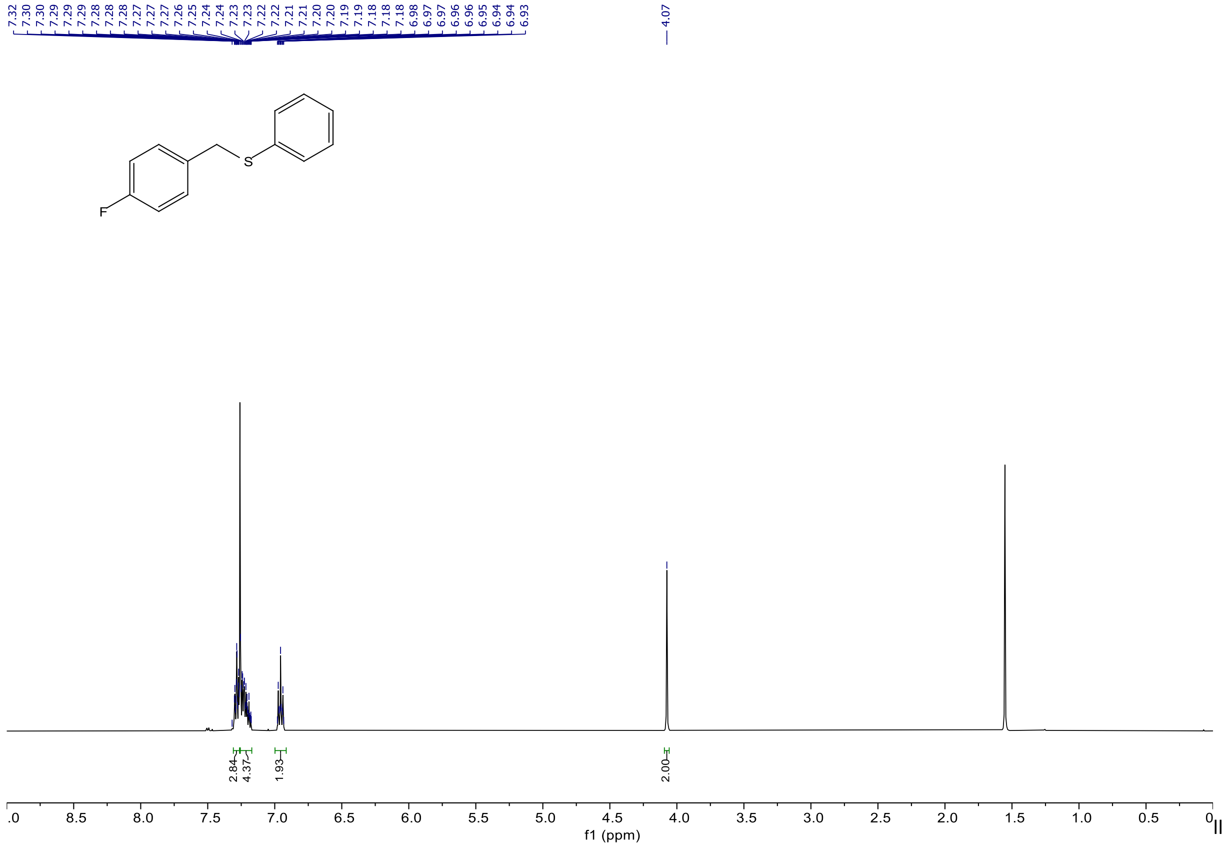

^{13}C NMR (126 MHz, $CDCl_3$)

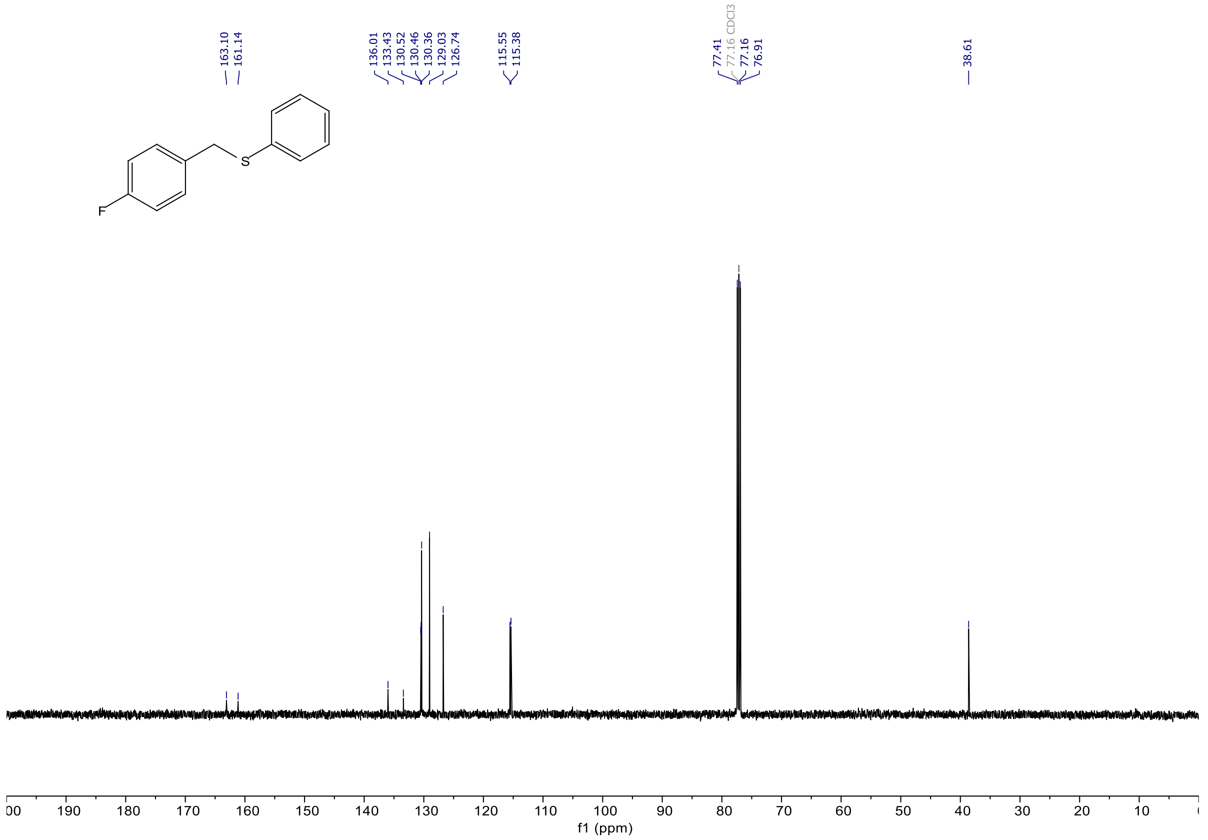

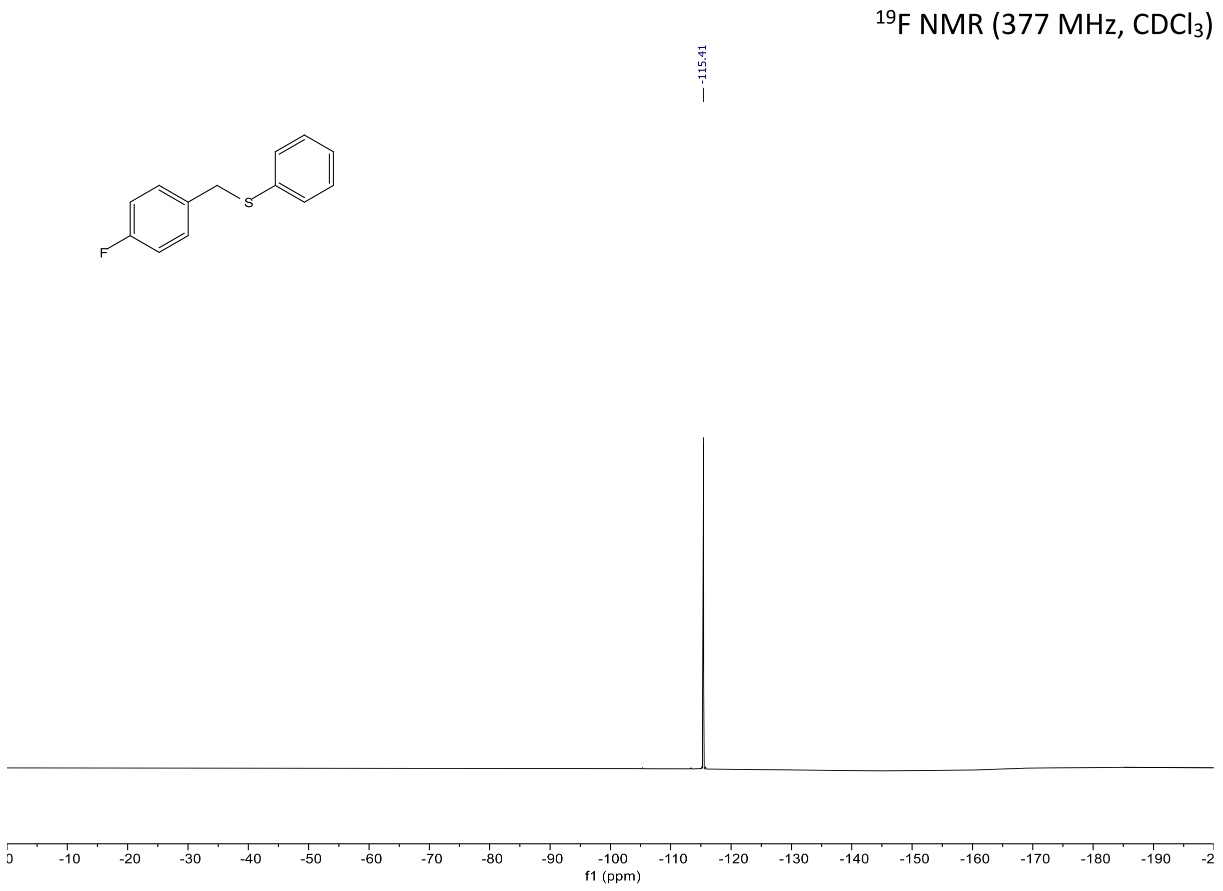

^{19}F NMR (377 MHz, $CDCl_3$)
-115.41
F
S
f1 (ppm)

S10.11 1j

^{1}H NMR (500 MHz, $CDCl_3$)

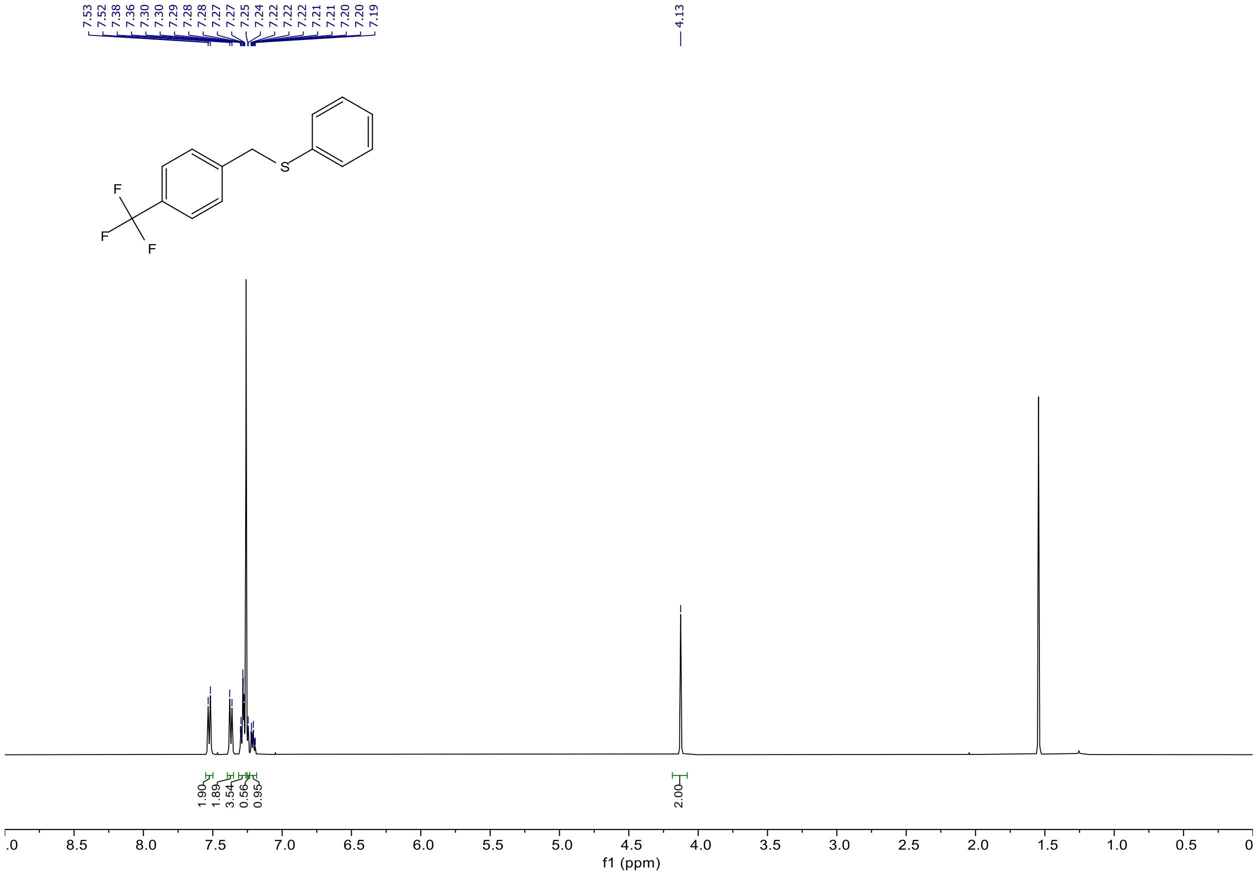

^{13}C NMR (126 MHz, $CDCl_3$)

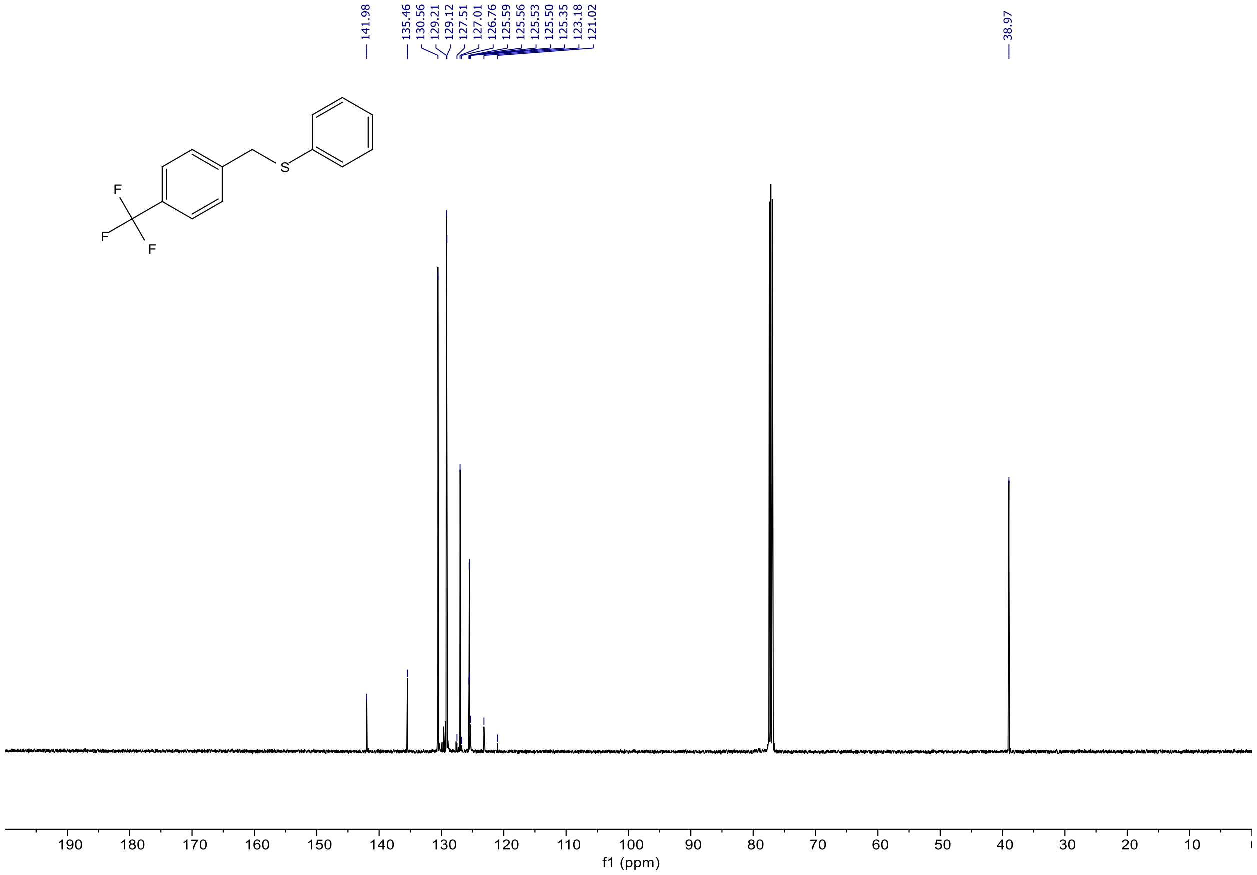

¹⁹F NMR (377 MHz, $CDCl_3$)

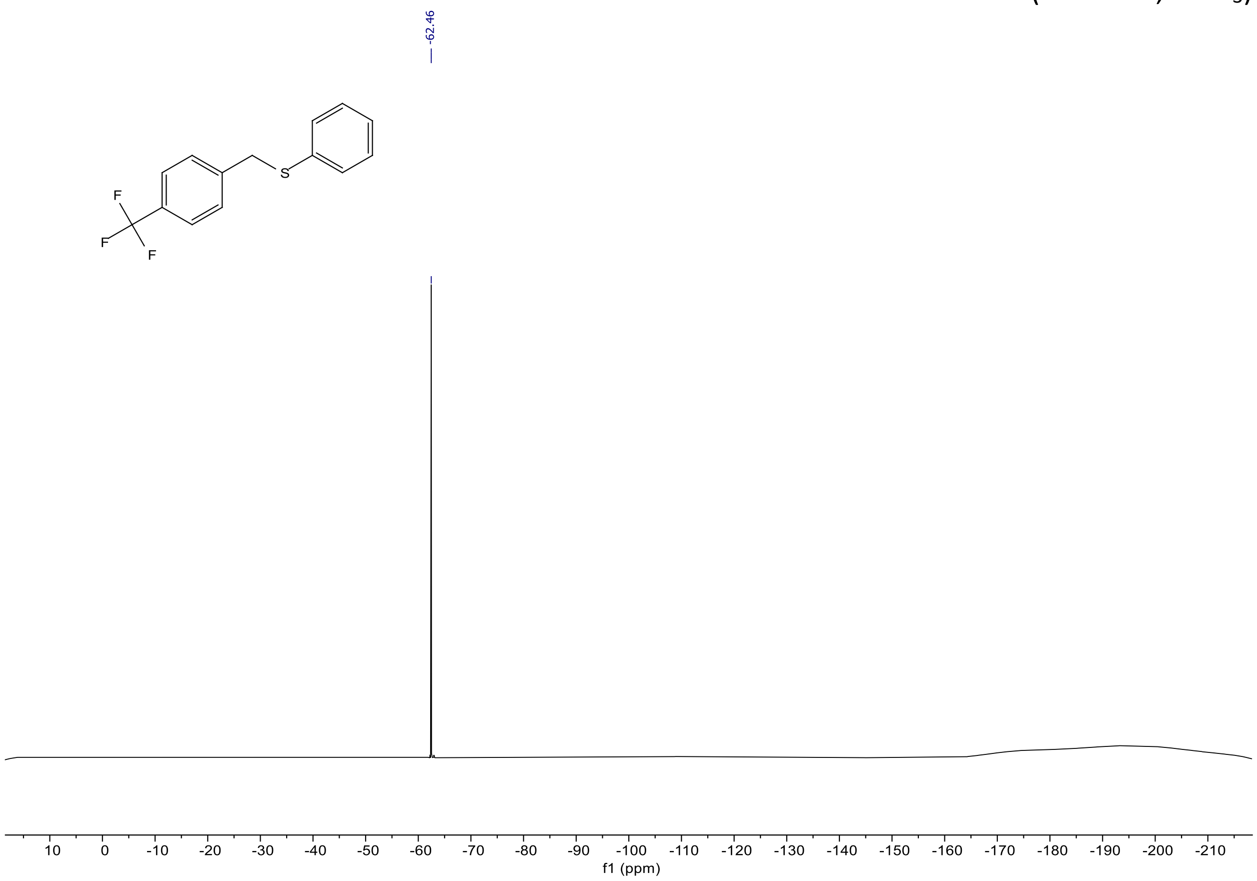

S10.12 1k

^{1}H NMR (400 MHz, $CDCl_3$)

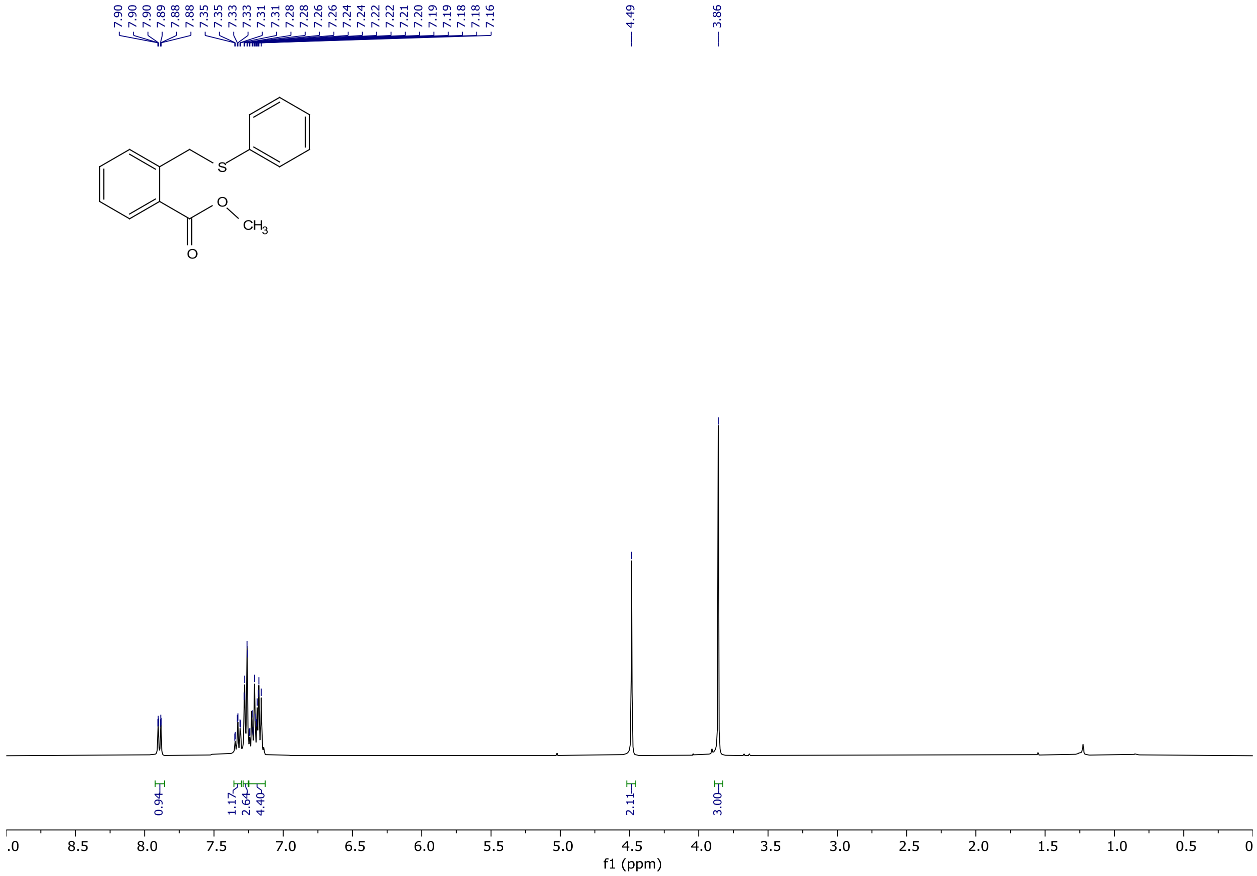

^{13}C NMR (101 MHz, $CDCl_3$)

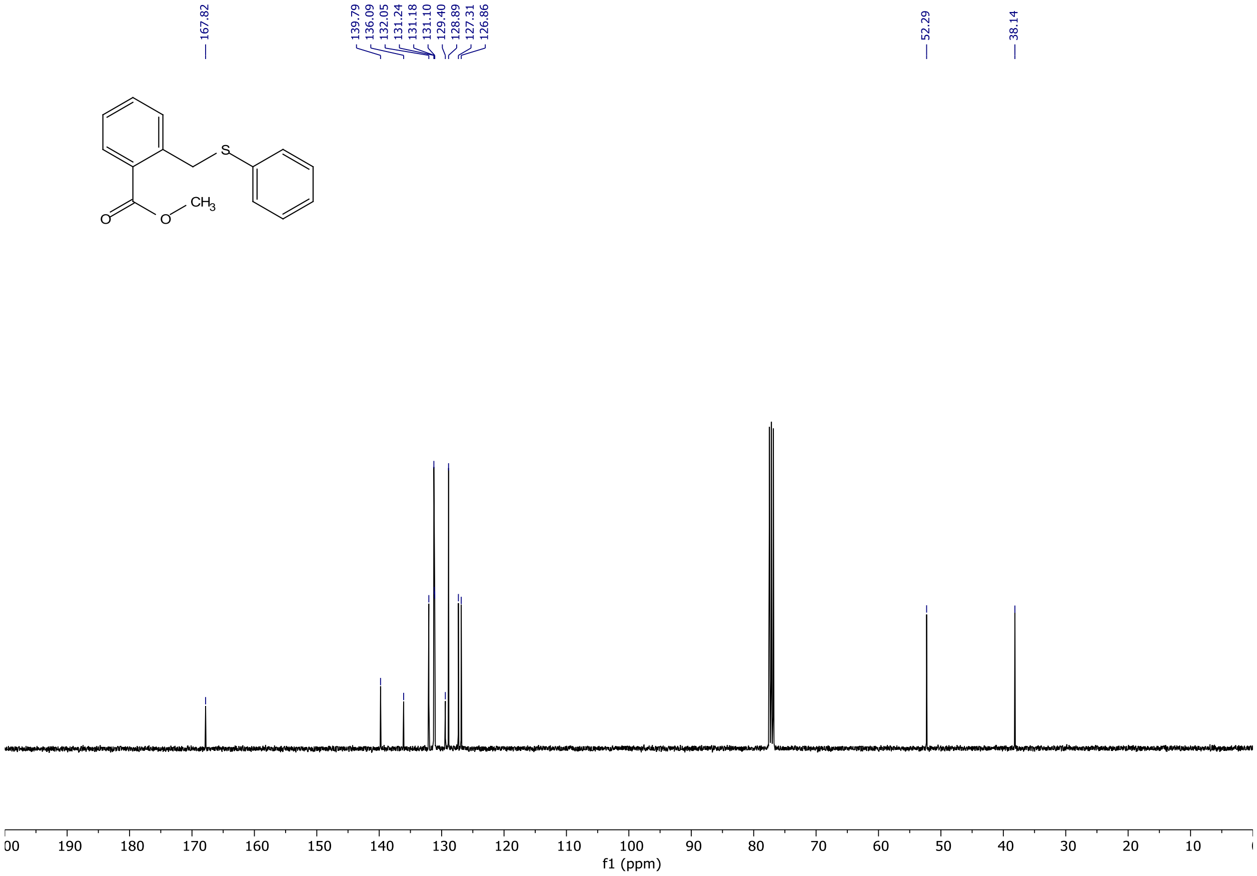

S10.13 1l

^{1}H NMR (400 MHz, $CDCl_3$)

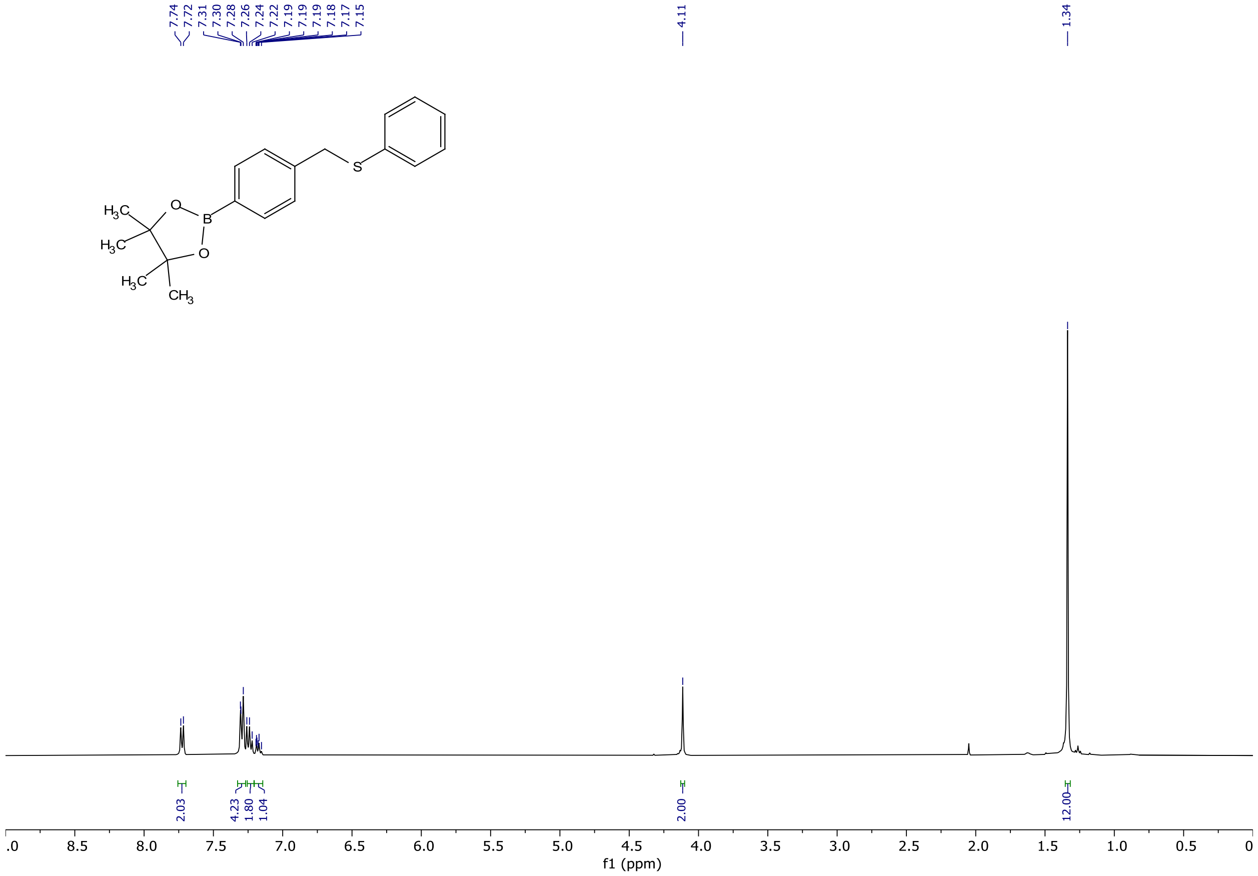

^{13}C NMR (101 MHz, $CDCl_3$)

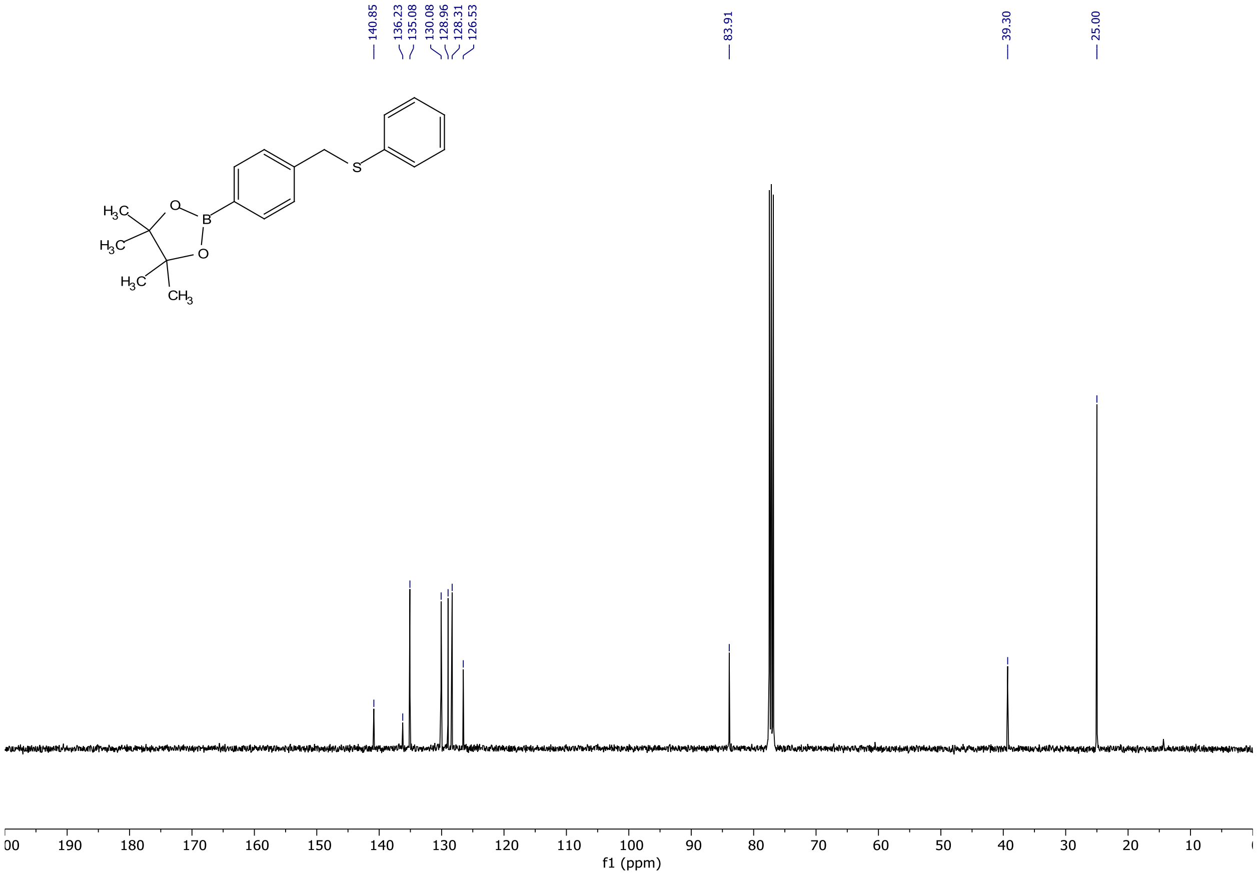

^{11}B NMR (128 MHz, $CDCl_3$)

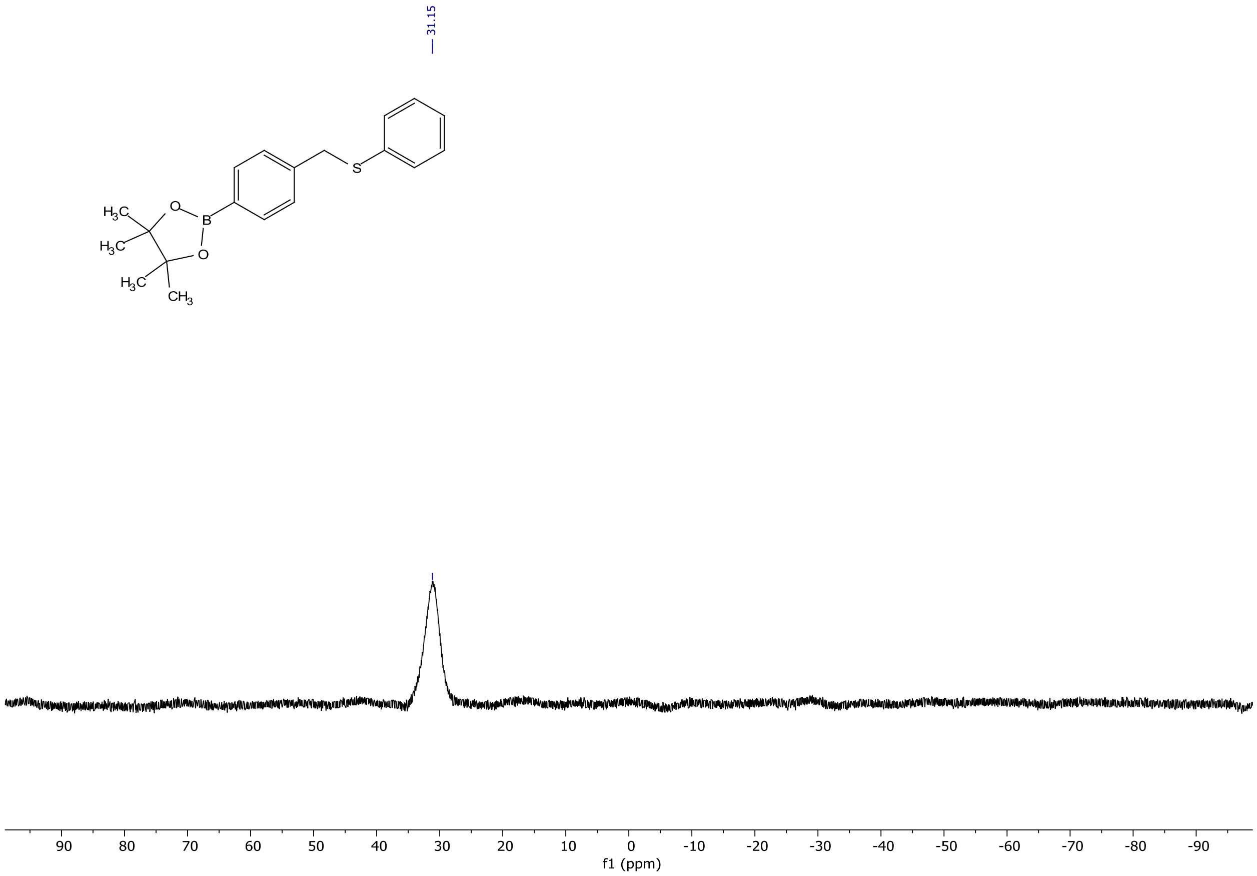

S10.14 1m

¹H NMR (500 MHz, $CDCl_3$)

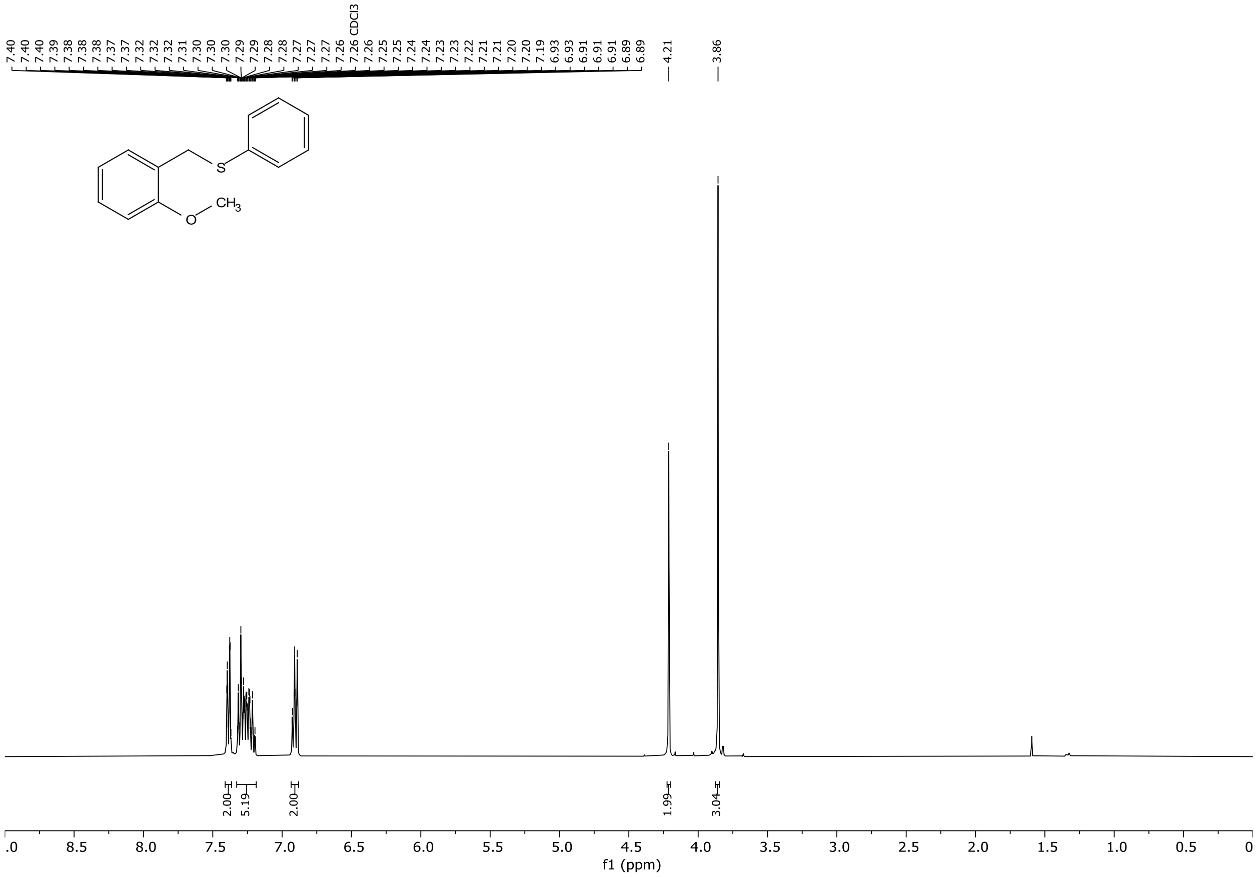

^{13}C NMR (126 MHz, $CDCl_3$)

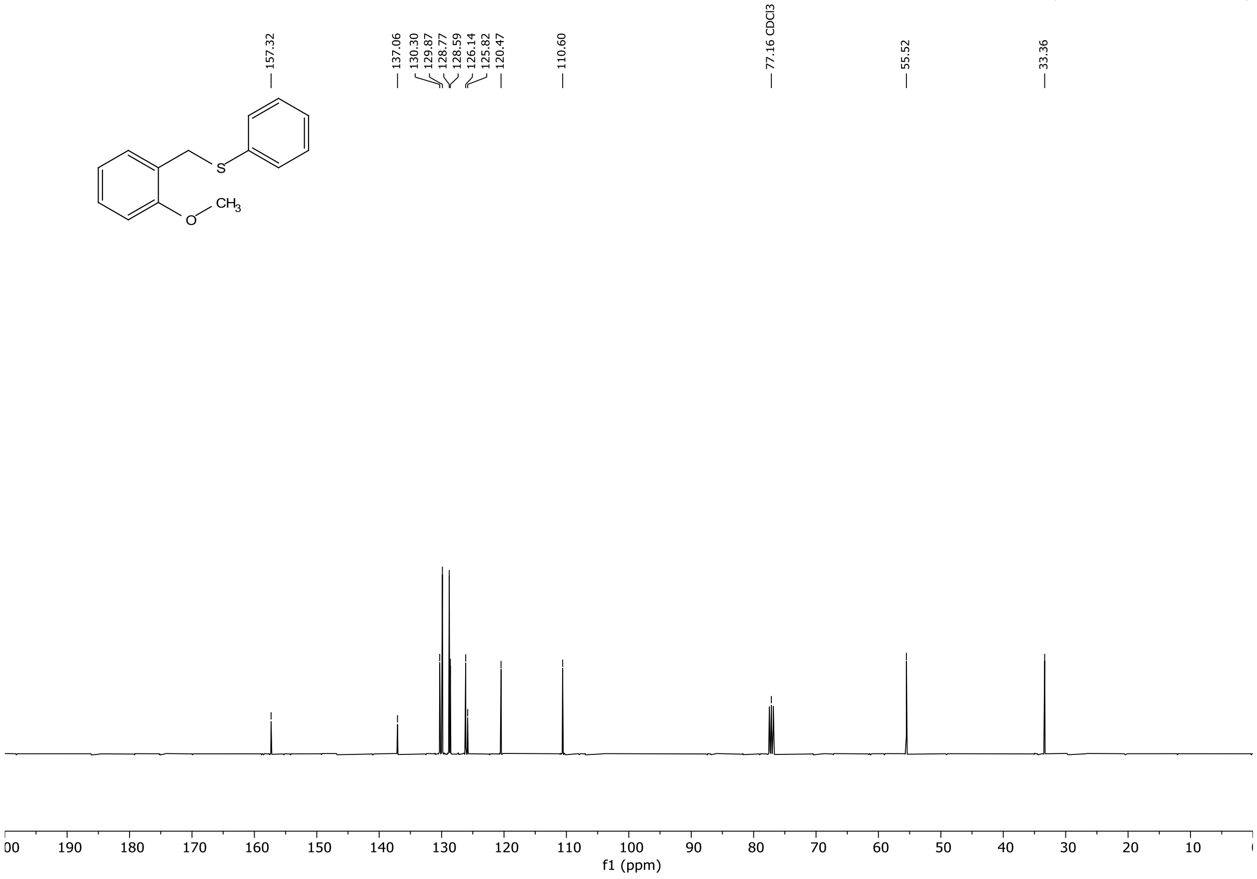

S10.15 1n

^{1}H NMR (500 MHz, $CDCl_3$)

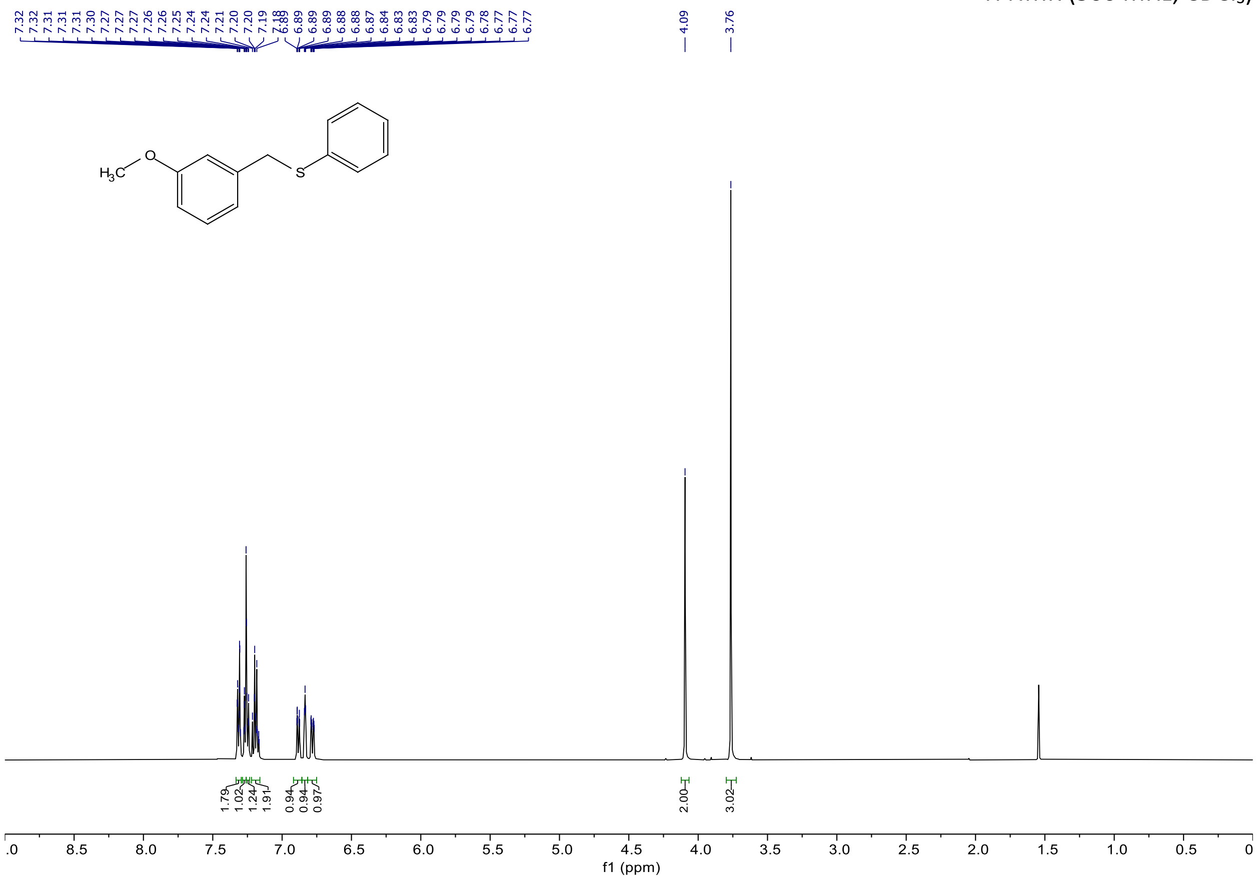

^{13}C NMR (126 MHz, $CDCl_3$)

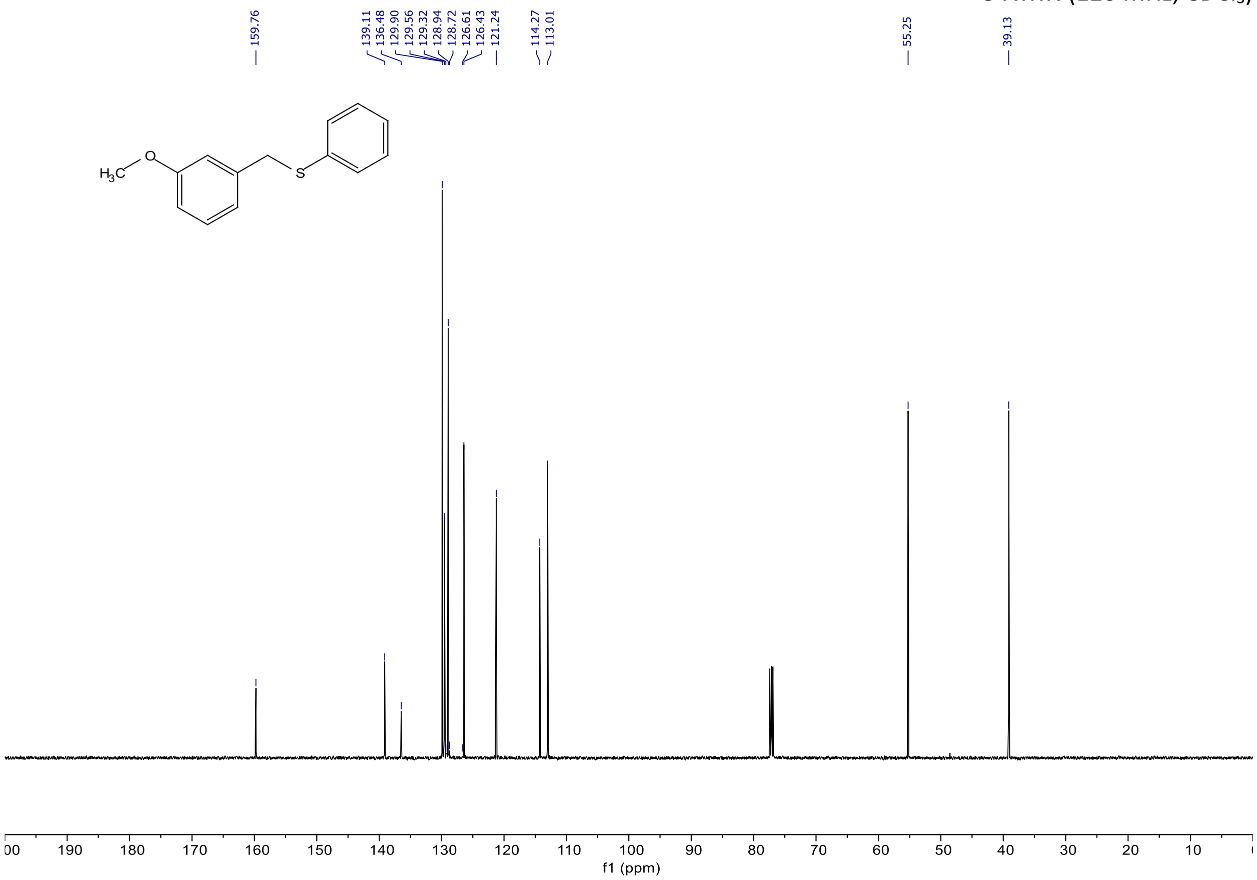

S10.16 1o

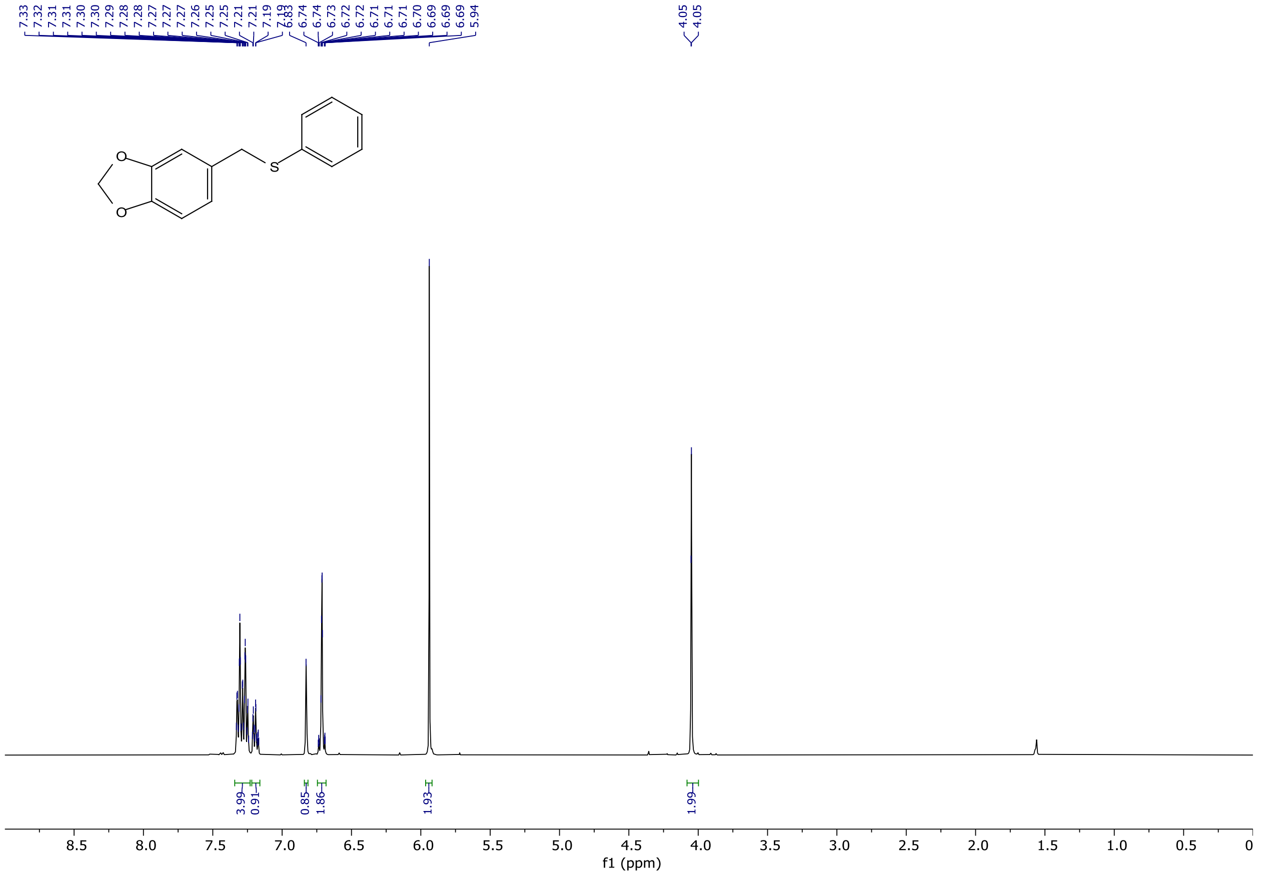

^{1}H NMR (400 MHz, $CDCl_3$)

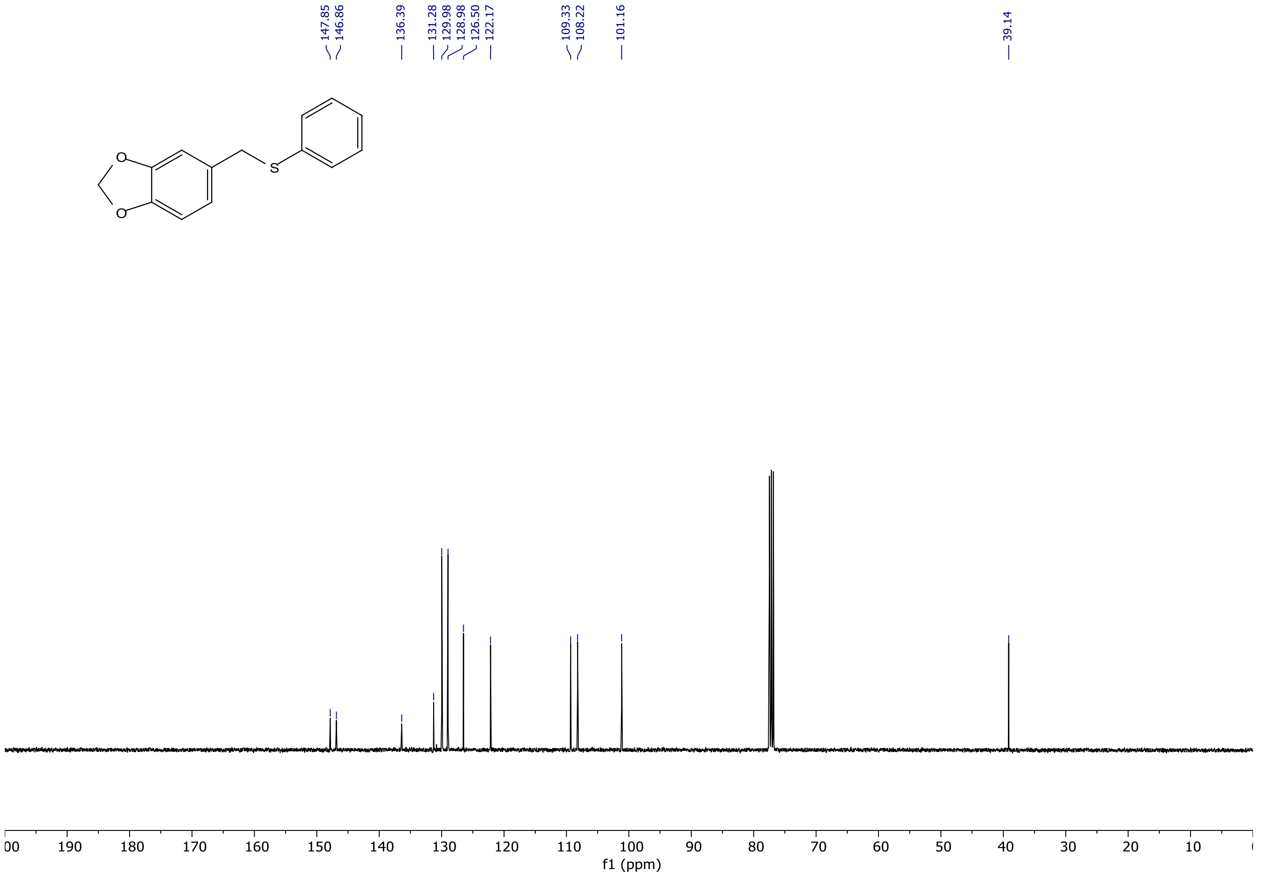

^{13}C NMR (101 MHz, $CDCl_3$)
147.85
146.86
136.39
131.28
129.98
128.98
126.50
122.17
109.33
108.22
101.16
39.14
f1 (ppm)

S10.17 1p

^1H NMR (400 MHz, $CDCl_3$)

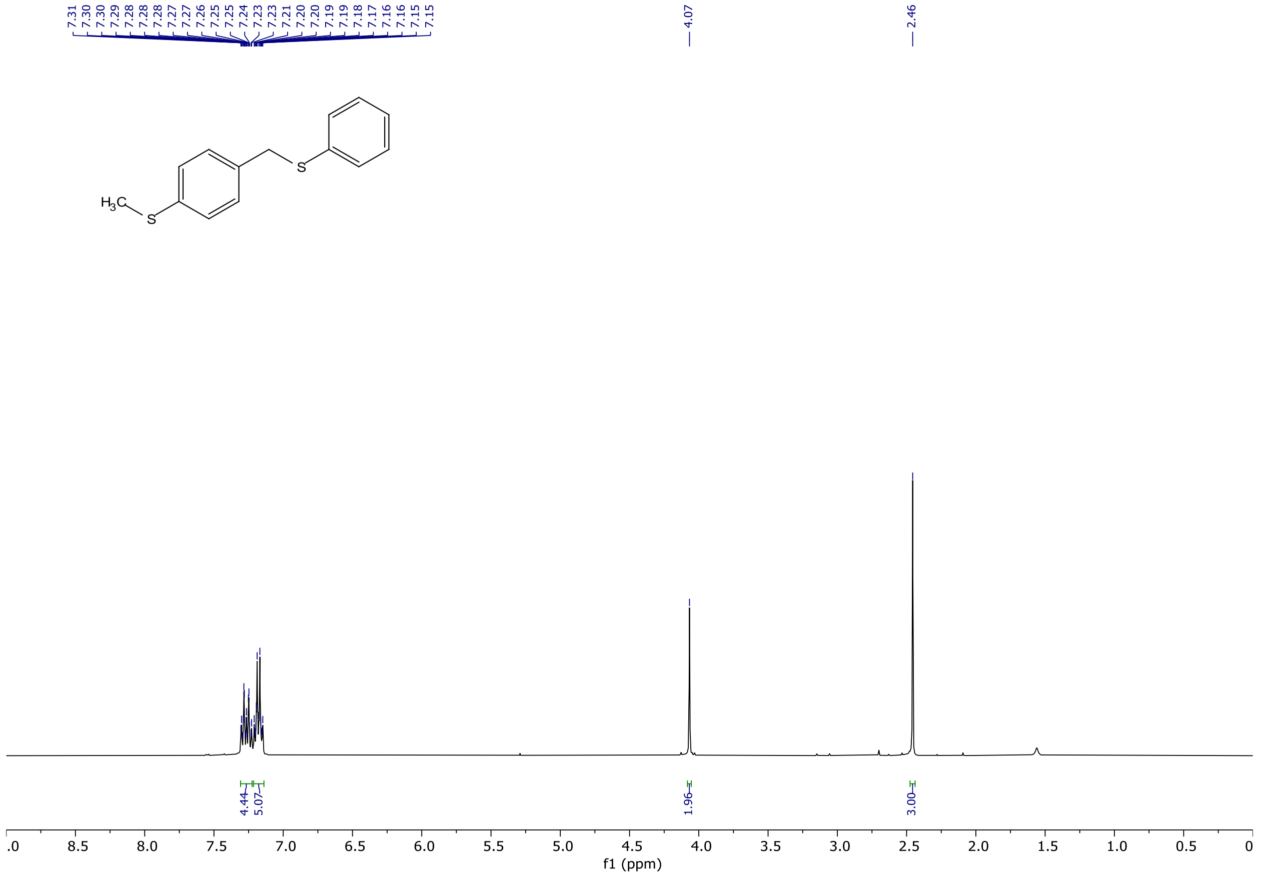

^{13}C NMR (101 MHz, $CDCl_3$)

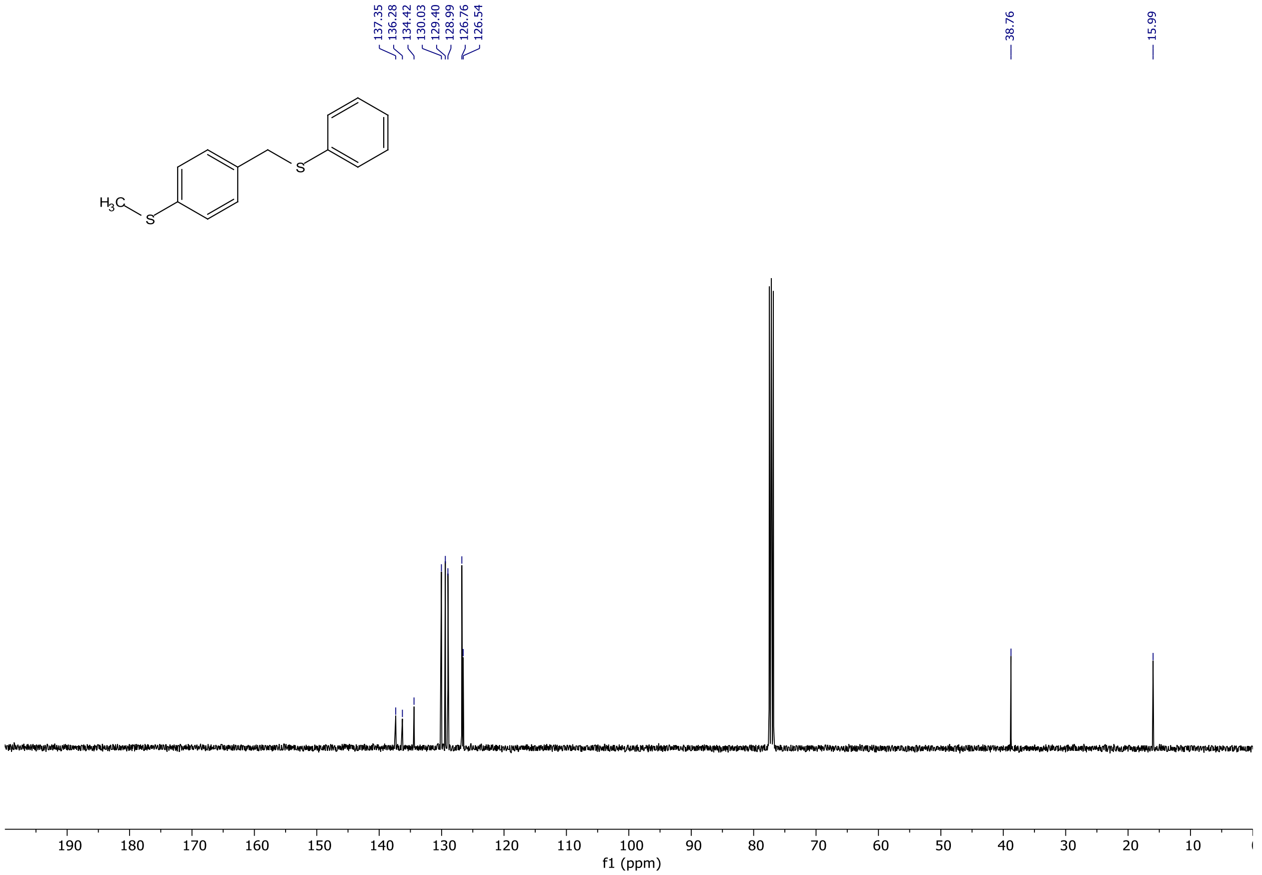

S10.18 1q

^{1}H NMR (500 MHz, $CDCl_3$)

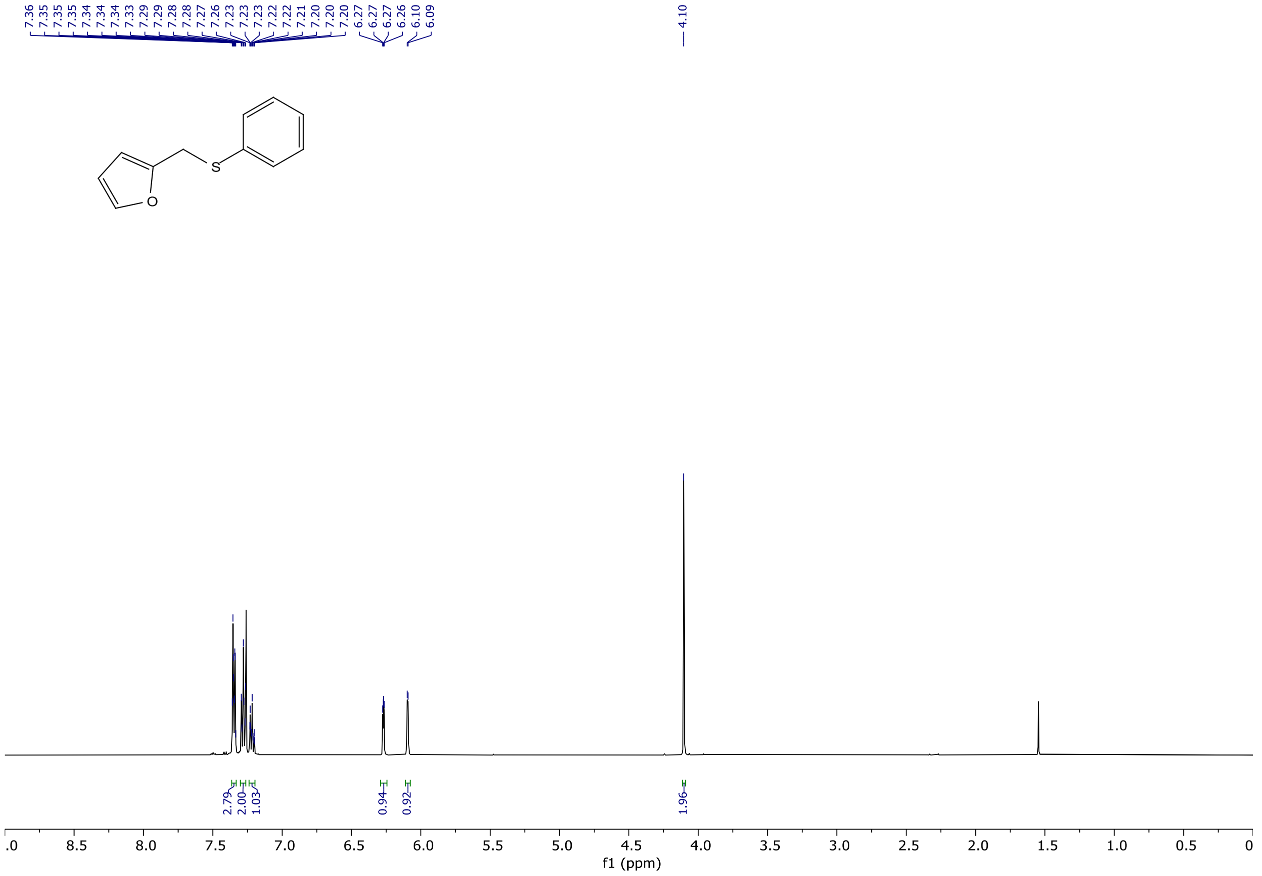

^{13}C NMR (126 MHz, $CDCl_3$)

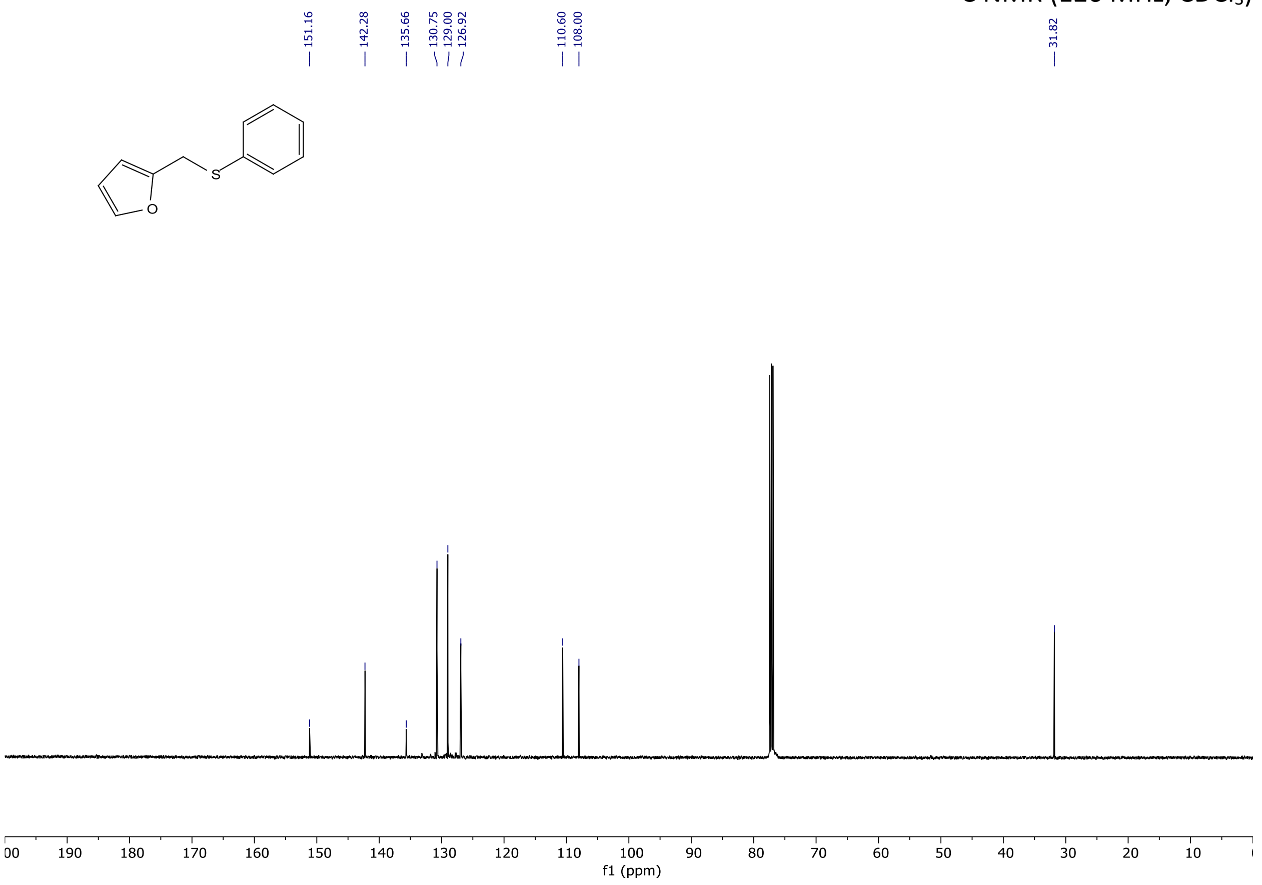

S10.19 1r

^1H NMR (400 MHz, $CDCl_3$)

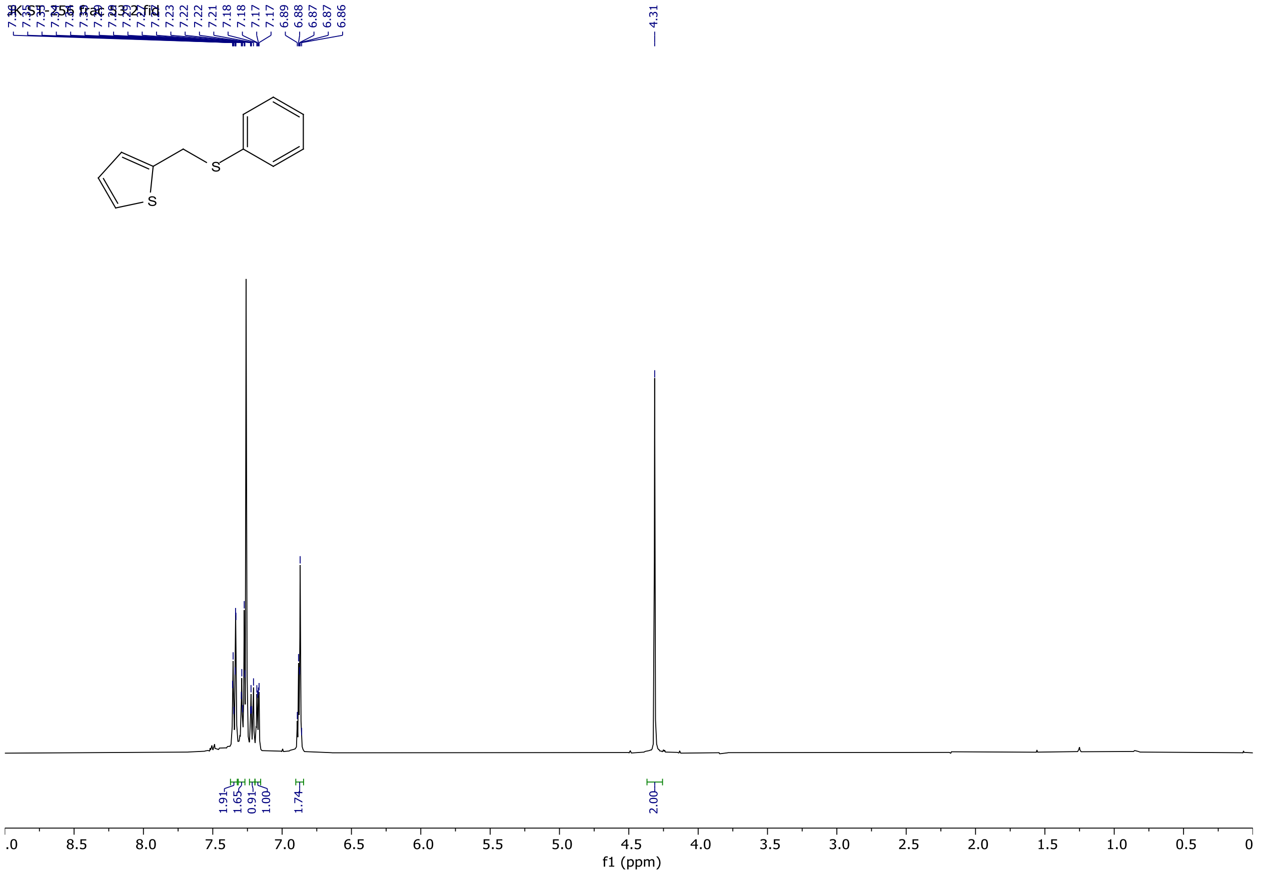

^{13}C NMR (101 MHz, $CDCl_3$)

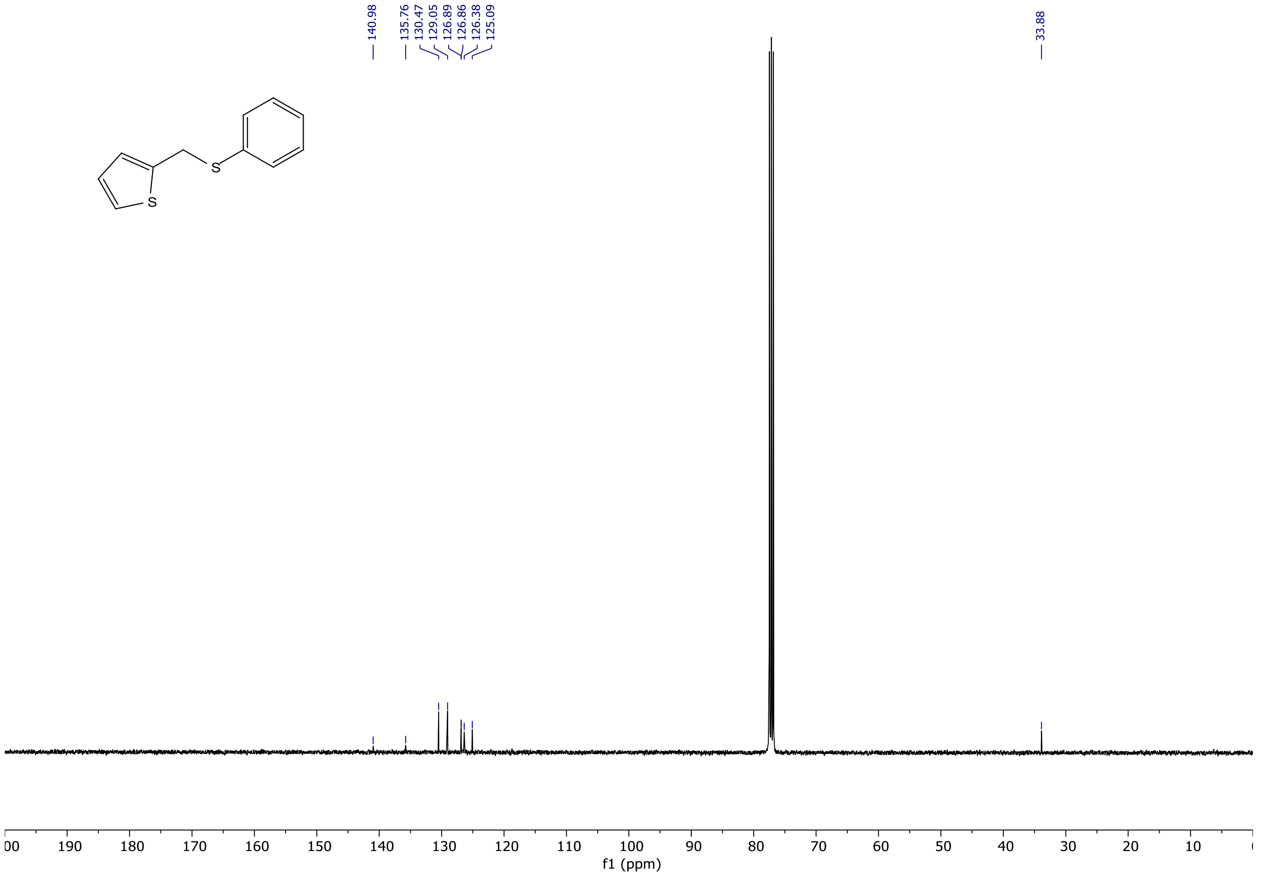

S10.20 1s

^{1}H NMR (400 MHz, $CDCl_3$)

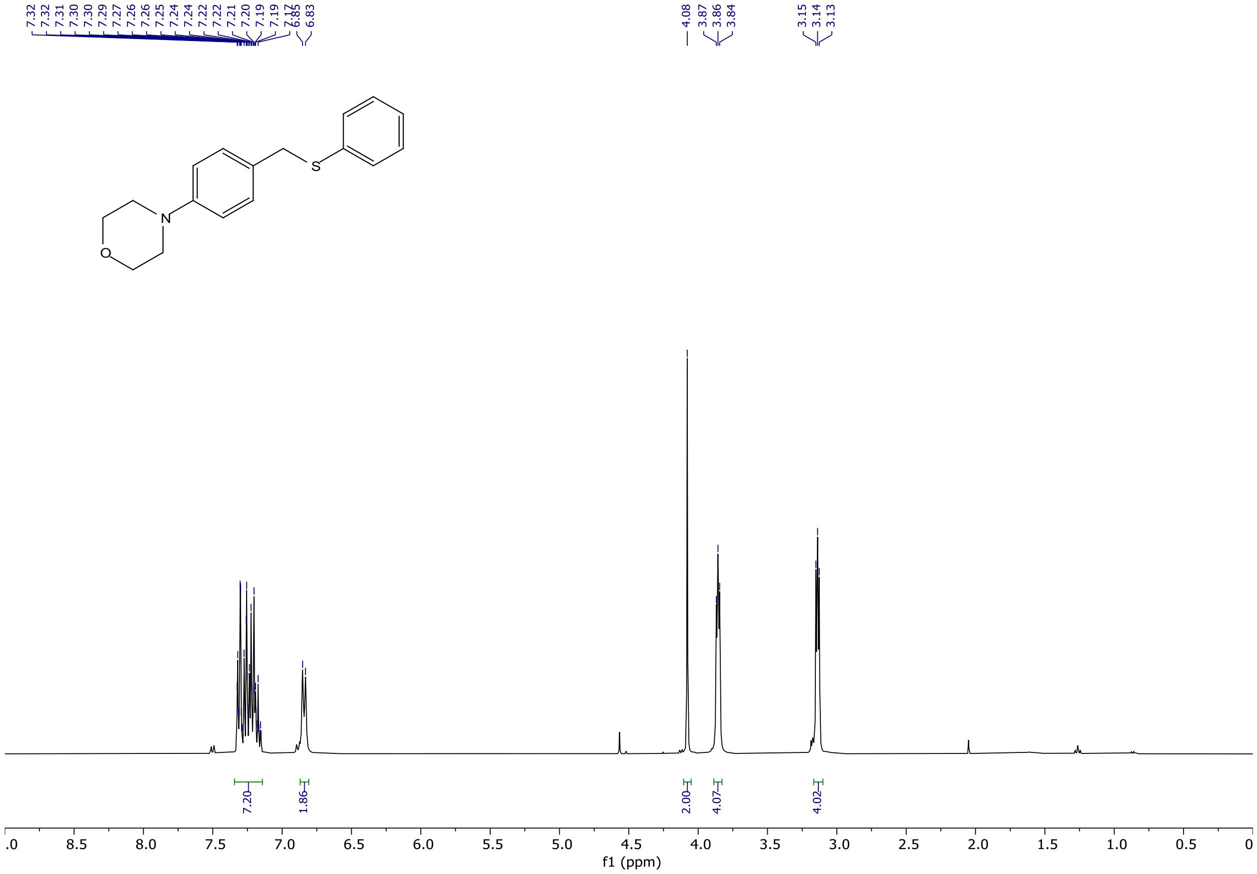

^{13}C NMR (101 MHz, $CDCl_3$)

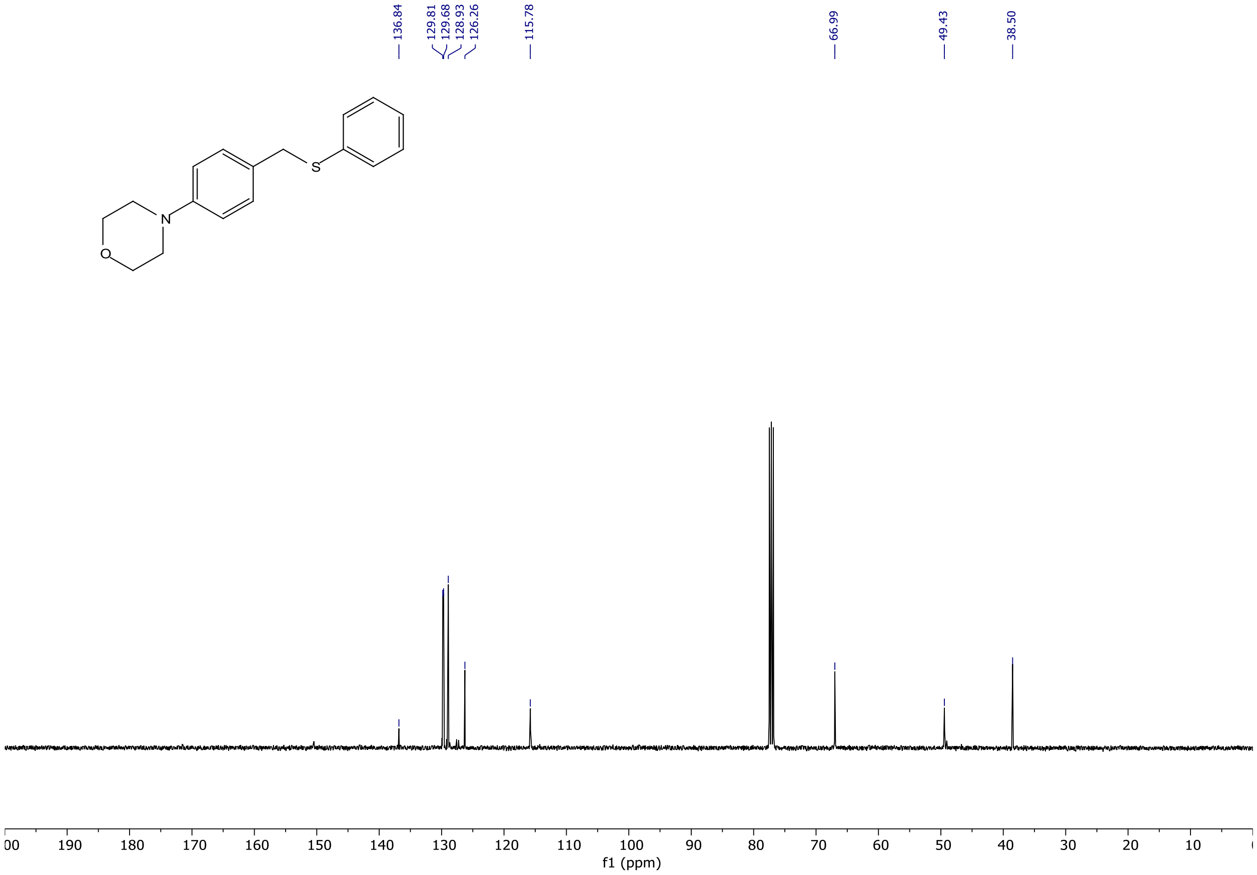

S10.21 1t

^{1}H NMR (500 MHz, $CDCl_3$)

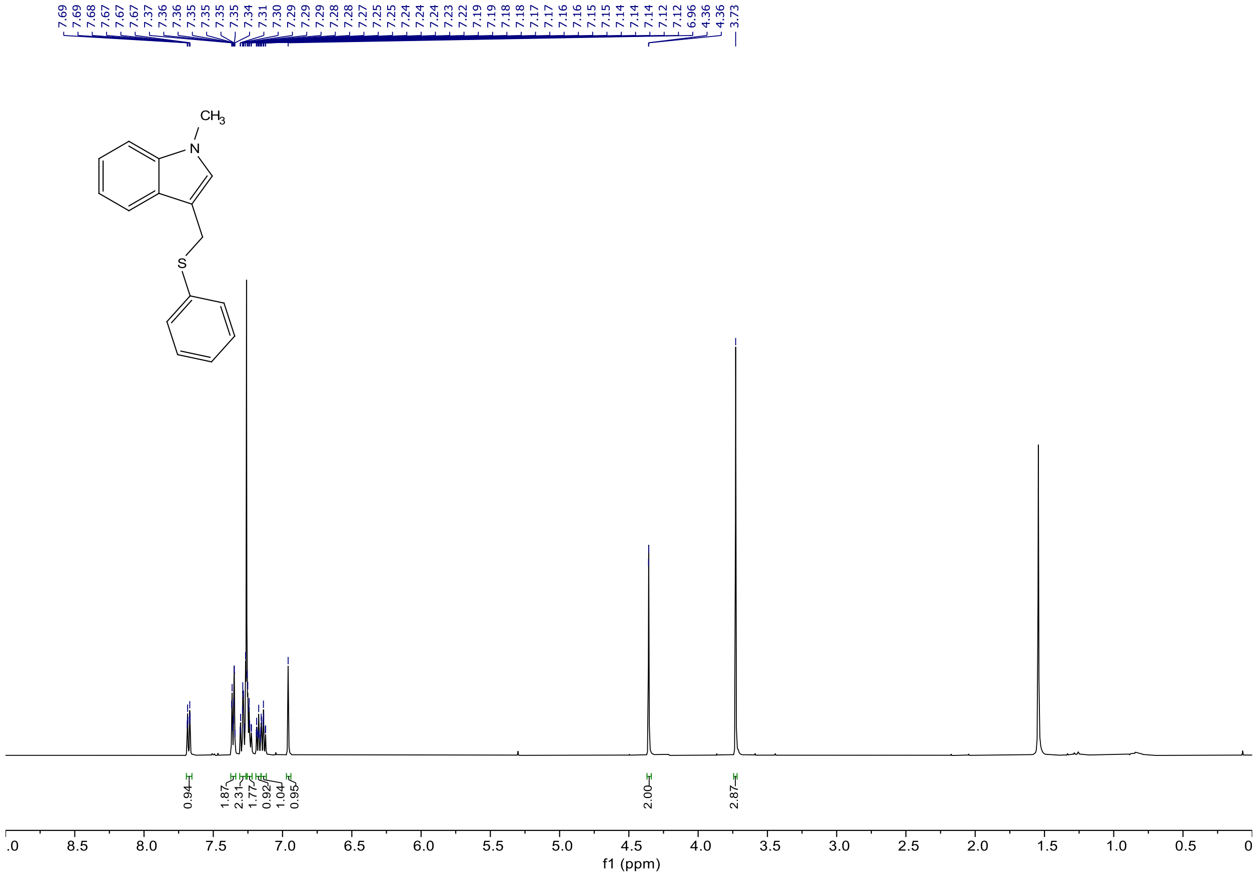

^{13}C NMR (126 MHz, $CDCl_3$)

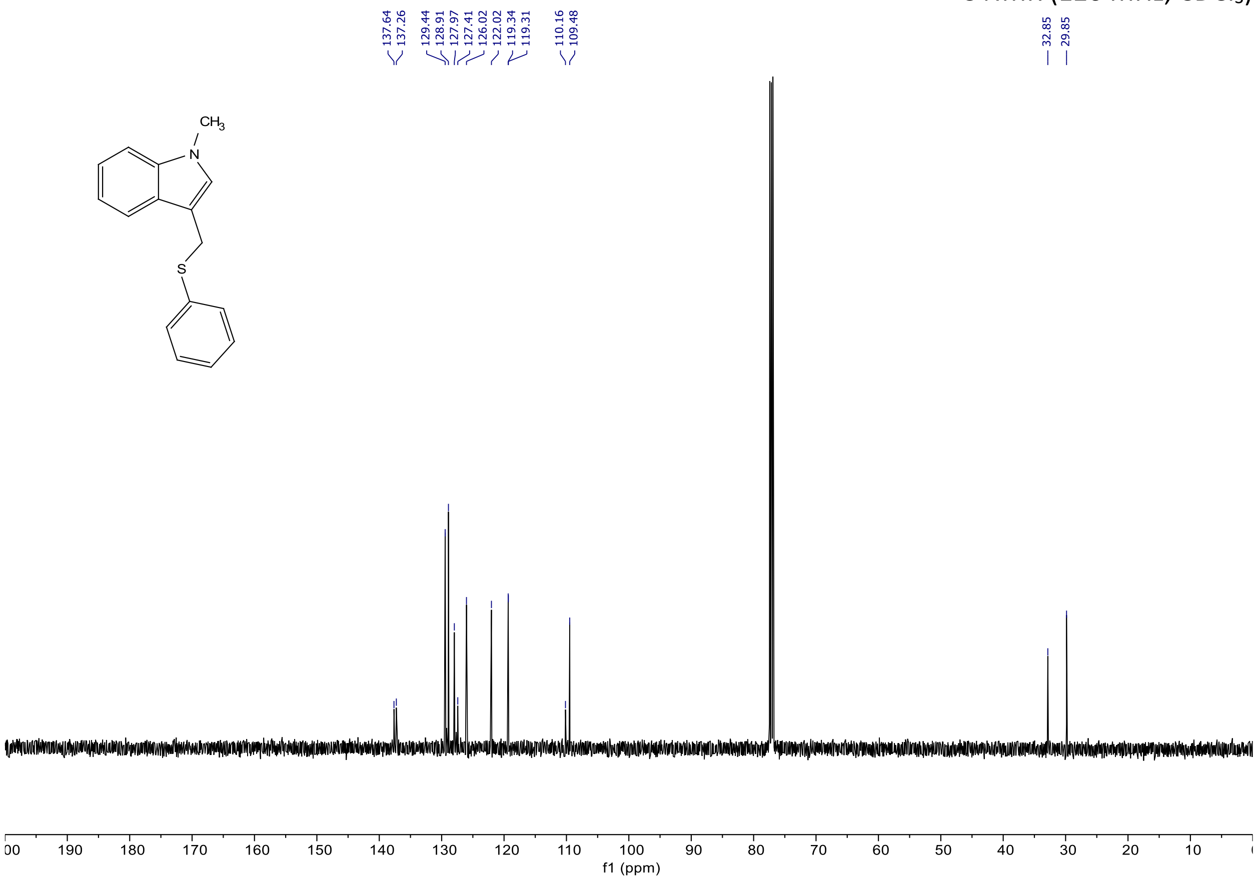

S10.22 1u

^{1}H NMR (400 MHz, CDCl$_3$)

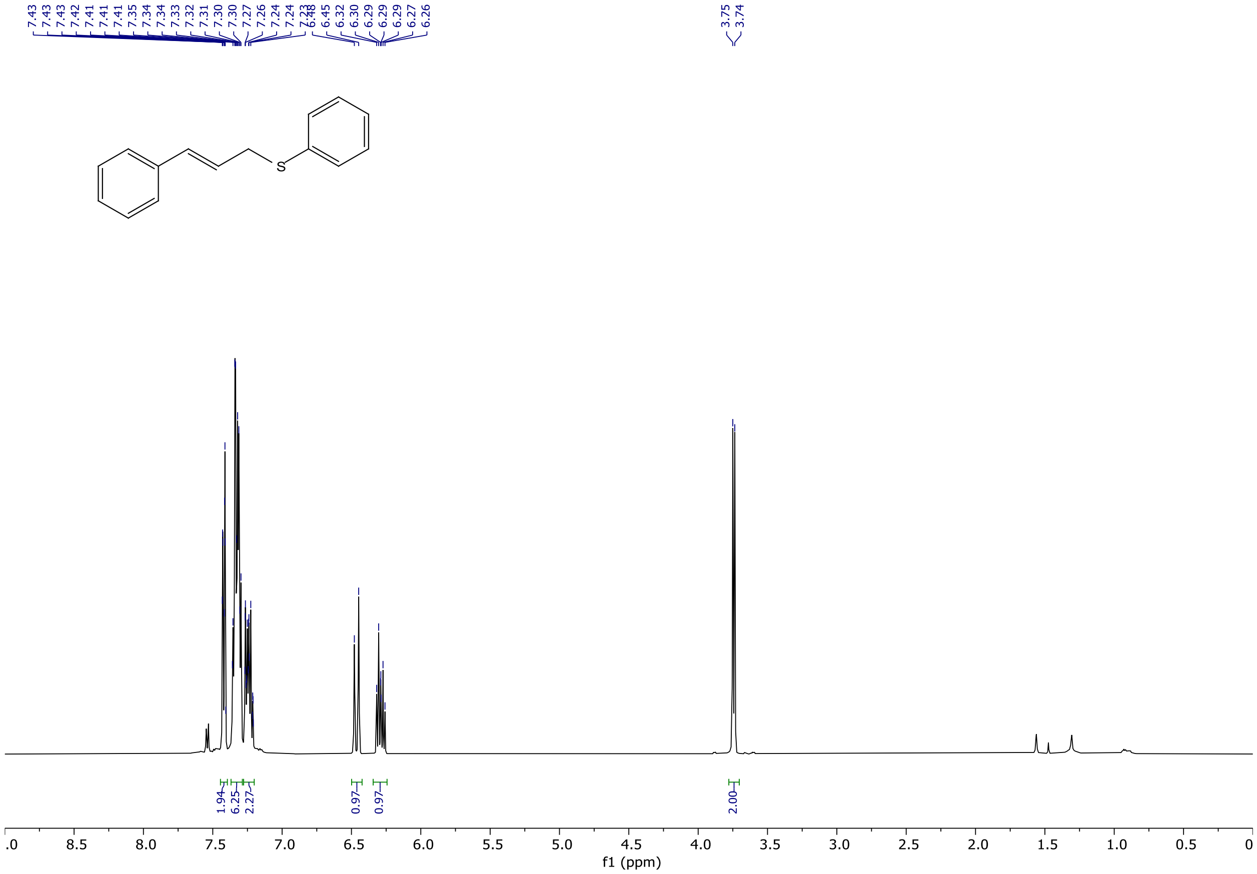

^{13}C NMR (101 MHz, CDCl$_3$)

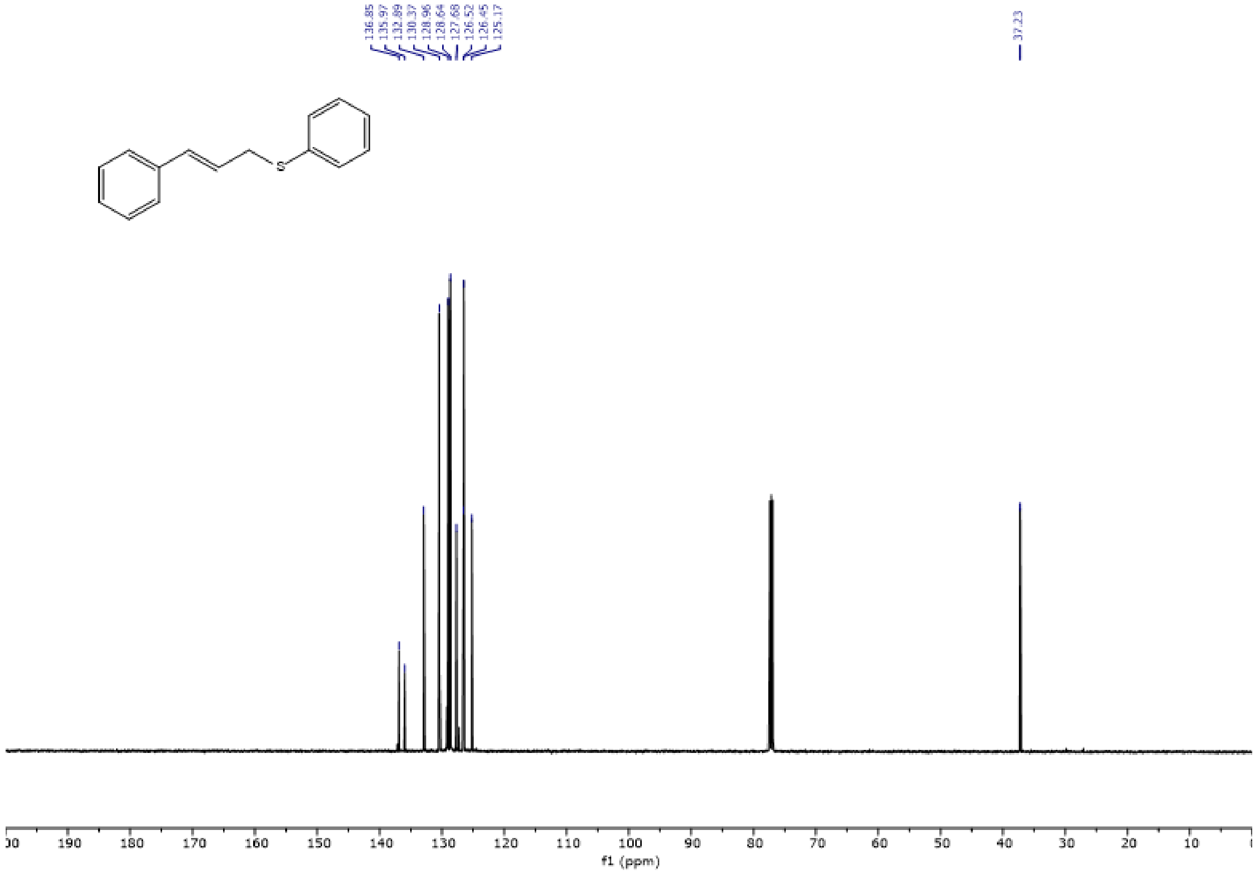

S10.23　　1v

^1H NMR (400 MHz, $CDCl_3$)

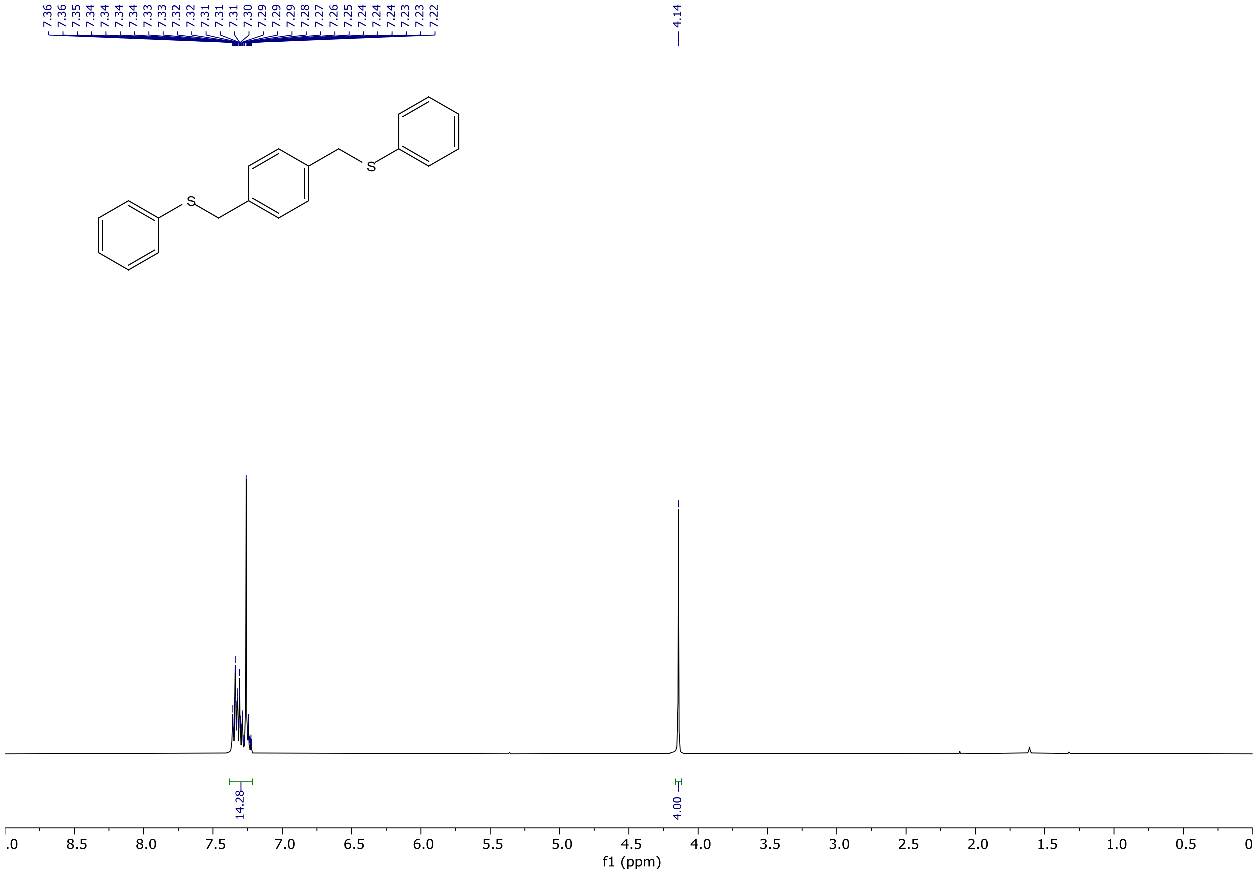

^{13}C NMR (101 MHz, $CDCl_3$)

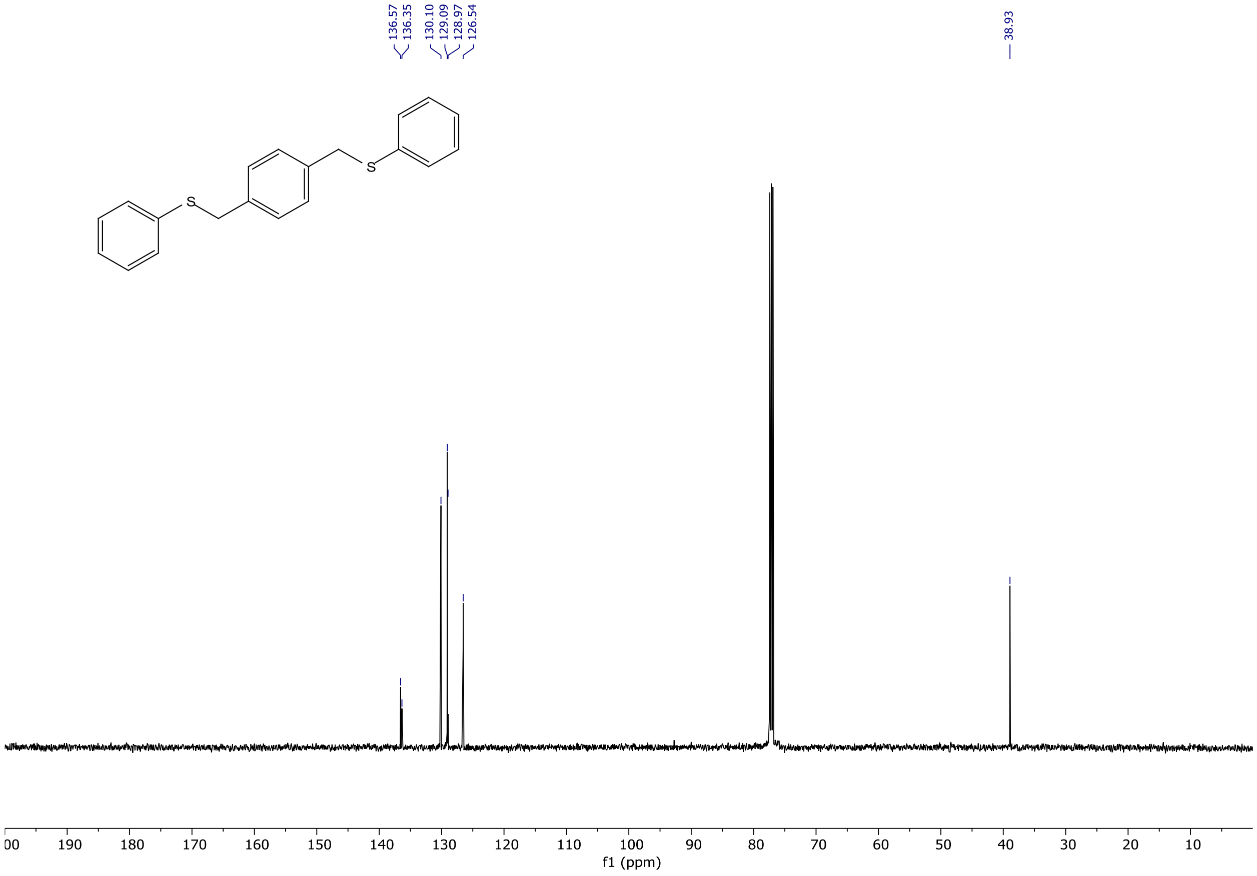

S10.24 1w

^{1}H NMR (400 MHz, $CDCl_3$)

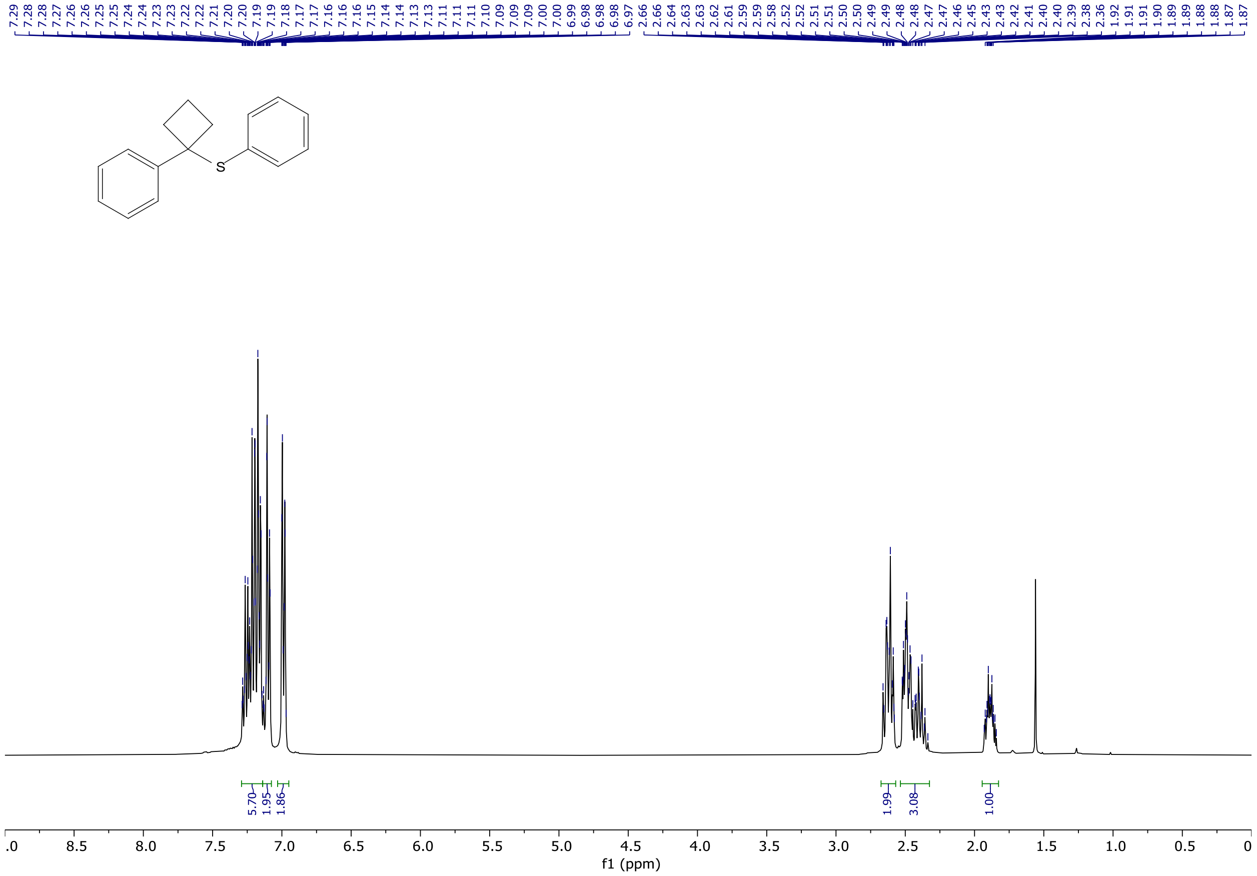

^{13}C NMR (101 MHz, $CDCl_3$)

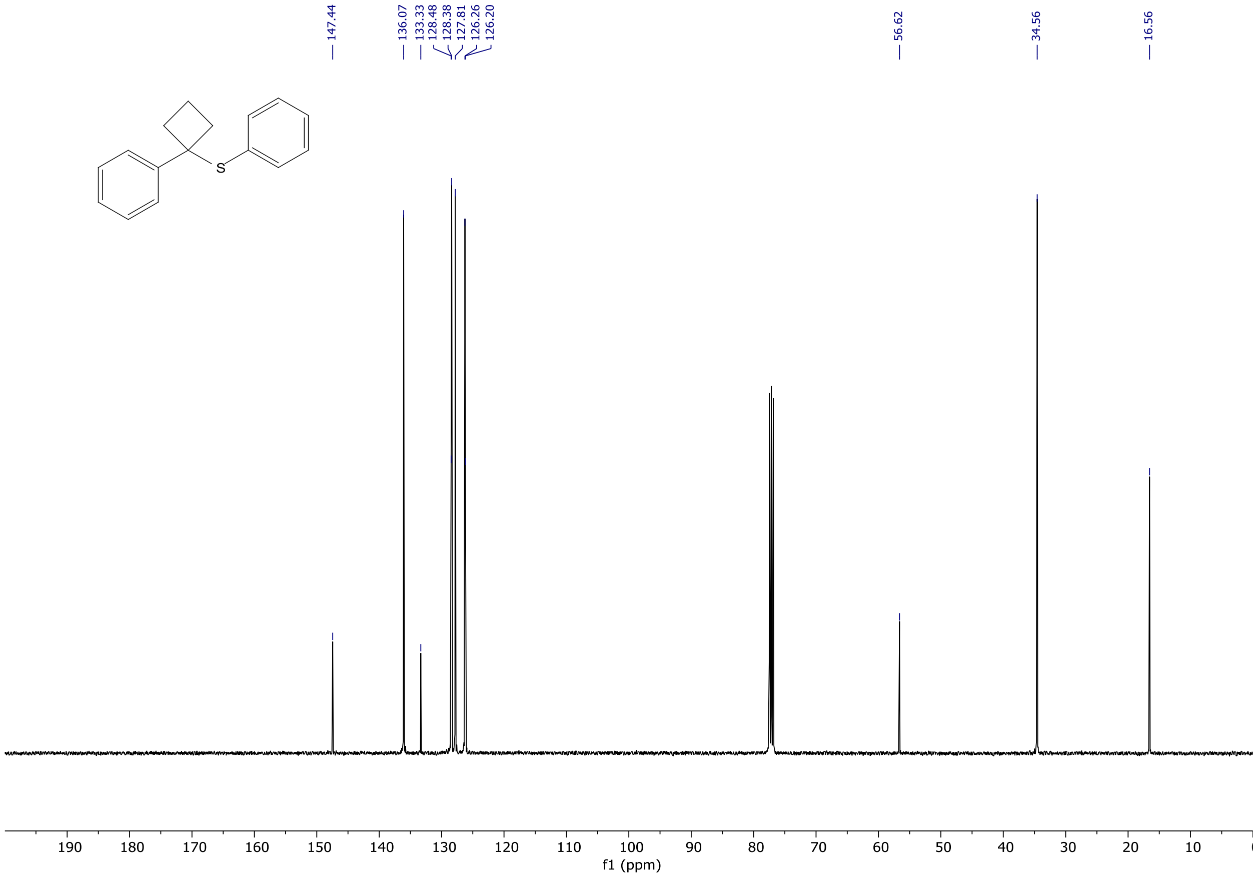

S10.25 1x

^{1}H NMR (400 MHz, $CDCl_3$)

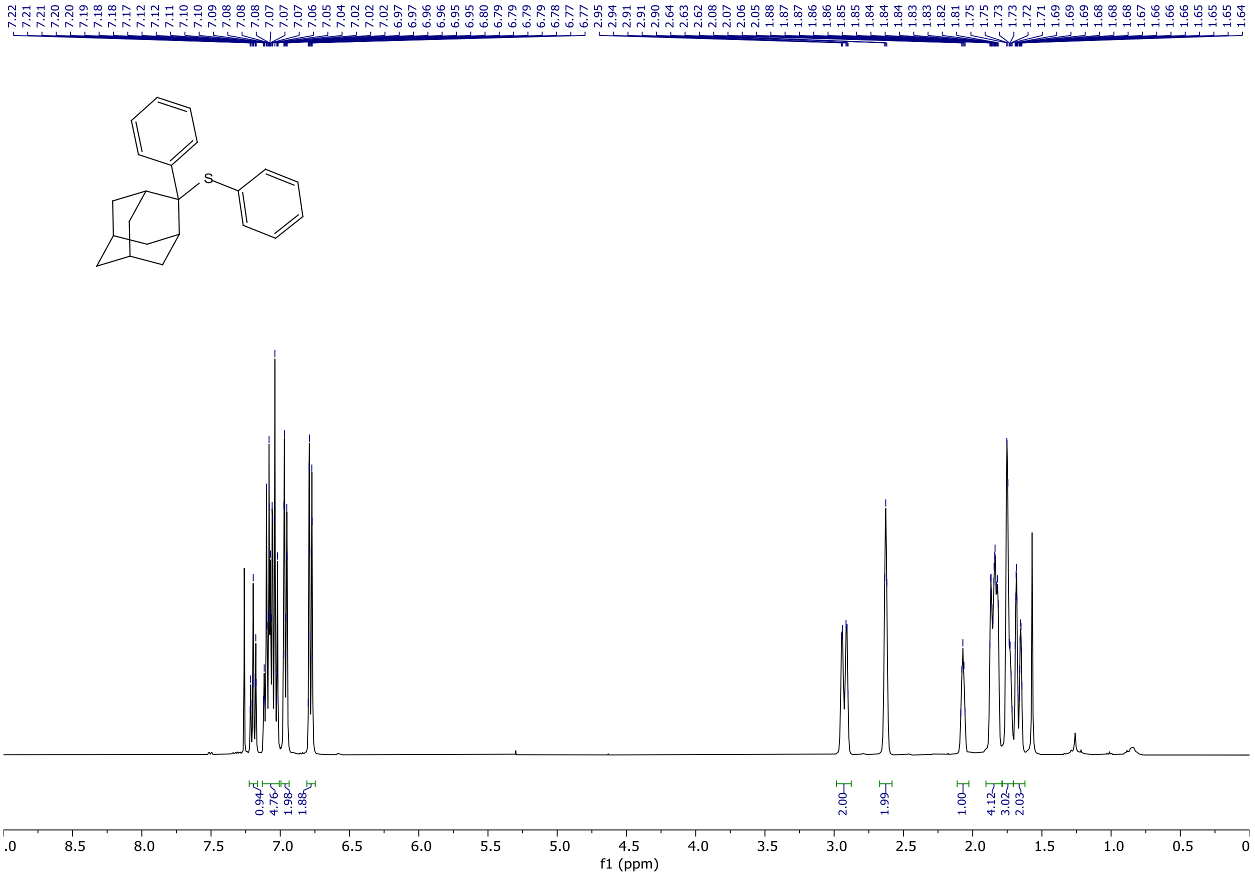

^{13}C NMR (101 MHz, $CDCl_3$)

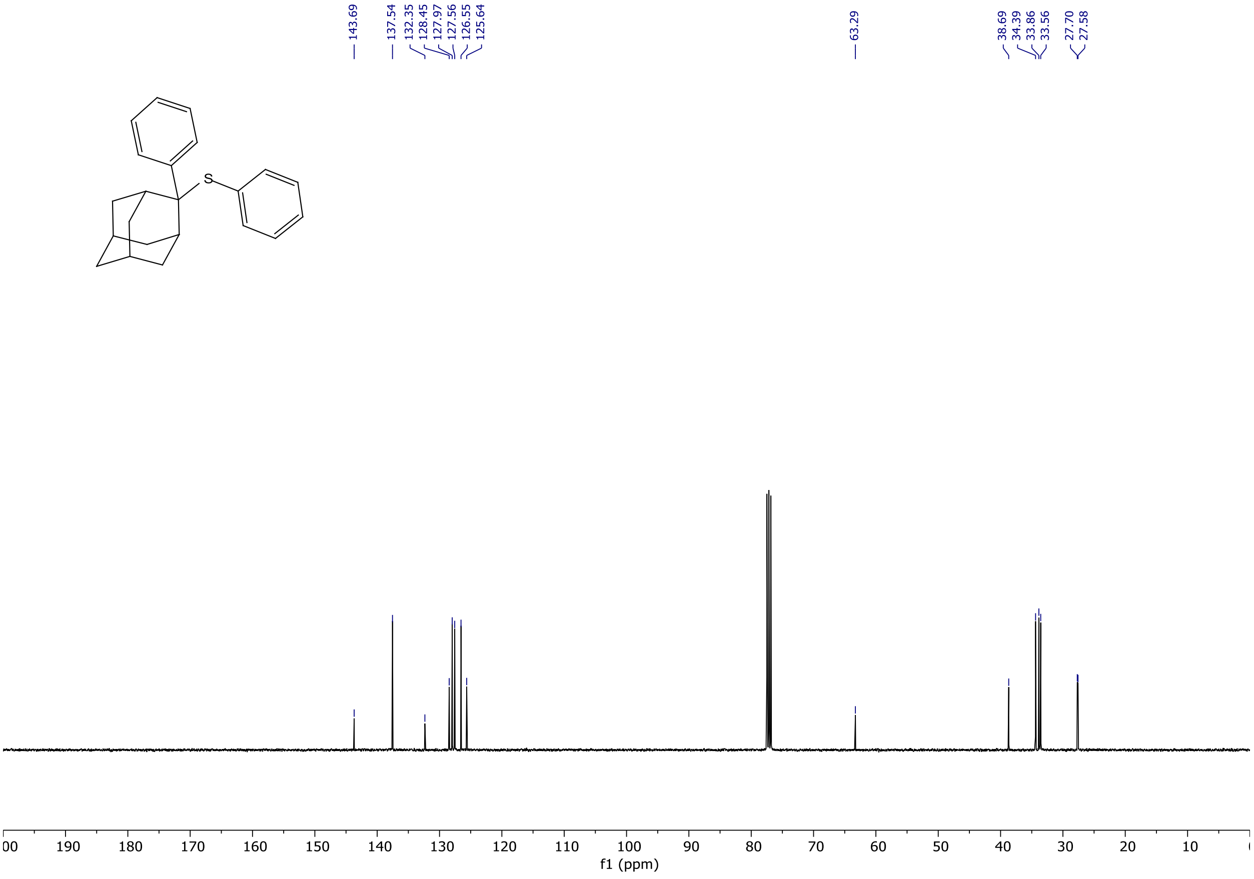

S10.26 1y

^{1}H NMR (400 MHz, $CDCl_3$)

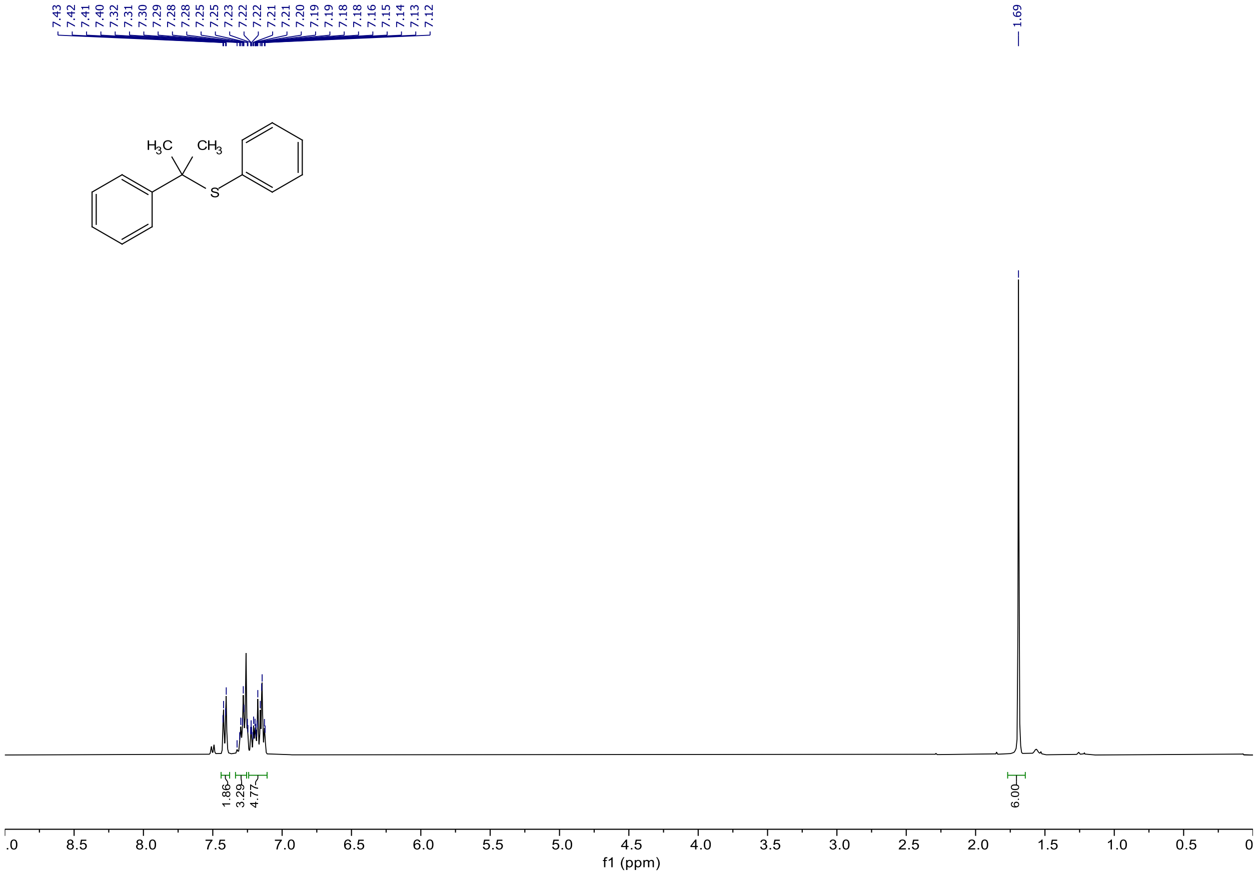

^{13}C NMR (101 MHz, $CDCl_3$)

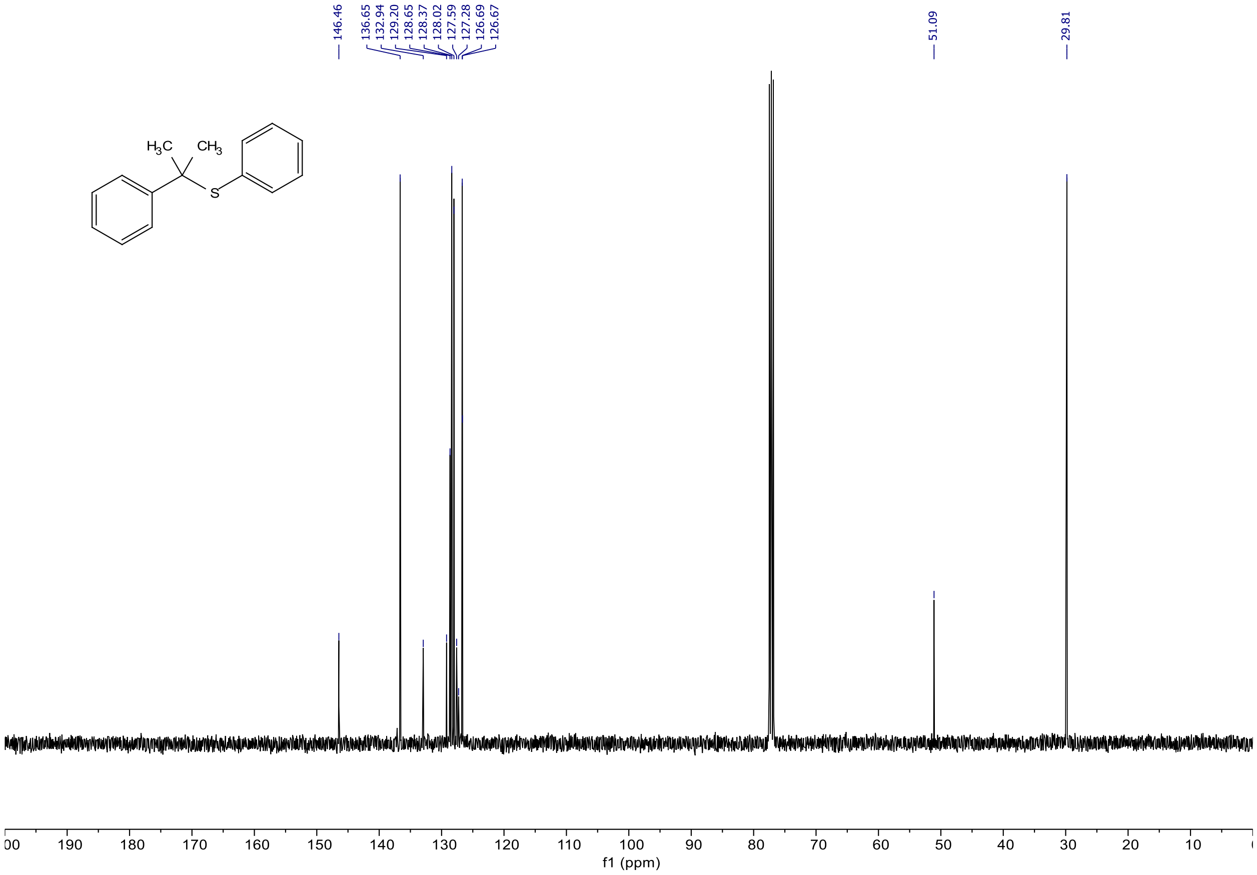

S10.27 1z

^{1}H NMR (500 MHz, $CDCl_3$)

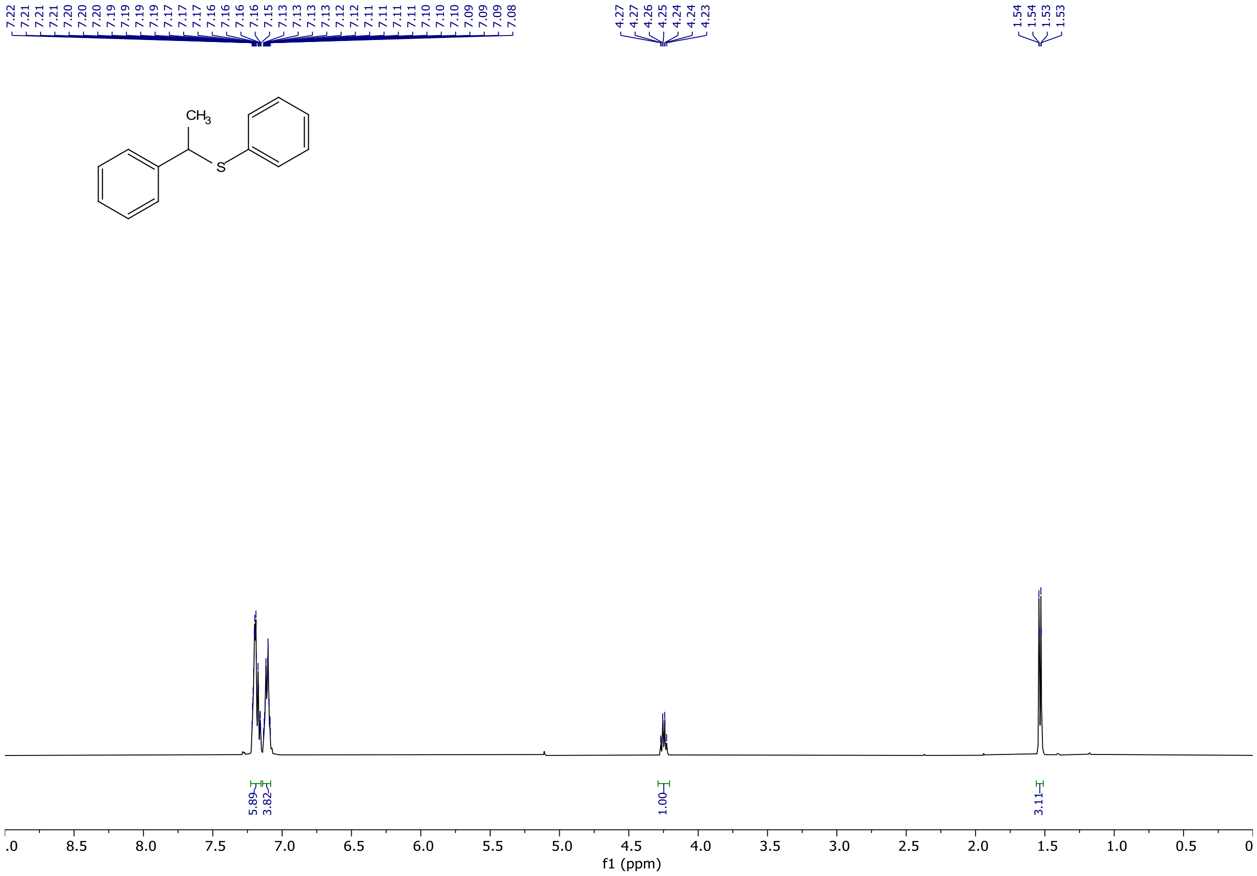

^{13}C NMR (126 MHz, $CDCl_3$)

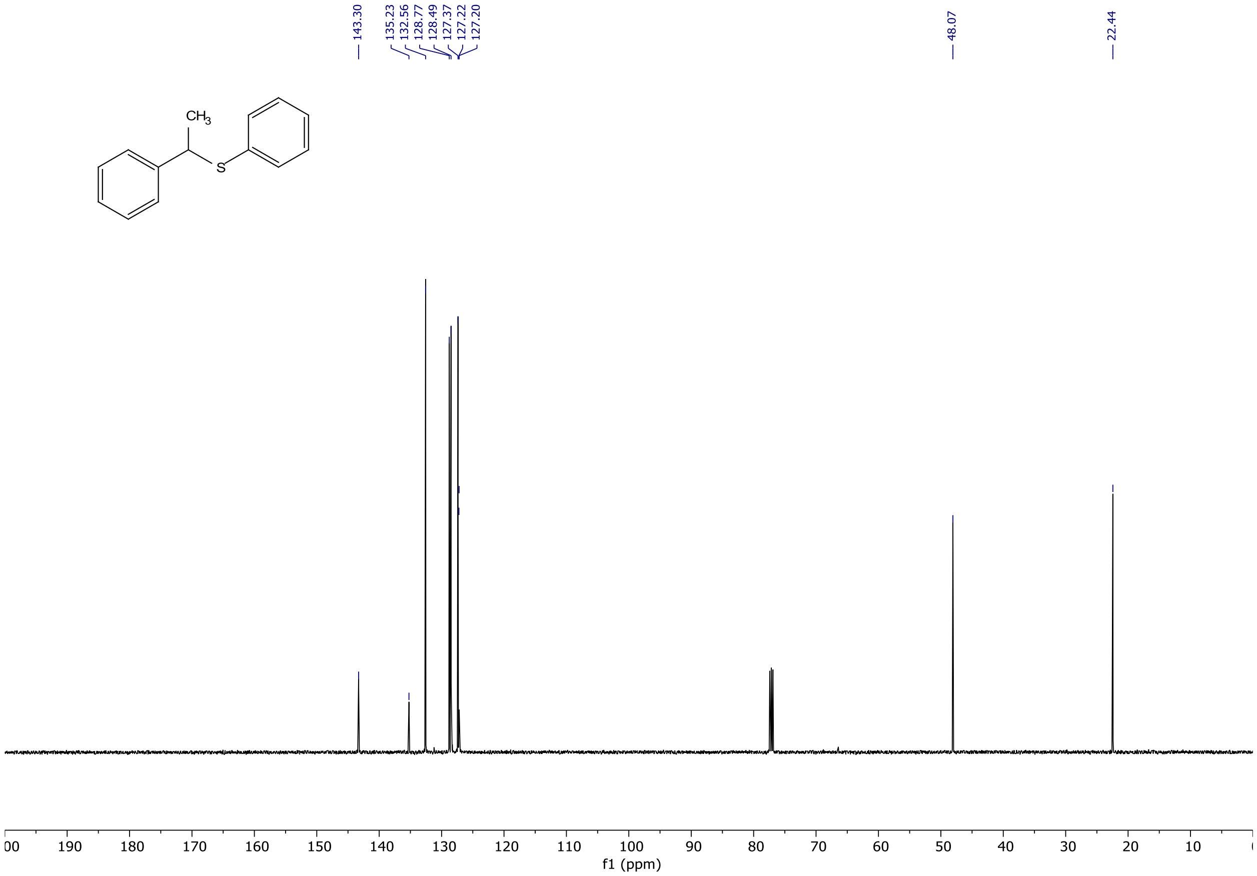

S10.28 1aa

^1H NMR (400 MHz, $CDCl_3$)

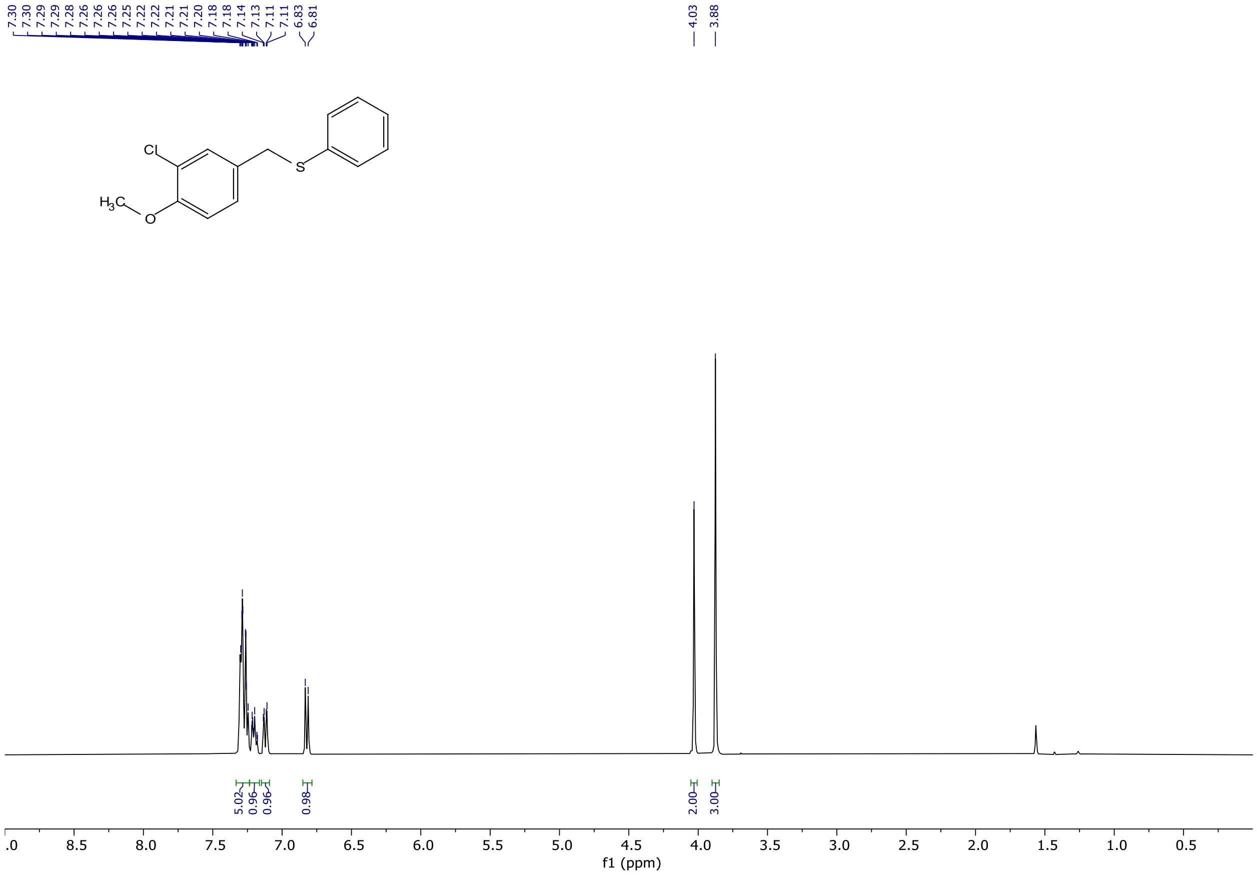

^{13}C NMR (101 MHz, $CDCl_3$)

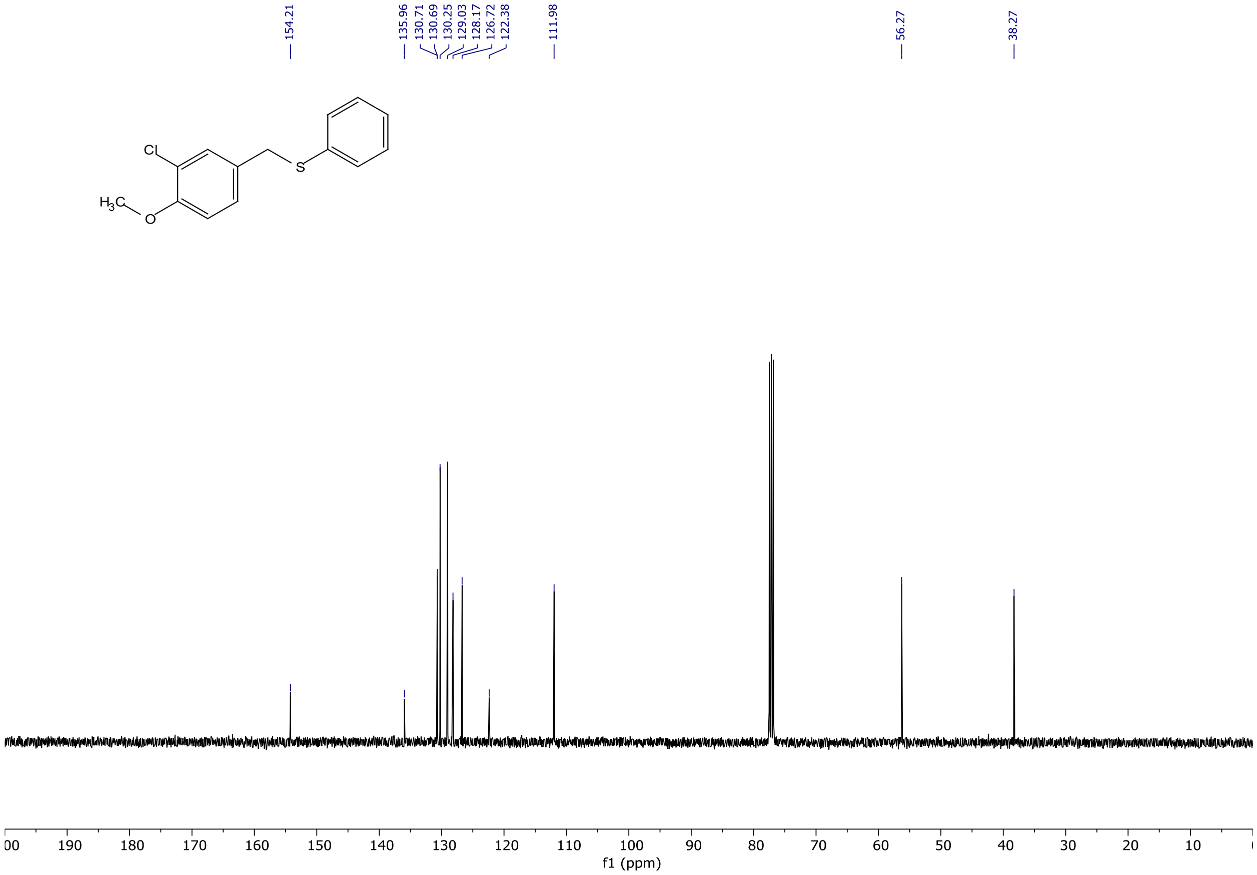

S10.29 1ab

^{1}H NMR (500 MHz, $CDCl_3$)

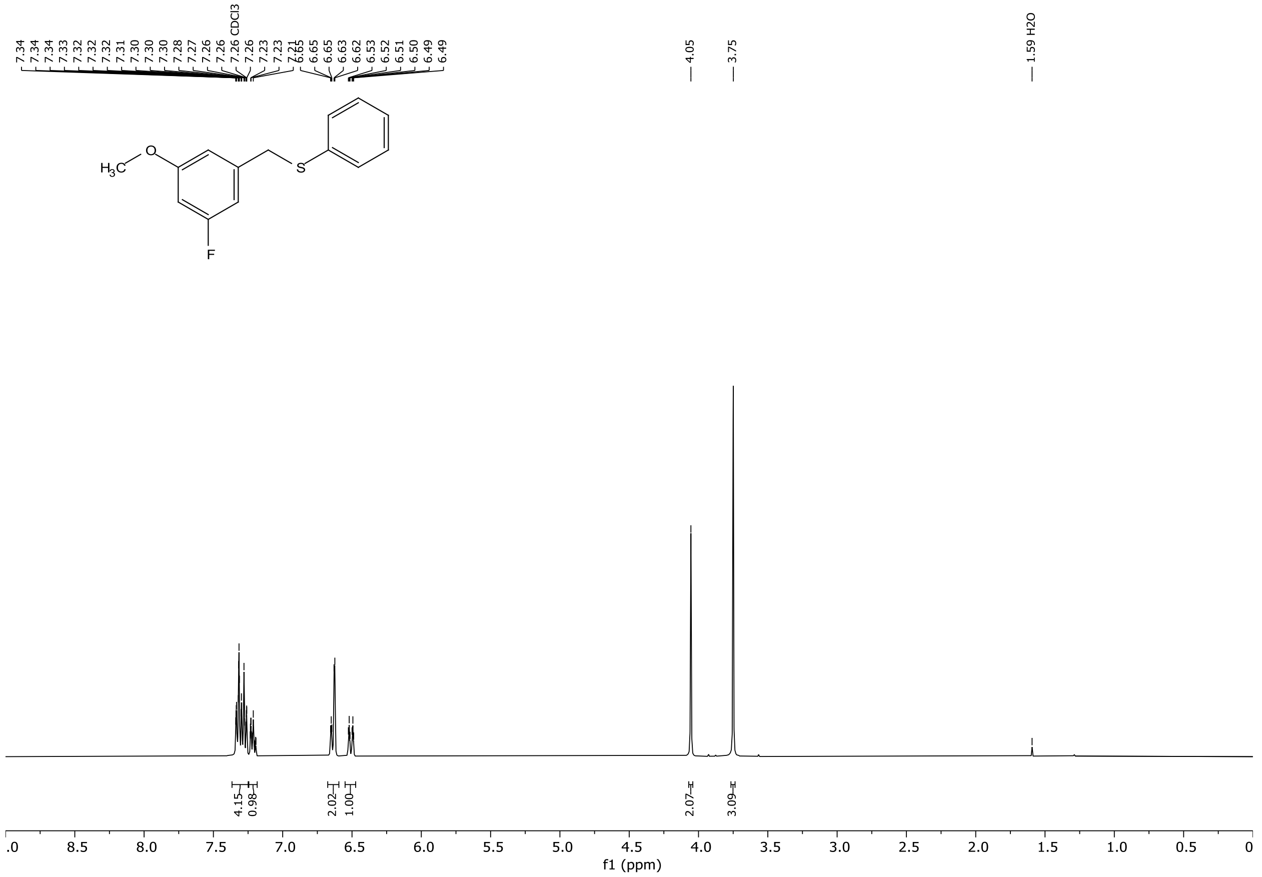

^{13}C NMR (126 MHz, $CDCl_3$)

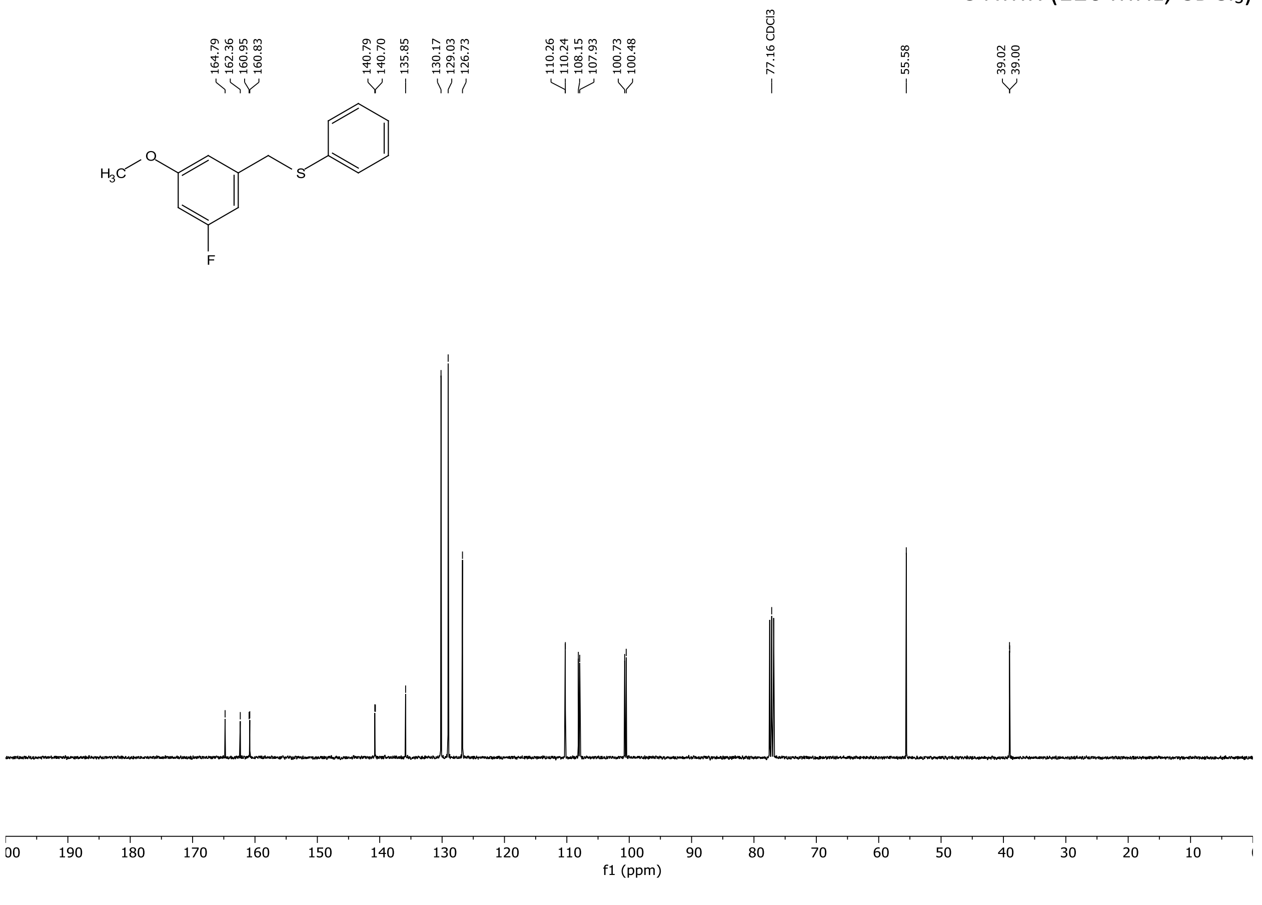

^{19}F NMR (377 MHz, $CDCl_3$)

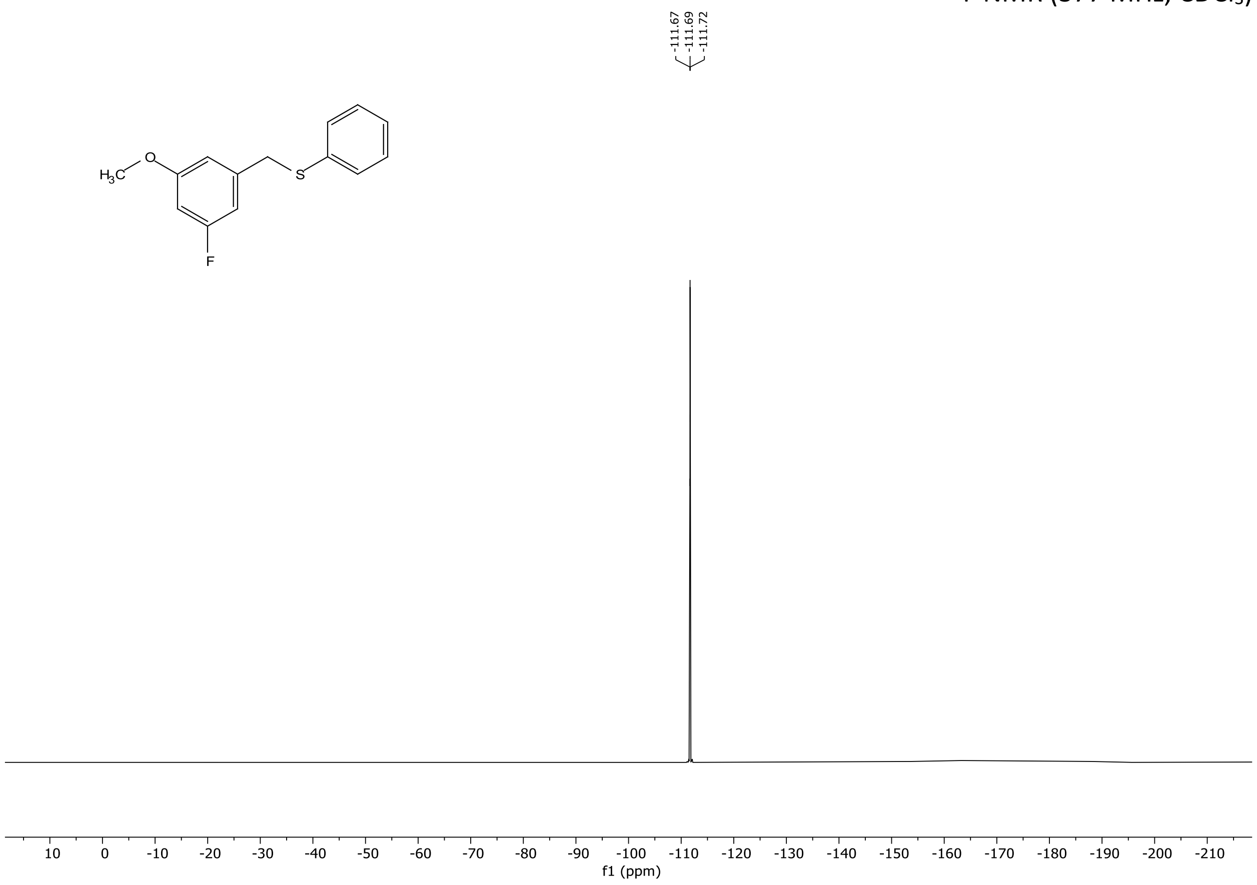

S10.30 **1ac**

^1H NMR (400 MHz, $CDCl_3$)

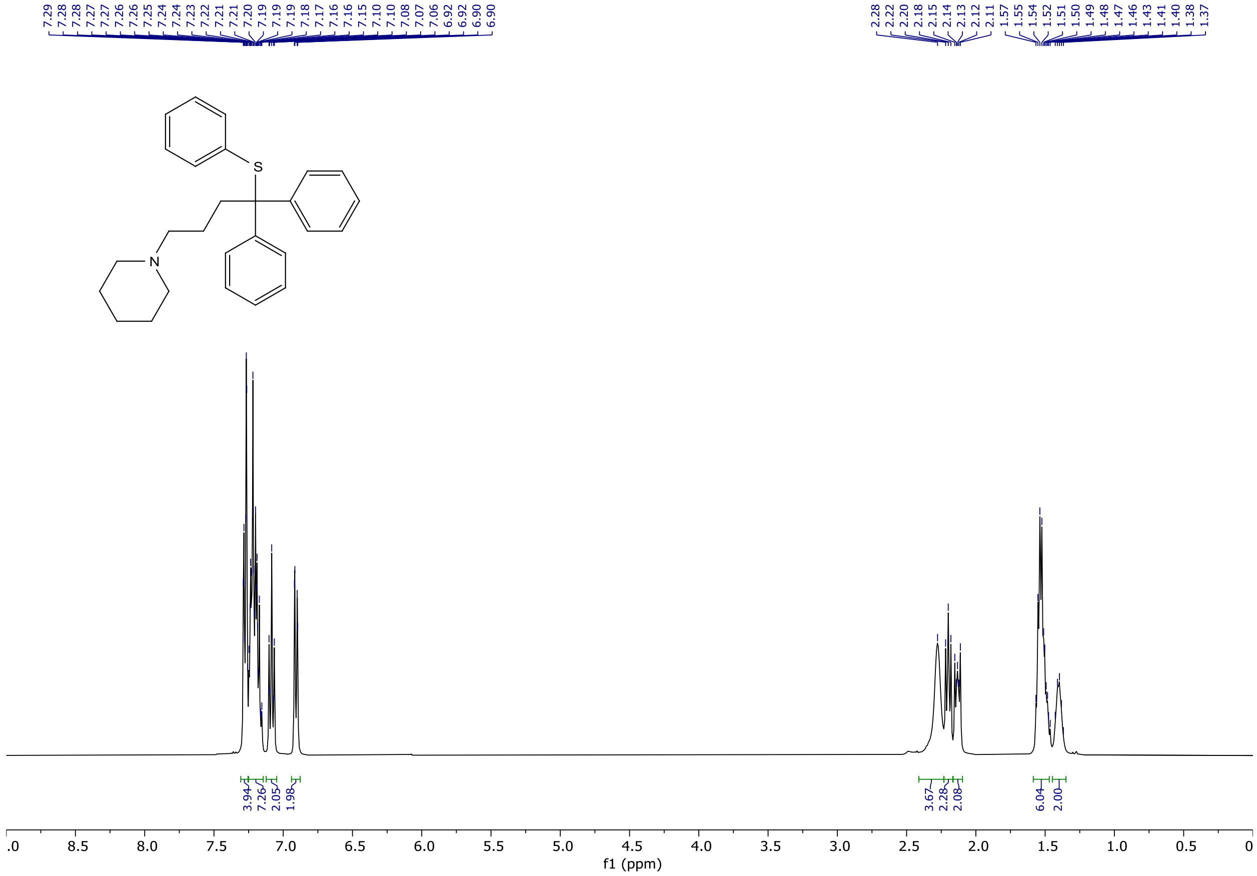

^{13}C NMR (101 MHz, $CDCl_3$)

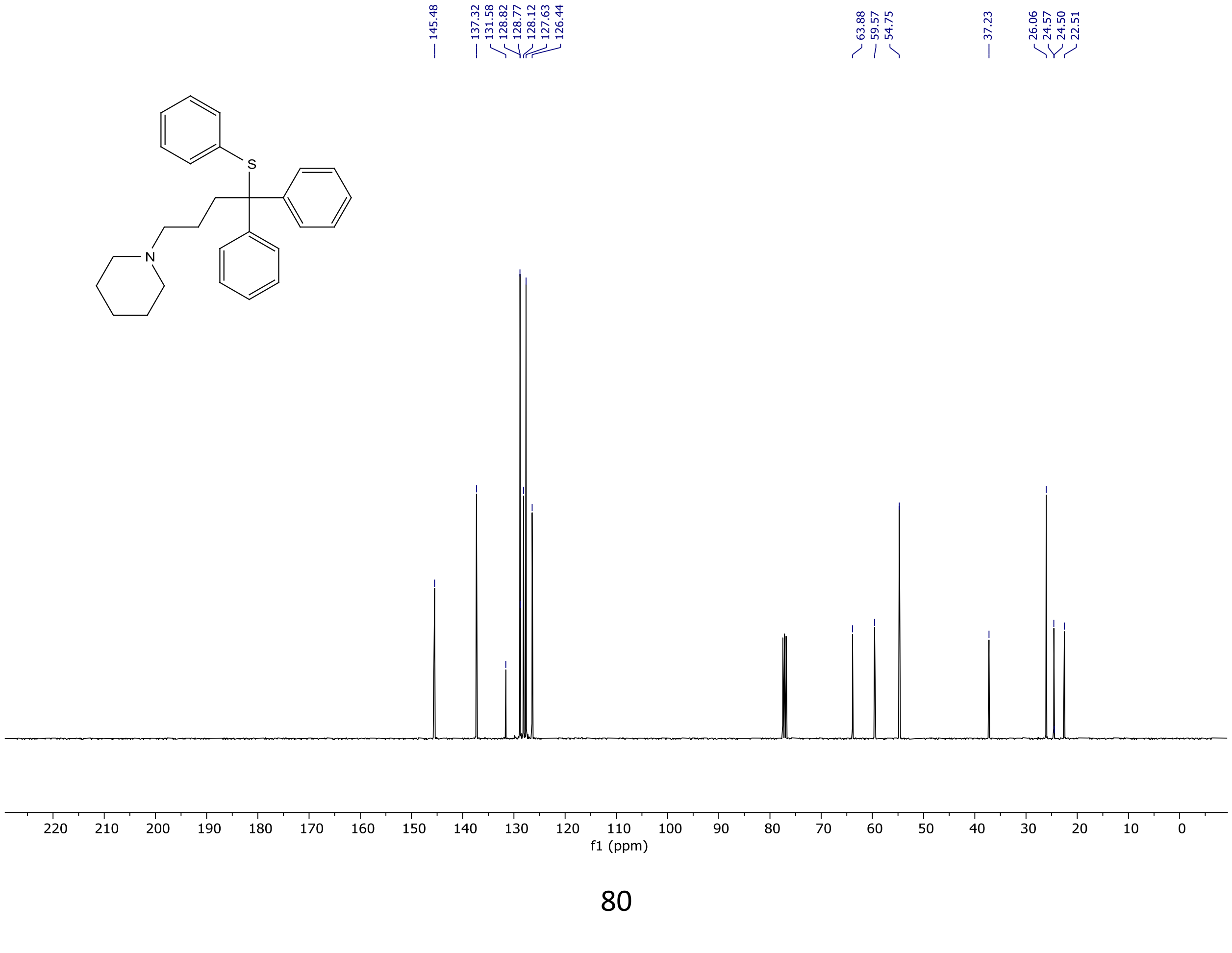

S10.31 1ad

^1H NMR (400 MHz, $CDCl_3$)

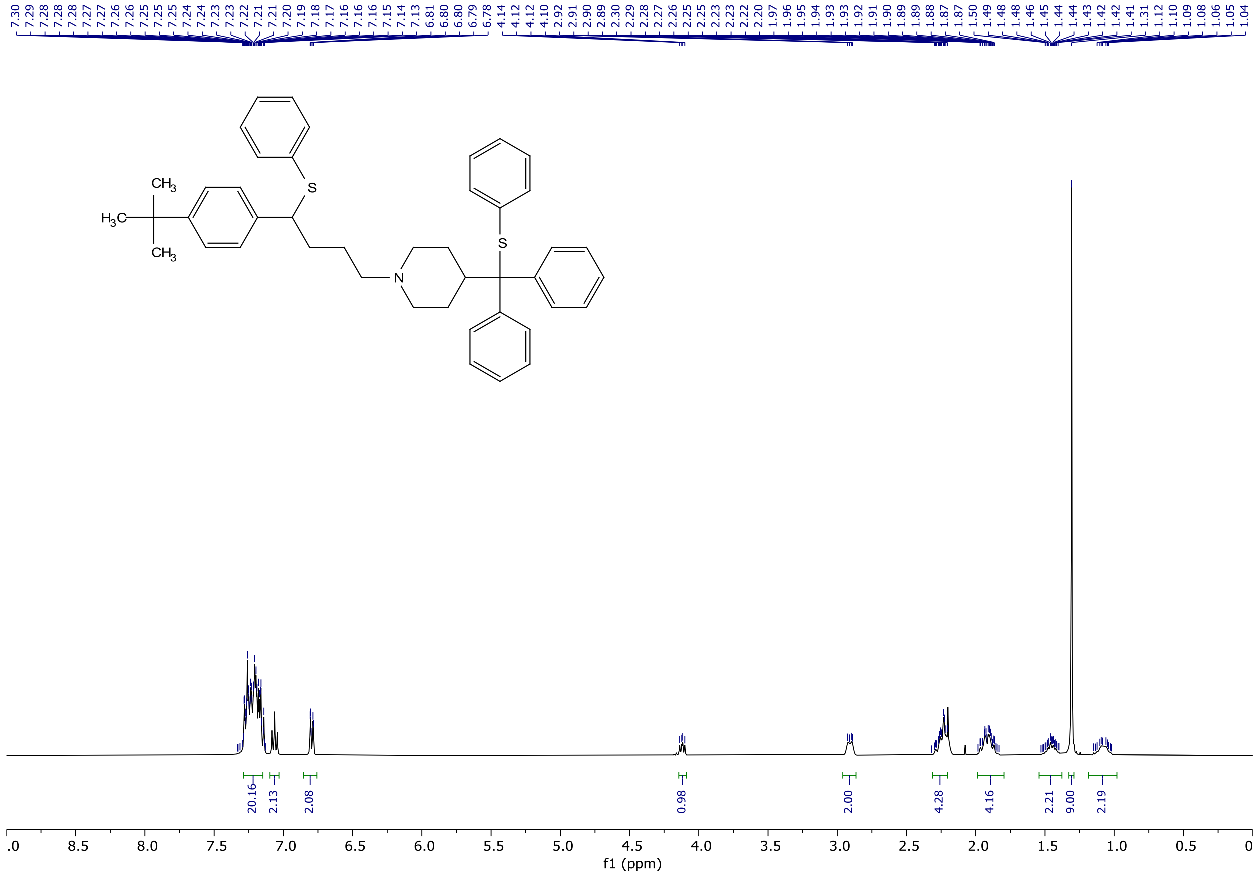

^{13}C NMR (101 MHz, $CDCl_3$)

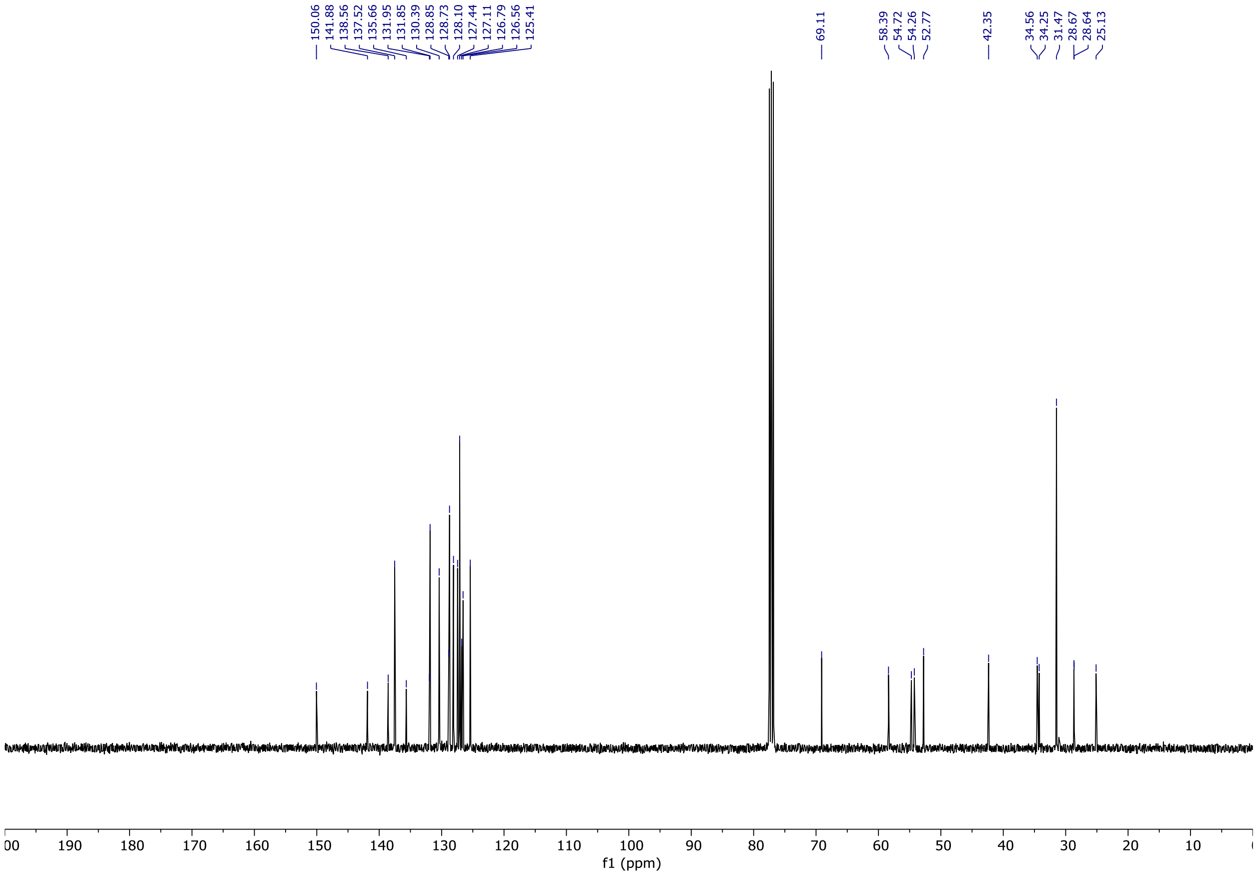

S10.32 1ae

^{1}H NMR (400 MHz, $CDCl_3$)

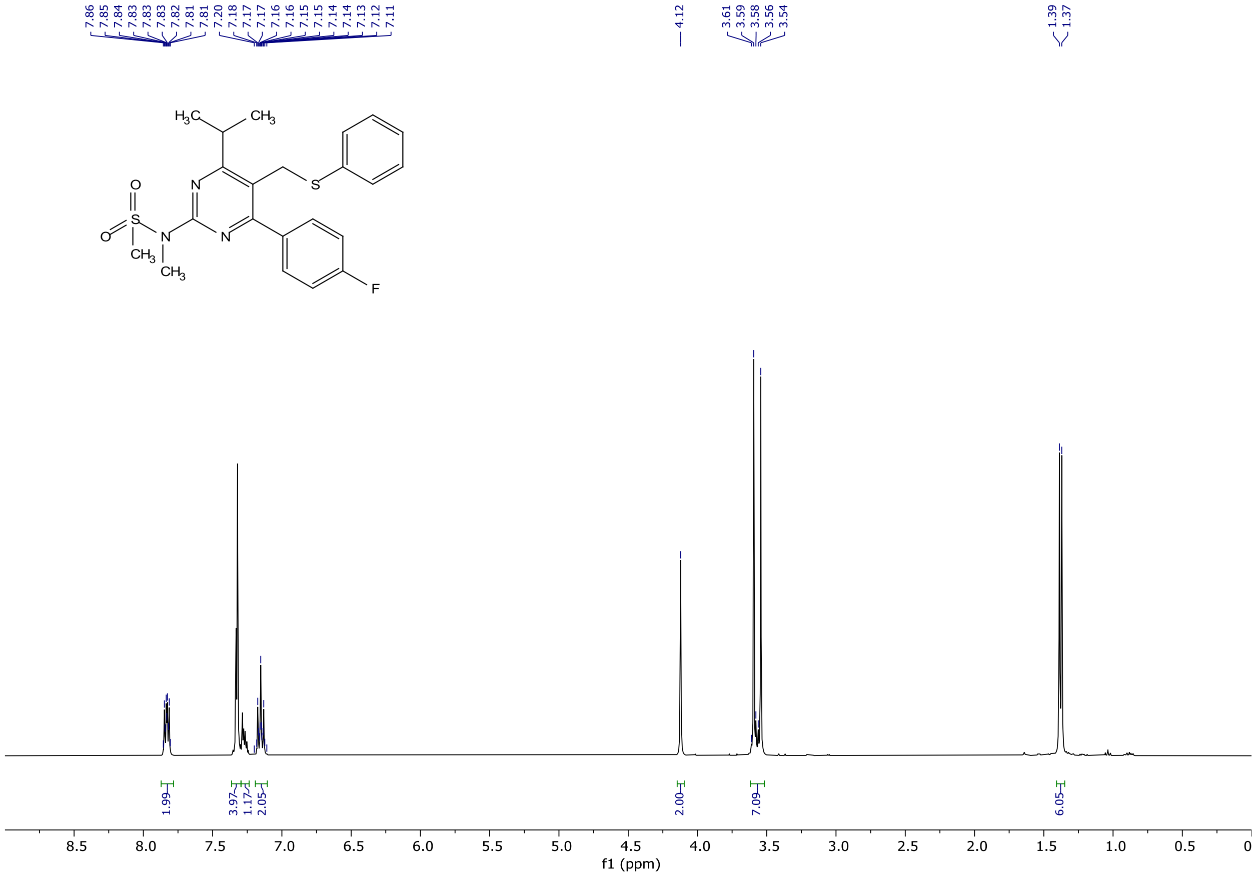

^{13}C NMR (126 MHz, $CDCl_3$)

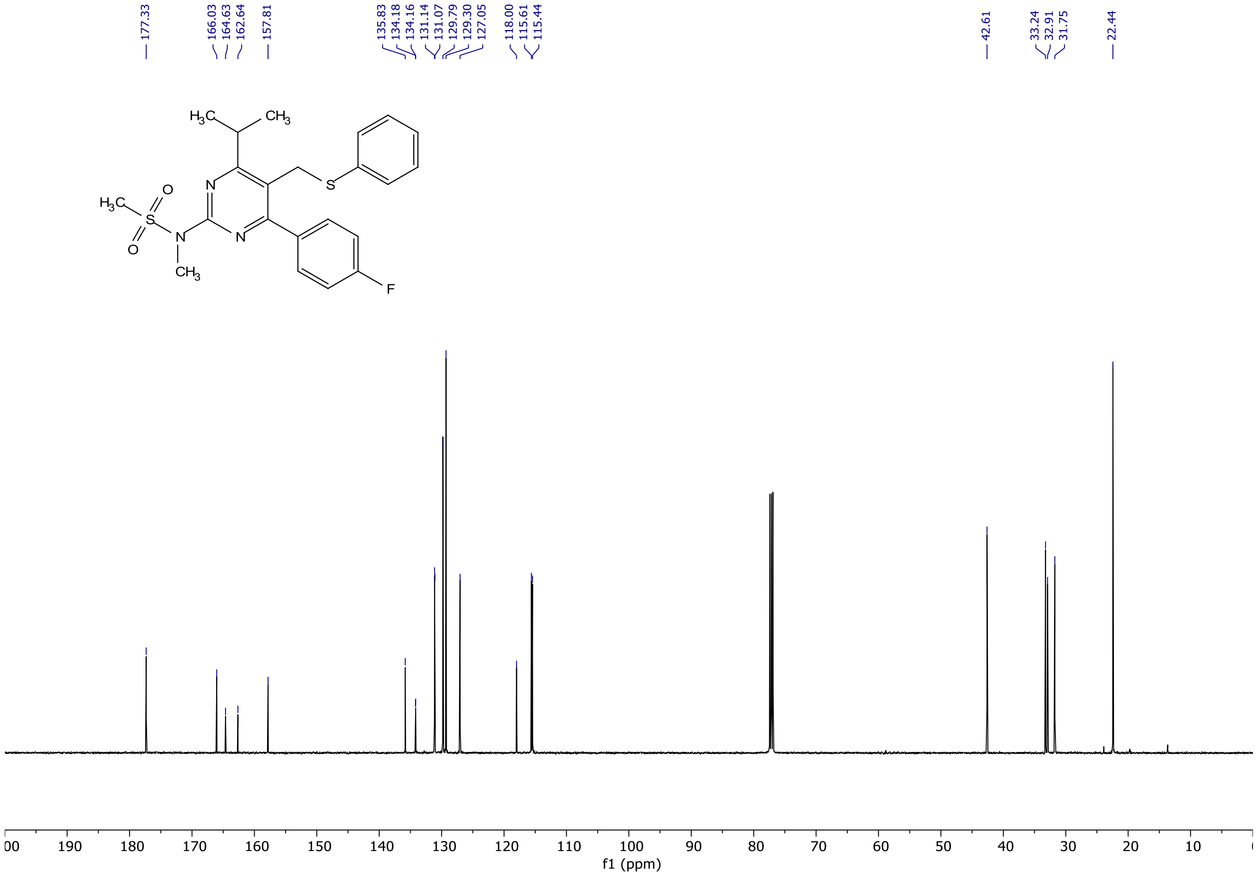

^{19}F NMR (377 MHz, $CDCl_3$)

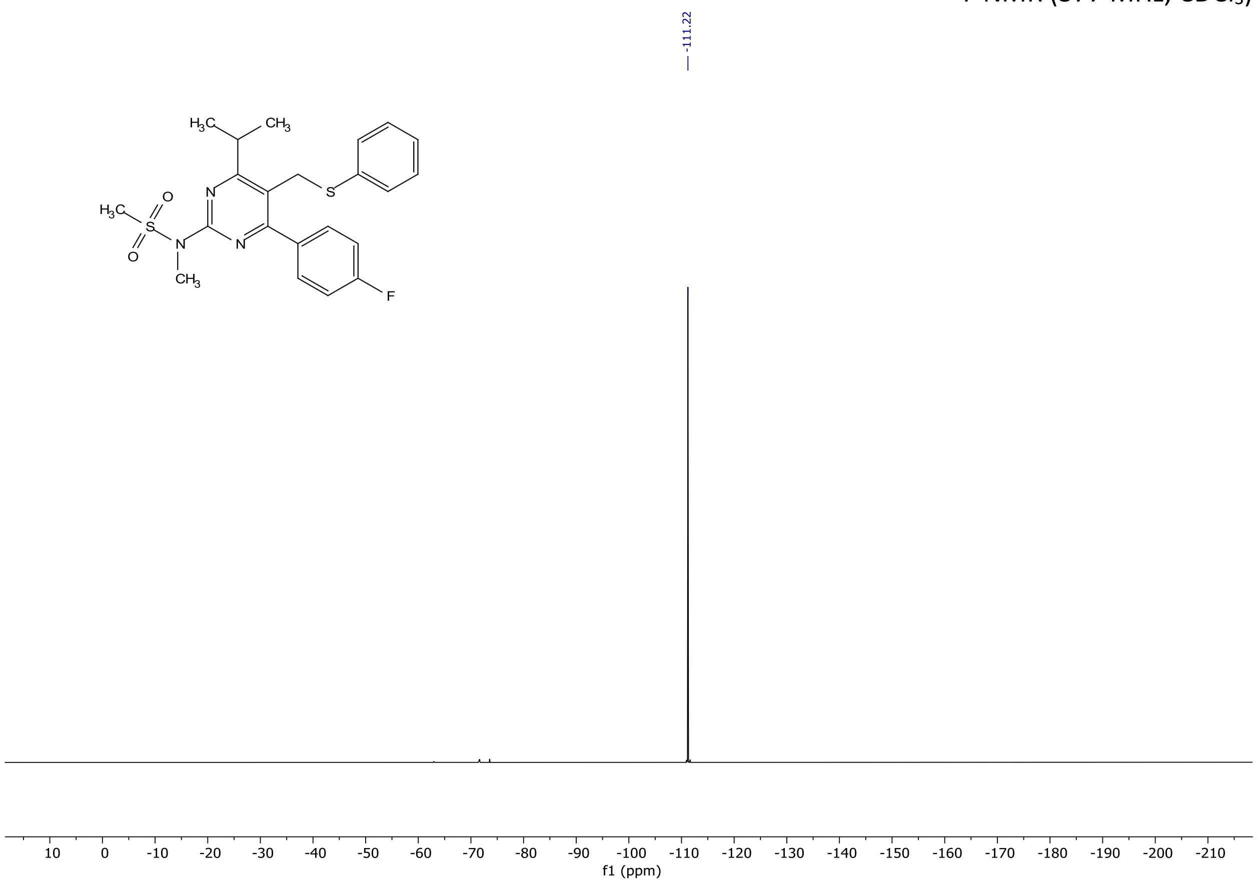

S10.34 1af

^{1}H NMR (400 MHz, $CDCl_3$)

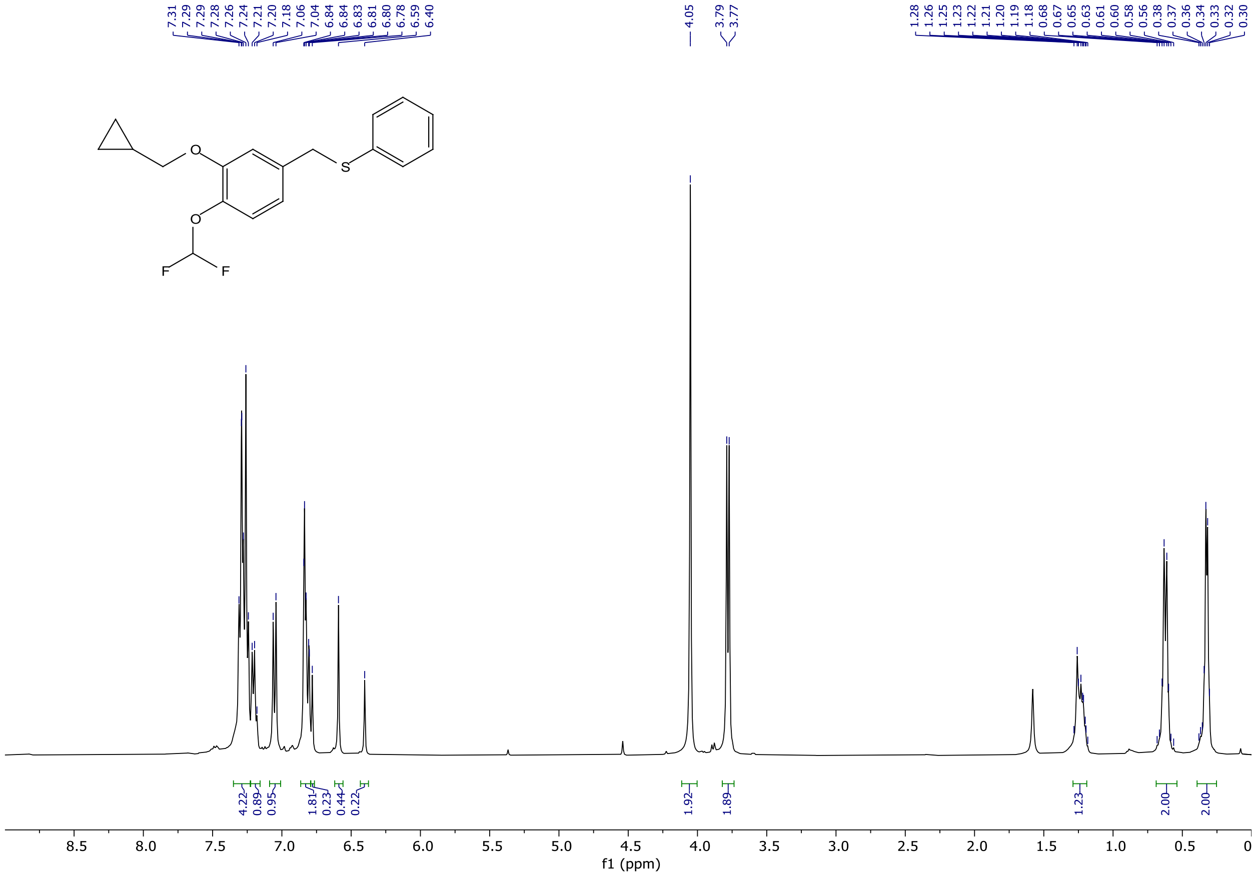

^{13}C NMR (126 MHz, $CDCl_3$)

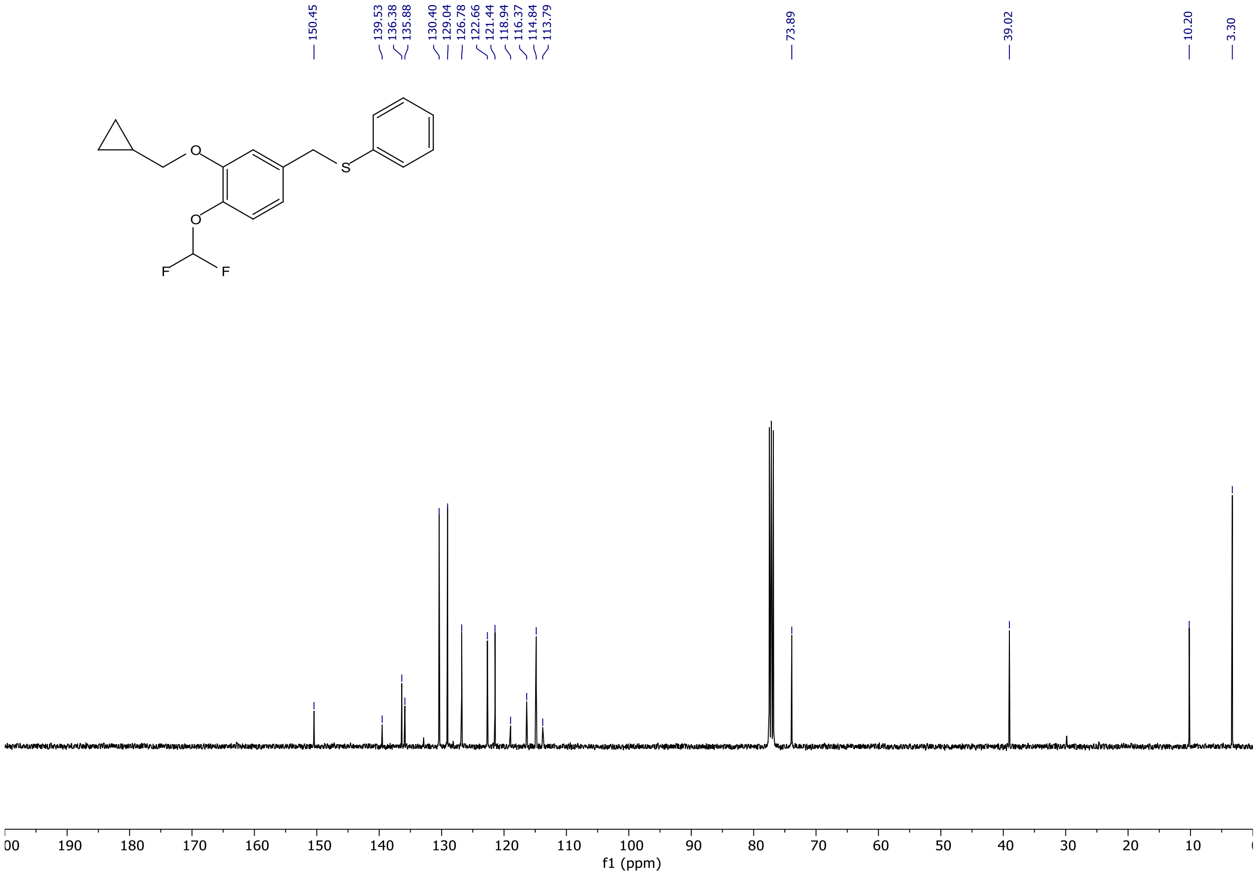

^{19}F NMR (377 MHz, $CDCl_3$)

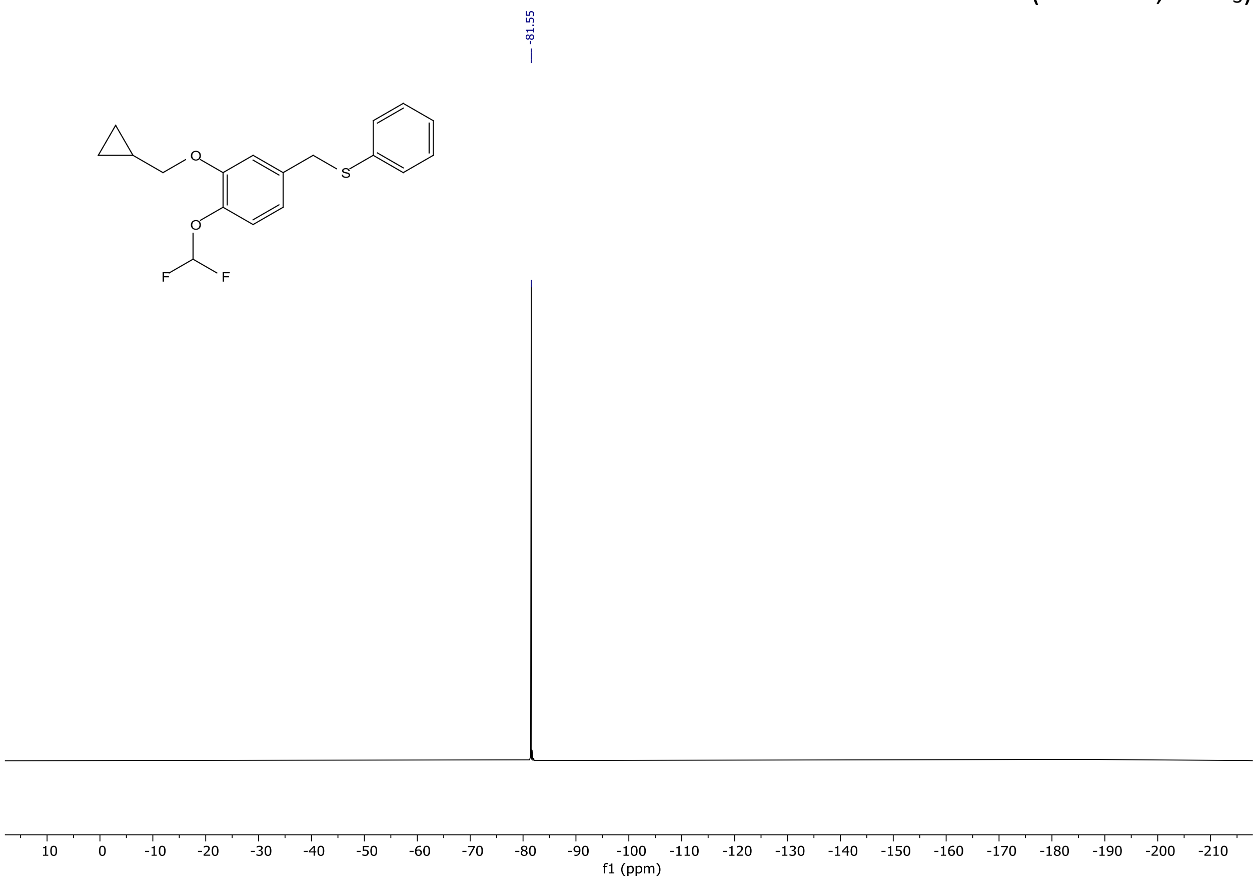

S10.35 1ag

^{1}H NMR (400 MHz, $CDCl_3$)

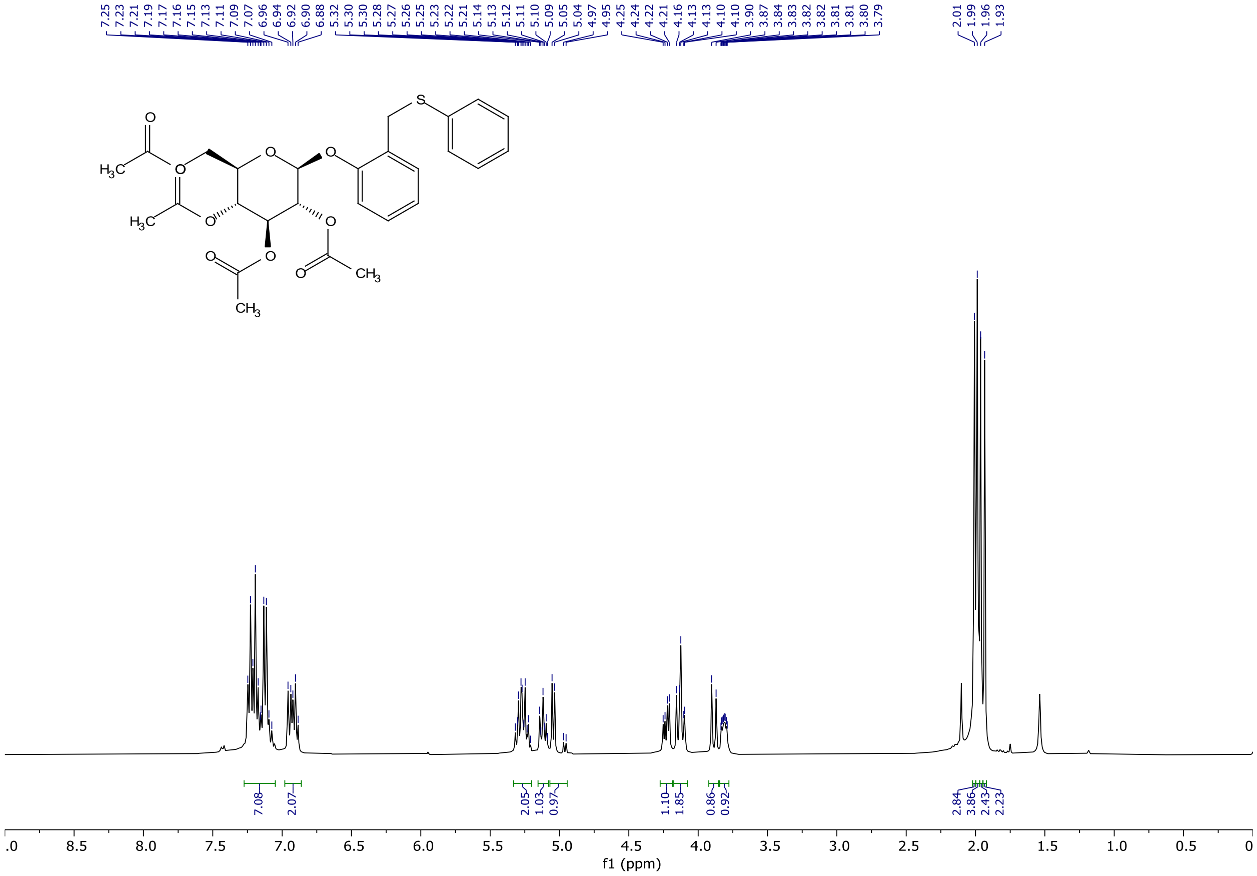

^{13}C NMR (101 MHz, $CDCl_3$)

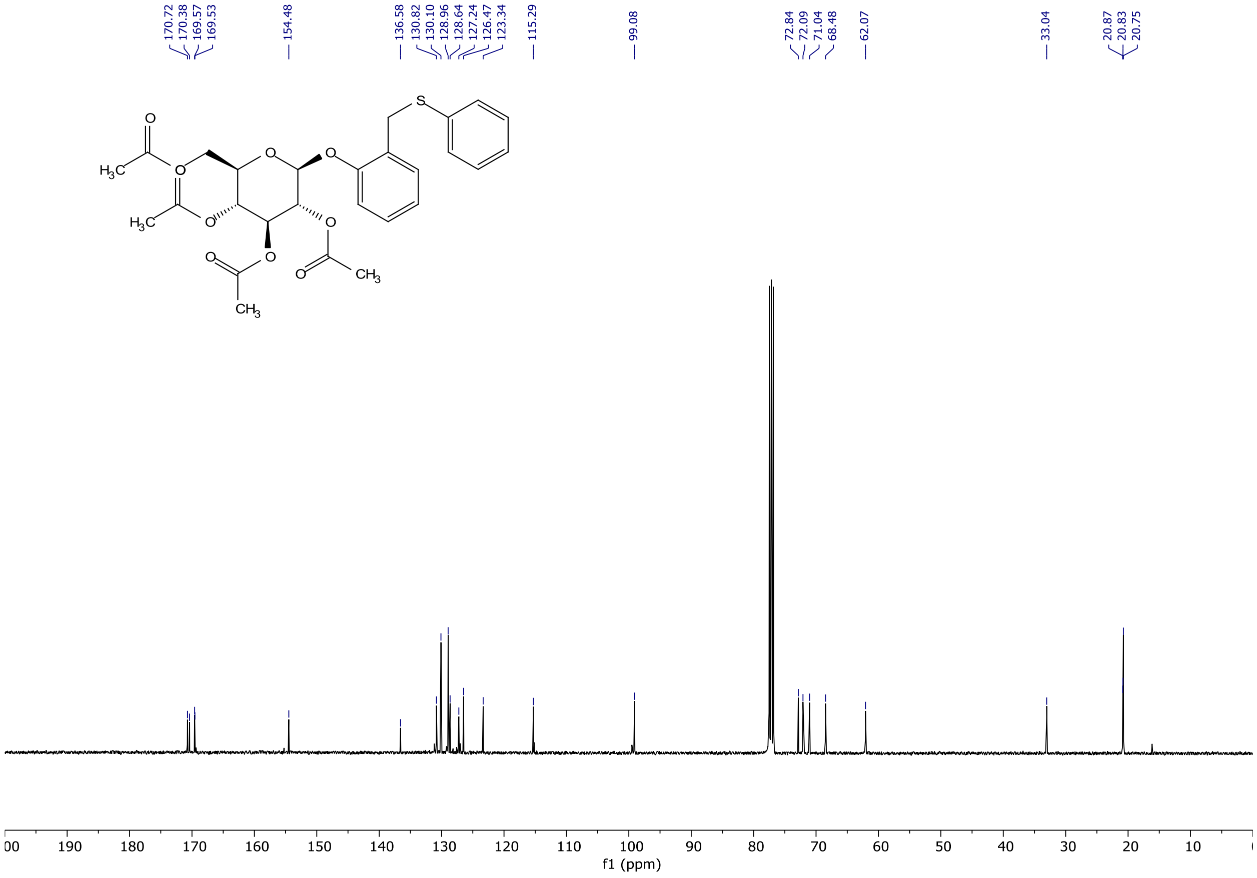

S10.36 1ah

^{1}H NMR (400 MHz, $CDCl_3$)

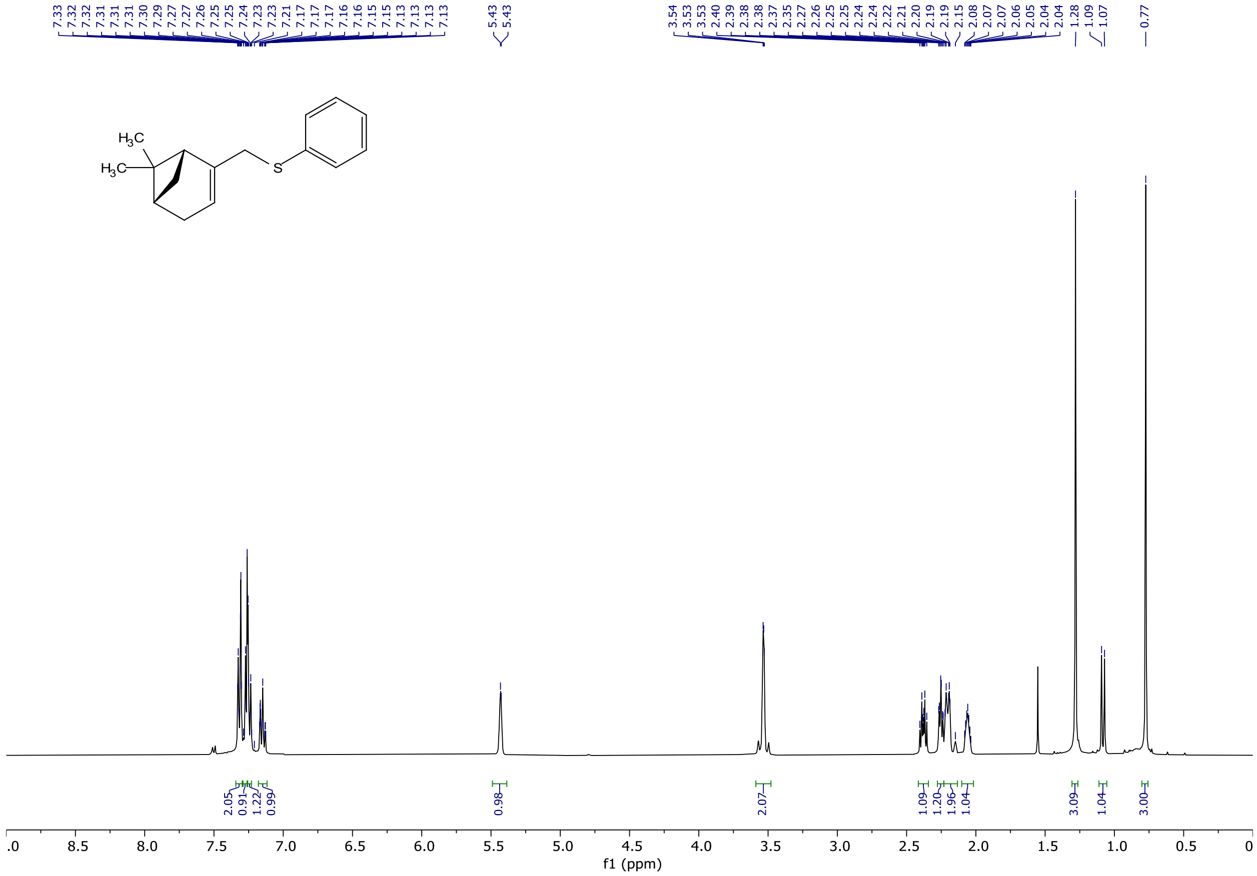

^{13}C NMR (101 MHz, $CDCl_3$)

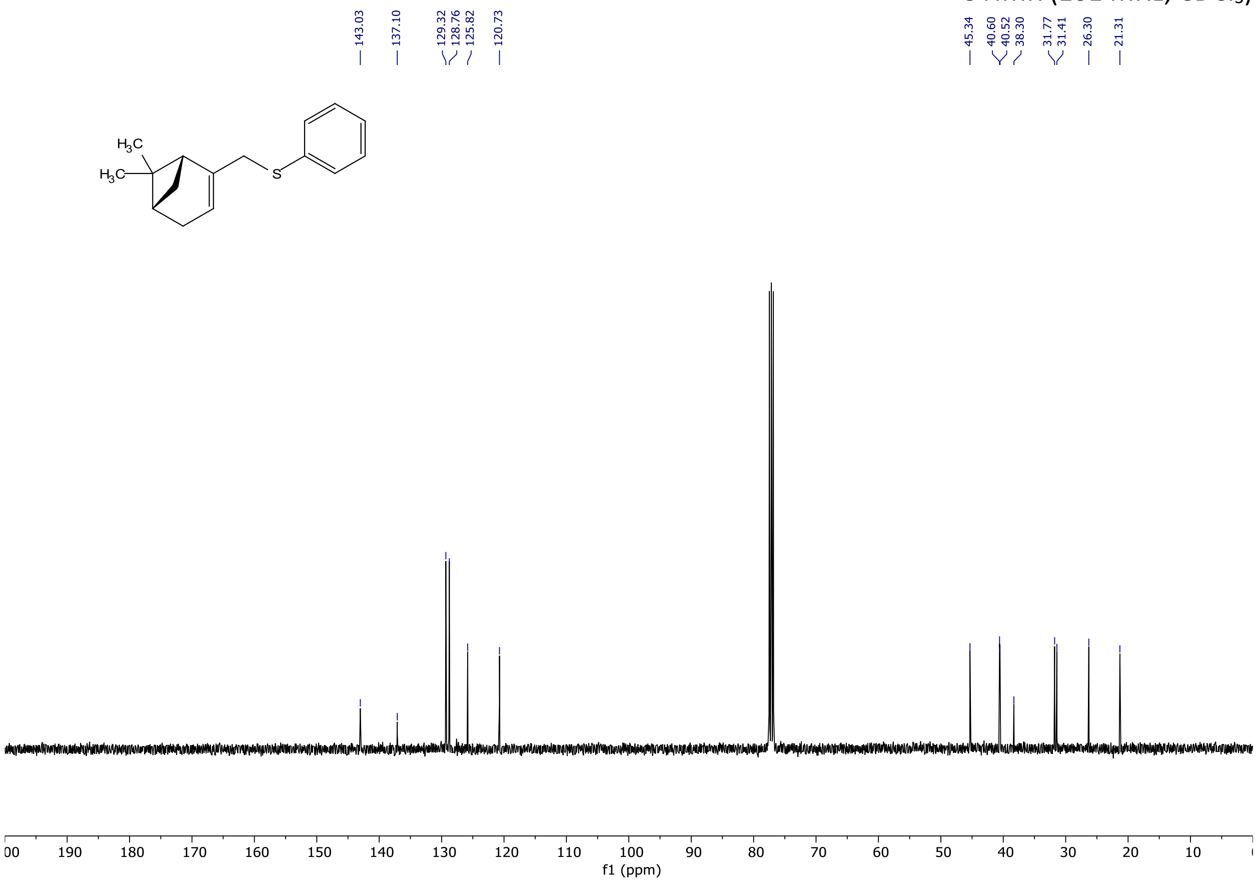

S10.37 1ai

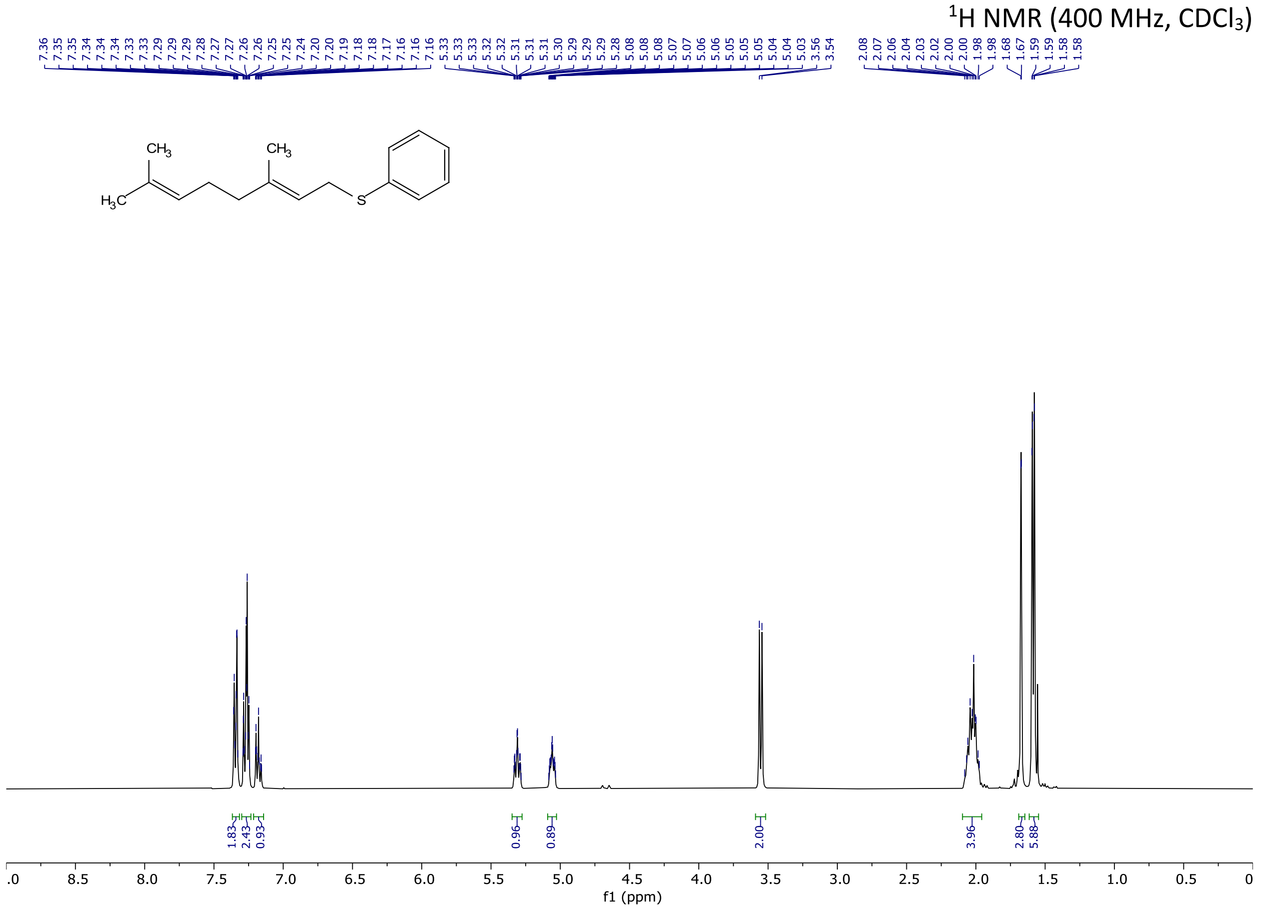

^{1}H NMR (400 MHz, CDCl$_3$)
f1 (ppm)

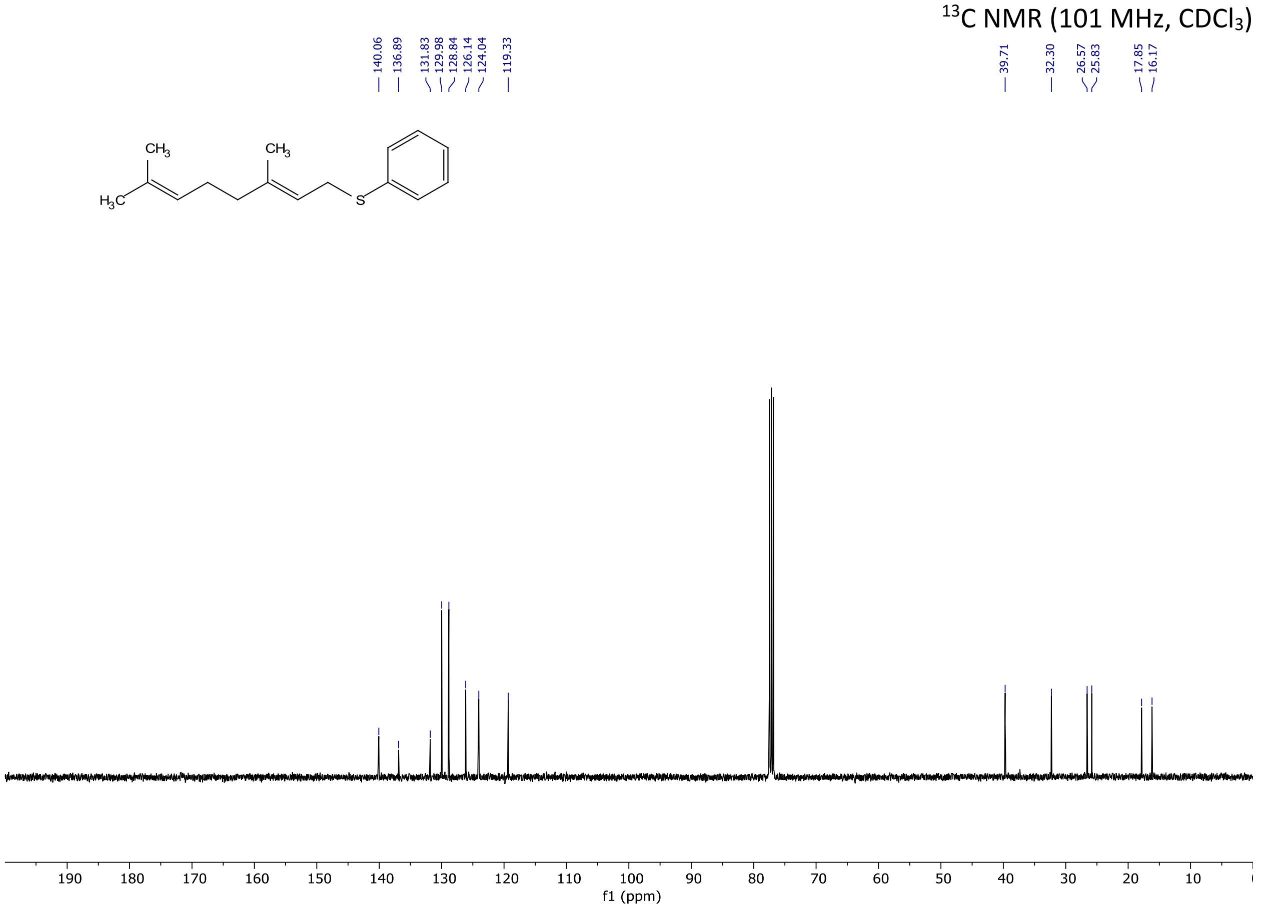

^{13}C NMR (101 MHz, CDCl$_3$)
140.06
136.89
131.83
129.98
128.84
126.14
124.04
119.33
39.71
32.30
26.57
25.83
17.85
16.17
f1 (ppm)

S10.38 1aj

^{1}H NMR (400 MHz, $CDCl_3$)

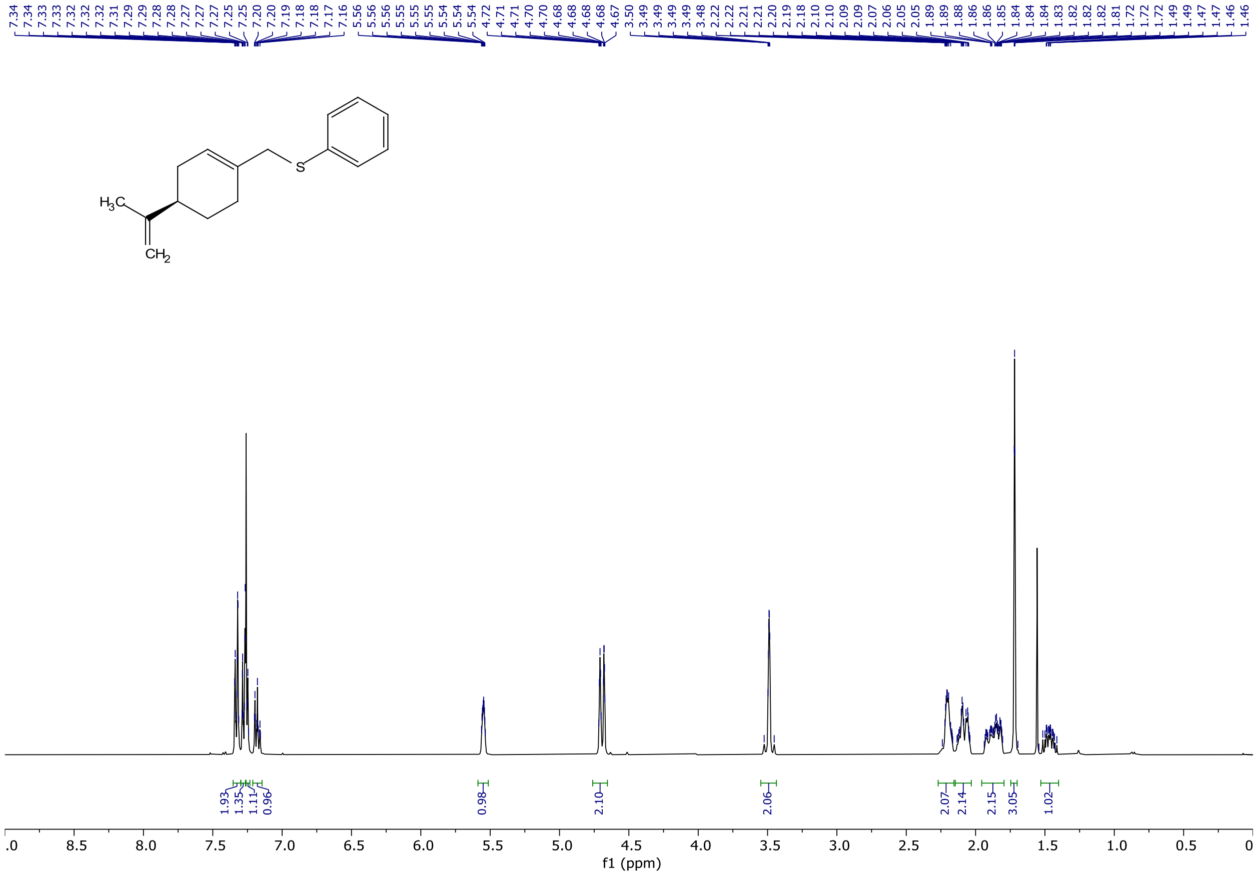

^{13}C NMR (101 MHz, $CDCl_3$)

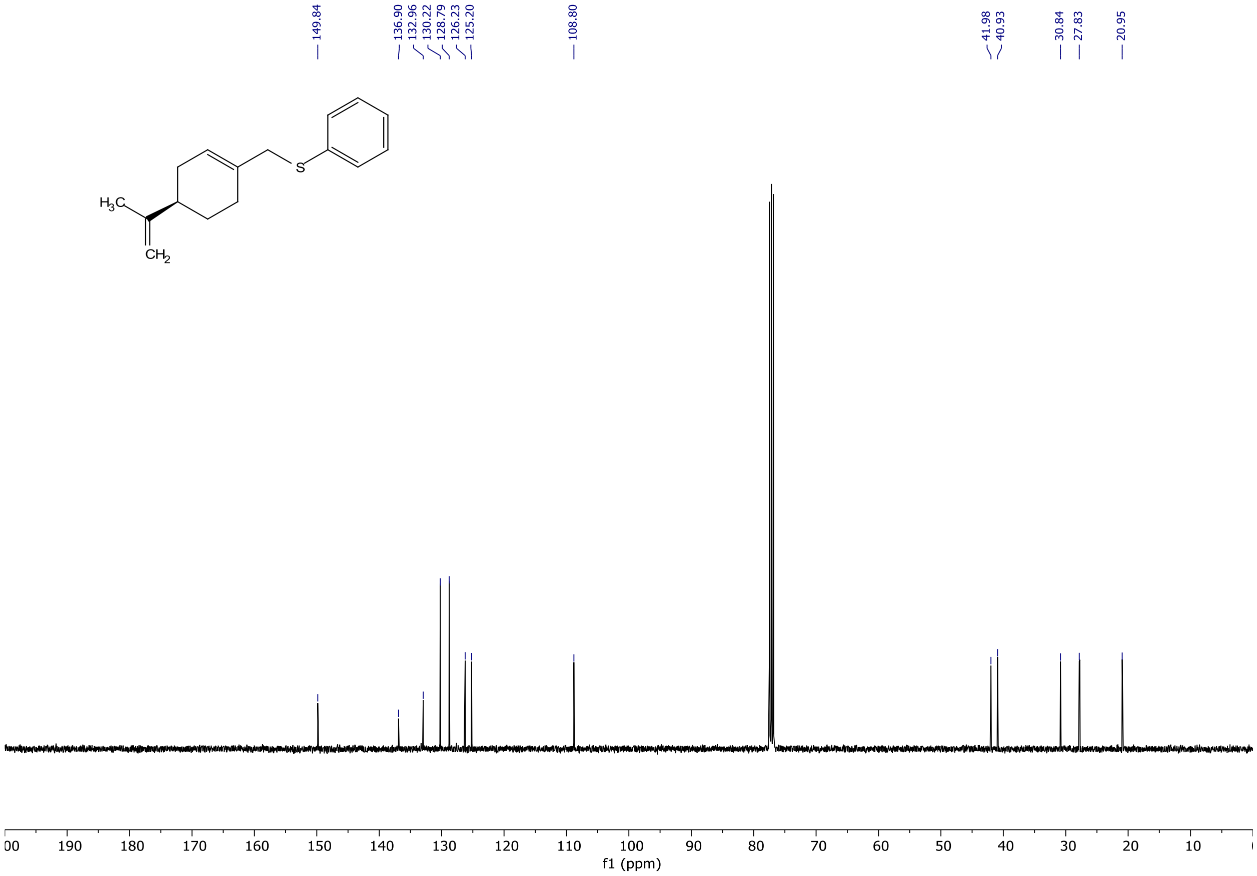

S10.39 1ak

^{1}H NMR (400 MHz, $CDCl_3$)

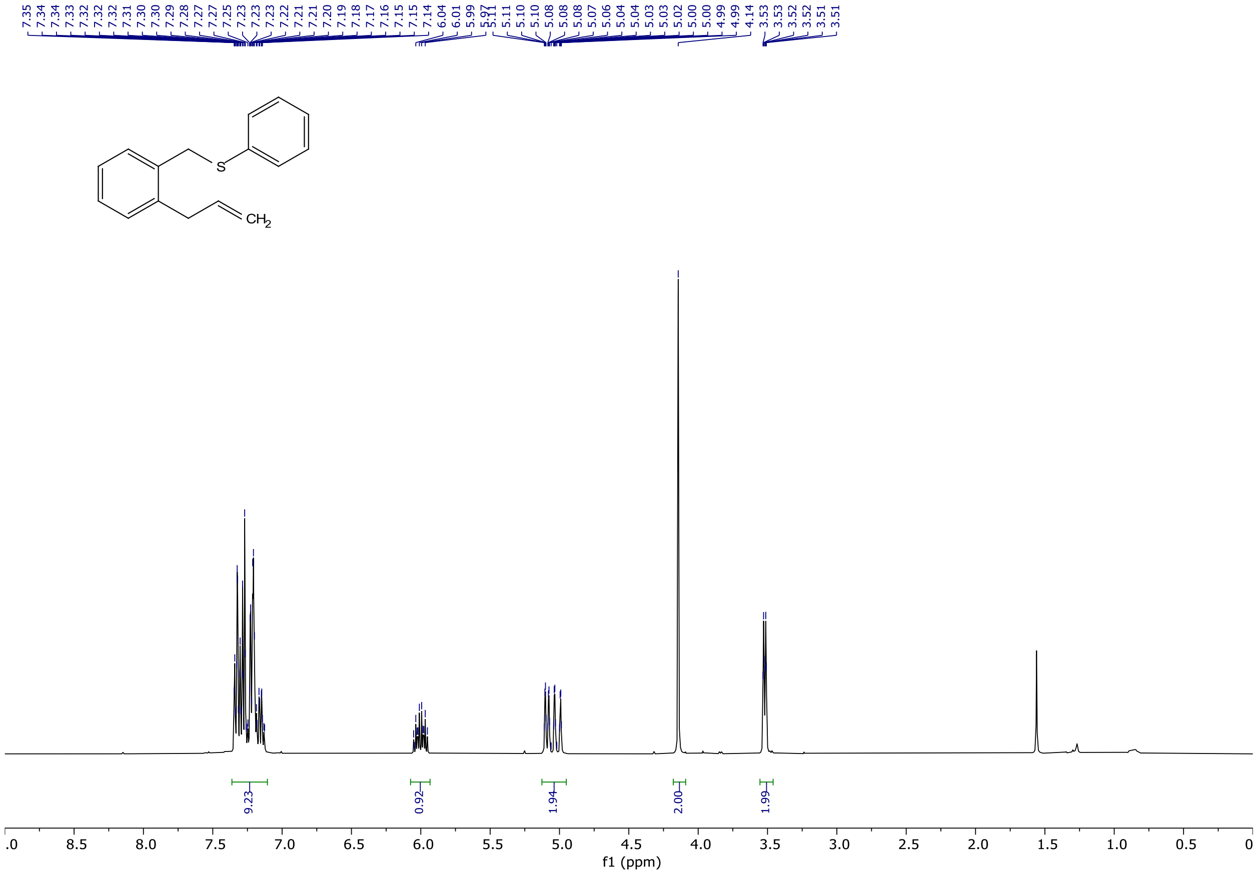

^{13}C NMR (101 MHz, $CDCl_3$)

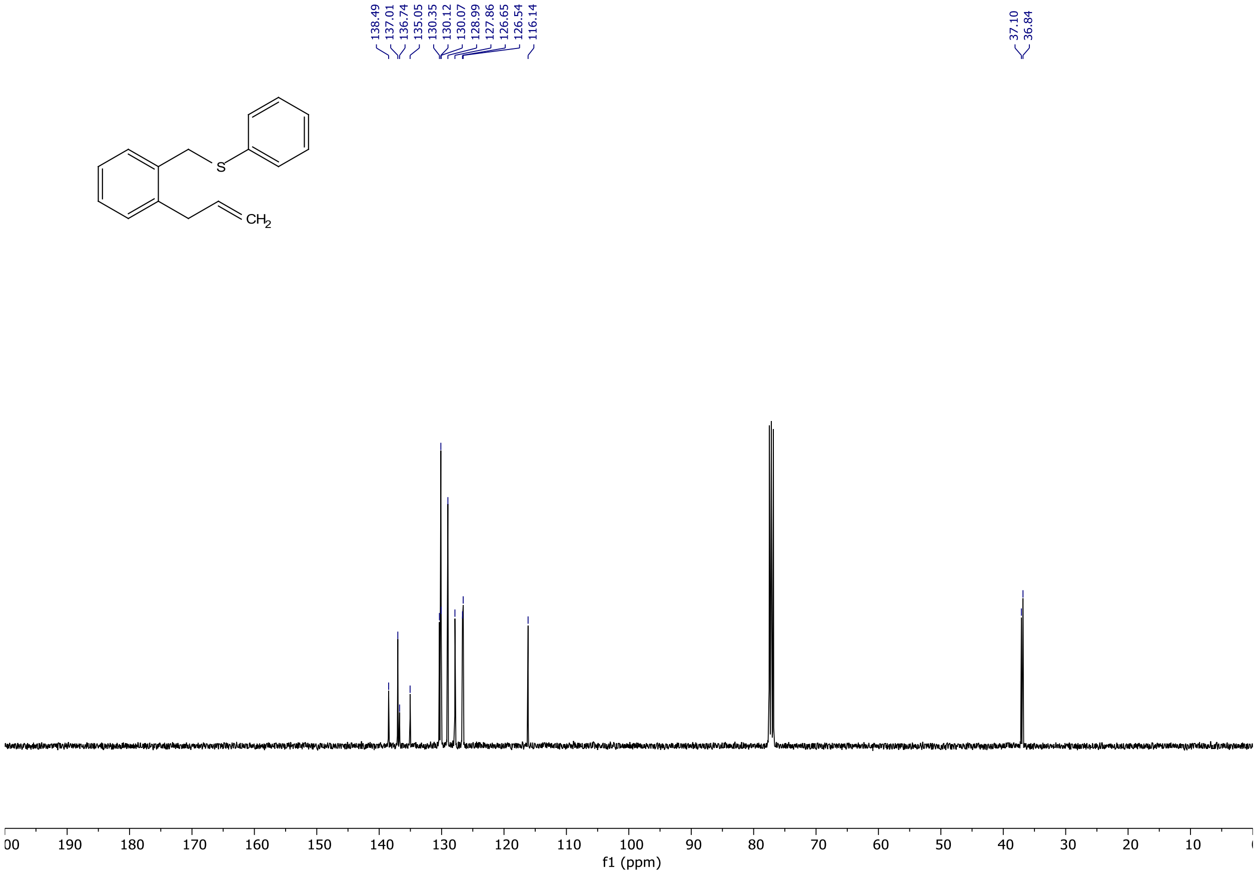

S10.40 1al

^1H NMR (400 MHz, $CDCl_3$)

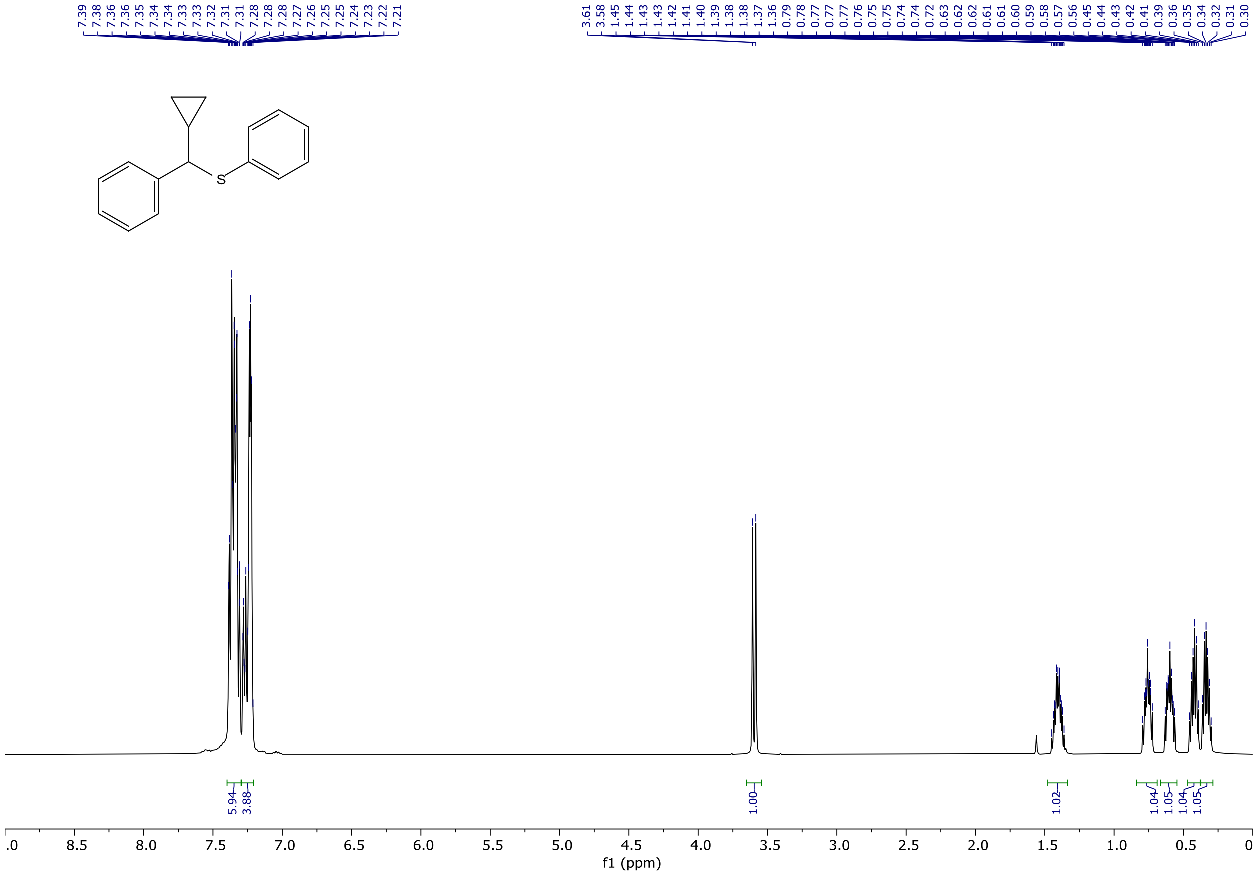

^{13}C NMR (101 MHz, $CDCl_3$)

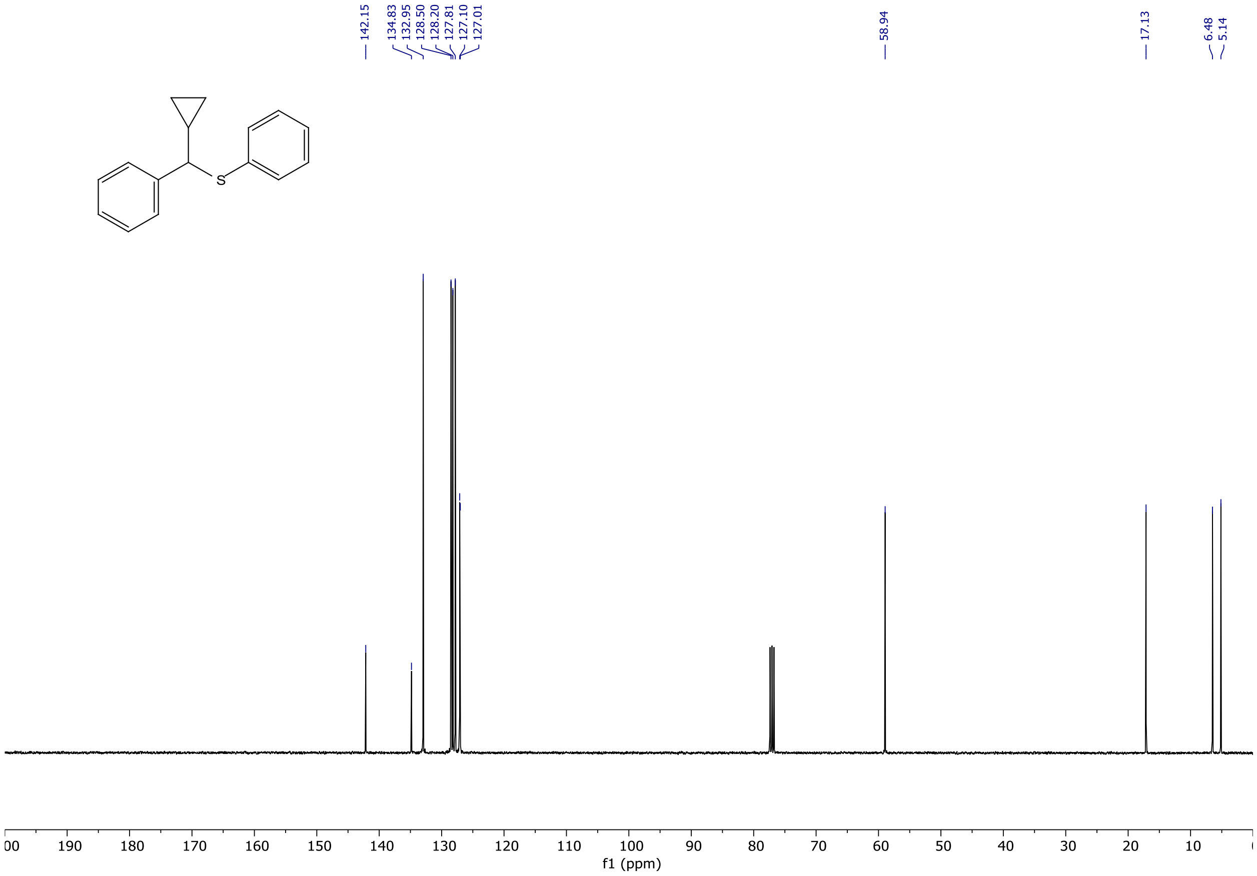

S10.41 **2a**

^1H NMR (400 MHz, $CDCl_3$)

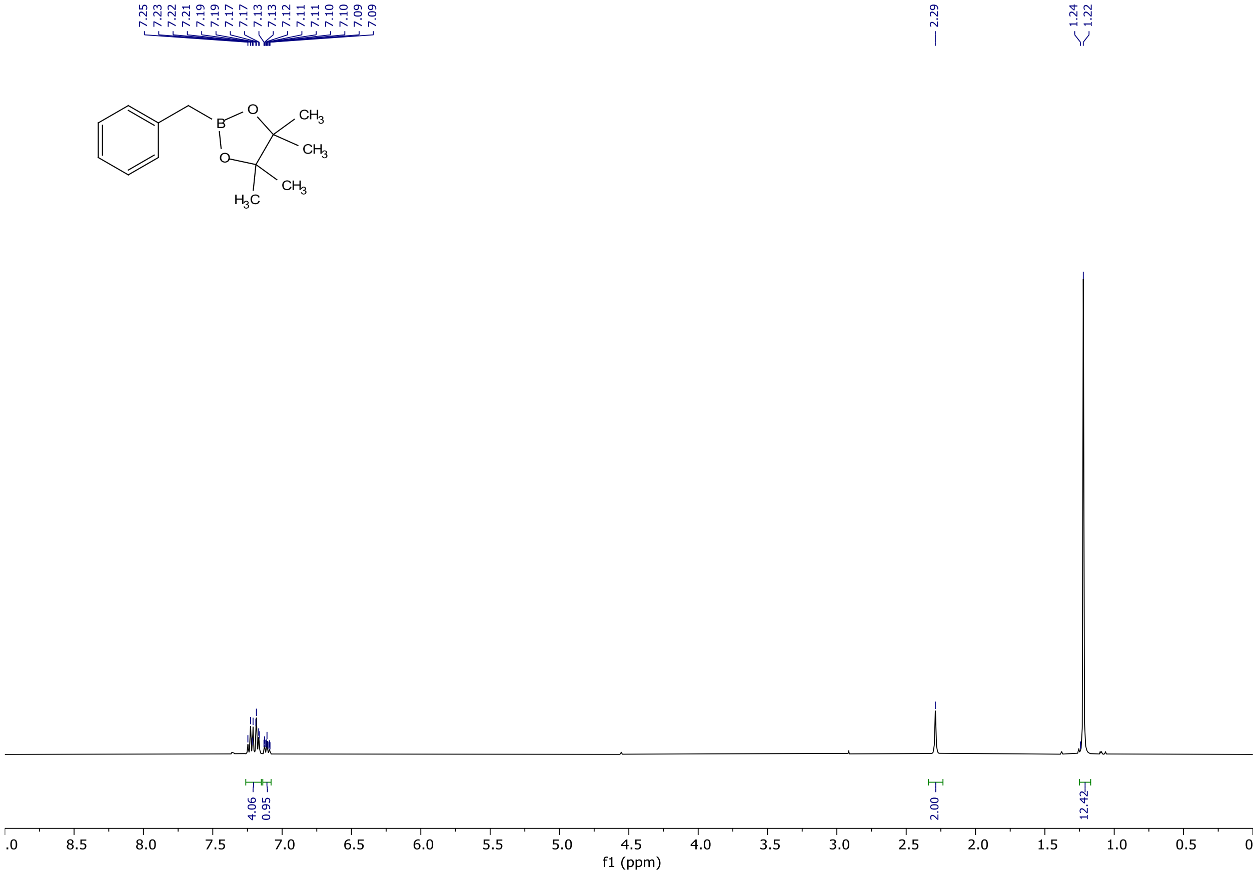

^{13}C NMR (101 MHz, $CDCl_3$)

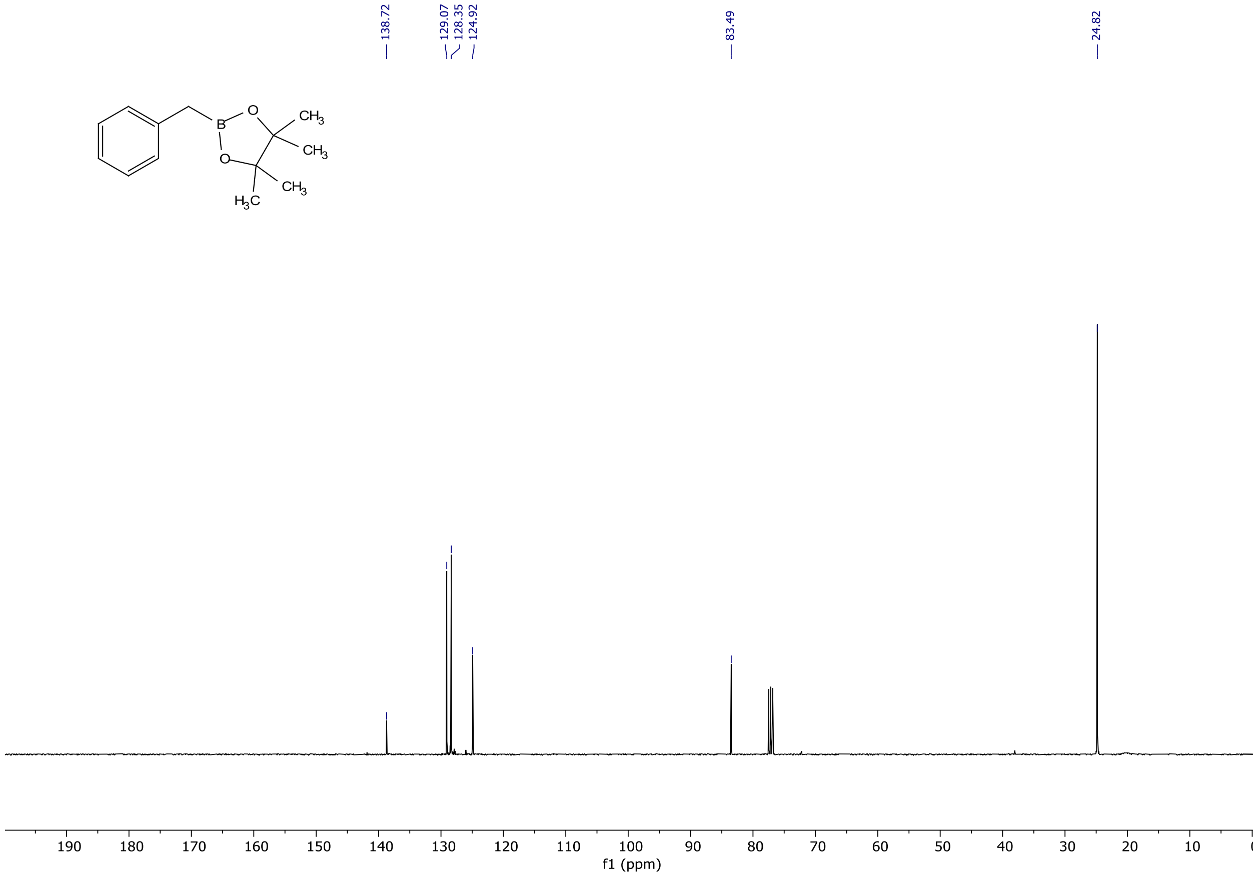

^{11}B NMR (128 MHz, $CDCl_3$)

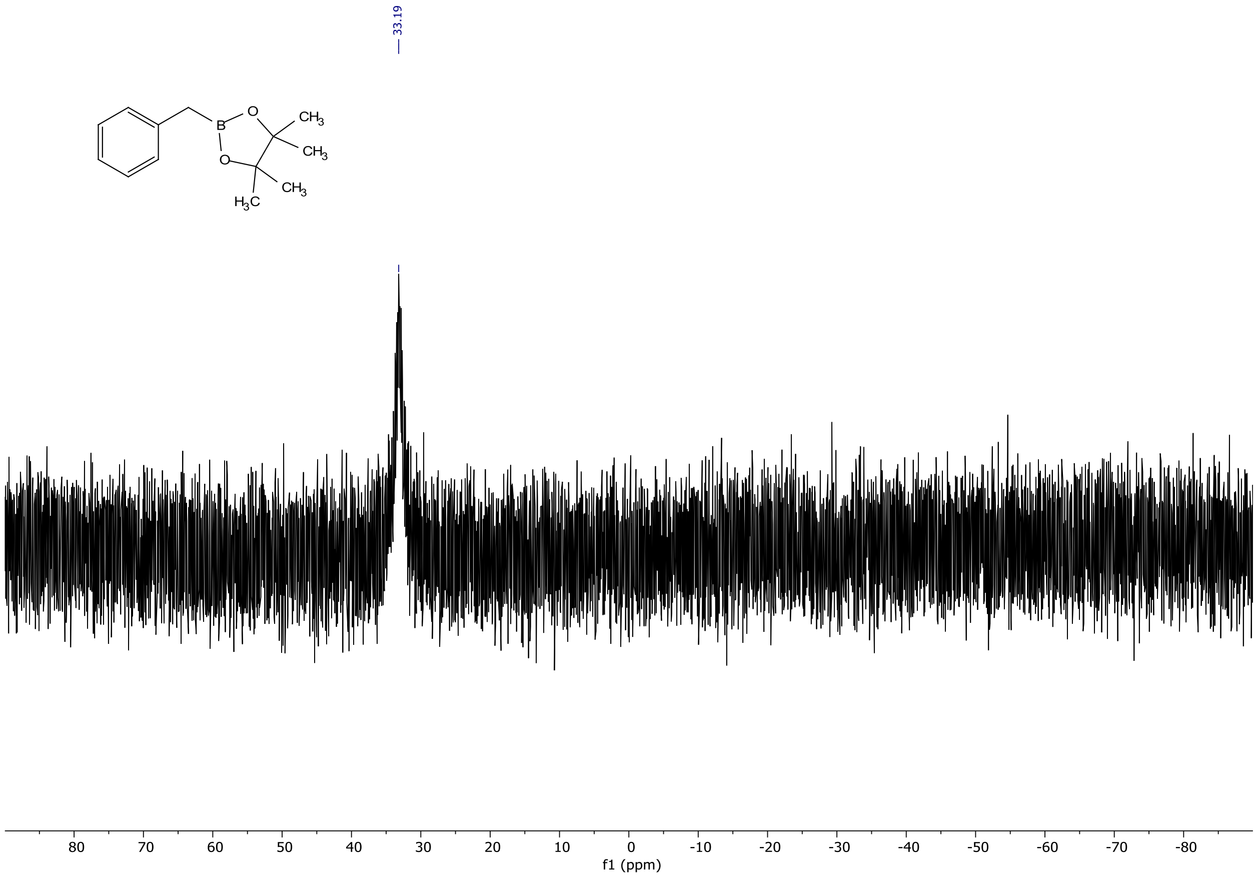

S10.42 **2b**

^{1}H NMR (400 MHz, $CDCl_3$)

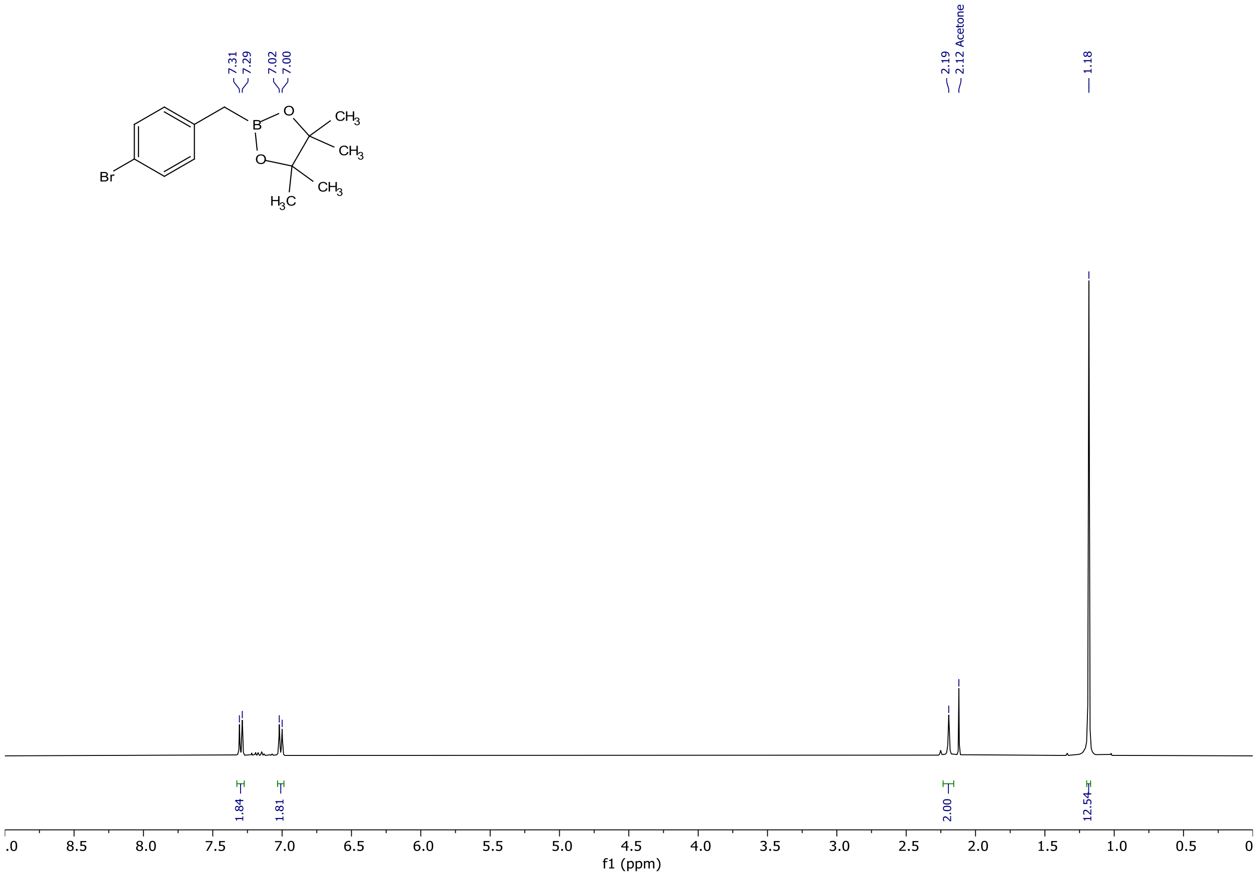

^{13}C NMR (101 MHz, $CDCl_3$)

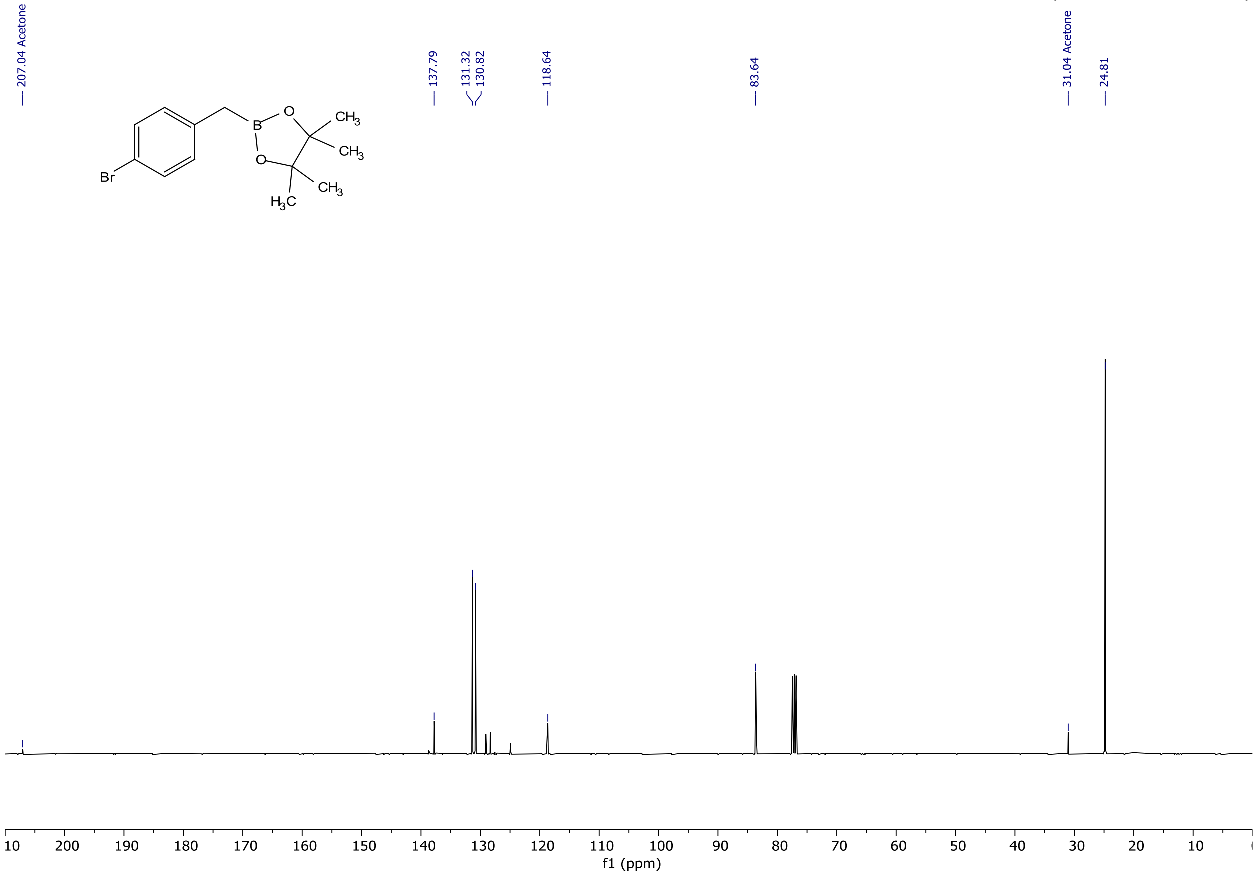

^{11}B NMR (128 MHz, $CDCl_3$)

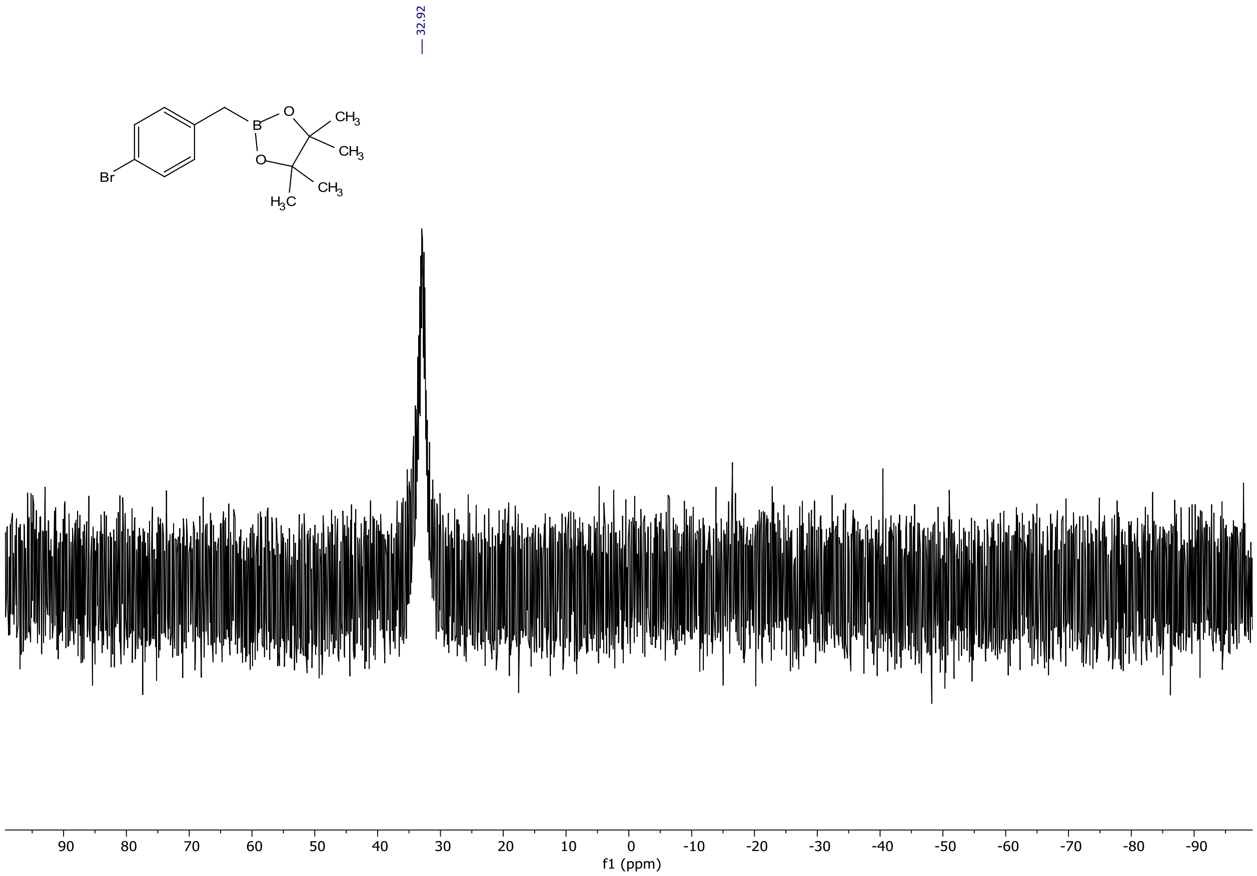

S10.43 2c

^{1}H NMR (400 MHz, $CDCl_3$)

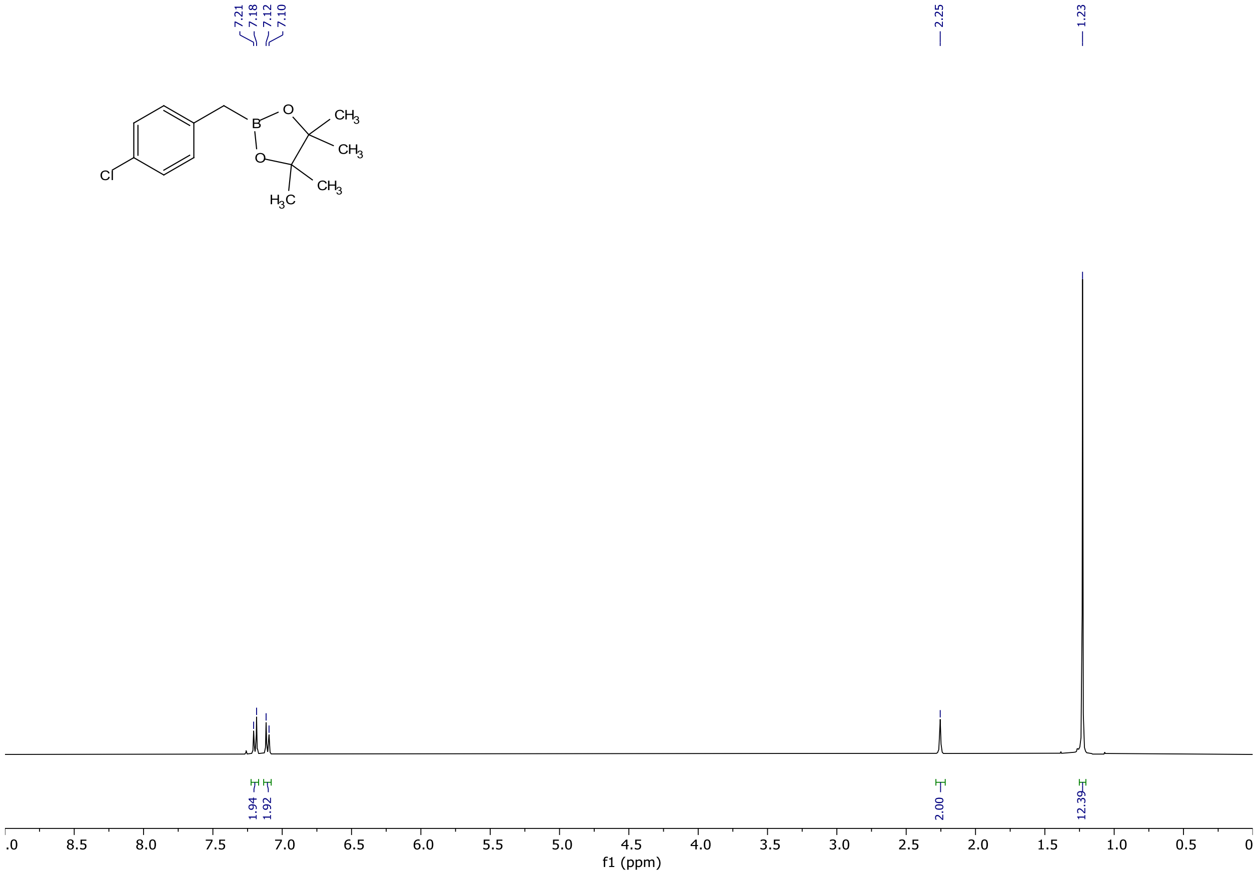

^{13}C NMR (101 MHz, $CDCl_3$)

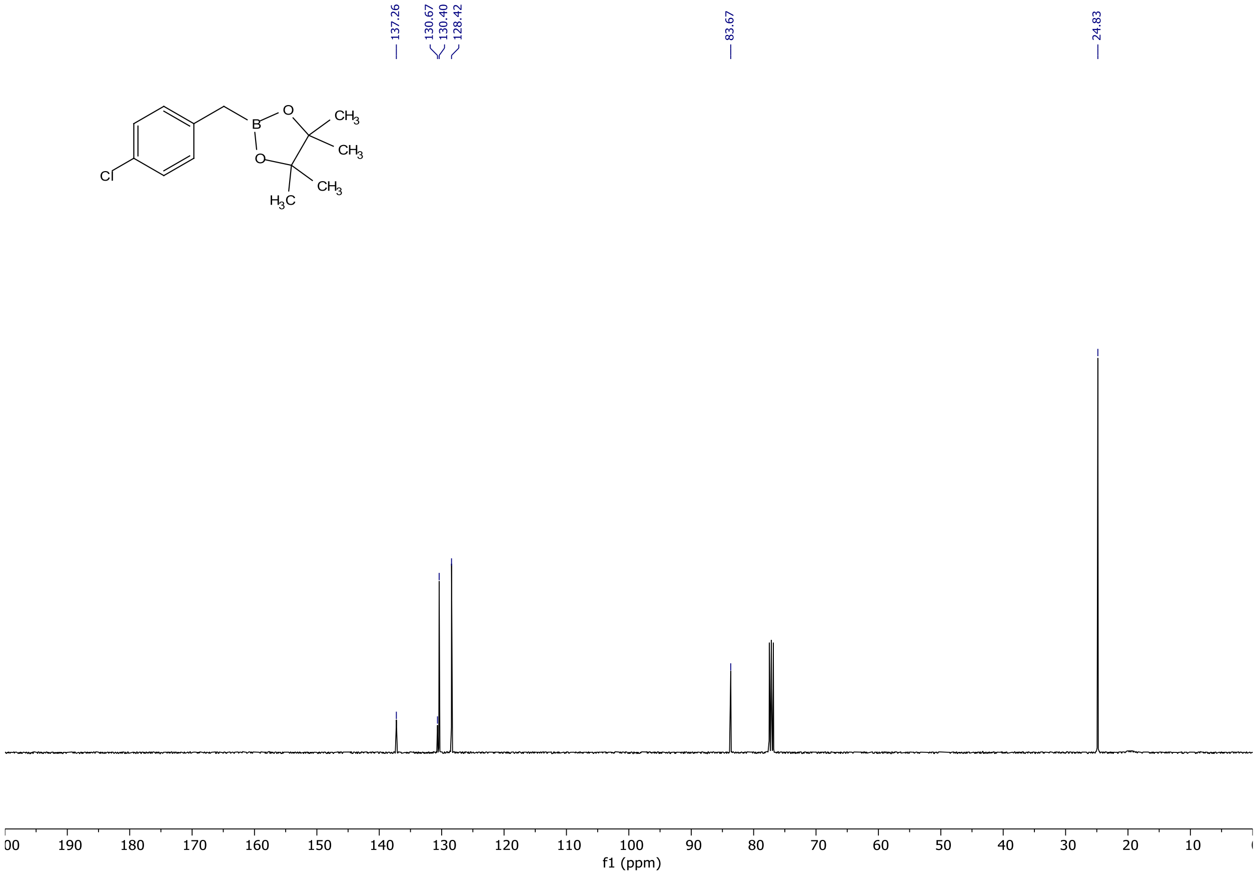

^{11}B NMR (128 MHz, $CDCl_3$)

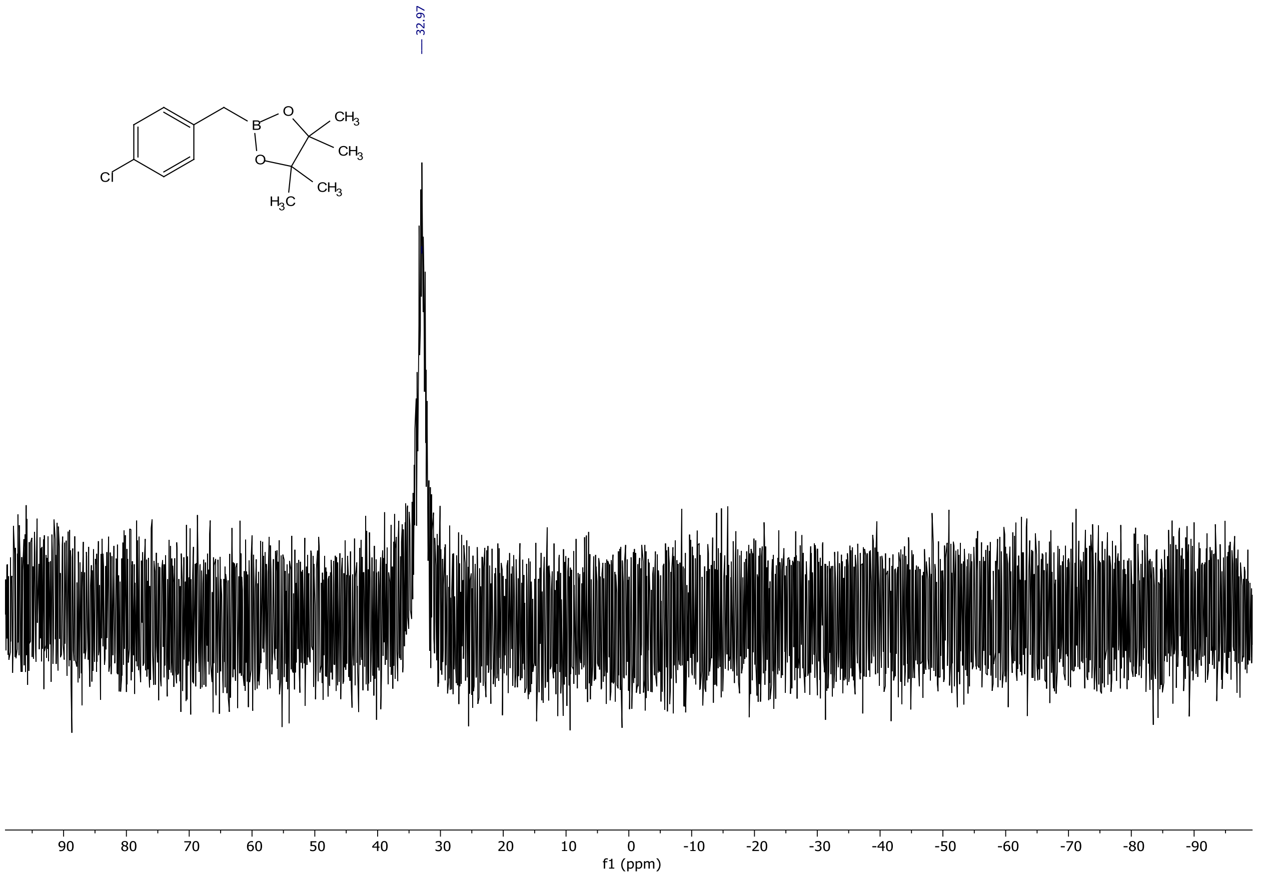

S10.44 2d

^{1}H NMR (400 MHz, $CDCl_3$)

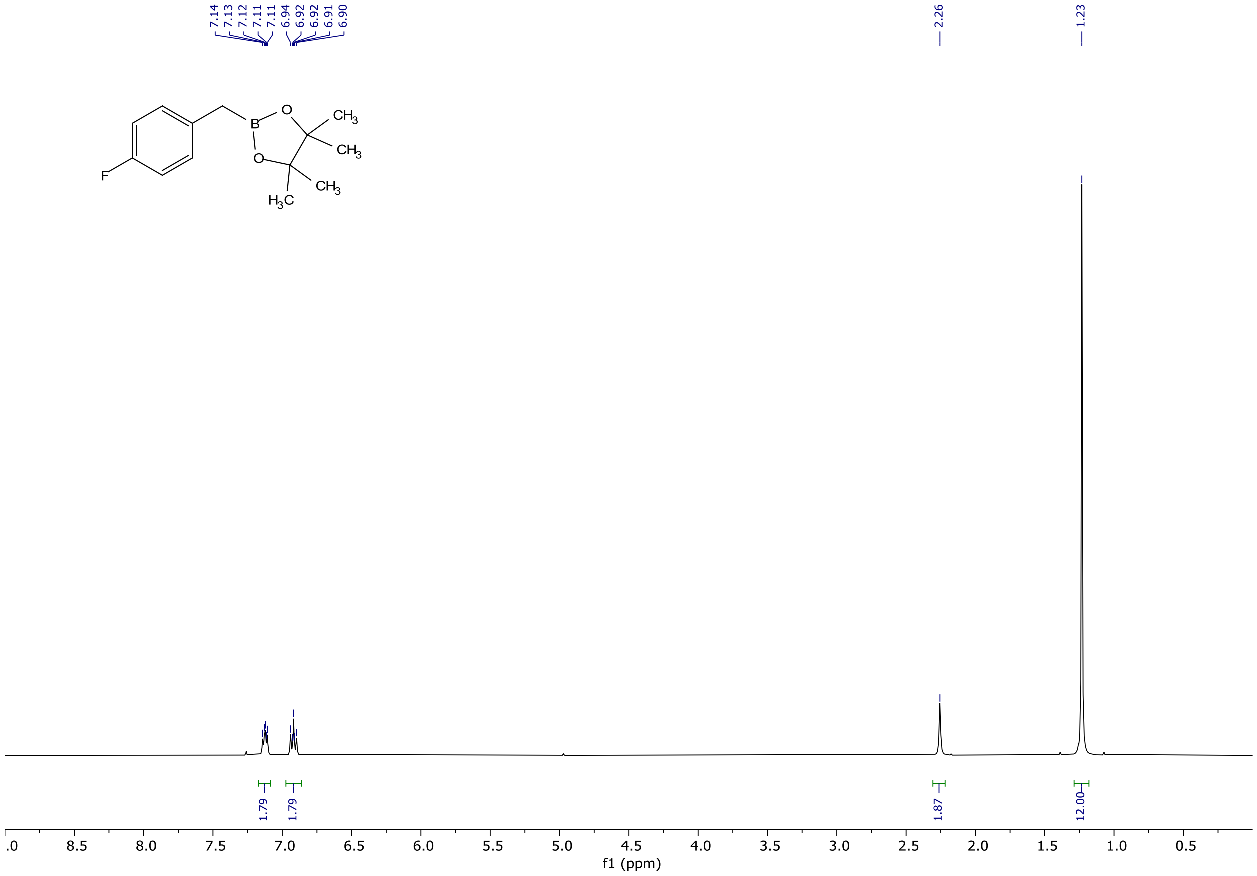

^{13}C NMR (101 MHz, $CDCl_3$)

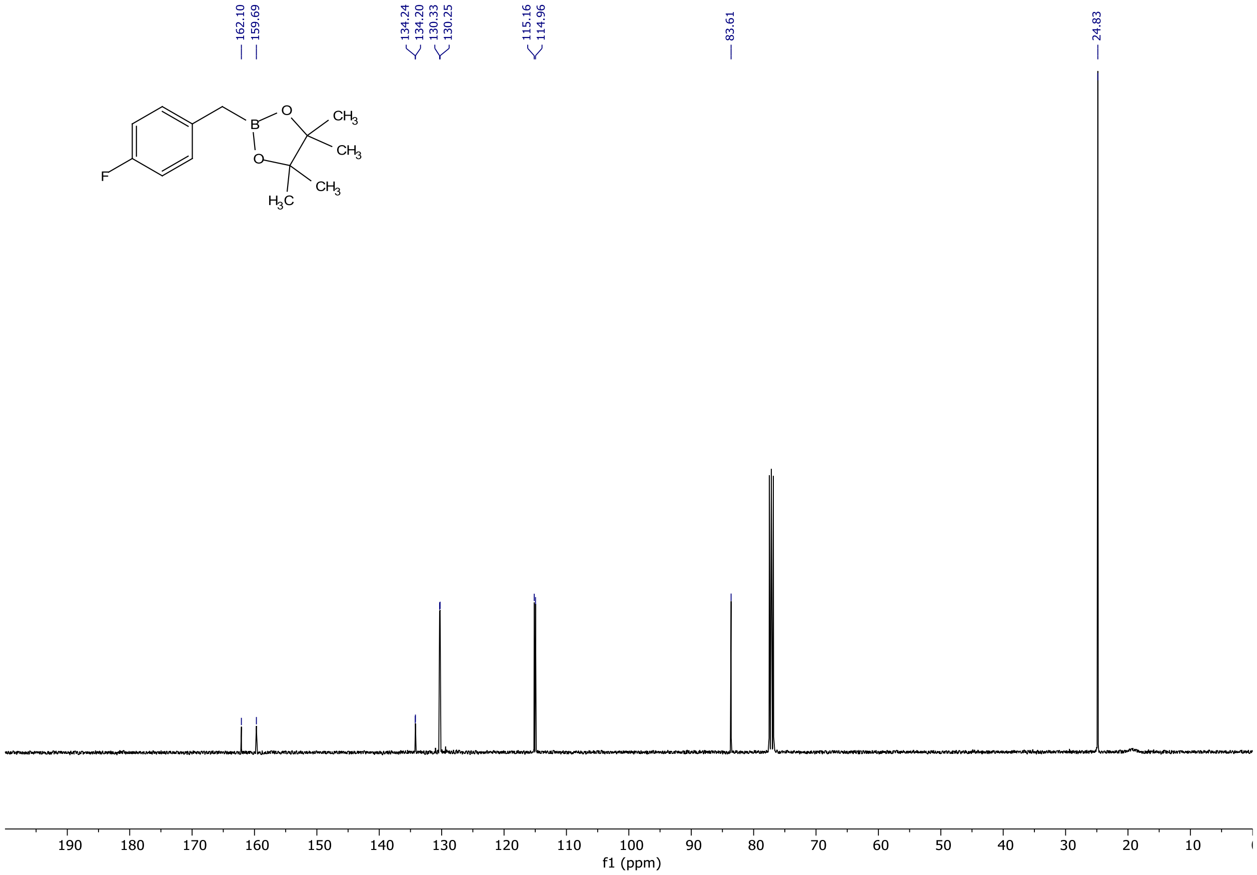

^{11}B NMR (128 MHz, $CDCl_3$)

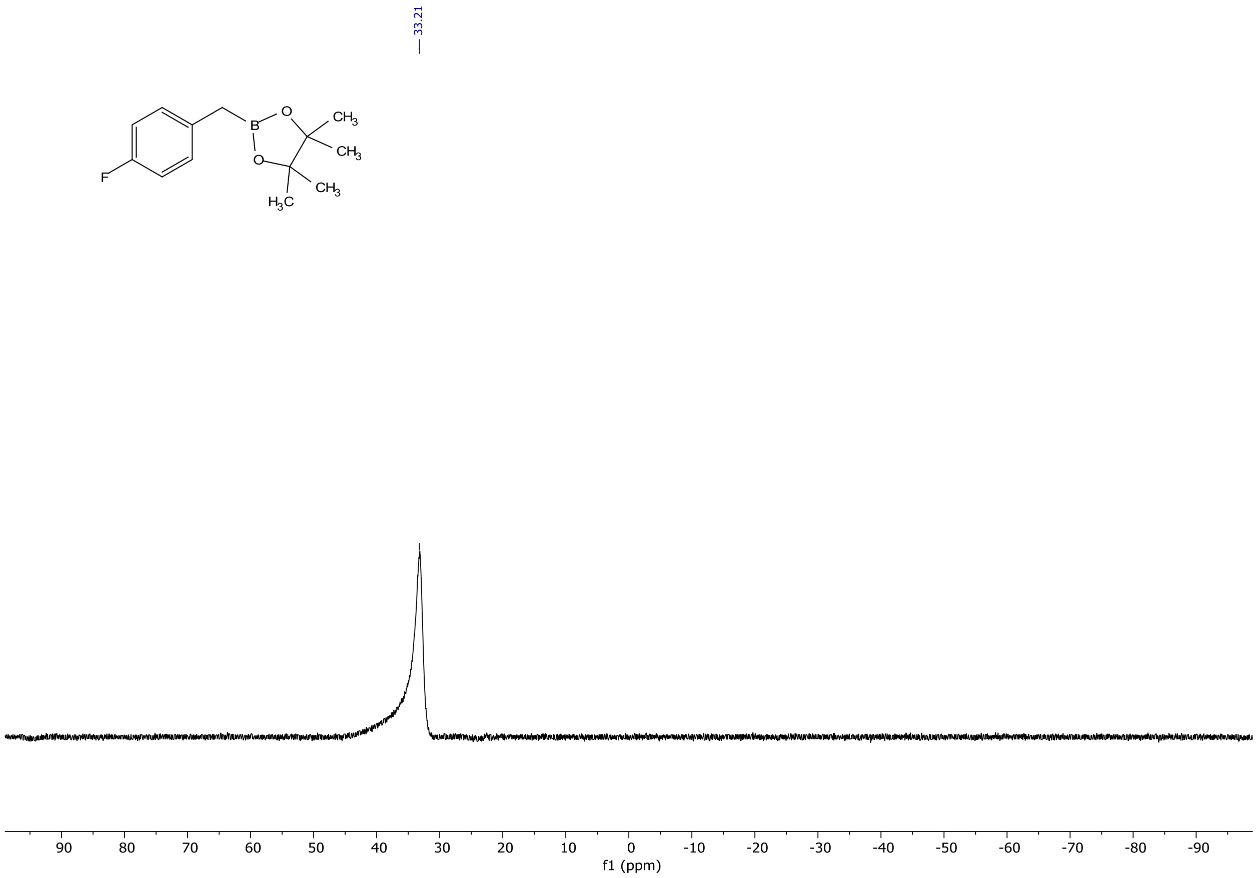

^{19}F NMR (377 MHz, $CDCl_3$)

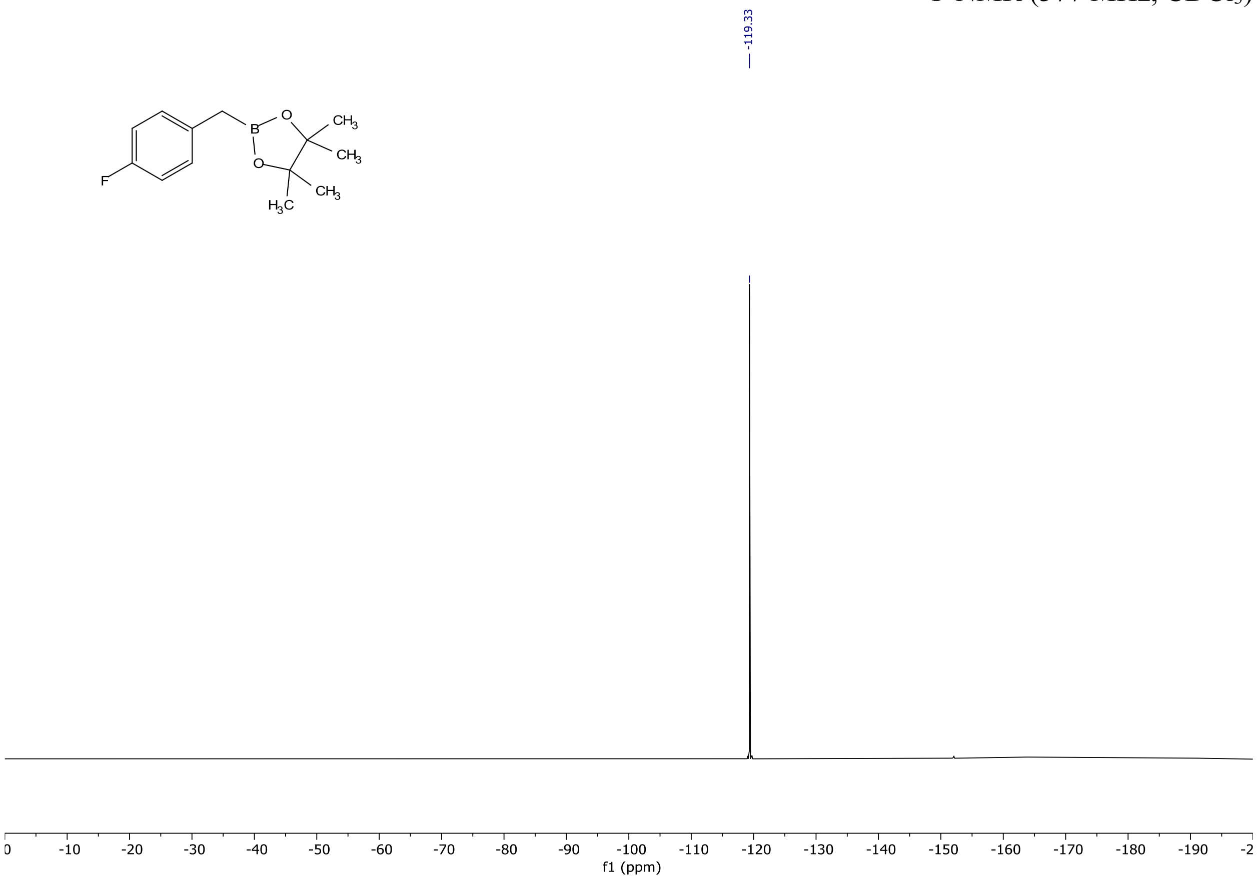

S10.45 2e

^{1}H NMR (400 MHz, $CDCl_3$)

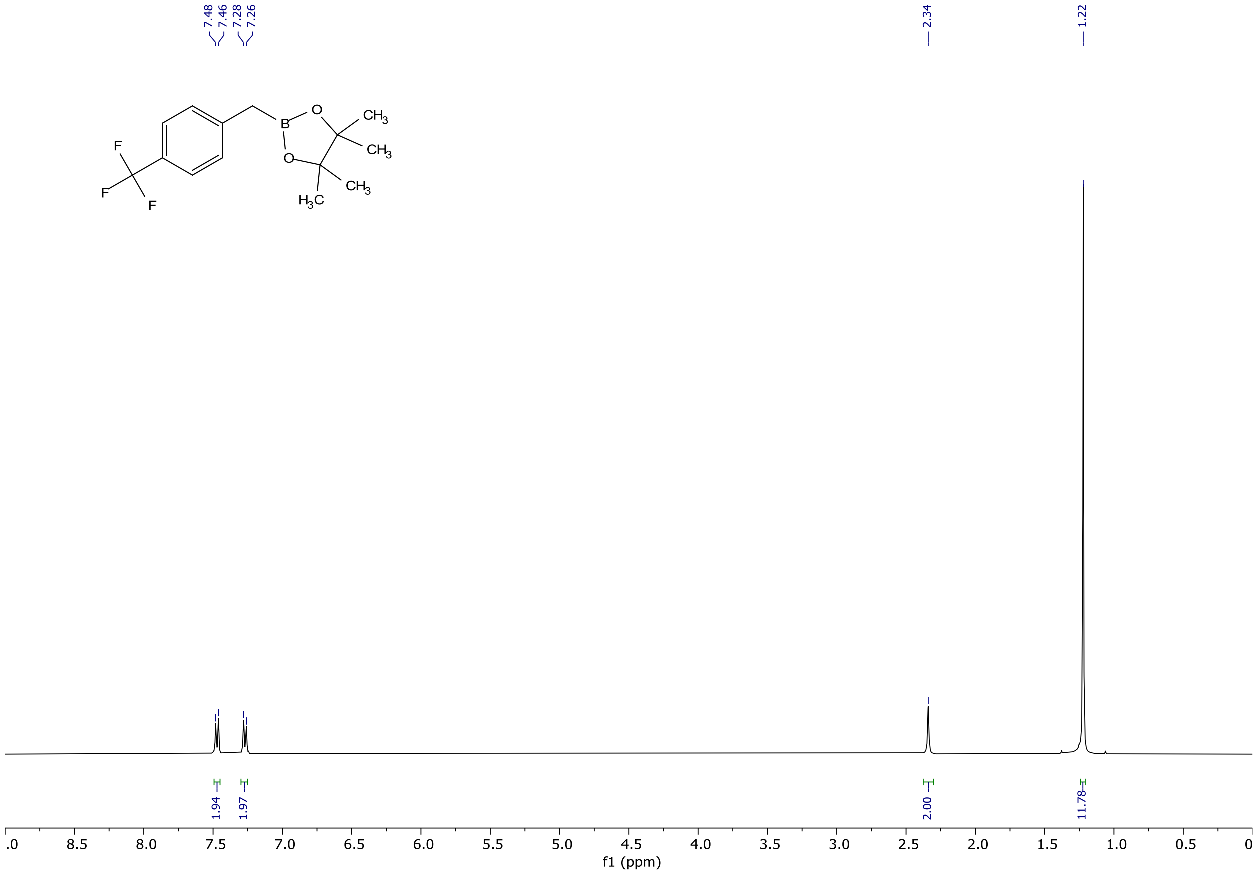

^{13}C NMR (101 MHz, $CDCl_3$)

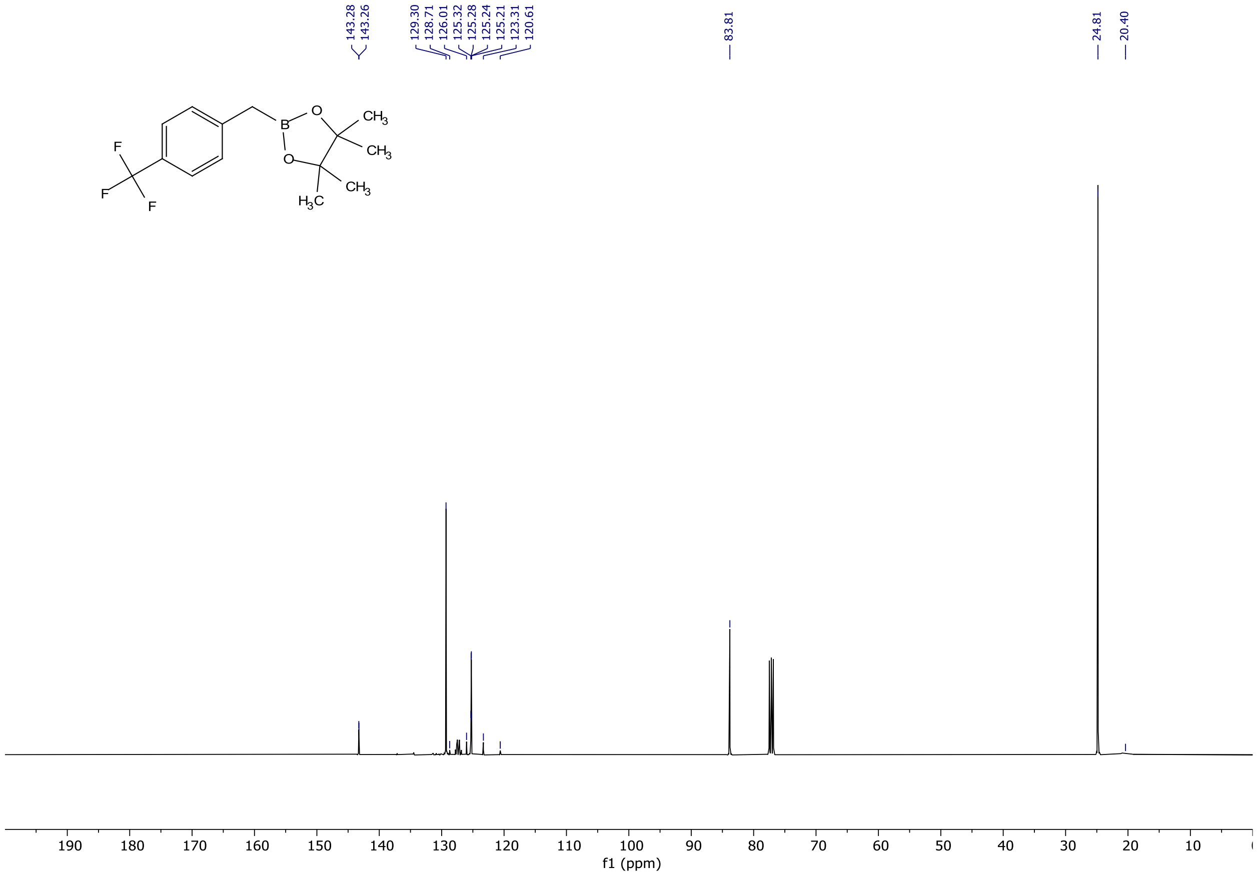

^{11}B NMR (128 MHz, $CDCl_3$)

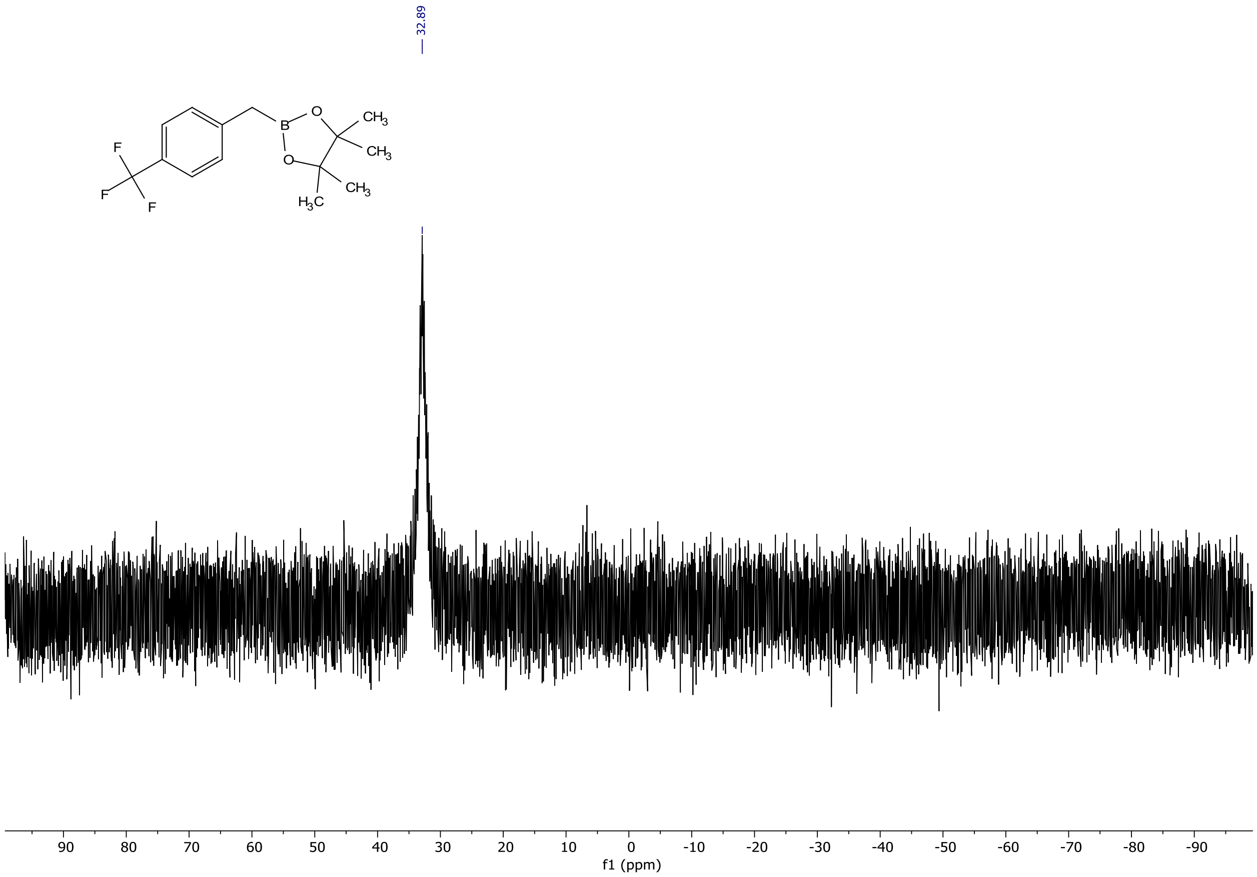

^{19}F NMR (377 MHz, $CDCl_3$)

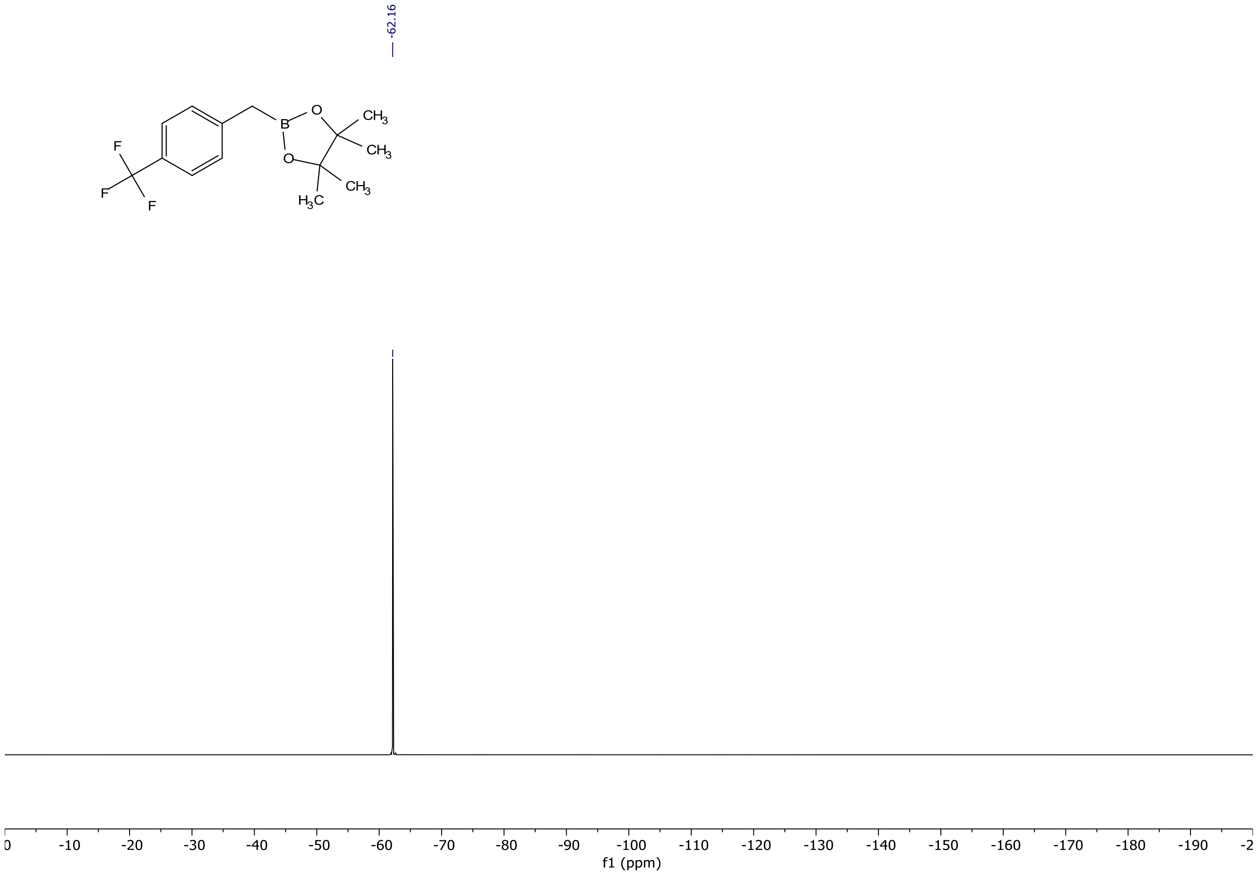

S10.46 2f

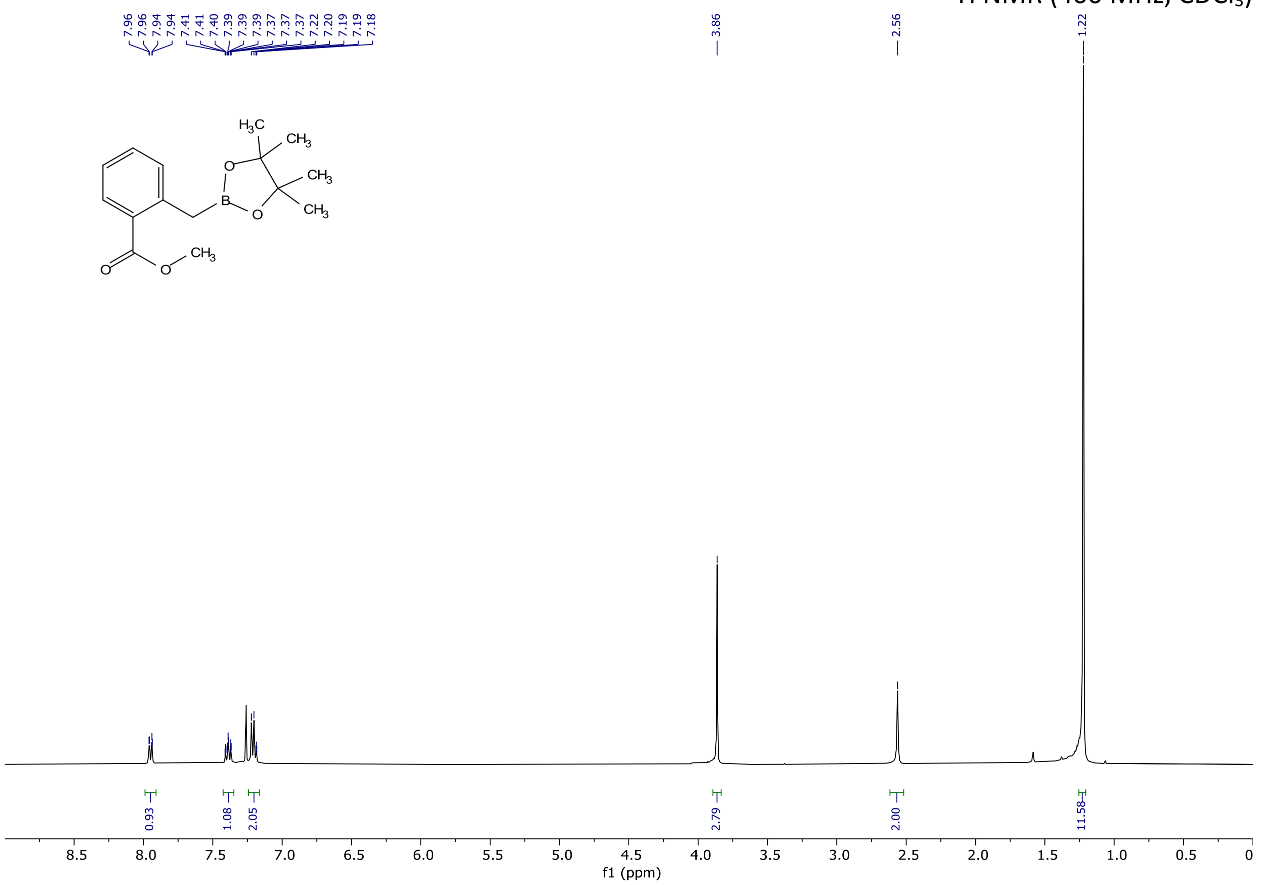

^{1}H NMR (400 MHz, CDCl$_3$)
7.96 7.96 7.94 7.94 7.41 7.41 7.40 7.39 7.39 7.39 7.37 7.37 7.37 7.22 7.20 7.19 7.19 7.18
3.86
2.56
1.22
0.93 1.08 2.05 2.79 2.00 11.58
f1 (ppm)

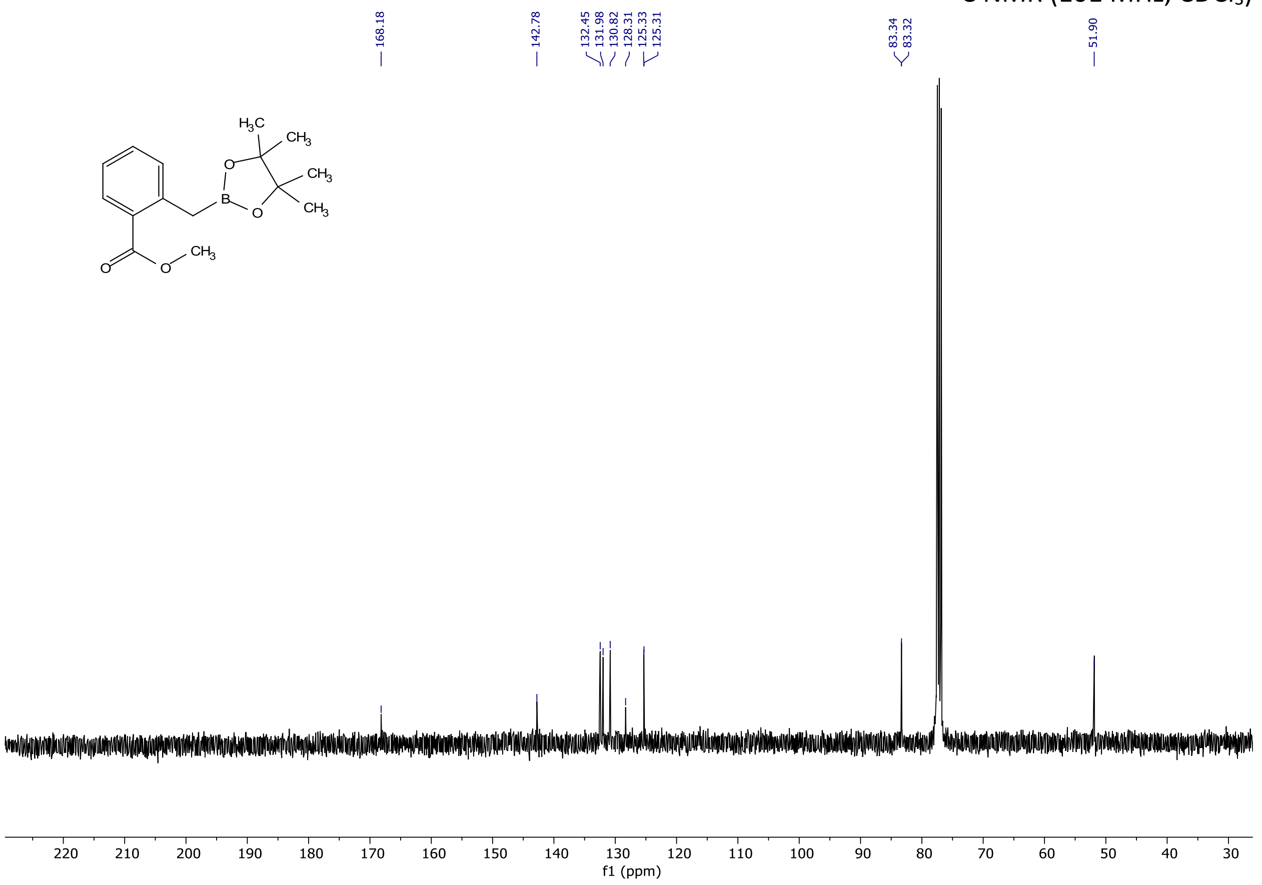

^{13}C NMR (101 MHz, CDCl$_3$)
168.18
142.78
132.45 131.98 130.82 128.31 125.33 125.31
83.34 83.32
51.90
f1 (ppm)

^{11}B NMR (128 MHz, $CDCl_3$)

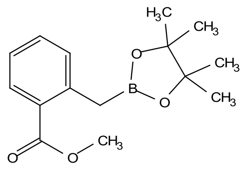

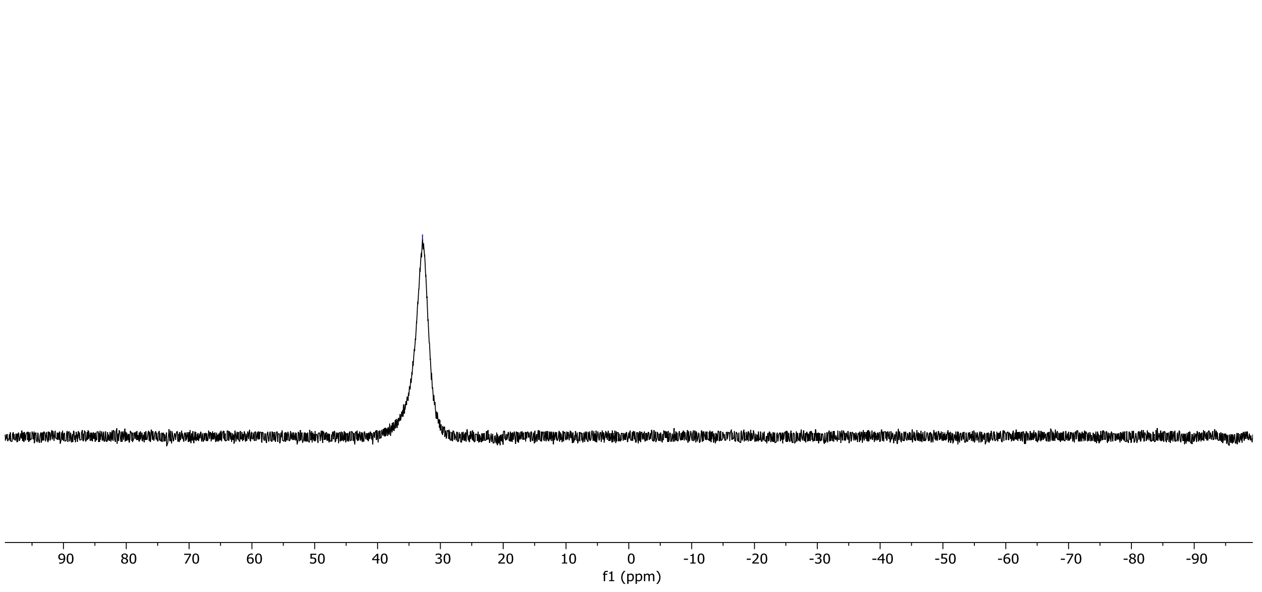

S10.47 **2g**

^{1}H NMR (400 MHz, $CDCl_3$)

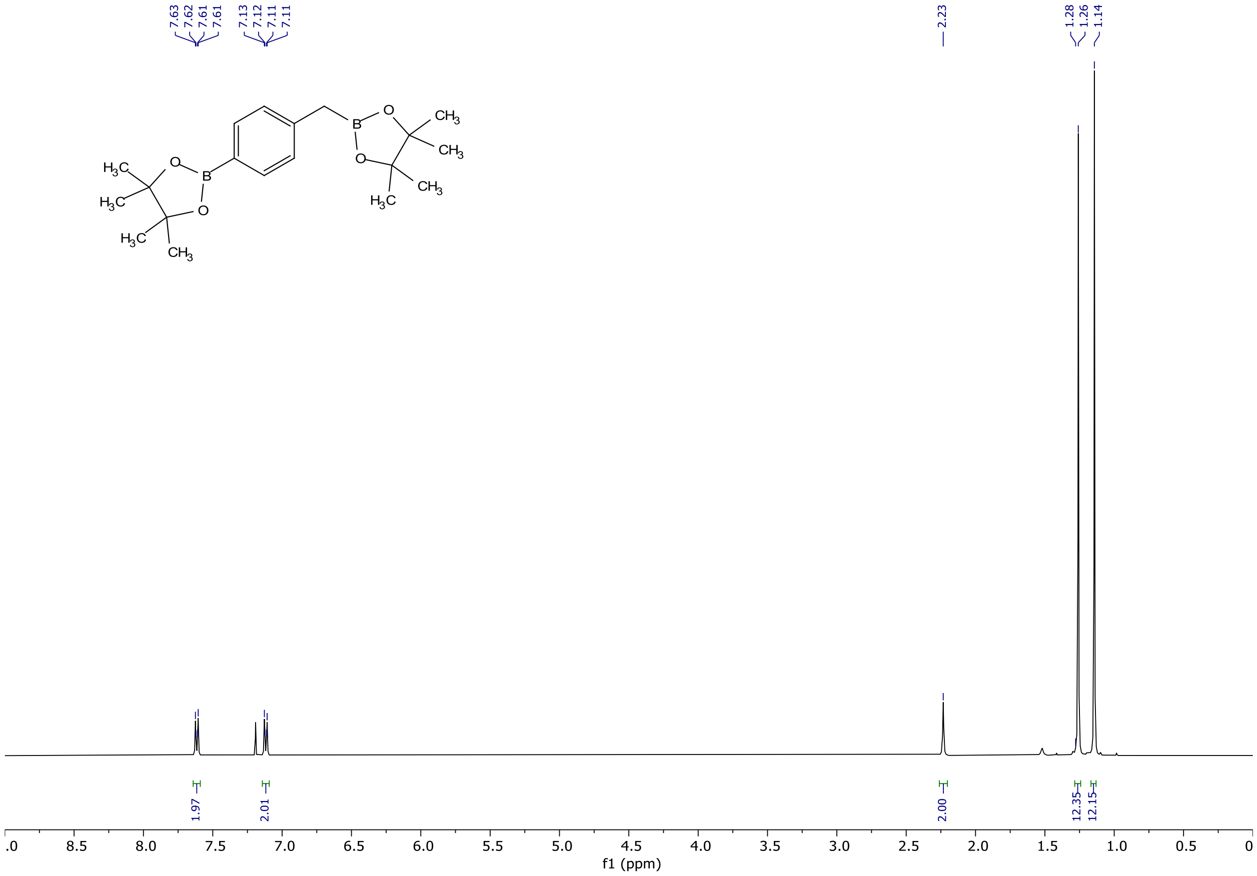

^{13}C NMR (101 MHz, $CDCl_3$)

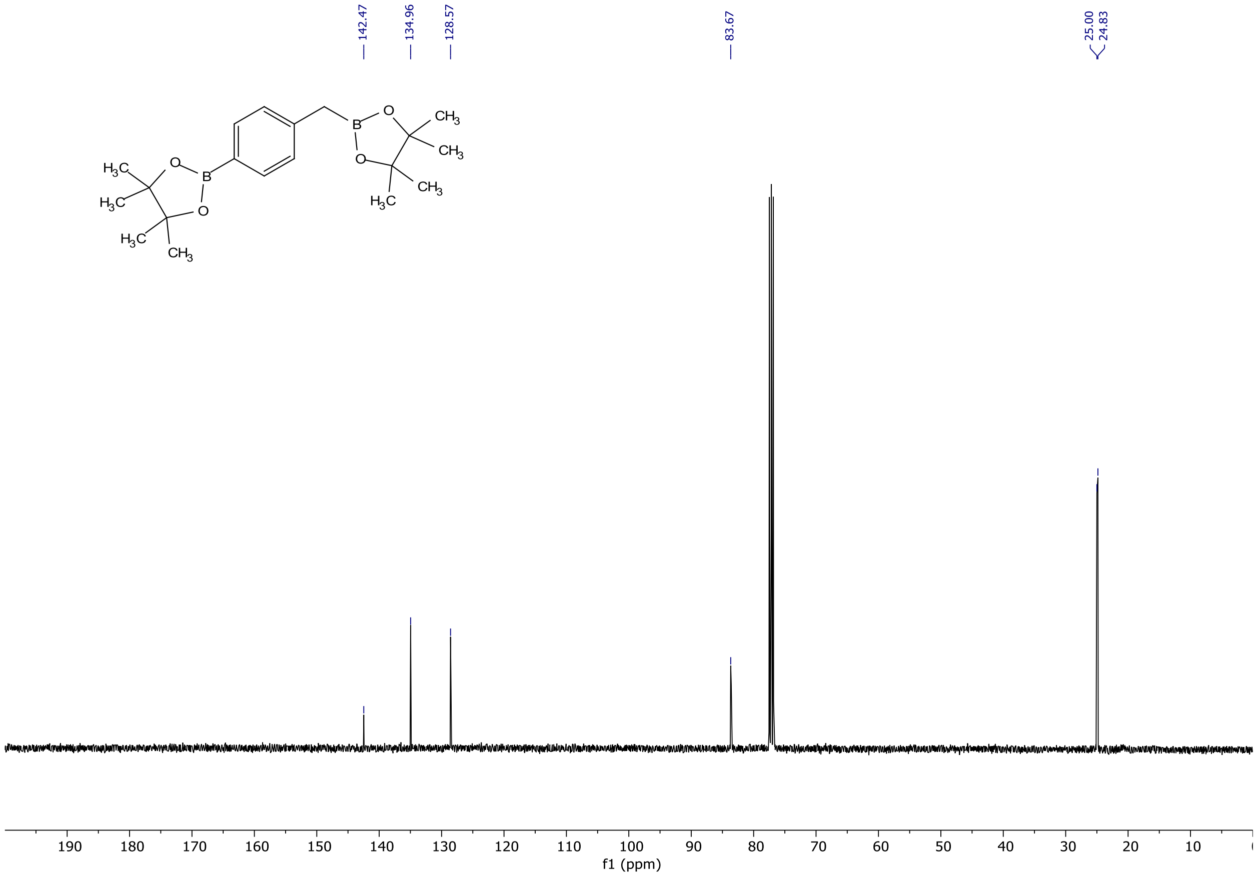

^{11}B NMR (128 MHz, $CDCl_3$)

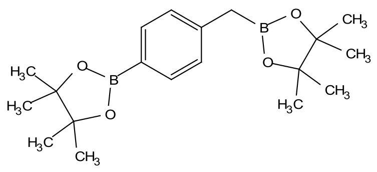

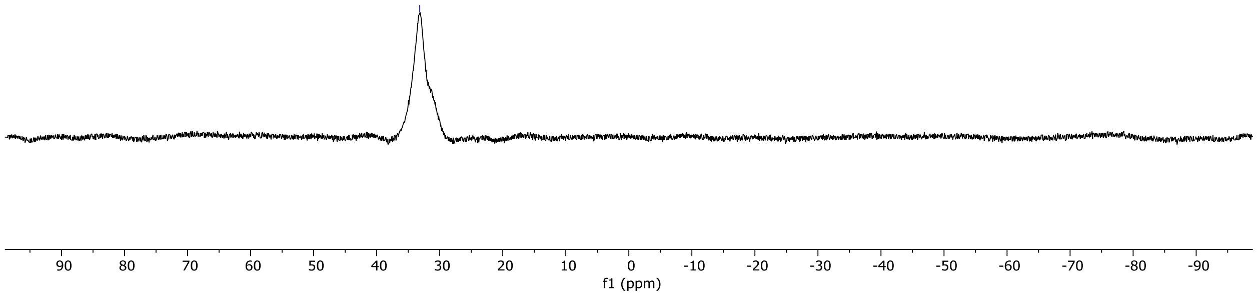

S10.48 2h

^{1}H NMR (500 MHz, $CDCl_3$)

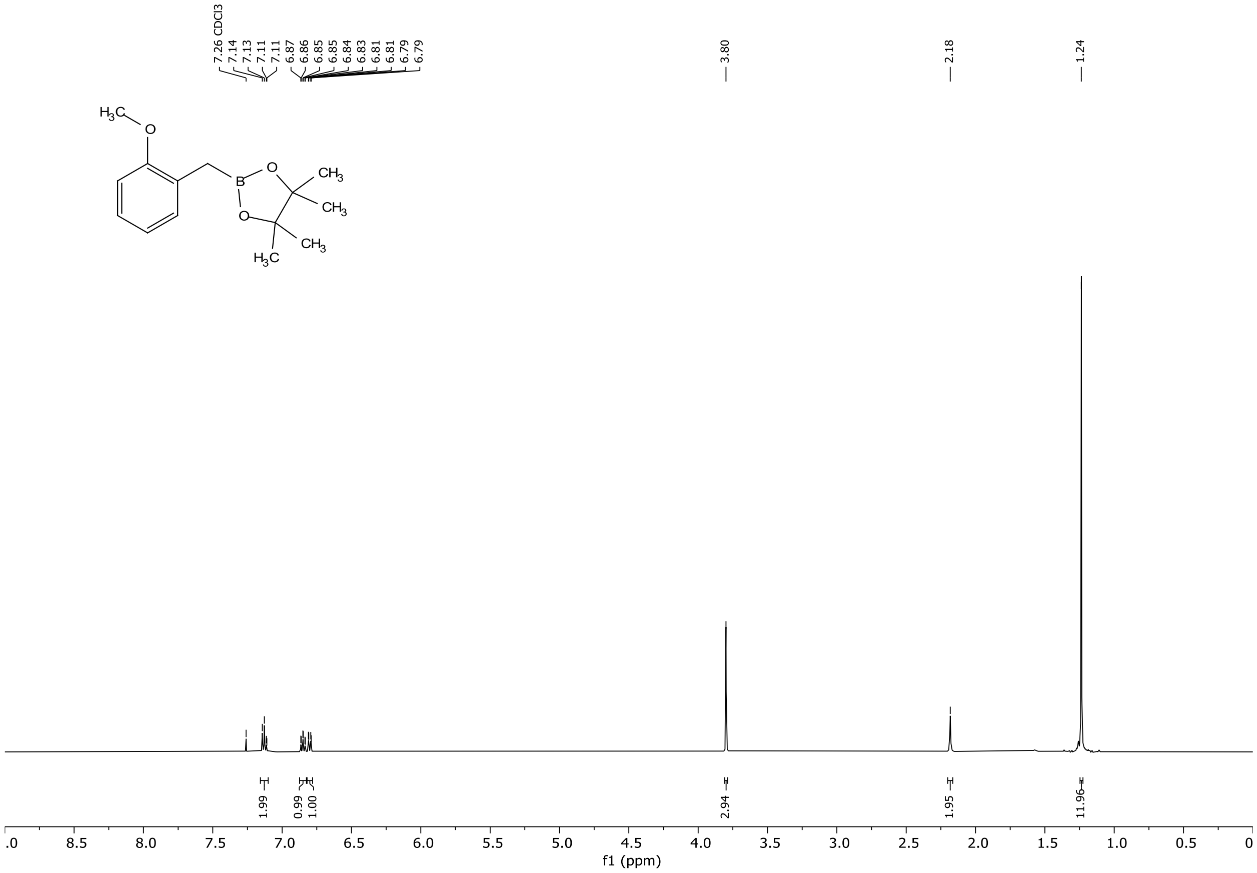

^{13}C NMR (126 MHz, $CDCl_3$)

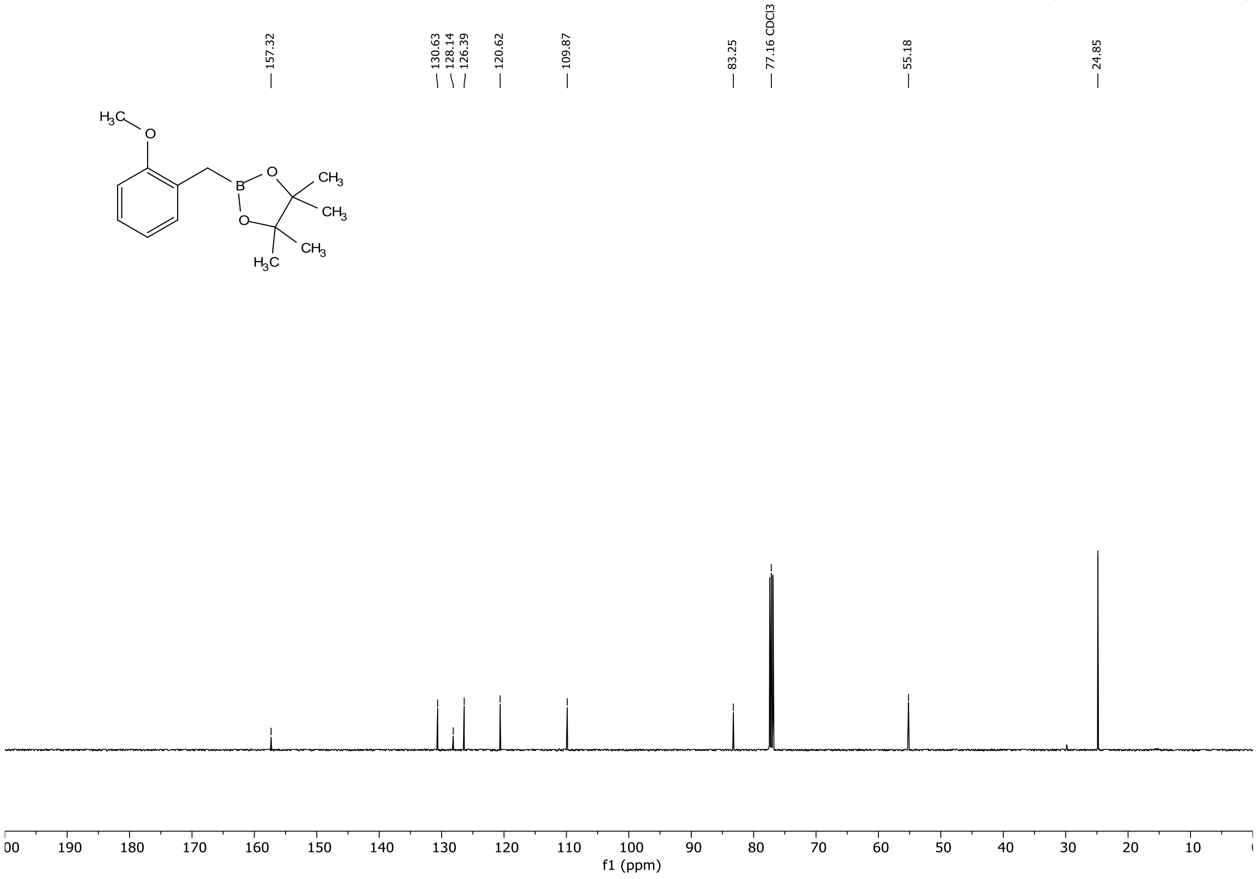

^{11}B NMR (160 MHz, $CDCl_3$)

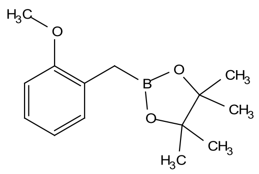

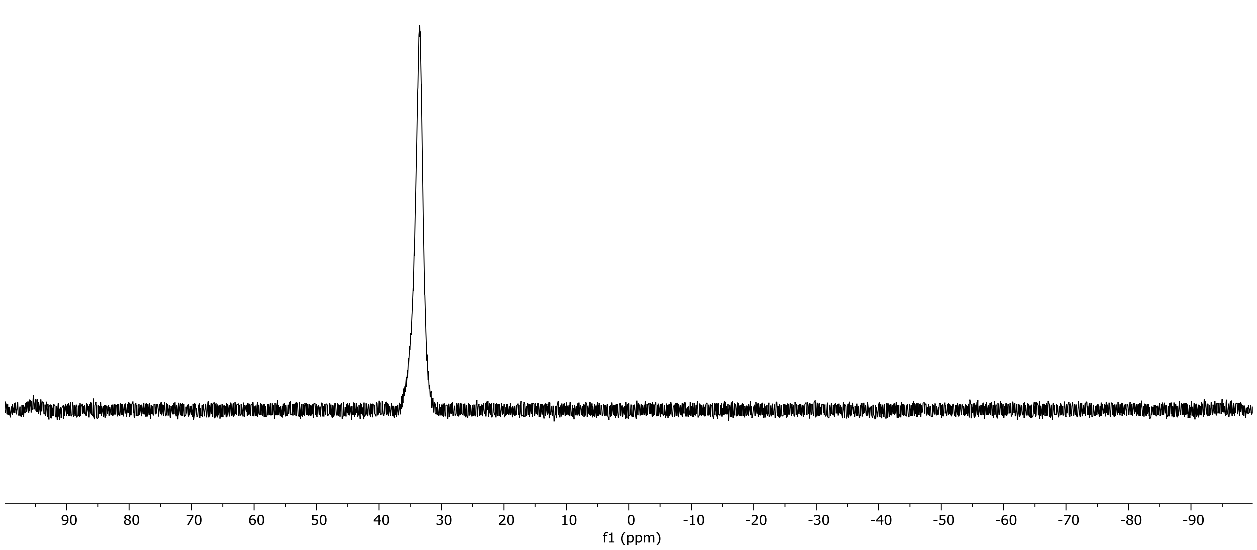

S10.49 2i

^{1}H NMR (400 MHz, $CDCl_3$)

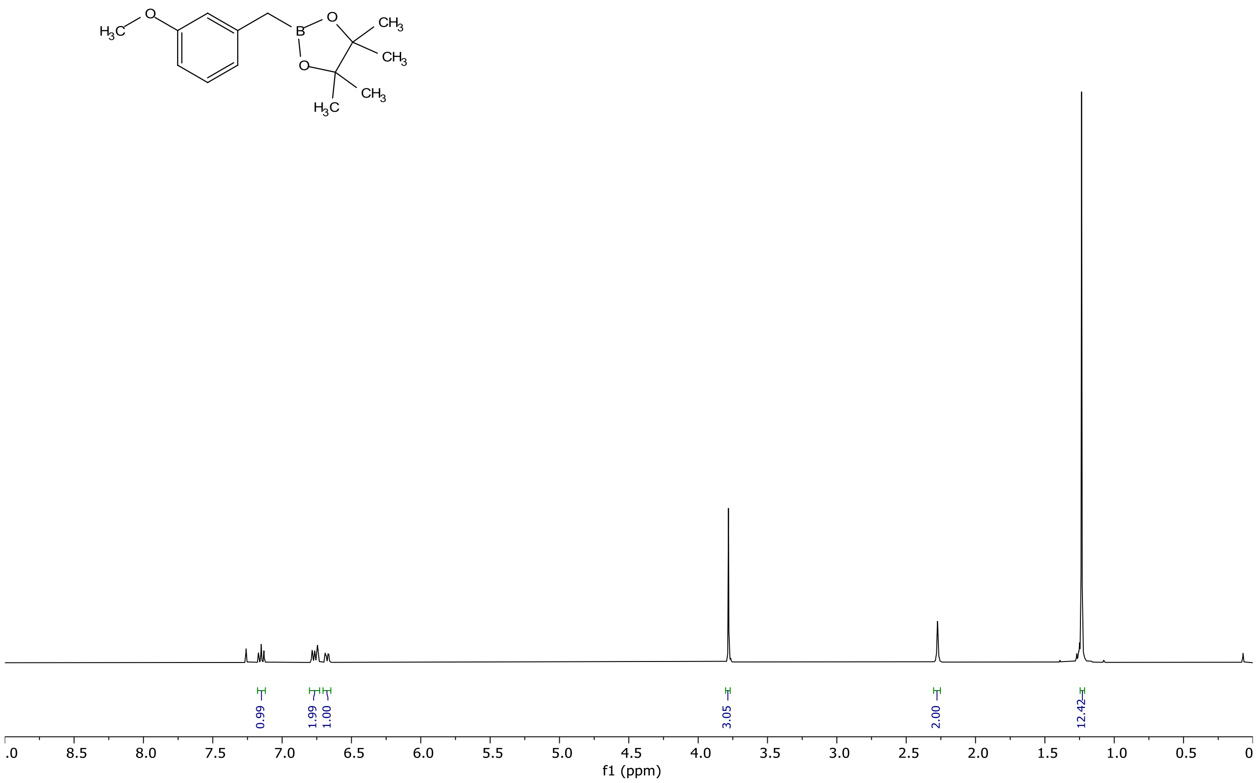

^{13}C NMR (101 MHz, $CDCl_3$)

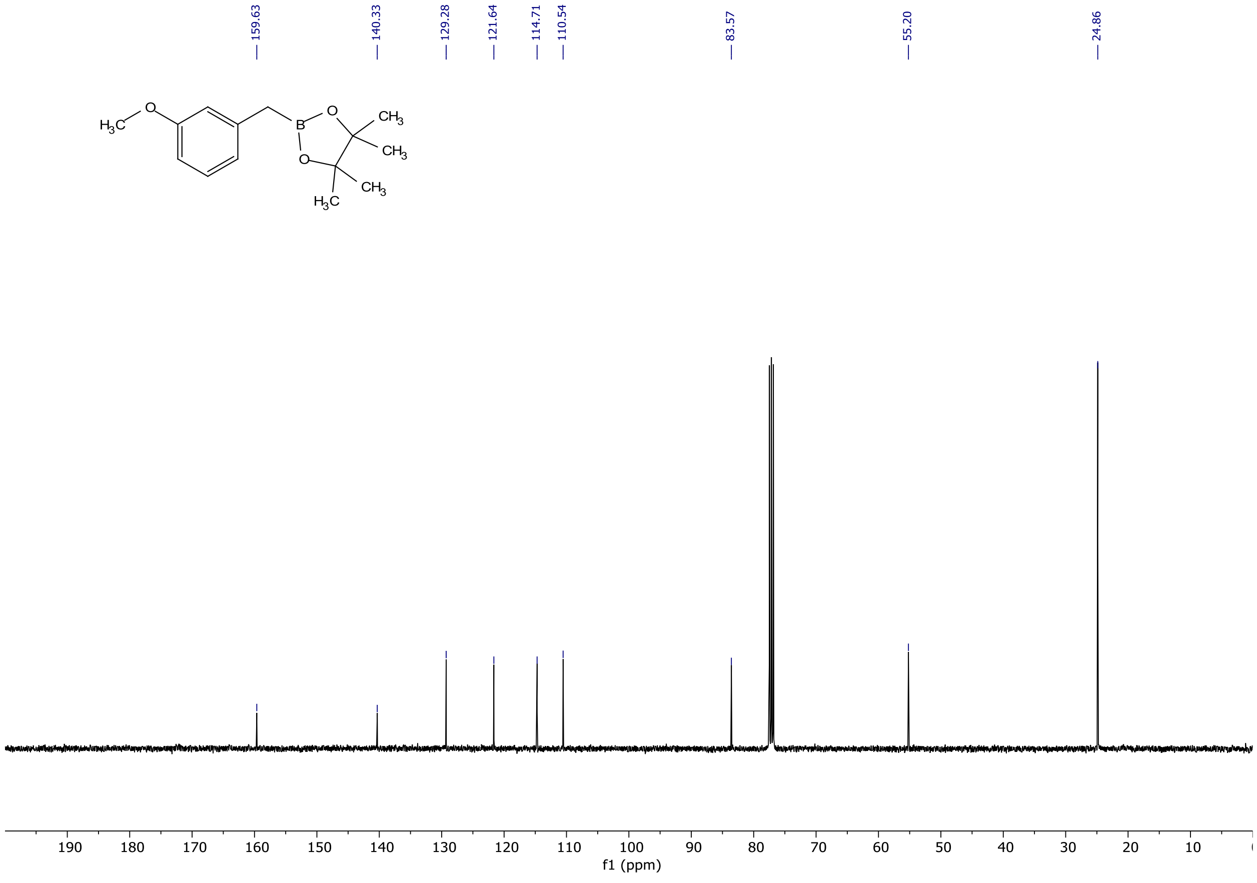

S10.50 2j

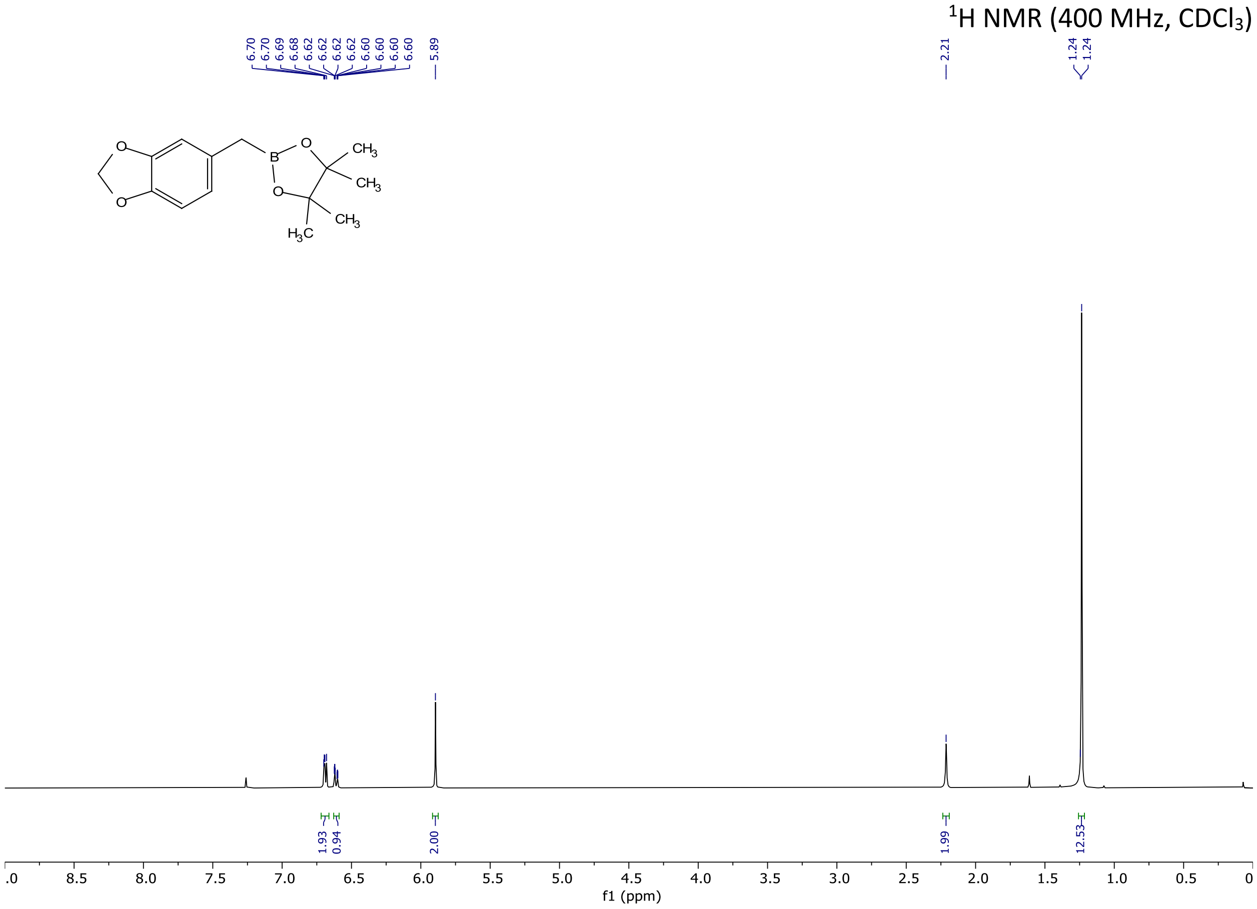
^{1}H NMR (400 MHz, CDCl$_3$)
6.70
6.70
6.69
6.68
6.62
6.62
6.62
6.62
6.60
6.60
6.60
6.60
5.89
2.21
1.24
1.24
O
O
B
O
O
CH$_3$
CH$_3$
CH$_3$
H$_3$C
1.93
0.94
2.00
1.99
12.53
f1 (ppm)

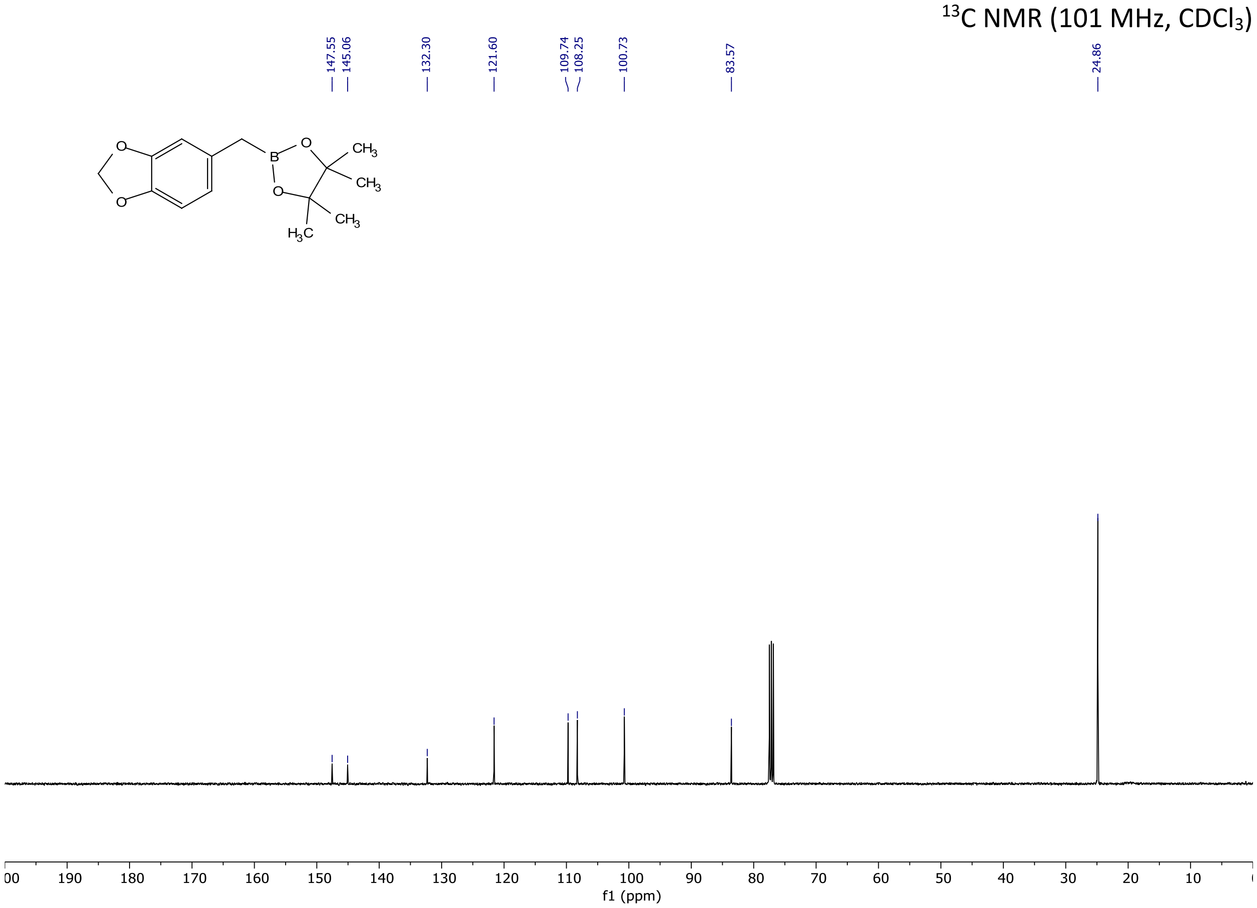
^{13}C NMR (101 MHz, CDCl$_3$)
147.55
145.06
132.30
121.60
109.74
108.25
100.73
83.57
24.86
O
O
B
O
O
CH$_3$
CH$_3$
CH$_3$
H$_3$C
f1 (ppm)

^{11}B NMR (128 MHz, $CDCl_3$)

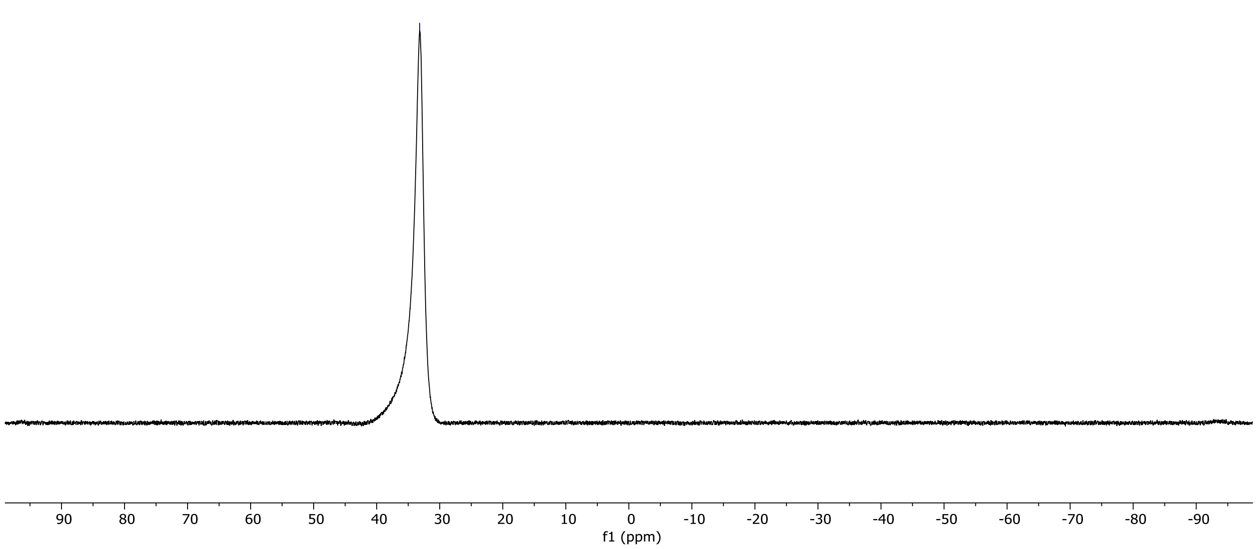

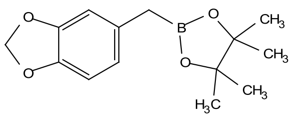

S10.51 2k

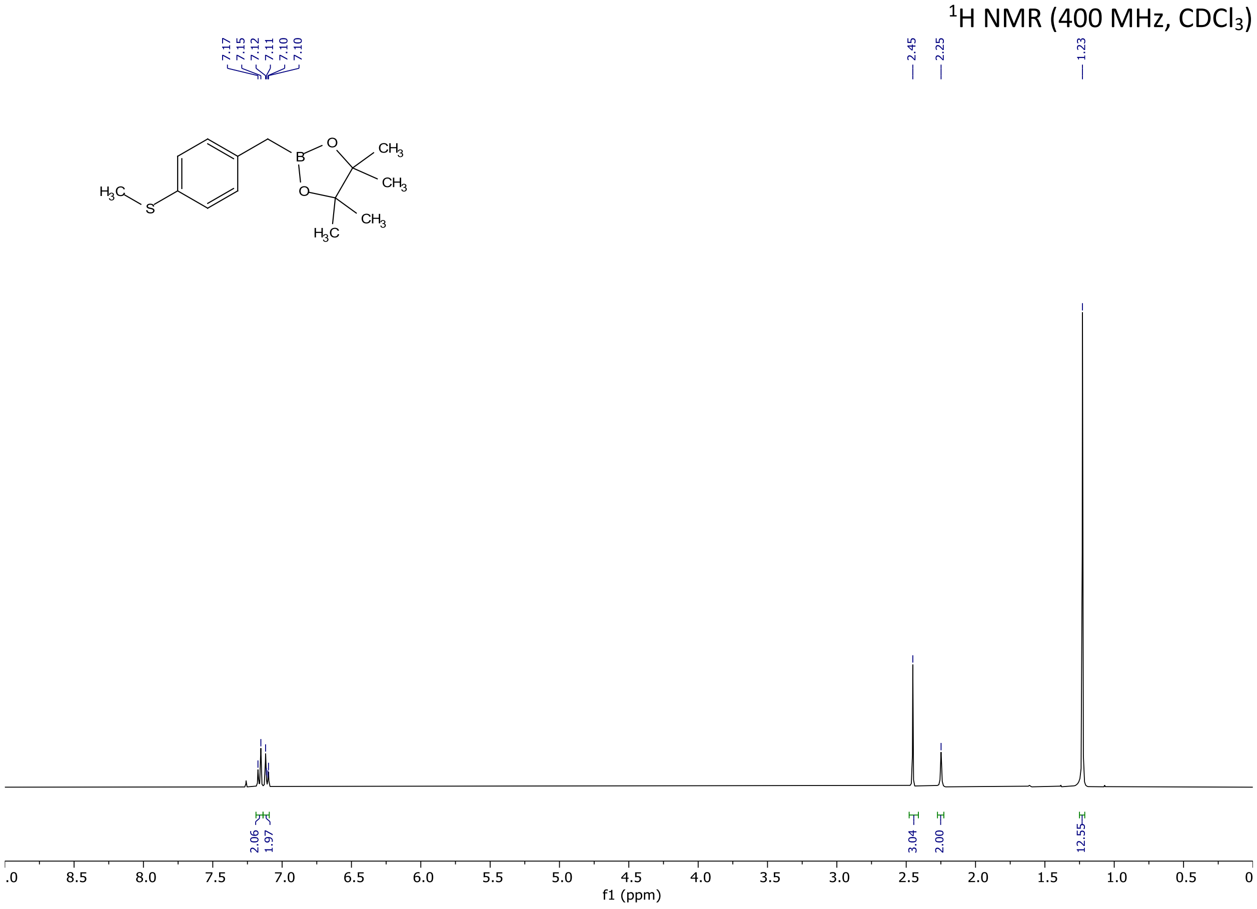

^{1}H NMR (400 MHz, $CDCl_3$)
7.17
7.15
7.12
7.11
7.10
7.10
2.45
2.25
1.23
2.06
1.97
3.04
2.00
12.55
f1 (ppm)

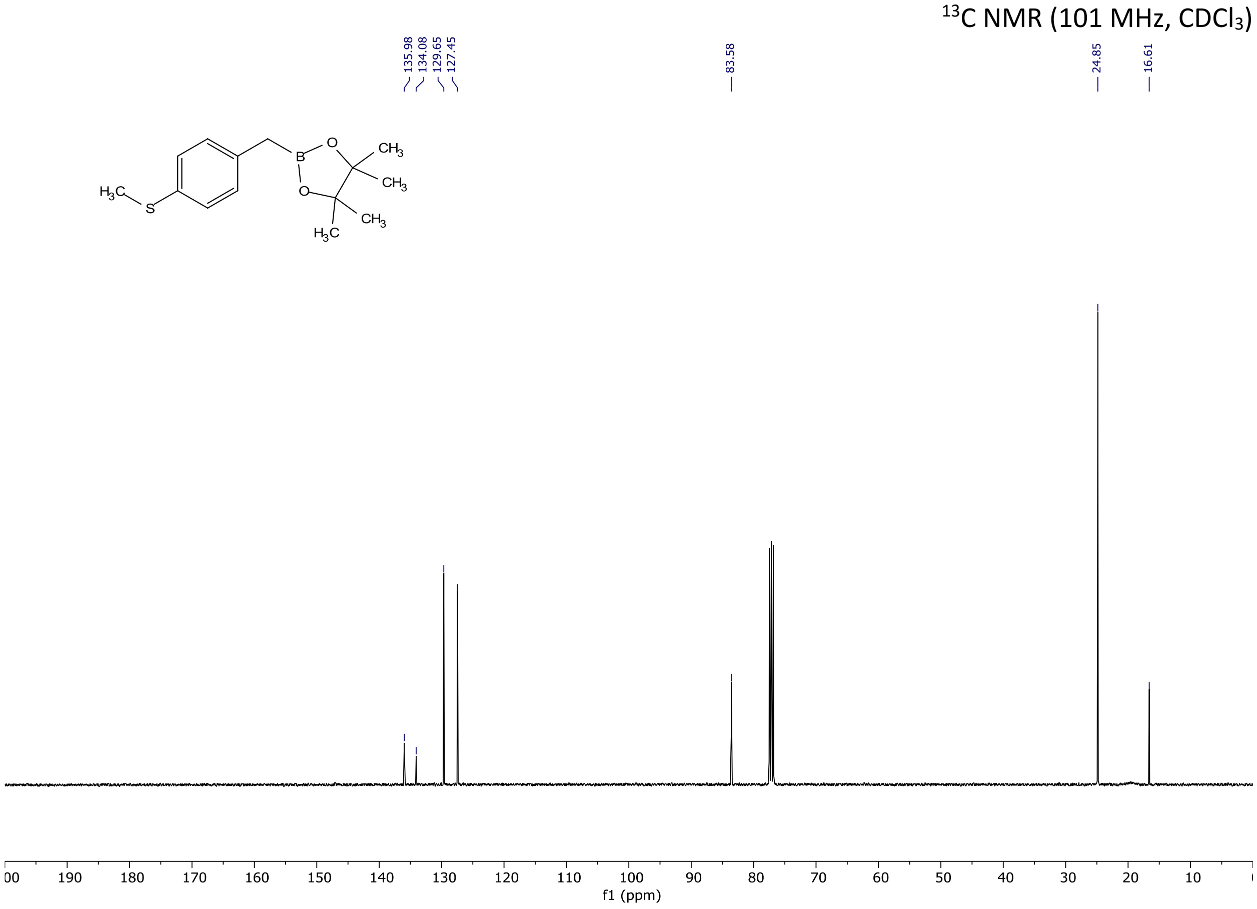

^{13}C NMR (101 MHz, $CDCl_3$)
135.98
134.08
129.65
127.45
83.58
24.85
16.61
f1 (ppm)

^{11}B NMR (128 MHz, $CDCl_3$)

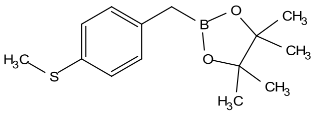

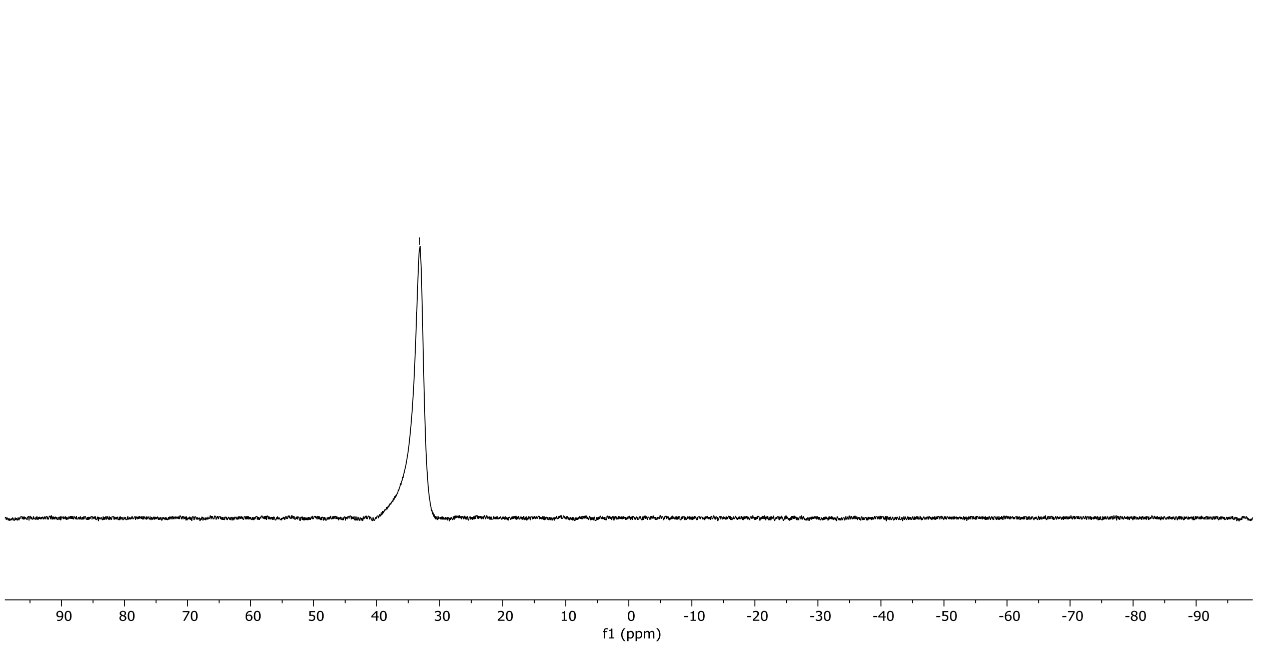

S10.52 2l

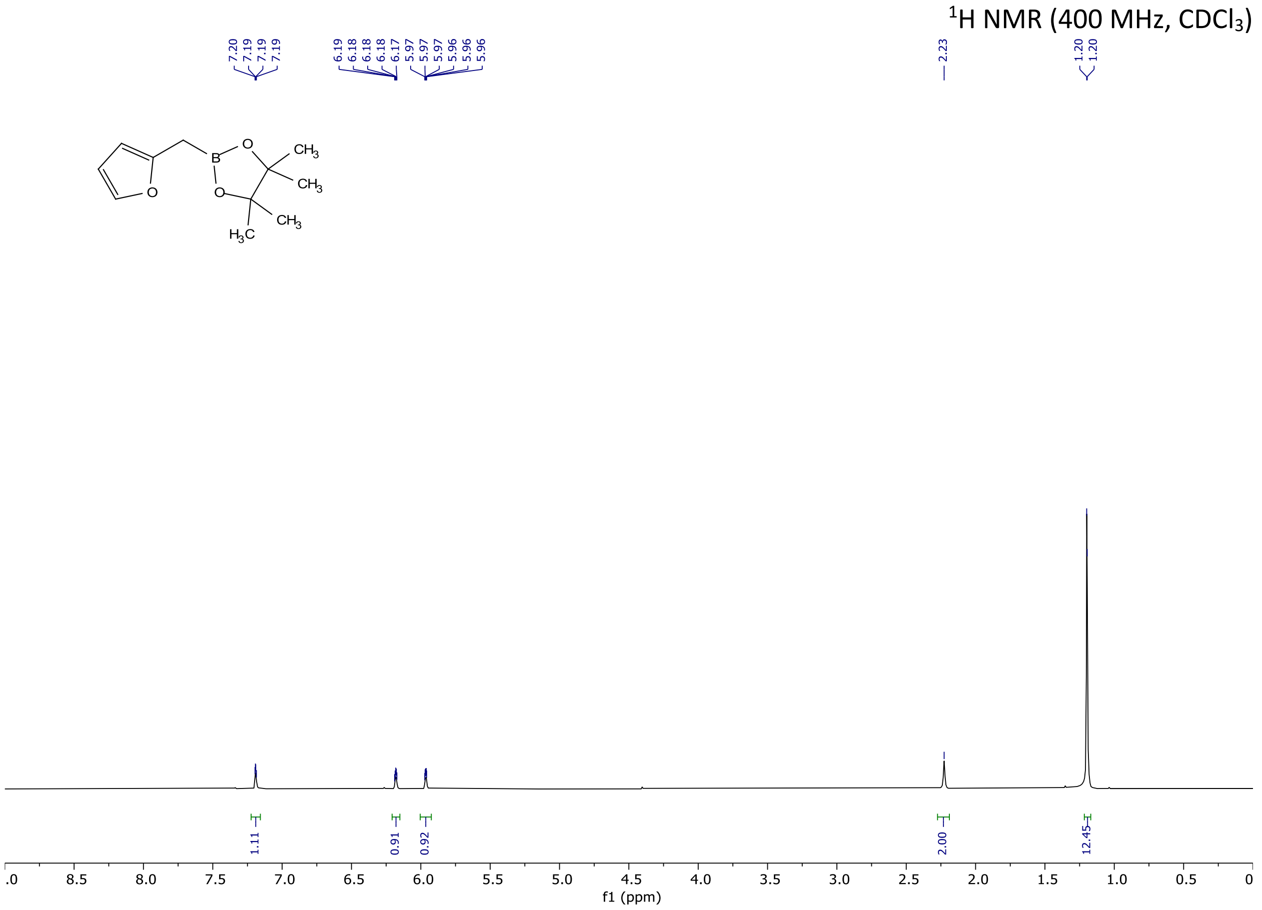

1H NMR (400 MHz, CDCl3)
7.20
7.19
7.19
7.19
6.19
6.18
6.18
6.18
6.17
5.97
5.97
5.97
5.96
5.96
5.96
2.23
1.20
1.20
CH3
CH3
CH3
H3C
B
O
O
O
1.11
0.91
0.92
2.00
12.45
f1 (ppm)

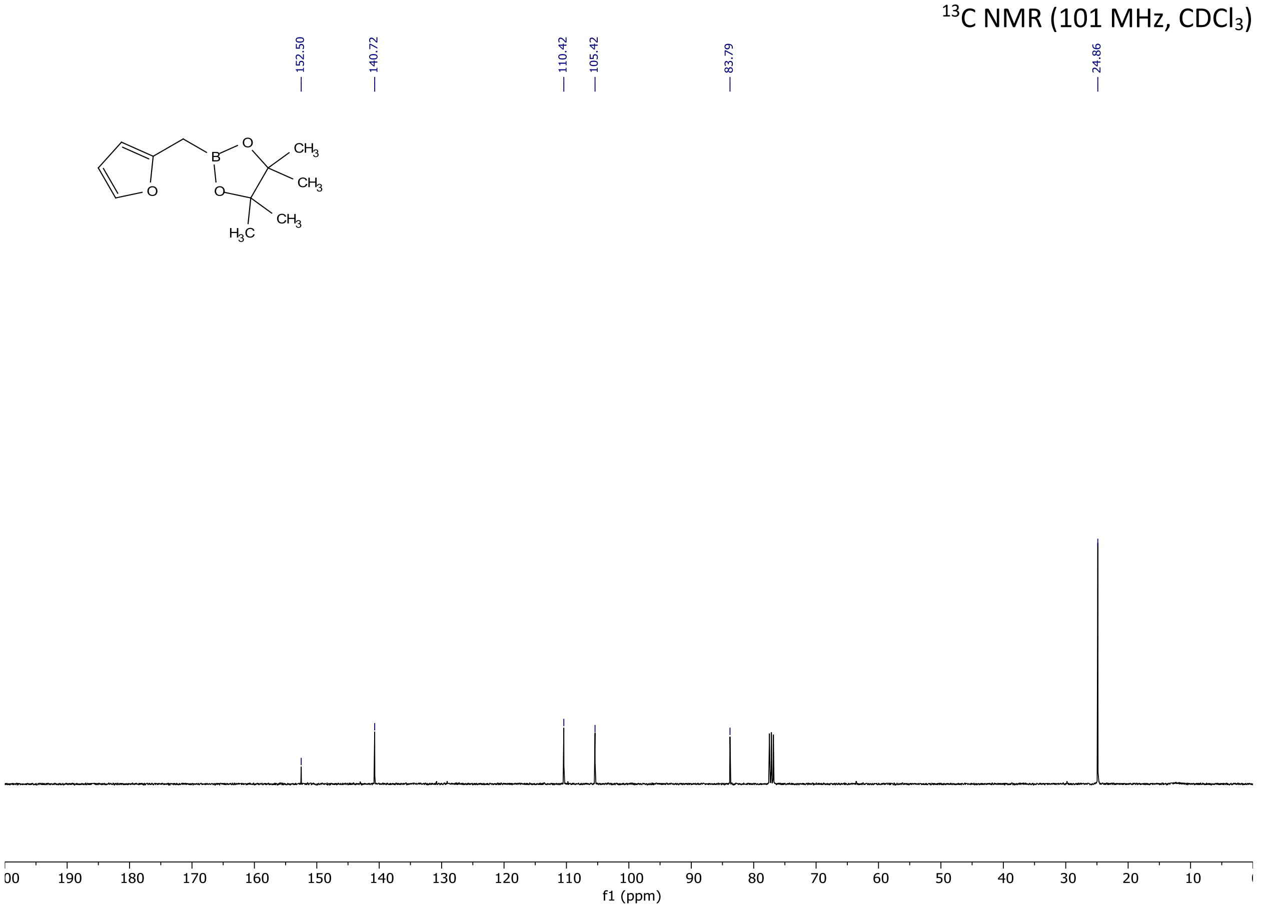

13C NMR (101 MHz, CDCl3)
152.50
140.72
110.42
105.42
83.79
24.86
CH3
CH3
CH3
H3C
B
O
O
O
f1 (ppm)

^{11}B NMR (128 MHz, $CDCl_3$)

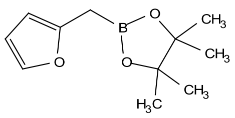

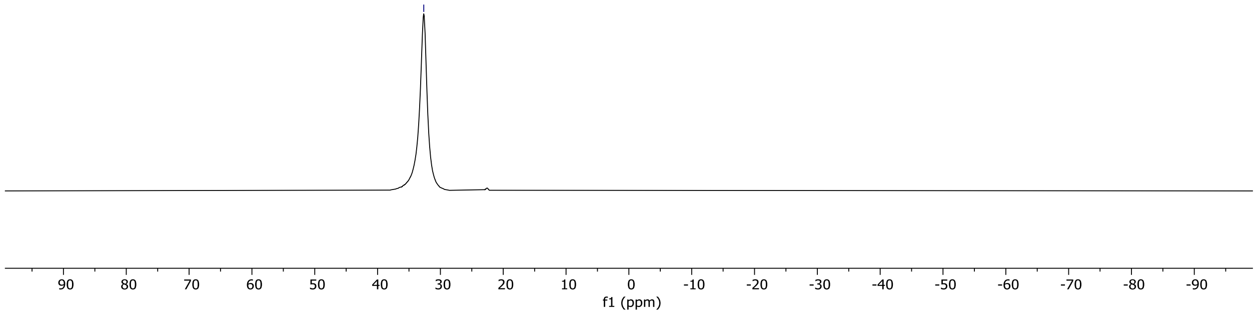

S10.53 2m

^1H NMR (400 MHz, $CDCl_3$)

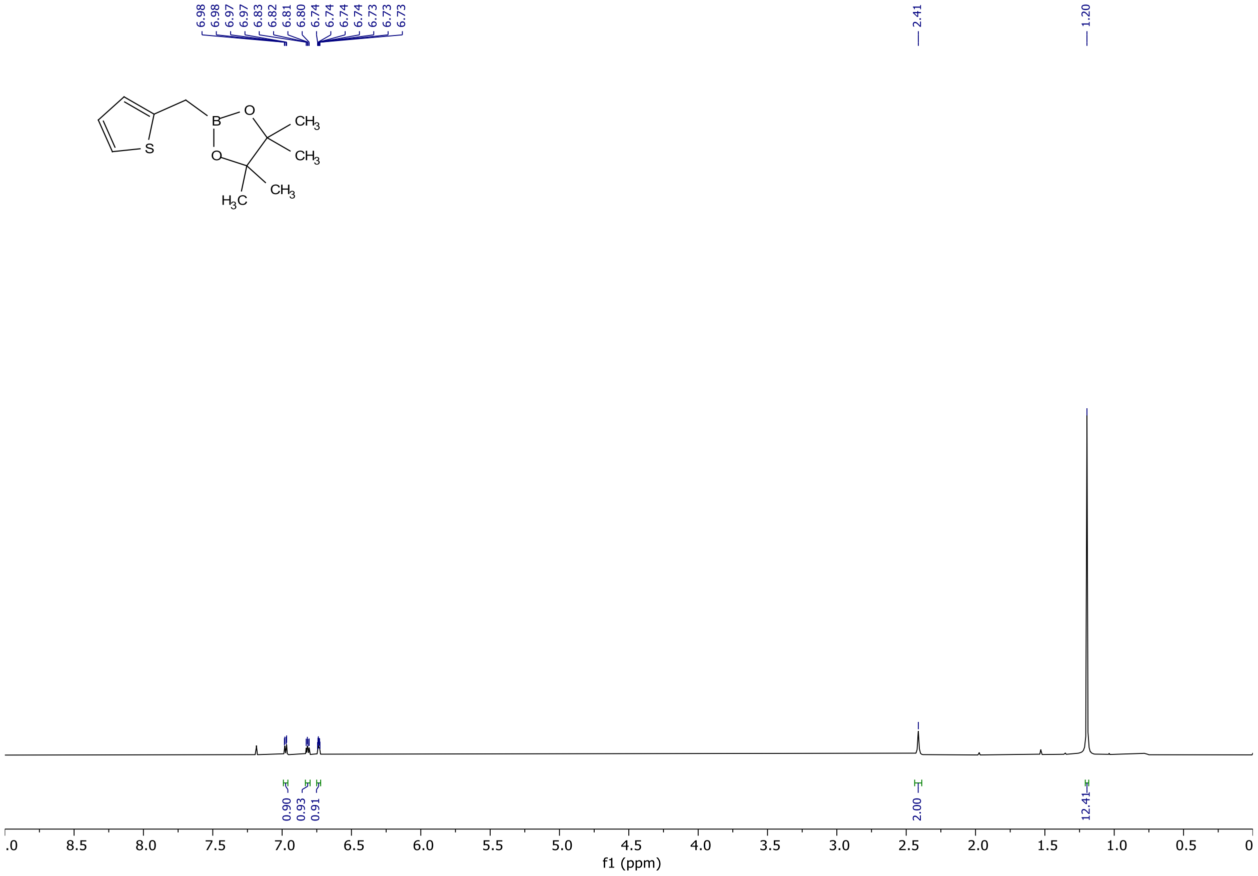

^{13}C NMR (101 MHz, $CDCl_3$)

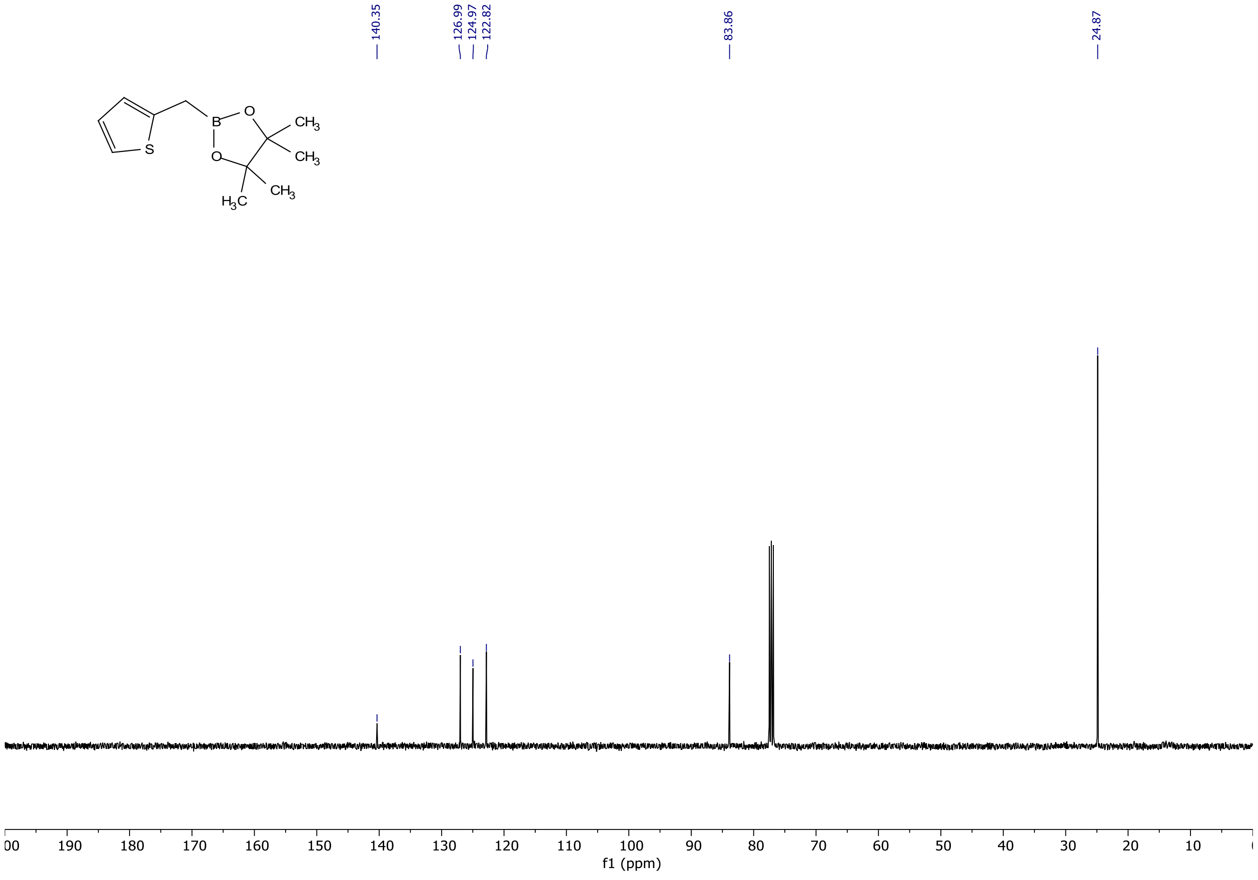

^{11}B NMR (128 MHz, $CDCl_3$)

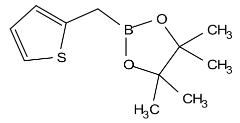

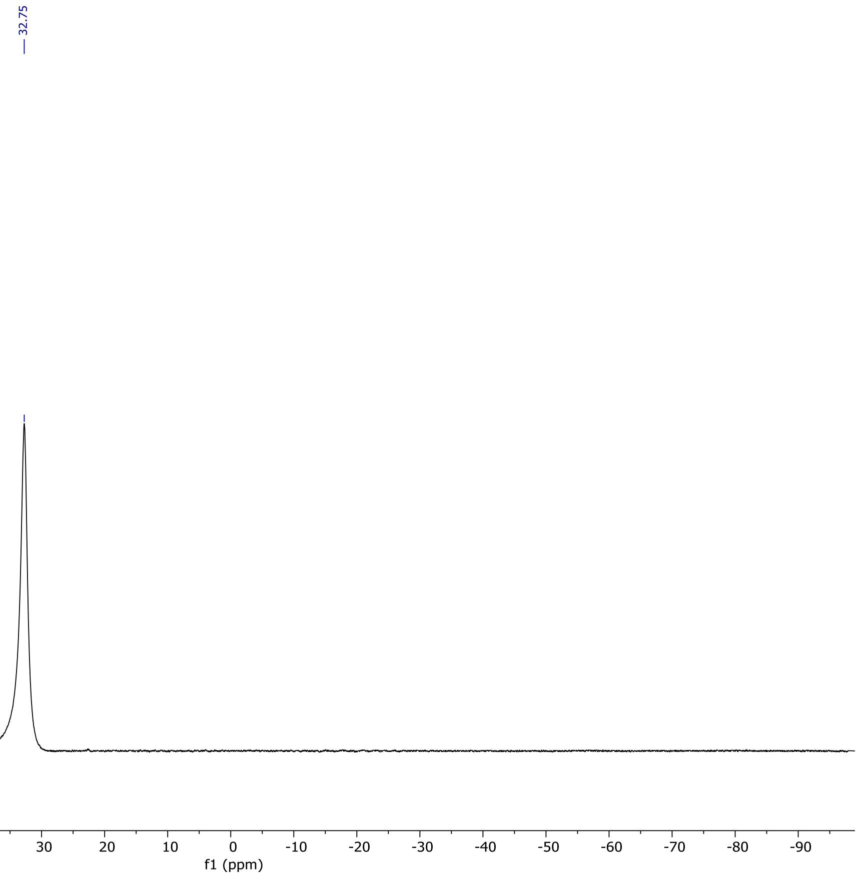

S10.54 **2n**

^{1}H NMR (400 MHz, $CDCl_3$)

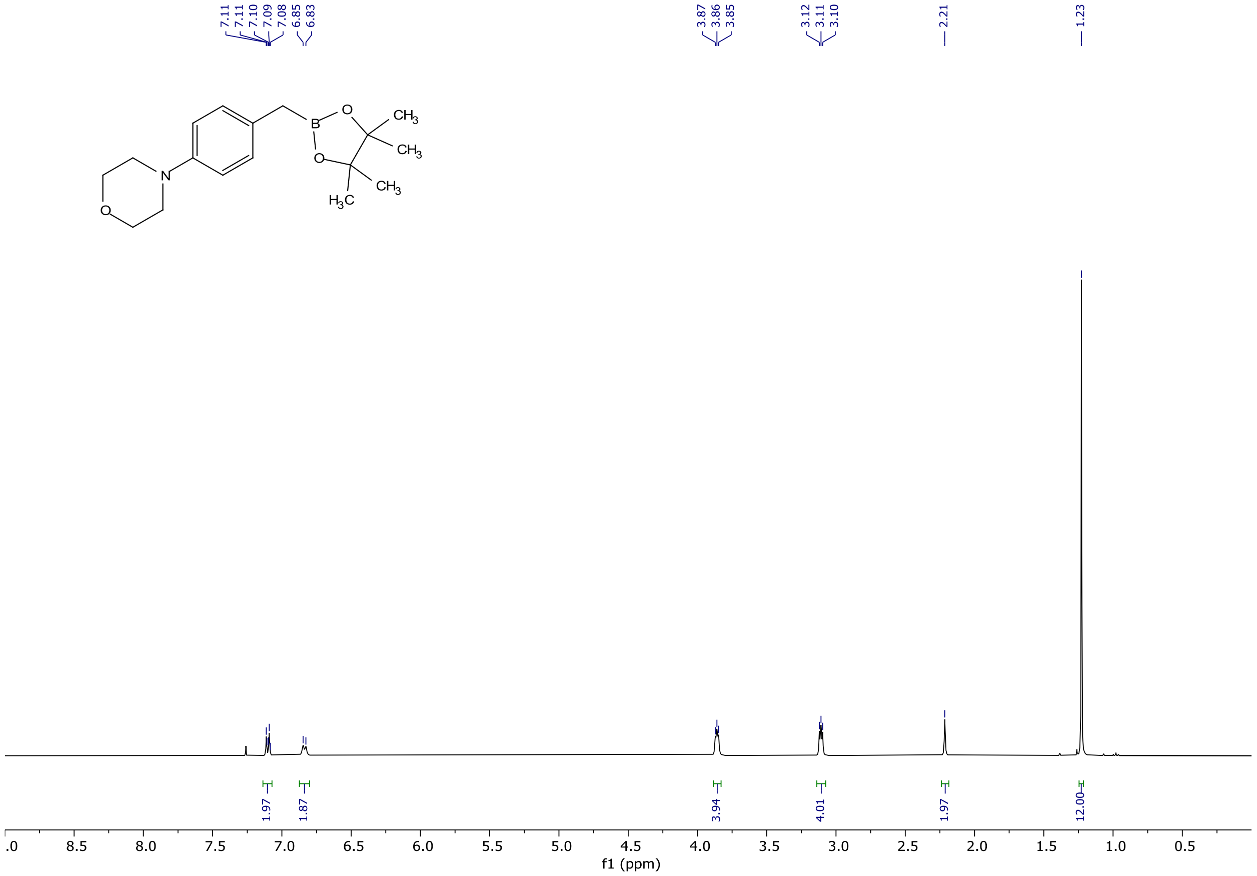

^{13}C NMR (101 MHz, $CDCl_3$)

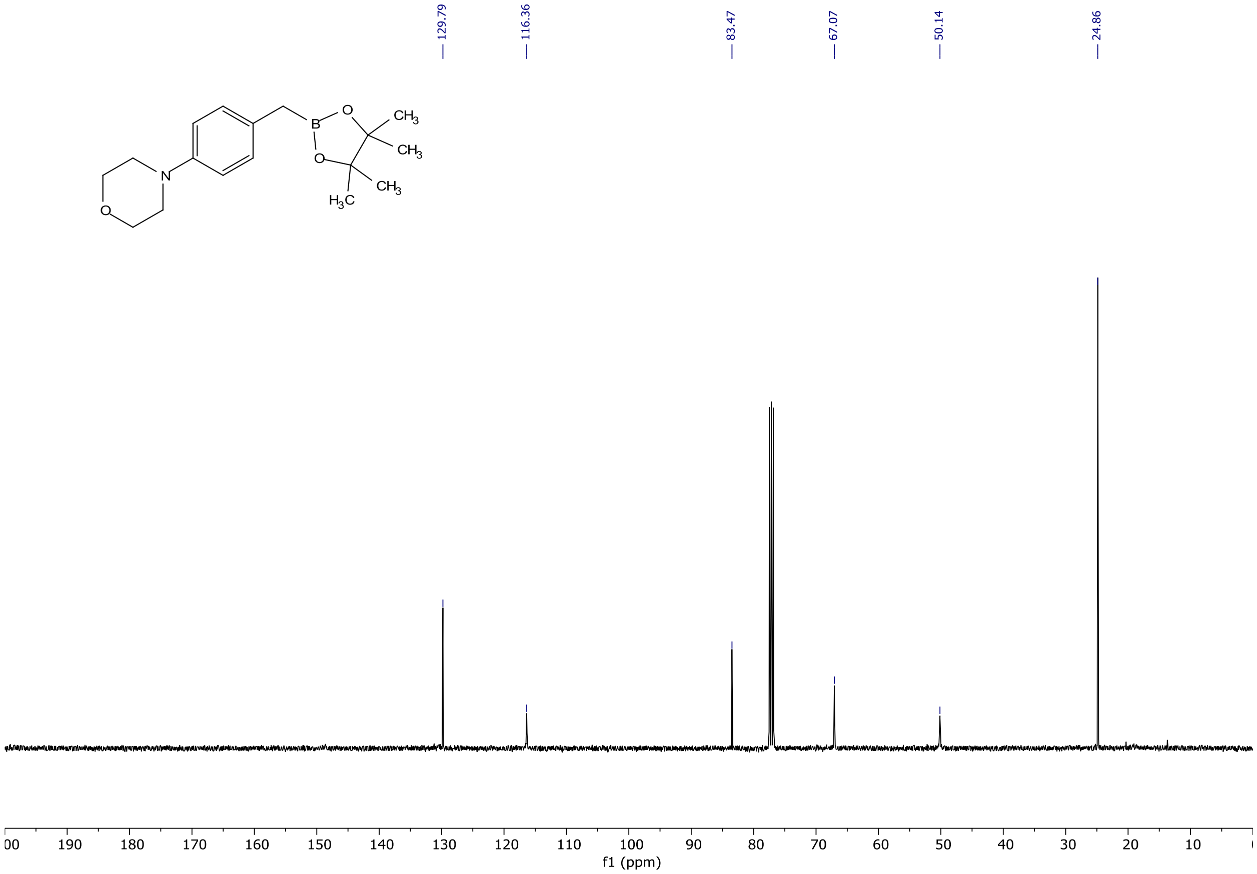

^{11}B NMR (128 MHz, $CDCl_3$)

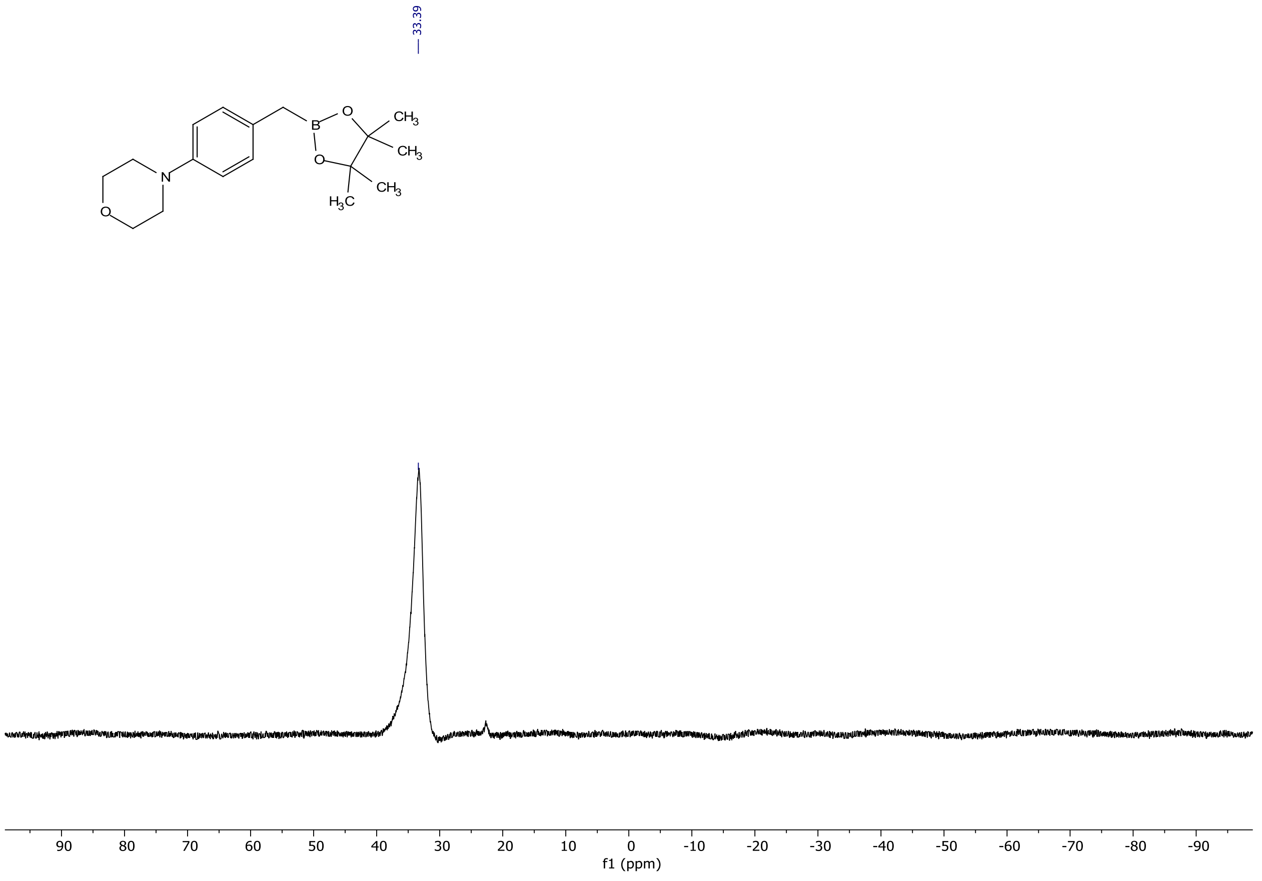

S10.55 2o

^1H NMR (400 MHz, $CDCl_3$)

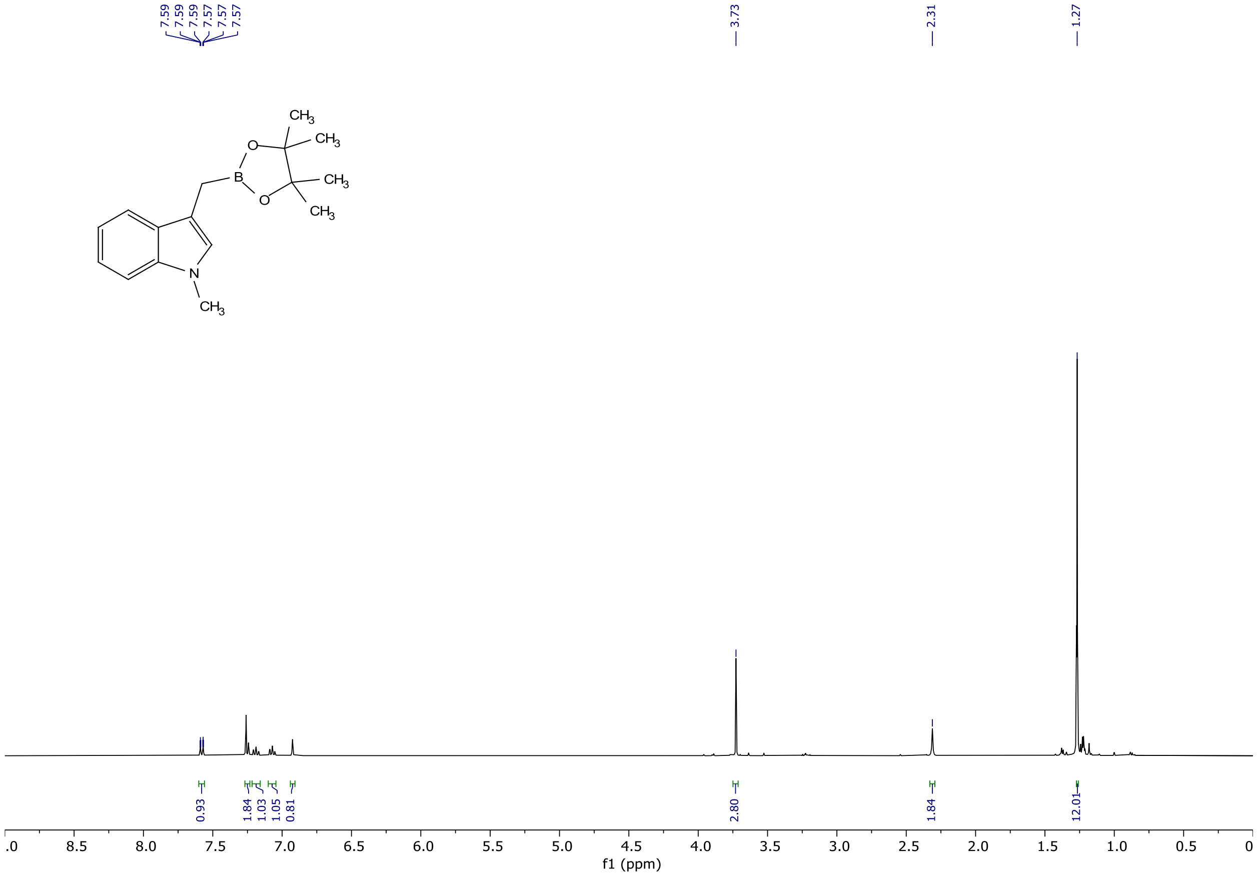

^{13}C NMR (101 MHz, $CDCl_3$)

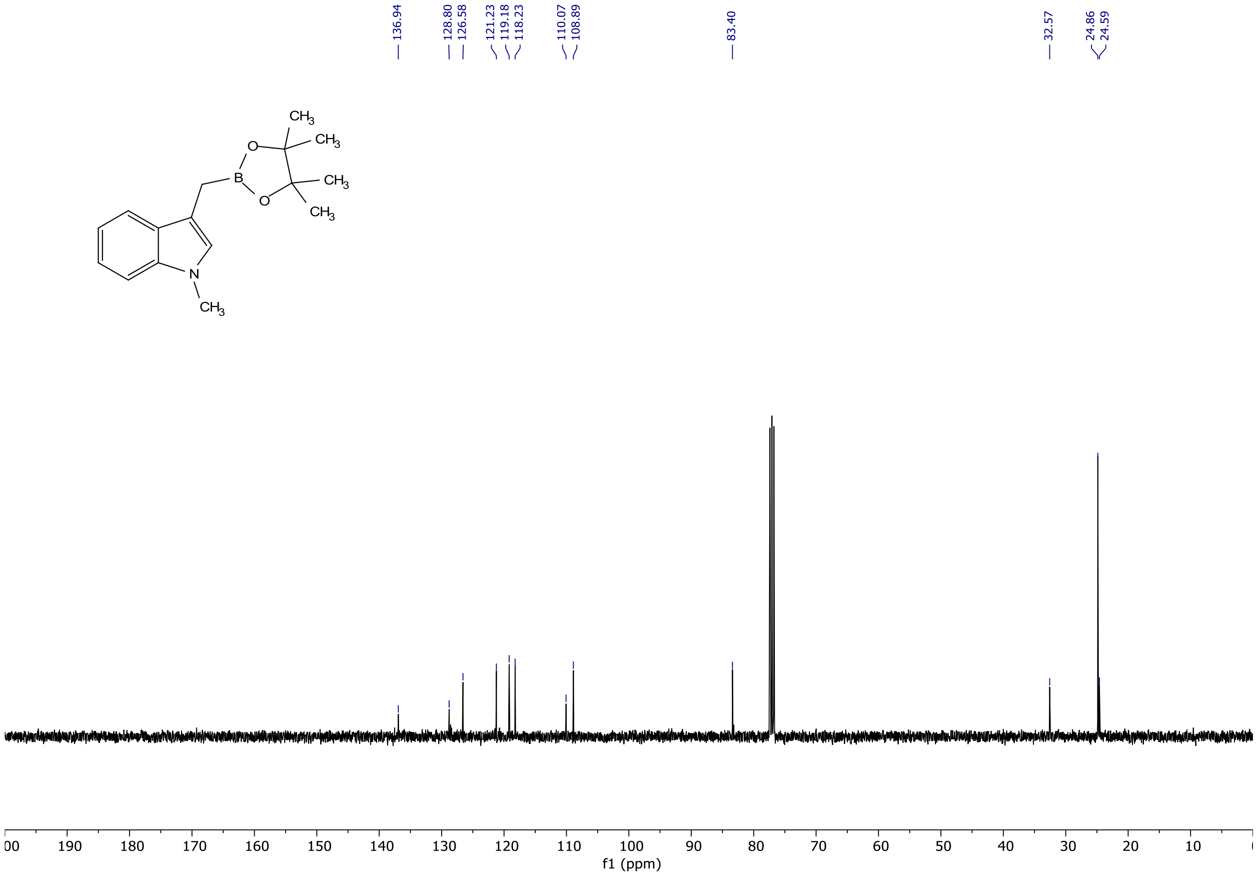

^{11}B NMR (128 MHz, $CDCl_3$)

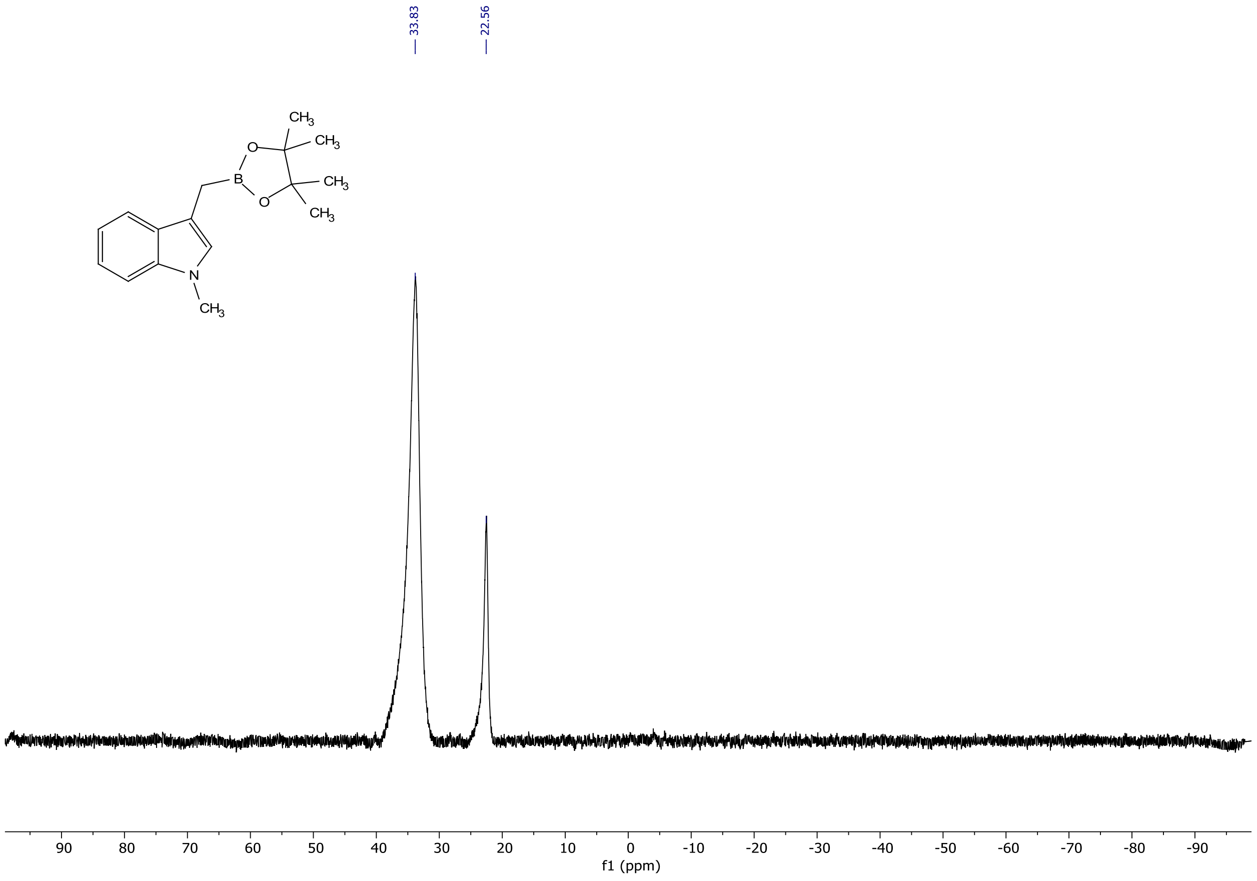

S10.56 2p

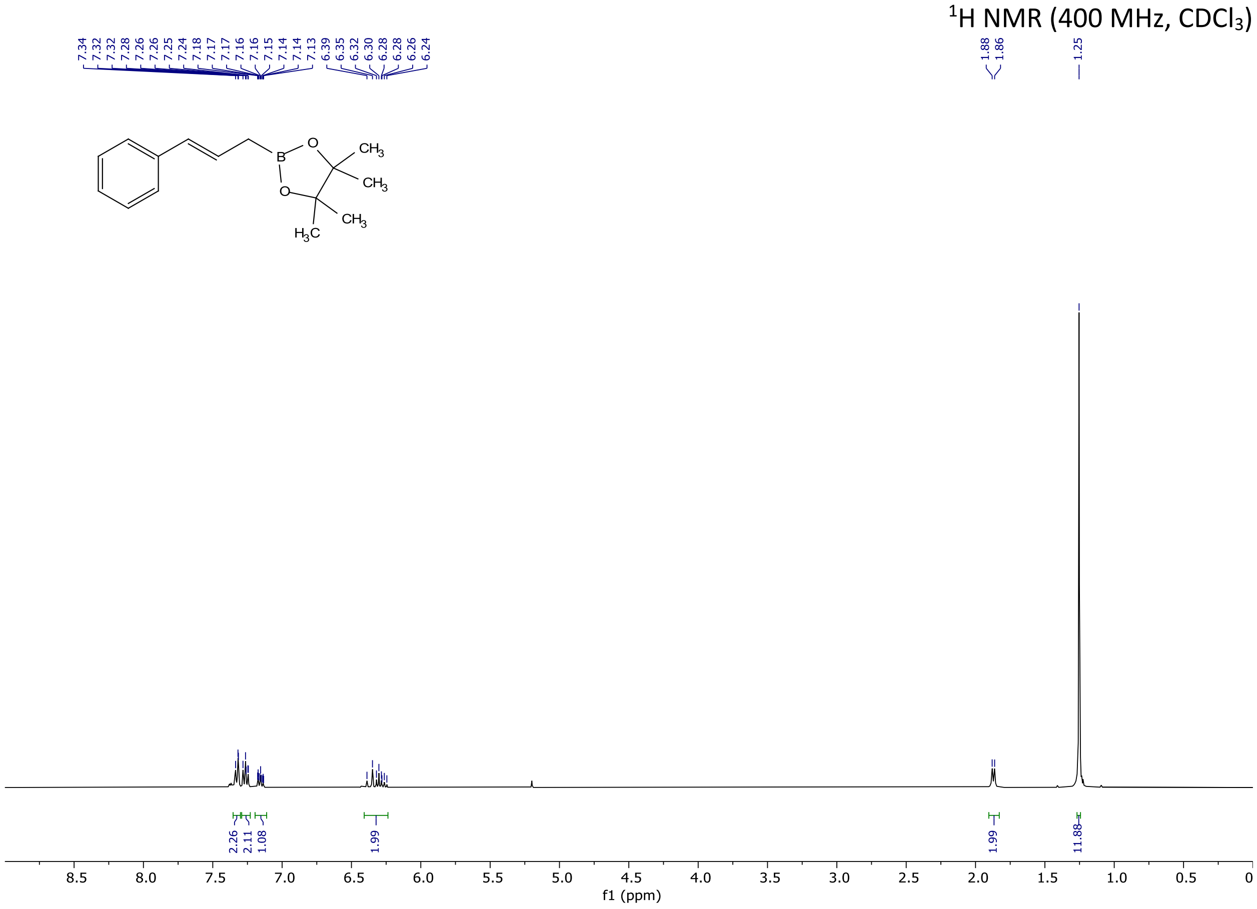

^{1}H NMR (400 MHz, $CDCl_3$)
7.34 7.32 7.32 7.28 7.26 7.26 7.25 7.24 7.18 7.17 7.17 7.16 7.16 7.15 7.14 7.14 7.13 6.39 6.35 6.32 6.30 6.28 6.28 6.26 6.24
1.88 1.86
1.25
CH3 CH3 CH3 H3C O O B
2.26 2.11 1.08 1.99 1.99 11.88
f1 (ppm)

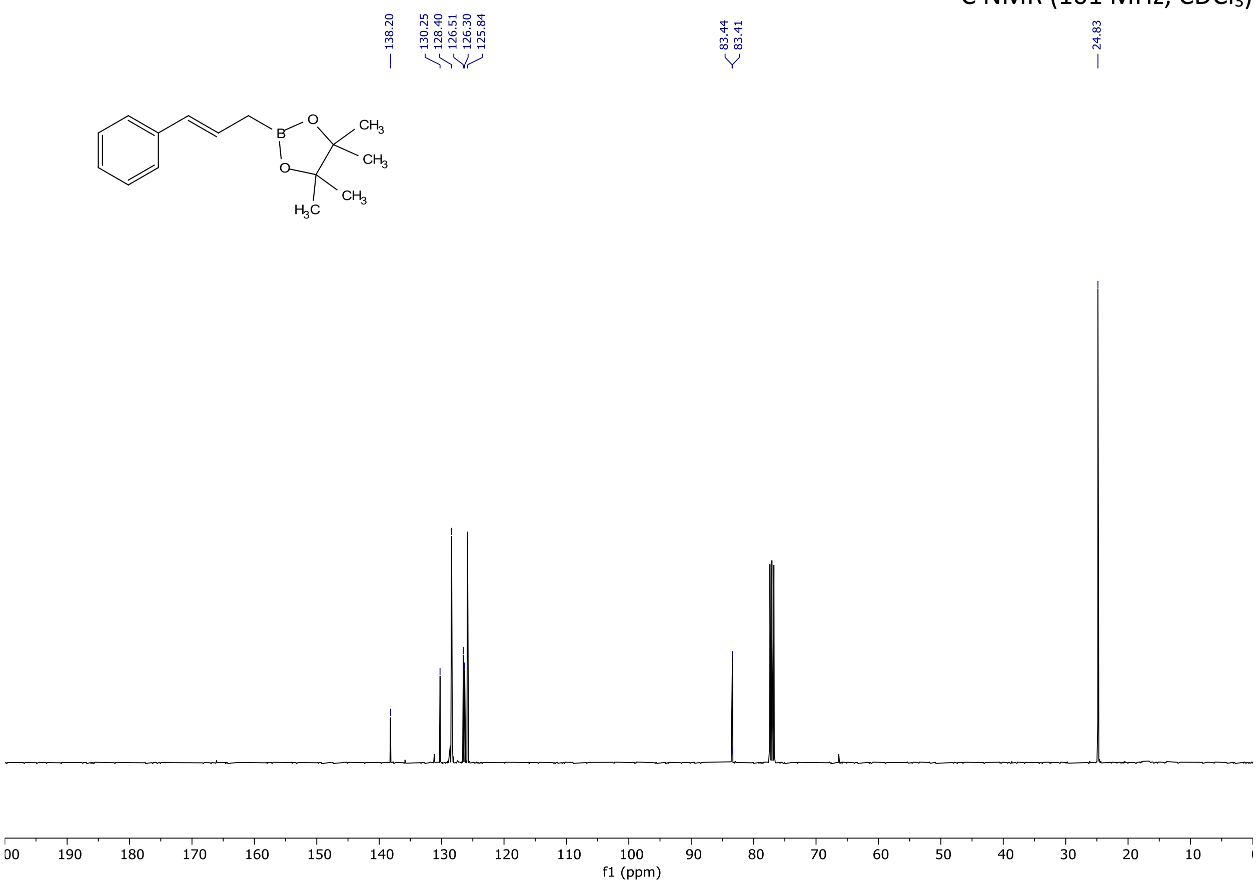

^{13}C NMR (101 MHz, $CDCl_3$)
138.20
130.25 128.40 126.51 126.30 125.84
83.44 83.41
24.83
CH3 CH3 CH3 H3C O O B
f1 (ppm)

^{11}B NMR (128 MHz, $CDCl_3$)

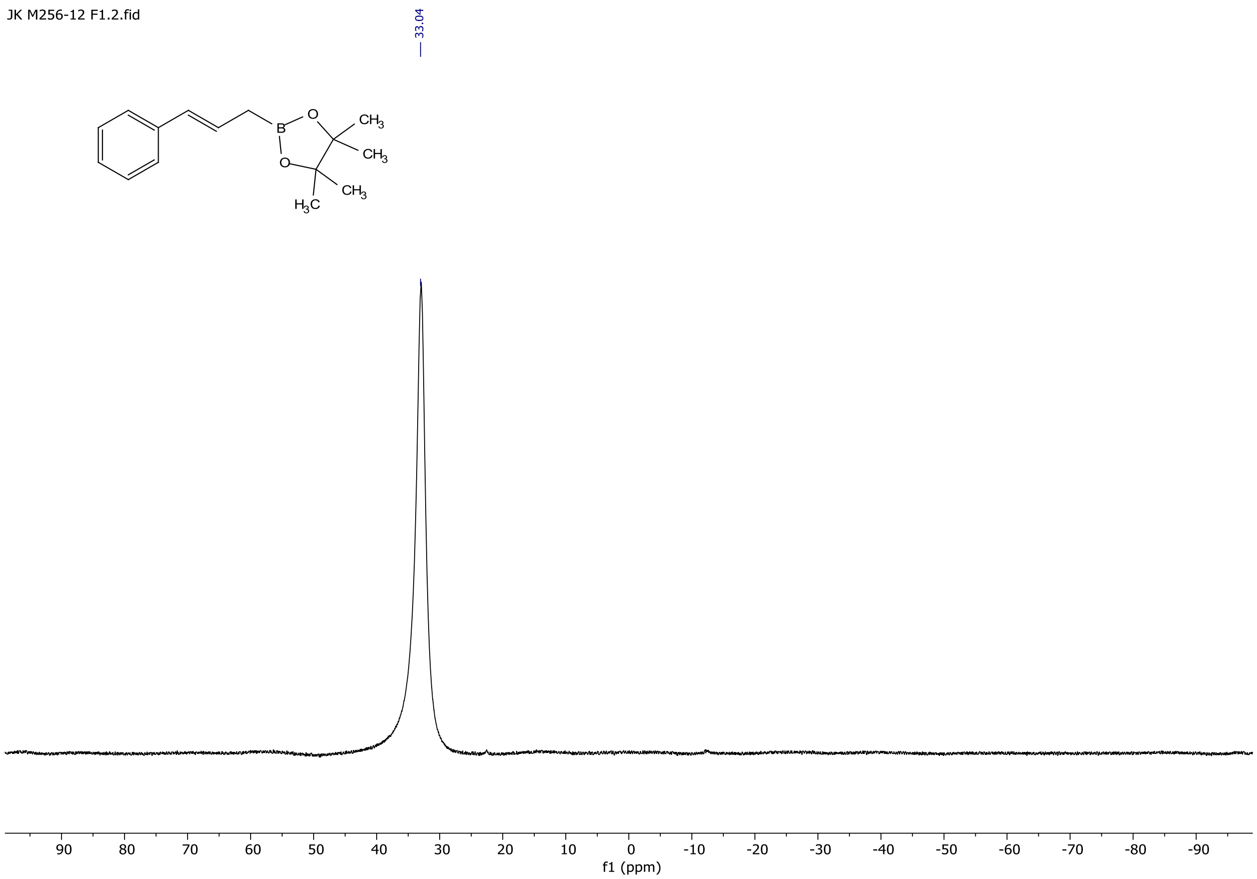

S10.57 **2q**

^{1}H NMR (400 MHz, $CDCl_3$)

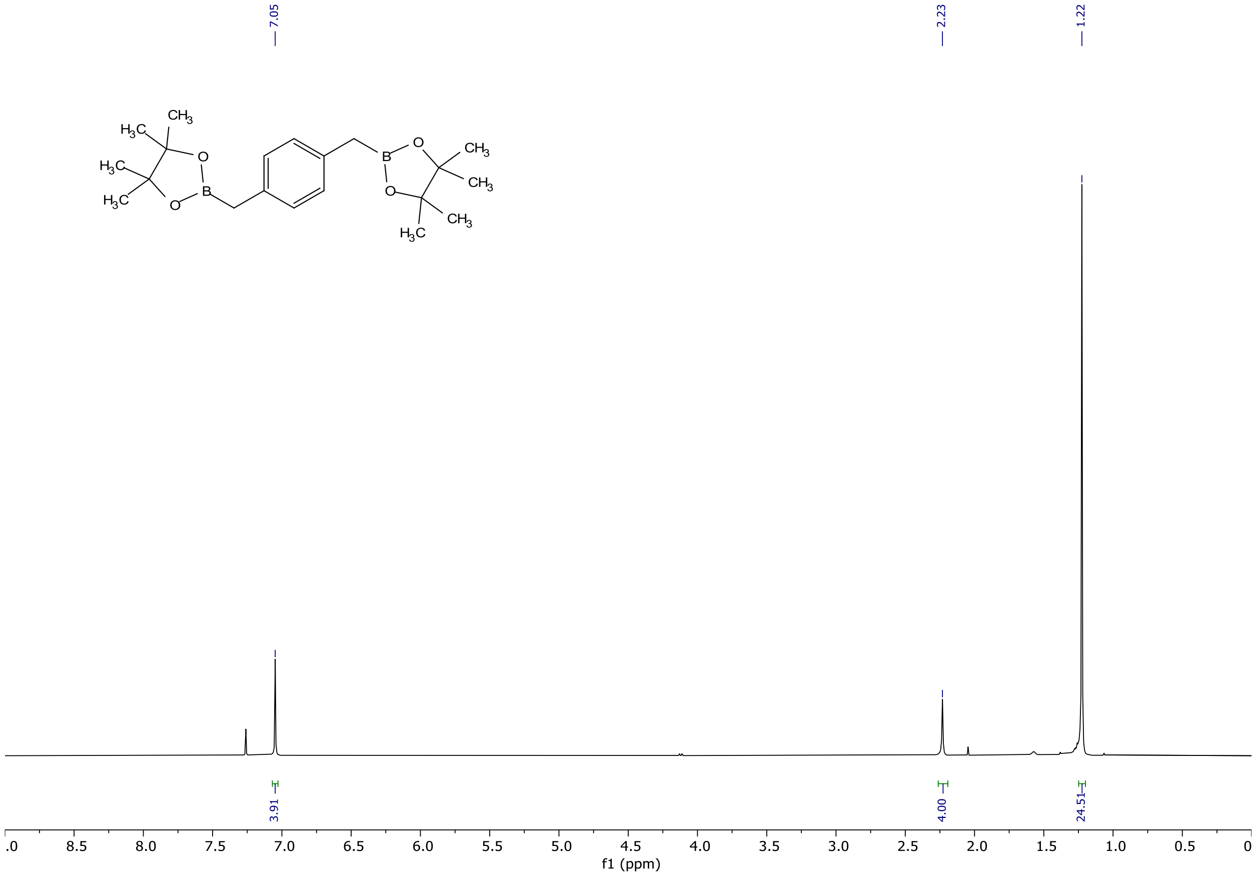

^{13}C NMR (101 MHz, $CDCl_3$)

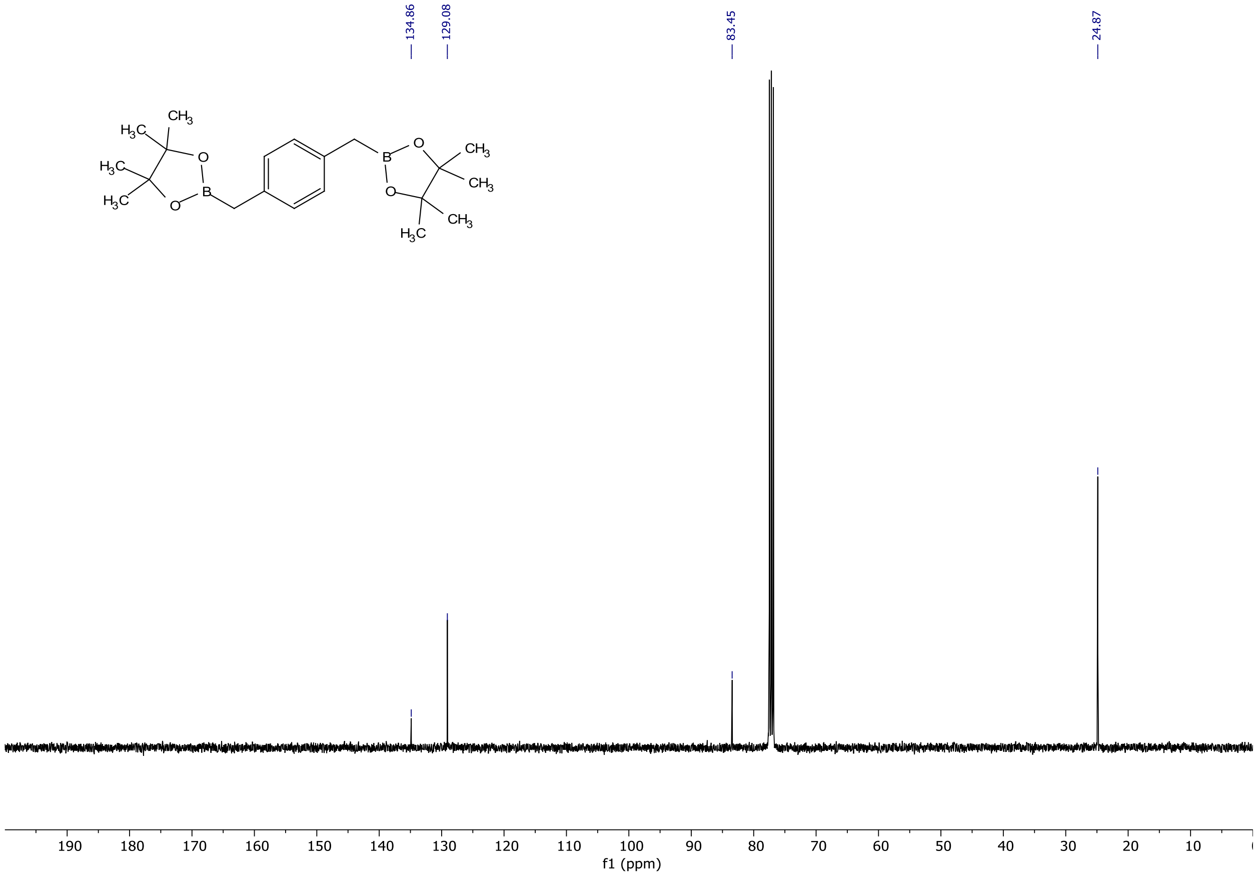

^{11}B NMR (128 MHz, $CDCl_3$)

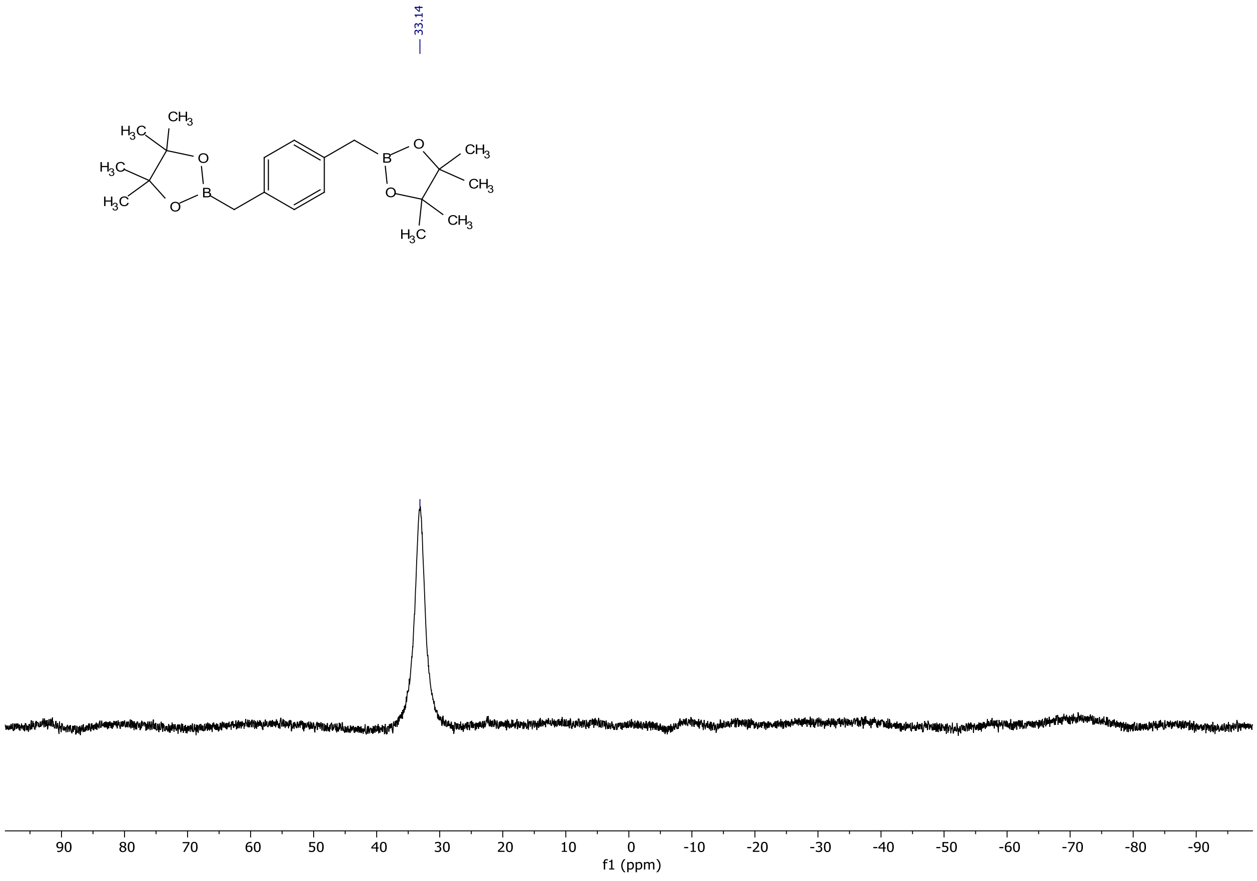

S10.59 2r

^{1}H NMR (400 MHz, $CDCl_3$)

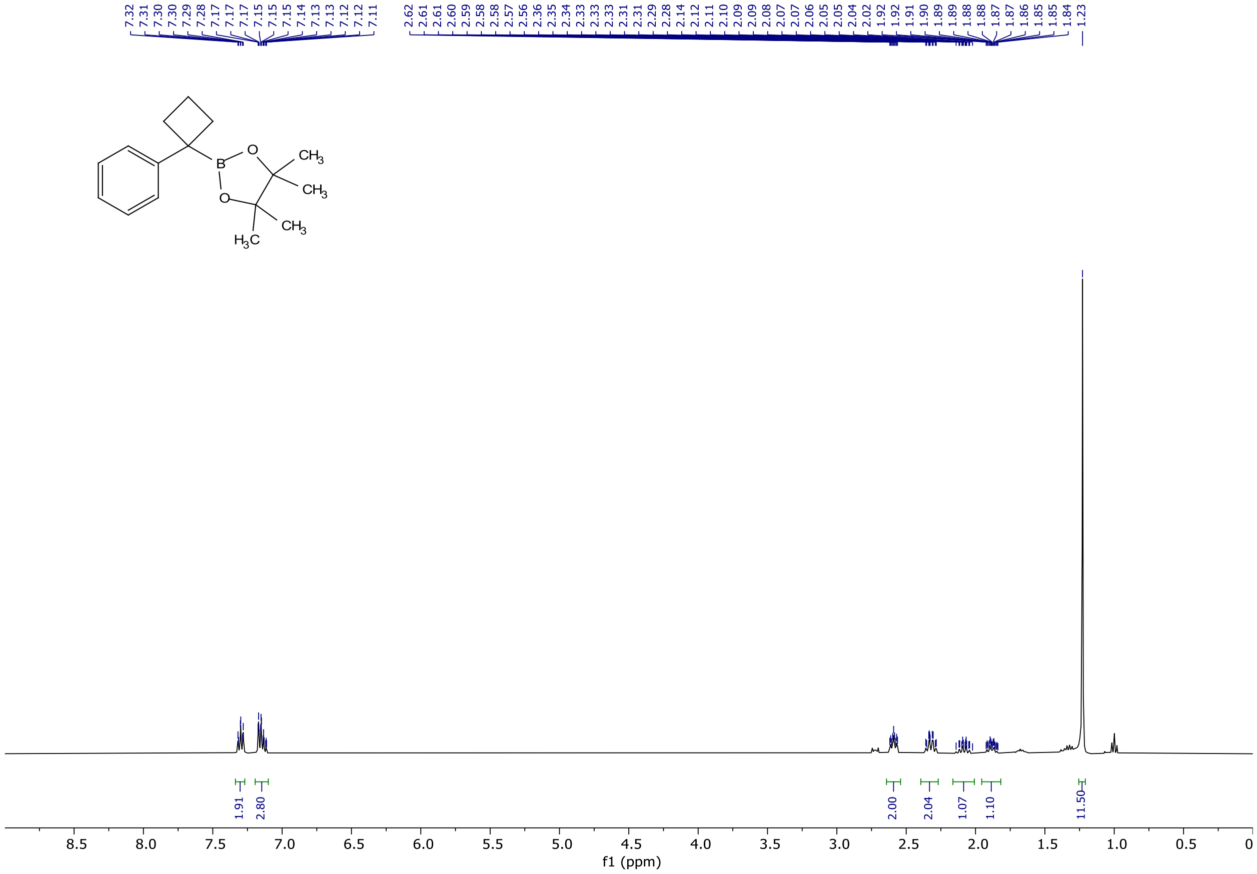

^{13}C NMR (101 MHz, $CDCl_3$)

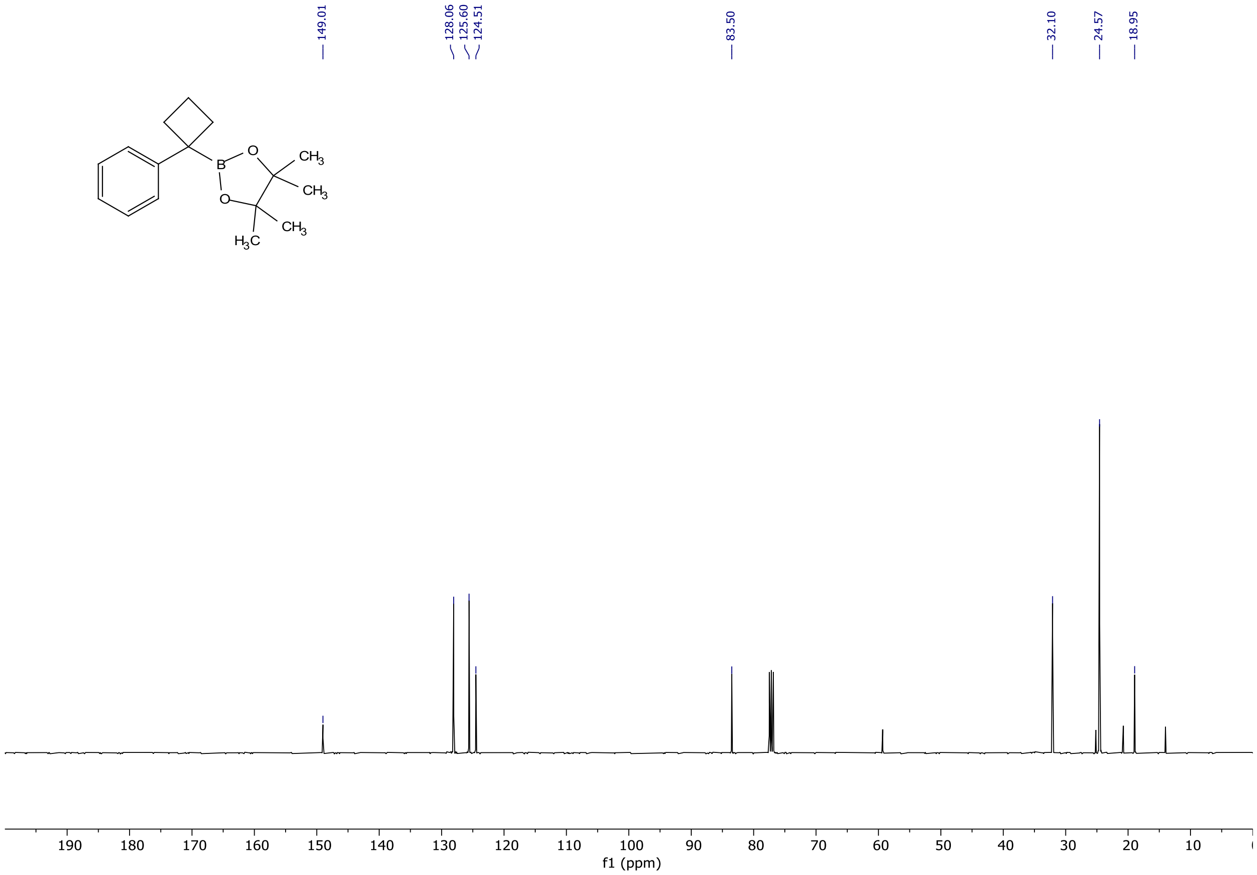

^{11}B NMR (128 MHz, $CDCl_3$)

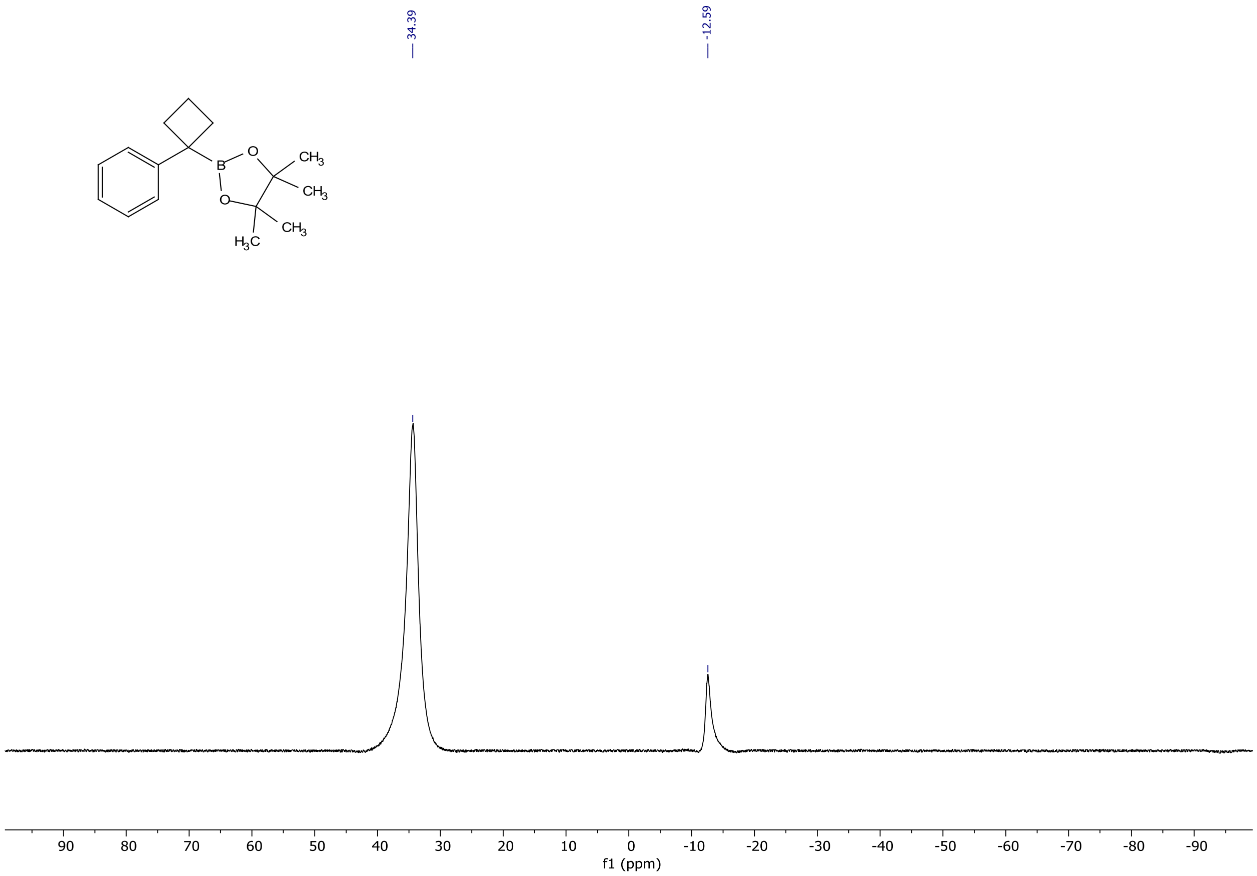

S10.60 2s

^{1}H NMR (400 MHz, $CDCl_3$)

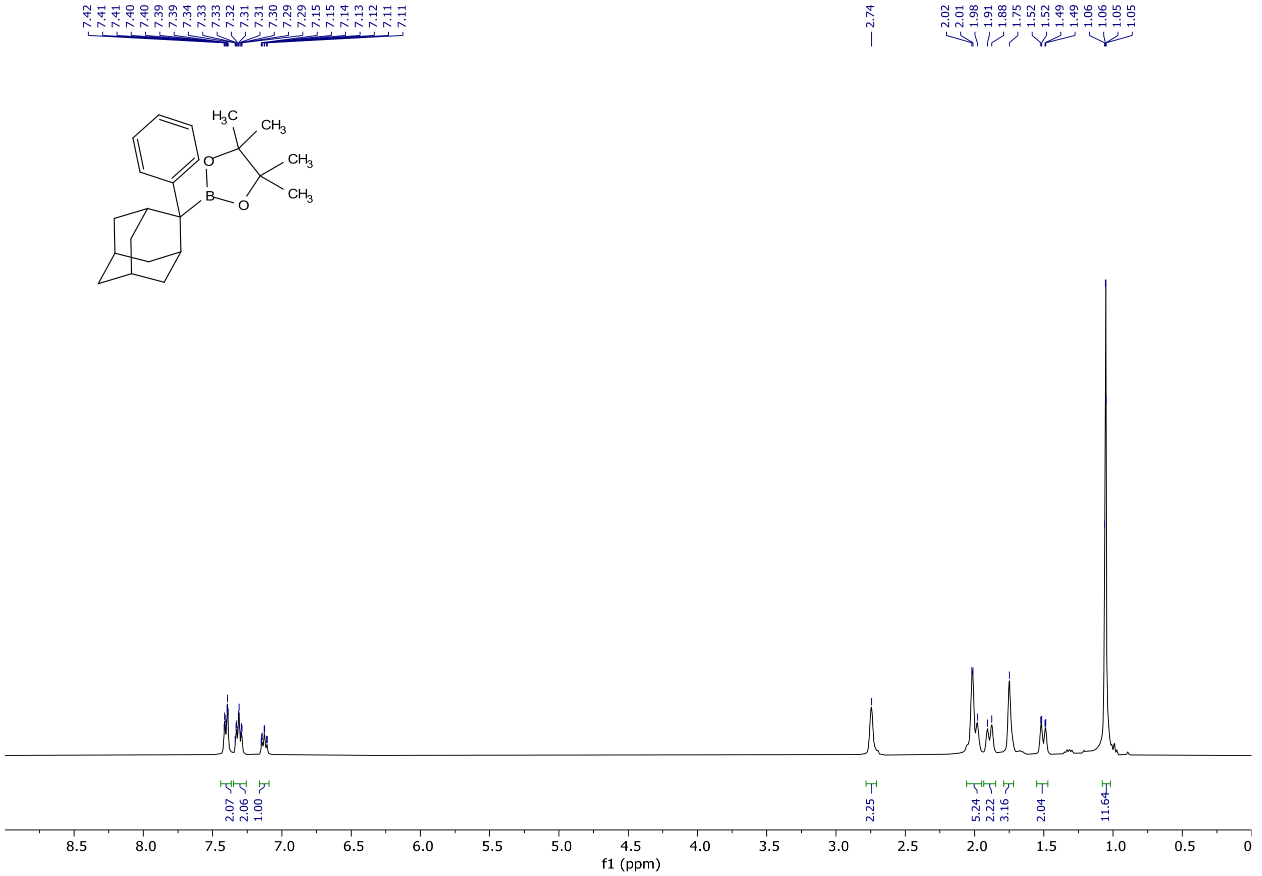

^{13}C NMR (101 MHz, $CDCl_3$)

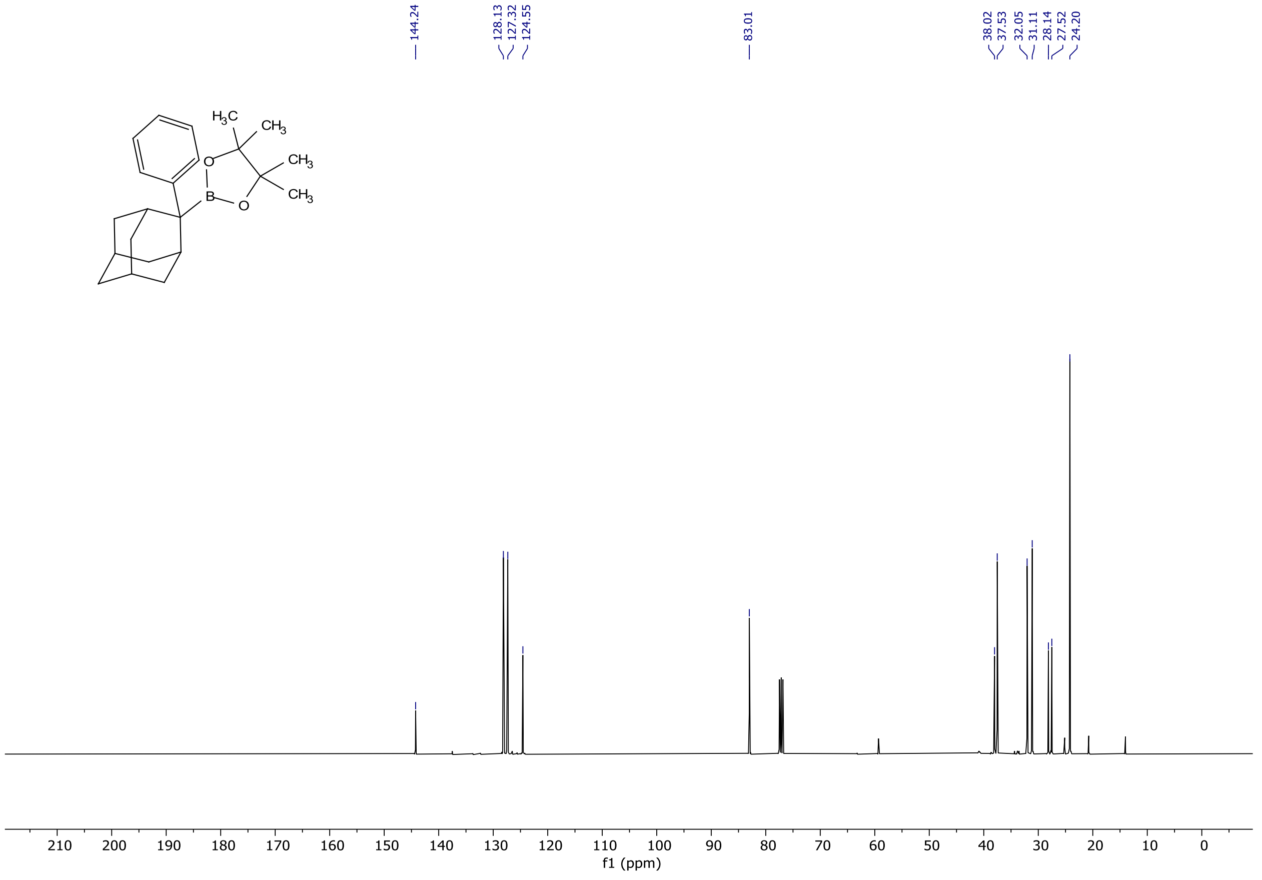

^{11}B NMR (128 MHz, CDCl3)

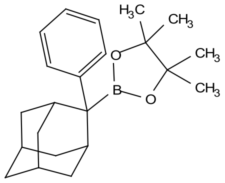

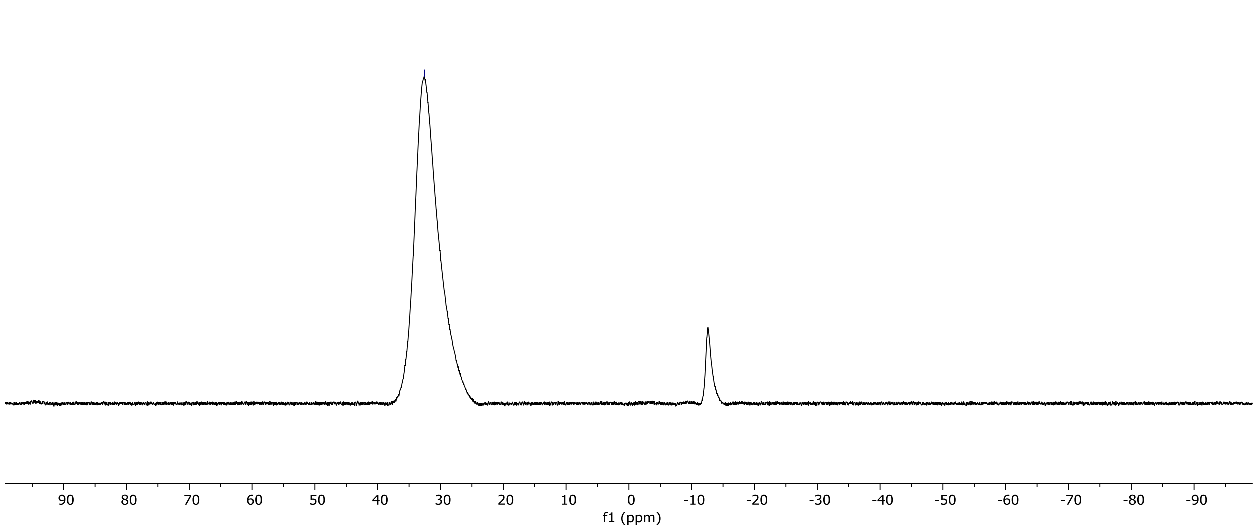

S10.62 2t

^{1}H NMR (400 MHz, $CDCl_3$)

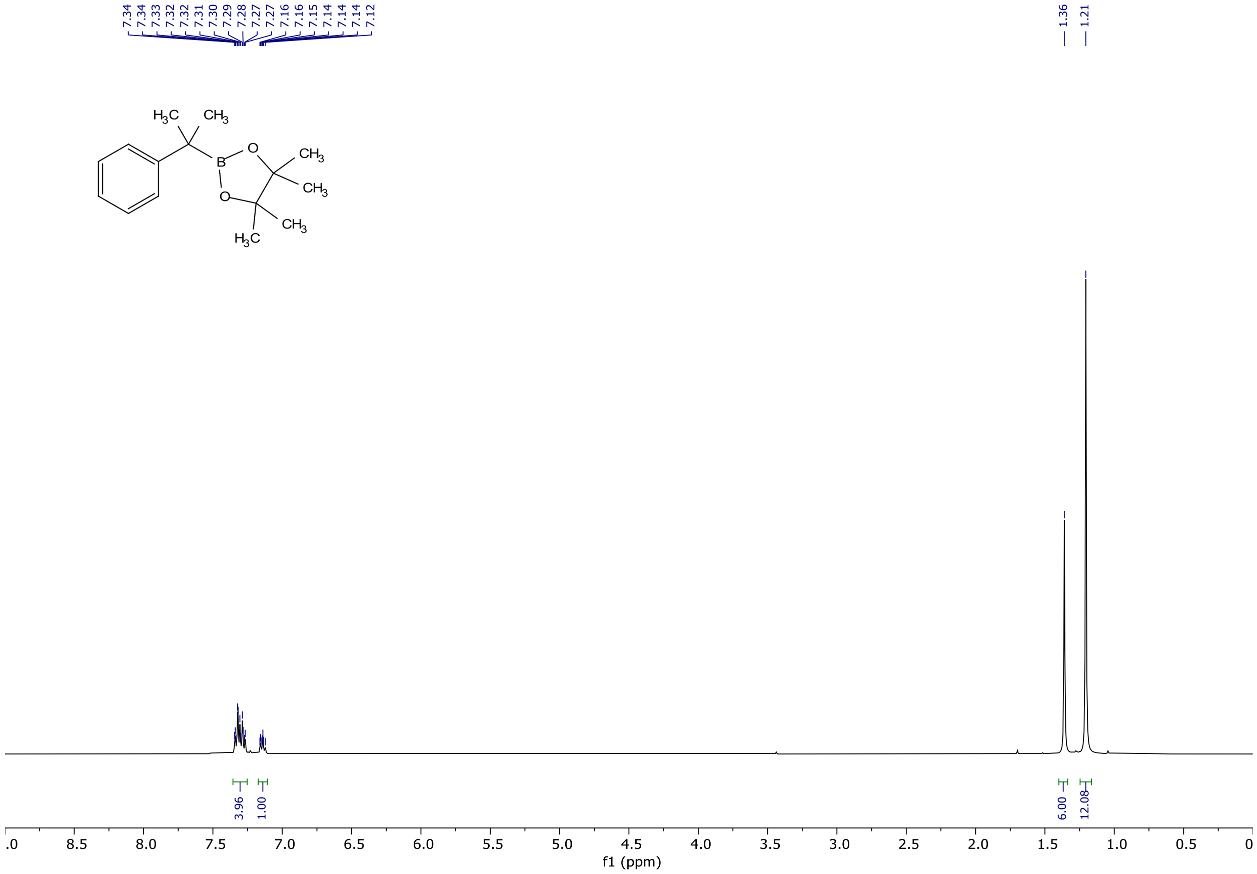

^{13}C NMR (101 MHz, $CDCl_3$)

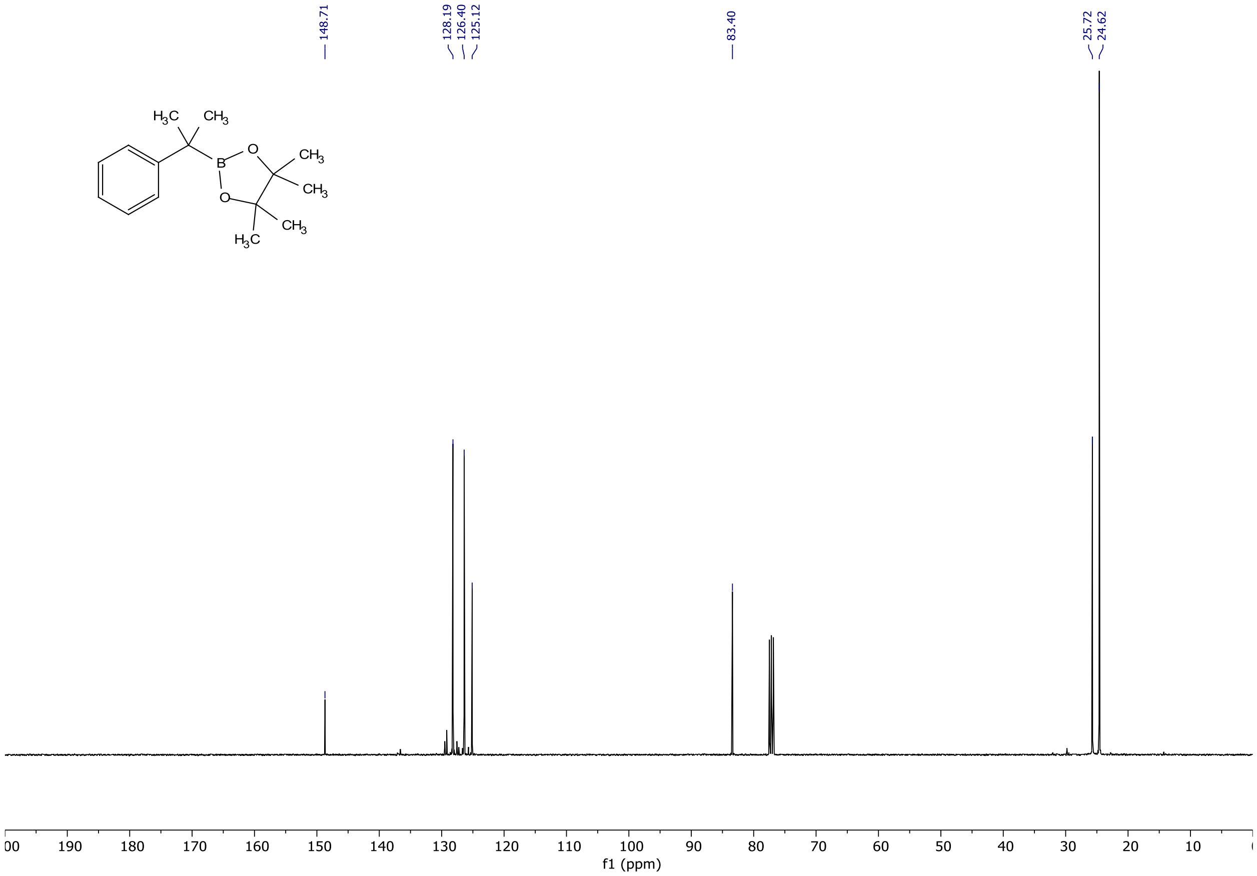

^{11}B NMR (128 MHz, $CDCl_3$)

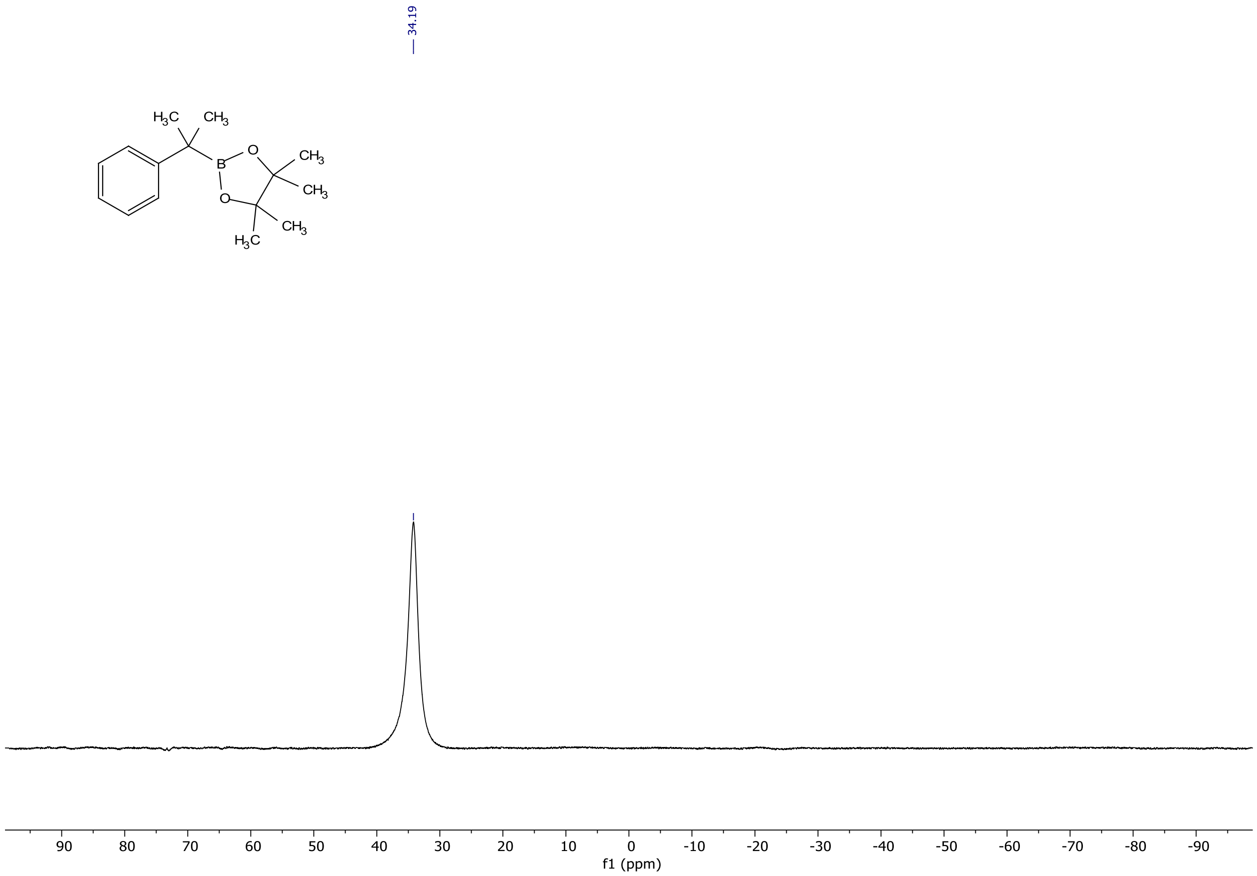

S10.63 2u

^{1}H NMR (500 MHz, $CDCl_3$)

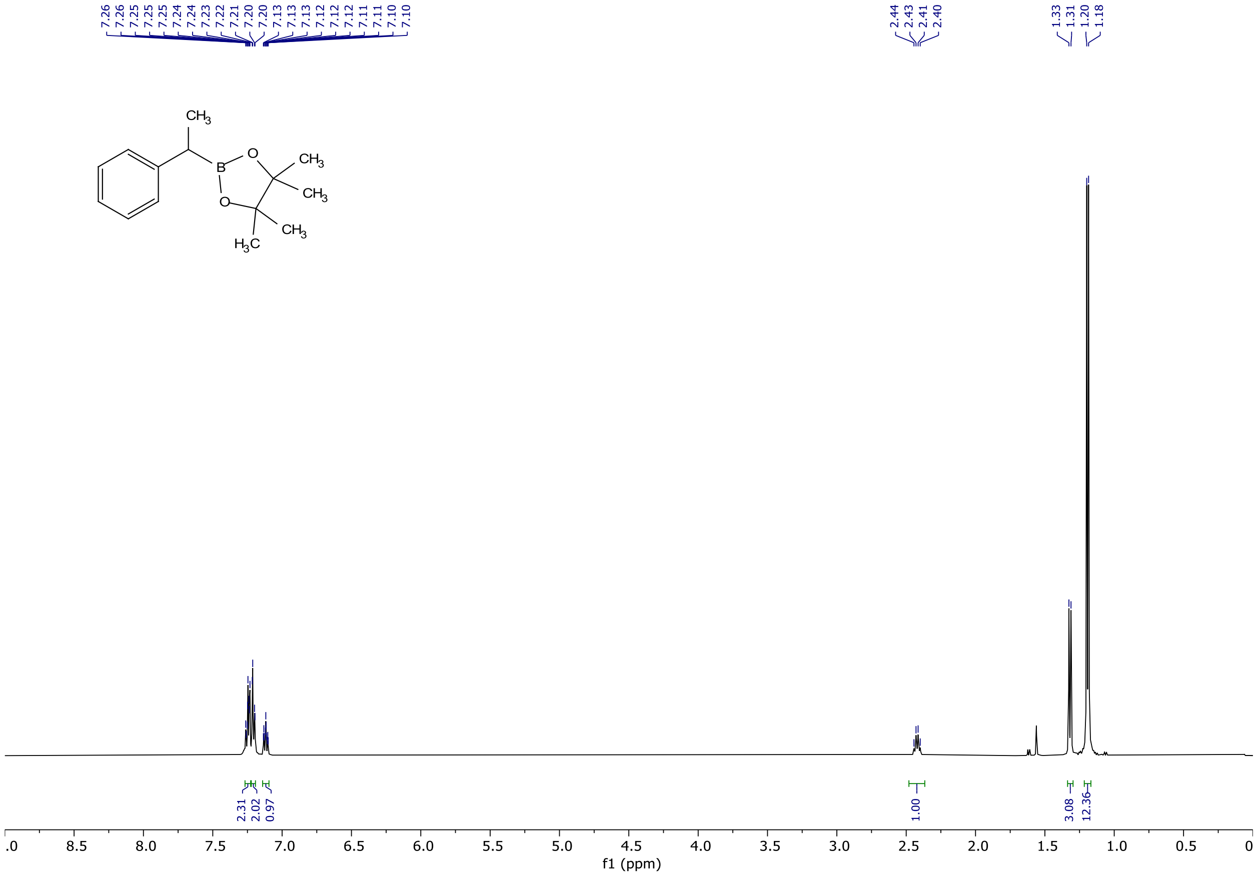

^{13}C NMR (101 MHz, $CDCl_3$)

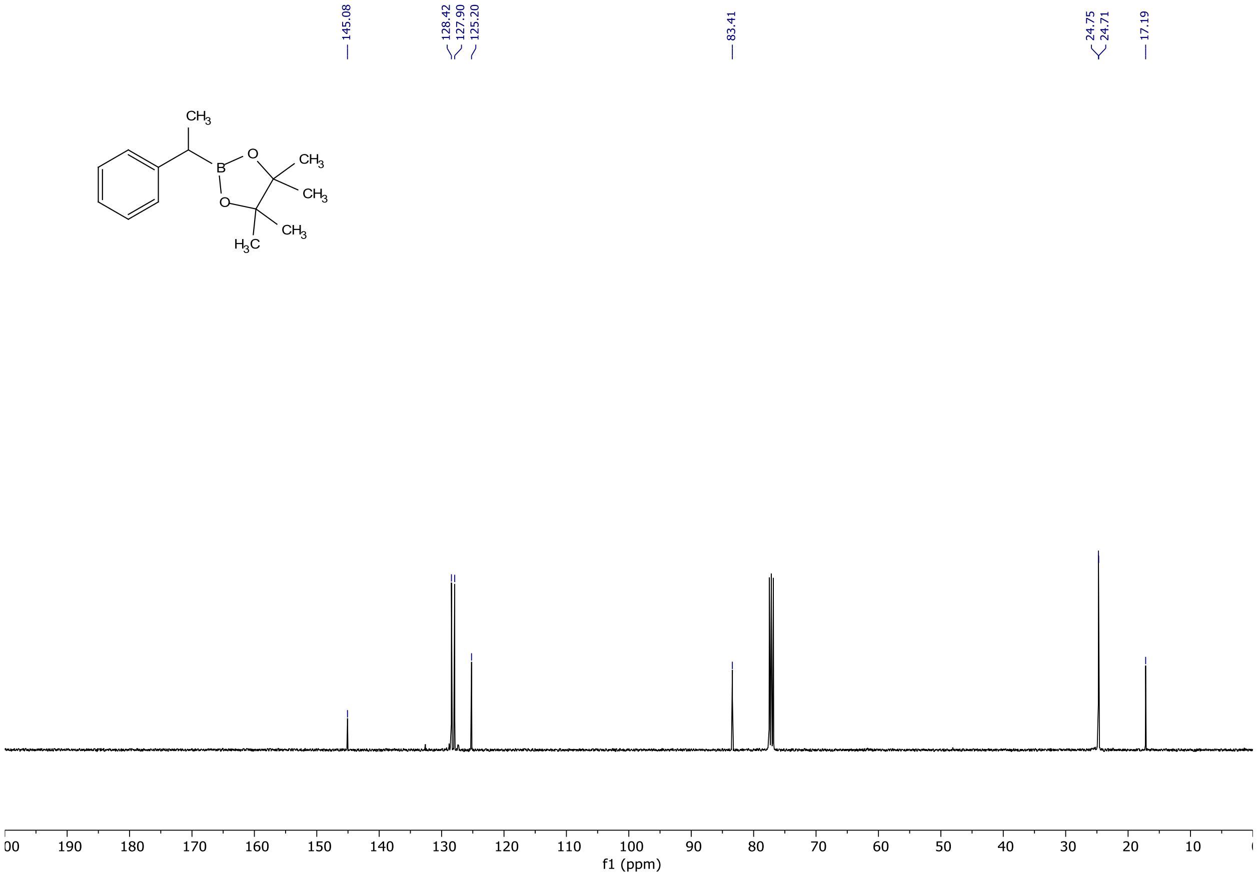

^{11}B NMR (128 MHz, $CDCl_3$)

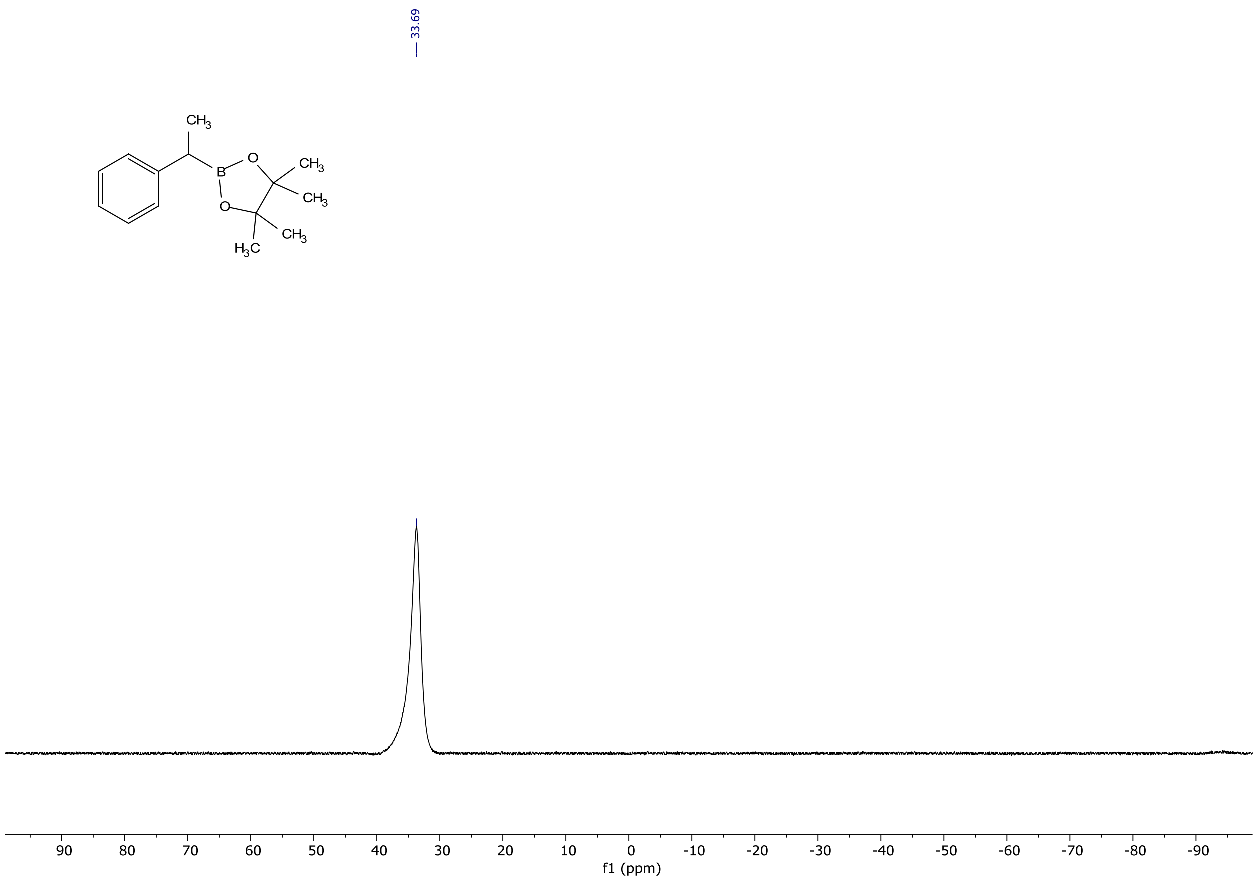

S10.64 2v

^1H NMR (400 MHz, $CDCl_3$)

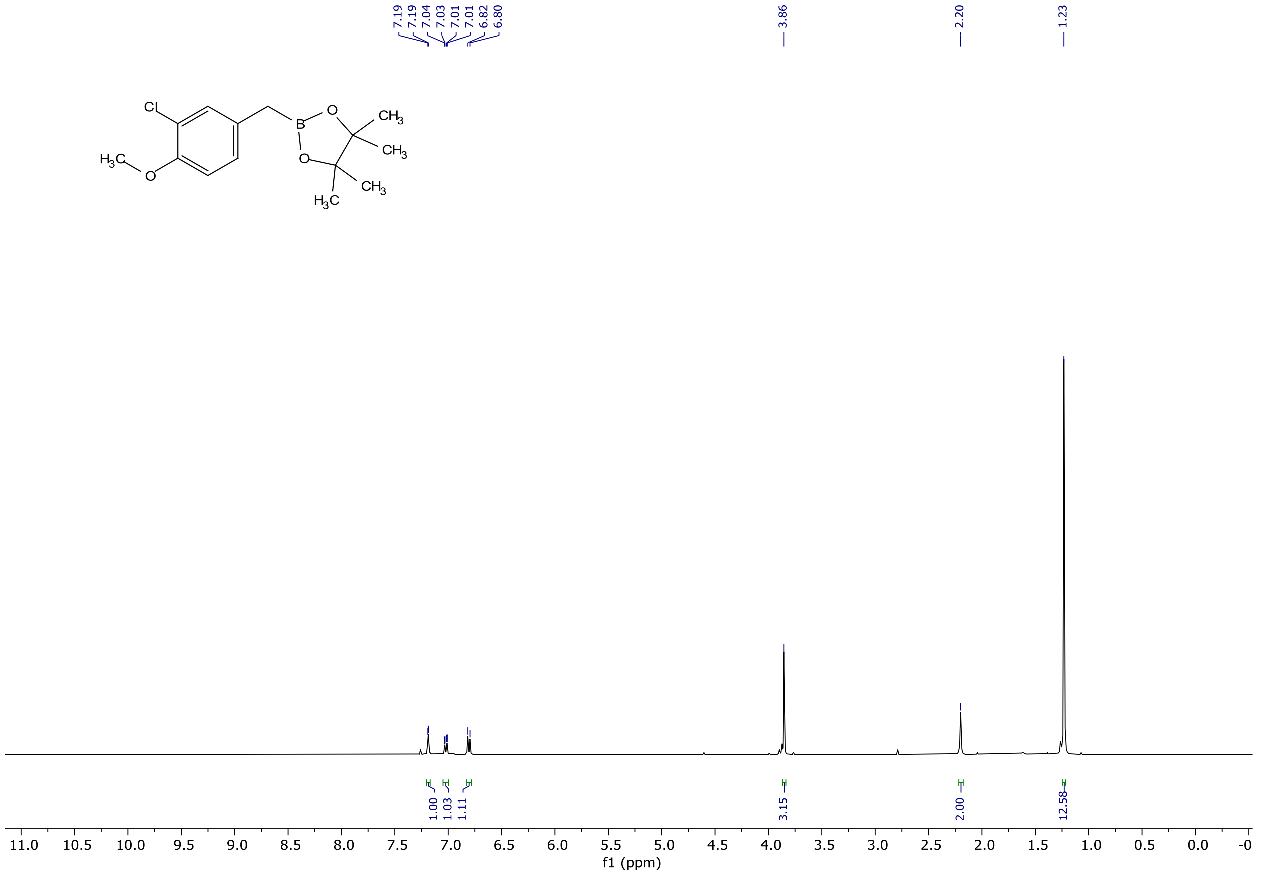

^{13}C NMR (126 MHz, $CDCl_3$)

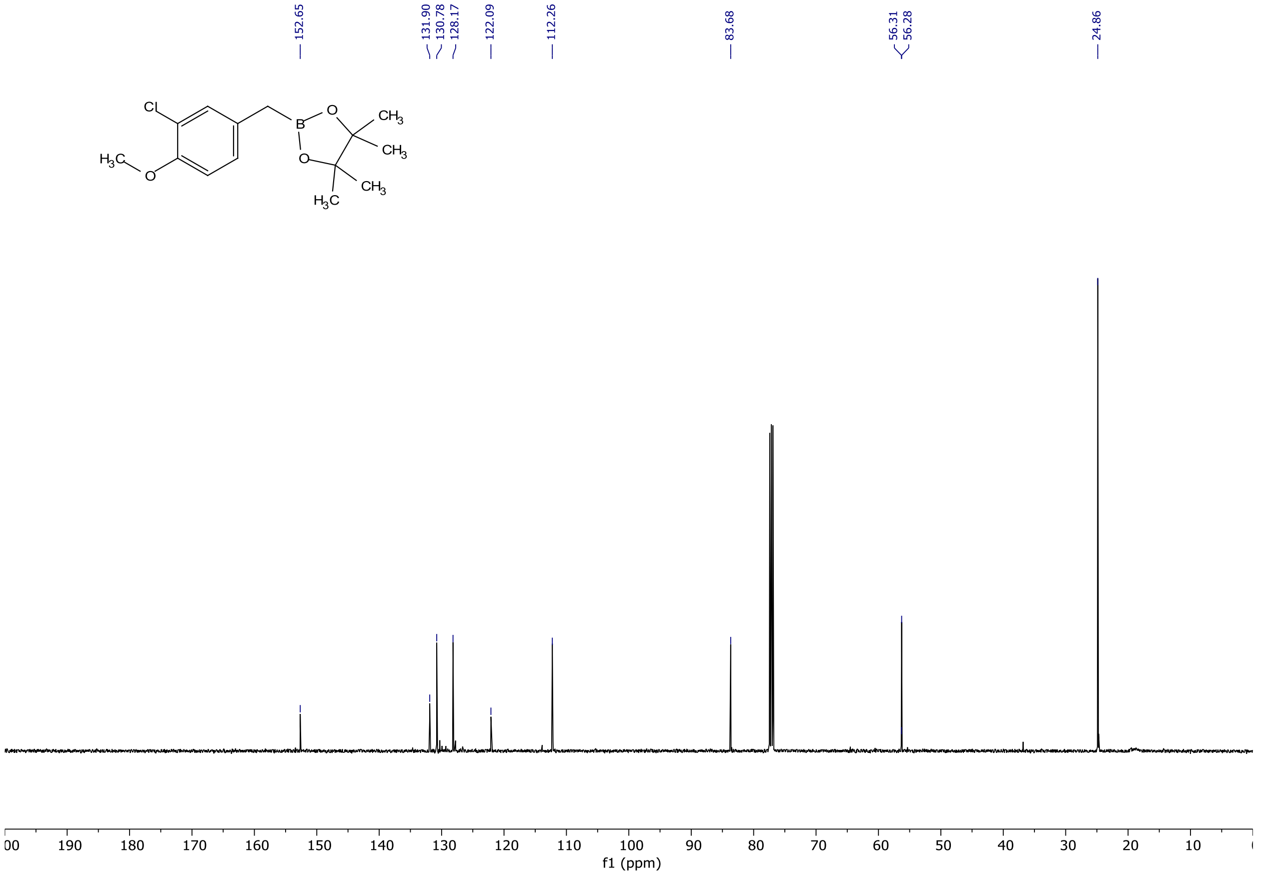

^{11}B NMR (160 MHz, $CDCl_3$)

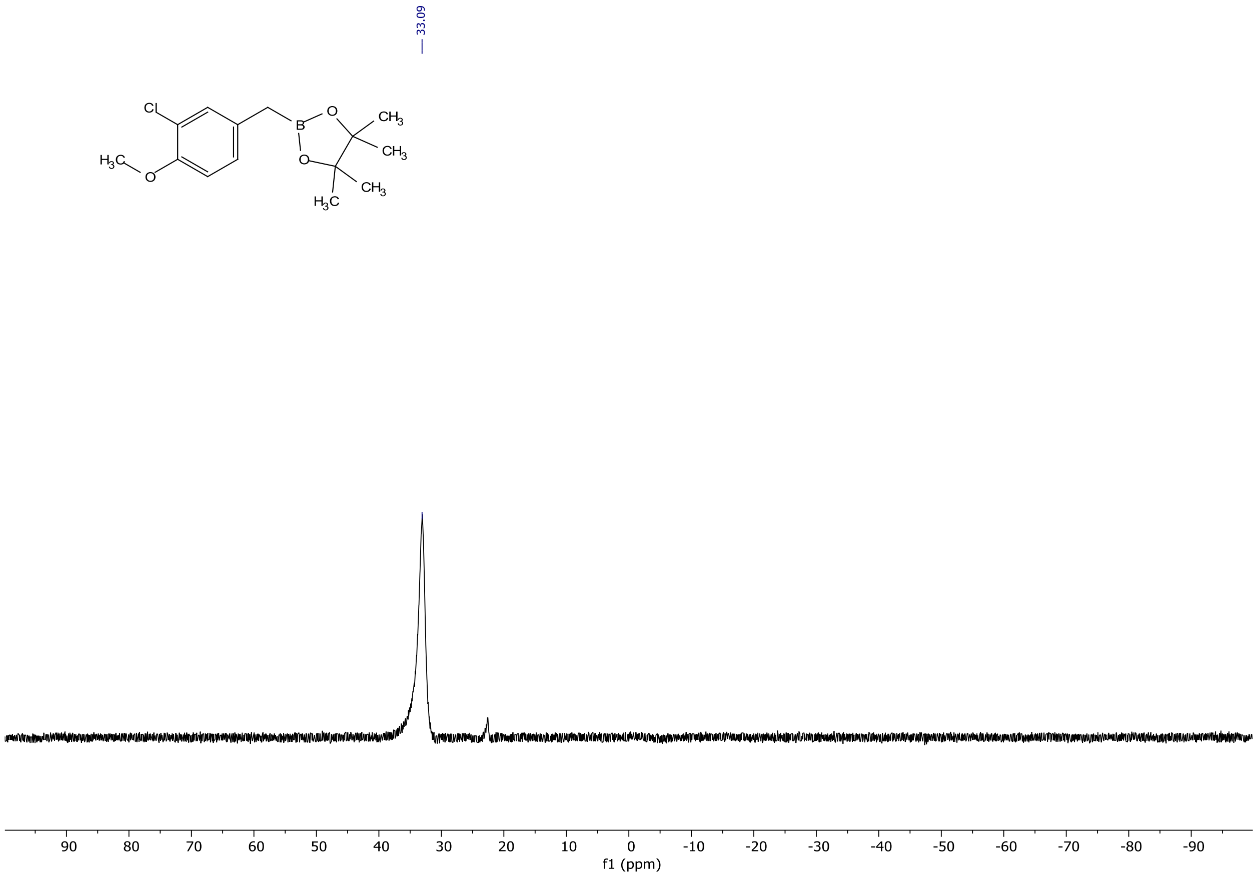

S10.65 2w

^{1}H NMR (500 MHz, $CDCl_3$)

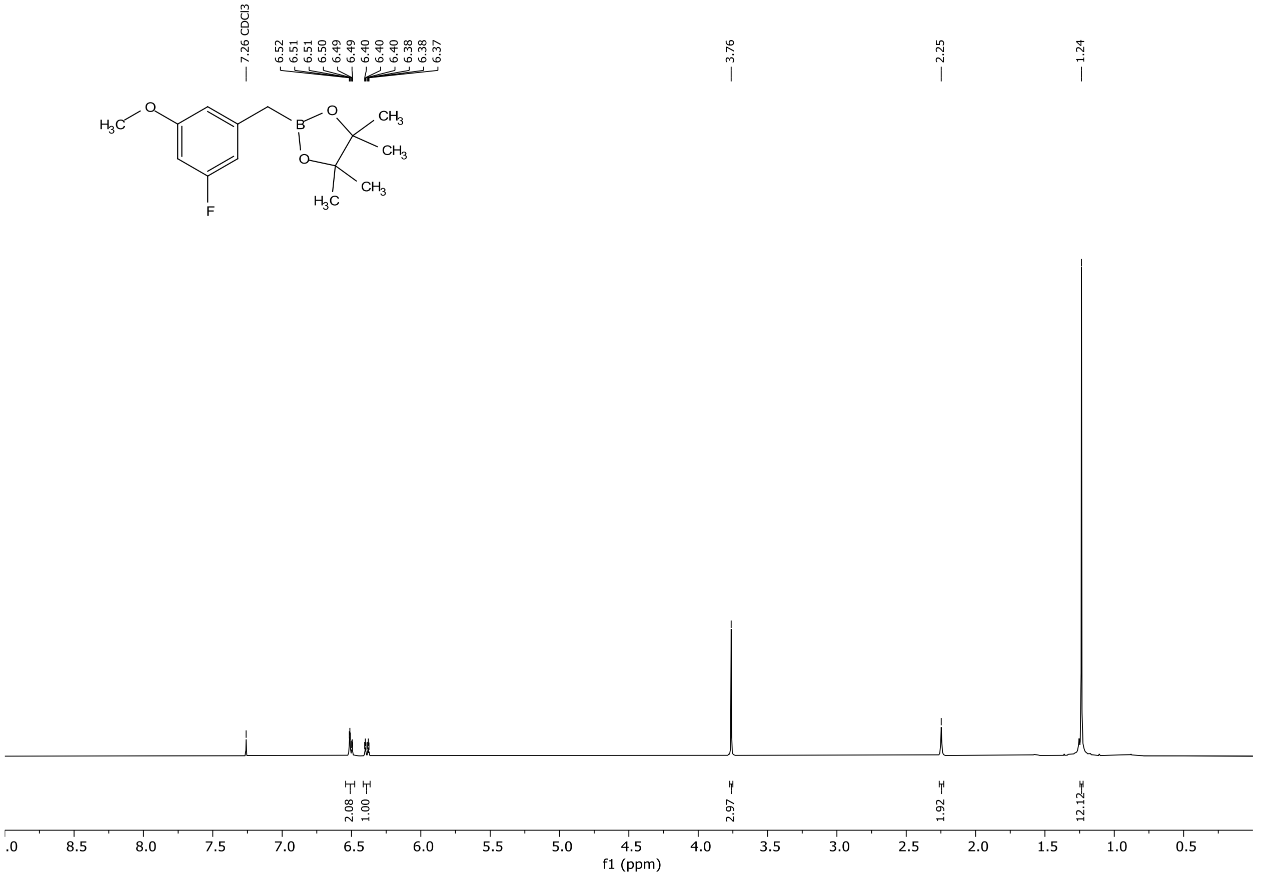

^{13}C NMR (126 MHz, $CDCl_3$)

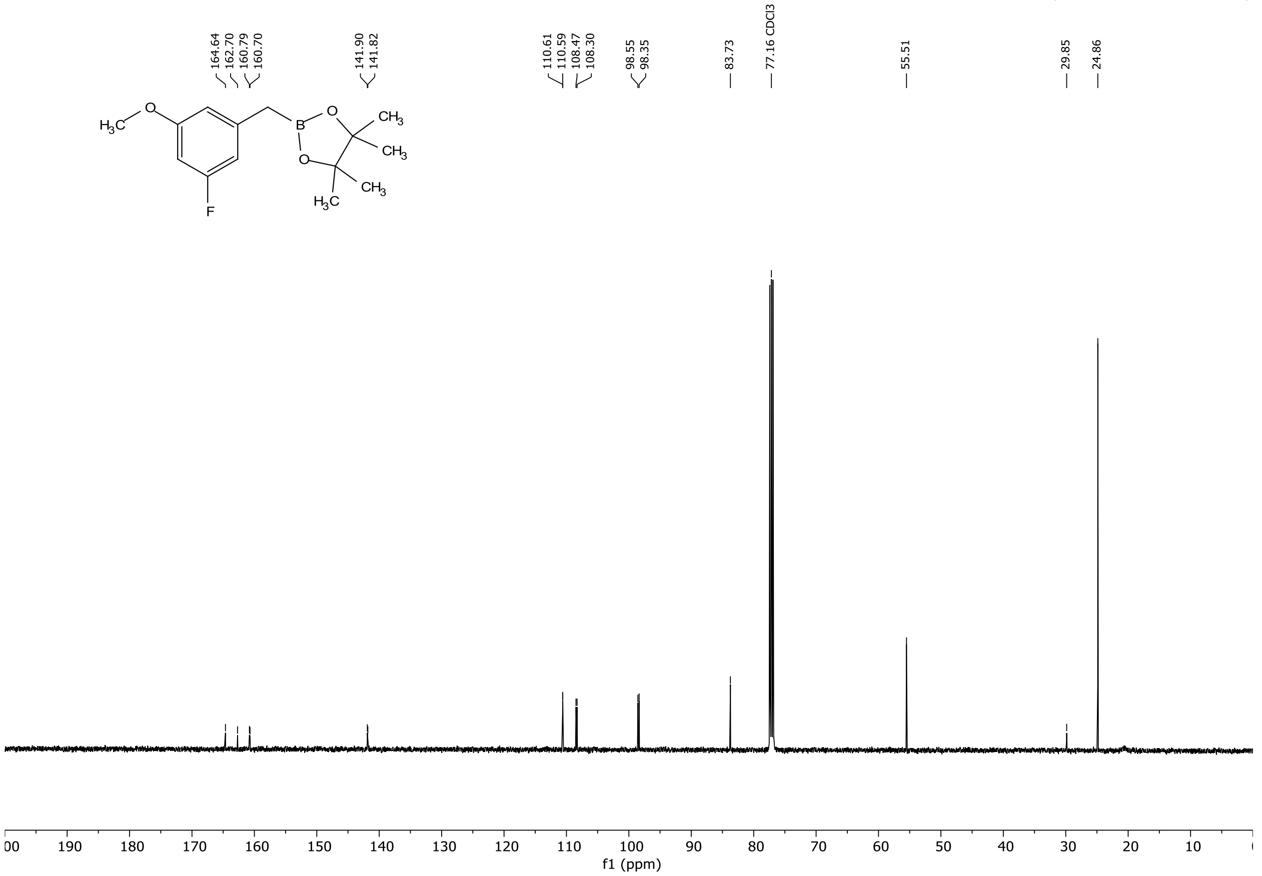

^{11}B NMR (160 MHz, $CDCl_3$)

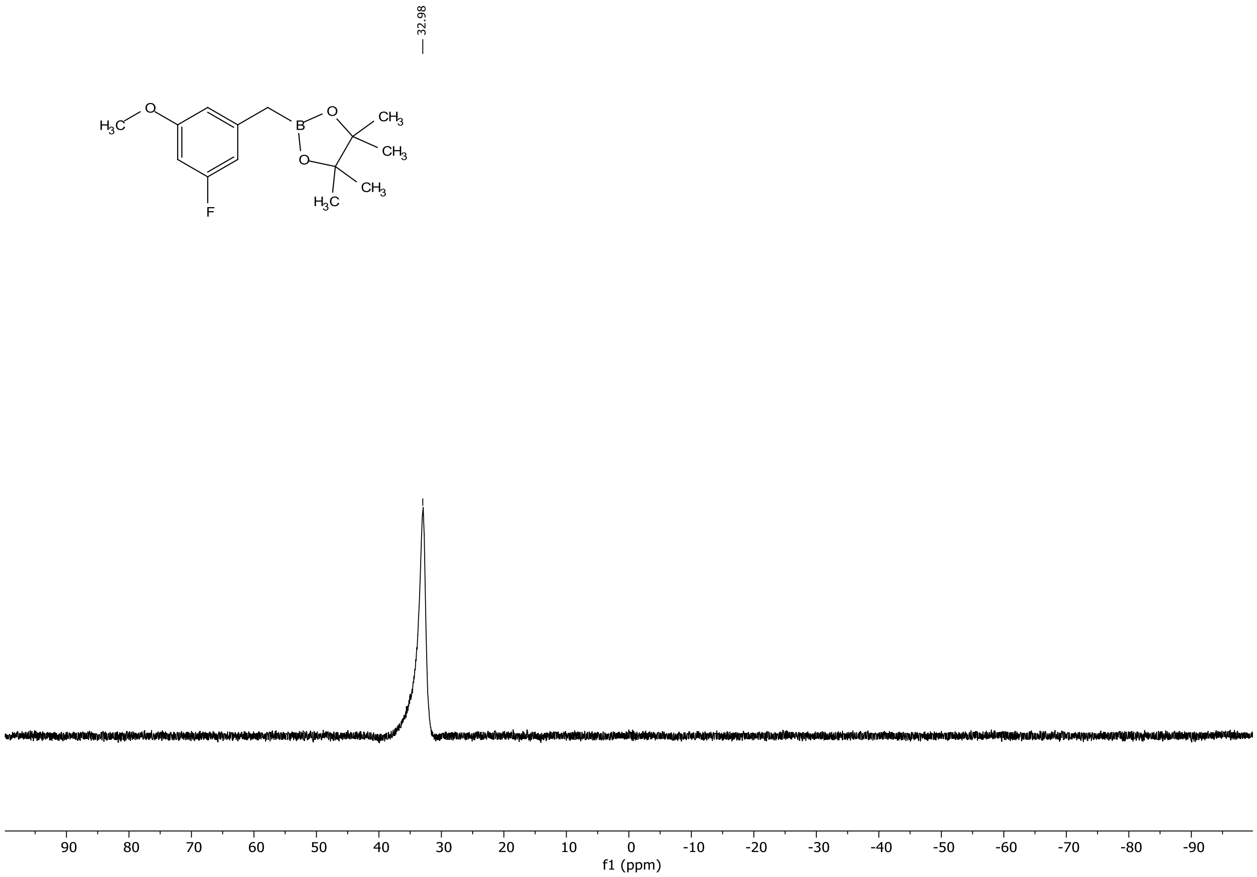

^{19}F NMR (377 MHz, $CDCl_3$)

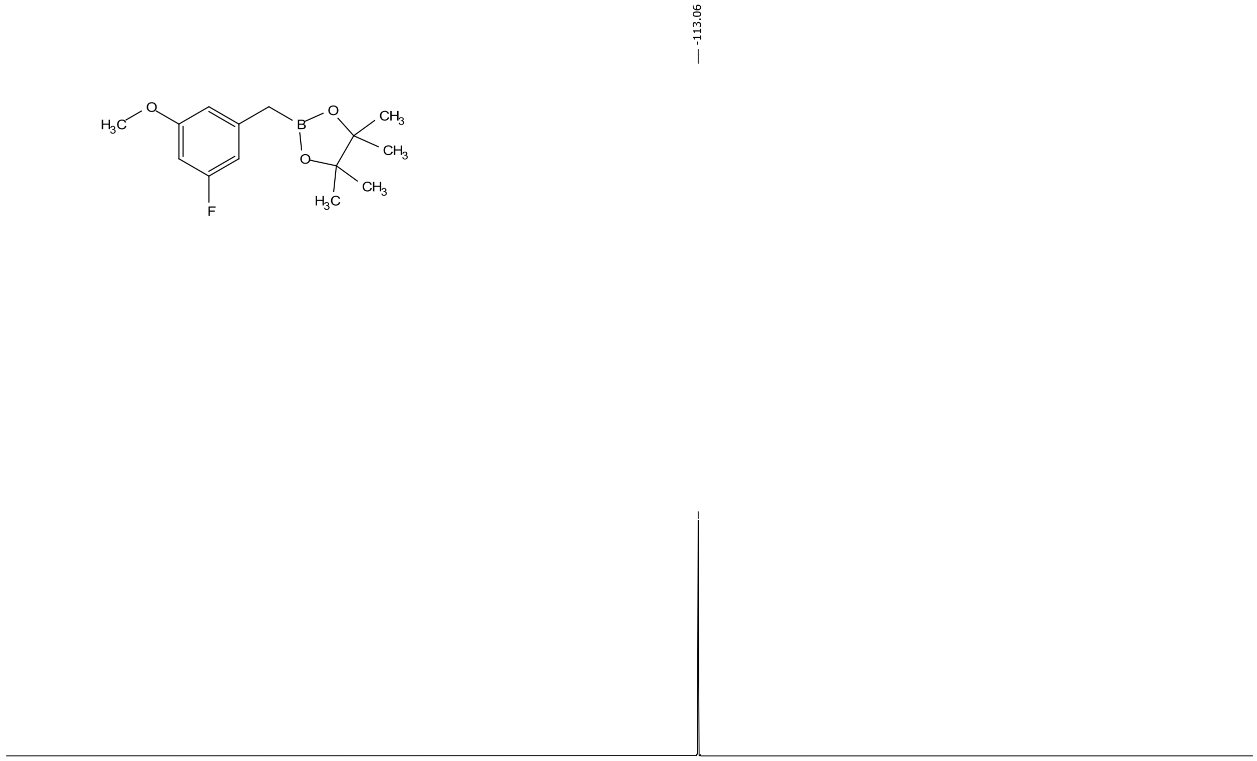

S10.66 2x

^{1}H NMR (400 MHz, $CDCl_3$)

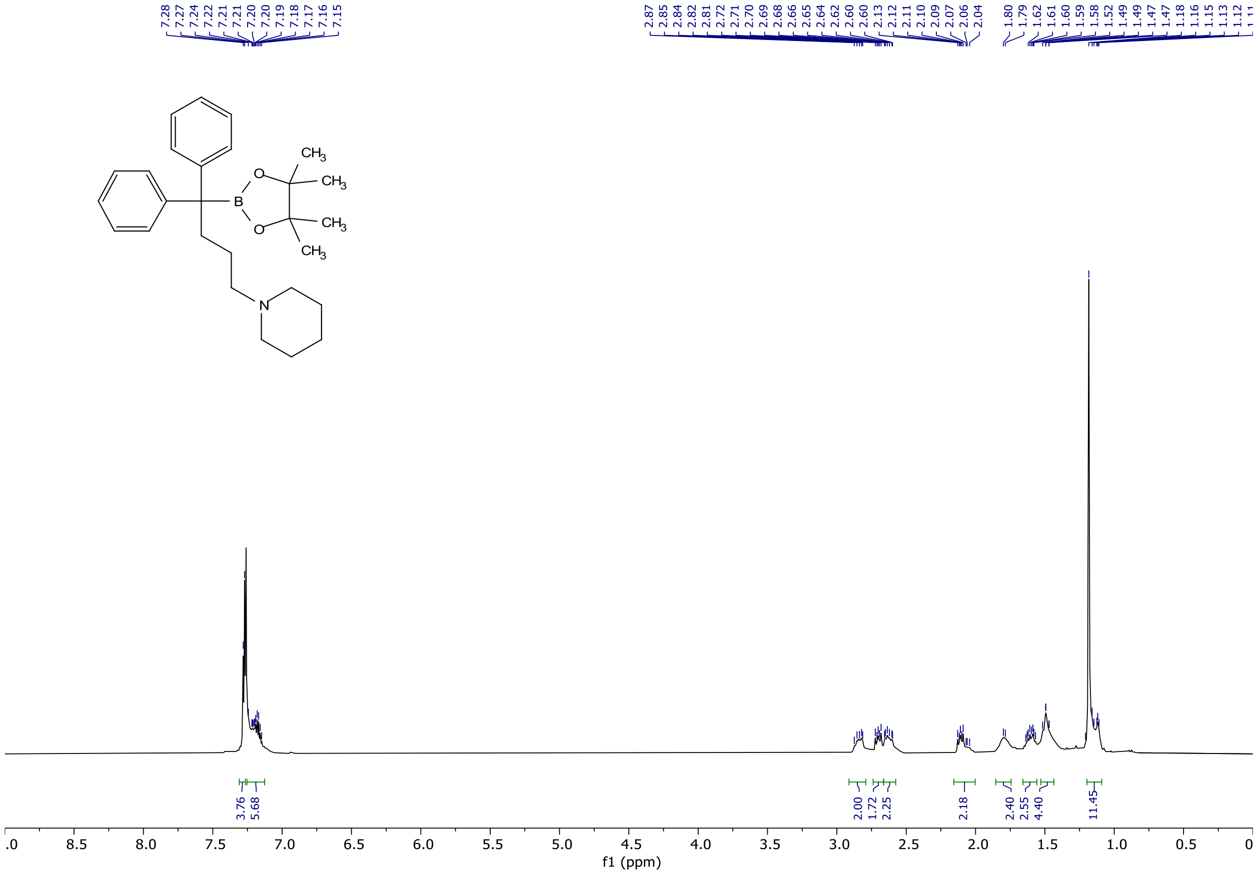

^{13}C NMR (101 MHz, $CDCl_3$)

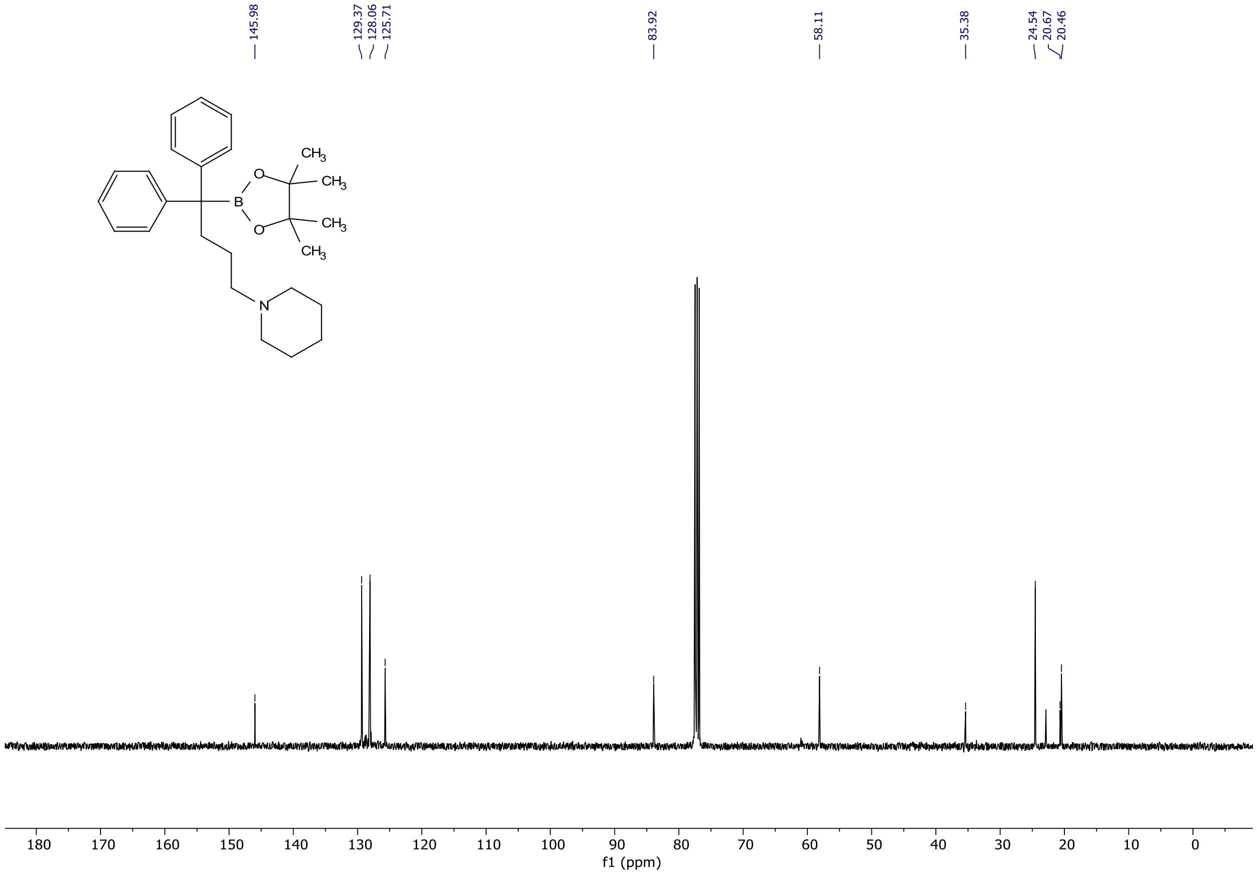

^{11}B NMR (128 MHz, $CDCl_3$)

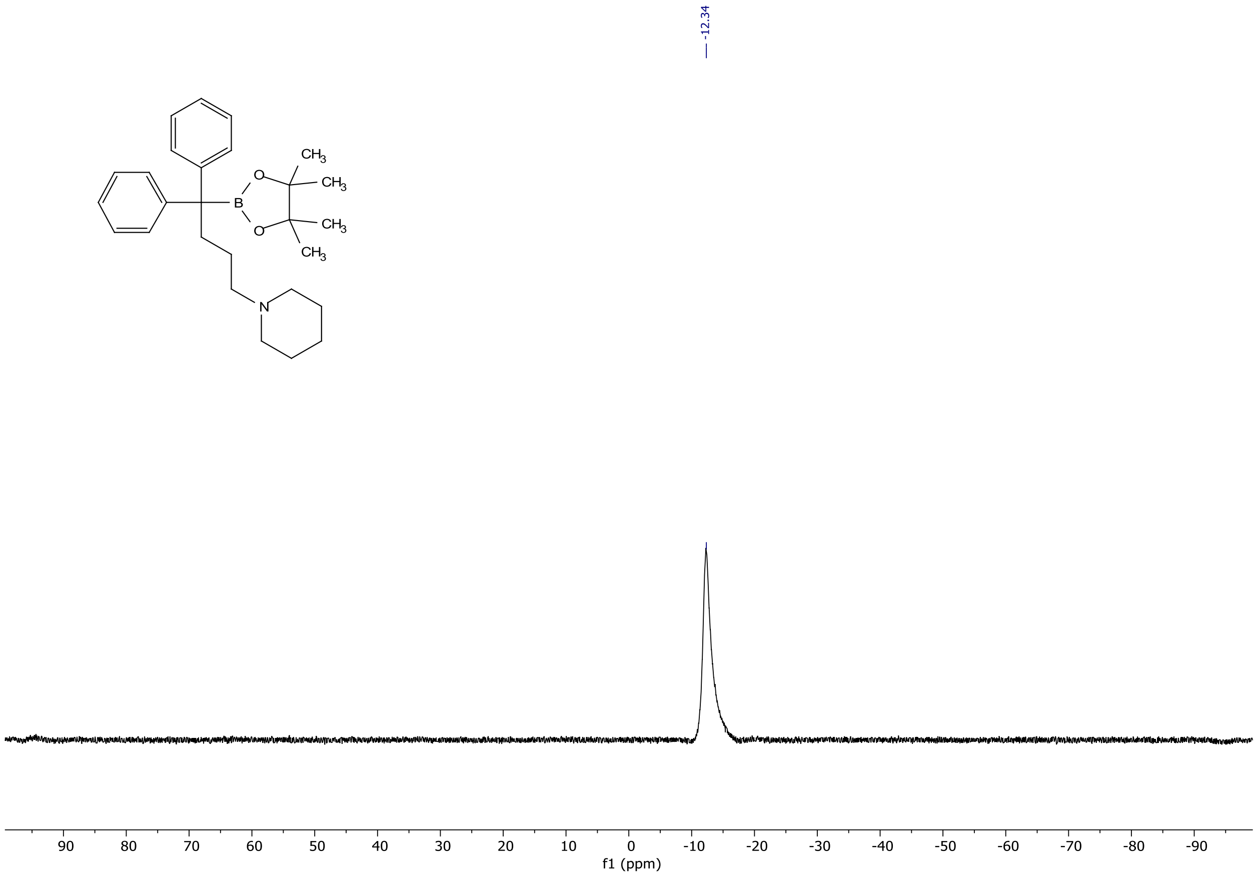

S10.67 2y

^{1}H NMR (400 MHz, $CDCl_3$)

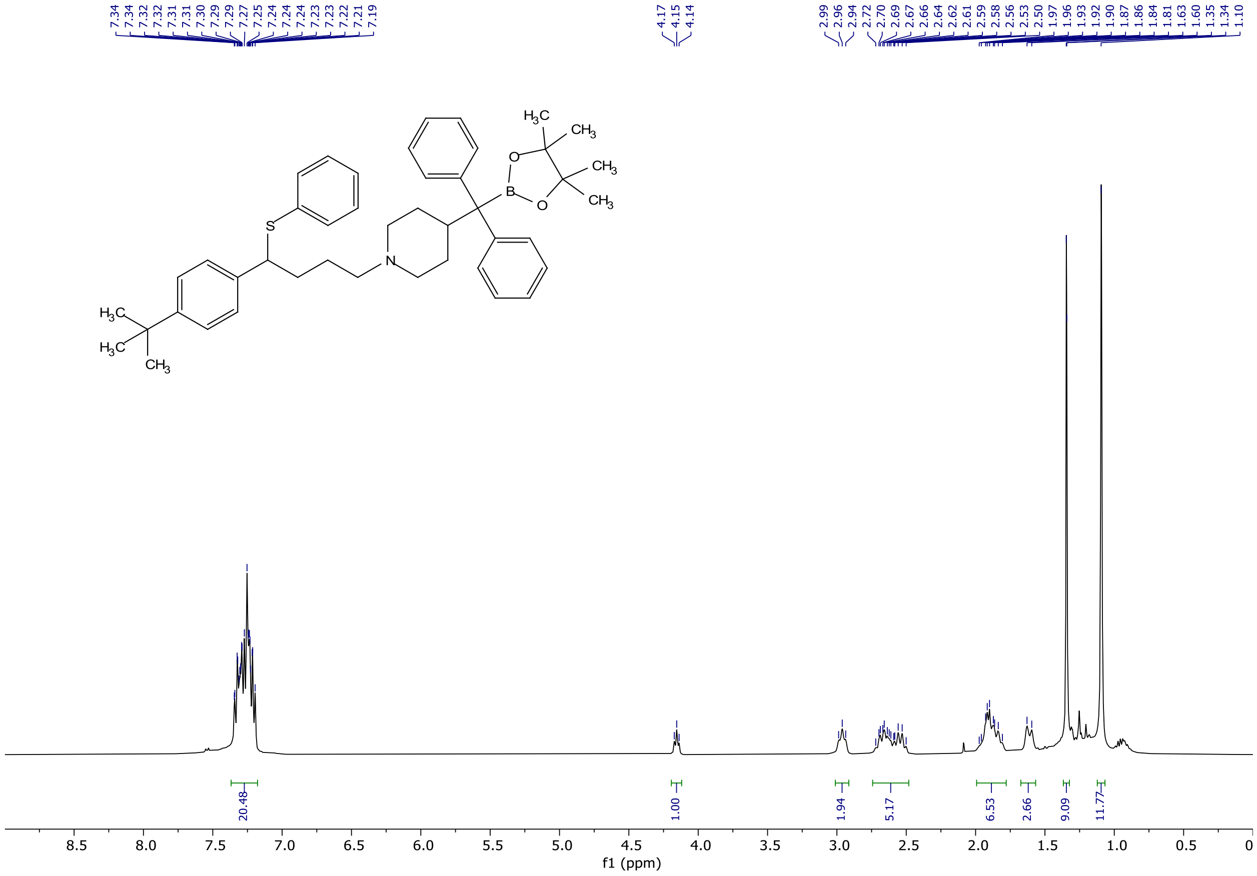

^{13}C NMR (101 MHz, $CDCl_3$)

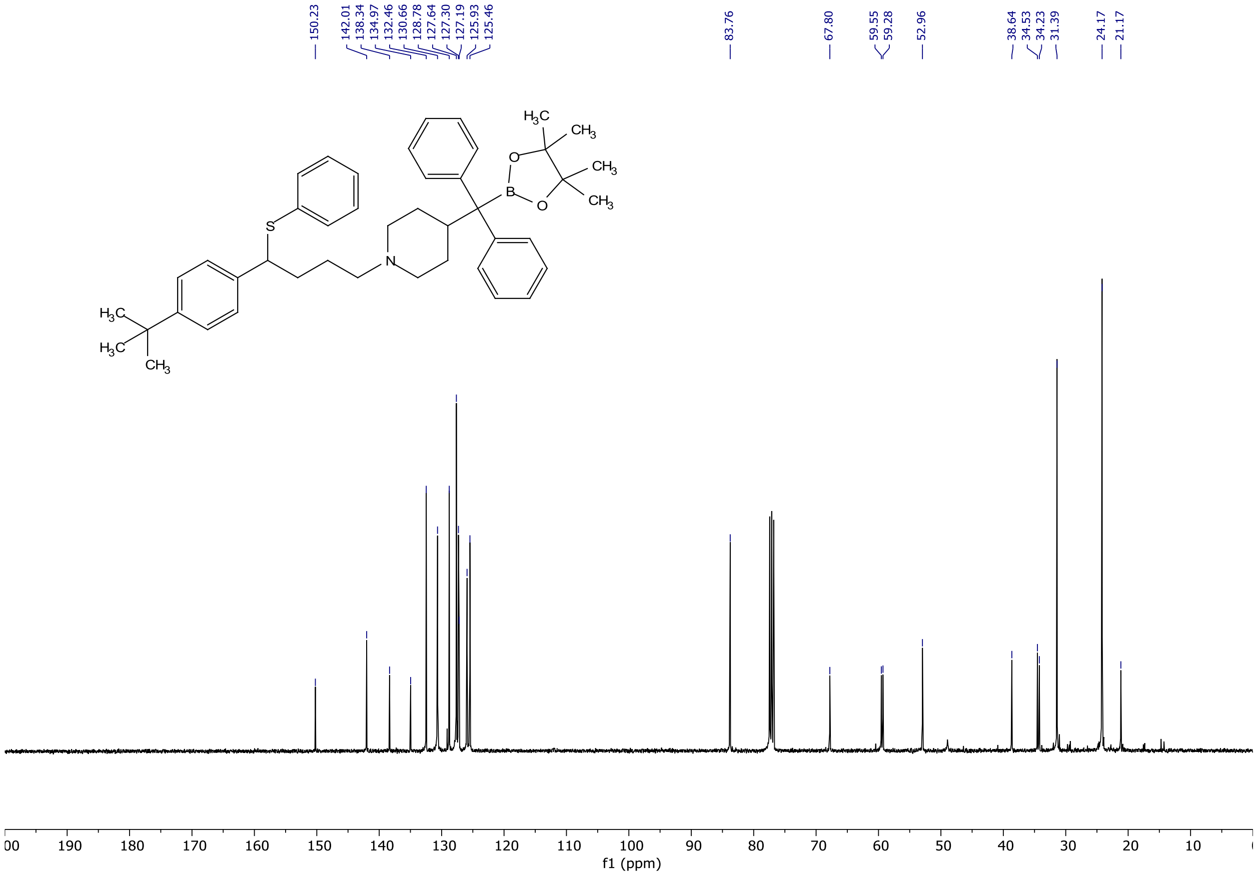

^{11}B NMR (128 MHz, $CDCl_3$)

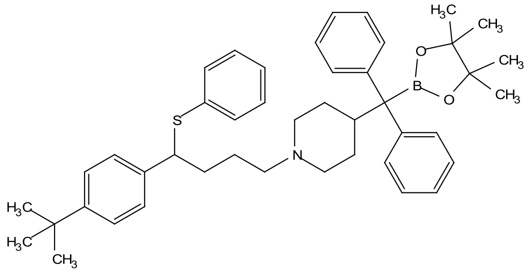

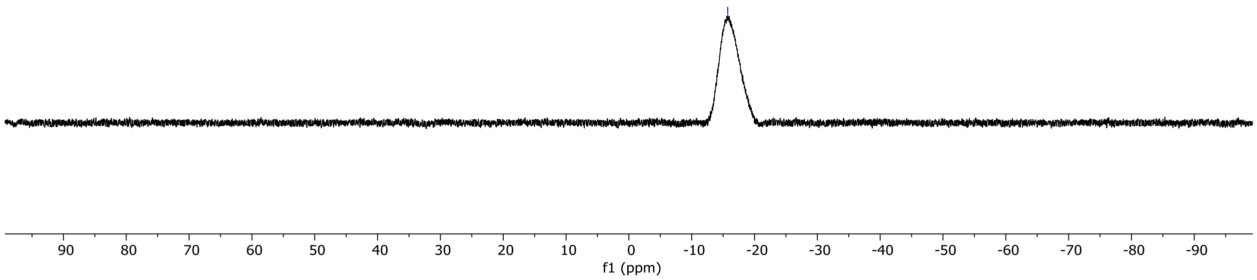

S10.68 2z

^{1}H NMR (400 MHz, $CDCl_3$)

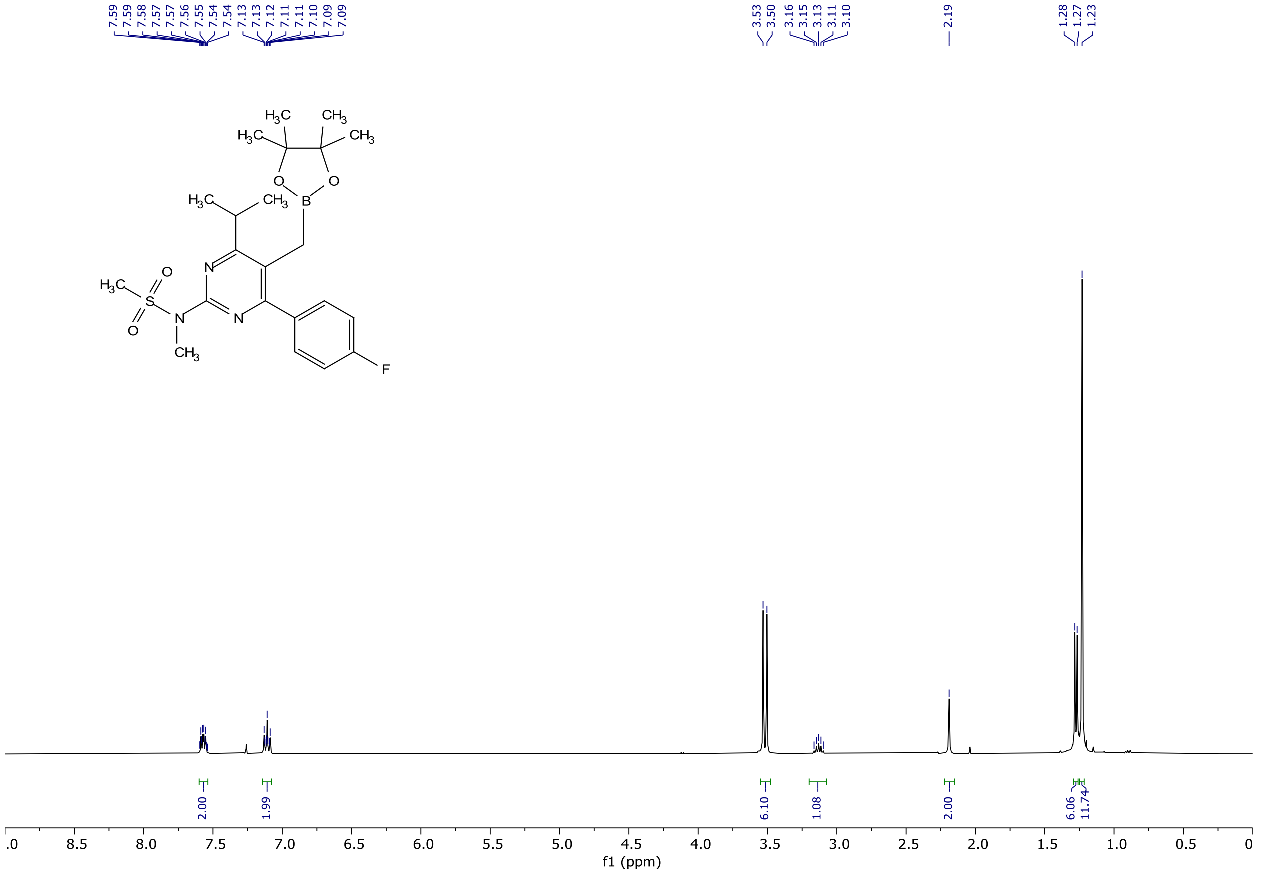

^{13}C NMR (101 MHz, $CDCl_3$)

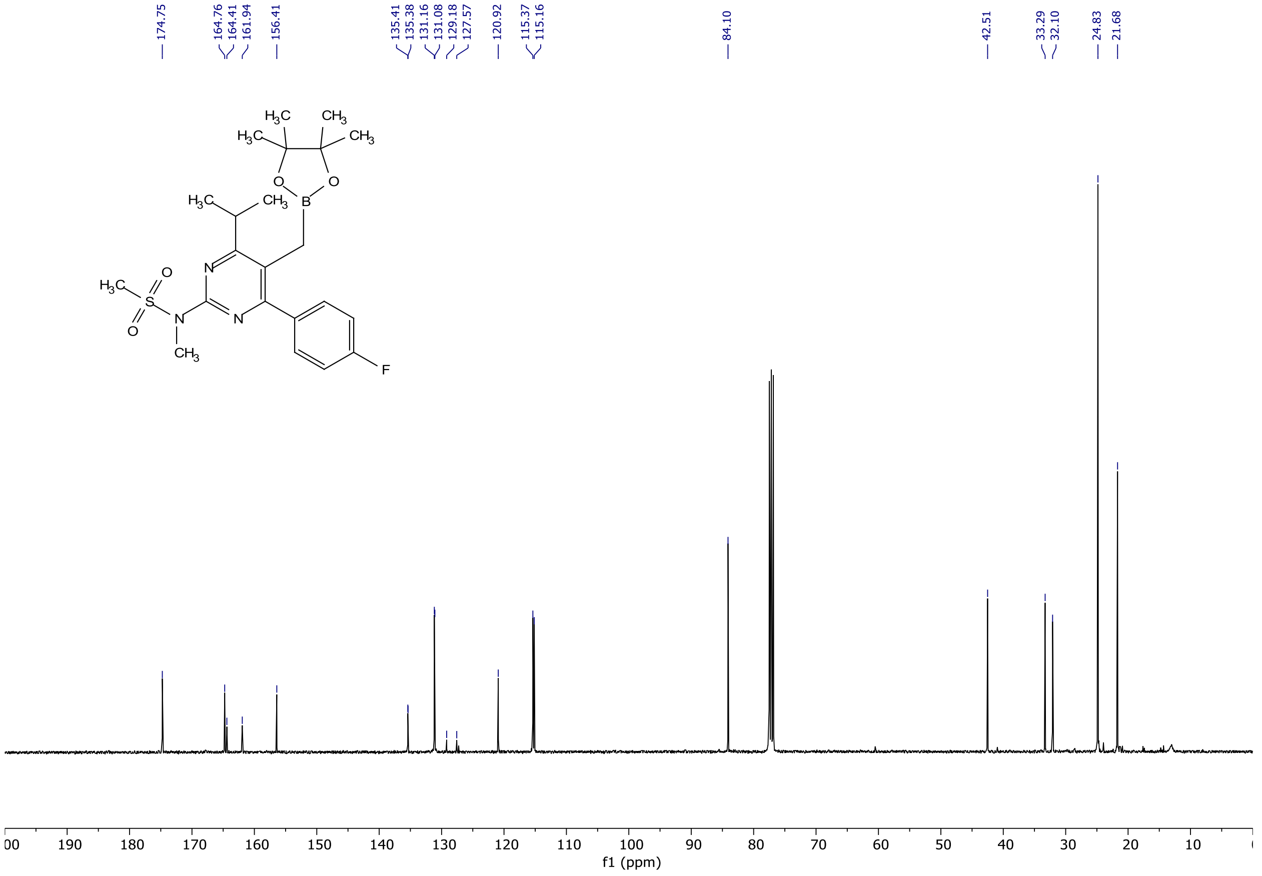

^{11}B NMR (128 MHz, $CDCl_3$)

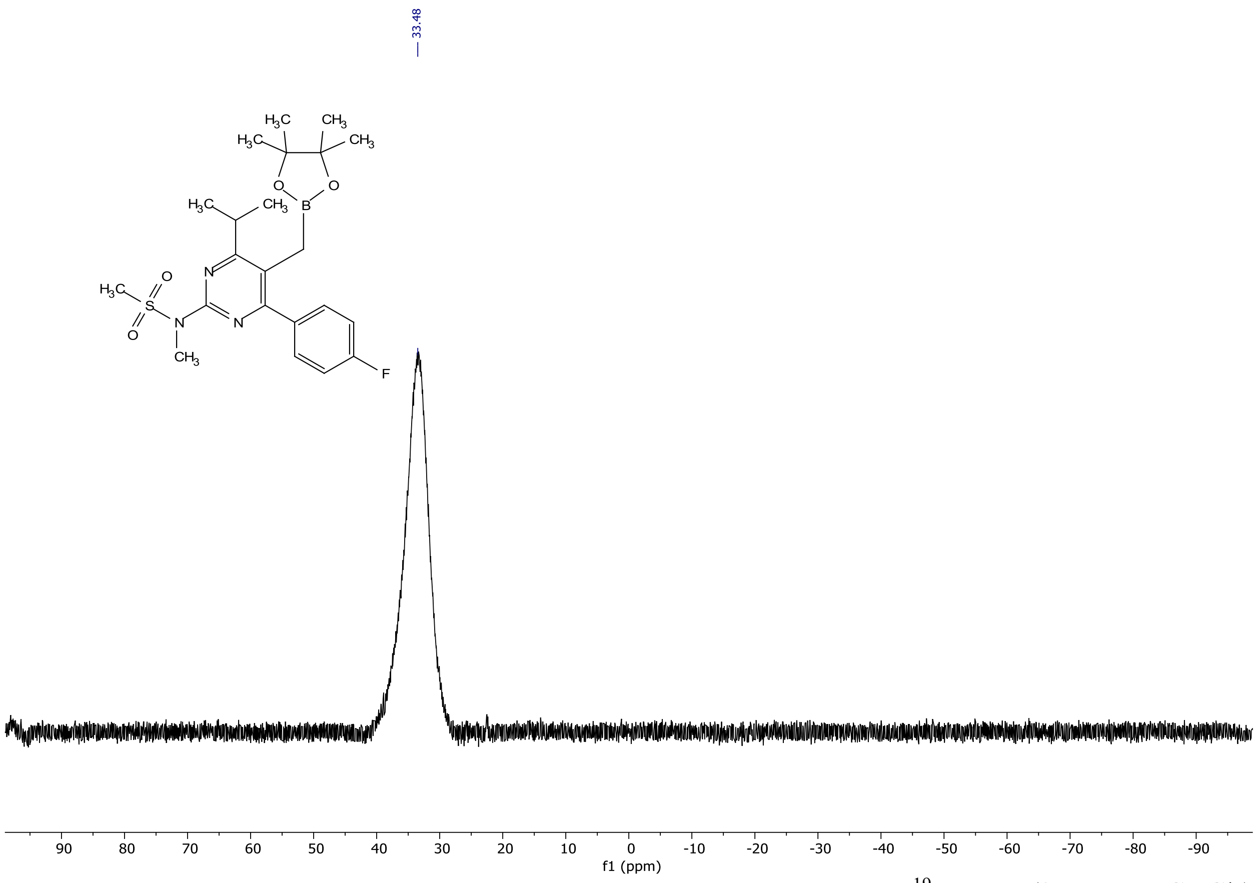

^{19}F NMR (377 MHz, $CDCl_3$)

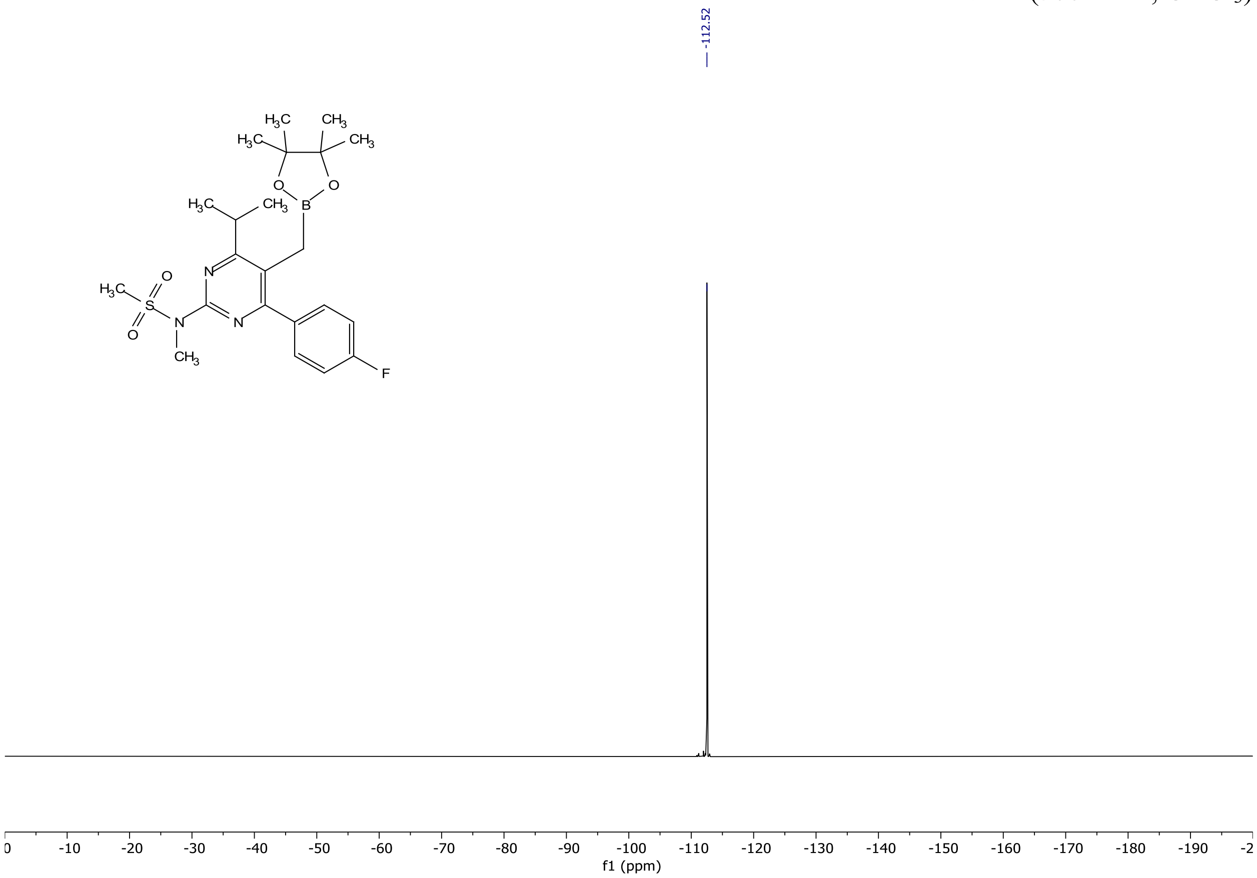

S10.69 2aa

^{1}H NMR (400 MHz, $CDCl_3$)

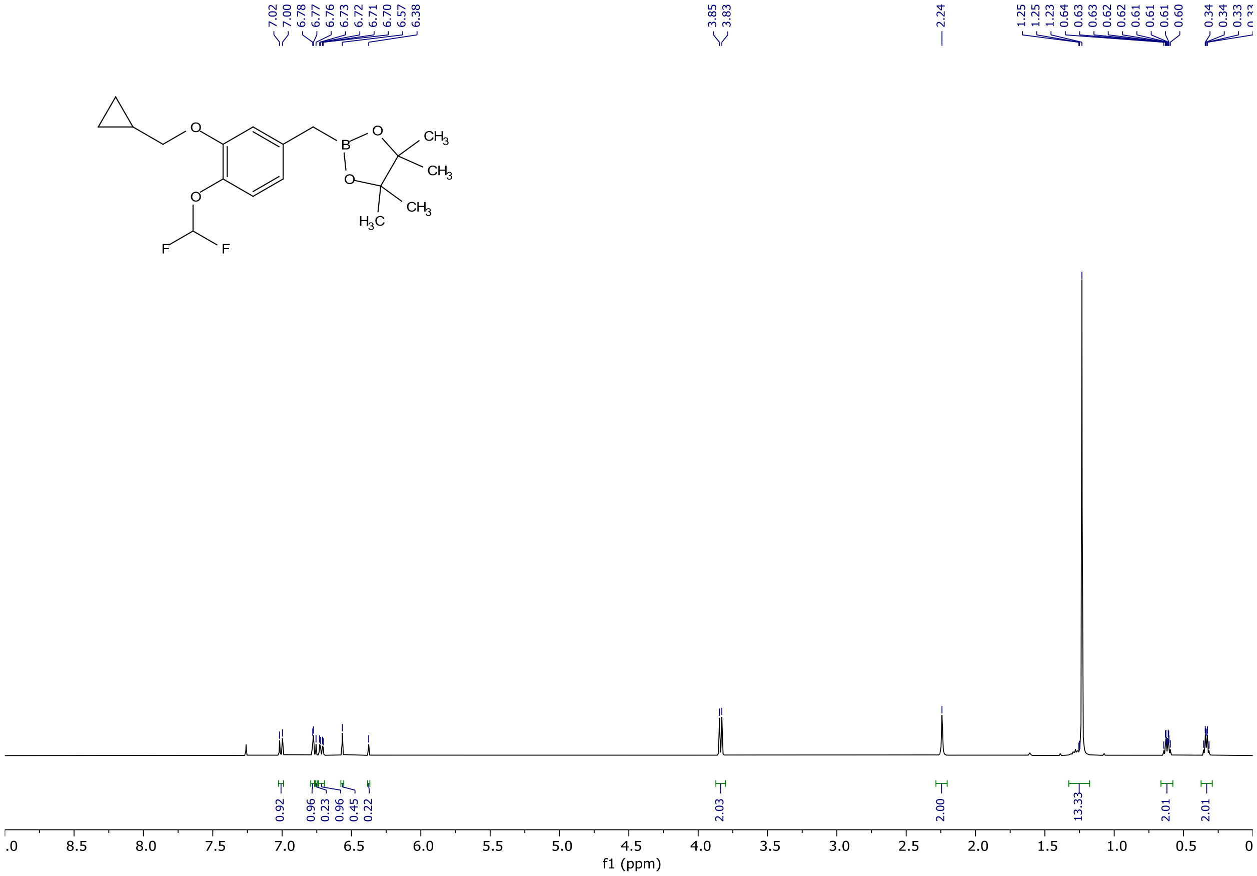

^{13}C NMR (101 MHz, $CDCl_3$)

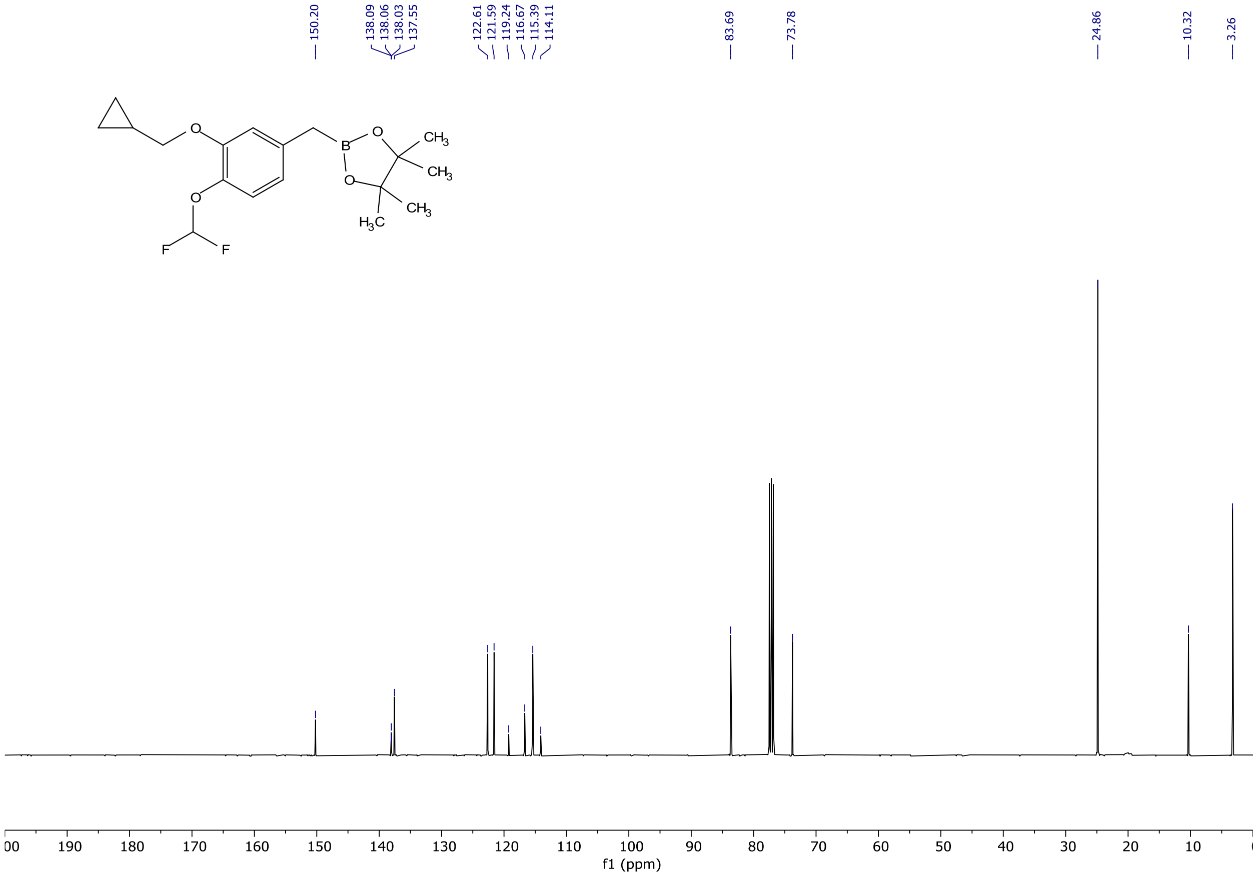

^{11}B NMR (128 MHz, $CDCl_3$)

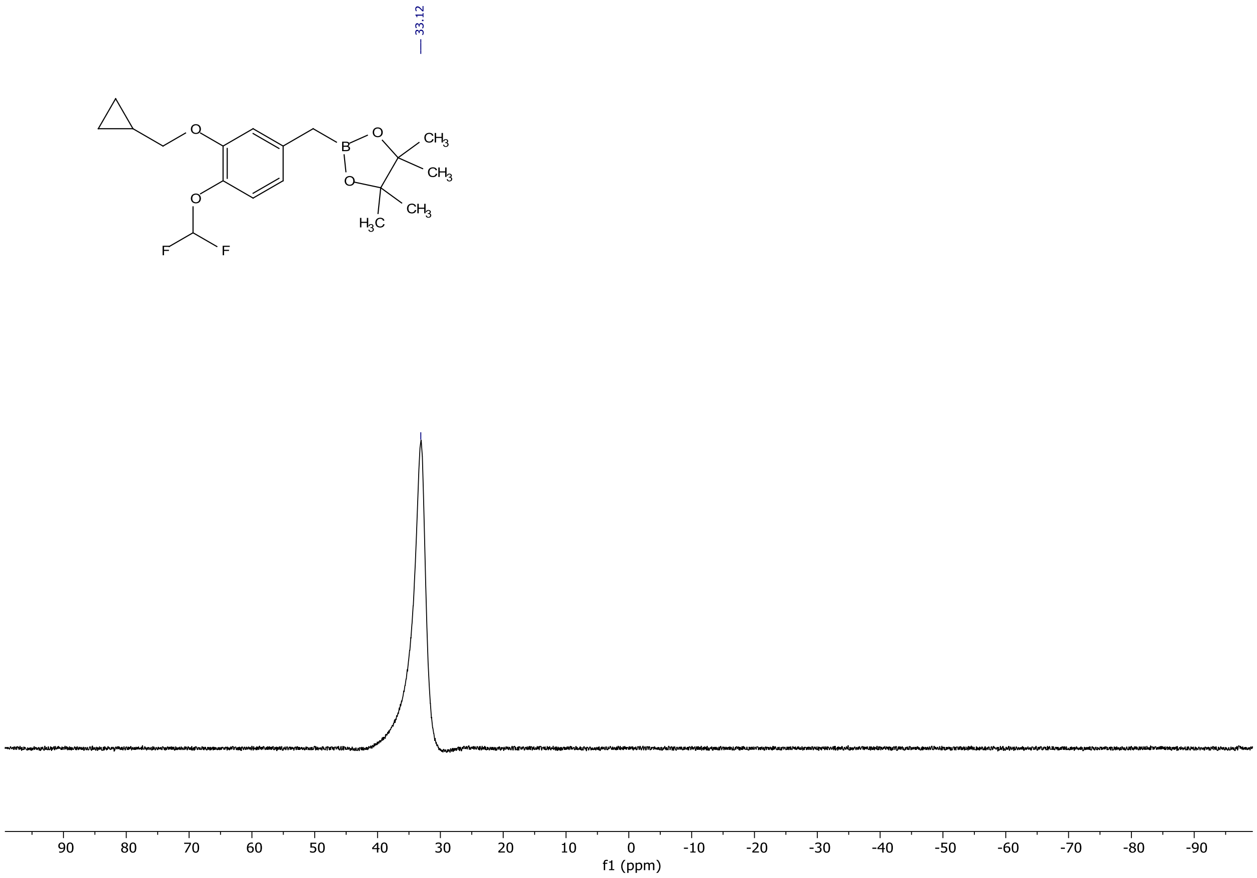

^{19}F NMR (377 MHz, $CDCl_3$)

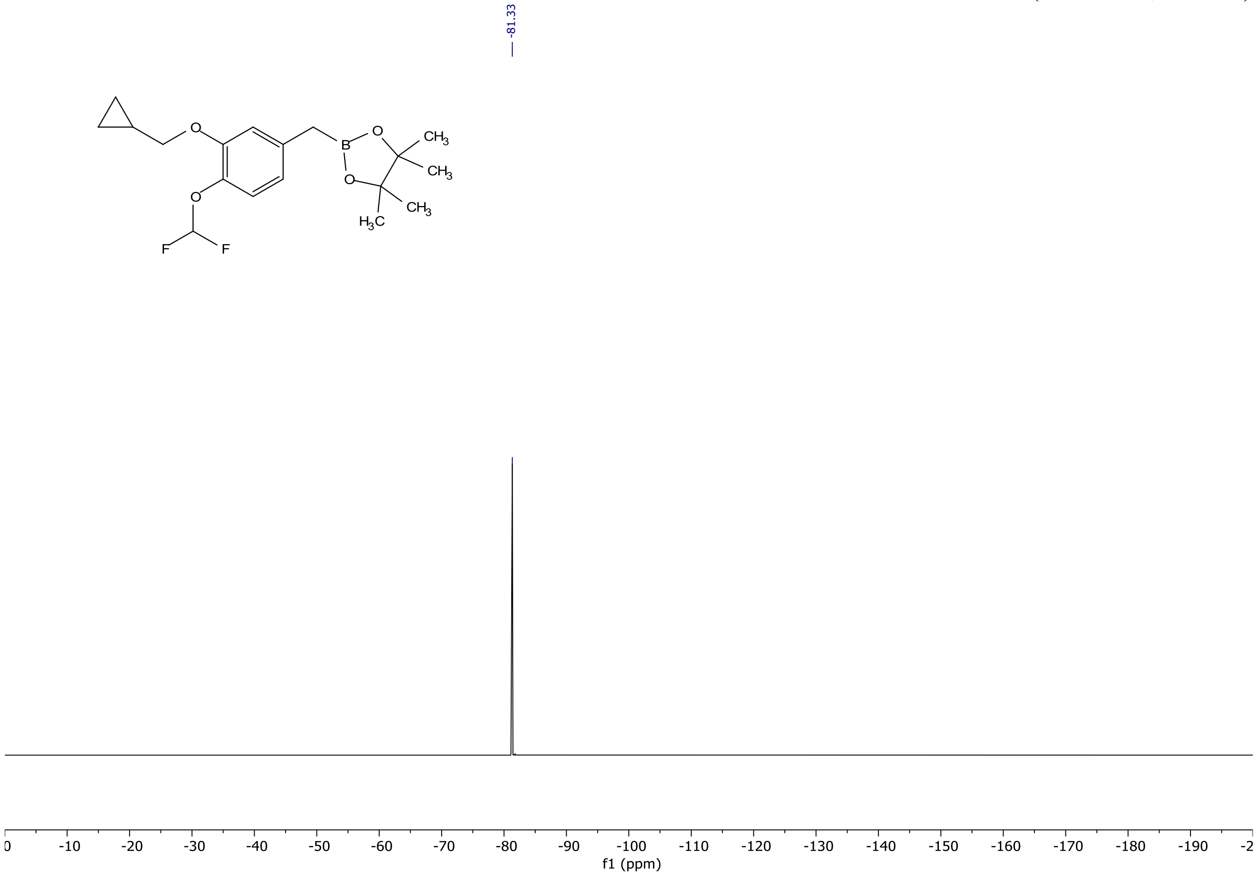

S10.70 2ab

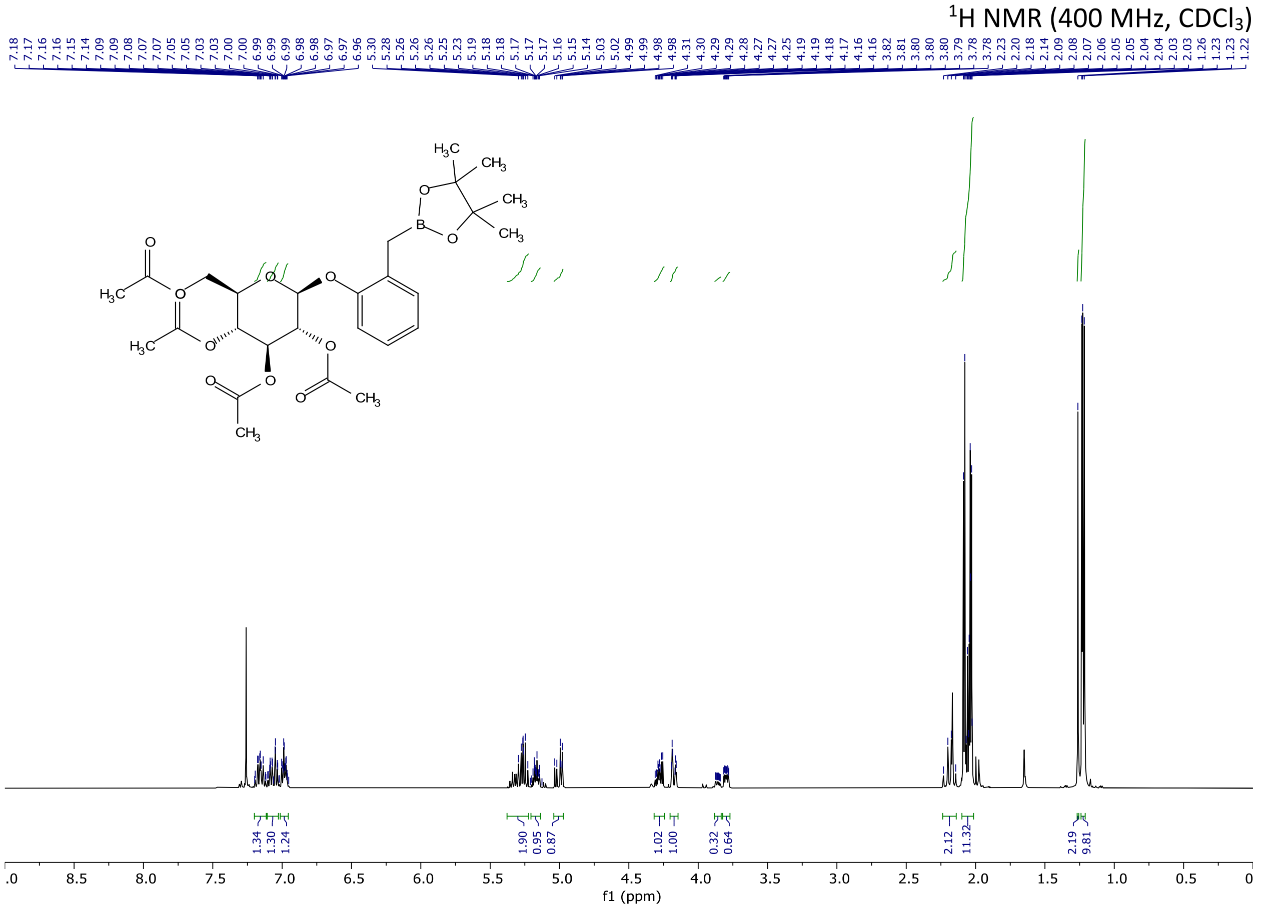

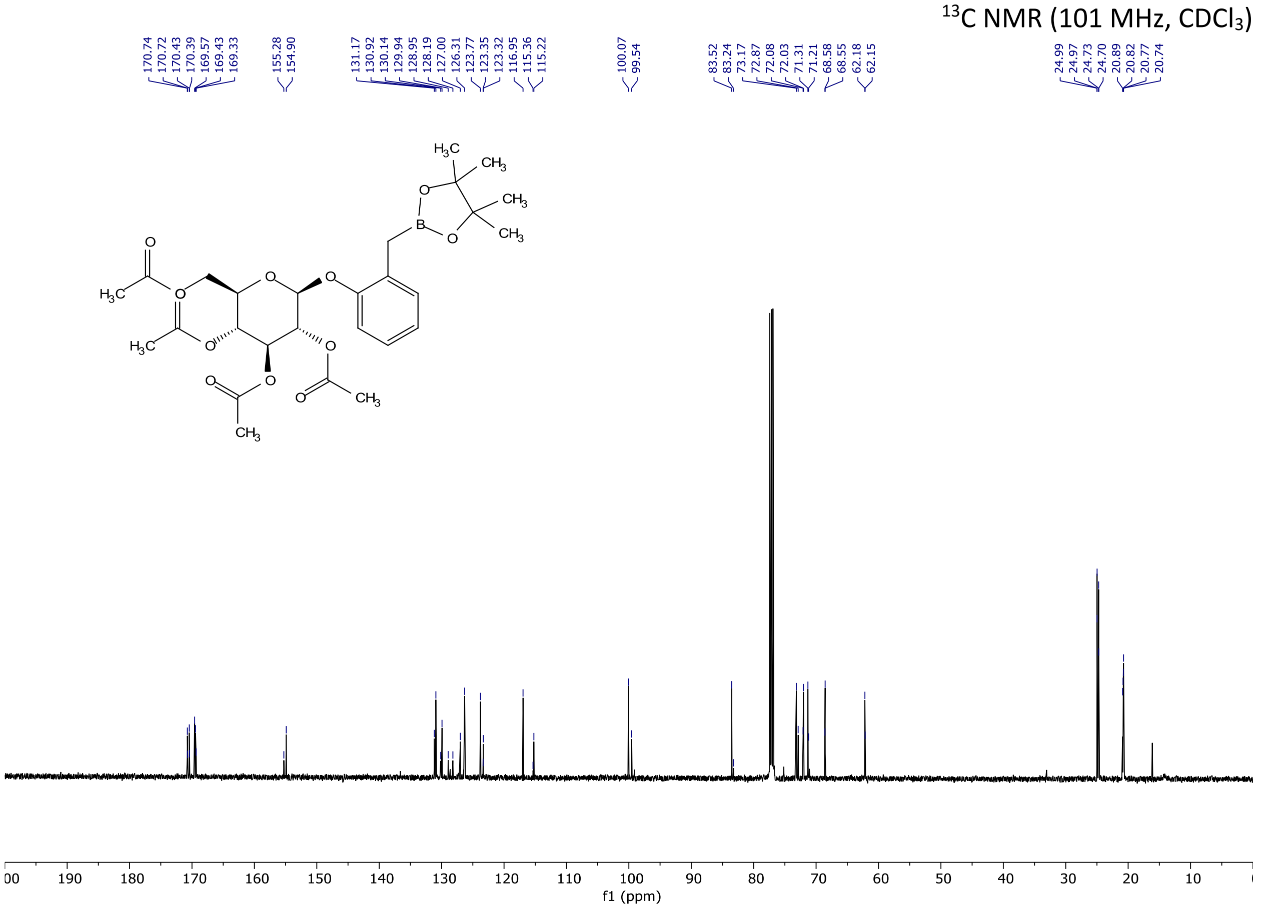

^{11}B NMR (128 MHz, $CDCl_3$)

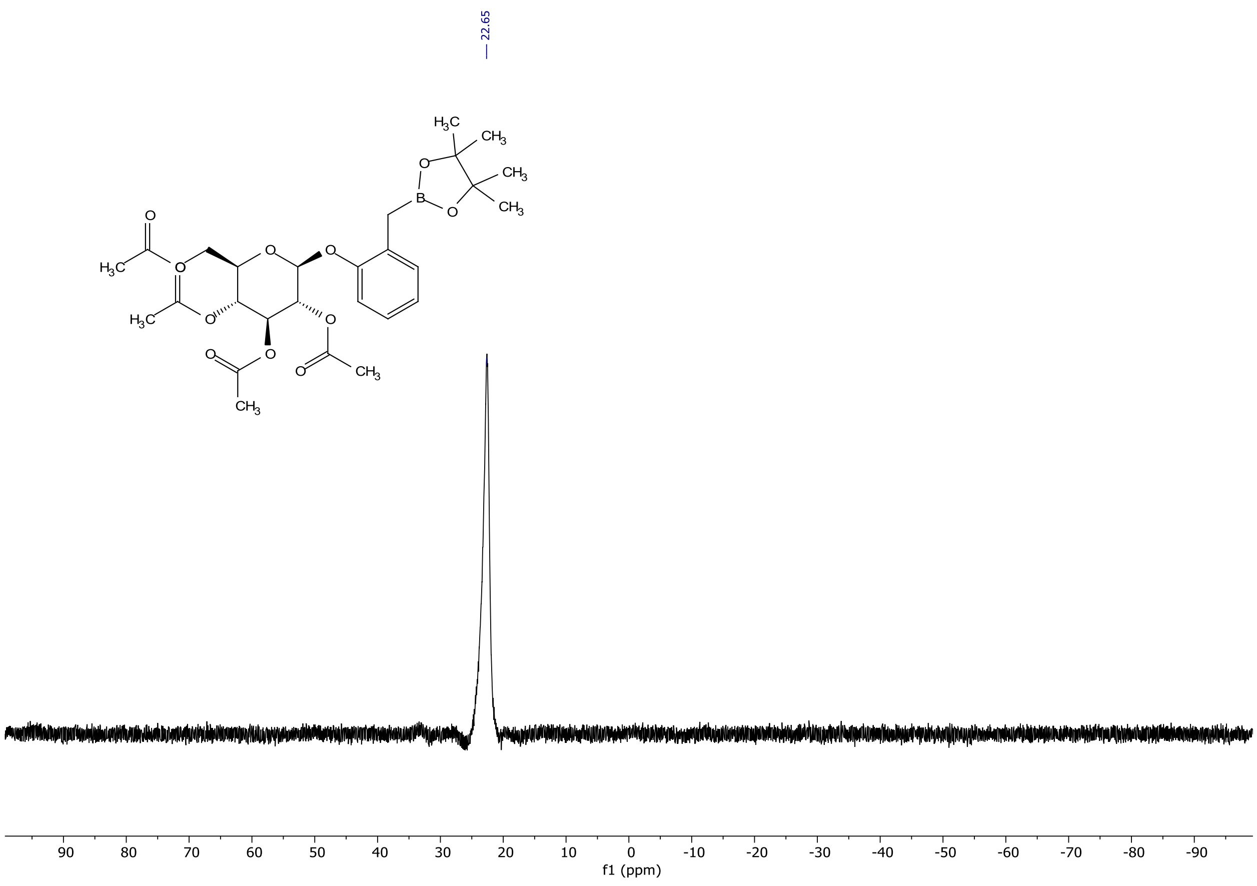

S10.71 2ac

^{1}H NMR (400 MHz, $CDCl_3$)

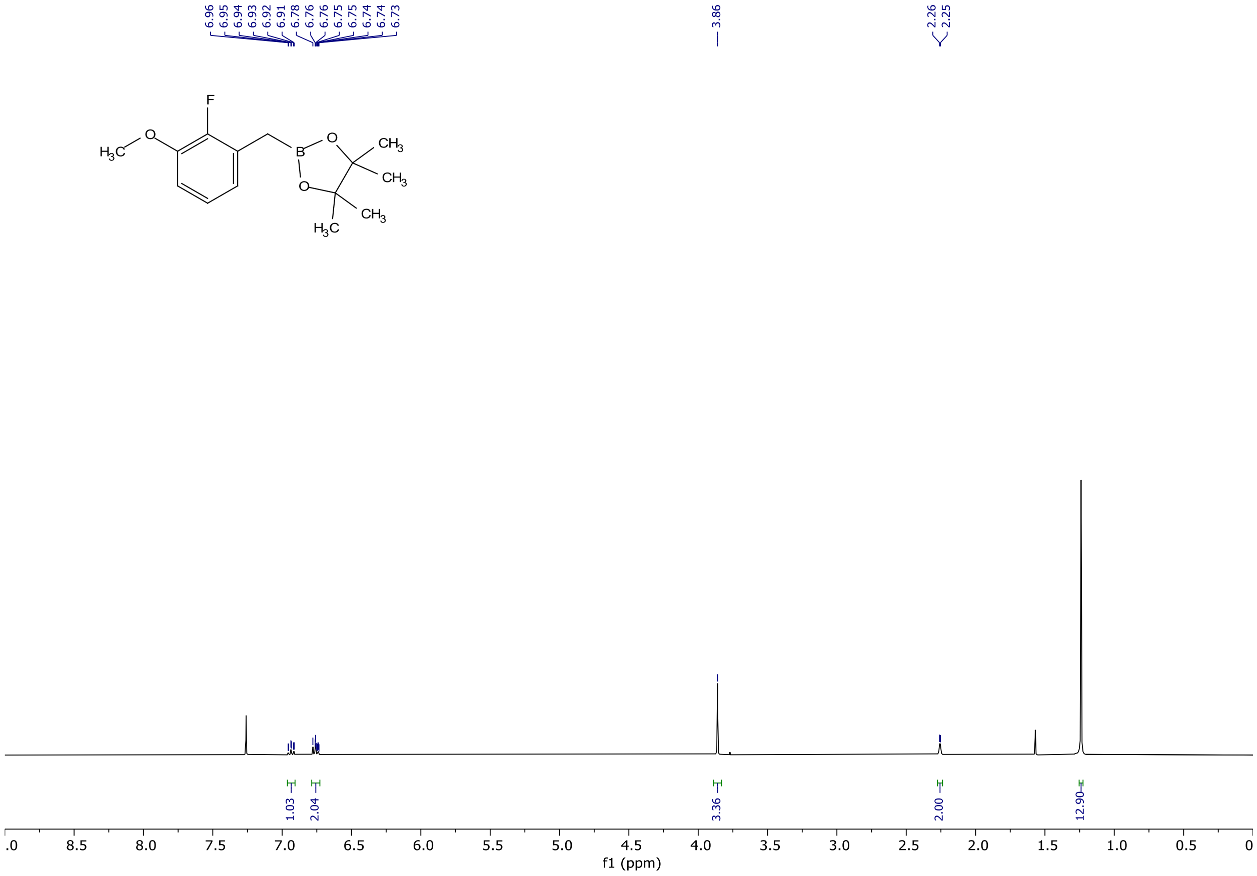

^{13}C NMR (101 MHz, $CDCl_3$)

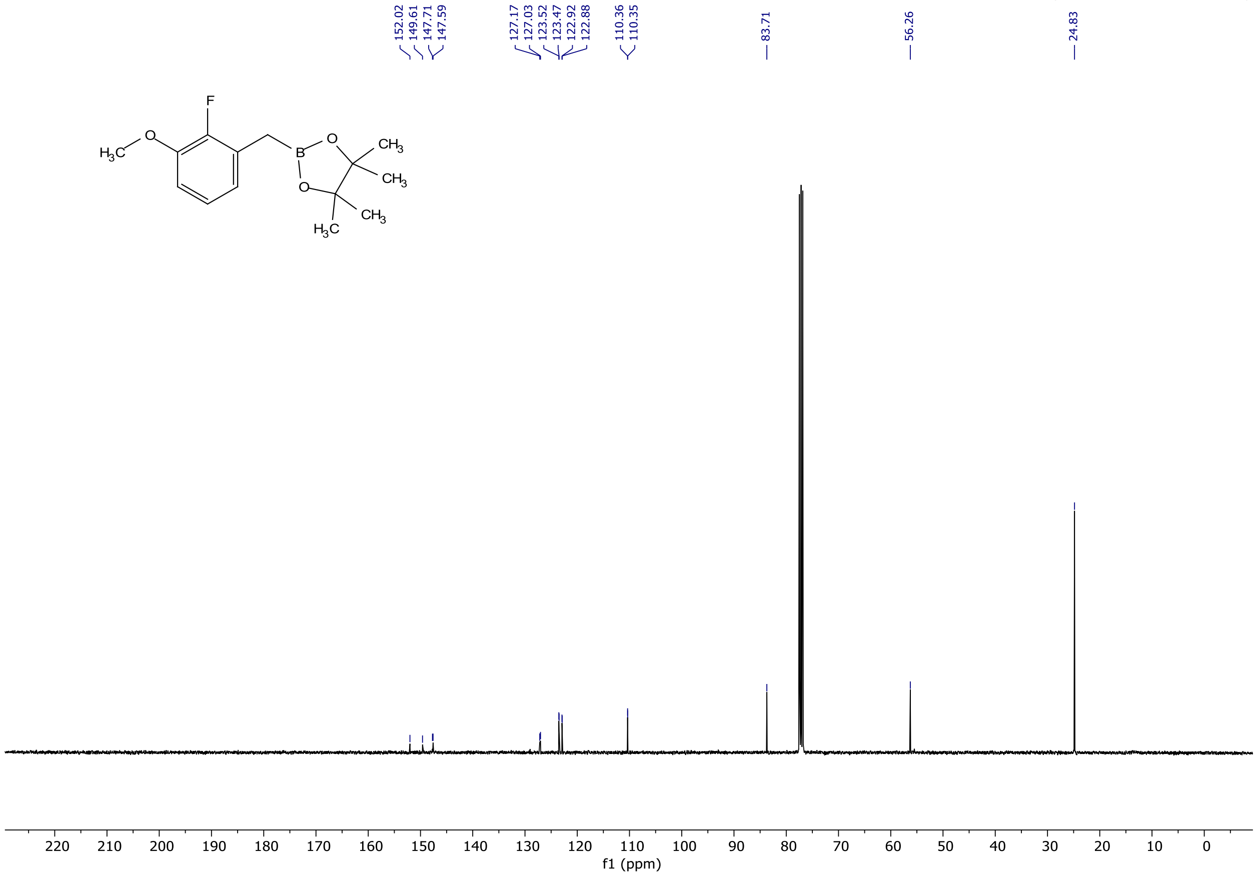

^{11}B NMR (128 MHz, $CDCl_3$)

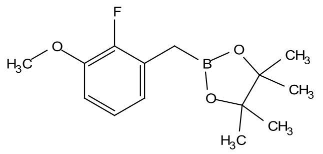

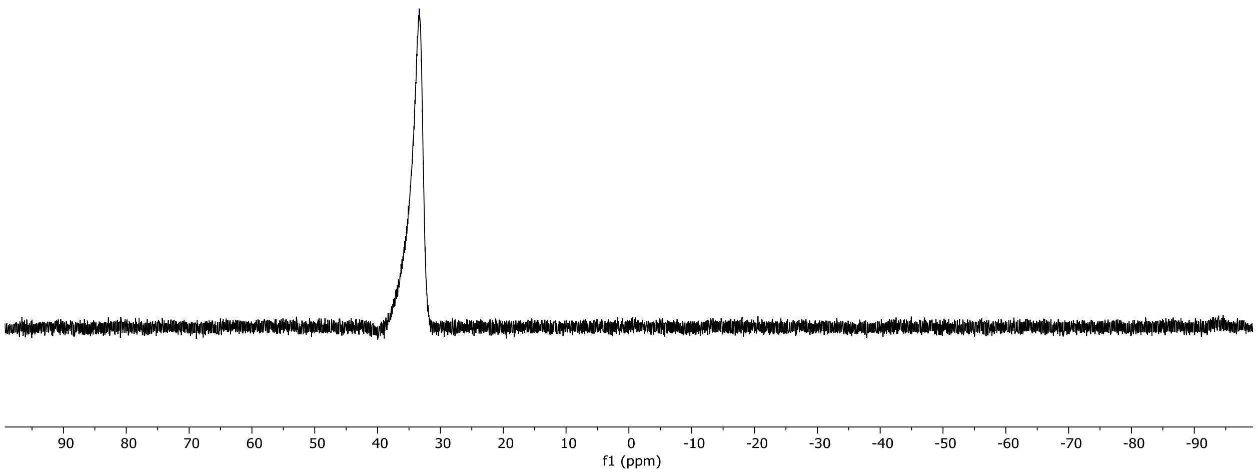

S10.72 2ad

^{1}H NMR (400 MHz, $CDCl_3$)

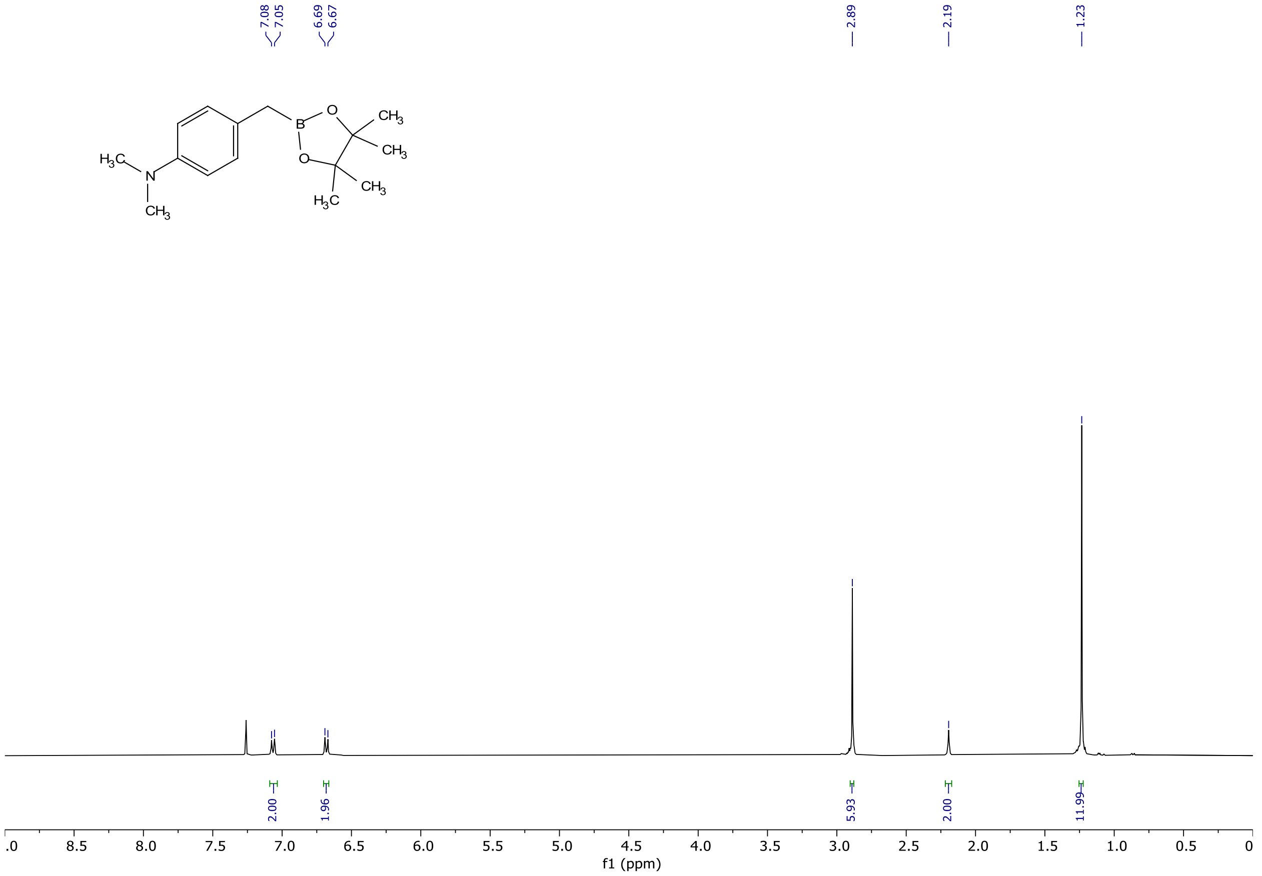

^{13}C NMR (101 MHz, $CDCl_3$)

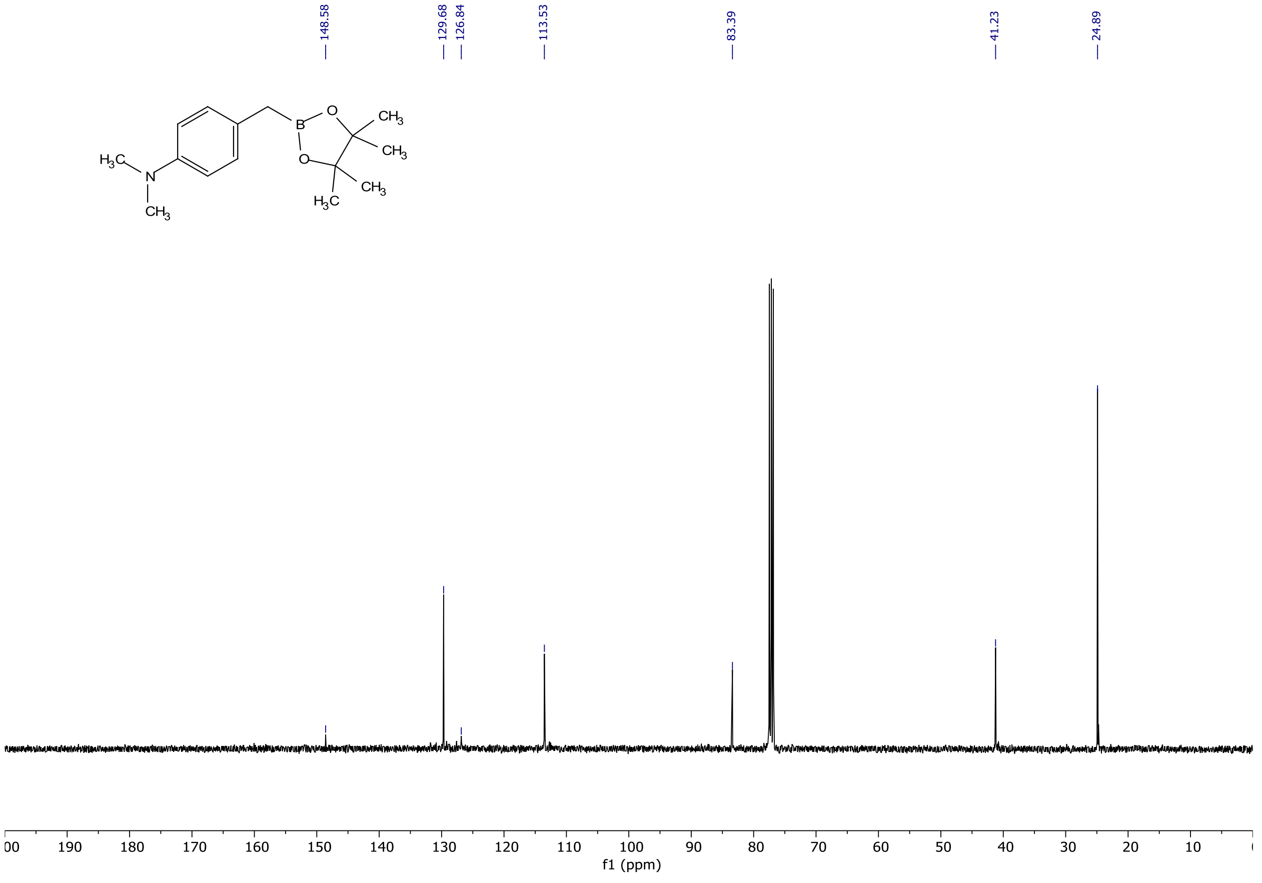

^{11}B NMR (128 MHz, $CDCl_3$)

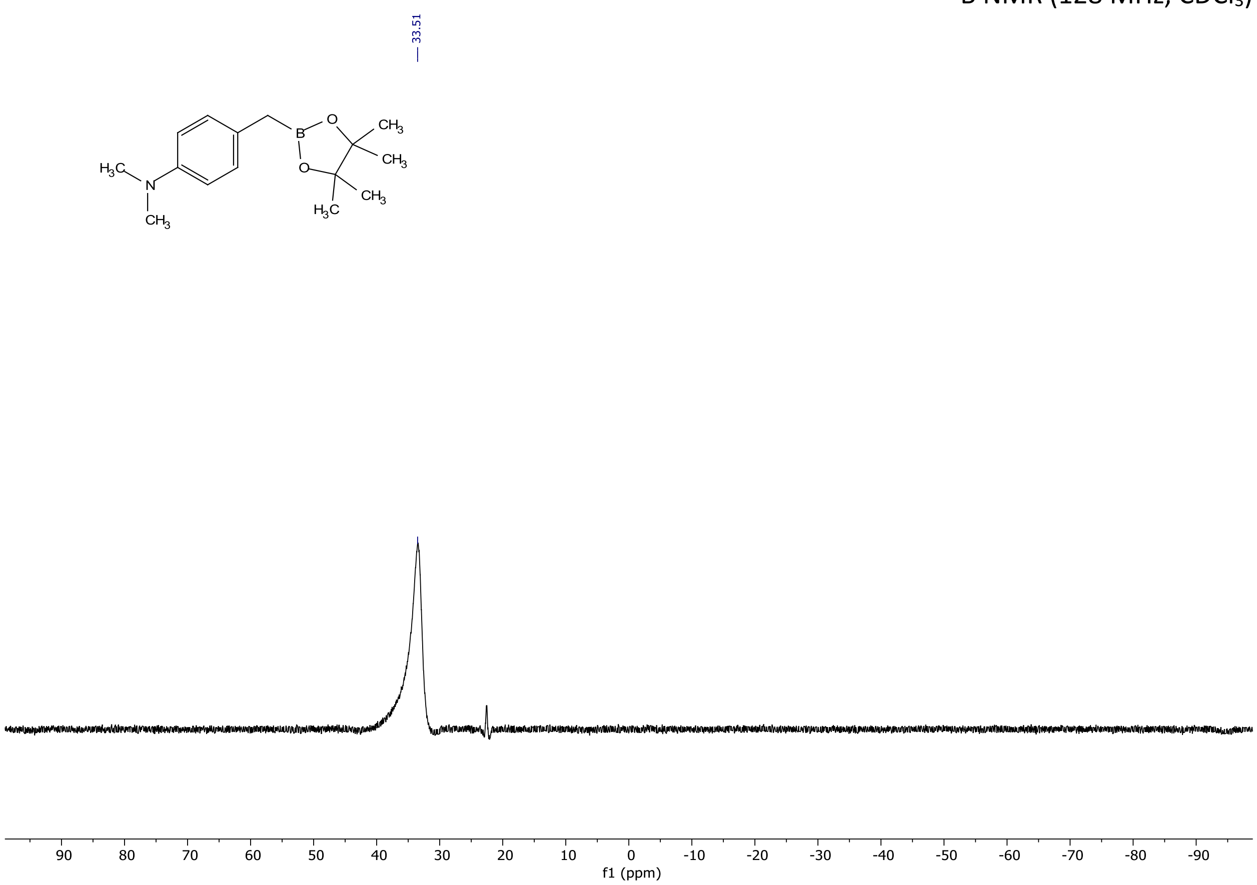

S10.73 2ae

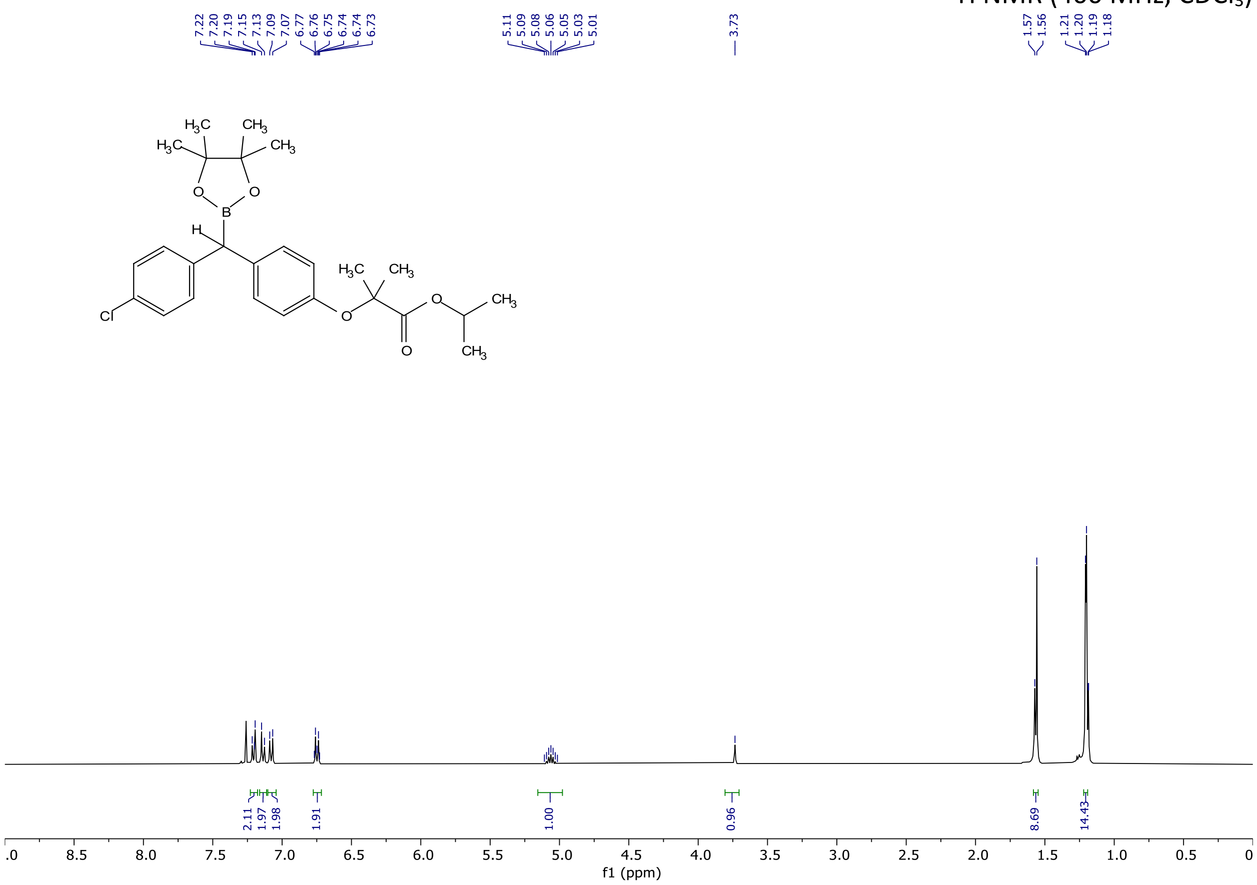

¹H NMR (400 MHz, CDCl3)
7.22
7.20
7.19
7.15
7.13
7.09
7.07
6.77
6.76
6.75
6.74
6.74
6.73
5.11
5.09
5.08
5.06
5.05
5.03
5.01
3.73
1.57
1.56
1.21
1.20
1.19
1.18
2.11
1.97
1.98
1.91
1.00
0.96
8.69
14.43
f1 (ppm)

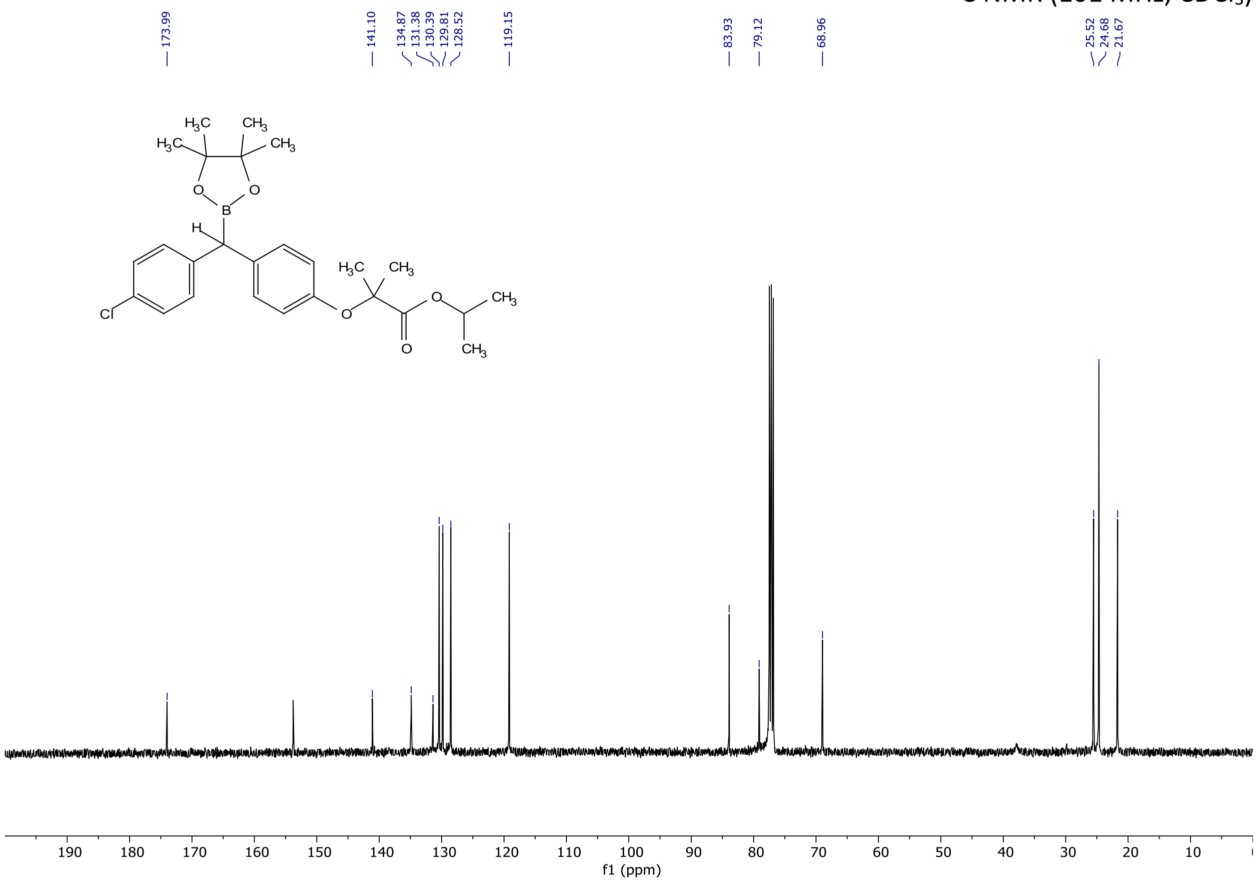

¹³C NMR (101 MHz, CDCl3)
173.99
141.10
134.87
131.38
130.39
129.81
128.52
119.15
83.93
79.12
68.96
25.52
24.68
21.67
f1 (ppm)

^{11}B NMR (128 MHz, $CDCl_3$)

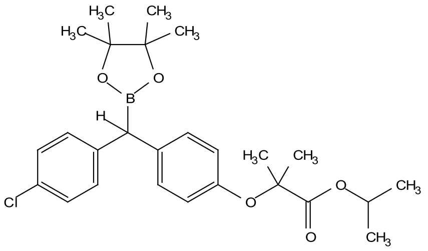

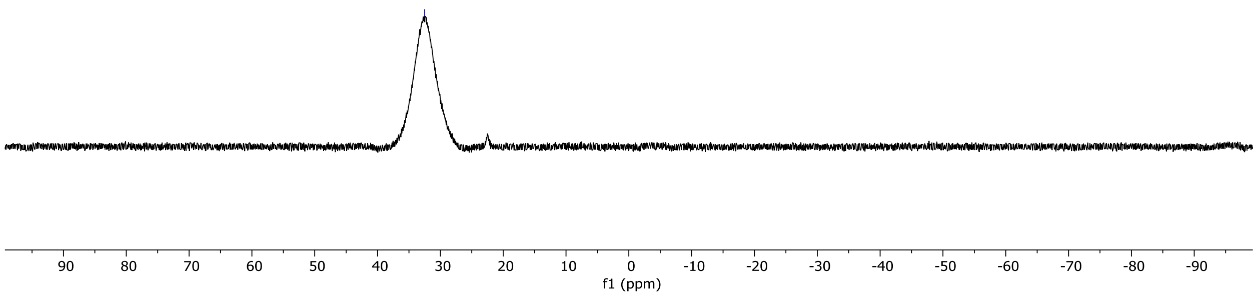

S10.74 **2af**

^{1}H NMR (400 MHz, CDCl$_3$)

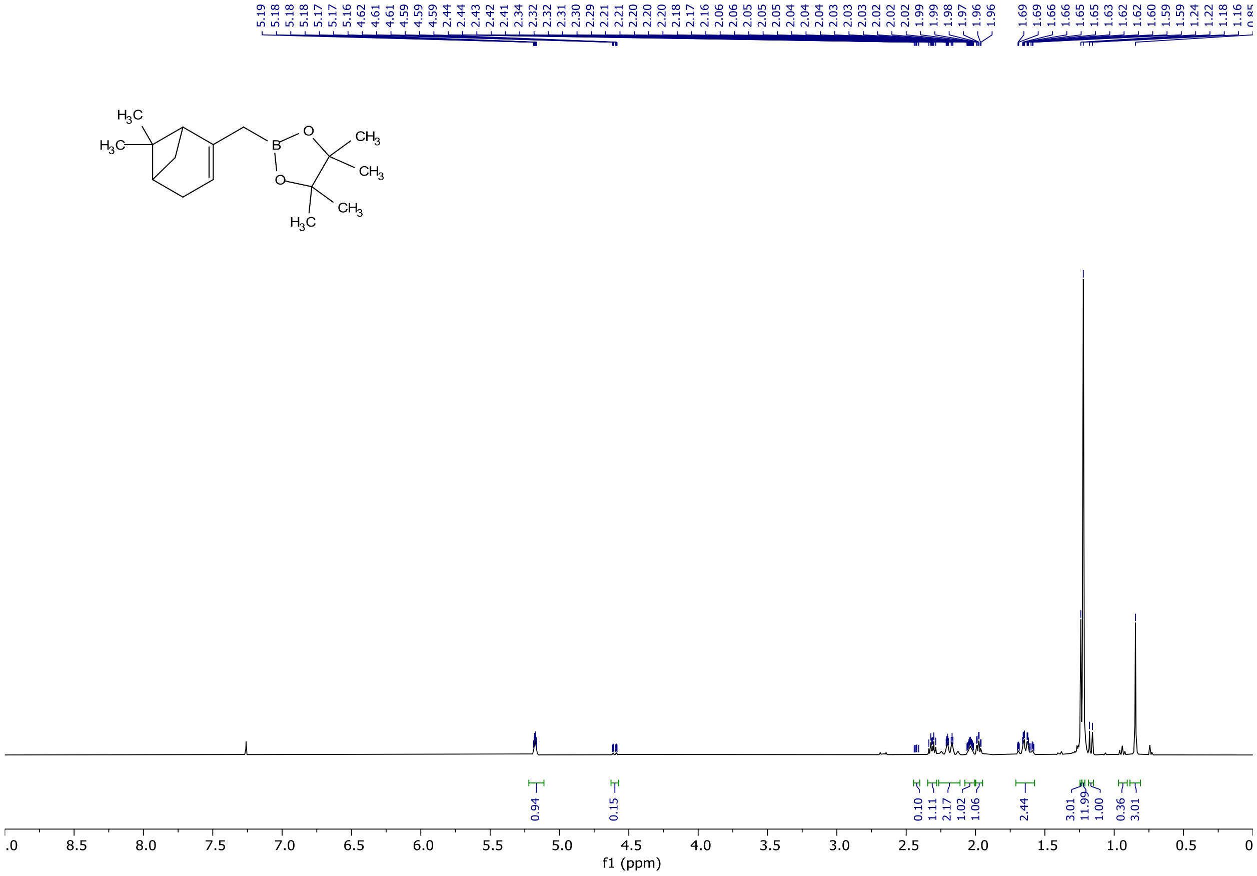

^{13}C NMR (101 MHz, CDCl$_3$)

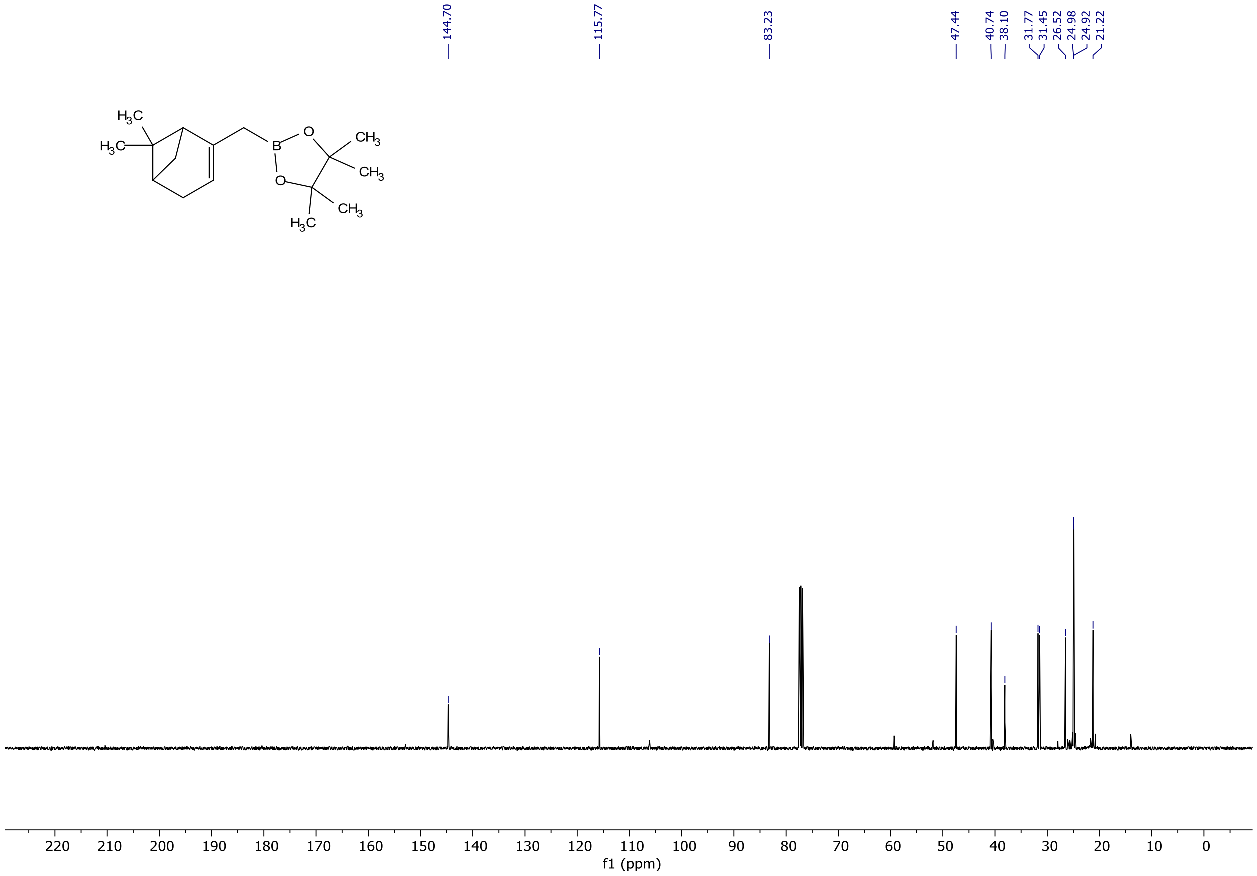

^{11}B NMR (128 MHz, $CDCl_3$)

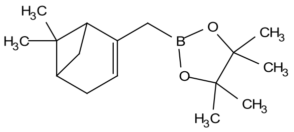

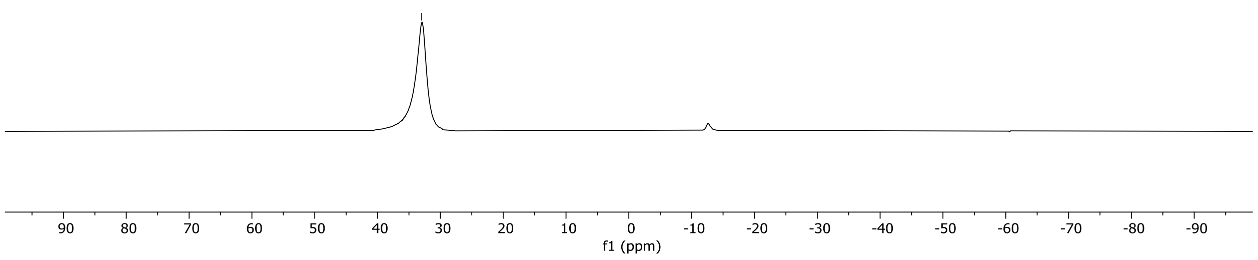

S10.75 2ag

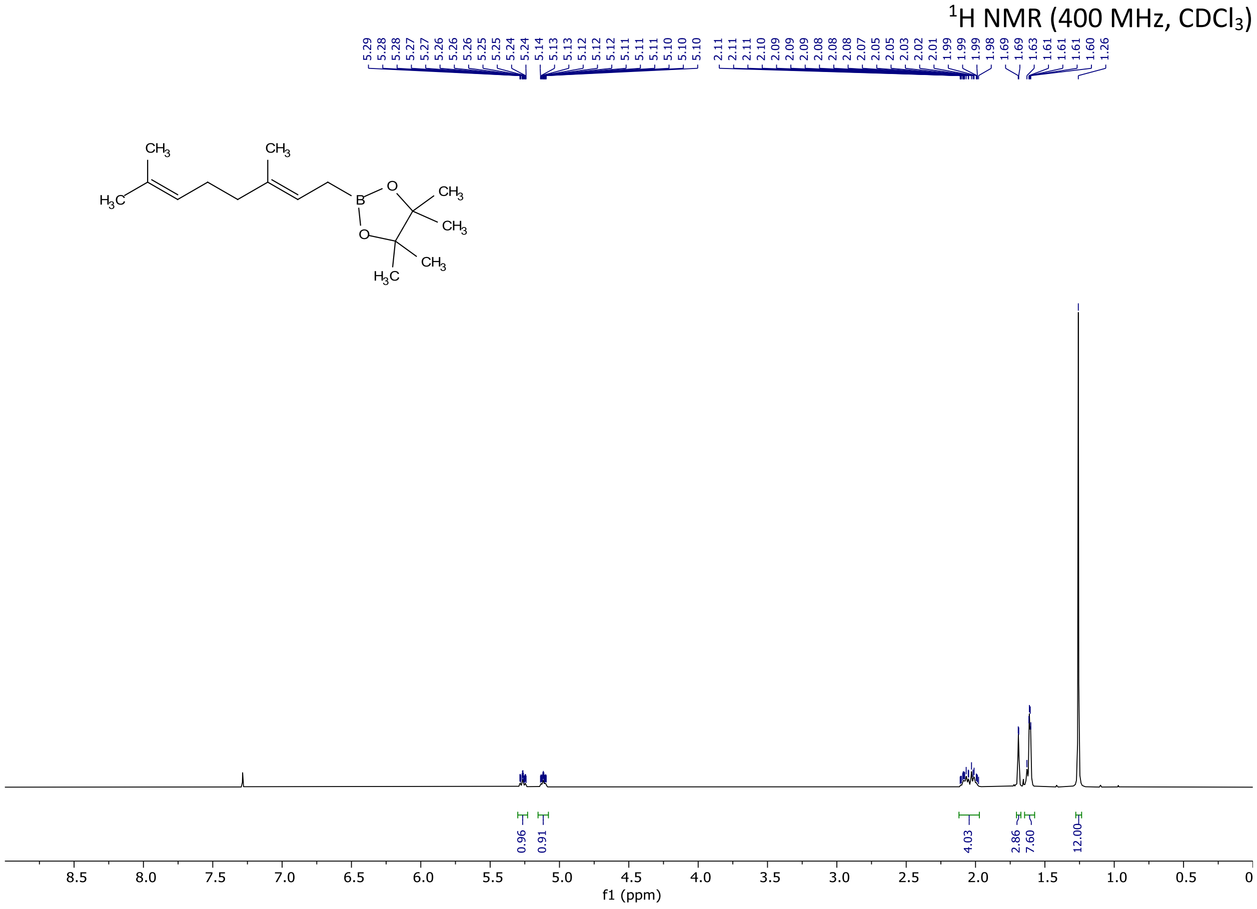

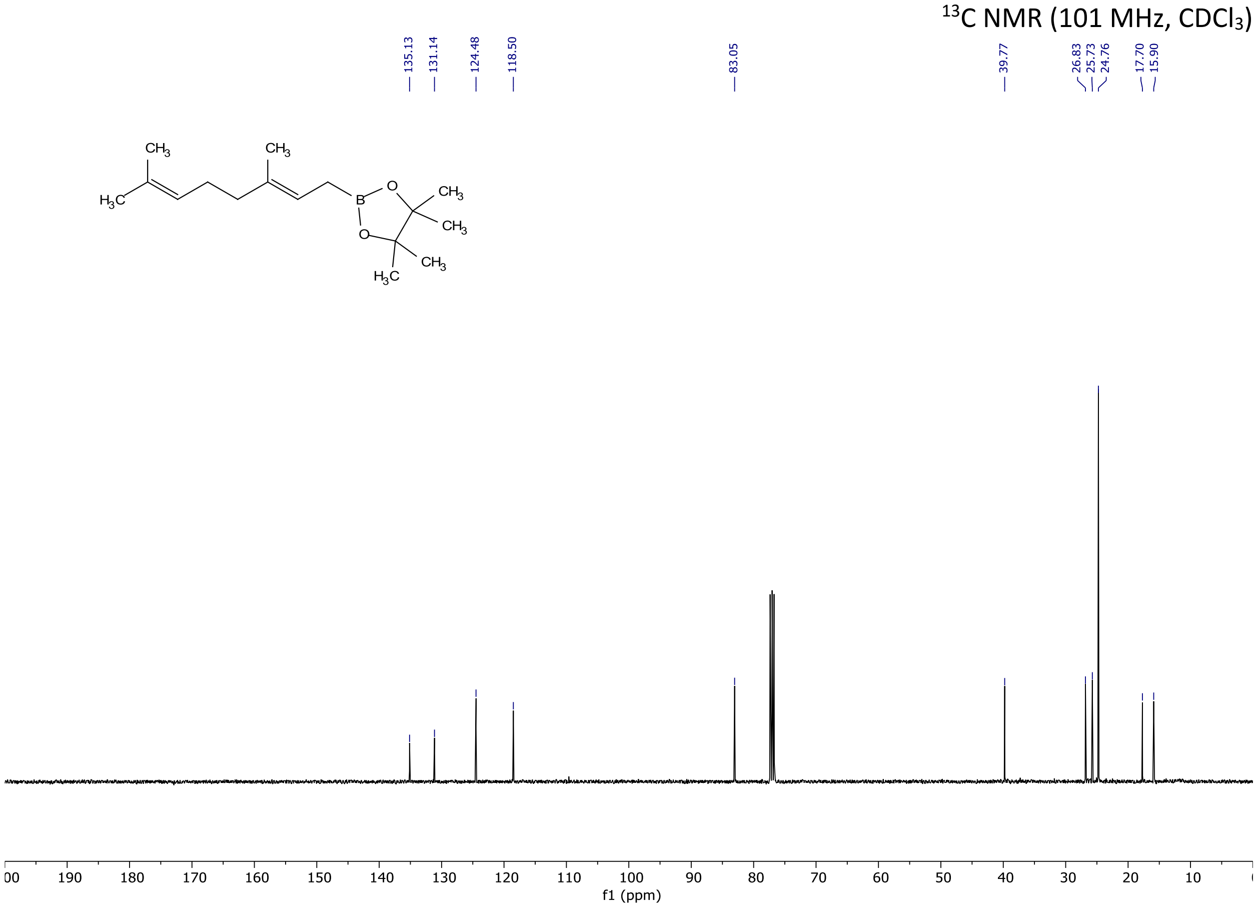

^{11}B NMR (128 MHz, $CDCl_3$)

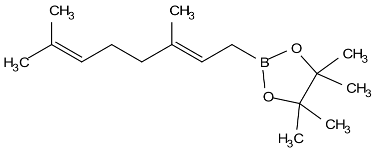

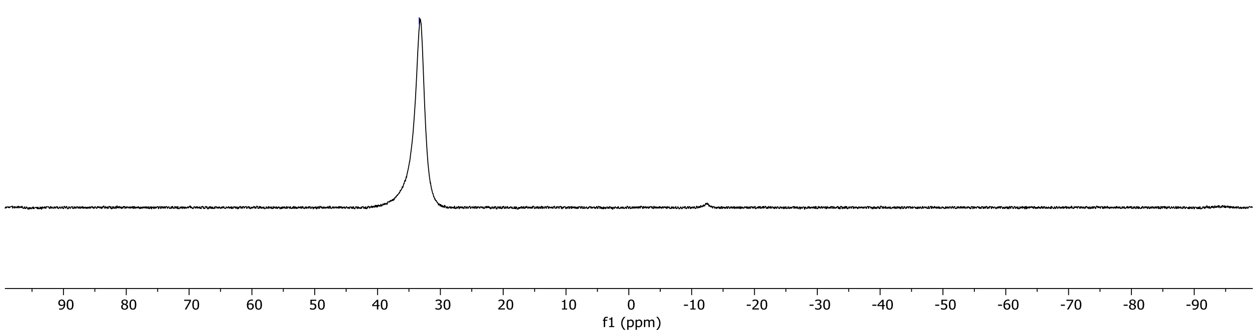

S10.76 2ah

^{1}H NMR (400 MHz, $CDCl_3$)

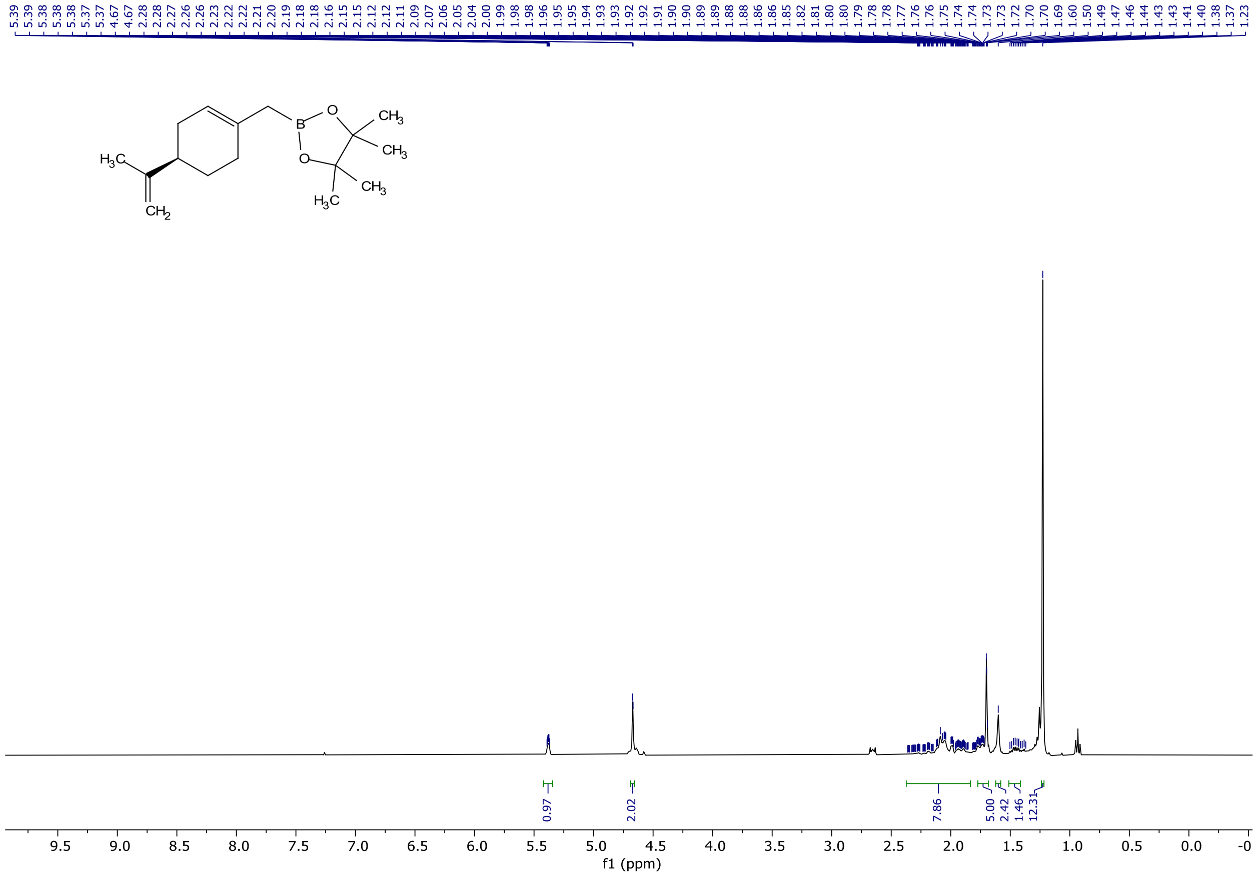

^{13}C NMR (101 MHz, $CDCl_3$)

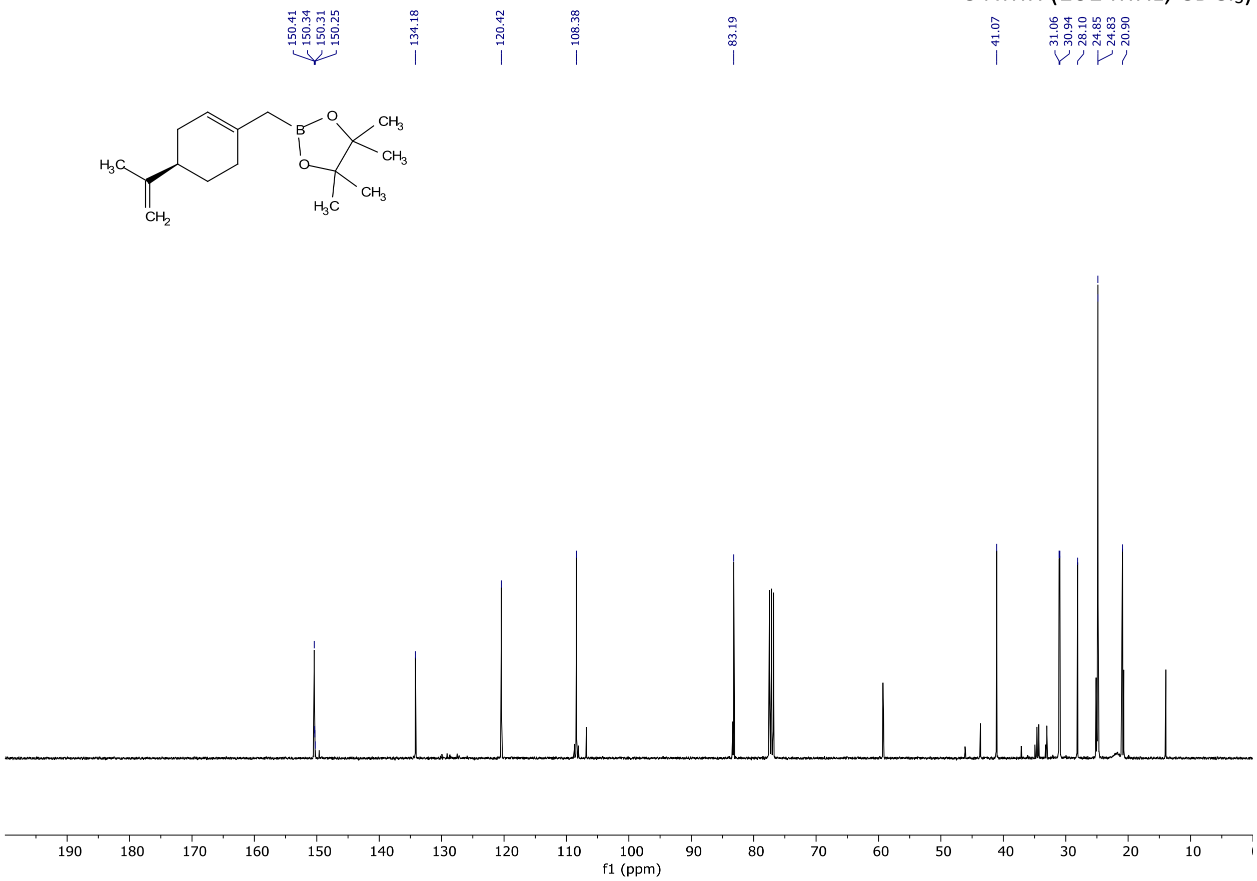

^{11}B NMR (128 MHz, $CDCl_3$)

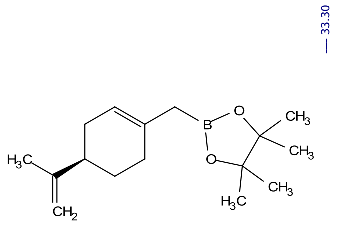

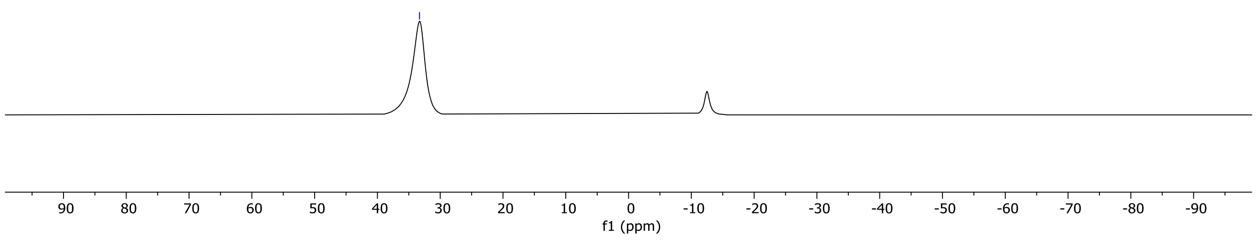

S10.77 2am

^{1}H NMR (400 MHz, $CDCl_3$)

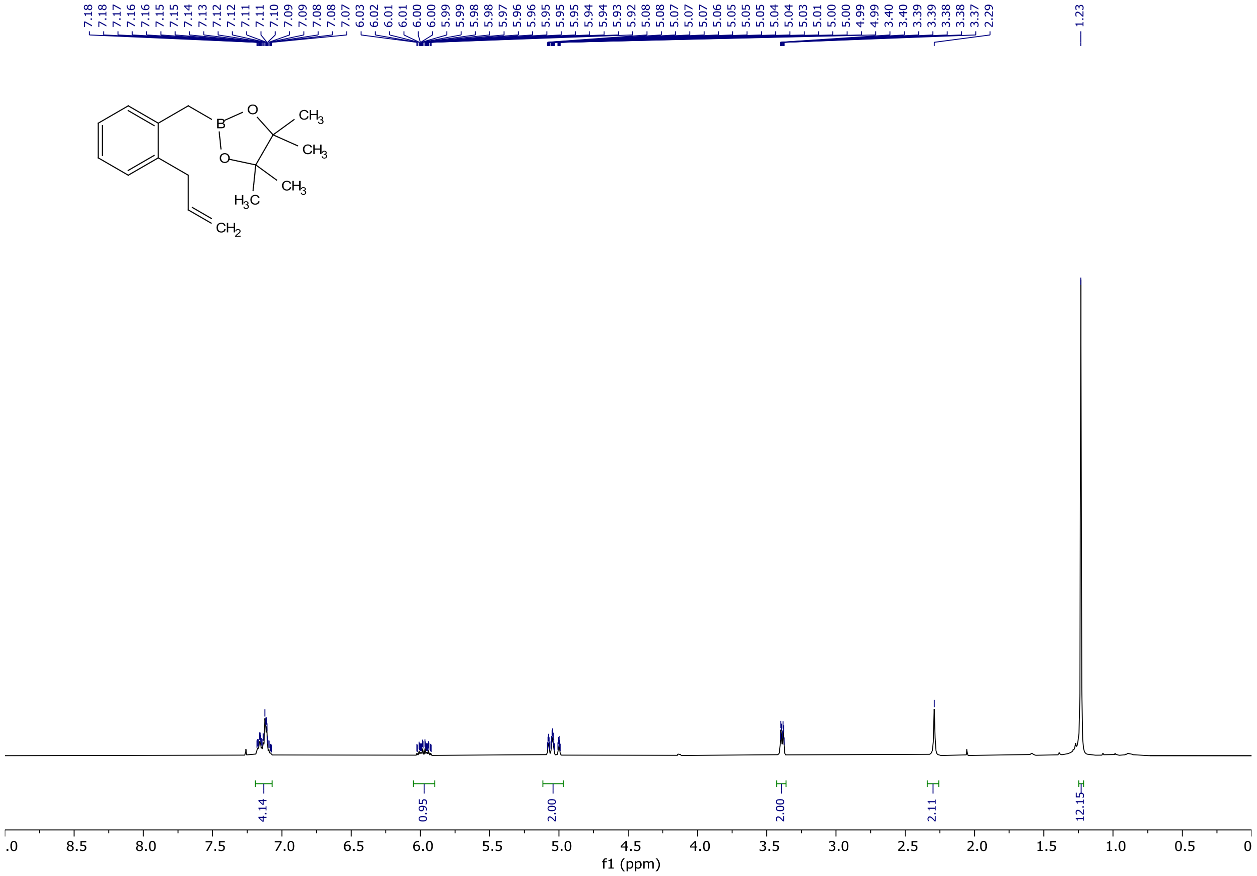

^{13}C NMR (101 MHz, $CDCl_3$)

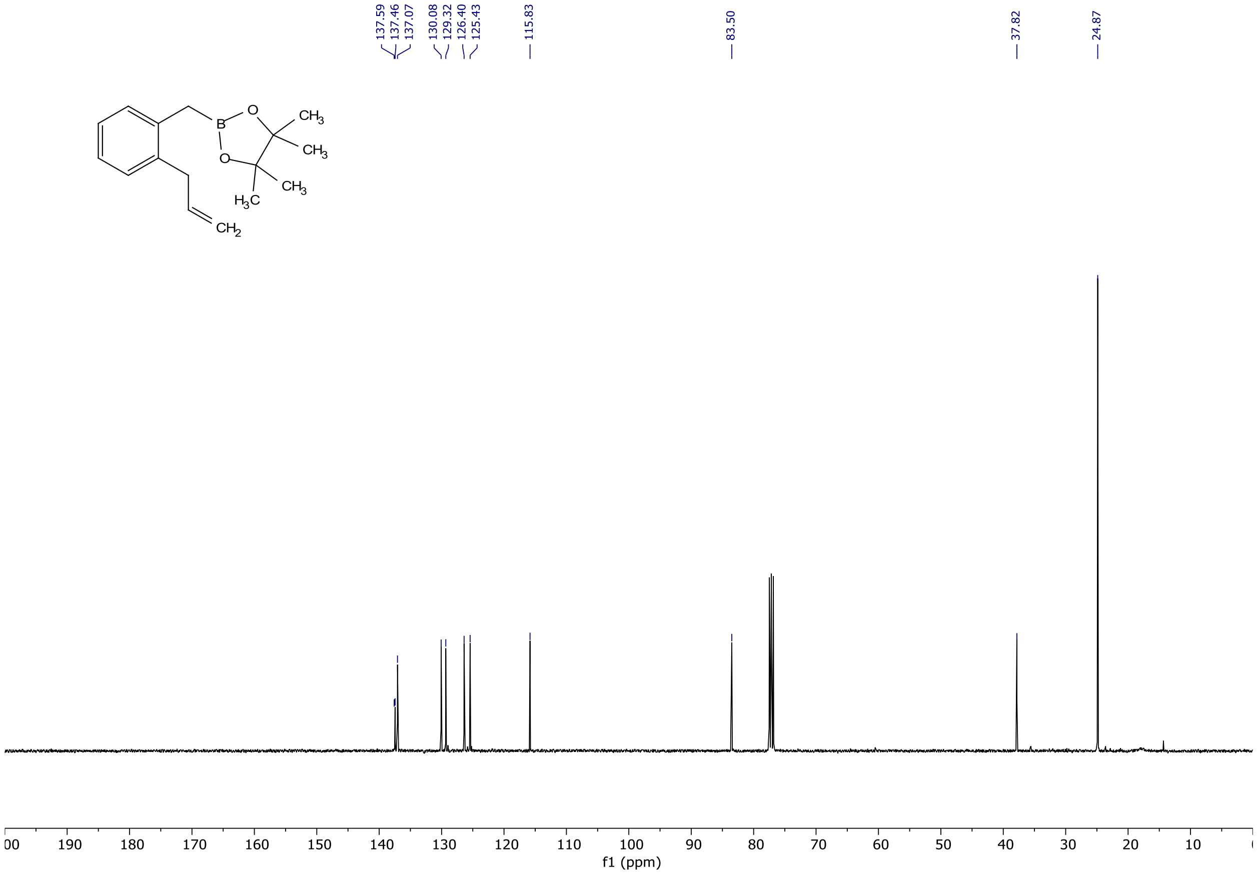

^{11}B NMR (128 MHz, $CDCl_3$)

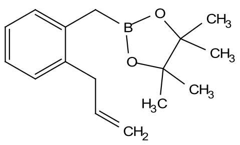

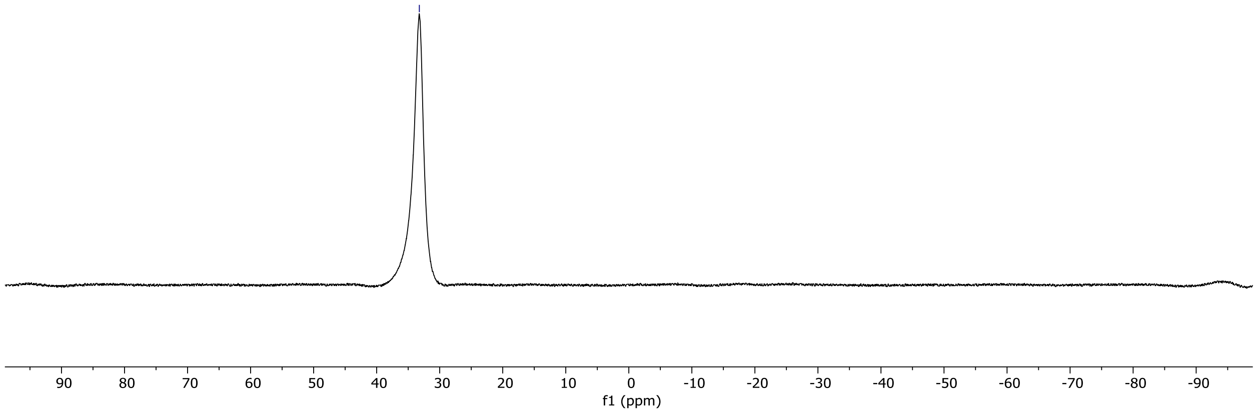

S10.78 3b

^{1}H NMR (400 MHz, $CDCl_3$)

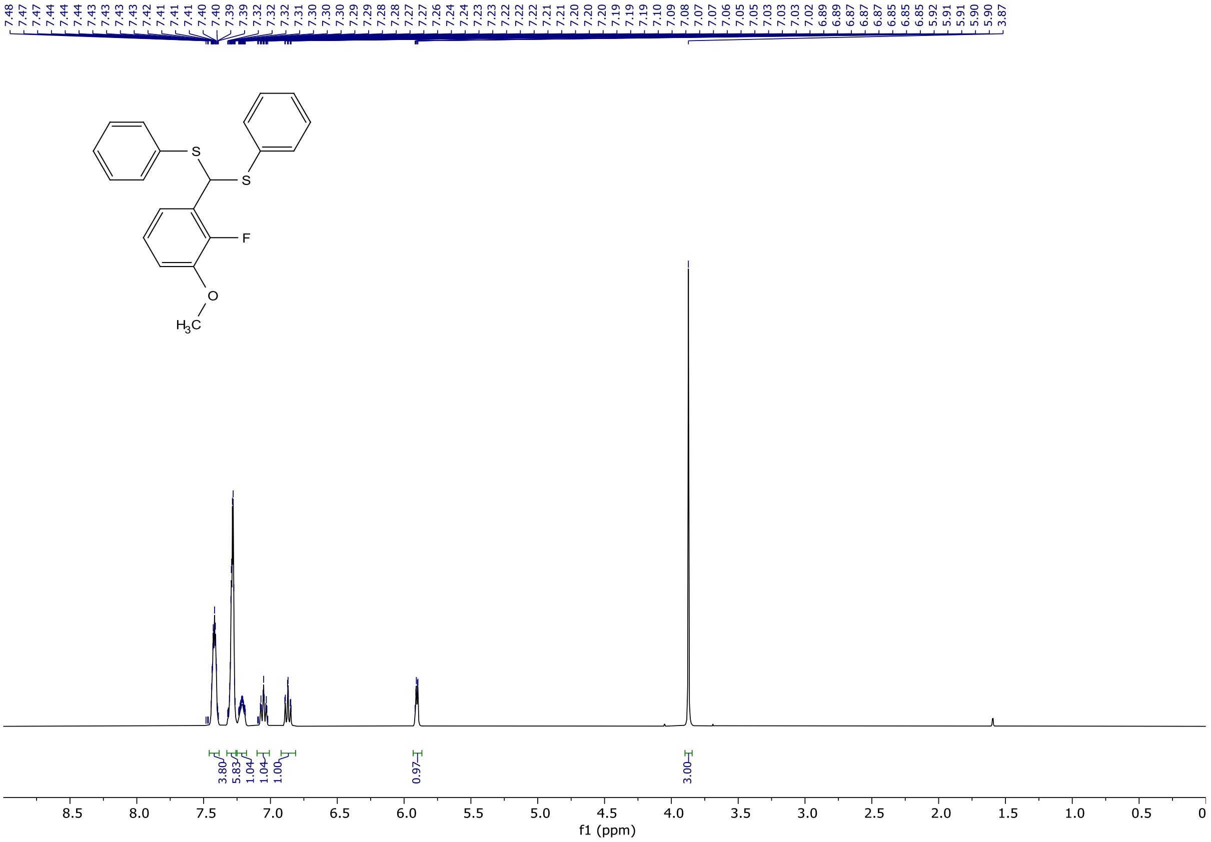

^{13}C NMR (101 MHz, $CDCl_3$)

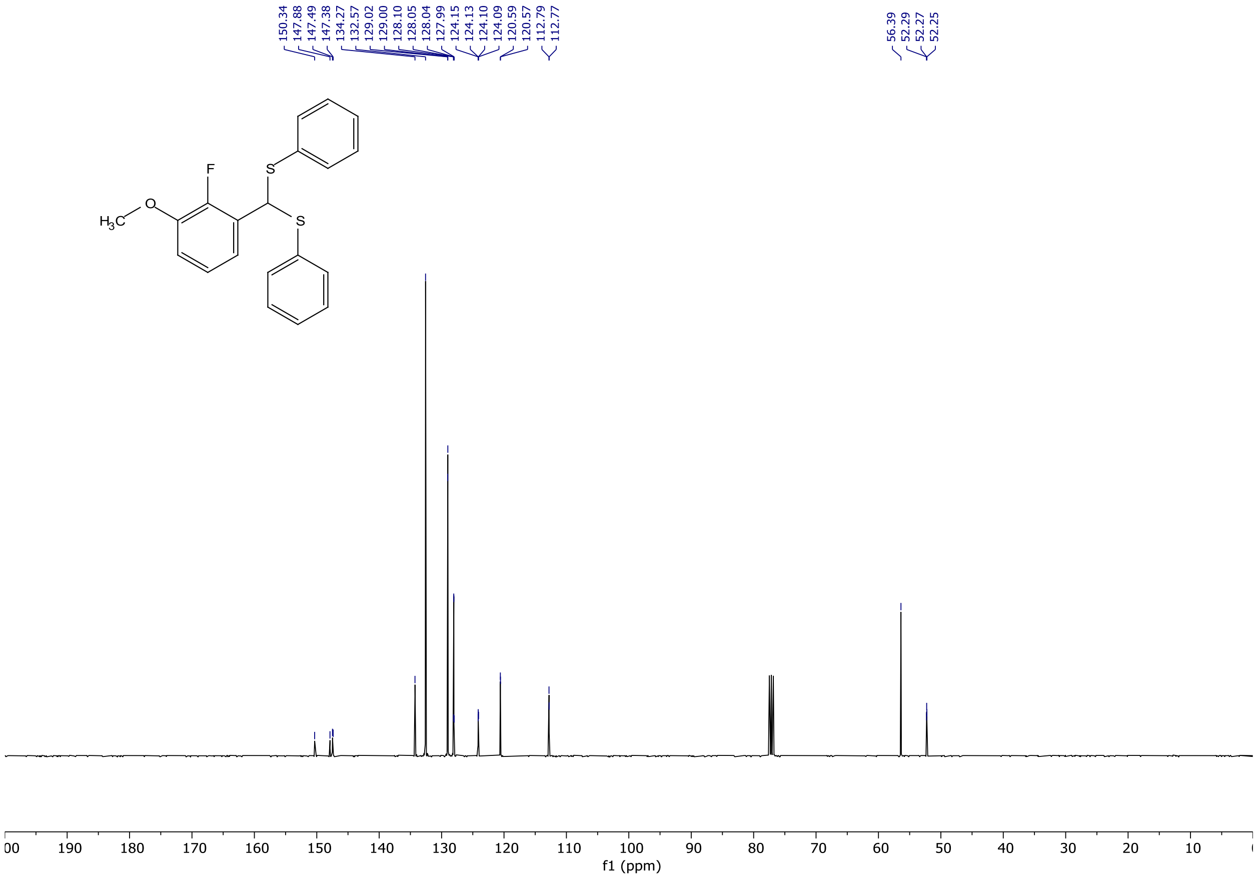
150.34
147.88
147.49
147.38
134.27
132.57
129.02
129.00
128.10
128.05
128.04
127.99
124.15
124.13
124.10
124.09
120.59
120.57
112.79
112.77
56.39
52.29
52.27
52.25
F
S
S
O
H3C
190
180
170
160
150
140
130
120
110
100
90
80
70
60
50
40
30
20
10
f1 (ppm)

^{19}F NMR (377 MHz, $CDCl_3$)

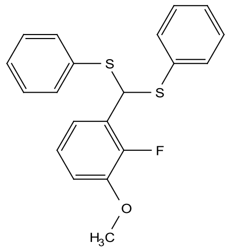

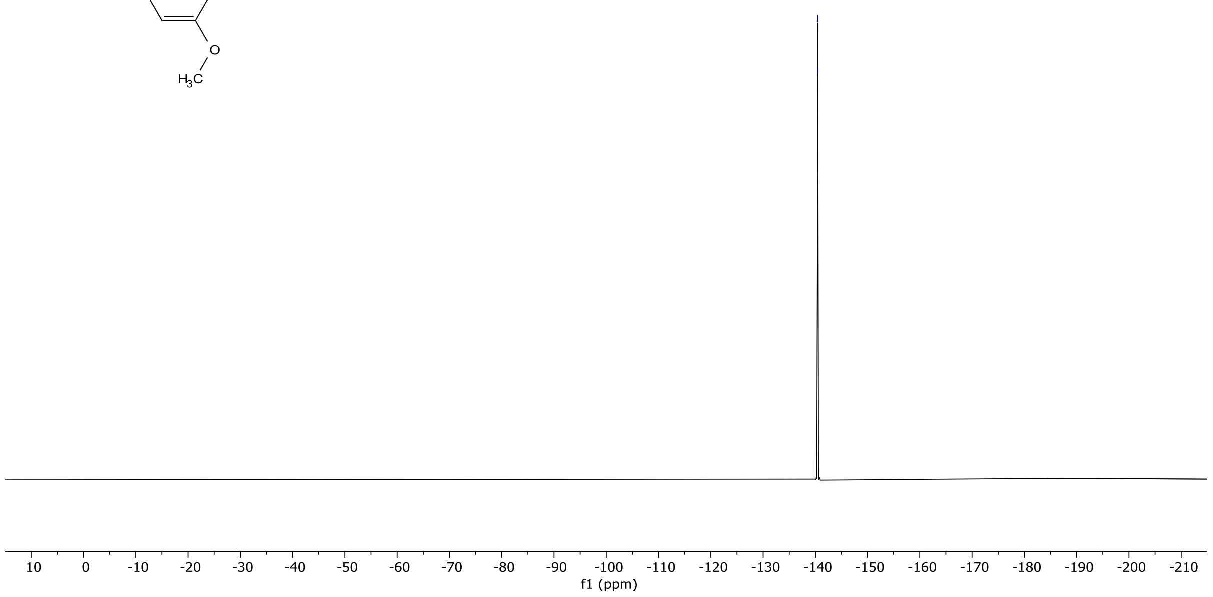

S10.79 3c

^1H NMR (400 MHz, $CDCl_3$)

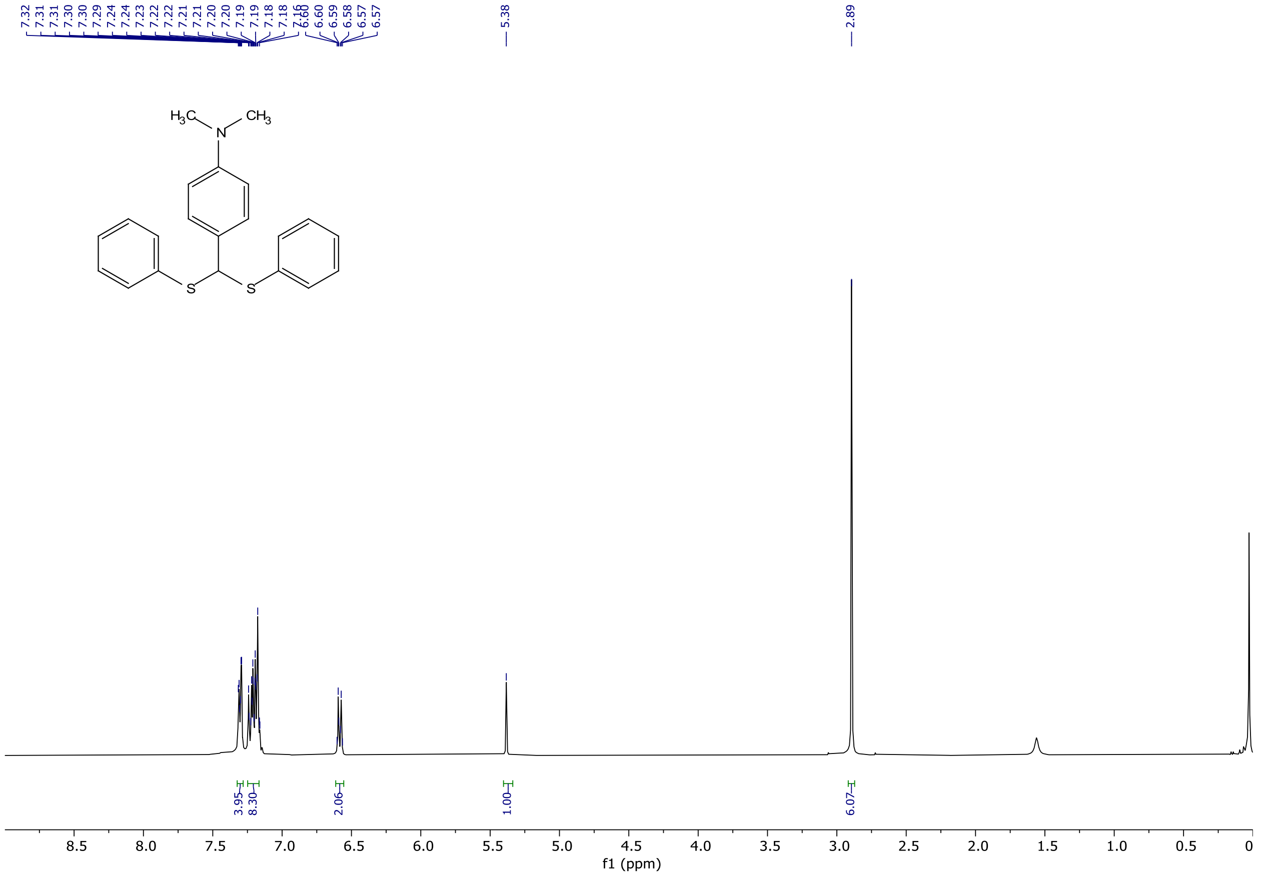

^{13}C NMR (101 MHz, $CDCl_3$)

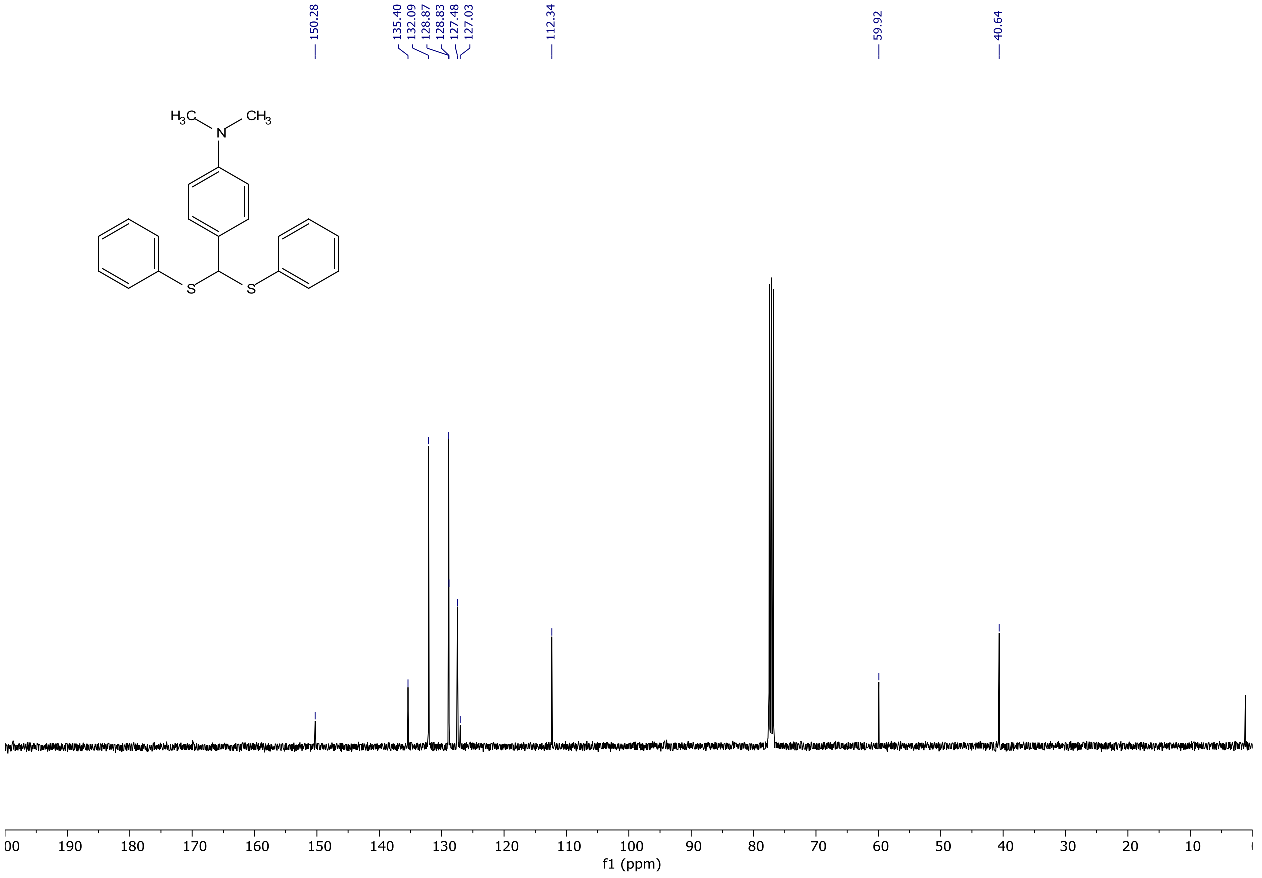

S10.80 **3d**

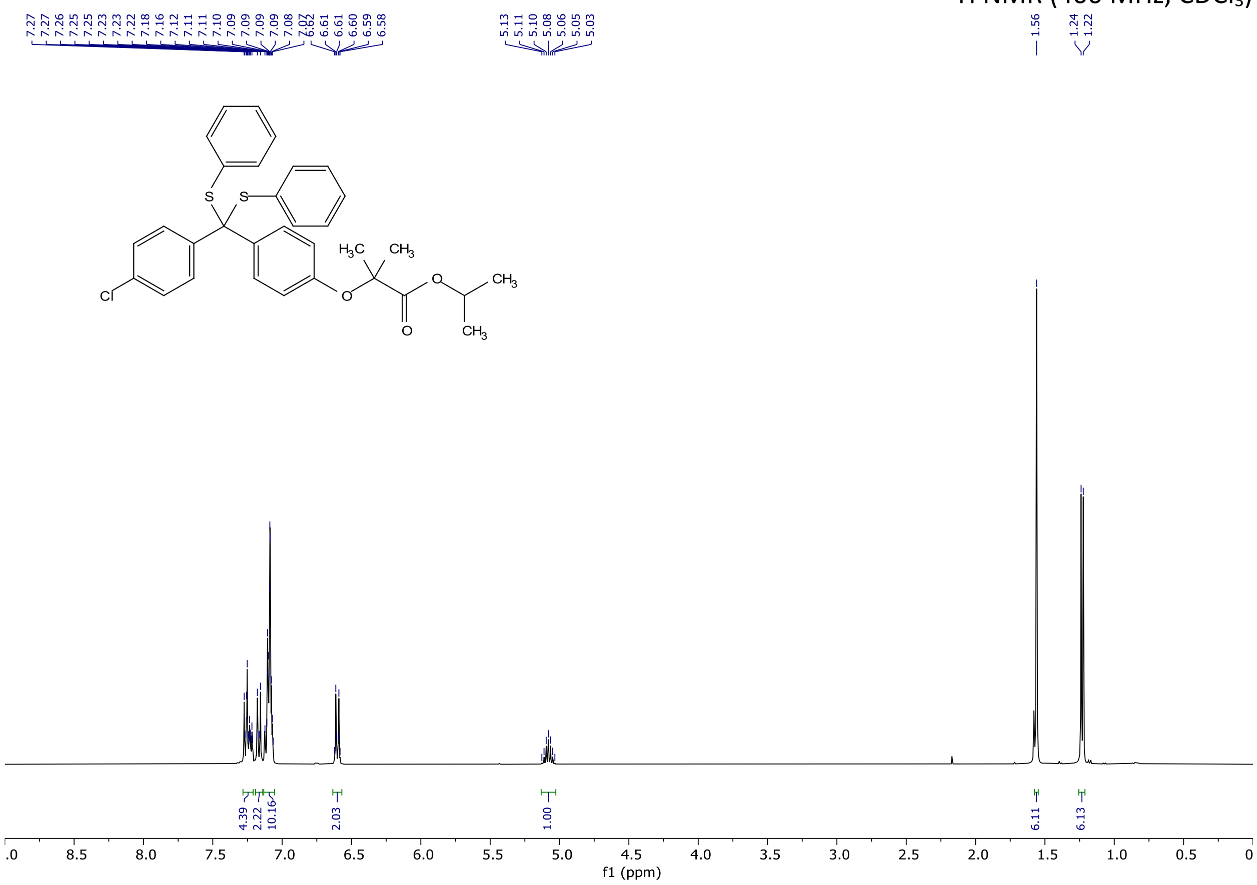

¹H NMR (400 MHz, CDCl₃)
f1 (ppm)

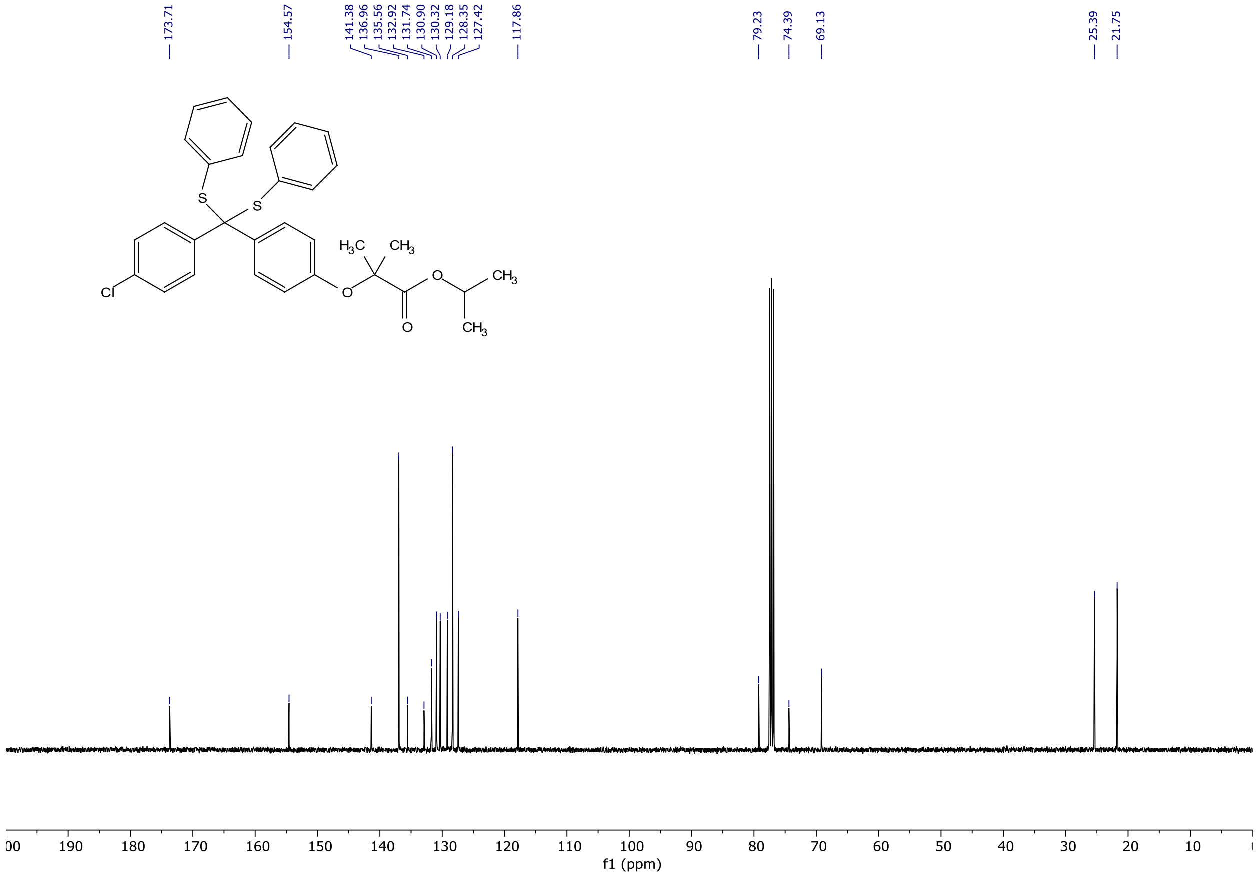

¹³C NMR (101 MHz, CDCl₃)
173.71
154.57
141.38
136.96
135.56
132.92
131.74
130.90
130.32
129.18
128.35
127.42
117.86
79.23
74.39
69.13
25.39
21.75
f1 (ppm)

S11 References

1. Chen, J., Lin, J.-H. & Xiao, J.-C. Dehydroxylation of alcohols for nucleophilic substitution. *Chem. Commun.* **54**, 7034–7037 (2018).
2. Rout, L., Sen, T. K. & Punniyamurthy, T. Efficient CuO-Nanoparticle-Catalyzed C[illegible]S Cross-Coupling of Thiols with Iodobenzene. *Angew. Chem. Int. Ed.* **46**, 5583–5586 (2007).
3. Kuzmin, J. *et al.* Electroreductive Desulfurative Transformations with Thioethers as Alkyl Radical Precursors *Angew. Chem. Int. Ed.* **62**, e202304272 (2023).
4. Li, L.-L., Gao, L.-X. & Han, F.-S. A straightforward synthesis of alkyl 1H-tetrazol-5-yl thioethers via a one-pot reaction of aldehydes and 1H-tetrazole-5-thiols mediated by N-tosylhydrazones. *RSC Adv.* **5**, 29996–30000 (2015).
5. Patra, A., Ghorai, S. K., De, S. R. & Mal, D. Regiospecific Synthesis of Benzo[b]fluorenones via Ring Contraction by Benzil-Benzilic Acid Rearrangement of Benz[a]anthracene-5,6-diones. *Synthesis* **2006**, 2556–2562 (2006).
6. Hazra, G. & Masarwa, A. Synthesis and Functionalization of Thiophosphonium Salts: A Divergent Approach to Access Thioether, Thioester, and Dithioester Derivatives. *Org. Lett.* **25**, 6396–6400 (2023).
7. Sorribes, I. & Corma, A. Nanolayered cobalt–molybdenum sulphides (Co–Mo–S) catalyse borrowing hydrogen C–S bond formation reactions of thiols or H2S with alcohols. *Chem. Sci.* **10**, 3130–3142 (2019).
8. Xiao, F., Yuan, S., Huang, H. & Deng, G.-J. Base-Promoted Three-Component One-Pot Synthesis of 3- (Thiomethyl)indoles with Paraformaldehyde under Aqueous Conditions. *Synlett* **29**, 2693–2696 (2018).
9. Chen, W. *et al.* Highly Conducting π-Conjugated Molecular Junctions Covalently Bonded to Gold Electrodes. *J. Am. Chem. Soc.* **133**, 17160–17163 (2011).

10. Croft, R. A., Mousseau, J. J., Choi, C. & Bull, J. A. Lithium-Catalyzed Thiol Alkylation with Tertiary and Secondary Alcohols: Synthesis of 3-Sulfanyl-Oxetanes as Bioisosteres. *Chem. Eur. J.* **24**, 818–821 (2018).
11. Tsay, S.-C. *et al.* Direct Synthesis of Allyl Sulfides from Allyl Alcohols and Thiols. *Synthesis* **1993**, 329–334 (1993).
12. Crich, D. *et al.* Dechalcogenative Allylic Selenosulfide and Disulfide Rearrangements: Complementary Methods for the Formation of Allylic Sulfides in the Absence of Electrophiles. Scope, Limitations, and Application to the Functionalization of Unprotected Peptides in Aqueous Media. *J. Am. Chem. Soc.* **129**, 10282–10294 (2007).
13. Bhawar, R., Saini, S., Patil, K. S., Nagaraju, D. H. & Bose, S. K. Synthesis of Alkyl and Aryl Boronate Esters via CeO2-Catalyzed Borylation of Alkyl and Aryl Electrophiles Including Alkyl Chlorides. *J. Org. Chem.* **88**, 16270–16279 (2023).
14. Zhang, Q. *et al.* Decarboxylative Borylation of Stabilized and Activated Carbon Radicals. *Angew. Chem. Int. Ed.* **59** 21875–21879 (2020).
15. Li, H., Wang, L., Zhang, Y. & Wang, J. Transition-Metal-Free Synthesis of Pinacol Alkylboronates from Tosylhydrazones. *Angew. Chem. Int. Ed.* **51**, 2943–2946 (2012).
16. Yin, C., Liao, Y., He, B., Li, H. & Su, W. Rh-Catalyzed Direct Decarbonylative Borylation of Carboxylic Acids. *Chem. Eur. J.* **29**, e202300184 (2023).
17. Ghosh, P., Schoch, R., Bauer, M. & Jacobi von Wangelin, A. Selective Benzylic CH-Borylations by Tandem Cobalt Catalysis. *Angew. Chem. Int. Ed.* **61**, e202110821 (2022).
18. Bastick, K. A. C. & Watson, A. J. B. Pd-Catalyzed Homologation of Arylboronic Acids as a Platform for the Diversity-Oriented Synthesis of Benzylic C–X Bonds. *Synlett* **34**, 2097–2102 (2023).

19. Guan, W., Chang, Y. & Lin, S. Electrochemically Driven Deoxygenative Borylation of Alcohols and Carbonyl Compounds. *J. Am. Chem. Soc.* **145**, 16966–16972 (2023).
20. Ji, P. *et al.* Cerium-Hydride Secondary Building Units in a Porous Metal–Organic Framework for Catalytic Hydroboration and Hydrophosphination. *J. Am. Chem. Soc.* **138**, 14860–14863 (2016).
21. Zhang, H., Hagihara, S. & Itami, K. Making Dimethylamino a Transformable Directing Group by Nickel-Catalyzed C-N Borylation. *Chem. Eur. J.* **21** 16796–16800 (2015).
22. Selander, N., Paasch, J. R. & Szabó, K. J. Palladium-Catalyzed Allylic C−OH Functionalization for Efficient Synthesis of Functionalized Allylsilanes. *J. Am. Chem. Soc.* **133**, 409–411 (2011).
23. Reller, C. & Mertens, F. The Recycling of Spent Ammonia Borane with HBr/AlBr3 and Other HX/AlX3-Based Schemes. *ChemPlusChem* **83**, 1013–1020 (2018).
24. Mykura, R. C. *et al.* Studies on the Lithiation, Borylation, and 1,2-Metalate Rearrangement of O-Cycloalkyl 2,4,6-Triisopropylbenzoates. *Angew. Chem. Int. Ed.* **60**, 11436–11441 (2021).
25. Shi, D., Wang, L., Xia, C. & Liu, C. Synthesis of Secondary and Tertiary Alkyl Boronic Esters by gem-Carboborylation: Carbonyl Compounds as Bis(electrophile) Equivalents. *Angew. Chem. Int. Ed.* **57**, 10318–10322 (2018).
26. Hu, J. *et al.* Nickel-Catalyzed Borylation of Aryl- and Benzyltrimethylammonium Salts via C–N Bond Cleavage. *J. Org. Chem.* **81**, 14–24 (2016).
27. Atack, T. C., Lecker, R. M. & Cook, S. P. Iron-Catalyzed Borylation of Alkyl Electrophiles. *J. Am. Chem. Soc.* **136**, 9521–9523 (2014).
28. Ghosh, P., Schoch, R., Bauer, M. & Jacobi von Wangelin, A. Selective Benzylic CH-Borylations by Tandem Cobalt Catalysis. *Angew. Chem. Int. Ed.* **61**, e202110821 (2022).
29. Becke, A. D. Density-functional thermochemistry. III. The role of exact exchange. *J. Chem. Phys.* **98**, 5648–5652 (1993).
